# Genome-wide analysis of *Candida albicans* gene expression patterns during infection of the mammalian kidney

**DOI:** 10.1016/j.fgb.2008.10.012

**Published:** 2009-02

**Authors:** Louise A. Walker, Donna M. MacCallum, Gwyneth Bertram, Neil A.R. Gow, Frank C. Odds, Alistair J.P. Brown

**Affiliations:** Aberdeen Fungal Group, School of Medical Sciences, University of Aberdeen, Institute of Medical Sciences, Foresterhill, Aberdeen AB25 2ZD, UK

**Keywords:** *Candida albicans*, Infection, Genomics, Microarrays, Transcript profiling

## Abstract

Global analysis of the molecular responses of microbial pathogens to their mammalian hosts represents a major challenge. To date few microarray studies have been performed on *Candida albicans* cells derived from infected tissues. In this study we examined the *C. albicans* SC5314 transcriptome from renal infections in the rabbit. Genes involved in adhesion, stress adaptation and the assimilation of alternative carbon sources were up-regulated in these cells compared with control cells grown in RPMI 1640, whereas genes involved in morphogenesis, fermentation and translation were down-regulated. When we compared the congenic virulent *C. albicans* strains NGY152 and SC5314, there was minimal overlap between their transcriptomes during kidney infections. This suggests that much of the gene regulation observed during infections is not essential for virulence. Indeed, we observed a poor correlation between the transcriptome and phenome for those genes that were regulated during kidney infection and that have been virulence tested.

## 1 Introduction

*Candida albicans* is a major opportunistic fungal pathogen of humans (Odds, 1988; Calderone, 2002). In many healthy individuals *C. albicans* exists as a commensal in the oral cavity and the gastrointestinal and urogenital tracts, generating no obvious pathology. However, this fungus frequently causes a range of mucosal infections such as oral thrush and vaginitis (Ruhnke, 2002). In patients with compromised immune defences, *C. albicans* can establish bloodstream infections that can progress to deep-seated infections of major organs such as the kidney, liver and brain, many of which are fatal (Filler and Kullberg, 2002; Kullberg and Filler, 2002). Clearly the immune status of the host strongly influences the ability of *C. albicans* to cause disease (Casadevall and Pirofski, 2003). Nevertheless, understanding the changes in the fungus that are associated with, and contribute to, the development of tissue-damaging disease represents a major challenge in the field.

Multiple factors are thought to contribute to the virulence of *C. albicans*. Cell surface adhesins promote binding to, and possibly the penetration of, host tissue (Staab et al., 1999; Hoyer et al., 2007; Phan et al., 2007). Secreted proteinases, lipases and phospholipases are thought to provide nutrients and may promote invasion (Naglik et al., 2003; Schaller et al., 2005). Morphological transitions between yeast and (pseudo)hyphal growth forms have been predicted to promote the dissemination and penetration of *C. albicans* cells (Gow et al., 2002, 2003; Sundstrom, 2006), and the expression of some adhesins and secreted proteinases is coordinated with yeast-hypha morphogenesis (Hube et al., 1994; Staab et al., 1996; Argimón et al., 2007). High frequency phenotypic switching of *C. albicans* cells between distinct epigenetic states that express different metabolic, morphological and cell surface properties is associated with changes in virulence and might help the fungus evade host immune responses (Odds, 1997; Soll, 2002). Other properties of *C. albicans*, which are not virulence factors that interact directly with the host (Odds et al., 2003), contribute to pathogenicity. These include the metabolic flexibility to adapt to diverse niches in the host (Lorenz and Fink, 2001; Barelle et al., 2006), and robust stress responses that enhance fungal survival following attack by host immune defences (Wysong et al., 1998; Hwang et al., 2002; Fradin et al., 2005; Enjalbert et al., 2007).

Over a decade ago it was predicted that the relative contributions of specific virulence factors and fitness attributes change temporally and spatially during the establishment and progression of *C. albicans* infections (Odds, 1994). This idea has been reinforced by data from a number of laboratories on the expression of virulence-associated genes in a range of infection models. These studies have generally focused on specific genes that are presumed or known to be important for the virulence of *C. albicans.* Members of the *SAP* (secreted aspartyl proteinase), *LIP* (lipase) and *ALS* (agglutinin-like sequence) gene families are regulated in a stage- and niche-specific fashion (reviewed by Brown et al., 2007). More recently, the advent of microarray technologies has allowed the generation of unbiased global views of *C. albicans* gene regulation that make no presumptions about the responses of this pathogen to specific stimuli. Transcript profiling of *C. albicans* has been performed on a range of *in vitro* conditions such as serum-stimulated morphogenesis, during phenotypic switching and biofilm formation, exposure to various stresses, and carbon and nitrogen starvation (Nantel et al., 2002; Lan et al., 2002; Enjalbert et al., 2003, 2006; Lorenz et al., 2004; Garcia-Sanchez et al., 2004; Hromatka et al., 2005). More interestingly from a virulence perspective, expression profiling has been performed on *C. albicans* cells following exposure to macrophages, neutrophils and blood fractions (Rubin-Bejerano et al., 2003; Lorenz et al., 2004; Fradin et al., 2003, 2005), and in *ex vivo* infection models such as reconstituted human epithelium and perfused pig liver (Thewes et al., 2007; Zakikhany et al., 2007). These studies have provided new insights into *C. albicans-*host interactions, highlighting the importance of metabolic and stress adaptation in the fungus, as well as classical virulence attributes.

The major challenge has been to extend these analyses into animal models of systemic infection since these are thought to best reflect clinical systemic infections. Few studies have been published because the transcript profiling of *C. albicans* from infected tissues presents significant technical challenges (reviewed by Brown et al., 2007). We address two of these technical challenges in this paper. The first is the need to generate sufficient fungal biomass for a microarray study. Previous expression profiling studies of *C. albicans* cells infecting mouse kidney and liver used various amplification strategies to increase hybridization signals from relatively small amounts of biomass (Andes et al., 2005; Thewes et al., 2007). We have avoided cDNA amplification by generating larger amounts of biomass in the rabbit model of systemic candidiasis. The second challenge is the “contamination” of fungal biomass with the mammalian tissue it is intimately associated with. Significant contamination has prevented the analysis of fungal samples (Thewes et al., 2007). We have addressed this by developing methods for the enrichment of fungal cells from infected tissues. We compare our expression profiling of *C. albicans* cells with data from other infection models, and discuss the relationship between gene regulation and gene essentiality with respect to the virulence of this major pathogen.

## 2 Materials and methods

### 2.1 Strains and growth conditions

The *C. albicans* clinical isolate SC5314 (Gillum et al., 1984) and its congenic derivative NGY152 were used in this study. NGY152 is CAI4 (*ura3::λimm434/ura3::λimm434*: Fonzi and Irwin, 1993) transformed with CIp10 (*URA3*: Murad et al., 2000). *C. albicans* was grown in the yeast form at 30 ^o^C in YPD (1% yeast extract, 2% mycological peptone, 2% glucose: Sherman, 1991). To form a mixture of hyphae and pseudohyphae, *C. albicans* was grown overnight at 37 ^o^C in NGY (0.1% Neopeptone, 0.4% glucose and 0.1% yeast extract), washed and resuspended in RPMI 1640 at 37 ^o^C (CLSI, 2002).

### 2.2 Preparation of fungal cells for transcript profiling

To prepare *C. albicans* cells from infected kidneys, strains were grown overnight in NGY at 30 °C, washed twice by centrifugation and resuspension in sterile saline, and injected into the marginal ear veins of male NZ white rabbits weighing 2.5 ± 0.5 kg at a dose of 1–4 × 10^7^ yeast cells/kg body weight. Inoculum sizes were confirmed by counting viable cells (cfus). The rabbits were given food and water *ad libitum*. Animal experimentation was done in full conformity with the laws and requirements of the UK Home Office. Three days after infection the rabbits were terminated by intravenous injection of sodium pentobarbitone and the abdomen rapidly opened. Both kidneys were removed, halved longitudinally, and the capsules peeled off and discarded. Slices of cortical tissue, where white microabscesses characteristic of *C. albicans* infection were seen, were shaved with a sterile scalpel directly into containers of liquid N_2_, to snap-freeze the lesion-rich tissue within 2.5 min of the animal’s death. Fungal burdens were measured by viable counting. Pieces of renal cortex were also fixed in formalin, and embedded in paraffin to prepare tissue sections (5 μm). Tissue sections were stained with periodic acid-Schiff’s reagent.

Tissue slices from a single rabbit kidney were combined and fixed in a total of 56 ml RLT buffer (Qiagen, West Sussex, UK) and homogenized for 30 s with an Ultra-Turrax homogeniser with 20 mm probe. Homogenate was divided into 7 ml aliquots and each was layered onto a sucrose gradient comprising 2 ml 20% sucrose, 2 ml 40% sucrose and 2 ml 60% sucrose. Gradients were centrifuged at 300*g* for 30 min with the brake switched off. The layer enriched with fungal material, between the 40% and 60% sucrose shelves, was transferred to microcentrifuge tubes and centrifuged at 13,000*g* for 10 min. The cell pellets were combined by washing in 1 ml of RLT lysis buffer, and RNA extracted immediately. Microscopic analyses of these pellets indicated that this sucrose gradient fractionation protocol is not selective with regard to *C. albicans* cell morphology. Yeast, pseudohyphal and hyphal cells were isolated.

To prepare control cells, *C. albicans* was grown overnight in NGY at 30 °C, resuspended in RPMI 1640, grown at 37 ^o^C for 6 h, and harvested by centrifugation. These exponentially growing cells were either immediately frozen in liquid N_2_, or subjected to sucrose density gradient fractionation before they were frozen, as described above. Other control *C. albicans* cells were prepared by growth in YPD at 30 °C to an OD_600_ of 0.6, before flash freezing in liquid N_2_, as described above.

### 2.3 RNA extraction

RNA was extracted from *C. albicans* cells isolated from rabbit kidneys by procedures modified from those of Hayes et al. (2002). Briefly, cell pellets were resuspended in 500 μl TRIzol reagent (Invitrogen Ltd., Paisley, UK). Glass beads (500 μl) were added and cells were disrupted with a Fastprep cell breakage machine (Thermo Savant, Middlesex, UK) run for three 30 s cycles at 6.5 m/s with chilling on ice for 1 min in between. Samples were centrifuged for 10 min at 12,000*g*, the supernatants extracted with chloroform, and the RNA precipitated with 0.5 volumes of isopropanol for 20 min at room temperature. Precipitates were harvested by centrifugation, and washed twice with ice-cold 70% ethanol. Pellets were resuspended in 200 μl diethylpyrocarbonate-treated water and RNA re-precipitated with 200 μl LiCl precipitation buffer (Ambion, TX, USA) overnight at −20 ^o^C. RNA was harvested by centrifugation, washed twice with ice-cold ethanol, and resuspended in 25 μl DEPC water. RNA was extracted from control samples as described above, except that these cells were sheared mechanically using a microdismembrator (Braun, Melsungen, Germany). The integrity of all RNA samples was confirmed by gel electrophoresis before use in microarray experiments (Supplementary data).

### 2.4 Transcript profiling

*Candida albicans* transcript profiling was performed as previously described (Copping et al., 2005; Enjalbert et al. 2006). Cy3- and Cy5- labeled cDNAs were prepared from total RNA preparations and hybridized with *C. albicans* whole genome microarrays (Eurogentec, Seraing, Belgium). The microarrays were scanned with a ScanArray Lite scanner (Perkin–Elmer Life Sciences, Beaconsfield, UK) at a resolution of 10 μM. Signals on the slides were located with the ScanArray 4000 Microarray Analysis System and quantified with QuantArray software (version 2.0). Approximately 85% of *C. albicans* genes gave expression levels above background levels in our experiments. The data were normalized with the Lowess algorithm and analysed with Genespring software (Silicon Genetics, Redwood City, CA). Genes were viewed as significantly induced or repressed if they were up- or down-regulated by 2-fold or more in three of four array experiments, and if they passed statistical filtering using SAM software using a false discovery rate of <1% (significance analysis of microarrays; Tusher et al., 2001). The complete datasets are available in the Supplementary data and at ArrayExpress (www.ebi.ac.uk/microarray).

### 2.5 Real-time PCR

For qRT-PCR, samples were incubated at room temperature for 15 min using 2 μg RNA, 2 μl DNase I buffer (Invitrogen), 1.5 μl DNase I and 1.5 μl RNase OUT (Invitrogen) in a 20 μl reaction mix to remove any contaminating DNA. cDNA was prepared using Superscript II (Invitrogen) as per the manufacturer’s protocol. Optimization of amplification efficiency and real-time RT-PCR SYBR green assays were carried out as described by Avrova et al. (2003). The constitutively expressed gene *EFB1* was used as a control for all reactions. The amplification efficiency of the endogenous control and the genes of interest were found to be equivalent, thereby allowing the use of the comparative Ct method (ΔΔCt), which allowed comparison of gene expression levels *in vivo* relative to expression levels *in vitro* (as per the manufacturers instructions; DyNAmo SYBR Green qPCR Kits). Calculations and statistical analyses were carried out as described in ABI PRISM 7700 Sequence Detection System User Bulletin 2 (Applied Biosystems, USA).

## 3 Results and discussion

### 3.1 Preparation of fungal biomass from infected tissue

Our first goal was to extract fungal RNA from infected renal tissue in quantities sufficient for transcript profiling. Gene expression within fungal lesions might change rapidly following the termination of the animal. Therefore, we only analysed lesions that had been frozen in liquid N_2_ within 2.5 min of death, and used procedures designed to fix the fungal transcriptome throughout processing.

To evaluate the speed of our fixation methods we measured temporal loss of viability following the addition of fixative. *C. albicans* SC5314 cells were added to the fixation buffer and cell viability determined at various intervals thereafter by plating onto YPD medium. No viable *C. albicans* cells were recovered after 15 s of fixation (the most rapid time point that was practical to measure), suggesting that our fixation methods were rapid and effective.

To examine the combined effects of fixation and sucrose density gradient fractionation on the *C. albicans* transcriptome, control *C. albicans* SC5314 cells were grown in RPMI 1640 and snap-frozen for transcript profiling. Cells from equivalent cultures were fixed for 15 or 30 min, subjected to density gradient fractionation, and harvested for transcript profiling. The expression profiles of these processed cells were compared against the control cells in three independent microarray experiments. The expression of only a small fraction of *C. albicans* genes in the processed cells differed from that of the unprocessed controls. Five genes (0.08% of the genome) were up-regulated, and seven genes (0.11%) were down-regulated after 15 min of fixation and subsequent centrifugation (Table 1). Three genes involved in carbon metabolism (*IDF1, PGI1, CIT1*) and two components of the F_1_F_0_-ATPase complex (*ATP1, ATP2*) and orf19.9556 were included in these gene sets. After 30 min of fixation, zero genes were reproducibly up-regulated, and only two genes were down-regulated (0.03% of the genome) in processed cells compared with unprocessed controls: orf19.9556 (0.45-fold change) and orf19.1287 (0.46-fold change). Neither of these genes has a known function. We conclude that this fixation protocol is rapid and effective, and had a minimal impact upon the *C. albicans* transcriptome.

To obtain adequate amounts of fungal biomass from infected tissues sufficient to generate significant microarray signals without RNA amplification steps, we worked with infected rabbits (mean kidney weight 25 g) instead of the more commonly used mouse model (mean kidney weight 0.17 g). Progression of infection in the rabbit is essentially the same as in the mouse, with primary involvement of the kidneys in both species (Hasenclever, 1959; Rippon and Anderson, 1978; Morrison et al., 2003). We used a relatively high intravenous challenge dose, to induce formation of profuse visible kidney lesions (microabscesses) within 3 days. The data from our preliminary experiments on fungal fixation, density gradient enrichment of fungal cells and RNA extraction confirm the suitability of our approach for the determination of expression profiles of *C. albicans* cells *in vivo*.

### 3.2 *In vivo* expression profiling of a clinical isolate

Having established procedures for the fixation and enrichment of *C. albicans* cells we then applied these methods to the analysis of expression profiling of *C. albicans* SC5314 cells harvested and enriched from rabbit kidney lesions. This fungal RNA was compared with control RNA from SC5314 cells growing exponentially in RPMI 1640. We used RPMI 1640-grown cells as the control (rather than YPD-grown cells, for example) because this tissue culture medium is generally considered to better reflect growth conditions *in vivo*. Therefore, we reasoned that a comparison with RPMI 1640 is more likely to reveal infection-associated changes in expression, rather than changes associated with transfer from a rich growth medium. This view was supported by expression profiling of cells grown in YPD and RPMI 1640, which revealed that a different subset of *C*. *albicans* genes is up regulated in YPD-grown cells compared with *in vivo-*grown cells, when compared with RPMI 1640-grown cells (Supplementary material).

**Fig. 1:**
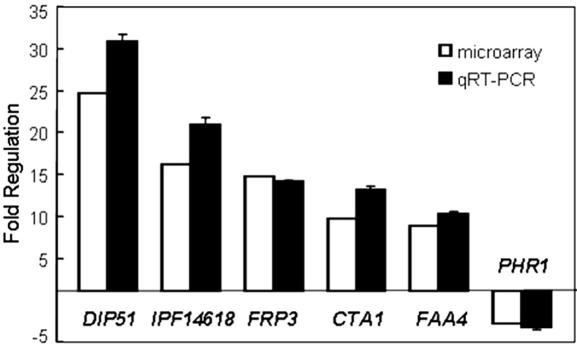
Comparison of qRT-PCR and microarray measurements of fold-regulation for six *C. albicans* SC5314 genes.

Relative to the RPMI 1640-grown control cells, 58 *C. albicans* genes were reproducibly induced by 2-fold or more in kidney lesions compared to the control cells in four independent replicate experiments (Table 2). These included genes involved in the assimilation of fatty acids and other alternative carbon sources (*ACO1*, *ACS1*, *CIT1*, *FAA4*, *MLS1*, *POX4*, *SDH12*), adhesion (*ALS1*, *ALS2*, *ALS4*), stress adaptation (*CTA1*, *ENA22*) and many genes of unknown function. In total, 50 genes were down-regulated in kidney lesions compared to control cells (Table 3). The down-regulated genes included functions associated with morphogenesis (*ECE1*, *HYR1*, *RBT5*), fermentation (*CDC19*, *HGT11*, *HXK2*, *HXT5*, *HXT61*, *HXT62*), protein biosynthesis (*BEL1*, *RPL18*, *RPS13*, *RPS21*) and genes associated with the cell surface (*ALS10*, *HYR1*, *IHD1*, *PGA54*, *PGA59*, *PGA10*, *PHR1*, *RBT5*, *SUN41*). To test the validity of these microarray datasets, we examined the expression levels of six genes by qRT-PCR. In all cases the qRT-PCR data displayed a high degree of concordance with the microarray data (Fig. 1).

The apparent down-regulation of hypha-specific genes was relative to the control RPMI 1640-grown control cells, and does not reflect a lack of expression of hypha-specific genes *in vivo*. Hypha-specific genes display dynamic changes in their expression levels during morphogenesis in *C. albicans* (e.g. *HYR1*: Bailey et al., 1996). Therefore, the observed regulation of hypha-specific genes might reflect temporal differences in the morphological development of the cells from kidney lesions compared with the control cells, as well as the heterogeneous morphologies of the fungal cells in these lesions. *C. albicans* SC5314 mainly formed pseudohyphae in RPMI 1640, whereas mixed populations of yeast, pseudohyphal and hyphal *C. albicans* cells were typically observed in sections from infected kidneys. Hyphal morphologies predominate in rabbit and mice kidneys, whereas pseudohyphal and yeast forms tend to predominate in guinea pig renal lesions (Odds et al., 2000).

The microarray data also indicated that *ALS* family members were differentially expressed in *C. albicans* cells infecting the kidney compared with cells growing in RPMI 1640. This is consistent with data from Hoyer’s group on differential *ALS* gene expression *in vitro* and *in vivo* (Hoyer, 2001; Green et al., 2005; Hoyer et al., 2007). Furthermore our data suggest that the *C. albicans* cells growing in RPMI 1640 and the mouse kidney differ with respect to their carbon metabolism. Most cells infecting the kidney are thought to assimilate carbon through glycolysis (Barelle et al., 2006). However, assuming that these changes in gene regulation reflect *bone fide* metabolic changes, our microarray data suggest that the population of *C. albicans* cells in kidney lesions are less glycolytically active than cells growing in RPMI 1640. Rather, alternative pathways of carbon assimilation such as fatty acid β-oxidation, the glyoxylate cycle and the TCA cycle may be more active in cells infecting the kidney. These pathways are known to be activated during phagocytosis by macrophages and neutrophils, and in a subset of cells infecting kidney tissue (Prigneau et al., 2003; Lorenz et al., 2004; Barelle et al., 2006). However they are not essential for virulence in the mouse model of systemic candidiasis (Barelle et al., 2006; Piekarska et al., 2006; Ramirez and Lorenz, 2007; Zhou and Lorenz, 2008).

**Fig. 2:**
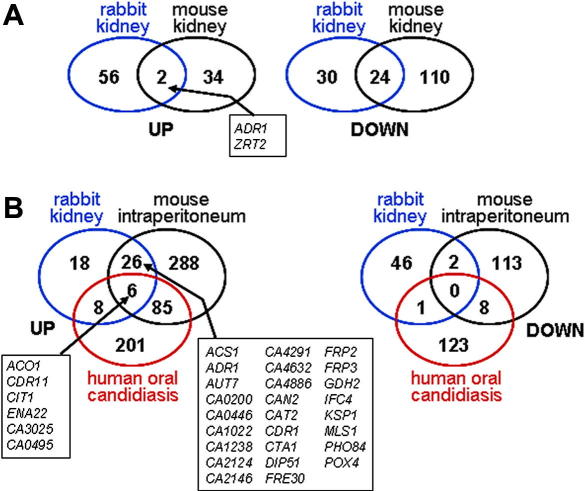
Comparison of the renal *C. albicans* SC5314 transcriptome with other *in vivo* microarray studies. The numbers of genes displaying >2-fold regulation in each study are illustrated in the Venn diagrams. (A) This rabbit renal study compared with the mouse kidney study of Andes and co-workers (2005). (B) This rabbit renal study compared with the mouse intraperitoneal study of Thewes et al. (2007) and the human oral candidiasis study of Zakikhany et al. (2007).

We compared our microarray data on rabbit renal infections with those from two other laboratories that have examined the *in vivo* transcriptome of *C. albicans*. Andes and co-workers (2005) examined the *C. albicans* transcriptome during mouse kidney infections, using YPD-grown cells as their comparator. They reported that 19% of all genes displayed >2-fold regulation in renal tissue compared with YPD-grown controls. They also observed up-regulation of glyoxylate cycle, lipid metabolism and stress genes, and the down regulation of genes involved in translation. However, there is limited overlap between their data and ours with respect to the *C. albicans* genes that were up- or down-regulated during renal infection (Fig. 2A). This is probably due in part to the different control conditions used in these studies: exponential RPMI 1640-grown cells in our case versus YPD-grown cells in the mouse renal study (Andes et al., 2005). Also, different microarray formats were used: Eurogentec microarrays were used in our case, whereas arrays from the Biotechnology Research Institute, National Research Council, Montreal were used by Andes and co-workers. Finally of course, different mammalian models were used: rabbits versus mice. These parameters might explain why only two *C. albicans* genes were up-regulated in both datasets: *ADR1* and *ZRT2*, both of which are putative zinc finger transcription factors. *ZRT2* is also transcriptionally induced during interactions with macrophages (Lorenz et al., 2004), but down-regulated *in vitro* in response to heat shock, osmotic stress, oxidative stress and amino acid starvation (Enjalbert et al., 2003; Tournu et al, 2005). Minimal regulation of *ADR1* has been reported in transcript profiling studies of *in vitro* culture conditions.

We also compared our results with data from Hube’s laboratory on mouse peritoneal infections and human oral infections (Thewes et al., 2007; Zakikhany et al., 2007). As expected there was greater overlap between the data from the kidney and peritoneal infections than between the datasets for either of the systemic infections (kidney or peritoneal) and the mucosal infections (Fig. 2B). Six *C. albicans* genes were up-regulated in the rabbit, mouse and human infections. Two of these encode functions involved in the utilization of alternative carbon sources (*ACO1*, *CIT1*) and one encodes a stress-related function (*ENA22*), once again reinforcing the view that stress and metabolic adaptation contribute to the fitness of this pathogen in its host. Twenty-six genes were up-regulated in both the rabbit and mouse infections (Fig. 2B). These included three genes involved in iron assimilation (*FRE30*), oxidative stress response (*CTA1*) and central carbon metabolism (*ACS1, MLS1*), reinforcing the view that these properties are important for virulence.

### 3.3 *In vivo* expression profiling of a congenic virulent strain

**Fig. 3:**
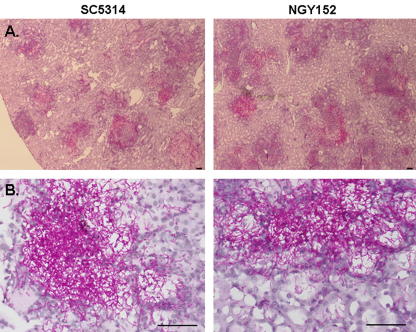
Histological analyses indicate that *C. albicans* SC5314 and NGY152 generate equivalent sizes of lesions and display similar cell morphologies in rabbit renal infections. Scale bars = 50 μm. (A) Low magnification. (B) Higher magnification.

The above data suggest that *C. albicans* genes associated with some virulence factors, fitness attributes and other functions are regulated during infection. We tested this further by examining a second *C. albicans* strain in the rabbit renal model. We chose the strain NGY152 because this strain is a virulent, prototrophic, congenic derivative of SC5314 (MacCallum and Odds, 2005). We confirmed the comparable virulence levels of these strains in the rabbit model by measuring fungal burdens in both kidneys of infected animals after 72 h of infection. For SC5314, the kidney burdens from one rabbit were 4.0 × 10^6^ and 4.6 × 10^6^ cfu/g, and for a second rabbit were 1.2 × 10^6^ and 1.5 × 10^6^ cfu/g. For NGY152, the kidney burdens in the first rabbit were 4.4 × 10^6^ and 3.7 × 10^6^ cfu/g, and in the second were 2.3 × 10^6^ and 3.8 × 10^6^ cfu/g. Animals infected with both strains displayed signs of clinical deterioration after three days. Furthermore histological analyses confirmed that kidney lesions generated by SC5314 and NGY152 were of similar size, and that SC5314 and NGY152 cells infecting the kidney displayed similar morphologies (Fig. 3). Therefore, the gross pathological effects of both strains were similar.

**Fig. 4:**
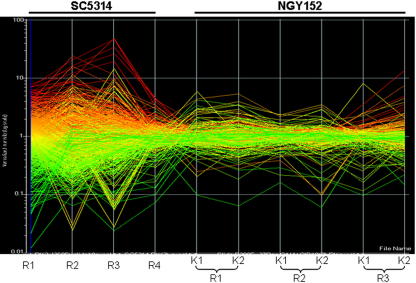
Comparison of the replicate microarray experiments for the *C. albicans* SC5314 and NGY152 renal infections. Each line represents a single gene, and each line is colour-coded on the basis of whether the corresponding gene was up- (red) or down-regulated (green) in the first SC5314 experiment: R1–R4, rabbits 1–4; K1–K2, kidneys 1–2. (For interpretation of colour mentioned in this figure the reader is referred to the web version of the article.)

Fig. 4 illustrates the consistency of the replicate *in vivo* expression profiles for *C. albicans* SC5314 and NGY152 and reveals significant differences between the transcriptomes of these closely related strains. Only a small number of *C. albicans* NGY152 genes were regulated reproducibly when cells from kidney lesions were compared to control cells grown in RPMI 1640 (Table 4). These differences were not caused by technical issues. NGY152 RNA isolated from cells infecting the kidney was of good quality (Supplementary data) and equivalent proportions of *C. albicans* genes gave significant signals on the SC5314 and NGY152 microarrays (Section 2.4).

To confirm the dramatic differences in the expression profiles of these closely related strains *in vivo* we performed qRT-PCR on the same set of transcripts that were used to validate the initial SC5314 microarray experiments: *DIP51*, *orf19.6079*, *FRP3*, *CTA1*, *FAA4* and *PHR1*. No significant regulation was observed for any of these transcripts in NGY152, in contrast to their strong regulation in SC5314 (Supplementary data). Therefore our qRT-PCR data validated our microarray experiments. Of the four genes that were up-regulated in NGY152 (*DDR48*, *GPM1*, *HSP12*, *PDC11*), none were in common with those genes that were up-regulated during SC5314 infections. However of the five that were down-regulated in NGY152 (*ADH1*, *ECE1*, *SOD5*; *IPF8762*, *PCK1*), the first three were also down-regulated during SC5314 infections. The functions of these genes that were up- or down-regulated in NGY152 further reinforce the view that morphogenesis, stress and metabolic adaptation contribute to disease progression. However, these data are also consistent with the idea that, while *C. albicans* gene regulation might occur during renal infections, much of this regulation is not essential for the infection process.

*Candida albicans* strain NGY152 is transcriptionally responsive to other conditions. For example, over 600 genes are regulated in response to *OCH1* inactivation (Carol Munro, personal communication). (*OCH1* encodes a mannosyltransferase involved in the glycosylation of cell wall mannoproteins: Bates et al., 2006). The *CRH11* and *SAP9* transcripts are down-regulated more than 5-fold, and the *PHO84* and *PGA29* mRNAs are up-regulated 5-fold following *OCH1* disruption in this strain background. Therefore a lack of responsiveness in the NGY152 transcriptome does not account for our observations in this study.

### 3.4 Comparison of *C. albicans* expression profiles from different kidneys

**Fig. 5:**
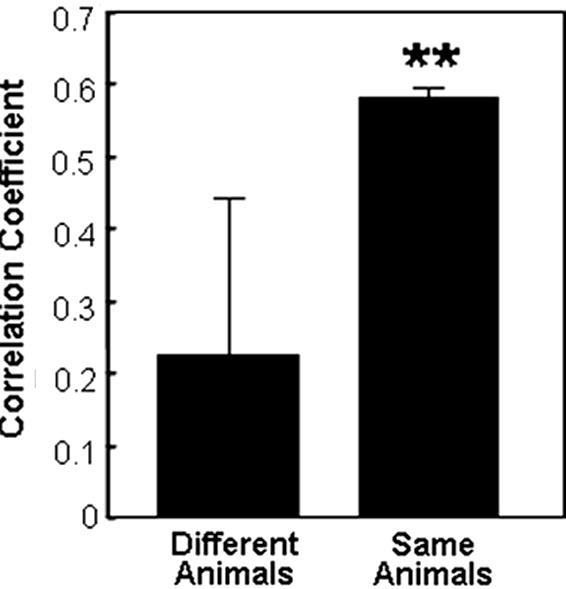
Comparison of genome-wide expression patterns for *C. albicans* NGY152 from different kidneys from the same animal versus kidneys from different animals. Mean correlation coefficients (±SD) for the pairwise comparisons of kidney infections using the whole microarray dataset for each kidney: **, significant at *p* < 0.01.

We compared the microarray data from individual kidneys infected with *C. albicans* NGY152. This was done by calculating pair-wise correlation coefficients for the global expression patterns for each kidney against all of the other kidneys. The mean correlation coefficient for the left and right kidneys from the same rabbit was significantly higher than for the mean correlation coefficient for kidneys from different rabbits (Fig. 5; Supplementary data). This indicates that the *C. albicans* expression profiles for cells infecting different kidneys in the same animal were more similar than the expression profiles from different animals (i.e. there is more biological variation between animals than between kidneys in the same animal). This is consistent with the idea that the behaviour of *C. albicans* is affected by the properties of the host and that variation between individual hosts can affect the expression profile of the pathogen. Our observation is also consistent with experimental variation in survival time that is generally observed for individual animals infected with equivalent inocula in mammalian models of disseminated candidiasis (MacCallum and Odds, 2005).

### 3.5 Comparison of *in vivo* phenome (virulence) with *in vivo* expression (profiling)

**Fig. 6:**
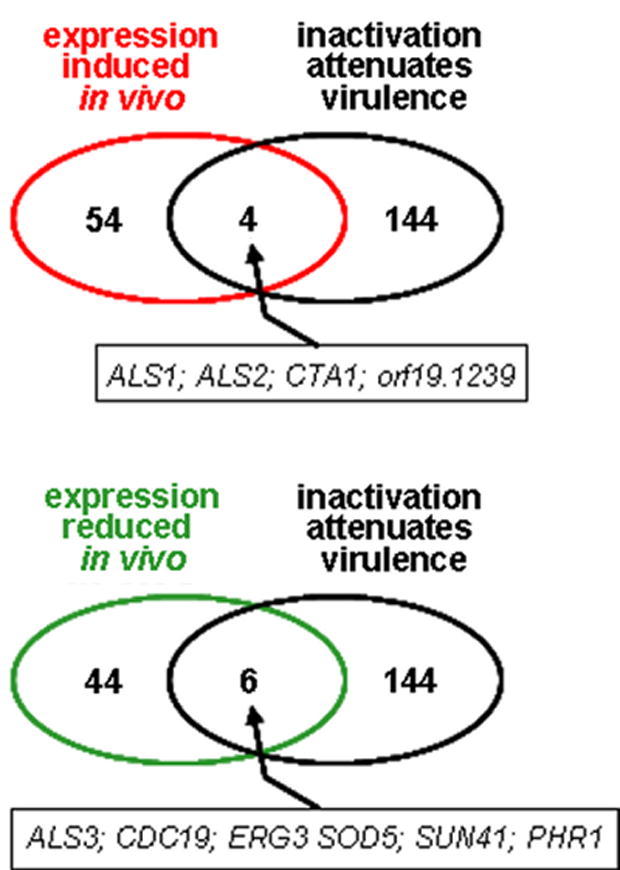
Comparison of the *in vivo* transcriptome (i.e. the subset of *C. albicans* SC5314 genes that were regulated during renal infections) with the *in vivo* phenome (i.e. the subset of *C. albicans* genes that affect the virulence of *C. albicans,* as defined by the *Candida* Genome Database @ August 2007) (Supplementary data).

Our data suggest that changes in expression occur during infection, but that many of these changes may not be essential for infection (Section 3.3). To test this we examined the overlap between the subset of *C. albicans* genes whose expression was induced *in vivo* (Table 2) and the subset of *C. albicans* genes that are essential for virulence (i.e. those genes that have been annotated as having an impact upon virulence by the *Candida* Genome Database: www.candidagenome.org) (Fig. 6; Supplementary data). Four of the 148 *C. albicans* genes that have been shown to contribute to virulence were up-regulated in the rabbit kidney lesions. These were *ALS1* and *ALS2* (both GPI-anchored cell surface adhesins: Hoyer et al., 1995, 2001), *CTA1* (which encodes catalase that contributes to oxidative stress protection: Wysong et al., 1998), and a gene of unknown function (*orf19.1239*). However, only a relatively small proportion of *C. albicans* genes have been virulence tested and the “phenome” of *C. albicans* is still very much incomplete. Indeed according to the *Candida* Genome database, only five of the *C. albicans* genes that were up-regulated in the rabbit kidney lesions have been virulence tested to date (Table 2). Four of these five genes are required for virulence.

We examined the relationship between the transcriptome and phenome further by looking at the genes that were down-regulated in the rabbit kidney (Fig. 6). We reasoned that, if there was a correlation between gene regulation and essentiality for infection, down-regulated genes would not display a virulence defect. However this was not the case. Seven of these down-regulated genes have been subjected to virulence testing (Table 3). Of these, six are required for virulence: *ALS3* (another GPI-anchored cell surface adhesin), *CDC19* (pyruvate kinase), *ERG3* (ergosterol biosynthesis), *SOD5* (a superoxide dismutase), *SUN41* (a cell wall glycosidase involved in biofilm formation) and *PHR1* (a pH-regulated cell surface glycosidase). Therefore in our experiments, there was a poor correlation between *in vivo* expression and virulence phenotype.

This poor correlation between the transcriptome and phenome is not surprising when *Saccharomyces cerevisiae* genomic datasets are considered. Genome-wide comparisons between the regulation of genes and their contribution to fitness under equivalent growth conditions revealed a poor correlation between the transcriptome and the “phenome” (Giaever et al., 2002). Several factors probably account for this. For example, the inactivation of individual genes that encode redundant functions would not be expected to impair fitness even if the function itself was essential. Also, the activities of many signal transduction proteins are regulated by post-translational modification, rather than at the transcriptional level. These phenomena may account, at least in part, for the lack of correlation between the *in vivo C. albicans* transcriptome and the subset of genes that significantly affect the virulence of this pathogen. Also, some genes that are expressed during infection and that contribute to virulence may not display significant changes in expression when compared to our control condition (growth in RPMI 1640). Moreover, the expression profile for the fungal cells in a particular lesion reflects the average expression pattern for these cells, rather than the contributions of individual cells within that lesion. Since heterogeneity in gene expression has been observed microscopically for intra-lesional fungal cells (Barelle et al., 2006, 2008; Enjalbert et al., 2007), functionally significant changes in gene expression that might occur in subsets of cells within a lesion may not be detected when the fungal cells are examined *en masse* by transcript profiling.

## 4 Conclusions

Several significant conclusions can be drawn from this study. Using new procedures for the analysis of the *C. albicans* transcriptome *in vivo*, which circumvent the need for PCR-based amplification, we have characterized the *C. albicans* transcriptome within rabbit renal lesions. The *C. albicans* genes that were found to be regulated during these infections did not show considerable overlap with those reported previously for mouse kidney infections or human oral infections (Andes et al., 2005; Zakikhany et al., 2007). Greater overlap was observed with datasets for mouse intraperitoneal infections (Thewes et al., 2007). Taken together, the data reinforce the view that the differential regulation of adhesins and morphogenesis, along with metabolic and stress adaptation, are associated with the development of systemic *C. albicans* infections.

Significantly, our comparison of the *in vivo* transcriptomes of two closely related *C. albicans* strains revealed minimal overlap. This suggested a poor correlation between the *C. albicans* transcriptome and phenome during renal infections. This view was reinforced by a comparison of *C. albicans* genes that were regulated during SC5315 kidney infections and those genes that have been reported to influence the virulence of this pathogen. This lack of correlation between the transcriptome and this phenome is consistent with genomic studies in the relatively benign model yeast, *S. cerevisiae* (Giaever et al., 2002). More comprehensive analyses of the *C. albicans* phenome, and more refined analyses of *C. albicans* virulence, for example using competition assays or specialized infection models, might reveal more subtle effects on virulence that relate to observed changes in gene expression *in vivo*.

## Figures and Tables

**Table 1:** Impact of fixation and enrichment procedures upon the *C. albicans* transcriptome. Genes displaying 2-fold regulation or more are listed.

**Table 2:** Up-regulated genes in *C. albicans*SC5314 kidney lesions.

**+**, virulence defect; **−**, no virulence defect; n, virulence not tested (according to CGD).

**Table 3:** Down-regulated genes in *C. albicans* SC5314 kidney lesions.

**+**, virulence defect; **−**, no virulence defect; n, virulence not tested (according to CGD).

**Table 4:** Regulated genes in *C. albicans* NGY152 kidney lesions.

**+**, virulence defect; n, virulence not tested (according to CGD). ^a^No genes in common with subset of up-regulated in *C. albicans* SC5314 cells. ^b^Also down-regulated in *C. albicans* SC5314 cells.
