# Supplementary material for: Genome-wide analysis of Candida albicans gene expression patterns during infection of the mammalian kidney
Source: Fungal Genet Biol. 2009 Feb;46(2):210–9. doi: 10.1016/j.fgb.2008.10.012 (PMC2698078; doi:10.1016/j.fgb.2008.10.012)
Supplement: Supplementary Data 1 [file mmc1.pdf]

Supplementary Data

	Sheet Title	Contents
	**Sheet Title**	**Contents**
1	Summary	Summary of data sheets in Supplementary Data
2	Control Complete Dataset	Control microarrays: complete dataset
3	Control Regulated Genes	Control microarrays: up- and down-regulated genes
4	SC5314 Complete Dataset	SC5314 microarrays: complete dataset
5	SC5314 - Up genes	SC5314 microarrays: up-regulated genes
6	SC5314 - Down genes	SC5314 microarrays: down-regulated genes
7	NGY152 Complete Dataset	NGY152 microarrays: complete dataset
8	NGY152 - Up genes	NGY152 microarrays: up-regulated genes
9	NGY152 - Down genes	NGY152 microarrays: down-regulated genes
10	Kidney Comparisons	Comparison of NGY152 expression profiles in different kidneys
11	Expression v Phenotype	Gene expression versus virulence phenotype
12	Primers	PCR primers used in this study
13	*In vivo* versus YPD	Microarray data comparing *C. albicans* grown *in vivo* or in YPD (relative to RMPI)
14	RNA integrity	Integrity of RNA preps from fungal cells infecting kidney
15	SC5314 versus NGY152	qRT-PCR of transcript levels in SC5314 and NGY152
