# Supplementary material for: Genome-wide analysis of Candida albicans gene expression patterns during infection of the mammalian kidney
Source: Fungal Genet Biol. 2009 Feb;46(2):210–9. doi: 10.1016/j.fgb.2008.10.012 (PMC2698078; doi:10.1016/j.fgb.2008.10.012)
Supplement: Supplementary Data 10 [file mmc10.pdf]

Comparison of NGY152 expression profiles in different kidneys

Same Animals

Kidneys	Correlation Coefficient
1A X 1B	0.51
2A X 2B	0.67
3A X 3B	0.55
**Mean**	**0.58**
**SD**	**0.08**

Different Animals

Kidneys	Correlation Coefficient
1B X 2A	0.04
1A X 2A	0.05
1A X 2B	0.06
1A X 3A	0.08
1A X 3B	0.11
1B X 2A	0.62
1B X 2B	0.07
1B X 3A	0.04
1B X 3B	0.11
2A X 3A	0.48
2A X 3B	0.52
2B X 3A	0.10
2B X 3B	0.64
**Mean**	**0.23**
**SD**	**0.24**
