# Supplementary material for: Genome-wide analysis of Candida albicans gene expression patterns during infection of the mammalian kidney
Source: Fungal Genet Biol. 2009 Feb;46(2):210–9. doi: 10.1016/j.fgb.2008.10.012 (PMC2698078; doi:10.1016/j.fgb.2008.10.012)
Supplement: Supplementary Data 11 [file mmc11.pdf]

Gene expression versus virulence phenotype

** Virulence phenotype as annotated in CGD (August 2007)

Gene	Gene product	Fold expression *in vivo*				Virulence **	First author	Journal	Volume page no.	Date
AAF1	unknown function	0.85	0.88		1.10	attenuated	Rieg	Infection and Immunity	67(7), 3193	1999
ACE2	cell separation factor	1.14	1.09	1.03	1.06	attenuated	Kelly	Molecular Microbiology	53(3), 969	2004
ADE2	phosphoribosylamino-	1.03	1.12	0.70	1.19	attenuated	Kirsch	Infection and Immunity	59(9), 3297	1991
ALO1	D-arabinono-1,4-lactone	1.06	0.53	1.01	0.93	attenuated	Huh	Infection and Immunity	69(6), 3939	2001
ALS1	surface adhesin	4.41	4.65	3.13	1.28	attenuated	Fu	Molecular Microbiology	44(1), 61	2002
AQY1	aquaporin	1.06	1.63	1.21	1.08	normal	Carbrey	Yeast	18(15), 1391	2001
ARN1 / SIT1	Iron transporter	2.34	0.75	1.27	1.26	normal	Chuan-Jiong Hu			2002
ASH1	transcription factor	1.35	0.91	0.99	1.20	attenuated	Inglis	Molecular and Celular Biology	22(24), 8669	2002
BGL2	1-3 b-glucosyltransferase	0.95	1.08	0.82	1.15	attenuated	Sarthy	Microbiology	143(Pt 2), 367	1997
BIG1	b-1,6-glucan biosynthesis	0.99	0.90	1.22		atteunated	Umeyama	Infection and Immunity	74(4), 2373	2006
BNI1	formin	0.62	1.25	0.94	1.23	attenuated	Li	Eukaryotic Cell	118(Pt. 12), 2637	2005
CAP1	adenyl cyclase regulator	1.47	1.72	1.51	1.32	attenuated	Bahn	Journal of Bacteriology	183(10), 3211	2001
CDC10	septin	0.50	0.59	0.91	0.91	attenuated	Warenda	Infection and Immunity	71(7), 4045	2003
CDC11	septin	0.90	0.81	0.78	0.83	attenuated	Warenda	Infection and Immunity	71(7), 4045	2003
CDC24	GDP:GTP exchange factor	1.68	1.31	1.25	0.88	attenuated	Bassilana	Eukaryotic Cell	2(1), 9	2003
CDC35 / CYR1	adenyl cyclase regulator	2.02	0.93	0.97	1.09	attenuated	Rocha	Molecular Biology of the Cell	12(11), 3631	2001
CDC42	G-protein (GTPase)	0.70	0.72	0.91	1.18	attenuated	Bassilana	Eukaryotic Cell	2(1), 9	2003
CEK1 / ERK1	MAP kinase	1.01	0.94	0.94	0.92	attenuated	Csank	Infection and Immunity	66(6), 2713	1998
CHS1	chitin synthase	0.81	0.88	1.06	0.80	attenuated	Munro	Molecular Microbiology	39(5), 1414	2001
CHS2	chitin synthase	1.30	0.89	0.90	1.25	normal	Gow	PNAS	91(13), 6216	1994
CHS3	chitin synthase	1.28	1.05	1.03	1.02	attenuated	Bulawa	PNAS	92(23), 10570	1995
CKA2	protein kinase	0.84	0.86	1.04	1.00	attenuated in model of oropharyngeal candidiasis	Chiang	Cellular Microbiology		2006
CLA4	ser/thr protein kinase	1.20	0.93	0.91	1.16	attenuated	Leberer	Current Biology	7(8), 539	1997
CNA1 / CMP1	calcineurin A	1.07	1.01	1.16	1.00	attenuated	Sanglard	Molecular Microbiology	48(4), 959	2003
CNB1	calcineurin B	0.58	0.85	0.92	1.00	attenuated	Blankenship	Eukaryotic Cell	2(3), 422	2003
COS1 / NIK1	histidine kinase	1.45	1.61	1.12	1.26	attenuated	Selitrennikoff	Medical Mycology	39(1), 69	2001
CPH1	regulatory cascade	1.05	1.04	1.02	1.22	normal	Csank	Infection and Immunity	66(6), 2713	1998
CPP1	protein phosphatase	1.22	0.98	1.00	0.93	attenuated	Csank	Molecular Biology of the Cell	8(12), 2539	1997
CRH11	cell wall protein	0.22	0.16	0.57	0.91	attenuated	Pardini	Journal of Biological Chemistry		2006
CRH12	cell wall protein	0.88	0.87	1.03	1.19	attenuated	Pardini	Journal of Biological Chemistry		2006
CRK1	Cdc2 kinase	1.79	1.22	1.07	0.85	attenuated	Chen	Molecular and Celular Biology	20(23), 8696	2000
CRZ1	calcineurin dependent zinc finger	1.05	1.13	1.28	0.89	normal	Onyewu	Infection and Immunity	72(12), 7330	2004
CSF4 / UTR2	glycosidase	1.01	1.09	0.92	1.00	attenuated	Alberti-Segui	Yeast	21(4), 285	2004
CSH1 / IFO4	cell surface hydrophobicity	0.54	0.93	0.97	0.88	attenuated	Singleton	FEMS Microbiology Letters	244(2), 373	2005
CSH3 / SHR3	ER packaging chaperone	1.52	0.94	0.90	0.85	attenuated	Martínez P	Molecular Microbiology	51(2), 371	2004
CSP37	37 kDa surface protein	1.72	1.91	1.10	0.94	attenuated	Sentandreu	Journal of Bacteriology	179(15), 4654	1997
CST20 / STE20	MEKK kinase	1.06	1.09	1.04	1.17	attenuated	Leberer	PNAS	93(23), 13217	1996
CTA1	catalase	9.63	3.90	5.31	1.23	attenuated	Wysong	Infection and Immunity	66(5), 1953	1998
DAC1 / NAG2	N-acetyl GlcNH2 catabm	0.80	1.30	0.97	1.09	attenuated	Singh	Infection and Immunity	69(12), 7898	2001
ECM33	surface protein	0.53	0.43	0.24	1.05	attenuated	Martinez-Lopez	Microbiology	150(Pt 10), 3341	2004
EFG1	transcription factor	1.90	1.82	1.55	1.06	attenuated	Lo	Cell	90(5), 939	1997
ERG3	ergosterol synthesis	0.43	0.20	0.46	0.85	attenuated	Chau	Antimicrobial Agents and Chemotherapy	49(9), 3636	2005
ESS1 / PIN1	peptidyl-prolyl cis/trans isomeras	0.80	0.86	0.79	1.03	attenuated	Li	Biochemistry	44(16), 6180	2005
FAD2	fatty acid synthase	1.49	1.03	1.17	0.88	normal	Murayama	Microbiology	152(Pt 5), 1551	2006
FAD3	fatty acid synthase	1.10	0.99	0.90	1.01	normal	Murayama	Microbiology	152(Pt 5), 1551	2006
FAS2	fatty acid synthase	0.78	0.53	1.08	0.83	attenuated	Zhao	Infection and Immunity	65(2), 829	1997

FBA1	fructose-1,6-biphosphate aldolas	0.50	1.76	0.80	0.72	partial attenuation	Rodaki	Eukaryotic Cell	5(8), 1371	2006
FET3	iron transport protein	1.11	1.04	1.02	1.01	normal	Eck	Microbiology	145(Pt9), 2415	1999
FLO8	transcription factor	4.46	1.05	1.37	0.96	avirulent	Cao	Molecular Biology of the Cell	17(1), 295	2006
FOX2	b-oxidation					attenuated	Piekarska	Eukaryotic Cell	5(11), 1847	2006
FTR1	high affinity iron permease	0.58	0.30	0.19	0.80	attenuated	Ramanan	Science	288(5468), 1062	2000
FTR2	high affinity iron permease	1.05	0.21	0.50	1.06	normal	Ramanan	Science	288(5468), 1062	2000
GAT1	transcription factor	1.12	0.86	0.99	1.02	attenuated	Limjindaporn	Molecular Microbiology	50(3), 993	2003
GNA1	gln-6-P acetyl transferase	0.74	1.02	1.84	1.05	attenuated	Mio	Microbiology	146(Pt 7), 1753	2000
GPI7	transcription factor	1.49	1.05	1.09	0.85	attenuated	Richard	Molecular Microbiology	44(3), 841	2002
HEM3	uroporphyrin I synthase	2.27	1.13	1.26	1.16	attenuated	Kirsch	Infection and Immunity	59(9), 3297	1991
HGT4 / SNF3 / GSP1	glucose sensor	3.26	1.71	2.30	1.12	slower than normal	Brown	Eukaryotic Cell	5(10), 1726	2006
HK1	histidine kinase	1.44	1.08	1.09	0.94	attenuated	Calera	Infection and Immunity	68(2), 518	1999
HOG1	MAP kinase	0.73	0.92	1.05	1.03	attenuated	Alonso-Monge	Journal of Bacteriology	181(10), 3058	1999
HSL1	protein kinase	0.65	0.91	1.00	1.07	attenuated	Umeyama	Molecular Microbiology	55(2), 381	2005
HST1	regulatory cascade					normal	Csank	Infection and Immunity	66(6), 2713	1998
HST7 / STE7	MEK kinase	1.05	0.88	1.03	0.93	normal	Leberer	PNAS	93(23), 13217	1996
HWP1	transglutaminase	0.38	0.96	0.03	0.95	attenuated	Staab	Science	283(5407), 1535	1999
ICL1	isocitrate lyase	1.96	4.54	1.42	1.26	attenuated	Lorenz	Nature	412(6842), 83	2001
INT1	surface protein	0.66	0.89	0.88	1.30	attenuated	Gale	Science	279:1355–1358	1998
IRO1	possible transcription factor	0.55	1.17	0.92	1.04	attenuated	Chibana	Microbiology and Immunology	49(10), 937	2005
IRS4	immunogenic protein	1.38	0.99	1.10	1.02	partial attenuation	Badrane	Microbiology	151(pt 9), 2923	2005
KEX2	kexin (subtilase)	0.92	1.18	0.90	1.15	attenuated	Newport	The Journal of Biological Chemistry	278(3), 1713	2003
KRE5	UDP-glucose:glycoprotein glucosyltransferase	1.27	0.97	1.12	0.93	attenuated	Herrero	Eukaryotic Cell	3(6), 1423	2004
LIG4 / CDC9	DNA ligase	0.87	1.16	0.69	1.22	attenuated	Andaluz	Infection and Immunity	69(1), 137	2001
MAD2	spindle assembly factor	0.83	1.04	1.08	0.82	attenuated	Bai	Molecular Microbiology	45(1), 31	2002
MDR1	membrane efflux pump	1.77	1.16	1.03	0.78	attenuated	Becker	Infection and Immunity	63(11), 4515	1995
MIG1	DNA-binding protein	1.01	0.86	1.05	1.12	normal	Zaragoza	Journal of Bacteriology	182(2), 320	2000
MIT1	inositol P-ceramide/	1.20	1.31	1.24	1.10	attenuated	Mille	The Journal of Biological Chemistry	279(46), 47952	2004
MKC1	MAP kinase	0.74	1.17	0.86	0.97	attenuated	Diez-Orejas	Infection and Immunity	65(2), 833	1997
MNN5	iron regulated a1,2-mannosyl transferase	1.22	0.87		1.24	attenuated	Bai	Eukaryotic Cell	5(2), 238	2006
MNT1	a1,2-mannosyl transferase	0.97	0.69	0.76	1.15	attenuated	Buurman	PNAS	95(13), 7670	1998
MNT2	mannosyl transferases	0.78	1.11		0.94	attenuated	Munro	The Journal of Biological Chemistry	280(2), 1051	2005
NAG2	NAGP deacetylase	0.80	1.30	0.97	1.09	attenuated	Yamada-Okabe	FEMS Microbiology Letters	212(1), 15	2002
NAG5 / HXK1	NAG kinase	1.20	1.24	0.97	0.94	attenuated	Yamada-Okabe	FEMS Microbiology Letters	212(1), 15	2002
NIK1 / COS1	histidine kinase	1.45	1.61	1.12	1.26	attenuated	Yamada-Okabe	Journal of Bacteriology	181(23), 7243	1999
NMT1	N-myristoyl transferase	1.04	1.06	1.01	1.13	attenuated	Weinberg	Molecular Microbiology	16(2), 241	1995
NOT4 / MOT2 / SIG1	transcription factor	1.00	1.00	1.08	0.93	attenuated	Krueger	Microbiology	150(Pt 1), 229	2004
NOT5	immunogenic protein	0.90	1.26	1.07	0.96	attenuated only in intravenous infection of neutropenic and immunosuppressed mice	Cheng	Infection and Immunity	73(11), 7190	2005
NRG1	transcriptional repressor	2.43	1.49		0.92	attenuated	Murad	The EMBO Journal	20(17), 4742	2001
NTC1 / NTH1	neutral trehalase	1.10	1.26	1.29	1.38	normal	Eck	Microbiology	143(Pt 12), 3747	1997
PEX5	b-oxidation	1.43	1.92	1.10	0.93	normal	Piekarska	Eukaryotic Cell	5(11), 1847	2006
PHR1	pH-regulated expression	0.36	0.26	0.23	0.42	attenuated	Ghannoum	Infection and Immunity	63(11), 4528	1995
PHR2	pH-regulated expression	0.81	0.92	1.06	1.05	normal	De Bernardis	Infection and Immunity	66(7), 3317	1998
PLB1	phospholipase B	0.88	1.01		1.00	attenuated	Leidich	The Journal of Biological Chemistry	273(40), 26078	1998
PLC2	phospholipase C					normal	Kunze	Microbiology	151(Pt 10), 3381	2005
PLC3	phospholipase C	1.54	0.97		0.80	normal	Kunze	Microbiology	151(Pt 10), 3381	2005
PLD1 / SPO14	phospholipase D	1.49	1.56	0.85	0.95	attenuated	Hube	Microbiology	147(Pt 4), 879	2001
PMR1	P-type ATPase	0.80	0.95	0.93	0.91	attenuated	Bates	The Journal of Biological Chemistry	280(24), 23408	2005

PMT1	protein-O-mannosylation	1.08	0.63		0.96	attenuated	Timpel	The Journal of Biological Chemistry	273(33), 20837	1998
PMT2	protein-O-mannosylation	0.50	0.64	0.64	0.94	attenuated	Rouabhia	Infection and Immunity	73(8), 4571	2005
PMT4	protein-O-mannosylation	0.41	0.85	0.67	1.18	attenuated	Rouabhia	Infection and Immunity	73(8), 4571	2005
PMT5	protein-O-mannosylation	0.76	1.01	0.97	1.08	attenuated	Rouabhia	Infection and Immunity	73(8), 4571	2005
PMT6	protein-O-mannosylation	0.66	0.81	1.11	1.01	attenuated	Rouabhia	Infection and Immunity	73(8), 4571	2005
RAD52	homologous recombination	0.93	0.85	0.92	0.92	1x10(6) and 1.5x10(6) cells	Chauhan	Infection and Immunity	73(12), 8069	2005
RAS1	regulates gene expression	1.16	1.50	1.23	1.09	attenuated	Leberer	Molecular Microbiology	42(3), 673	2001
RBT1	hypha-specific wall protein	0.31	0.56	1.02	1.31	attenuated	Braun	Genetics	156(1), 31	2000
RBT4	unknown function	0.30	0.59	0.22	0.90	attenuated	Braun	Genetics	156(1), 31	2000
RBT5	unknown function	0.43	0.56	0.32	0.30	attenuated	Braun	Genetics	156(1), 31	2000
RFG1 / ROX1	transcription factor	0.89	0.83	0.99	0.83	attenuated	Kadosh	Molecular and Celular Biology	21(7), 2496	2001
RIM101	transcription factor	1.51	0.94	2.86	0.83	attenuated	Davis	Infection and Immunity	68(10), 5953	2000
RIM8	transcription factor	1.16	1.27	0.97	0.90	attenuated	Davis	Infection and Immunity	68(10), 5953	2000
RSR1	GTPase	0.72	0.99	1.20	1.00	attenuated	Yaar	Microbiology	143(Pt 9), 3033	1997
SAP1	aspartyl proteinase	0.86	0.91	1.09	1.14	attenuated	Hube	Infection and Immunity	65(9), 3529	1997
SAP2	aspartyl proteinase	1.07	0.78	0.62	1.11	attenuated	Hube	Infection and Immunity	65(9), 3529	1997
SAP3	aspartyl proteinase	0.98	0.87	0.88	0.93	attenuated	Hube	Infection and Immunity	65(9), 3529	1997
SAP4	SAP4-6 KO	0.94	0.85	1.15	1.00	attenuated	Sanglard	Infection and Immunity	65(9), 3539	1997
SAP5	SAP4-6 KO	0.95	0.89	1.24	0.88	attenuated	Sanglard	Infection and Immunity	65(9), 3539	1997
SAP6	SAP4-6 KO	0.96	0.92	1.06	1.01	attenuated	Sanglard	Infection and Immunity	65(9), 3539	1997
SET1	methyltransferase	0.92	1.14	1.22	1.09	partial attenuation	Raman	Molecular Microbiology	60(3), 697	2006
SIT4	protein phosphatase	1.15	1.01	1.15	1.14	attenuated	Lee	Molecular Microbiology	51(3), 691	2004
SLN1	histidine kinase	1.48	1.21	0.92	0.72	attenuated	Yamada-Okabe	Journal of Bacteriology	181(23), 7243	1999
SNF1	essential for viability	1.12	1.04	0.99	0.87	normal	Petter	Infection and Immunity	65(12), 4909	1997
SOD1	superoxide dismutase	0.85	0.74	0.82	0.95	attenuated	Hwang	Microbiology	148(Pt 11), 3705	2002
SOD5 / PGA3	superoxide dismutase	0.11	0.12	0.10	0.68	attenuated	Martchenko	Molecular Biology of the Cell	15(2), 456	2004
SPA2	cell polarity regulator	1.07	1.19	1.15	1.11	attenuated	Zheng	Molecular Microbiology	49(5), 1391	2003
SPT3	transcriptional activator	1.32	1.05	0.87	0.76	attenuated	Laprade	Genetics	161(2), 509	2002
SSK1	2-component response reg.	1.16	1.35	1.14	1.25	attenuated	Calera	Infection and Immunity	67(8), 4280	2000
SSN6	transcriptional corepressor	1.22	0.85	0.97	1.41	attenuated	Hwang	Microbiology	47(4), 1029	2003
SWI1	chromatin remodelling	0.89	0.92		1.01	attenuated	Mao	FEBS Letters	580 (11), 2615	2006
TEC1	hyphal transcription factor	0.95	1.42	0.95	1.36	attenuated	Schweizer	Molecular Microbiology	38 (3), 435	2000
TOP1	topoisomerase	0.75	1.04	1.07	0.92	attenuated	Jiang	Microbiology	143 (Pt 2), 377	1997
TPK1	protein kinase A subunit	1.14	0.96	1.00	1.07	normal	Park			2005
TPK2	protein kinase A subunit	0.71	0.84	0.88	1.03	attenuated	Sonneborn	Molecular Microbiology	35 (2) 386	2000
TPS1	trehalose phos. synthase	0.76	0.96	0.96	1.07	attenuated	Zaragoza	Journal of Bacteriology	182(2), 320	1998
TPS2	trehaloseP phosphatase	1.01	0.93	0.88	0.66	attenuated	Van Dijck	Infection and Immunity	70 (4) 1772	2002
TUP1	transcriptional repressor	1.68	0.68	1.11	0.95	attenuated	Murad	The EMBO Journal	20(17), 4742	2001
URA3	nucleotide synthesis	1.51	0.96	0.95	0.83	attenuated	Kirsch	Infection and Immunity	59(9), 3297	1991
UTR2	cell wall protein	1.02	1.11	0.93	1.01	attenuated	Pardini	Journal of Biological Chemistry	281 (52), 40399	2006
VAC1 / PEP7	vesicle transport	1.44	1.03	1.05	0.85	attenuated	Franke	Microbiology	152 (Pt 10), 3111	2006
VMA7	vacuolar ATPase	0.81	1.21	1.05	0.94	attenuated	Poltermann	Microbiology	151 (Pt 5) 1645	2005
VPS28	endocytic coponents	0.71	1.13	1.03	0.95	attenuated	Cornet	Infection and Immunity	73 (12), 7977	2005
VPS32 / SNF7	endocytic components	1.25	0.97		1.17	attenuated	Cornet	Infection and Immunity	73 (12), 7977	2005
VPS34	P-inositol-3-kinase	0.91	1.21		1.17	attenuated	Bruckmann	Microbiology	146 (Pt 11), 2755	2000
XOG1 / EXG1	exoglucanase	0.56	1.03	0.91	1.20	normal	Gonzalez	Microbiology	143 (Pt 9), 3023	1997
YHB1	flavohemoglobin	1.41	1.67		1.11	attenuated	Hromatka	Molecular Biology of the Cell	16, 10, 4814	2005
YVH1	protein phosphatase	0.89	1.00	0.77	1.03	attenuated	Hanaoka	Microbiology	151 (Pt 7), 2223	2005
YWP1 / PGA24 / FLO1 / TEP1	putative cell wall protein	1.77	1.11	1.29	1.12	normal	Granger	Microbiology	151 (Pt 5), 1631	2005
