# Supplementary material for: Genome-wide analysis of Candida albicans gene expression patterns during infection of the mammalian kidney
Source: Fungal Genet Biol. 2009 Feb;46(2):210–9. doi: 10.1016/j.fgb.2008.10.012 (PMC2698078; doi:10.1016/j.fgb.2008.10.012)
Supplement: Supplementary Data 12 [file mmc12.pdf]

PCR primers used in this study

Primer	Sequence (5’ to 3’)
EFB1-5’	gctgctaaaggtccaaaacc
EFB1-3’	catcccatggtttgacatcc
DIP51.5F-5’	cagctcataatgcagaagaagg
DIP51.5F-3’	agaagacctgttcccaatgc
FAA4-5’	gaccgaaacttgtgctaatgc
FAA4-3’	ttgatcccaccaaagtaccc
CTA1-5’	tttggccacacaagaaatacc
CTA1-3’	agcagcttgttcaacttcagc
FRP3-5’	gtttgaattggccgttgg
FRP3-3’	acttgaatagcagcccaagc
IPF14618-5’	aggacccaactcttttgagg
IPF14618-3’	cagcagcttcagtgacaagg
