# Supplementary material for: Genome-wide analysis of Candida albicans gene expression patterns during infection of the mammalian kidney
Source: Fungal Genet Biol. 2009 Feb;46(2):210–9. doi: 10.1016/j.fgb.2008.10.012 (PMC2698078; doi:10.1016/j.fgb.2008.10.012)

Microarray data comparing growth in RPMI and YPD in vitro

Genome-wide comparisons of *in vivo*- grown and YPD-grown *C. albicans* cells.
Functional categories containing up-regulated genes in each comparison are shown:

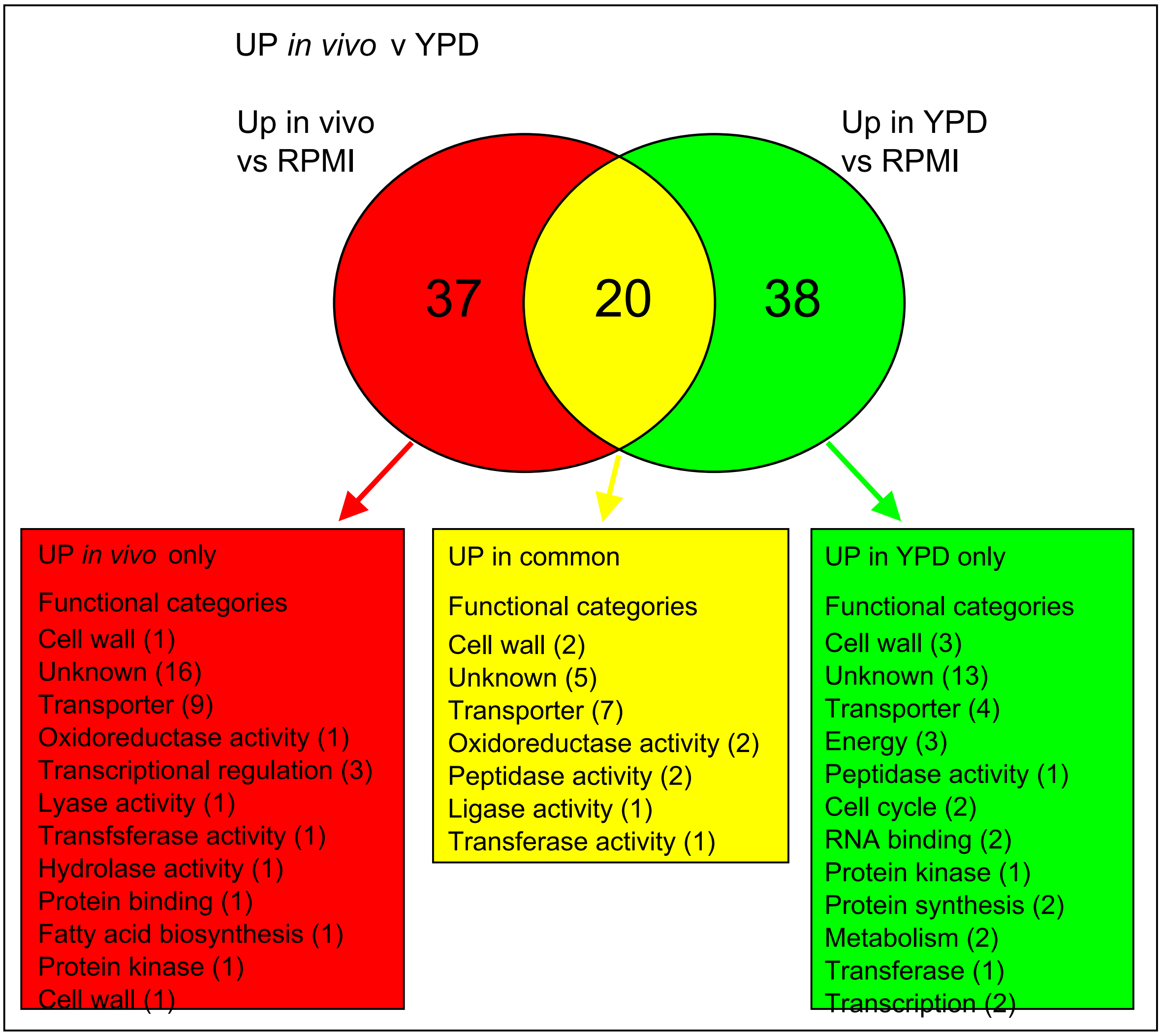

Supplement: Supplementary Data 13 [file mmc13.pdf]
