# Supplementary material for: Genome-wide analysis of Candida albicans gene expression patterns during infection of the mammalian kidney
Source: Fungal Genet Biol. 2009 Feb;46(2):210–9. doi: 10.1016/j.fgb.2008.10.012 (PMC2698078; doi:10.1016/j.fgb.2008.10.012)

Integrity of RNA preps from fungal cells infecting kidney

The integrity of RNA preparations from fungal cells isolated from rabbit kidneys was confirmed by agarose gel electrophoresis:

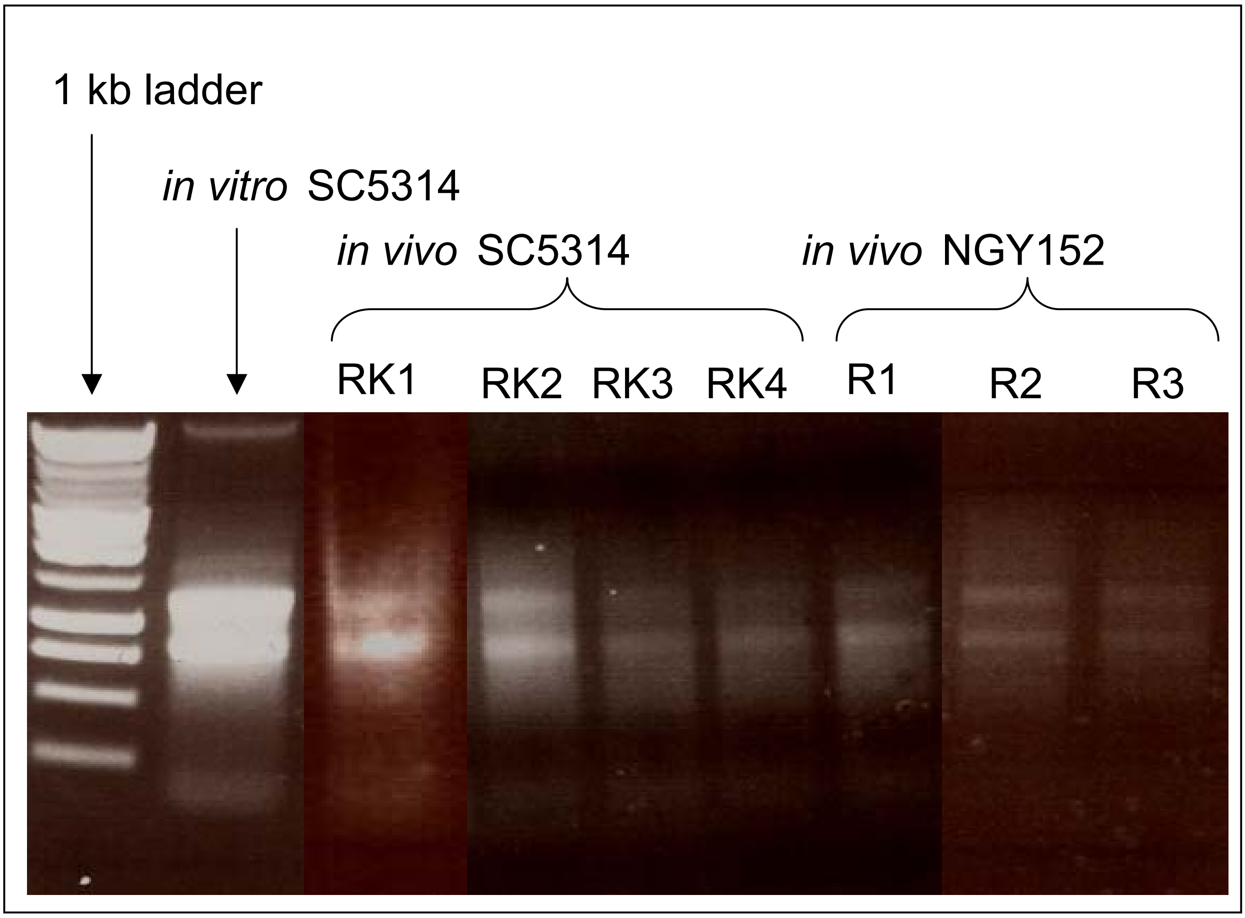

Supplement: Supplementary Data 14 [file mmc14.pdf]
