# Supplementary figures and images for: Genome-wide analysis of Candida albicans gene expression patterns during infection of the mammalian kidney
Source: Fungal Genet Biol. 2009 Feb;46(2):210–9. doi: 10.1016/j.fgb.2008.10.012 (PMC2698078; doi:10.1016/j.fgb.2008.10.012)

qRT-PCR of transcript levels in SC5314 and NGY152

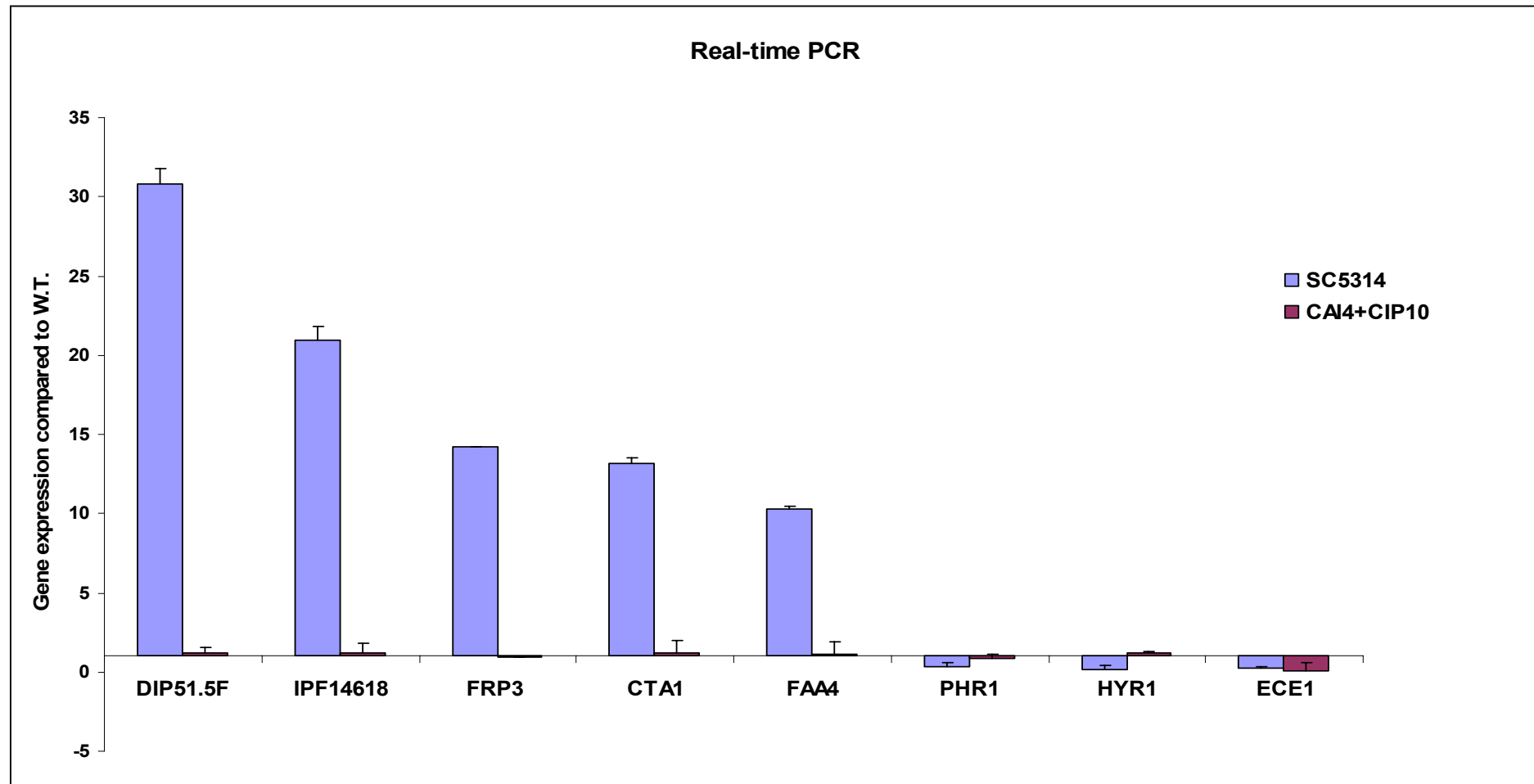

Supplement: Supplementary Data 15 [file mmc15.pdf]
