# Supplementary material for: Genome-wide analysis of Candida albicans gene expression patterns during infection of the mammalian kidney
Source: Fungal Genet Biol. 2009 Feb;46(2):210–9. doi: 10.1016/j.fgb.2008.10.012 (PMC2698078; doi:10.1016/j.fgb.2008.10.012)
Supplement: Supplementary Data 2 [file mmc2.pdf]

All Genes

Regulation of SC5314 genes in RPMI 1640 plus fixation and centrifugation versus SC5314 genes in RPMI 1640

Replicate	1	2	3									
File Name	RPMI30 min	RPMI30 min	F200E_29									
Systematic	Normalized	Normalized	Normalized	Common	Map	Description	Function	orf19.#	IPF#			
CA0001	1.0	1.0	0.9	IPF19501	complemer	unknown function			19501	Name	MIPS Cate	GO molecular function
CA0002	1.1	1.0	1.0	DOC1	23221..246	component of the anap		orf19.1252	12212	IPF19501	No significant S.c. match	
CA0003	0.9	1.0	0.9	IPF19484	31023..322	putative zinc amino peptidase (by			19484	CaDOC1	CELL CYC	enzyme regulator activity
CA0005	1.0	1.1	1.0	IPF14994	64471..654	unknown function		orf19.2414	14994	IPF19484	PROTEIN FATE [folding modification destination]	
CA0006	0.9	0.9	1.0	IPF2072	78709..797	unknown function		orf19.1156	2072	IPF14994	UNCLASSI	molecular_function unknown
CA0007	1.0	1.1	1.0	IPF12061	complemer	unknown function		orf19.7807	12061	IPF2072	UNCLASSI	molecular_function unknown
CA0008	1.0	1.0	0.9	IPF12110	complemer	1,4-butanediol diacryla		orf19.376	12110	IPF12061	No significant S.c. match	
CA0009	1.0	0.9	0.9	IPF11382	complemer	unknown function		orf19.8247	11382	IPF12110	No significant S.c. match	
CA0010	1.0	1.1	1.0	IPF16036	130706..13	similar to Saccharomy		orf19.7790	16036	IPF11382	No significant S.c. match	
CA0011	1.1	1.0	1.0	IPF17430	complemer	possible zinc protease		orf19.73	17430	IPF16036	PROTEIN I	transporter activity
CA0012	0.9	1.0	0.9	IPF19448	153218..15	similar to Saccharomy		orf19.1343	19448	IPF17430	PROTEIN I	molecular_function unknown
CA0013	1.1	1.0	0.9	MAK32	164949..16	sugar kinase (by homo		orf19.4548	14749	IPF19448	Lipid fatty-a	transferase activity
CA0014	0.9	1.1	1.0	IPF13368	complemer	unknown function		orf19.1103	13368	CaMAK32	TRANSPO	molecular_function unknown
CA0015	0.9	0.9	1.0	PHO87.5E	180249..18	Member of the phosph		orf19.9990	19824	IPF13368	No significant S.c. match	
CA0016	1.0	1.0	1.1	CAN5.5	196184..19	basic-amino-acid permease, 5-prir			14582	CaPHO87.	Phosphate metabolism REGULATION OF/INTERACTION WITH CELLULAR ENVIRONMENT TRANSPORT FACILITATION	
CA0017	1.0	0.9	0.9	IPF17811	208654..20	unknown function			17811	CaCAN5.5	No significant S.c. match	
CA0018	1.0	0.9	1.0	IPF19435	complemer	unknown function			19435	IPF17811	No significant S.c. match	
CA0019	1.0	1.0	1.0	IPF17799	229761..23	unknown function			17799	IPF19435	No significant S.c. match	
CA0020	0.9	0.9	1.1	IPF7046	complemer	unknown function		orf19.124	7046	IPF17799	UNCLASSI	molecular_function unknown
CA0021	0.8	0.8	0.9	IPF19425	complemer	unknown function		orf19.161	19425	IPF7046	UNCLASSI	protein binding
CA0022	0.8	0.9	0.9	IPF19421	complemer	unknown function		orf19.162	19421	IPF19425	No significant S.c. match	
CA0023	0.9	0.9	1.1	IPF6922	complemer	unknown function		orf19.3788	6922	IPF19421	No significant S.c. match	
CA0024	1.0	1.0	1.1	RPC31.5E	271877..27	DNA-directed RNA pol		orf19.1034	14908	IPF6922	No significant S.c. match	
CA0025	1.0	0.9	1.0	IPF17945	281103..28	unknown function		orf19.178	17945	CaRPC31.	No significant S.c. match	
CA0026	0.8	0.8	0.6	IPF16843	complemer	unknown function			16843	IPF17945	No significant S.c. match	
CA0027	1.0	0.9	1.0	RCL1	345150..34	RNA 3 -terminal phosp		orf19.1886	9148	IPF16843	UNCLASSI	molecular_function unknown
CA0028	0.9	0.7	0.9	IPF15345	350274..35	peptidyl-tRNA-hydrolas		orf19.1502	15345	CaRCL1	TRANSCR	molecular_function unknown
CA0029	1.0	0.9	1.0	PET56	complemer	ribosomal RNA methyl		orf19.182	13421	IPF15345	No significant S.c. match	
CA0030	0.9	0.9	1.0	IPF17790	367177..36	unknown function		orf19.7710	17790	CaPET56	TRANSCR	transferase activity
CA0031	1.2	1.1	1.1	COX7	complemer	Subunit VII of cytochro		orf19.227	15250	IPF17790	TRANSCR	RNA binding
CA0032	1.0	1.0	1.0	IPF15248	complemer	zinc finger protein (by I		orf19.229	15248	CaCOX7	ENERGY "	oxidoreductase activity
CA0034	1.1	1.1	1.0	IPF8016	complemer	unknown function			8016	IPF15248	UNCLASSI	molecular_function unknown
CA0035	1.0	1.0	1.0	PRC3	418049..41	Carboxypeptidase Y pr		orf19.1001	7205	IPF8016	No significant S.c. match	
CA0036	0.8	5.3	0.8	IPF19377	437969..43	unknown function		orf19.3336	19377	CaPRC3	PROTEIN I	peptidase activity
CA0037	1.4	3.3	1.0	IPF17652.3	443186..44	reverse transcriptase,		orf19.6078	17652	IPF19377	No significant S.c. match	
CA0038	0.6	0.6	0.9	CYC1	451363..45	cytochrome-c isoform		orf19.1770	17424	IPF17652.3	No significant S.c. match	
CA0039	1.0	1.0	0.9	IPF3998	466972..46	unknown function		orf19.8278	3998	CaCYC1	ENERGY S	transporter activity
CA0040	1.0	0.8	1.0	IPF15632	complemer	unknown function		orf19.1097	15632	IPF3998	No significant S.c. match	
CA0041	0.9	0.9	0.9	IPF10360	complemer	unknown function		orf19.3804	10360	IPF15632	No significant S.c. match	
CA0043	0.3	0.2	1.0	HRD1	498030..49	Involved in degradatior		orf19.719	17505	IPF10360	UNCLASSI	molecular_function unknown
CA0044	1.0	0.9	0.9	IPF17347	complemer	unknown function		orf19.6492	17347	CaHRD1	PROTEIN I	ligase activity
CA0045	1.1	1.3	1.3	IPF8985.5E	complemer	unknown function, 5-pr		orf19.1368	8985	IPF17347	TRANSCRIPTION	
CA0046	1.2	1.1	1.0	IPF14850	complemer	Hypothetical protein		orf19.9988	14850	IPF8985.5e	No significant S.c. match	
CA0047	1.3	1.2	1.1	PTC2	556497..55	Protein phosphatase ty		orf19.2538	17818	IPF14850	No significant S.c. match	
CA0048	0.9	1.0	1.1	TIF4631	complemer	mRNA cap-binding pro		orf19.3599	17394	CaPTC2	CLASSIFIC	protein phosphatase activity
CA0049	1.0	1.0	1.0	IPF3908	586067..58	unknown function		orf19.757	3908	CaTIF4631	PROTEIN S	translation regulator activity
CA0050	1.0	0.9	1.1	CTA25	595830..59	transcriptional activatic		orf19.362	10947	IPF3908	UNCLASSIFIED PROTEINS	
CA0051	1.0	1.2	1.1	IPF4443	607166..60	unknown function		orf19.1217	4443	CaCTA25	No significant S.c. match	
CA0052	1.0	0.9	0.9	IPF19567	complemer	Unknown function		orf19.379	19567	IPF4443	No significant S.c. match	
CA0053	1.0	1.1	1.0	IFA16	621045..62	unknown function		orf19.380	19573	IPF19567	UNCLASSI	molecular_function unknown
CA0054	1.1	1.2	1.1	RIB4.3	complemer	6,7-dimethyl-8-ribityllumazine synt			4908	CaIFA16	Nucleotide metabolism CELL CYCLE AND DNA PROCESSING CELLULAR COMMUNICATION/SIGNAL TRANSDUCTION MECHANISM	
CA0055	1.2	1.4	1.1	IPF12442	complemer	unknown function		orf19.1091	12442	CaRIB4.3	Metabolism	isomerase activity
CA0056	0.9	0.9	0.9	IPF15839	complemer	unknown function		orf19.556	15839	IPF12442	UNCLASSI	molecular_function unknown
CA0057	1.0	1.0	1.0	IPF3767	complemer	unknown function			3767	IPF15839	No significant S.c. match	
CA0058	1.0	0.9	0.9	IPF3770	691896..69	similar to Saccharomyces cerevisi			3770	IPF3767	UNCLASSI	molecular_function unknown
CA0059	1.0	1.1	1.0	IPF17840	694776..69	unknown function		orf19.566	17840	IPF3770	CELL CYCLE AND DNA PROCESSING ""PROTEIN FATE [folding modification destination] ""CELLULAR TRANSPORT AND TRANSPOR	
CA0060	1.0	1.0	0.9	IPF13661	complemer	unknown function		orf19.1699	13661	IPF17840	UNCLASSIFIED PROTEINS	
CA0061	0.9	0.9	1.0	IPF19315.3	complemer	unknown function, 3-prime end			19316	IPF13661	UNCLASSI	molecular_function unknown
CA0062	1.0	1.0	1.1	IPF19315.5	complemer	unknown function, 5-prime end			19315	IPF19315.3	No significant S.c. match	
CA0063	1.0	0.9	1.0	IPF19310.3	719768..72	unknown function, 3-prime end			19310	IPF19315.5	No significant S.c. match	
CA0064	1.0	0.9	0.9	IPF19308.3	complemer	unknown function, 3-prime end			19308	IPF19310.3	No significant S.c. match	
CA0066	1.0	1.0	0.9	IPF15051	751455..75	unknown function		orf19.8843	15051	IPF19308.3	No significant S.c. match	
CA0067	0.9	0.9	0.9	IPF13904	756390..75	farnesyl cysteine carbo		orf19.120	13904	IPF15051	No significant S.c. match	
CA0068	1.0	0.9	1.0	ARC18	complemer	subunit of the Arp2/3 c		orf19.121	13905	IPF13904	Lipid fatty-acid and isoprenoid metabolism """"PROTEIN FATE [folding modification destination] ""SUBCELLULAR LOCALISATION	
CA0069	0.9	1.0	1.0	IPF19295.5	767511..76	unknown function, inte		orf19.6468	19295	CaARC18	CELLULAF	structural molecule activity

CA0070	1.0	1.1	0.9	IPF19295.3	768787..77	unknown function, 3-pr	orf19.6469	19294	IPF19295.5	No significant S.c. match	
CA0071	1.0	0.9	1.0	IPF19290.3	complemen	unknown function, 3-pr	orf19.621	19290	IPF19295.3	UNCLASSIFIED PROTEINS	
CA0072	0.8	0.7	0.9	IPF19283.3	complemen	unknown function, 3-pr	orf19.625	19284	IPF19290.3	No significant S.c. match	
CA0073	1.0	1.0	0.9	IPF19283.5	complemen	unknown function, 5-prime end		19283	IPF19283.3	No significant S.c. match	
CA0074	1.1	1.3	1.1	IFD7	786781..78	Putative aryl-alcohol de	orf19.629	17480	IPF19283.5	No significant S.c. match	
CA0075	0.9	0.9	1.0	IFA24.3	789556..79	unknown function, 3-pr	orf19.9164	17205	CaIFD7	C-compound and carbohydrate metabolism ENERGY	
CA0076	1.1	1.5	1.3	IPF8866	complemen	unknown function	orf19.4906	8866	CaIFA24.3	UNCLASSIFIED PROTEINS	
CA0077	1.0	1.0	1.0	IPF1239	801371..80	unknown function		1239	IPF8866	No significant S.c. match	
CA0078	1.0	1.0	1.0	CTA27	complemen	transcriptional activation		17380	IPF1239	PROTEIN FATE [folding modification destination] ""SUBCELLULAR LOCALISATION	
CA0079	0.9	0.8	0.9	IPF14323	complemen	unknown function	orf19.4730	14323	CaCTA27	No significant S.c. match	
CA0080	1.1	0.9	1.0	IPF11378	826422..82	unknown function	orf19.632	11378	IPF14323	UNCLASSI	molecular_function unknown
CA0081	0.9	0.9	1.0	IPF11379	complemen	unknown function	orf19.633	11379	IPF11378	No significant S.c. match	
CA0083	1.0	1.8	0.8	PHO84	complemen	high-affinity inorganic p	orf19.655	5438	IPF11379	No significant S.c. match	
CA0084	1.2	1.1	0.9	NAB3	complemen	polyadenylated RNA-b	orf19.1297	4338	CaPHO84	Phosphate metabolism CELLULAR TRANSPORT AND TRANSPORT MECHANISMS REGULATION OF/INTERACTION WITH CELLULAR	
CA0085	0.8	1.0	0.8	IPF16201	851158..85	unknown function	orf19.693	16201	CaNAB3	TRANSCR	molecular_function unknown
CA0086	1.1	1.0	1.2	IPF16598	complemen	unknown function	orf19.7781	16598	IPF16201	No significant S.c. match	
CA0087	1.1	0.9	0.9	SMF11	858028..85	manganese transporte	orf19.4690	14122	IPF16598	Lipid fatty-a	transferase activity
CA0088	1.0	1.0	1.0	IPF7349	866183..86	similar to Saccharomy	orf19.579	7349	CaSMF11	PROTEIN FATE [folding modification destination] ""CELLULAR TRANSPORT AND TRANSPORT MECHANISMS REGULATION OF/INTE	
CA0089	1.0	0.9	0.9	MRPL3	complemen	ribosomal protein of th	orf19.5064	19552	IPF7349	Metabolism	transferase activity
CA0090	0.8	0.6	1.0	UBP13	875277..87	ubiquitin carboxyl-term	orf19.2026	4114	CaMRPL3	PROTEIN S	structural molecule activity
CA0092	0.9	0.9	1.0	IPF8268.3f	890368..89	member of the FRP fa	orf19.1571	8269	CaUBP13	PROTEIN I	peptidase activity
CA0093	1.0	1.0	1.0	IPF19980.f	complemen	putative lipase (by hom	orf19.7747	10462	IPF8268.3f	C-compound and carbohydrate metabolism	
CA0094	0.6	0.4	0.8	IPF12819	932035..93	unknown function	orf19.3213	12819	IPF19980.r	Lipid fatty-acid and isoprenoid metabolism	
CA0095	1.0	1.0	1.0	ALG11	936850..93	required for asparagine	orf19.3468	19249	IPF12819	UNCLASSIFIED PROTEINS	
CA0096	1.1	1.0	1.0	IPF13409	complemen	unknown function	orf19.751	13409	CaALG11	C-compour	transferase activity
CA0097	1.0	0.9	1.1	IPF13290	949021..94	unknown function	orf19.5314	13290	IPF13409	UNCLASSI	molecular_function unknown
CA0098	0.8	1.0	1.0	IPF16479	954432..95	unknown function	orf19.2319	16479	IPF13290	PROTEIN SYNTHESIS SUBCELLULAR LOCALISATION	
CA0100	1.1	1.1	1.0	IPF11569	complemen	unknown function	orf19.7682	11569	IPF16479	UNCLASSI	molecular_function unknown
CA0101	1.0	1.0	1.0	IPF10105	complemen	RNA polymerase-like (	orf19.232	10105	IPF11569	No significant S.c. match	
CA0102	1.0	1.0	1.0	IPF16695	complemen	unknown function	orf19.736	16695	IPF10105	TRANSCR	transcription regulator activity
CA0103	1.2	1.1	1.0	IPF17515	1017198..1	unknown function	orf19.749	17515	IPF16695	C-compour	transcription regulator activity
CA0104	1.0	1.1	1.1	RBT4	complemen	repressed by TUP1 pro	orf19.1358	8153	IPF17515	PROTEIN FATE [folding modification destination] ""CELLULAR TRANSPORT AND TRANSPORT MECHANISMS	
CA0105	0.9	1.0	1.0	IPF17529.3	complemen	unknown function, 3-pr	orf19.786	17529	CaRBT4	CELL FATE Hypha-specific	
CA0106	1.1	1.0	1.1	IPF16308	complemen	unknown function	orf19.1133	16308	IPF17529.3	No significant S.c. match	
CA0107	1.0	1.0	1.1	POL3.5EO	complemen	DNA Polymerase III, 5-	orf19.5183	17951	IPF16308	No significant S.c. match	
CA0108	0.8	0.9	0.9	HIK1.5EOC	1035652..1	histidine kinase, 5-prim	orf19.5181	19235	CaPOL3.5e	CELL CYC	nucleotidyltransferase activity
CA0109	1.0	1.1	1.0	IPF13723	complemen	unknown function	orf19.7892	13723	CaHIK1.5e	C-compound and carbohydrate metabolism CELLULAR COMMUNICATION/SIGNAL TRANSDUCTION MECHANISM ""CELL RESCUE DE	
CA0110	1.0	1.1	1.0	IPF17190	1043744..1	unknown function	orf19.1135	17190	IPF13723	No significant S.c. match	
CA0111	0.9	0.9	1.0	IPF16830	1049792..1	similar to Saccharomy	orf19.4151	16830	IPF17190	No significant S.c. match	
CA0112	1.0	1.0	1.1	IPF17358	complemen	unknown function	orf19.5754	17358	IPF16830	Lipid fatty-a	hydrolase activity
CA0113	1.0	1.0	1.2	AUT2	1070596..1	anchor protein mediate	orf19.9938	14934	IPF17358	UNCLASSI	molecular_function unknown
CA0114	1.1	1.2	1.1	SSU72	1072101..1	suppressor of cs muta	orf19.9939	14932	CaAUT2	PROTEIN I	protein binding
CA0116	1.0	1.0	1.1	IPF17975	1082630..1	unknown function	orf19.915	17975	CaSSU72	TRANSCR	protein phosphatase activity
CA0117	1.0	1.1	1.0	IPF19231	1085355..1	unknown function	orf19.916	19231	IPF17975	No significant S.c. match	
CA0118	1.0	1.0	0.9	IPF16430.3	1088771..1	similar to Saccharomyces cerevisi		16430	IPF19231	UNCLASSI	molecular_function unknown
CA0119	0.9	0.9	1.0	RMT2	1092915..1	N-delta-arginine methy	orf19.920	16809	IPF16430.3	ENERGY S	transporter activity,oxidoreductase activity
CA0120	1.0	1.1	1.0	IPF19593	complemen	similar to Saccharomyces cerevisi		19593	CaRMT2	Amino acid	transferase activity
CA0121	0.8	0.7	1.0	IPF17048	1104885..1	unknown function	orf19.983	17048	IPF19593	PROTEIN I	peptidase activity
CA0122	1.0	1.0	1.0	SNF4	complemen	Nuclear regulatory prot	orf19.1319	6988	IPF17048	UNCLASSI	molecular_function unknown
CA0123	1.3	0.9	1.2	MXR1	1122583..1	methionine sulfoxide re	orf19.9576	4117	CaSNF4	C-compour	enzyme regulator activity
CA0124	0.9	1.2	1.0	RFC5	1123567..1	DNA replication factor	orf19.9577	4118	CaMXR1	Amino acid	oxidoreductase activity
CA0125	0.9	1.0	1.0	IPF4119	complemen	unknown function	orf19.9578	4119	CaRFC5	CELL CYC	molecular_function unknown
CA0126	1.0	1.1	1.0	IPF4284	complemen	unknown function	orf19.8177	4284	IPF4119	No significant S.c. match	
CA0127	2.7	2.6	1.6	HXK2.3F	complemen	hexokinase II, 3-prime	orf19.8176	4286	IPF4284	UNCLASSI	molecular_function unknown
CA0129	1.0	1.0	1.0	IPF10495	complemen	unknown function	orf19.246	10495	CaHXK2.3f	C-compour	transferase activity
CA0130	0.9	1.0	0.9	IPF4470	complemen	unknown function	orf19.3533	4470	IPF10495	UNCLASSI	molecular_function unknown
CA0131	1.0	0.9	1.0	IPF13402	complemen	unknown function	orf19.36	13402	IPF4470	UNCLASSI	molecular_function unknown
CA0132	0.8	0.9	0.9	SPT10	1160657..1	Transcription regulator	orf19.2361	16032	IPF13402	UNCLASSI	molecular_function unknown
CA0133	1.0	1.0	1.0	IPF3746	1166485..1	unknown function	orf19.1007	3746	CaSPT10	TRANSCR	transferase activity
CA0136	1.1	1.0	1.0	IFH1	complemen	Dioxygenase (by homo	orf19.9207	10444	IPF3746	TRANSCR	transferase activity
CA0137	0.9	1.1	1.0	SEC232	1186849..1	Component of COPII c	orf19.9206	10446	CaIFH1	CELL RESCUE DEFENSE AND VIRULENCE	
CA0138	1.1	1.1	0.7	INH1	1195101..1	Inhibitor of mitochondrial ATPase		7584	CaSEC232	CELLULAR TRANSPORT AND TRANSPORT MECHANISMS SUBCELLULAR LOCALISATION	
CA0139	1.1	1.0	1.1	RUD3	1204769..1	Suppressor of uso1-1 t	orf19.1418	17098	CaINH1	No significant S.c. match	
CA0140	1.1	1.0	1.0	MDM1.3	complemen	intermediate filament p	orf19.1009	19597	CaRUD3	CELLULAR	molecular_function unknown
CA0141	0.9	1.0	0.9	IPF17520.5	1228742..1	unknown function, inte	orf19.1021	17520	CaMDM1.3	CELL CYC	structural molecule activity
CA0142	1.0	1.0	1.1	CSL4	1235322..1	Involved in kinetochore	orf19.1026	17757	IPF17520.5	No significant S.c. match	
CA0143	1.0	1.0	1.0	IPF19195.5	1265314..1	putative amino acid or	orf19.153	19195	CaCSL4	Nucleotide	RNA binding
CA0144	1.1	1.0	1.1	IPF19195.3	1265891..1	putative amino acid or	orf19.151	17861	IPF19195.5	Lipid fatty-acid and isoprenoid metabolism ""TRANSPORT FACILITATION	
CA0145	0.8	0.7	1.0	IPF11449	1272911..1	unknown function		11449	IPF19195.3	Lipid fatty-a	molecular_function unknown
CA0146	1.0	0.9	1.0	IPF9689	1274376..1	unknown function	orf19.1113	9689	IPF11449	No significant S.c. match	
CA0147	0.8	0.8	0.8	IPF9690	complemen	unknown function	orf19.1113	9690	IPF9689	No significant S.c. match	
CA0148	1.1	1.1	1.0	POP1	1278472..1	protein component of r	orf19.2404	14929	IPF9690	UNCLASSIFIED PROTEINS	

CA0149	1.0	1.0	1.0	VPS34	1288898..1	1-phosphatidylinositol :	orf19.6243	17276	CaPOP1	TRANSCR	RNA binding
CA0150	1.0	1.0	1.0	CDC123	1296609..1	similar to Saccharomyc	orf19.1023(	6404	CaVPS34	Lipid fatty-a	protein kinase activity
CA0151	1.0	1.0	1.0	IPF15923.5	complemer	unknown function, 5-pr	orf19.177	15923	CDC123	CELL CYC	molecular_function unknown
CA0152	1.4	1.1	1.3	IPF16368.3	complemer	unknown function, 3-pr	orf19.254	16370	IPF15923.5	CELL CYCLE AND DNA PROCESSING CELL FATE SUBCELLULAR LOCALISATION	
CA0153	1.0	1.0	1.0	IPF16368.5	complemer	Unknown function, 5-p	orf19.255	16368	IPF16368.3	No significant S.c. match	
CA0154	1.0	1.8	1.1	CPH1	complemer	Transcription factor	orf19.4433	5536	IPF16368.5	No significant S.c. match	
CA0155	1.1	1.2	1.2	RPT6	complemer	26S proteasome regula	orf19.1107	13102	CaCPH1	TRANSCR	DNA binding,transcription regulator activity
CA0156	1.4	1.7	1.2	SCW1	1355380..1	glucanase (by homolog	orf19.9345	15898	CaRPT6	CELL CYC	peptidase activity
CA0157	1.0	1.0	1.0	IPF19178.3	complemer	unknown function, 3-prime end		19179	CaSCW1	CLASSIFIC	hydrolase activity
CA0158	1.1	1.0	1.0	IPF19178.5	complemer	unknown function, 3-pr	orf19.1073	19178	IPF19178.3	No significant S.c. match	
CA0159	0.9	0.9	0.9	TUB4.3	complemer	gamma-tubulin, 3-prim	orf19.1238	12541	IPF19178.5	No significant S.c. match	
CA0160	1.0	1.0	1.0	GAP7.5EO	complemer	general amino acid per	orf19.1070	11171	CaTUB4.3	CELL CYC	structural molecule activity
CA0161	0.8	0.8	0.7	RMS1	complemer	(putative) transcription	orf19.1017	10588	CaGAP7.5	Amino acid metabolism CELLULAR TRANSPORT AND TRANSPORT MECHANISMS SUBCELLULAR LOCALISATION TRANSPORT FA	
CA0162	1.0	0.9	1.0	IPF19168	complemer	unknown function	orf19.5616	19168	CaRMS1	TRANSCR	molecular_function unknown
CA0163	1.1	1.3	1.1	AYR2	1387498..1	1-acyl dihydroxyacetor	orf19.5615	15021	IPF19168	No significant S.c. match	
CA0164	1.0	0.9	1.0	IPF11694	complemer	similar to Saccharomyc	orf19.4335	11694	CaAYR2	C-compound and carbohydrate metabolism CELL FATE	
CA0165	1.0	1.0	1.0	IPF19165.3	complemer	unknown function, 3-prime end		19165	IPF11694	TRANSPO	transporter activity
CA0166	1.1	1.0	1.0	IPF19161.5	complemer	unknown function, internal fragmer		19161	IPF19165.3	No significant S.c. match	
CA0167	1.1	1.1	1.0	IPF19160	complemer	unknown function	orf19.1075	19160	IPF19161.5	No significant S.c. match	
CA0169	1.0	1.1	1.0	RBT7	complemer	repressed by TUP1	orf19.1019	14885	IPF19160	No significant S.c. match	
CA0170	1.0	1.1	1.1	IPF15959	1442925..1	unknown function	orf19.1105	15959	CaRBT7	Nucleotide metabolism	
CA0171	4.8	3.9	2.1	IPF15957	complemer	unknown function		15957	IPF15959	No significant S.c. match	
CA0172	1.0	0.9	1.0	IPF19154	1451467..1	unknown function	orf19.1126	19154	IPF15957	No significant S.c. match	
CA0173	1.0	1.0	1.0	IPF6913	complemer	unknown function	orf19.1127	6913	IPF19154	No significant S.c. match	
CA0174	1.0	1.0	1.0	IPF11182.3	1458377..1	unknown function, 3-prime end		11182	IPF6913	SUBCELLULAR LOCALISATION	
CA0175	0.8	1.0	0.8	NPL4	complemer	nuclear protein localiza	orf19.9970	19684	IPF11182.3	UNCLASSI	hydrolase activity
CA0176	0.9	1.0	1.0	IPF11388	complemer	unknown function	orf19.8267	11388	CaNPL4	PROTEIN I	protein binding,structural molecule activity
CA0177	1.0	1.0	0.9	IFL2	1473706..1	unknown function	orf19.8268	20048	IPF11388	UNCLASSI	DNA binding
CA0178	1.0	1.0	0.9	CDC6	complemer	Cell division control pro	orf19.1270	2017	CaIFL2	CELL RESCUE DEFENSE AND VIRULENCE ""CELL FATE	
CA0179	0.9	3.4	1.0	DCP1	1486194..1	mRNA decapping enzy	orf19.423	10483	CaCDC6	CELL CYC	protein binding
CA0180	0.9	1.0	1.0	SPT20	1487158..1	transcription factor, me	orf19.422	10486	CaDCP1	TRANSCR	hydrolase activity
CA0181	1.0	1.0	1.0	NOT3	complemer	unknown function	orf19.2012	15994	CaSPT20	TRANSCR	transcription regulator activity
CA0182	1.0	1.0	0.9	IFI1	1504556..1	unknown function	orf19.1130	6166	CaNOT3	TRANSCRIPTION SUBCELLULAR LOCALISATION	
CA0183	1.1	1.3	0.9	CAP1	1525251..1	transcriptional acivator	orf19.9191	4242	CaIFI1	CLASSIFICATION NOT YET CLEAR-CUT	
CA0184	1.1	1.1	0.9	IPF19142	complemer	unknown function	orf19.1146	19142	CaCAP1	TRANSCR	DNA binding,transcription regulator activity
CA0185	1.0	0.9	0.9	PLB4.5F	1545784..1	Phospholipase, 5-prim	orf19.9017	15731	IPF19142	UNCLASSIFIED PROTEINS	
CA0186	0.9	0.9	1.0	PLB4.3F	1546979..1	phospholipase, 3-prime	orf19.9018	15733	CaPLB4.5f	Lipid fatty-acid and isoprenoid metabolism ""Other virulence attributes	
CA0187	1.0	0.9	1.0	IPF15734	1548674..1	unknown function		15734	CaPLB4.3f	Lipid fatty-acid and isoprenoid metabolism ""Other virulence attributes	
CA0188	1.2	1.4	1.3	IPF15442	1550839..1	unknown function	orf19.9467	15442	IPF15734	No significant S.c. match	
CA0189	0.9	0.9	0.7	IPF12201	1556912..1	Na+-nucleoside cotran	orf19.1160	12201	IPF15442	UNCLASSI	molecular_function unknown
CA0190	1.1	1.0	1.1	IPF14773	1564584..1	unknown function	orf19.1107	14773	IPF12201	No significant S.c. match	
CA0191	1.1	1.0	1.0	PEX7	1568044..1	peroxisomal import pro	orf19.89	9594	IPF14773	UNCLASSI	molecular_function unknown
CA0192	1.1	1.1	1.1	IPF15134	1577818..1	Unknown function	orf19.1177	15134	CaPEX7	PROTEIN I	signal transducer activity
CA0193	0.9	1.0	1.0	IPF6493	1583508..1	unknown function	orf19.1541	6493	IPF15134	SUBCELLU	molecular_function unknown
CA0194	0.9	1.6	0.9	IPF16228	1589730..1	unknown function	orf19.1140	16228	IPF6493	UNCLASSIFIED PROTEINS	
CA0195	0.9	1.0	1.0	IPF12688	1590754..1	unknown function	orf19.1140	12688	IPF16228	UNCLASSI	hydrolase activity
CA0196	0.8	0.8	1.0	IPF15799	1598544..1	unknown function	orf19.1651	15799	IPF12688	Amino acid	molecular_function unknown
CA0197	1.2	1.0	1.0	IPF9787	complemer	similar to Saccharomyc	orf19.1934	9787	IPF15799	UNCLASSIFIED PROTEINS	
CA0198	1.0	1.2	0.9	IPF19685	1609514..1	unknown function	orf19.9227	19685	IPF9787	TRANSCR	DNA binding
CA0199	1.0	0.9	0.9	KAP104	complemer	karyopherin-beta prote	orf19.3556	16463	IPF19685	UNCLASSI	molecular_function unknown
CA0200	1.1	1.6	0.9	IPF15119	complemer	unknown function	orf19.9109	15119	CaKAP104	CELLULAF	nucleotidyltransferase activity
CA0201	1.0	1.0	0.9	IPF15116	complemer	Unknown function	orf19.9108	15116	IPF15119	CELLULAF	transporter activity
CA0202	1.0	0.9	1.0	IPF19126	complemer	putative aminoacid trar	orf19.1210	19126	IPF15116	UNCLASSI	molecular_function unknown
CA0203	0.9	1.1	1.1	MID1	1638166..1	involved in Ca2+ influx	orf19.1072	12820	IPF19126	TRANSPO	transporter activity
CA0204	0.8	0.9	0.8	IKI3	1641499..1	killer toxin insensitive p	orf19.1222	15693	CaMID1	REGULATI	transporter activity
CA0205	1.0	1.1	1.0	DBF2	complemer	putative ser/thr protein	orf19.1223	13853	CaIKI3	CLASSIFIC	transcription regulator activity
CA0206	0.9	0.9	1.0	IPF14089	1675193..1	putative serine/threonir	orf19.1029	14089	CaDBF2	CELL CYC	protein kinase activity
CA0207	1.0	1.0	1.0	IPF18002	1695412..1	Unknown function	orf19.1226	18002	IPF14089	ENERGY CELL CYCLE AND DNA PROCESSING SUBCELLULAR LOCALISATION	
CA0208	1.0	1.0	1.1	IPF19850	1697550..1	unknown function	orf19.1227	19850	IPF18002	UNCLASSIFIED PROTEINS	
CA0209	0.9	1.0	0.9	HAP2	1699253..1	CCAAT-binding factor	orf19.1228	16519	IPF19850	Amino acid metabolism TRANSCRIPTION SUBCELLULAR LOCALISATION UNCLASSIFIED PROTEINS	
CA0210	0.6	0.4	1.0	IPF4328	complemer	unknown function	orf19.1297	4328	CaHAP2	TRANSCR	transcription regulator activity
CA0211	1.0	1.0	1.0	IPF4326	complemer	unknown function	orf19.1297	4326	IPF4328	UNCLASSI	molecular_function unknown
CA0212	0.9	0.9	1.0	IPF4325	1705735..1	unknown function	orf19.1296	4325	IPF4326	No significant S.c. match	
CA0213	1.0	1.0	1.0	CDS1	complemer	CDP-diacylglycerol syr	orf19.1279	16312	IPF4325	No significant S.c. match	
CA0214	1.1	1.1	1.1	SCT11	1727719..1	Suppresses a choline-t	orf19.1289	9596	CaCDS1	Lipid fatty-a	nucleotidyltransferase activity
CA0215	1.0	0.9	0.9	IPF15350	1733843..1	putative zinc finger trar	orf19.2745	15350	CaSCT11	TRANSPO	transferase activity
CA0216	0.9	1.0	1.0	IPF11681	complemer	unknown function	orf19.1682	11681	IPF15350	Amino acid	transcription regulator activity,DNA binding
CA0217	1.0	0.9	1.0	MNN4	1752492..1	regulates the mannosy	orf19.849	19522	IPF11681	No significant S.c. match	
CA0218	0.9	1.0	0.9	IPF15294	1765941..1	unknown function	orf19.6238	15294	CaMNN4	PROTEIN I	molecular_function unknown
CA0219	0.9	1.1	1.0	YAP3	complemer	transcription factor of a	orf19.3193	19852	IPF15294	No significant S.c. match	
CA0220	1.0	1.0	1.0	ABZ1	complemer	para-aminobenzoate s	orf19.1291	10474	CaYAP3	TRANSCR	DNA binding,transcription regulator activity
CA0221	0.9	0.9	1.0	PUS4	1785176..1	pseudouridine synthas	orf19.9509	16835	CaABZ1	Metabolism	lyase activity

CA0222	0.9	1.0	0.9	IPF12141	1786478..1	unknown function	orf19.9508	12141	CaPUS4	Nucleotide	lyase activity
CA0223	1.0	1.0	0.9	VAS1	complemer	valyl-tRNA synthetase	orf19.1295	13980	IPF12141	UNCLASSIFIED PROTEINS	
CA0224	0.9	0.7	1.0	PRP31	complemer	pre-mRNA splicing pro	orf19.1296	13982	CaVAS1	PROTEIN S	ligase activity
CA0225	0.9	1.0	1.0	NUP84	complemer	nuclear pore protein (b	orf19.1298	16027	CaPRP31	TRANSCR	RNA binding
CA0226	1.0	1.0	1.0	IPF16024	1805331..1	unknown function	orf19.1297	16024	CaNUP84	CELLULAF	structural molecule activity
CA0227	1.1	1.0	1.0	PHO23	complemer	Involved in transcriptio	orf19.9328	6854	IPF16024	UNCLASSIFIED PROTEINS	
CA0228	0.9	0.9	0.9	IPT1	complemer	Mannosyl diphosphory	orf19.1223	11674	CaPHO23	TRANSCR	hydrolase activity
CA0229	0.9	0.9	1.0	IPF8147	1820547..1	unknown function	orf19.1358	8147	CaIPT1	Lipid fatty-a	transferase activity
CA0230	1.0	0.9	1.0	TOM37	1826653..1	Mitochondrial outer me	orf19.1532	17420	IPF8147	CELL FATE	
CA0231	1.0	1.2	1.0	RAD23	complemer	nucleotide excision rep	orf19.1494	16846	CaTOM37	No significant S.c. match	
CA0232	0.9	0.9	1.0	IFA1	1837394..1	Unknown function	orf19.156	15128	CaRAD23	CELL CYC	DNA binding
CA0233	0.8	0.6	0.9	URE2	1840615..1	Nitrogen catabolite rep	orf19.7794	15126	CaIFA1	Nucleotide metabolism CELL CYCLE AND DNA PROCESSING CELLULAR COMMUNICATION/SIGNAL TRANSDUCTION MECHANISM	
CA0234	1.0	1.0	1.0	IFA3	1842051..1	unknown function	orf19.7793	19598	CaURE2	Nitrogen ar	transcription regulator activity
CA0235	1.0	0.9	0.9	HIS3	complemer	imidazole glycerol pho	orf19.7813	13420	CaIFA3	UNCLASSI	molecular_function unknown
CA0236	1.0	0.9	1.0	MDM10	1853443..1	Involved in mitochondr	orf19.7814	13419	CaHIS3	Amino acid	lyase activity
CA0237	0.9	1.0	1.0	IPF13416	complemer	Unknown function	orf19.7818	13416	CaMDM10	SUBCELLU	molecular_function unknown
CA0238	1.0	0.9	1.0	PHA2.3	complemer	prephenate dehydratas	orf19.7864	10101	IPF13416	SUBCELLU	molecular_function unknown
CA0239	1.1	1.2	1.1	RPL9B	complemer	RPL9B ribosomal pro	orf19.236	10099	CaPHA2.3	Amino acid	lyase activity
CA0241	1.0	0.9	0.9	IPF15630	complemer	unknown function	orf19.3470	15630	CaRPL9B	PROTEIN S	structural molecule activity
CA0242	1.0	0.9	0.9	IPF12047	complemer	unknown function	orf19.3471	12047	IPF15630	UNCLASSI	molecular_function unknown
CA0243	1.1	0.9	1.0	BSD2	complemer	Metal homeostasis pro	orf19.5869	15266	IPF12047	No significant S.c. match	
CA0244	1.1	0.9	1.0	IPF19082	1890894..1	unknown function		19082	CaBSD2	REGULATI	molecular_function unknown
CA0245	1.0	1.0	1.1	POL5	1892763..1	DNA polymerase V (by	orf19.1304	17372	IPF19082	No significant S.c. match	
CA0246	0.9	1.0	1.0	IPF14452.F	1896228..1	F1-ATPase epsilon subunit (by ho		17243	CaPOL5	CLASSIFIC	nucleotidyltransferase activity
CA0247	1.1	1.1	1.1	RIB21	1900939..1	DRAP deaminase (by I	orf19.2788	7263	IPF14452.r	No significant S.c. match	
CA0248	1.3	1.0	1.2	IPF7262	complemer	unknown function	orf19.2789	7262	CaRIB21	Metabolism of vitamins cofactors and prosthetic groups	
CA0249	0.9	0.9	0.8	IPF19617	1906767..1	unknown function	orf19.1350	19617	IPF7262	No significant S.c. match	
CA0250	1.0	0.9	0.9	IPF17661	complemer	unknown function	orf19.1348	17661	IPF19617	No significant S.c. match	
CA0251	0.9	1.0	1.0	IPF9146	complemer	unknown function	orf19.1362	9146	IPF17661	No significant S.c. match	
CA0252	1.2	1.1	1.1	SBP1.5EO	complemer	RNA binding protein-lik	orf19.1327	19078	IPF9146	No significant S.c. match	
CA0253	0.9	0.9	1.0	FIL1	1921259..1	Putative mitochondrial	orf19.477	7101	CaSBP1.5e	No significant S.c. match	
CA0254	0.9	1.0	1.0	IPF14968	1939294..1	unknown function	orf19.3461	14968	CaFIL1	PROTEIN S	translation regulator activity
CA0255	0.9	0.8	0.8	IPF16558.3	1941150..1	putative transcription ir	orf19.735	16558	IPF14968	No significant S.c. match	
CA0256	1.0	1.1	1.0	IPF10309	1948932..1	unknown function	orf19.2278	10309	IPF16558.3	CELL CYCLE AND DNA PROCESSING TRANSCRIPTION SUBCELLULAR LOCALISATION	
CA0257	1.0	0.9	1.0	IPF10079	complemer	unknown function	orf19.2280	10084	IPF10309	UNCLASSI	molecular_function unknown
CA0258	1.1	1.0	1.0	IPF17112	1962316..1	thiosulfate sulfurtransfe	orf19.1356	17112	IPF10079	C-compound and carbohydrate metabolism ENERGY TRANSCRIPTION SUBCELLULAR LOCALISATION	
CA0259	1.0	0.7	1.0	SOL1	complemer	multicopy suppressor c	orf19.1355	17111	IPF17112	Nitrogen ar	transferase activity
CA0260	0.9	0.8	1.0	IPF13683	1973066..1	unknown function	orf19.2547	13683	CaSOL1	TRANSCR	molecular_function unknown
CA0261	1.0	1.0	1.0	LYS22	1981734..1	Homocitrate synthase	orf19.8394	3880	IPF13683	No significant S.c. match	
CA0262	1.3	1.3	1.1	IPF20054	1986390..1	unknown function	orf19.1407	20054	CaLYS22	Amino acid	transferase activity
CA0263	1.7	1.4	1.2	GLK1	complemer	aldohexose specific glu	orf19.1408	8388	IPF20054	UNCLASSI	molecular_function unknown
CA0264	0.9	1.0	1.0	IPF8434	1992595..1	unknown function		8434	CaGLK1	C-compour	transferase activity
CA0265	1.1	1.3	0.8	STI1	2002146..2	stress-induced protein	orf19.1070	9198	IPF8434	No significant S.c. match	
CA0266	0.9	1.1	1.0	IPF17773	complemer	unknown function		17773	CaSTI1	CELL RES	chaperone activity
CA0267	0.9	0.8	1.0	IPF12262	2020672..2	extracellular alpha-1,4-	orf19.9183	12262	IPF17773	No significant S.c. match	
CA0268	0.8	0.8	0.7	PUF2.EXO	complemer	RNA-binding protein, e	orf19.4262	15191	IPF12262	No significant S.c. match	
CA0269	1.0	1.0	1.0	PUF2.EXO	complemer	RNA-binding protein (b	orf19.4263	15189	CaPUF2.ex	CELL CYC	RNA binding
CA0270	1.1	1.4	1.1	PRB2	complemer	Protease B, vacuolar (I	orf19.9783	12896	CaPUF2.ex	No significant S.c. match	
CA0271	1.0	1.1	1.0	IPF12897	complemer	putative oxidoreductas	orf19.9785	12897	CaPRB2	PROTEIN FATE [folding modification destination] ""SUBCELLULAR LOCALISATION	
CA0272	1.0	1.1	1.1	DPB11	complemer	DNA polymerase II cor	orf19.1434	17695	IPF12897	C-compound and carbohydrate metabolism	
CA0273	1.0	1.0	0.9	IPF19066	2039965..2	unknown function	orf19.1433	19066	CaDPB11	CELL CYC	nucleotidyltransferase activity
CA0274	1.9	1.4	1.3	IPF15153	2045228..2	unknown function	orf19.1328	15153	IPF19066	UNCLASSIFIED PROTEINS	
CA0275	1.1	1.0	1.1	NTA1	complemer	Amino-terminal amidas	orf19.8470	14821	IPF15153	CELL RESCUE DEFENSE AND VIRULENCE ""SUBCELLULAR LOCALISATION	
CA0276	1.0	1.0	1.0	IPF15015	2056178..2	unknown function	orf19.1305	15015	CaNTA1	PROTEIN I	hydrolase activity
CA0277	1.8	1.2	2.0	RNH1	2059216..2	ribonuclease H (by hor	orf19.1305	12328	IPF15015	No significant S.c. match	
CA0278	1.0	0.9	1.1	IPF15772	complemer	unknown function		15772	CaRNH1	Nucleotide metabolism CONTROL OF CELLULAR ORGANIZATION	
CA0279	1.1	1.0	0.9	IPF10568	2076229..2	unknown function	orf19.1440	10568	IPF15772	No significant S.c. match	
CA0280	1.0	1.0	1.0	IPF10566	2078226..2	unknown function	orf19.1439	10566	IPF10568	No significant S.c. match	
CA0281	0.9	1.0	0.9	IPF10564.3	complemer	unknown function, 3-pr	orf19.1438	10565	IPF10566	CELLULAF	transferase activity
CA0282	0.9	1.0	1.0	IPF17417	2086004..2	Unknown function	orf19.1216	17417	IPF10564.3	No significant S.c. match	
CA0283	1.0	1.1	1.1	IPF18034	2087607..2	Unknown function		18034	IPF17417	CELL RESCUE DEFENSE AND VIRULENCE ""SUBCELLULAR LOCALISATION TRANSPORT FACILITATION UNCLASSIFIED PROTEI	
CA0284	1.0	1.1	0.9	IPF14657	2093067..2	unknown function	orf19.1441	14657	IPF18034	UNCLASSIFIED PROTEINS	
CA0285	1.1	0.9	1.0	IPF14392	2100169..2	unknown function	orf19.1535	14392	IPF14657	No significant S.c. match	
CA0286	0.6	0.9	0.3	IPF14618	complemer	unknown function	orf19.6079	14618	IPF14392	No significant S.c. match	
CA0287	0.9	1.0	0.9	IPF14615	2105364..2	unknown function	orf19.6080	14615	IPF14618	No significant S.c. match	
CA0288	1.0	1.0	1.1	IPF14614	2107184..2	unknown function		14614	IPF14615	CELL CYCLE AND DNA PROCESSING	
CA0289	1.1	1.1	1.2	IPF13333	complemer	unknown function	orf19.1590	13333	IPF14614	No significant S.c. match	
CA0290	1.0	0.9	1.1	POT14	complemer	acetyl-CoA acetyltrans	orf19.1591	13331	IPF13333	No significant S.c. match	
CA0291	0.9	1.0	1.1	IPF13328	2112387..2	unknown function	orf19.1592	13328	CaPOT14	Lipid fatty-a	transferase activity
CA0292	1.1	1.0	1.0	IPF19855	complemer	unknwon function	orf19.1384	19855	IPF13328	UNCLASSI	molecular_function unknown
CA0293	1.2	0.9	1.1	IPF6624	complemer	unknown function	orf19.6489	6624	IPF19855	No significant S.c. match	
CA0294	1.0	1.0	0.9	IPF16061	2125621..2	unknown function	orf19.9322	16061	IPF6624	UNCLASSI	molecular_function unknown

CA0295	1.0	1.0	0.9	IPF15033	complemer	unknown function	orf19.1595	15033	IPF16061	UNCLASSI	isomerase activity
CA0296	1.0	0.9	0.9	IFA11	complemer	Unknown function	orf19.1596	15031	IPF15033	UNCLASSI	molecular_function unknown
CA0297	1.1	1.0	1.0	CDC53	2137417..2	Cell division control pro	orf19.1674	14223	CaIFA11	Nucleotide metabolism CELL CYCLE AND DNA PROCESSING CELLULAR COMMUNICATION/SIGNAL TRANSDUCTION MECHANISM	
CA0298	1.0	1.0	1.0	PPT1	complemer	Protein ser/thr phospha	orf19.1673	14222	CaCDC53	Amino acid	structural molecule activity
CA0299	1.0	0.9	1.0	IPF3765	2142629..2	unknown function	orf19.1597	3765	CaPPT1	CLASSIFIC	protein phosphatase activity
CA0300	0.9	0.9	1.0	ERG24	complemer	C-14 sterol reductase (	orf19.1598	3764	IPF3765	UNCLASSI	molecular_function unknown
CA0301	1.1	1.0	1.0	IPF19688	complemer	unknown function	orf19.1606	19688	CaERG24	Lipid fatty-a	oxidoreductase activity
CA0302	0.9	0.9	1.2	MEP3	2164073..2	low affinity high capaci	orf19.9181	12620	IPF19688	No significant S.c. match	
CA0303	1.1	0.9	1.0	IPF17507	complemer	putative glutathione S-	orf19.8339	17507	CaMEP3	TRANSPO	transporter activity
CA0304	1.0	1.1	1.0	IPF12829	complemer	unknown function	orf19.8340	12829	IPF17507	Nitrogen and sulphur metabolism	
CA0305	1.1	1.1	1.0	INO80	2176470..2	DNA helicase (by hom	orf19.1734	13578	IPF12829	UNCLASSI	molecular_function unknown
CA0306	0.9	1.0	0.9	HEM3	2181626..2	porphobilinogen deami	orf19.1742	15629	CaINO80	TRANSCR	hydrolase activity
CA0307	1.0	1.0	1.0	IPF4719	complemer	similar to Saccharomy	orf19.3736	4719	CaHEM3	Metabolism	transferase activity
CA0308	1.1	1.1	1.0	RHK1	complemer	Mannosyltransferase (l	orf19.8693	17442	IPF4719	CELL CYC	transcription regulator activity
CA0309	1.1	1.1	1.0	IPF16935	complemer	unknown function	orf19.1091	16935	CaRHK1	C-compour	transferase activity
CA0310	1.0	1.0	0.8	PEX11	complemer	peroxisomal membran	orf19.8690	19049	IPF16935	PROTEIN I	molecular_function unknown
CA0311	0.9	1.0	1.0	DAK2.3EO	2199549..2	dihydroxyacetone kinase, 3-prime		19048	CaPEX11	SUBCELLU	molecular_function unknown
CA0312	1.0	1.0	1.0	IPF6396	complemer	unknown function	orf19.8211	6396	CaDAK2.3	C-compound and carbohydrate metabolism ""CELL RESCUE DEFENSE AND VIRULENCE ""	
CA0315	0.9	0.9	0.8	ALS9.5EO	complemer	agglutinin-like protein,	orf19.5742	19856	IPF6396	UNCLASSI	molecular_function unknown
CA0316	1.0	1.1	0.9	ALS1.3EO	complemer	agglutinin-like protein,	orf19.5741	13002	CaALS9.5e	SUBCELLULAR LOCALISATION Other virulence attributes	
CA0317	1.1	1.1	1.0	SSD1	2223396..2	Protein phosphatase (t	orf19.3959	5791	CaALS1.3e	SUBCELLULAR LOCALISATION Other virulence attributes	
CA0318	1.0	1.0	1.0	FOL2	2227680..2	GTP cyclohydrolase (b	orf19.3957	5793	CaSSD1	CELL CYC	RNA binding
CA0319	1.0	1.0	1.0	IPF15244.3	complemer	unknown function, 3-prime end		19858	CaFOL2	Metabolism	hydrolase activity
CA0321	1.1	1.0	1.1	APL2	2234514..2	AP-1 complex subunit,	orf19.7861	15242	IPF15244.3	No significant S.c. match	
CA0322	1.0	1.1	1.0	RPS9B	2242781..2	Ribosomal protein	orf19.8459	3402	CaAPL2	PROTEIN I	protein binding
CA0323	1.2	1.1	1.1	CDC95	2245800..2	translation initiation fac	orf19.9378	12555	CaRPS9B	PROTEIN S	structural molecule activity
CA0324	1.0	1.1	0.9	STT4	2247127..2	Phosphatidylinositol-4-	orf19.9377	14337	CaCDC95	PROTEIN S	molecular_function unknown
CA0325	1.1	1.1	1.1	IPF15222	complemer	Unknown function	orf19.3781	15222	CaSTT4	Lipid fatty-a	transferase activity
CA0326	0.9	1.0	1.0	IPF15220	2255216..2	Unknown function	orf19.3780	15220	IPF15222	UNCLASSIFIED PROTEINS	
CA0327	1.0	1.0	0.9	IPF15217.5	2256723..2	WD-repeat protein, 5-p	orf19.3779	15219	IPF15220	No significant S.c. match	
CA0328	1.0	1.0	1.1	IPF15217.3	2257498..2	WD-repeat protein, 3-p	orf19.3778	15217	IPF15217.5	SUBCELLULAR LOCALISATION	
CA0329	1.0	1.0	1.0	IMG2	2258435..2	Required for Integrity c	orf19.3777	15216	IPF15217.3	SUBCELLU	molecular_function unknown
CA0330	0.9	0.6	1.2	BAT21	complemer	branched-chain amino	orf19.797	5075	CaIMG2	CONTROL	structural molecule activity
CA0331	1.1	1.0	1.0	ANC1	2264913..2	TFIIF subunit, transcrip	orf19.798	5074	CaBAT21	Amino acid	transferase activity
CA0332	0.9	0.9	0.8	FEN2	2271840..2	allantoate permease tr	orf19.1298	14381	CaANC1	Phosphate	transcription regulator activity
CA0334	0.9	0.9	0.9	IPF19562	complemer	unknown function	orf19.7824	19562	CaFEN2	Amino acid	transporter activity
CA0335	1.1	1.1	1.1	IFA2	2277979..2	unknown function	orf19.7825	16389	IPF19562	No significant S.c. match	
CA0336	1.0	0.9	1.0	IPF9869	2284147..2	unknown function	orf19.1371	9869	CaIFA2	Nucleotide metabolism CELL CYCLE AND DNA PROCESSING CELLULAR COMMUNICATION/SIGNAL TRANSDUCTION MECHANISM	
CA0337	0.9	1.1	0.9	IPF9867	complemer	unknown function	orf19.1370	9867	IPF9869	TRANSCRIPTION SUBCELLULAR LOCALISATION	
CA0338	0.9	1.0	0.9	IPF13252	2291654..2	unknown function	orf19.3378	13252	IPF9867	No significant S.c. match	
CA0339	1.0	0.9	0.9	IPF13247	2293360..2	unknown function	orf19.3376	13247	IPF13252	No significant S.c. match	
CA0340	1.0	0.9	0.9	IPF19859	2295931..2	unknown function	orf19.3375	19859	IPF13247	No significant S.c. match	
CA0341	1.0	1.3	1.1	XKS1	2298575..2	xylulokinase (by homol	orf19.1788	10472	IPF19859	No significant S.c. match	
CA0342	0.9	0.9	1.0	IPF10470	2300711..2	unknown function	orf19.1789	10470	CaXKS1	C-compour	transferase activity
CA0343	1.0	0.9	1.0	LYS1.5EO	2302869..2	saccharopine dehydrogenase, 5-p		10469	IPF10470	UNCLASSI	molecular_function unknown
CA0344	1.0	1.1	1.1	IFF1.3EOC	complemer	Unknown function, 3-p	orf19.1346	16164	CaLYS1.5e	Amino acid	oxidoreductase activity
CA0345	1.3	1.3	1.1	ACH1	complemer	acetyl-coenzyme-A hy	orf19.1068	12009	CaIFF1.3e	No significant S.c. match	
CA0346	1.2	2.1	1.4	ROX1	2327367..2	Possible heme-depenc	orf19.1034	19029	CaACH1	Lipid fatty-a	hydrolase activity
CA0347				FOX2	2337294..2340014				CaROX1	Metabolism	DNA binding
CA0348	0.9	0.9	1.0	IFK1	complemer	probable monoxygenas	orf19.9098	15750			
CA0349	1.0	0.9	1.2	IPF19026	complemer	unknown function	orf19.1813	19026	CaIFK1	No significant S.c. match	
CA0350	0.9	1.0	1.0	SNU114	complemer	snRNP-specific proteir	orf19.7784	16740	IPF19026	UNCLASSI	molecular_function unknown
CA0351	1.0	0.9	1.0	RPB4.3F	complemer	DNA-directed RNA pol	orf19.145	19621	CaSNU114	PROTEIN S	RNA binding,helicase activity
CA0352	1.0	0.9	0.8	RPB4.5F	complemer	DNA-directed RNA pol	orf19.146	19620	CaRPB4.3f	TRANSCR	nucleotidyltransferase activity
CA0353	0.9	1.0	1.0	YAK1.3F	complemer	serine/threonine protei	orf19.147	16043	CaRPB4.5f	TRANSCRIPTION ""CELL RESCUE DEFENSE AND VIRULENCE ""SUBCELLULAR LOCALISATION	
CA0354	1.0	1.2	1.0	YAK1.5F	complemer	serine/threonine protei	orf19.7788	16042	CaYAK1.3f	CELL CYC	protein kinase activity
CA0355	0.9	0.7	0.9	IPF2535	complemer	unknown function	orf19.1395	2535	CaYAK1.5f	No significant S.c. match	
CA0356	0.9	1.0	1.0	IPF2532	2378711..2	unknown function	orf19.1395	2532	IPF2535	No significant S.c. match	
CA0357	1.2	1.2	1.2	FCY22	complemer	purine-cytosine perme	orf19.333	12764	IPF2532	UNCLASSI	molecular_function unknown
CA0358	0.9	0.8	1.0	IPF17488.5	2397615..2	unknown function, 5-pr	orf19.1818	17488	CaFCY22	Nucleotide	transporter activity
CA0359	1.0	1.0	1.0	IPF17488.3	2398456..2	unknown function, 3-pr	orf19.1820	17489	IPF17488.5	No significant S.c. match	
CA0360	1.0	1.0	1.0	IPF2328	complemer	unknown function	orf19.8720	2328	IPF17488.3	No significant S.c. match	
CA0361	0.9	0.8	0.9	IPF2326	2404599..2	unknown function	orf19.1124	2326	IPF2328	No significant S.c. match	
CA0362	0.7	0.6	1.2	TEF1	complemer	translation elongation f	orf19.9009	9732	IPF2326	No significant S.c. match	
CA0363	1.0	1.0	1.1	SPR3	2414124..2	sporulation-specific se	orf19.1524	16732	CaTEF1	PROTEIN S	translation regulator activity
CA0364	1.0	1.0	1.0	IPF15983	2416756..2	unknown function	orf19.1525	15983	CaSPR3	CELL FATE SUBCELLULAR LOCALISATION	
CA0365	1.0	0.9	1.0	IPF15177	complemer	Unknown function	orf19.750	15177	IPF15983	Nucleotide	molecular_function unknown
CA0366	1.0	1.0	1.0	IPF19017	2443661..2	unknown function	orf19.8931	19017	IPF15177	No significant S.c. match	
CA0367	1.1	0.9	1.0	TIM22	complemer	Mitochondrial import in	orf19.1352	16416	IPF19017	No significant S.c. match	
CA0368	0.9	1.3	0.8	IPF11646	2446413..2	unknown function	orf19.1353	11646	CaTIM22	PROTEIN I	transporter activity
CA0369	1.0	1.1	1.0	IPF13675	complemer	unknown function	orf19.1656	13675	IPF11646	No significant S.c. match	
CA0370	1.1	1.2	1.2	IPF13678	complemer	unknown function		13678	IPF13675	UNCLASSIFIED PROTEINS	

CA0371	1.0	1.0	1.0	IPF19578	complemer	unknown function	orf19.1087	19578	IPF13678	CLASSIFIC	molecular_function unknown
CA0372	1.1	1.1	1.0	IPF10062	complemer	unknown function	orf19.1086	10062	IPF19578	No significant S.c. match	
CA0373	1.0	0.9	1.0	IPF7655.5E	complemer	unknown function, 5-prime end		7655	IPF10062	UNCLASSI	molecular_function unknown
CA0374	0.9	1.1	0.9	IFK3	complemer	Probable monooxygen	orf19.8477	7651	IPF7655.5€	No significant S.c. match	
CA0375	1.1	1.5	1.2	CRH11	2480849..2	Probable membrane pi	orf19.2706	14360	CaIFK3	No significant S.c. match	
CA0376	1.1	1.2	1.2	QCR9	2482622..2	ubiquinol--cytochrome-c reductase		14357	CaCRH11	SUBCELLL	molecular_function unknown
CA0377	0.9	0.9	0.7	IPF14356	2483232..2	unknown function	orf19.2708	14356	CaQCR9	ENERGY S	transporter activity,oxidoreductase activity
CA0378	1.0	1.1	1.0	IPF3178	complemer	Unknown function	orf19.3170	3178	IPF14356	UNCLASSI	molecular_function unknown
CA0379	1.0	1.0	1.0	SSP120	2494755..2	secretory protein (by h	orf19.3173	12012	IPF3178	UNCLASSI	molecular_function unknown
CA0380	0.8	0.8	0.8	IPF14109	2512372..2	unknown function	orf19.2451	14109	CaSSP120	CELLULAF	molecular_function unknown
CA0381	0.8	0.9	0.7	IPF16047	complemer	unknown function	orf19.2825	16047	IPF14109	No significant S.c. match	
CA0382	1.0	1.0	1.0	IPF16030	2528963..2	similar to Saccharomy	orf19.1842	16030	IPF16047	UNCLASSI	molecular_function unknown
CA0383	0.9	0.8	1.0	IPF16028	2533990..2	unknown function	orf19.1841	16028	IPF16030	CELL FATE	signal transducer activity
CA0384	0.9	1.1	0.8	PCL1	2549651..2	cyclin, G1/S-specific (b	orf19.1017	16634	IPF16028	Lipid fatty-acid and isoprenoid metabolism ""CELL CYCLE AND DNA PROCESSING PROTEIN SYNTHESIS CELLULAR COMMUNICATIC	
CA0385	1.1	1.0	0.9	IPF4071	complemer	unknown function	orf19.1861	4071	CaPCL1	CELL CYC	protein kinase activity,enzyme regulator activity
CA0386	1.6	1.6	1.2	IPF4065	2557981..2	unknown function	orf19.1862	4065	IPF4071	CELL CYCLE AND DNA PROCESSING SUBCELLULAR LOCALISATION	
CA0387	1.2	1.1	1.1	IPF10318.5	complemer	unknown function, 5-prime end		14475	IPF4065	UNCLASSI	molecular_function unknown
CA0388	0.8	0.9	0.8	IPF14468	2564217..2	unknown function	orf19.1907	14468	IPF10318.5	No significant S.c. match	
CA0389	1.1	1.1	1.1	IPF14465	complemer	unknown function	orf19.1910	14465	IPF14468	UNCLASSI	molecular_function unknown
CA0390	1.0	1.0	0.9	DCG1	complemer	involved in nitrogen-ca	orf19.244	10499	IPF14465	No significant S.c. match	
CA0391	1.0	1.0	1.0	DDC1	2572793..2	DNA damage checkpo	orf19.7875	10496	CaDCG1	Nitrogen ar	molecular_function unknown
CA0393	1.1	0.9	1.0	IPF12758.3	complemer	unknown function, 3-prime end		12759	CaDDC1	CELL CYC	molecular_function unknown
CA0394	1.1	1.0	1.2	IPF12758.5	complemer	unknown function, 5-pr	orf19.7966	12758	IPF12758.3	No significant S.c. match	
CA0395	1.0	1.0	1.0	SEF11.5EC	complemer	Putative transcription f	orf19.1926	15112	IPF12758.5	No significant S.c. match	
CA0396	0.9	1.1	1.0	SNM1	2596774..2	RNA binding protein of	orf19.1927	15109	CaSEF11.5	TRANSCRIPTION CELL FATE SUBCELLULAR LOCALISATION	
CA0397	1.1	1.1	1.1	FRE31	complemer	Ferric reductase (by hc	orf19.1930	15107	CaSNM1	No significant S.c. match	
CA0398	1.1	1.3	1.1	ARO2	2602824..2	chorismate synthase (t	orf19.1986	14673	CaFRE31	REGULATI	oxidoreductase activity
CA0399	1.1	1.0	1.1	CDC45	complemer	Chromosomal DNA rep	orf19.1988	14671	CaARO2	Amino acid	lyase activity
CA0400	1.0	1.0	1.0	IPF7423.3E	2608466..2	similar to Saccharomy	orf19.6223	11910	CaCDC45	CELL CYC	DNA binding
CA0401	0.9	0.9	0.9	RTA1	complemer	unknown function	orf19.6224	11914	IPF7423.3€	CELL FATE	
CA0402	0.9	0.7	0.9	IPF15225	complemer	Sorting nexin-like prote	orf19.1990	15225	CaRTA1	UNCLASSIFIED PROTEINS	
CA0403	1.1	1.1	1.1	IPF15224	complemer	unknown function	orf19.1989	15224	IPF15225	PROTEIN FATE [folding modification destination]	
CA0404	1.0	1.0	1.0	LYS14	2624704..2	transcription factor inv	orf19.5548	14435	IPF15224	UNCLASSI	molecular_function unknown
CA0405	0.9	1.0	0.9	IPF19862.3	complemer	unknown function, 3-pr	orf19.1299	19861	CaLYS14	Amino acid	transcription regulator activity
CA0407				SAC6.3F	complement(2627330..2627737)				IPF19862.3	No significant S.c. match	
CA0408	1.3	1.5	1.2	SAC6.5F	complemer	actin filament bundling	orf19.5544	8093			
CA0409	0.9	0.9	1.0	PHO80	2633763..2	Cyclin (by homology)	orf19.5755	15067	CaSAC6.5	CELLULAF	protein binding
CA0410	1.1	1.0	1.1	IPF15065	complemer	unknown function	orf19.1318	15065	CaPHO80	Phosphate	protein kinase activity,enzyme regulator activity
CA0411	1.0	0.9	0.9	IPF17676	2640129..2	similar to Saccharomy	orf19.1507	17676	IPF15065	UNCLASSI	molecular_function unknown
CA0413	1.0	1.2	1.2	ALS2	2649386..2	agglutinin-like protein,	orf19.2122	18994	IPF17676	SUBCELLL	protein binding
CA0414	1.1	1.0	1.1	IPF4068	complemer	reverse transcriptase	orf19.2164	4068	CaALS2	Other virulence attributes	
CA0415	0.6	0.7	0.4	FRP3	2666833..2	member of the FRP fai	orf19.1224	16160	IPF4068	No significant S.c. match	
CA0416	1.0	1.0	0.9	IPF17492	complemer	unknown function	orf19.1225	17492	CaFRP3	C-compour	transporter activity
CA0417	1.2	1.4	1.1	UBC13	2678425..2	E2 ubiquitin-conjugatin	orf19.2225	6529	IPF17492	No significant S.c. match	
CA0418	1.0	1.0	1.0	IPF9051.5E	complemer	unknown function, 5-pr	orf19.2227	9051	CaUBC13	PROTEIN FATE [folding modification destination]	
CA0419	1.0	1.0	0.9	IPF19864	2683154..2	unknown function	orf19.2228	19864	IPF9051.5€	CELL CYCLE AND DNA PROCESSING ""PROTEIN FATE [folding modification destination] ""CONTROL OF CELLULAR ORGANIZATION	
CA0420	0.8	1.0	0.9	IPF9048	complemer	unknown function	orf19.2229	9048	IPF19864	CELL FATE	protein binding
CA0421	1.0	1.0	1.0	IPF9047	2686402..2	unknown function	orf19.2230	9047	IPF9048	UNCLASSI	molecular_function unknown
CA0422	0.6	0.8	0.5	IPF9046	complemer	unknown function	orf19.2231	9046	IPF9047	UNCLASSI	DNA binding,transcription regulator activity
CA0423	1.1	1.3	1.1	IPF15273	complemer	unknown function	orf19.9381	15273	IPF9046	No significant S.c. match	
CA0424	1.0	0.9	1.1	IPF10795	complemer	putative transcription fa	orf19.2356	10795	IPF15273	Amino acid metabolism Nitrogen and sulphur metabolism ""Lipid fatty-acid and isoprenoid metabolism ""CELL CYCLE AND DNA PROCES	
CA0425	1.0	1.1	1.1	PPH21	2710813..2	protein ser/thr phospha	orf19.9252	11679	IPF10795	TRANSCRIPTION ""CELL RESCUE DEFENSE AND VIRULENCE ""REGULATION OF/INTERACTION WITH CELLULAR ENVIRONMENT	
CA0426	0.8	1.0	0.9	CFT1	complemer	pre-mRNA 3 -end proc	orf19.2760	11996	CaPPH21	C-compour	protein phosphatase activity
CA0427	1.0	1.0	1.0	IPF11995	2720571..2	unknown function	orf19.2761	11995	CaCFT1	TRANSCR	RNA binding
CA0428	1.0	1.1	1.1	ILV2	2723926..2	acetolactate synthase	orf19.1613	14829	IPF11995	Lipid fatty-a	transferase activity
CA0429	1.0	1.0	1.0	IPF14827	complemer	unknown function	orf19.1611	14827	CaILV2	Amino acid	transferase activity
CA0430	1.3	1.4	1.3	PRC1	complemer	Carboxypeptidase Y pi	orf19.1339	7928	IPF14827	No significant S.c. match	
CA0431	1.1	0.9	1.0	IPF7927	2734025..2	putative aldose reducta	orf19.1340	7927	CaPRC1	PROTEIN I	peptidase activity
CA0432	1.0	1.0	1.0	IPF7926	2735092..2	putative protein kinase	orf19.1341	7926	IPF7927	C-compour	molecular_function unknown
CA0433	1.0	1.1	1.1	SHM1	2736327..2	Serine hydroxymethylti	orf19.1342	7924	IPF7926	CLASSIFICATION NOT YET CLEAR-CUT	
CA0434	1.1	1.0	1.0	IPF13577	complemer	unknown function	orf19.9303	13577	CaSHM1	Nucleotide	transferase activity
CA0435	1.5	1.4	2.0	UGP1	2743481..2	UTP--glucose-1-phosp	orf19.9305	15411	IPF13577	No significant S.c. match	
CA0436	1.1	1.0	1.1	RBL2	complemer	Beta-tubulin binding protein (by hc		15412	CaUGP1	C-compour	nucleotidyltransferase activity
CA0437	1.1	1.1	1.0	DIT1	complemer	Spore wall maturation	orf19.9308	15414	CaRBL2	CELL CYC	chaperone activity
CA0438	0.9	0.8	1.0	STE4.5EO	complemer	GTP-binding protein be	orf19.8419	19618	CaDIT1	CELL FATE	
CA0439	0.9	0.9	0.9	IPF12478	2757659..2	unknown function	orf19.1301	12478	CaSTE4.5€	CELLULAF	hydrolase activity
CA0440	1.0	1.1	1.1	IPF12473	2759972..2	unknown function	orf19.5571	12473	IPF12478	UNCLASSI	molecular_function unknown
CA0441	0.9	1.0	1.0	IPF12472	complemer	unknown function	orf19.1301	12472	IPF12473	No significant S.c. match	
CA0442	0.6	0.8	0.5	IFC4	2766149..2	unknown function	orf19.2292	12066	IPF12472	C-compound and carbohydrate metabolism ""PROTEIN FATE [folding modification destination] ""CONTROL OF CELLULAR ORGANIZATI	
CA0443	1.0	1.0	0.9	SPB1	complemer	Putative methyltransfe	orf19.7727	19691	CaIFC4	TRANSPORT FACILITATION	
CA0444	1.1	1.0	1.1	IPF17429.3	complemer	similar to Saccharomy	orf19.75	16249	CaSPB1	TRANSCR	transferase activity
CA0445	0.9	1.0	1.0	IPF17429.5	complemer	similar to Saccharomy	orf19.7725	17429	IPF17429.3	CELLULAF	molecular_function unknown

CA0446	1.0	0.9	0.8	IPF14282	complemer	Similar to mucin protei	orf19.2296	14282	IPF17429.5	UNCLASSIFIED PROTEINS	
CA0447	1.1	1.0	0.9	IPF14275	complemer	Probable ADP-ribosyla	orf19.2297	14275	IPF14282	No significant S.c. match	
CA0448	1.8	2.5	5.4	ALS10	2802350..2	agglutinin like protein	orf19.2355	12997	IPF14275	PROTEIN I	hydrolase activity
CA0449	1.0	1.0	0.9	MOT2.3	complemer	transcriptional repress	orf19.2379	9357	CaALS10	CELL FATE SUBCELLULAR LOCALISATION Other virulence attributes	
CA0450	1.0	1.0	1.1	IPF9353	2818634..2	unknown function	orf19.2378	9353	CaMOT2.3	UNCLASSI	transcription regulator activity
CA0451	1.0	1.0	1.0	IPF13556	2822970..2	unknown function	orf19.8882	13556	IPF9353	TRANSPO	molecular_function unknown
CA0452	1.1	1.1	1.1	IPF13554	2824363..2	Hypothetical acidic pro	orf19.8881	13554	IPF13556	No significant S.c. match	
CA0453	1.0	1.0	1.0	IPF13552	complemer	putative methyltransfer	orf19.8880	13552	IPF13554	UNCLASSIFIED PROTEINS	
CA0454	1.1	1.0	1.1	RPN6	2826397..2	subunit of the regulato	orf19.8879	13551	IPF13552	UNCLASSI	transferase activity
CA0455	1.0	0.9	1.0	TCA5A	2830521..2	polyprotein of Tca5 ret	orf19.2427	11655	CaRPN6	PROTEIN I	structural molecule activity
CA0457	0.9	1.5	1.0	IPF15604	2842182..2	transcription factor (by	orf19.2432	15604	CaTca5a	UNCLASSIFIED PROTEINS	
CA0458	1.1	0.9	1.0	IPF15601	2843873..2	unknown function	orf19.2433	15601	IPF15604	Lipid fatty-a	transcription regulator activity
CA0459	1.0	0.9	1.0	HOL3	2846242..2	member of major facilit	orf19.2517	4969	IPF15601	No significant S.c. match	
CA0460	1.1	1.1	1.0	SEC17	complemer	transport vesicle fusior	orf19.2518	4971	CaHOL3	CELL RESCUE DEFENSE AND VIRULENCE ""TRANSPORT FACILITATION	
CA0461	1.0	0.9	1.0	IPF4972	2849425..2	unknown function	orf19.2519	4972	CaSEC17	CELLULAF	transporter activity
CA0462	1.0	1.1	0.9	MRPS28	complemer	ribosomal protein (by h	orf19.2520	4973	IPF4972	CELL CYC	molecular_function unknown
CA0463	1.1	1.1	1.1	IPF18979	complemer	unknown function	orf19.7797	18979	CaMRPS2	PROTEIN	RNA binding
CA0464	0.7	0.5	0.9	IPF14116	2853561..2	unknown function	orf19.7798	14116	IPF18979	Metabolism of vitamins cofactors and prosthetic groups	
CA0465	0.9	1.0	0.9	IPF14113	2856442..2	unknown function	orf19.7800	14113	IPF14116	UNCLASSI	hydrolase activity
CA0466	1.0	0.9	1.0	URA4	complemer	dihydrooratase (by hor	orf19.1977	8687	IPF14113	TRANSCR	molecular_function unknown
CA0467	0.8	1.0	1.0	TRX2	complemer	thioredoxin (by homolc	orf19.1976	8686	CaURA4	Nucleotide	hydrolase activity
CA0468	1.0	1.0	1.0	DIB1	2866091..2	Component of the U4/l	orf19.9531	8685	CaTRX2	CELL CYCLE AND DNA PROCESSING ""PROTEIN FATE [folding modification destination] """"CELL RESCUE DEFENSE AND VIRULEN	
CA0469	0.9	1.0	1.0	IPF18977	2869617..2	similar to Saccharomy	orf19.2621	18977	CaDIB1	CELL CYC	RNA binding
CA0470	1.1	1.2	1.1	YPT32	complemer	small GTP-binding pro	orf19.2622	12928	IPF18977	TRANSCR	RNA binding
CA0471	1.0	1.0	1.0	ECM22	complemer	putative protein involve	orf19.2623	12926	CaYPT32	CELLULAF	hydrolase activity
CA0472	1.0	1.0	1.1	STL1	2879598..2	sugar transporter (by h	orf19.1317	19866	CaECM22	Lipid fatty-acid and isoprenoid metabolism ""TRANSCRIPTION	
CA0473	1.0	0.9	0.9	GRP1	2886031..2	dihydroflavonol-4-redu	orf19.1224	12888	CaSTL1	C-compour	transporter activity
CA0474	1.0	1.2	1.0	IPF12887	complemer	Putative multidrug prot	orf19.1224	12887	CaGRP1	Metabolism of vitamins cofactors and prosthetic groups	
CA0475	1.0	1.2	1.0	IPF2580	2891145..2	unknown function	orf19.8085	2580	IPF12887	TRANSPORT FACILITATION	
CA0476	1.0	0.9	0.9	UBP2	complemer	Ubiquitin-specific prote	orf19.8088	2578	IPF2580	TRANSCR	transcription regulator activity
CA0477	0.8	0.8	0.8	IPF13839	2901451..2	unknown function	orf19.6017	13839	CaUBP2	PROTEIN I	peptidase activity
CA0478	1.1	1.0	1.0	YAE8	2903317..2	GTP-binding protein (b	orf19.1343	13843	IPF13839	No significant S.c. match	
CA0479	1.0	1.1	1.1	IPF16191	2912423..2	similar to Saccharomy	orf19.2630	16191	CaYAE8	PROTEIN I	molecular_function unknown
CA0480	1.0	1.0	1.0	IPF8610	complemer	permease (by homolog	orf19.2633	8610	IPF16191	CELL CYC	protein binding
CA0481	1.1	1.0	0.9	STE14	2922192..2	farnesyl cystein carbox	orf19.7766	14764	IPF8610	C-compound and carbohydrate metabolism CELLULAR TRANSPORT AND TRANSPORT MECHANISMS SUBCELLULAR LOCALISATIO	
CA0482	0.9	1.1	1.0	IPF14763	complemer	delta-12 fatty acid desa	orf19.7765	14763	CaSTE14	Lipid fatty-a	transferase activity
CA0483	0.9	0.9	0.9	SEC9	complemer	transport protein (by hc	orf19.7764	19868	IPF14763	No significant S.c. match	
CA0484	1.0	1.0	1.0	IPF19622	2928945..2	unknown function	orf19.7762	19622	CaSEC9	CELLULAF	transporter activity
CA0485	1.0	1.1	1.1	IPF17574	2936416..2	Unknown function	orf19.1103	17574	IPF19622	No significant S.c. match	
CA0486	0.9	1.0	1.0	IPF15540	2949943..2	unknown function	orf19.258	13725	IPF17574	UNCLASSI	molecular_function unknown
CA0487	1.0	1.1	1.0	IPF13724	2951078..2	unknown function	orf19.259	13724	IPF15540	No significant S.c. match	
CA0488	0.9	0.9	1.0	SEC59	complemer	Dolichol kinase (by hor	orf19.261	13722	IPF13724	No significant S.c. match	
CA0489	1.0	4.9	0.9	IPF6960	2957473..2	unknown function	orf19.2636	6960	CaSEC59	Lipid fatty-a	transferase activity
CA0490	0.9	1.0	0.9	COS161	2958234..2	involved in manganese	orf19.2637	6961	IPF6960	UNCLASSIFIED PROTEINS	
CA0491	0.8	0.9	1.0	IPF18966	2965098..2	unknown function		18966	CaCOS161	REGULATION OF/INTERACTION WITH CELLULAR ENVIRONMENT SUBCELLULAR LOCALISATION	
CA0492	1.0	1.0	0.9	SNU71	complemer	Associated with U1 snl	orf19.9068	14405	IPF18966	No significant S.c. match	
CA0493	0.9	1.0	0.9	PRP39.3	2967769..2	pre-mRNA splicing fac	orf19.1492	14401	CaSNU71	TRANSCR	RNA binding
CA0494	0.9	1.1	1.0	RAD7	complemer	nucleotide excision rep	orf19.9070	14400	CaPRP39.3	TRANSCR	RNA binding
CA0495	1.0	0.4	0.4	IPF20056	2975622..2	unknown function	orf19.2659	20057	CaRAD7	CELL CYC	DNA binding
CA0496	1.0	0.8	1.0	IPF11644	complemer	unknown function	orf19.2660	11644	IPF20056	UNCLASSI	molecular_function unknown
CA0498	1.0	1.0	1.0	CTA211.3F	2982842..2	transcriptional activato	orf19.2661	11534	IPF11644	UNCLASSI	transferase activity
CA0499	1.0	1.0	1.0	IFA9	2983962..2	unknown function	orf19.2663	11533	CaCTA211	No significant S.c. match	
CA0500	1.0	1.0	1.0	IPF20058	2997081..2	unknown function	orf19.4793	20058	CaIFA9	Nucleotide metabolism CELL CYCLE AND DNA PROCESSING CELLULAR COMMUNICATION/SIGNAL TRANSDUCTION MECHANISM	
CA0501	1.0	1.0	1.0	IPF16501	complemer	unknown function	orf19.1225	16501	IPF20058	UNCLASSI	molecular_function unknown
CA0502	1.0	1.0	0.9	IPF16761	3004358..3	unknown function	orf19.2664	16761	IPF16501	TRANSCRIPTION CELLULAR TRANSPORT AND TRANSPORT MECHANISMS SUBCELLULAR LOCALISATION	
CA0503	1.1	1.0	1.1	PMT6	complemer	protein mannosyltransf	orf19.3802	9088	IPF16761	TRANSPO	molecular_function unknown
CA0504	1.0	1.0	1.1	IPF16466	complemer	unknown function	orf19.2778	16466	CaPMT6	C-compound and carbohydrate metabolism ""PROTEIN FATE [folding modification destination] ""	
CA0505	1.0	1.1	1.0	IPF11988.3	complemer	unknown function, 3-prime end		18957	IPF16466	UNCLASSI	molecular_function unknown
CA0506	1.0	1.0	0.9	IPF11988	complemer	unknown function	orf19.3626	11988	IPF11988.3	No significant S.c. match	
CA0507	1.0	1.0	1.0	IPF11989	complemer	unknown function	orf19.3625	11989	IPF11988	Amino acid	molecular_function unknown
CA0508	1.0	1.0	1.0	IPF11991	complemer	similar to Saccharomy	orf19.1110	11991	IPF11989	PROTEIN FATE [folding modification destination]	
CA0509	0.9	0.9	0.9	CDC24	3045005..3	GTP/GDP exchange fa	orf19.1068	19624	IPF11991	Nucleotide	RNA binding
CA0510	1.0	0.9	0.9	ETF1	3048333..3	ETF-UBIQUINONE OX	orf19.1068	16916	CaCDC24	CELL CYC	signal transducer activity
CA0511	1.1	1.1	1.1	IPF13975	3057583..3	unknown function	orf19.4953	13975	CaETF1	ENERGY	oxidoreductase activity
CA0512	1.0	1.0	1.0	VMA16	complemer	H+-ATPase 23 KD sub	orf19.4954	13974	IPF13975	UNCLASSI	molecular_function unknown
CA0513	1.0	1.0	1.1	IPF13972	3060211..3	unknown function	orf19.4955	13972	CaVMA16	SUBCELLU	transporter activity
CA0514	1.2	0.9	1.1	BEM1	3064900..3	bud emergence media	orf19.4645	13602	IPF13972	CELL CYC	molecular_function unknown
CA0516	0.9	1.0	0.9	IPF11526	3073275..3	unknown function	orf19.2804	11526	CaBEM1	CELL CYC	protein binding
CA0517	1.0	0.9	1.1	HEM13	complemer	by homology S. cerev.	orf19.2803	11525	IPF11526	No significant S.c. match	
CA0519	0.9	1.0	0.9	MRPL10.3	3081012..3	ribosomal protein, 3-pr	orf19.1101	19871	CaHEM13	Metabolism	oxidoreductase activity
CA0520	1.1	0.9	1.0	IPF4466	complemer	unknown function	orf19.3531	4466	CaMRPL10	PROTEIN	structural molecule activity
CA0521	0.8	0.4	0.9	CKA2	3083229..3	casein kinase II alpha	orf19.3530	4465	IPF4466	UNCLASSIFIED PROTEINS	

CA0522	1.1	1.0	1.1	IPF4463	complemer	unknown function	orf19.1101	4463	CaCKA2	CELL CYC	protein kinase activity
CA0523	0.9	0.8	0.9	IPF4459	3087654..3	unknown function	orf19.1101	4459	IPF4463	CELLULAR TRANSPORT AND TRANSPORT MECHANISMS SUBCELLULAR LOCALISATION TRANSPORT FACILITATION UNCLASS	
CA0524	1.0	1.1	0.9	CYT12	3089174..3	cytochrome-c1 (by hon	orf19.1101	18952	IPF4459	UNCLASSI	molecular_function unknown
CA0525	1.0	1.0	1.0	MSB1	3093273..3	Morphogenesis-relatec	orf19.8726	6172	CaCYT12	ENERGY SUBCELLULAR LOCALISATION	
CA0526	1.0	0.9	0.9	BUB3	complemer	cell cycle arrest proteir	orf19.2655	10586	CaMSB1	CELL FATI	molecular_function unknown
CA0528	1.0	1.0	1.1	ANP1	complemer	Golgi mannosyltransfe	orf19.3622	5178	CaBUB3	CELL CYC	molecular_function unknown
CA0529	0.9	0.8	0.9	SMC2	complemer	chromosome segregat	orf19.3623	11993	CaANP1	PROTEIN I	transferase activity
CA0530	0.9	0.9	1.0	IPF19633.	3132995..3	similar to Saccharomy	orf19.8685	19634	CaSMC2	CELL CYC	DNA binding
CA0531	1.0	1.1	1.1	UBC1	3139627..3	ubiquitin-conjugating e	orf19.8686	10066	IPF19633.3	TRANSCR	RNA binding
CA0532	0.9	1.0	0.9	IRE1	3142571..3	protein kinase (by hom	orf19.5068	16157	CaUBC1	PROTEIN FATE [folding modification destination] ""CELLULAR TRANSPORT AND TRANSPORT MECHANISMS CELL FATE SUBCELLU	
CA0533	1.1	1.0	1.0	IPF19872	complemer	unknown function	orf19.5067	19872	CaIRE1	Lipid fatty-a	protein kinase activity
CA0534	1.1	1.1	1.1	IPF15607	3147427..3	unknown function	orf19.5066	15607	IPF19872	UNCLASSI	molecular_function unknown
CA0535	0.9	1.0	0.9	IPF15606	3150263..3	similar to Saccharomy	orf19.5065	15606	IPF15607	UNCLASSI	molecular_function unknown
CA0536	1.0	1.0	1.0	PANC.3	3153095..3	pantothenate syntheta	orf19.2815	18946	IPF15606	PROTEIN I	molecular_function unknown
CA0537	1.0	1.0	0.9	PRP16	complemer	RNA-dependent ATPa	orf19.2818	19698	CaPANC.3	Metabolism	ligase activity
CA0538	1.0	1.0	1.0	IPF10901	3157112..3	unknown function	orf19.2819	10901	CaPRP16	TRANSCR	RNA binding,helicase activity
CA0539	0.9	1.1	0.9	NUP57	complemer	nuclear pore protein (b	orf19.2820	10900	IPF10901	No significant S.c. match	
CA0540	0.6	0.8	0.4	IPF10896	complemer	NADH dehydrogenase	orf19.2821	10896	CaNUP57	CELLULAF	structural molecule activity
CA0541	1.0	0.9	0.9	IPF14574	complemer	unknown function	orf19.1083	14574	IPF10896	No significant S.c. match	
CA0543	1.2	0.9	0.9	IPF3255	complemer	similar to Saccharomy	orf19.2867	3255	IPF14574	UNCLASSI	molecular_function unknown
CA0544	1.1	1.0	1.1	IPF3252	3175336..3	unknown function	orf19.2868	3252	IPF3255	PROTEIN I	transporter activity
CA0545	1.0	1.0	1.1	IPF3251	3176156..3	unknown function	orf19.2869	3251	IPF3252	No significant S.c. match	
CA0546	1.0	1.0	1.0	IPF3250	3176959..3	unknown function	orf19.2870	3250	IPF3251	No significant S.c. match	
CA0548	1.0	1.0	1.0	PHO87	3183582..3	Member of the phosph	orf19.2454	14853	IPF3250	No significant S.c. match	
CA0549	1.0	1.1	1.0	IPF14031	3188241..3	putative secreted aspa	orf19.852	14031	CaPHO87	Phosphate transporter activity	
CA0550	1.1	1.1	1.0	IPF14030	complemer	unknown function	orf19.851	14030	IPF14031	PROTEIN FATE [folding modification destination] ""SUBCELLULAR LOCALISATION	
CA0551	1.3	1.4	1.1	CDC37	complemer	Cell division control pro	orf19.5531	4339	IPF14030	PROTEIN FATE [folding modification destination]	
CA0552	1.0	1.2	1.0	IPF4343	complemer	similar to Saccharomy	orf19.5533	4343	CaCDC37	CELL CYC	chaperone activity
CA0553	1.0	1.1	1.0	IPF16653	complemer	unknown function	orf19.5534	16653	IPF4343	PROTEIN I	enzyme regulator activity
CA0554	0.7	0.8	0.7	HAS1	3205905..3	ATP-DEPENDENT RN	orf19.1144	5784	IPF16653	UNCLASSI	molecular_function unknown
CA0555	1.0	1.0	1.0	HYS2	3207834..3	DNA-directed DNA pol	orf19.3960	5785	CaHAS1	CELL CYC	molecular_function unknown
CA0556	1.0	1.1	1.0	IPF15920	complemer	zinc-finger containing p	orf19.4972	15920	CaHYS2	CELL CYC	nucleotidyltransferase activity
CA0557	1.2	1.2	1.0	HYR1.53	3223440..3	Hyphally regulated protein, interna		7957	IPF15920	TRANSCRIPTION SUBCELLULAR LOCALISATION	
CA0558	1.0	1.4	1.0	GPX2	3225137..3	glutathione peroxidase	orf19.85	13205	CaHYR1.5	Hypha-specific	No significant S.c. match
CA0559	1.0	1.5	1.1	GPX1	3227250..3	glutathione peroxidase	orf19.86	13207	CaGPX2	CELL RESCUE DEFENSE AND VIRULENCE	
CA0560	1.0	0.9	0.9	GPX3	3229232..3	glutathione peroxidase	orf19.87	13211	CaGPX1	CELL RES	oxidoreductase activity
CA0561	1.0	1.0	1.0	CIN4	complemer	GTP-binding protein	orf19.2925	15237	CaGPX3	CELL RESCUE DEFENSE AND VIRULENCE	
CA0562	0.9	1.0	1.0	PSO2	3232781..3	Interstrand crosslink re	orf19.2926	15236	CaCIN4	CELL CYCLE AND DNA PROCESSING SUBCELLULAR LOCALISATION	
CA0563	1.0	1.0	1.1	MNN11	complemer	Mannosyltransferase c	orf19.2927	14216	CaPSO2	CELL CYC	DNA binding
CA0565	1.1	1.1	0.9	IPF13526	complemer	unknown function	orf19.6250	13526	CaMNN11	PROTEIN I	transferase activity
CA0566	0.9	1.2	1.0	IPF13522	3242173..3	unknown function	orf19.6252	13522	IPF13526	CELLULAF	molecular_function unknown
CA0567	1.8	1.9	1.4	RPS23	complemer	Ribosomal protein S23	orf19.1363	13519	IPF13522	SUBCELLU	transcription regulator activity
CA0568	1.1	1.1	1.1	APS2	3246330..3	AP-2 complex subunit,	orf19.8729	16570	CaRPS23	PROTEIN S	structural molecule activity
CA0569	1.0	1.0	0.9	CDC8	complemer	dTMP kinase (by homo	orf19.8730	16571	CaAPS2	PROTEIN I	molecular_function unknown
CA0570	0.7	0.8	0.7	SVL3	3250629..3	Involved in vacuole fur	orf19.8732	17015	CaCDC8	Nucleotide	transferase activity
CA0571	1.1	0.9	1.0	CDC43	3256714..3	geranylgeranyltransfer	orf19.9369	14866	CaSVL3	CELL FATI	molecular_function unknown
CA0572	0.9	0.8	0.9	IPF14864	3258387..3	unknown function	orf19.9370	18938	CaCDC43	Lipid fatty-a	signal transducer activity
CA0573	0.4	0.5	0.3	PEX14	3259960..3	peroxisomal protein (by	orf19.9371	15186	IPF14864	CELLULAF	transporter activity
CA0574	1.1	1.3	1.5	IPF15183	3262553..3	unknown function	orf19.9373	15183	CaPEX14	PROTEIN I	protein binding
CA0575	0.9	0.9	0.9	IPF15178	3264478..3	Unknown function	orf19.1808	15178	IPF15183	No significant S.c. match	
CA0576	1.0	1.0	1.0	NPR1	3271396..3	nitrogen permease rea	orf19.6232	12521	IPF15178	No significant S.c. match	
CA0577	0.9	0.8	1.0	IPF16051	3276393..3	unknown function	orf19.3068	16051	CaNPR1	Nitrogen and sulphur metabolism CELLULAR TRANSPORT AND TRANSPORT MECHANISMS	
CA0578	1.0	1.1	1.0	IPF9605	3283180..3	similar to Saccharomy	orf19.3071	9605	IPF16051	TRANSCRIPTION	
CA0579	0.9	1.0	1.0	IPF3184.E	3296789..3	unknown function, exo	orf19.3560	3184	IPF9605	CELL CYC	protein phosphatase activity
CA0580	1.0	0.9	0.9	IPF3184.E	3297542..3	unknown function, exo	orf19.3559	3182	IPF3184.e	UNCLASSIFIED PROTEINS	
CA0581	1.0	1.0	1.0	IPF3181	3298373..3	similar to Saccharomy	orf19.3558	3181	IPF3184.e	UNCLASSI	structural molecule activity
CA0582	1.0	0.9	0.9	IPF3180	3299065..3	unknown function	orf19.1104	3180	IPF3181	CELLULAF	molecular_function unknown
CA0583	1.1	1.1	1.0	GSH1.EXC	complemer	gamma-glutamylcystei	orf19.1252	12218	IPF3180	UNCLASSI	molecular_function unknown
CA0584	1.0	1.0	1.1	GSH1.EXC	complemer	Gamma-glutamylcyste	orf19.1252	18932	CaGSH1.e	CELL RES	ligase activity
CA0585	1.1	1.3	1.1	ADE5,7	complemer	phosphoribosylamine-g	orf19.1252	13495	CaGSH1.e	CELL RESCUE DEFENSE AND VIRULENCE	
CA0586	1.1	1.1	1.2	IPF13493	3310418..3	Unknown function	orf19.1252	17932	CaADE5,7	Nucleotide	ligase activity
CA0587	1.1	1.1	1.0	SPO14.5E	complemer	phospholipase D, 5-pri	orf19.8753	13085	IPF13493	PROTEIN FATE [folding modification destination] ""CELLULAR TRANSPORT AND TRANSPORT MECHANISMS SUBCELLULAR LOCAL	
CA0588	1.0	1.1	1.1	IPF13081	complemer	unknown function	orf19.1162	13081	CaSPO14.	Lipid fatty-a	hydrolase activity
CA0589	1.0	1.0	1.0	IPF13080	complemer	unknown function		13080	IPF13081	No significant S.c. match	
CA0590	1.0	1.0	1.0	IPF13079	3317420..3	unknown function	orf19.8756	13079	IPF13080	UNCLASSIFIED PROTEINS	
CA0591	1.0	0.9	1.0	ALS3.5EO	complemer	agglutinin-like protein,	orf19.1816	12561	IPF13079	UNCLASSI	molecular_function unknown
CA0592	1.0	1.1	1.0	IPF8862	complemer	unknown function	orf19.1237	8862	CaALS3.5e	CELL FATI	signal transducer activity
CA0593	1.0	1.0	1.0	ARP6	3333180..3	actin-related protein (b	orf19.4904	8861	IPF8862	No significant S.c. match	
CA0594	1.0	0.9	0.9	IPF8860	complemer	similar to Saccharomy	orf19.1236	8860	CaARP6	SUBCELLU	molecular_function unknown
CA0595	1.0	1.0	0.9	IPF8856	complemer	unknown function	orf19.1236	8856	IPF8860	Lipid fatty-a	hydrolase activity
CA0596	1.0	0.9	1.0	IPF9706	3349430..3	unknown function	orf19.1234	9706	IPF8856	UNCLASSIFIED PROTEINS	
CA0597	1.0	1.0	0.9	IPF9704	3352289..3	similar to Saccharomy	orf19.1234	9704	IPF9706	UNCLASSI	molecular_function unknown

CA0598	1.1	1.0	1.0	IPF9703	complemer	unknown function	orf19.1234	9703	IPF9704	TRANSCR	transcription regulator activity
CA0599	1.0	1.0	0.9	IPF12428	3357819..3	unknown function	orf19.1018	12428	IPF9703	No significant S.c. match	
CA0600	0.9	0.9	0.9	MSN5.5F	3359459..3	Importin-beta family m	orf19.2666	12426	IPF12428	UNCLASSI	RNA binding
CA0601	1.0	0.9	1.0	MSN5.3F	3361884..3	Importin-beta family m	orf19.1018	18929	CaMSN5.5	CELLULAF	protein binding
CA0602	1.0	1.0	1.0	CTA22	3369449..3	Protein with putative tr	orf19.3074	1708	CaMSN5.3	CELLULAR TRANSPORT AND TRANSPORT MECHANISMS CELL FATE	
CA0603	1.0	1.0	1.1	IPF1709	complemer	unknown function	orf19.3076	1709	CaCTA22	No significant S.c. match	
CA0604	1.0	1.1	1.0	VID21	3370884..3	unknown function	orf19.3077	1713	IPF1709	CLASSIFIC	molecular_function unknown
CA0605	1.2	1.7	1.1	UTR2	3376805..3	1,3-1,4-beta-glucanase	orf19.9240	8080	CaVID21	UNCLASSI	molecular_function unknown
CA0606	1.1	1.1	1.1	COP1	3378711..3	coatomer complex alpl	orf19.9241	8079	CaUTR2	CONTROL	molecular_function unknown
CA0609	0.6	0.5	0.6	CDR11.3F	complemer	multidrug resistance pr	orf19.919	12978	CaCOP1	CELLULAF	molecular_function unknown
CA0610	1.0	0.9	1.0	CDR11.5F	complemer	multidrug resistance pr	orf19.8533	12975	CaCDR11.	Lipid fatty-acid and isoprenoid metabolism """"CELL RESCUE DEFENSE AND VIRULENCE ""REGULATION OF/INTERACTION WITH CE	
CA0611	1.1	1.1	1.1	IPF17542	3400215..3	unknown function	orf19.1302	17542	CaCDR11.	Lipid fatty-acid and isoprenoid metabolism """"CELL RESCUE DEFENSE AND VIRULENCE ""REGULATION OF/INTERACTION WITH CE	
CA0612	1.1	1.1	1.1	IPF17545	complemer	unknown function	orf19.5579	17545	IPF17542	No significant S.c. match	
CA0614	1.2	1.0	1.0	IPF19700	3410866..3	unknown function	orf19.1600	19700	IPF17545	No significant S.c. match	
CA0615	2.0	3.6	1.4	RPL3	3413519..3	60S large subunit ribos	orf19.1601	6742	IPF19700	No significant S.c. match	
CA0616	1.0	0.9	1.0	PHO11	complemer	Secreted acid phospha	orf19.2619	7796	CaRPL3	PROTEIN S	structural molecule activity
CA0617	1.1	1.1	1.5	MET2	complemer	Homoserine O-acetyltr	orf19.2618	7797	CaPHO11	Phosphate	hydrolase activity
CA0618	0.8	0.8	0.9	UGT51	complemer	UDP-glucose:sterol glu	orf19.2616	7800	CaMET2	Amino acid	transferase activity
CA0619	1.1	1.0	1.0	IPF18924	3427421..3	unknown function	orf19.3146	18924	CaUGT51	Lipid fatty-a	transferase activity
CA0620	1.1	1.0	1.0	IPF17998	3428371..3	unknown function	orf19.3147	17998	IPF18924	No significant S.c. match	
CA0621	1.0	1.0	1.0	IPF17999	complemer	unknown function	orf19.3148	17999	IPF17998	No significant S.c. match	
CA0622	1.0	2.1	1.3	IPF14171	complemer	unknown function	orf19.3149	14171	IPF17999	No significant S.c. match	
CA0623	1.3	1.2	1.2	MSS4.3	3440139..3	phosphatidylinositol-4-	orf19.3153	15141	IPF14171	UNCLASSI	molecular_function unknown
CA0624	0.9	1.2	1.0	IPF312	3442861..3	unknown function	orf19.3154	15139	CaMSS4.3	Lipid fatty-a	transferase activity
CA0625	1.0	1.0	0.9	IPF19637	3448212..3	unknown function		19638	IPF312	UNCLASSI	molecular_function unknown
CA0626	0.9	1.0	0.7	IPF3161	complemer	Unknown function	orf19.3161	3161	IPF19637	No significant S.c. match	
CA0627	2.0	2.1	1.0	HSP12	complemer	Heat shock protein (by	orf19.3160	3160	IPF3161	UNCLASSI	transcription regulator activity
CA0628	1.1	1.1	1.1	IPF3878	3458867..3	unknown function	orf19.773	3878	CaHSP12	C-compour	chaperone activity
CA0629	1.1	1.2	1.1	IPF3876	3460112..3	unknown function	orf19.775	3876	IPF3878	UNCLASSI	molecular_function unknown
CA0630	1.1	1.0	1.0	IPF3875.5I	3463174..3	unknown function, 5-pr	orf19.776	3875	IPF3876	REGULATION OF/INTERACTION WITH CELLULAR ENVIRONMENT	
CA0631	1.0	0.9	1.0	IPF11698	3468528..3	similar to Saccharomy	orf19.1181	11698	IPF3875.5	CELL CYCLE AND DNA PROCESSING CELL FATE	
CA0632	1.0	1.7	1.0	RPS5	complemer	ribosomal protein S5.e	orf19.1181	11696	IPF11698	TRANSPO	transporter activity
CA0633	1.0	1.0	1.0	IPF16269	complemer	unknown function	orf19.9340	16269	CaRPS5	PROTEIN S	structural molecule activity
CA0634	0.9	0.9	1.0	IPF16267	3477624..3	unknown function	orf19.1772	16267	IPF16269	UNCLASSI	molecular_function unknown
CA0635	1.1	1.0	1.1	IPF9036	complemer	similar to Saccharomy	orf19.9342	9036	IPF16267	UNCLASSI	molecular_function unknown
CA0636	1.3	1.7	1.0	FDH4.3F	complemer	Formate dehydrogenase, 3-prime		18026	IPF9036	Nitrogen ar	DNA binding
CA0639	0.8	1.1	1.0	FDH13.3F	complemer	Putative formate dehydrogenase, 3		18922	CaFDH4.3	ENERGY	
CA0641	1.2	1.2	1.1	IPF15977	complemer	unknown function	orf19.4698	15977	CaFDH13.3	ENERGY	
CA0642	1.0	1.0	1.3	ERG25	complemer	C-4 sterol methyl oxida	orf19.3732	9179	IPF15977	No significant S.c. match	
CA0643	0.9	1.0	0.8	IDP2	complemer	isocitrate dehydrogena	orf19.3733	9177	CaERG25	Lipid fatty-a	oxidoreductase activity
CA0644	1.0	1.1	1.0	IPF17131	3520884..3	unknown function	orf19.69	17131	CaIDP2	C-compour	oxidoreductase activity
CA0645	1.0	0.9	1.0	IPF18917.5	3523342..3	unknown function, 5-prime end		18917	IPF17131	No significant S.c. match	
CA0646	0.9	0.8	0.9	IPF19702	3524828..3	unknown function	orf19.1180	19702	IPF18917.5	PROTEIN FATE [folding modification destination] ""CELLULAR TRANSPORT AND TRANSPORT MECHANISMS	
CA0647	2.1	0.7	1.2	IPF19706	complemer	unknown function	orf19.4334	19706	IPF19702	No significant S.c. match	
CA0648	0.9	0.9	0.9	IPF12399	3540983..3	unknown function	orf19.3210	12399	IPF19706	No significant S.c. match	
CA0649	0.8	0.9	1.0	RFC3	complemer	DNA replication factor	orf19.3211	12401	IPF12399	No significant S.c. match	
CA0650	1.0	1.0	1.0	IPF11566	3546264..3	unknown function	orf19.11	11566	CaRFC3	CELL CYC	DNA binding
CA0651	1.1	1.0	0.9	ALK8	3547768..3	n-alkane inducible cytc	orf19.10	11568	IPF11566	No significant S.c. match	
CA0652	1.0	0.8	1.1	SHP1	complemer	potential regulatory sul	orf19.1008	11553	CaALK8	CELL RESCUE DEFENSE AND VIRULENCE ""CELL FATE CONTROL OF CELLULAR ORGANIZATION	
CA0653	1.6	1.9	3.0	MET6	complemer	BY HOMOLOGY TO S	orf19.1008	11555	CaSHP1	ENERGY C	molecular_function unknown
CA0654	1.0	0.9	1.1	IPF11550.3	complemer	Ca2+-transporting P-ty	orf19.1008	11557	CaMET6	Amino acid	transferase activity
CA0655	1.0	0.9	1.0	IPF11560.5	complemer	Ca2+-transporting P-ty	orf19.1008	11560	IPF11550.3	CELLULAR TRANSPORT AND TRANSPORT MECHANISMS REGULATION OF/INTERACTION WITH CELLULAR ENVIRONMENT SUB	
CA0656	1.1	1.1	1.0	IPF12470	complemer	unknown function	orf19.5573	12470	IPF11560.5	CELLULAR TRANSPORT AND TRANSPORT MECHANISMS REGULATION OF/INTERACTION WITH CELLULAR ENVIRONMENT SUB	
CA0657	0.9	0.9	0.9	IPF19877	3576492..3	putative cysteine synth	orf19.5574	19877	IPF12470	No significant S.c. match	
CA0658	1.0	0.9	1.0	IPF16505	complemer	Unknown function	orf19.5575	16505	IPF19877	Amino acid	lyase activity
CA0659	0.9	0.9	1.0	IPF16189.3	complemer	panthotenate kinase, 3	orf19.5576	16507	IPF16505	UNCLASSI	molecular_function unknown
CA0660	1.2	1.1	1.1	IPF16189.5	complemer	panthotenate kinase, 5	orf19.5577	16189	IPF16189.3	UNCLASSIFIED PROTEINS	
CA0661	0.9	1.0	1.0	APG7	3586042..3	component of the auto	orf19.8326	12207	IPF16189.5	UNCLASSI	transferase activity
CA0662	1.0	1.0	1.0	SPC97	3588160..3	spindle pole body com	orf19.708	12206	CaAPG7	CONTROL OF CELLULAR ORGANIZATION SUBCELLULAR LOCALISATION	
CA0663	1.0	0.9	0.9	PUP2	complemer	20S proteasome subur	orf19.709	12205	CaSPC97	CELL CYC	structural molecule activity
CA0664	1.0	1.1	2.0	LSC2.3EO	complemer	succinate-CoA ligase b	orf19.710	12204	CaPUP2	CELL CYC	peptidase activity
CA0665	1.0	1.1	0.9	INT1	3593265..3	integrin-like protein alp	orf19.1173	19580	CaLSC2.3	C-compour	ligase activity
CA0666	1.0	1.0	1.0	FIP1	3598777..3	Component of pre-mRI	orf19.1173	18911	CaINT1	CELL FATE SUBCELLULAR LOCALISATION Other virulence attributes	
CA0667	1.2	1.7	1.2	TIF5	complemer	Translation initiation fa	orf19.1173	15194	CaFIP1	TRANSCR	RNA binding
CA0669	1.0	0.8	0.9	IPF5918	3603864..3	unknown function	orf19.448	5918	CaTIF5	PROTEIN S	translation regulator activity,enzyme regulator activity
CA0670	1.0	1.1	1.1	IPF5915	3606013..3	phosphatidyl synthase	orf19.449	5915	IPF5918	UNCLASSI	molecular_function unknown
CA0671	1.2	1.2	1.2	GRP4	complemer	putative reductase (by	orf19.1066	15555	IPF5915	UNCLASSI	molecular_function unknown
CA0672	1.0	0.9	1.1	GRP6	complemer	Putative reductase (by	orf19.3151	15804	CaGRP4	Metabolism of vitamins cofactors and prosthetic groups	
CA0673	0.8	0.8	0.8	AMO2	complemer	amine oxidase (by hon	orf19.3152	15803	CaGRP6	Metabolism of vitamins cofactors and prosthetic groups	
CA0674	0.4	0.2	0.7	IPF19608	complemer	unknown function	orf19.8770	19608	CaAMO2	CELL CYCLE AND DNA PROCESSING	
CA0675	1.0	1.1	1.0	IPF13098	3623987..3	unknown function	orf19.8771	13098	IPF19608	No significant S.c. match	
CA0676	1.1	1.0	1.1	IPF13097	3625442..3	unknown function	orf19.8772	13097	IPF13098	UNCLASSI	molecular_function unknown

CA0677	1.2	1.1	1.2	IPF13095	complemer	unknown function	orf19.8773	13095	IPF13097	Lipid fatty-acid and isoprenoid metabolism ""CELL CYCLE AND DNA PROCESSING CELL FATE	
CA0678	1.3	1.5	1.2	IPF13094	3627562..3	unknown function	orf19.8774	13094	IPF13095	CELLULAF	transporter activity
CA0679	1.1	1.0	1.1	IPF13885.F	3629816..3	unknown function, 5-prime end		13092	IPF13094	UNCLASSI	molecular_function unknown
CA0680	1.0	1.0	1.1	IPF14511.5	3632428..3	unknown function, 5-pr	orf19.1216	14511	IPF13885.r	No significant S.c. match	
CA0681	1.0	0.9	1.0	IPF14511.3	3633712..3	unknown function, 3-pr	orf19.1217	14513	IPF14511.5	UNCLASSI	hydrolase activity
CA0682	1.0	1.0	1.0	IPF14514	complemer	unknown function	orf19.1217	14514	IPF14511.3	UNCLASSIFIED PROTEINS	
CA0683	1.0	1.0	1.1	IPF14519	3636213..3	unknown function	orf19.1217	14519	IPF14514	TRANSCR	transcription regulator activity
CA0684	1.0	1.2	0.9	IPF4450	complemer	unknown function	orf19.1217	4450	IPF14519	No significant S.c. match	
CA0685	1.1	1.0	1.0	ADO1	complemer	adenosine kinase (by h	orf19.1303	6804	IPF4450	No significant S.c. match	
CA0686	0.9	0.8	0.8	IPF162	complemer	sulphate transporter (b	orf19.3222	162	CaADO1	Nucleotide	transferase activity
CA0687	1.0	1.2	1.0	CPA2	3660069..3	arginine-specific carba	orf19.3221	4967	IPF162	UNCLASSI	molecular_function unknown
CA0688	1.0	0.9	1.0	IPF10555.3	3664415..3	unknown function, 3-pr	orf19.1215	18902	CaCPA2	Amino acid	ligase activity
CA0689	1.1	1.3	1.1	IPF14119	3669098..3	unknown function	orf19.1215	14119	IPF10555.3	C-compound and carbohydrate metabolism ENERGY	
CA0690	1.0	1.0	0.9	IPF18901.3	3671035..3	unknown function, 3-prime end		18901	IPF14119	No significant S.c. match	
CA0691	0.8	0.9	0.8	CIRT4A	3682647..3	Transposase (by homo	orf19.1035	15077	IPF18901.3	No significant S.c. match	
CA0692	1.7	2.4	1.4	PGM2	3684723..3	Phosphoglucomutase (	orf19.1035	15073	CaCirt4a	No significant S.c. match	
CA0693	1.0	1.2	0.9	IPF12606.5	complemer	unknown function, 5-pr	orf19.3288	12606	CaPGM2	C-compour	isomerase activity
CA0694	1.0	0.9	0.9	IPF12603	complemer	unknown function	orf19.3289	12603	IPF12606.5	UNCLASSI	molecular_function unknown
CA0695	0.9	1.0	1.0	IPF12601	3697815..3	unknown function	orf19.3290	12601	IPF12603	No significant S.c. match	
CA0696	1.0	1.0	1.1	HMT1	3699135..3	hnRNP methyltransfera	orf19.3291	12599	IPF12601	No significant S.c. match	
CA0697	1.1	1.0	1.0	VTC4	complemer	putative polyphosphate	orf19.3363	15753	CaHMT1	PROTEIN I	transferase activity
CA0700	1.1	1.1	1.2	SHR3	3709534..3	endoplasmatic reticulu	orf19.3366	10396	CaVTC4	UNCLASSI	molecular_function unknown
CA0701	0.9	1.0	1.0	IPF10397	3710871..3	unknown function		10397	CaSHR3	PROTEIN I	chaperone activity
CA0702	0.9	1.0	1.0	IPF11812	3712258..3	unknown function	orf19.1043	11812	IPF10397	No significant S.c. match	
CA0703	1.1	1.0	1.0	IPF11807	complemer	unknown function	orf19.2914	11807	IPF11812	CELLULAF	molecular_function unknown
CA0704	1.0	1.0	1.0	IPF11806	3716310..3	similar to Saccharomy	orf19.2915	11806	IPF11807	No significant S.c. match	
CA0705	1.0	1.0	1.0	IPF11804	3718709..3	unknown function	orf19.1043	11804	IPF11806	PROTEIN I	hydrolase activity
CA0706	2.7	2.4	1.1	IPF7715	3720359..3	unknown function	orf19.8300	7715	IPF11804	No significant S.c. match	
CA0707	0.8	0.8	0.8	IPF7711	complemer	related to Neurospora	orf19.8298	7711	IPF7715	CELL FATE	
CA0709	1.1	1.0	1.1	ZORRO2A	complemer	Reverse transcriptase,	orf19.3387	3477	IPF7711	No significant S.c. match	
CA0711	1.1	1.3	1.0	VMA5	complemer	H+-ATPase V1 domair	orf19.9712	13718	CaZorro2a	CELL CYCLE AND DNA PROCESSING	
CA0712	1.1	1.0	1.0	IPF13717	3739000..3	unknown function	orf19.2167	13717	CaVMA5	PROTEIN I	transporter activity
CA0713	1.1	1.0	0.9	IFJ2	complemer	Unknown function	orf19.2168	13715	IPF13717	UNCLASSI	molecular_function unknown
CA0714	1.0	1.0	1.0	IPF9480	3748514..3	unknown function	orf19.3394	9480	CaIFJ2	UNCLASSI	molecular_function unknown
CA0715	1.0	1.1	1.0	DBP9	complemer	dead box helicase	orf19.3393	9477	IPF9480	UNCLASSIFIED PROTEINS	
CA0716	0.9	1.0	1.0	DOG2	complemer	2-deoxyglucose-6-pho	orf19.3392	9474	CaDBP9	CLASSIFIC	RNA binding,helicase activity
CA0717	0.9	0.9	1.0	IPF9785	3755079..3	unknown function	orf19.3453	9785	CaDOG2	Phosphate	hydrolase activity
CA0718	1.1	1.0	1.0	IPF9782	complemer	unknown function	orf19.3455	9782	IPF9785	CELL FATE PROTEIN ACTIVITY REGULATION	
CA0719	1.0	1.0	1.0	IPF9779.5	3759945..3	serine/threonine protei	orf19.3456	9779	IPF9782	TRANSCR	transporter activity
CA0720	0.9	0.9	0.9	IPF17932	complemer	unknown function	orf19.3457	17933	IPF9779.5	CELL CYCLE AND DNA PROCESSING CELL FATE	
CA0721	1.2	1.0	1.1	IPF10429	3766761..3	unknown function	orf19.1040	10429	IPF17932	UNCLASSI	DNA binding
CA0722	1.0	1.0	1.0	ERK1	complemer	mitogen-activated prot	orf19.1040	14791	IPF10429	UNCLASSI	molecular_function unknown
CA0723	0.9	0.9	0.9	PRI2	complemer	DNA-directed DNA pol	orf19.2885	14788	CaERK1	REGULATI	protein kinase activity,signal transducer activity
CA0724	1.0	0.9	0.9	IPF13398	complemer	protein kinase (by hom	orf19.7708	13398	CaPRI2	CELL CYC	nucleotidyltransferase activity
CA0725	1.2	1.7	0.9	IPF20063	3788443..3	unknown function	orf19.1749	20063	IPF13398	UNCLASSIFIED PROTEINS	
CA0727	1.1	1.0	1.0	IPF7347	complemer	unknown function	orf19.1121	7347	IPF20063	No significant S.c. match	
CA0728	1.0	1.0	1.0	IPF7345	3795607..3	unknown function	orf19.1121	7345	IPF7347	UNCLASSI	molecular_function unknown
CA0729	0.9	1.1	0.9	IPF7338	3800930..3	unknown function	orf19.1121	7338	IPF7345	UNCLASSI	molecular_function unknown
CA0730	1.0	1.1	0.9	IPF7334	3804299..3	unknown function	orf19.1120	7334	IPF7338	No significant S.c. match	
CA0731	1.0	1.3	1.0	IPF7333	complemer	unknown function	orf19.1120	7333	IPF7334	UNCLASSI	molecular_function unknown
CA0732	1.0	1.0	1.0	IPF9000	complemer	unknown function	orf19.4636	9000	IPF7333	CLASSIFIC	DNA binding,transcription regulator activity
CA0733	1.0	0.9	1.0	NIP1	3809260..3	translation initiation fac	orf19.4635	8998	IPF9000	No significant S.c. match	
CA0734	1.0	1.0	1.0	IPF8996	3812285..3	unknown function	orf19.4634	8996	CaNIP1	PROTEIN S	translation regulator activity
CA0735	1.0	1.0	1.0	IPF8995	3813827..3	unknown function	orf19.4633	8995	IPF8996	UNCLASSI	molecular_function unknown
CA0736	1.2	1.6	1.0	RPL20B	complemer	ribosomal protein (by h	orf19.4632	8994	IPF8995	CLASSIFIC	oxidoreductase activity
CA0737	0.9	1.0	1.0	SEC16.53F	3817616..3	Multidomain vesicle coat protein, i		19510	CaRPL20B	PROTEIN S	structural molecule activity
CA0738	1.0	0.9	0.9	SEC16.3F	3820133..3	Multidomain vesicle co	orf19.1181	14620	CaSEC16.5	C-compound and carbohydrate metabolism SUBCELLULAR LOCALISATION	
CA0739	1.0	1.0	1.1	IPF18885	complemer	similar to Saccharomy	orf19.1181	18885	CaSEC16.3	No significant S.c. match	
CA0740	1.1	0.9	1.1	IPF3262.3	3832587..3	unknown function, 3-prime end		3262	IPF18885	CLASSIFIC	hydrolase activity
CA0741	1.0	1.0	1.0	IPF3261	3833641..3	unknown function	orf19.3141	3261	IPF3262.3	PROTEIN I	chaperone activity
CA0742	1.0	0.9	1.0	IPF3259	complemer	unknown function	orf19.3142	3259	IPF3261	SUBCELLU	molecular_function unknown
CA0743	1.1	1.1	1.0	RNA14.3E	3839805..3	component of pre-mRNA 3 -end p		14917	IPF3259	No significant S.c. match	
CA0744	1.0	1.1	1.0	IPF14919	3841658..3	unknown function	orf19.9105	14919	CaRNA14.3	TRANSCR	RNA binding
CA0745	1.0	1.2	1.0	MTF1	complemer	RNA polymerase spec	orf19.9104	14920	IPF14919	No significant S.c. match	
CA0746	4.8	1.1	1.0	IPF14921	3845097..3	unknown function	orf19.1527	14921	CaMTF1	CELL CYC	transcription regulator activity
CA0747	0.9	1.0	1.0	SNF2	complemer	component of SWI/SN	orf19.9102	15319	IPF14921	No significant S.c. match	
CA0748	1.2	1.6	1.4	TFS1	complemer	cdc25-dependent nutri	orf19.1974	6281	CaSNF2	C-compour	transcription regulator activity
CA0749	1.1	1.0	1.1	HAP5	complemer	CCAAT-binding factor	orf19.1973	6283	CaTFS1	CELL CYC	enzyme regulator activity
CA0750	0.9	0.8	0.9	IPF6284	complemer	unknown function	orf19.1972	6284	CaHAP5	TRANSCR	transcription regulator activity
CA0751	1.0	1.0	1.0	IPF6286	3858216..3	unknown function	orf19.1971	6286	IPF6284	UNCLASSI	molecular_function unknown
CA0752	1.0	0.9	1.0	MNN22	3864361..3	Golgi alpha-1,2-mann	orf19.1128	10363	IPF6286	TRANSCRIPTION	
CA0753	1.0	1.0	0.9	IPF10359	complemer	unknown function	orf19.1128	10359	CaMNN22	CELL FATE	

CA0754	0.9	1.0	1.1	IPF15880	complemer	unknown function	orf19.3486	15880	IPF10359	UNCLASSI	molecular_function unknown
CA0755	1.0	0.8	1.1	IPF14861	3877364..3	unknown function	orf19.3487	14861	IPF15880	No significant S.c. match	
CA0756	0.9	0.9	1.0	IPF14859	3878814..3	acyl-coenzyme-A dehy	orf19.3488	14859	IPF14861	No significant S.c. match	
CA0757	0.9	1.2	0.9	GAP7.3EO	3903803..3	general amino-acid permease, 3-p		11170	IPF14859	No significant S.c. match	
CA0758	0.9	0.8	0.9	PAP11	complemer	poly(A) polymerase by	orf19.3197	19882	CaGAP7.3	Amino acid metabolism CELLULAR TRANSPORT AND TRANSPORT MECHANISMS SUBCELLULAR LOCALISATION TRANSPORT FA	
CA0759	1.3	1.3	1.1	IPF11167	3907343..3	unknown function	orf19.3198	11167	CaPAP11	TRANSCR	nucleotidyltransferase activity
CA0760	1.0	0.9	1.1	IPF11161.3	3913153..3	unknown function, 3-pr	orf19.3201	11161	IPF11167	Lipid fatty-acid and isoprenoid metabolism	
CA0761	0.9	1.1	0.9	IPF8267	complemer	P-type ATPase	orf19.9146	8267	IPF11161.3	CELL FATE	transcription regulator activity
CA0762	1.0	1.0	1.0	TAF67	3920176..3	TFIID subunit (by homo	orf19.9147	15686	IPF8267	TRANSPO	molecular_function unknown
CA0763	1.2	1.4	1.1	RPL11	complemer	60S ribosomal protein	orf19.2232	9044	CaTAF67	TRANSCR	transcription regulator activity
CA0764	1.0	0.9	1.0	PRE2	complemer	20S proteasome subur	orf19.2233	9042	CaRPL11	PROTEIN S	structural molecule activity
CA0765	1.0	0.9	1.0	IPF9040.3E	complemer	similar to Saccharomyc	orf19.9778	9040	CaPRE2	CELL CYC	peptidase activity
CA0766	0.9	0.9	0.9	IPF15335	3933411..3	unknown function	orf19.1243	15335	IPF9040.3E	TRANSCR	DNA binding,transcription regulator activity
CA0767	1.1	1.2	1.0	KEM1.3	3936597..3	multifunctional nucleas	orf19.1243	13214	IPF15335	No significant S.c. match	
CA0768	1.0	1.0	1.0	COX19	complemer	Protein required for cyt	orf19.1243	13215	CaKEM1.3	Nucleotide	DNA binding
CA0769	0.8	1.2	0.7	IPF10967	complemer	unknown function	orf19.2826	10967	CaCOX19	No significa	transporter activity
CA0770	1.0	1.0	1.0	IPF10963	3951799..3	similar to Saccharomyc	orf19.1034	10963	IPF10967	No significant S.c. match	
CA0771	1.1	1.1	1.1	IPF11977	complemer	asaprtic proteinase (by	orf19.853	11977	IPF10963	SUBCELLU	structural molecule activity
CA0772	1.0	1.0	0.9	UGA11.EX	complemer	4-aminobutyrate aminotransferase		11976	IPF11977	PROTEIN FATE [folding modification destination] ""SUBCELLULAR LOCALISATION	
CA0773	0.9	0.9	0.7	UGA11.EX	complemer	4-aminobutyrate aminc	orf19.854	11975	CaUGA11.	Amino acid metabolism Nitrogen and sulphur metabolism	
CA0774	1.0	1.0	0.9	IPF11974	complemer	unknown function	orf19.855	11974	CaUGA11.	Amino acid	transferase activity
CA0775	1.0	1.1	1.1	IFK2	complemer	probable monoxygenas	orf19.856	11973	IPF11974	No significant S.c. match	
CA0777	1.0	1.0	1.0	IPF11777	3968540..3	unknown function	orf19.4778	11777	CaIFK2	No significant S.c. match	
CA0778	1.3	1.1	1.0	IPF12884	complemer	unknown function	orf19.4779	12884	IPF11777	Amino acid metabolism TRANSCRIPTION SUBCELLULAR LOCALISATION UNCLASSIFIED PROTEINS	
CA0779	1.1	1.0	1.0	IPF20065	complemer	similar to Saccharomyc	orf19.3491	20065	IPF12884	CELL RES	molecular_function unknown
CA0780	0.9	1.0	0.9	CIRT1A	3982286..3	transposase	orf19.3492	3762	IPF20065	CELL CYC	DNA binding
CA0781	1.0	0.9	0.9	CAR1.3EO	3985861..3	arginase, 3-prime end	orf19.1141	16256	CaCirt1a	No significant S.c. match	
CA0782	0.7	0.6	1.0	IPF16253	3987286..3	unknown function	orf19.1141	16253	CaCAR1.3	Amino acid metabolism Nitrogen and sulphur metabolism SUBCELLULAR LOCALISATION	
CA0783	0.9	0.9	0.9	ACR1	3990073..3	Succinate-fumarate tra	orf19.3931	14601	IPF16253	No significant S.c. match	
CA0784	0.9	0.9	1.0	YUH1.3	3991413..3	Putative ubiquitin carbo	orf19.3930	14602	CaACR1	C-compour	transporter activity
CA0785	0.7	0.5	1.0	IPF14603	complemer	unknown function	orf19.1141	14603	CaYUH1.3	PROTEIN FATE [folding modification destination] ""SUBCELLULAR LOCALISATION	
CA0786	1.1	0.9	1.0	IPF7473	3996026..3	unknown function	orf19.2446	7473	IPF14603	UNCLASSI	molecular_function unknown
CA0787	1.2	1.2	1.2	IPF7472	complemer	unknown function	orf19.2447	7472	IPF7473	UNCLASSI	molecular_function unknown
CA0788	1.0	0.9	0.9	IPF14107	complemer	unknown function	orf19.9985	14107	IPF7472	UNCLASSI	transferase activity
CA0789	0.9	1.1	1.0	ESP1	complemer	Required for sister chro	orf19.3356	14196	IPF14107	No significant S.c. match	
CA0790	0.9	1.0	0.9	IPF14193	complemer	unknown function	orf19.3357	14193	CaESP1	CELL CYC	peptidase activity
CA0791	1.2	1.4	1.0	LSC1	complemer	succinate-CoA ligase /	orf19.3358	14191	IPF14193	UNCLASSI	molecular_function unknown
CA0792	1.0	1.0	1.1	DIT2	4015047..4	putative cytochrome F	orf19.554	20066	CaLSC1	C-compour	ligase activity
CA0793	1.0	0.9	1.0	IPF16273	4016707..4	similar to Saccharomyc	orf19.553	16273	CaDIT2	CELL RESCUE DEFENSE AND VIRULENCE ""CELL FATE CONTROL OF CELLULAR ORGANIZATION	
CA0794	1.0	1.0	1.0	IPF15639	4018478..4	unknown function	orf19.552	15639	IPF16273	CELL RES	transporter activity
CA0795	0.9	0.8	0.9	IPF15641	complemer	unknown function	orf19.8186	15641	IPF15639	TRANSPO	molecular_function unknown
CA0796	1.0	1.2	1.0	ALR1	4025800..4	divalent cation transpo	orf19.9175	13562	IPF15641	TRANSCRIPTION SUBCELLULAR LOCALISATION	
CA0797	1.0	0.9	0.9	IPF13564	4029562..4	putative arylsulfatase (	orf19.1608	13564	CaALR1	CELLULAR	transporter activity
CA0798	1.0	1.1	1.0	IPF13565	complemer	unknown function	orf19.9177	13565	IPF13564	No significant S.c. match	
CA0799	1.0	1.0	1.0	IPF14825	complemer	unknown function	orf19.9178	13567	IPF13565	UNCLASSI	molecular_function unknown
CA0800	1.0	0.9	1.0	IPF14064	4037327..4	similar to Saccharomyc	orf19.3494	14064	IPF14825	No significant S.c. match	
CA0801	1.0	1.2	1.0	CHC1	complemer	clathrin heavy chain (b	orf19.3496	14063	IPF14064	SUBCELLU	protein binding
CA0802	0.9	0.9	0.9	IPF14060	complemer	unknown function	orf19.3498	14060	CaCHC1	PROTEIN I	structural molecule activity
CA0803	1.0	0.9	1.0	IFL4	4047742..4	unknown function	orf19.3512	12574	IPF14060	UNCLASSIFIED PROTEINS	
CA0804	0.9	1.1	1.1	BNA1	4049307..4	3-hydroxyanthranilic ac	orf19.3515	12570	CaIFL4	CELL RESCUE DEFENSE AND VIRULENCE ""SUBCELLULAR LOCALISATION	
CA0805	1.0	0.9	1.1	IPF12568	complemer	unknown function	orf19.3516	12568	CaBNA1	Amino acid	oxidoreductase activity
CA0806	1.0	1.0	1.0	IPF12567	4050879..4	unknown function	orf19.3517	12567	IPF12568	No significant S.c. match	
CA0807	1.0	0.9	0.9	IPF12564	4053164..4	ADP/ATP carrier prote	orf19.3518	12564	IPF12567	UNCLASSI	molecular_function unknown
CA0808	1.0	1.0	1.1	SUA71	4054757..4	TFIIB subunit (transcrip	orf19.3519	12563	IPF12564	CELLULAR	transporter activity
CA0810	1.1	1.0	1.0	RRN3	4062688..4	RNA polymerase I spe	orf19.1923	19708	CaSUA71	TRANSCRIPTION SUBCELLULAR LOCALISATION	
CA0811	1.1	1.0	1.1	IPF17625	complemer	putative cell wall protei	orf19.1920	17625	CaRRN3	TRANSCR	transcription regulator activity
CA0812	1.0	1.2	1.1	IPF3348	complemer	unknown function	orf19.1917	16243	IPF17625	CELL RESCUE DEFENSE AND VIRULENCE ""SUBCELLULAR LOCALISATION	
CA0813	1.2	1.0	0.9	MPP10	complemer	component of the U3 s	orf19.1915	16234	IPF3348	UNCLASSIFIED PROTEINS	
CA0814	0.9	1.0	0.9	IPF16233	complemer	unknown function	orf19.1914	16233	CaMPP10	TRANSCR	molecular_function unknown
CA0815	0.9	0.9	0.9	IPF16231	4069461..4	unknown function	orf19.1913	16231	IPF16233	No significant S.c. match	
CA0817	0.9	1.0	0.7	IPF6339	4087533..4	unknown function	orf19.1023	6399	IPF16231	No significant S.c. match	
CA0819	1.0	1.0	1.0	VPS1	complemer	member of the dynami	orf19.1949	19610	IPF6339	No significant S.c. match	
CA0820	1.0	1.0	1.0	IPF12138	complemer	unknown function	orf19.1950	18864	CaVPS1	PROTEIN I	hydrolase activity
CA0821	1.1	1.1	1.1	IPF16795	4104298..4	glycerate/formate-dehy	orf19.2989	16795	IPF12138	UNCLASSI	hydrolase activity
CA0822	1.1	0.9	1.0	EXG1	complemer	glucan 1,3-beta-glucos	orf19.2990	14073	IPF16795	C-compour	oxidoreductase activity
CA0823	1.0	1.0	0.9	IPF11987.3	4112204..4	unknown function, 3-pr	orf19.692	11987	CaEXG1	C-compour	hydrolase activity
CA0824	1.1	1.3	1.0	GPD2	4114960..4	Glycerol 3-phosphate c	orf19.691	11982	IPF11987.3	No significant S.c. match	
CA0825	1.0	1.0	0.9	PLB2	complemer	phospholipase B	orf19.8309	11981	CaGPD2	C-compound and carbohydrate metabolism ""CELL RESCUE DEFENSE AND VIRULENCE ""REGULATION OF/INTERACTION WITH CEI	
CA0826	0.8	0.9	0.9	IPF19885	4118749..4	unknown function	orf19.8308	19885	CaPLB2	Lipid fatty-acid and isoprenoid metabolism ""SUBCELLULAR LOCALISATION Other virulence attributes	
CA0827	1.0	0.9	0.9	IPF18859	4122110..4	unknown function	orf19.7884	18859	IPF19885	No significant S.c. match	
CA0828	2.9	2.9	2.2	IPF17186	4124295..4	unknown function	orf19.7882	17186	IPF18859	UNCLASSI	molecular_function unknown
CA0829	0.9	0.7	1.1	SLC1	4125941..4	fatty acyltransferase (b	orf19.250	19886	IPF17186	UNCLASSI	molecular_function unknown

CA0830	1.2	1.0	1.1	APL5	complemer	AP-3 complex subunit,	orf19.7879	14699	CaSLC1	Lipid fatty-a	transferase activity
CA0831	1.0	0.8	1.1	IPF10493.5	4130615..4	unknown function, 5-pr	orf19.7878	14701	CaAPL5	CELLULAF	molecular_function unknown
CA0832	0.9	1.0	1.0	IPF14225	complemer	unknown function	orf19.9244	14225	IPF10493.5	No significant S.c. match	
CA0833	0.9	0.9	1.0	IPF12501	4136657..4	unknown function	orf19.9245	12501	IPF14225	UNCLASSI	molecular_function unknown
CA0834	1.0	1.0	1.1	IPF12498.3	complemer	unknown function, 3-pr	orf19.1677	12499	IPF12501	UNCLASSI	molecular_function unknown
CA0835	1.1	1.1	1.2	IPF12498.5	complemer	unknown function, internal fragmer		12498	IPF12498.3	No significant S.c. match	
CA0836	1.2	1.0	1.1	IPF12495.5	4140843..4	unknown function, 5-pr	orf19.9248	12495	IPF12498.5	No significant S.c. match	
CA0837	0.7	0.6	0.9	IPF12495.3	4141422..4	unknown function, 3-prime end		12494	IPF12495.5	No significant S.c. match	
CA0838	1.1	1.0	1.0	TFP1	complemer	vacuolar ATPase subu	orf19.9249	12493	IPF12495.3	No significant S.c. match	
CA0839	0.9	1.0	0.9	IPF12492	complemer	unknown function		12492	CaTFP1	PROTEIN I	DNA binding
CA0840	2.2	2.2	1.8	IFD1	4146777..4	Putative aryl-alcohol de	orf19.8650	18856	IPF12492	PROTEIN FATE [folding modification destination] ""SUBCELLULAR LOCALISATION	
CA0841	1.0	1.1	1.0	IPF15638	complemer	unknown function	orf19.8652	15638	CaIFD1	C-compound and carbohydrate metabolism ENERGY	
CA0842	0.9	1.0	1.0	IPF15633	complemer	similar to Saccharomy	orf19.1053	15633	IPF15638	No significant S.c. match	
CA0843	1.0	1.1	1.0	IPF18853	complemer	unknown function	orf19.8656	18853	IPF15633	CELLULAF	molecular_function unknown
CA0844	1.1	1.1	1.1	CDC3	complemer	Cell division control pr	orf19.8657	16098	IPF18853	UNCLASSI	molecular_function unknown
CA0845	1.1	1.1	1.1	IPF16314	complemer	unknown function	orf19.7896	16314	CaCDC3	CELL CYC	structural molecule activity
CA0846	0.9	1.0	1.0	SMC3	complemer	required for structural r	orf19.7895	12682	IPF16314	No significant S.c. match	
CA0848	0.2	0.3	0.1	ACS1	4172674..4	acetyl-coenzyme-A syr	orf19.1743	15005	CaSMC3	CELL CYC	hydrolase activity
CA0849	1.0	1.0	1.0	HEM4	complemer	uroporphyrinogen III sy	orf19.9311	15006	CaACS1	C-compour	ligase activity
CA0850	1.1	1.1	1.0	IPF16671	4177044..4	unknown function	orf19.9312	16671	CaHEM4	Metabolism	lyase activity
CA0851	1.0	0.9	1.0	IPF16670	4177799..4	unknown function	orf19.9313	16670	IPF16671	No significant S.c. match	
CA0852	1.0	1.0	1.0	KIP2	4179505..4	Kinesin-related protein	orf19.9315	10752	IPF16670	UNCLASSI	molecular_function unknown
CA0853	0.8	1.0	0.8	IPF19640	4182073..4	unknown function	orf19.9316	19639	CaKIP2	CELL CYC	motor activity
CA0854	1.1	1.2	1.0	IPF8854	complemer	similar to Saccharomy	orf19.4900	8854	IPF19640	No significant S.c. match	
CA0855	1.0	0.9	1.0	AMYG1	complemer	glucoamylase	orf19.4899	8948	IPF8854	C-compour	transferase activity
CA0856	1.0	1.0	1.1	IFL1	4203810..4	unknown function	orf19.1194	14489	CaAMYG1	C-compound and carbohydrate metabolism ENERGY SUBCELLULAR LOCALISATION	
CA0857	1.0	1.0	0.9	SEN15	4205571..4	tRNA splicing endonuc	orf19.4464	14490	CaIFL1	CELL RESCUE DEFENSE AND VIRULENCE ""CELL FATE	
CA0858	0.9	0.9	0.9	IPF14493	complemer	unknown function	orf19.4465	14493	CaSEN15	No significant S.c. match	
CA0859	0.9	1.0	1.0	IPF14495.3	complemer	similar to Saccharomy	orf19.1194	14495	IPF14493	No significant S.c. match	
CA0860	1.1	1.0	1.1	IPF17272	4213479..4	unknown function	orf19.3522	17272	IPF14495.3	PROTEIN I	transferase activity
CA0861	1.1	1.0	1.0	CRK1.3F	complemer	Protein kinase, 3-prime	orf19.3523	17271	IPF17272	No significant S.c. match	
CA0862	1.0	1.0	1.0	CRK1.5F	complemer	Protein kinase, 5-prime	orf19.3524	17267	CaCRK1.3	No significant S.c. match	
CA0863	1.1	1.0	1.0	ITR2	complemer	Myo-inositol transporte	orf19.3526	4456	CaCRK1.5	CELL CYC	protein kinase activity
CA0864	0.8	0.9	0.8	CYT1	complemer	cytochrome-c1 (by hor	orf19.3527	4458	CaITR2	C-compour	transporter activity
CA0865	1.0	1.0	1.0	IFM2	4224960..4	Glycerate-formate-deh	orf19.3584	14777	CaCYT1	ENERGY S	transporter activity
CA0866	1.0	1.0	1.1	IPF14775	complemer	unknown function	orf19.3585	14775	CaIFM2	C-compound and carbohydrate metabolism	
CA0867	1.0	0.6	1.0	IPF14773.8	complemer	unknown function, exo	orf19.3586	14774	IPF14775	UNCLASSI	molecular_function unknown
CA0869	1.1	1.1	1.0	IPF14772	4228888..4	unknown function	orf19.3589	14772	IPF14773.8	No significant S.c. match	
CA0870	1.2	1.7	1.2	IPP1	4230096..4	inorganic pyrophospha	orf19.3590	19604	IPF14772	CELL CYC	DNA binding
CA0871	0.9	0.9	1.1	APE3	4231613..4	aminopeptidase (by hc	orf19.3591	16625	CaIPP1	Phosphate	hydrolase activity
CA0872	1.0	1.1	1.1	IPF13100	complemer	unknown function	orf19.3592	13100	CaAPE3	PROTEIN I	peptidase activity
CA0873	0.9	1.0	0.9	BUL1	4237825..4	Ubiquitin ligase binding	orf19.1209	9241	IPF13100	PROTEIN I	chaperone activity
CA0874	1.1	1.2	1.1	CPA1	4241368..4	Arginine-specific carba	orf19.1210	11885	CaBUL1	PROTEIN I	protein binding
CA0875	1.0	1.1	1.1	ERG251	4244711..4	C-4 sterol methyl oxida	orf19.1210	8993	CaCPA1	Amino acid	ligase activity
CA0876	1.1	1.3	1.0	VMA6	4247506..4	H+-ATPase V0 domair	orf19.7996	19887	CaERG251	Lipid fatty-acid and isoprenoid metabolism ""SUBCELLULAR LOCALISATION	
CA0877	1.1	1.1	0.9	CNH1.5F	4249699..4	Na+/H+ antiporter, 5-p	orf19.8000	10959	CaVMA6	PROTEIN I	transporter activity
CA0878	1.0	1.0	0.9	CNH1.3F	4251525..4	Na+/H+ antiporter, 3-p	orf19.8001	18845	CaCNH1.5	REGULATI	transporter activity
CA0879	0.7	0.8	0.4	IPF19542.5	4252167..4	unknown function, 5-pr	orf19.8003	19542	CaCNH1.3	REGULATION OF/INTERACTION WITH CELLULAR ENVIRONMENT TRANSPORT FACILITATION	
CA0880	1.0	1.0	1.0	IPF19542.3	4253029..4	unknown function, 3-pr	orf19.372	16966	IPF19542.5	Amino acid metabolism TRANSCRIPTION SUBCELLULAR LOCALISATION	
CA0881	1.0	1.1	1.0	IPF16965	complemer	unknown function	orf19.8006	16965	IPF19542.3	UNCLASSI	molecular_function unknown
CA0882	1.0	1.1	1.0	PHR3	4257794..4	surface glycoprotein (b	orf19.8010	12107	IPF16965	CLASSIFIC	molecular_function unknown
CA0883	2.0	1.5	2.2	SUN41	4266853..4	Putative cell wall beta-	orf19.3642	14577	CaPHR3	CLASSIFIC	molecular_function unknown
CA0884	1.1	1.2	1.1	CAN5	complemer	amino acid permease (	orf19.3641	19550	CaSUN41	CELL CYC	molecular_function unknown
CA0885	1.0	0.9	1.2	IPF12381	4273724..4	unknown function	orf19.1389	12381	CaCAN5	Amino acid metabolism CELLULAR TRANSPORT AND TRANSPORT MECHANISMS SUBCELLULAR LOCALISATION TRANSPORT FA	
CA0886	0.9	0.9	1.0	IPF12382	4276766..4	unknown function	orf19.1388	12382	IPF12381	TRANSCR	RNA binding
CA0887	1.0	1.0	1.1	IPF12383	complemer	unknown function	orf19.1387	12383	IPF12382	SUBCELLU	molecular_function unknown
CA0888	0.9	0.9	1.0	BET1	4278785..4	involved in ER-Golgi tr	orf19.1386	12385	IPF12383	UNCLASSI	molecular_function unknown
CA0889	1.0	1.0	1.0	IPF11767	4284439..4	mitochondrial carrier p	orf19.4733	11767	CaBET1	CELLULAF	transporter activity
CA0890	0.8	0.8	1.0	IPF11766	4285793..4	unknown function	orf19.4734	11766	IPF11767	CELLULAR TRANSPORT AND TRANSPORT MECHANISMS SUBCELLULAR LOCALISATION TRANSPORT FACILITATION	
CA0891	0.9	1.0	1.0	IPF11764	4287107..4	unknown function	orf19.4735	11764	IPF11766	PROTEIN FATE [folding modification destination]	
CA0892	1.1	1.2	1.0	PHO8.5	4288986..4	repressible alkaline ph	orf19.4736	19888	IPF11764	UNCLASSI	molecular_function unknown
CA0893	1.0	1.1	1.1	IPF13517	4294111..4	unknown function	orf19.5752	13517	CaPHO8.5	Phosphate	hydrolase activity
CA0894	1.0	0.9	0.9	ORM1	4296932..4	unknown function	orf19.5751	13514	IPF13517	UNCLASSI	molecular_function unknown
CA0895	1.1	1.3	1.0	SHM2	4299044..4	Serine hydroxymethylt	orf19.5750	15120	CaORM1	UNCLASSI	molecular_function unknown
CA0896	1.0	1.3	0.9	SBA1	4300930..4	Hsp90 (Ninety) Associ	orf19.5749	15122	CaSHM2	Nucleotide	transferase activity
CA0897	1.0	0.9	1.0	IPF4902	4304092..4	unknown function	orf19.8043	4902	CaSBA1	PROTEIN I	chaperone activity
CA0898	1.1	1.2	1.1	SSH1.3	complemer	involved in co-translati	orf19.412	4903	IPF4902	No significant S.c. match	
CA0899	1.0	1.1	0.9	IPF4905	complemer	unknown function	orf19.411	4905	CaSSH1.3	PROTEIN I	transporter activity
CA0900	1.1	1.0	1.0	IPF12031	4316540..4	unknown function	orf19.3411	12031	IPF4905	No significant S.c. match	
CA0901	0.9	0.9	0.9	IPF12033	4317758..4	unknown function	orf19.3412	12033	IPF12031	Metabolism	molecular_function unknown
CA0902	0.9	0.9	1.0	IPF12034	complemer	unknown function	orf19.3413	12034	IPF12033	UNCLASSI	hydrolase activity
CA0903	0.8	0.9	0.8	IPF4401	4323951..4	putative succinate deh	orf19.1480	4401	IPF12034	No significant S.c. match	

CA0904	0.9	1.0	0.9	IPF4403	complemer	unknown function	orf19.1481	4403	IPF4401	ENERGY	molecular_function unknown
CA0905	0.9	0.9	0.9	IPF4405	4328219..4	unknown function	orf19.1482	4405	IPF4403	No significant S.c. match	
CA0906	1.0	1.0	1.1	IPF4406	complemer	unknown function	orf19.1483	4406	IPF4405	No significant S.c. match	
CA0907	1.0	0.9	1.0	IPF19889	4329345..4	Unknown Function	orf19.1484	19889	IPF4406	UNCLASSI	molecular_function unknown
CA0908	0.9	0.9	1.0	MRPL31	complemer	Mitochondrial riboso	orf19.1485	6016	IPF19889	No significant S.c. match	
CA0910	1.0	1.1	1.0	MRPL16	4342281..4	ribosomal protein	orf19.9569	4102	CaMRPL3	PROTEIN	structural molecule activity
CA0911	0.9	1.0	1.0	IPF14814	complemer	unknown function	orf19.9568	14814	CaMRPL1	PROTEIN	structural molecule activity
CA0912	0.9	1.1	1.0	RPA49	4344552..4	DNA-directed RNA pol	orf19.9567	14813	IPF14814	PROTEIN I	molecular_function unknown
CA0913	1.0	1.0	1.0	IPF14810	complemer	unknown function	orf19.9566	14810	CaRPA49	TRANSCR	nucleotidyltransferase activity
CA0914	1.1	1.5	1.1	SRA1	complemer	cAMP dependent prote	orf19.9565	19590	IPF14810	Lipid fatty-a	molecular_function unknown
CA0915	1.4	1.2	1.2	KAR2	4350772..4	dnaK-type molecular c	orf19.9564	15827	CaSRA1	C-compour	enzyme regulator activity
CA0916	0.9	1.0	0.9	LYS2	complemer	L-aminoadipate-semial	orf19.2970	19710	CaKAR2	CELL CYC	chaperone activity
CA0917	0.9	1.0	1.1	RAD16	complemer	nucleotide excision rep	orf19.2969	15502	CaLYS2	Amino acid	oxidoreductase activity
CA0918	1.0	0.9	1.1	IPF17881	complemer	cyclin (by homology)	orf19.1041	17881	CaRAD16	CELL CYC	hydrolase activity
CA0919	0.8	1.1	0.7	POR1	complemer	mitochondrial outer me	orf19.1042	12994	IPF17881	TRANSCR	protein kinase activity,enzyme regulator activity
CA0920	1.0	1.0	1.0	IPF18833	4367748..4	unknown function		18833	CaPOR1	CELLULAF	transporter activity
CA0921	0.9	1.0	1.0	IPF12992	4368845..4	unknown function	orf19.1043	12992	IPF18833	No significant S.c. match	
CA0922	1.0	1.1	1.0	IPF12991	complemer	unknown function	orf19.1045	12991	IPF12992	UNCLASSI	molecular_function unknown
CA0923	0.8	1.0	0.9	IPF12987	4370933..4	unknown function	orf19.1047	12987	IPF12991	TRANSCR	RNA binding
CA0924	2.7	2.2	2.8	IFD5	4374190..4	Putative aryl-alcohol d	orf19.1048	17049	IPF12987	UNCLASSI	molecular_function unknown
CA0925	0.9	0.8	1.0	IPF17050	complemer	unknown function	orf19.1049	17050	CaIFD5	C-compound and carbohydrate metabolism ENERGY	
CA0926	1.0	0.9	1.0	IPF11335	4381652..4	Member of the phosph	orf19.3663	11335	IPF17050	ENERGY	
CA0927	1.0	1.1	1.1	IPF11332	4385006..4	unknown function	orf19.3661	11332	IPF11335	Phosphate	transporter activity
CA0928	1.0	0.9	0.9	IPF19891	4388461..4	unknown function	orf19.8192	19891	IPF11332	PROTEIN I	peptidase activity
CA0929	0.8	1.0	0.9	GUT1	complemer	Glycerol kinase (by ho	orf19.558	3751	IPF19891	No significant S.c. match	
CA0930	1.0	1.0	1.0	ZORRO1A	complemer	Putative reverse transc	orf19.559	3750	CaGUT1	C-compour	transferase activity
CA0931	0.9	1.0	1.1	IPF3748	complemer	unknown function	orf19.562	3748	CaZorro1a	CELL CYCLE AND DNA PROCESSING CELLULAR TRANSPORT AND TRANSPORT MECHANISMS SUBCELLULAR LOCALISATION	
CA0932	1.1	1.0	1.0	EPT1	complemer	alcohol phosphatidyl tr	orf19.3695	8065	IPF3748	CELL CYCLE AND DNA PROCESSING CONTROL OF CELLULAR ORGANIZATION SUBCELLULAR LOCALISATION	
CA0933	1.0	1.0	1.0	ATM1	complemer	ATP-binding cassette t	orf19.8678	14199	CaEPT1	Lipid fatty-a	transferase activity
CA0934	1.0	0.9	1.0	IPF14203.3	complemer	similarity to several tra	orf19.1078	14202	CaATM1	CELLULAF	transporter activity,hydrolase activity
CA0935	0.9	1.0	1.1	IPF14203.5	complemer	similarity to several tra	orf19.1079	14203	IPF14203.3	Amino acid metabolism Nitrogen and sulphur metabolism	
CA0936	1.0	1.1	1.1	IPF16291	4422849..4	unknown function	orf19.1080	16291	IPF14203.5	Amino acid	transferase activity
CA0937	1.1	0.9	1.0	IPF19892	4425035..4	unknown function	orf19.8683	19892	IPF16291	No significant S.c. match	
CA0938	3.2	2.7	1.5	COX12	4425874..4	cytochrome-c oxidase, subunit VIE		16288	IPF19892	No significant S.c. match	
CA0939	0.9	1.0	1.1	DBP6	complemer	RNA helicase required	orf19.3704	8614	CaCOX12	ENERGY "	oxidoreductase activity
CA0940	1.0	0.8	1.0	IPF8616	4429583..4	similar to Saccharomyces cerevisi		8616	CaDBP6	TRANSCR	RNA binding,helicase activity
CA0941	0.9	0.8	1.0	IPF8617	complemer	unknown function	orf19.3705	8617	IPF8616	PROTEIN	structural molecule activity
CA0942	0.9	1.1	1.1	IPF8619	4432166..4	unknown function	orf19.3706	8619	IPF8617	UNCLASSI	protein phosphatase activity
CA0943	0.9	0.9	0.8	YHB1	complemer	flavohemoglobin (by h	orf19.3707	8621	IPF8619	PROTEIN FATE [folding modification destination]	
CA0944	1.0	1.1	1.1	IPF10280	complemer	unknown function	orf19.8852	10280	CaYHB1	CELL RES	molecular_function unknown
CA0945	1.1	1.2	0.9	IPF10278	4442823..4	DNA-J - like protein (b	orf19.8853	10278	IPF10280	No significant S.c. match	
CA0946	1.0	1.1	1.1	IPF10277	4444560..4	unknown function		10277	IPF10278	PROTEIN FATE [folding modification destination]	
CA0947	1.0	1.0	1.0	CFL3	4445719..4	ferric reductase-like pr	orf19.1270	10273	IPF10277	UNCLASSI	molecular_function unknown
CA0948	0.9	0.9	1.0	IPF10270	complemer	unknown function	orf19.1272	10270	CaCFL3	REGULATION OF/INTERACTION WITH CELLULAR ENVIRONMENT Other virulence attributes	
CA0949	1.0	1.0	0.9	IPF10269	4450250..4	Gim complex compone	orf19.8859	10269	IPF10270	UNCLASSI	molecular_function unknown
CA0950	1.0	1.0	1.0	IPF3737	complemer	similar to Saccharomy	orf19.1012	3737	IPF10269	CELL CYC	protein binding
CA0951	1.1	1.0	0.9	MNN6	4458347..4	putative golgi alpha-1,	orf19.1011	3740	IPF3737	PROTEIN I	protein binding
CA0952	1.0	1.1	1.0	MNT3.3EO	complemer	Putative mannosyltransferase, 3-p		19564	CaMNN6	CELL FATI	transferase activity
CA0953	1.2	1.4	1.1	IPF13166.3	4462120..4	unknown function, 3-pr	orf19.1144	13166	CaMNT3.3	C-compound and carbohydrate metabolism ""PROTEIN FATE [folding modification destination] ""SUBCELLULAR LOCALISATION	
CA0954	1.0	0.9	1.0	IPF13162	4468223..4	unknown function	orf19.1142	13162	IPF13166.3	C-compound and carbohydrate metabolism	
CA0955	1.0	1.0	1.0	IPF13160	complemer	unknown function	orf19.1140	13160	IPF13162	UNCLASSI	transporter activity
CA0956	1.0	1.1	1.0	IPF14914	4484183..4	putative ankyrin (by ho	orf19.1219	14914	IPF13160	UNCLASSIFIED PROTEINS	
CA0957	0.9	1.0	1.0	IPF14322	4489509..4	unknown function		14322	IPF14914	UNCLASSI	hydrolase activity
CA0958	1.0	1.0	1.1	SEC24	complemer	component of COPII c	orf19.1219	14321	IPF14322	UNCLASSI	molecular_function unknown
CA0959	1.6	1.3	1.4	SAM2	complemer	S-adenosylmethionine	orf19.657	15535	CaSEC24	CELLULAF	protein binding
CA0960	1.1	1.0	1.0	DPP1	complemer	Diacylglycerol Pyropho	orf19.8271	5432	CaSAM2	Amino acid	transferase activity
CA0961	0.9	1.1	1.0	IPF18822	4509850..4	unknown function	orf19.3720	18822	CaDPP1	Lipid fatty-acid and isoprenoid metabolism ""CELL CYCLE AND DNA PROCESSING CELL FATE	
CA0962	1.1	1.1	1.0	IPF9145	complemer	unknown function	orf19.6245	9145	IPF18822	C-compound and carbohydrate metabolism CELL CYCLE AND DNA PROCESSING CELLULAR TRANSPORT AND TRANSPORT MECH	
CA0963	1.0	1.0	1.0	IPF9143	complemer	similar to Saccharomy	orf19.6246	9143	IPF9145	No significant S.c. match	
CA0964	1.1	1.0	0.9	IPF9141	complemer	similar to Saccharomy	orf19.6247	9141	IPF9143	PROTEIN I	transferase activity
CA0965	1.0	1.0	1.1	IPF9139	4524296..4	unknown function	orf19.6248	9139	IPF9141	CELL CYC	DNA binding
CA0966	1.0	1.1	1.0	IPF9136.5E	4526750..4	potassium transporter,	orf19.6249	9136	IPF9139	No significant S.c. match	
CA0968	0.9	1.0	1.0	SAP6	4534813..4	secreted aspartyl prote	orf19.1298	12747	IPF9136.5	CELL RESCUE DEFENSE AND VIRULENCE ""TRANSPORT FACILITATION	
CA0969	1.0	1.0	1.0	IPF9132	4538352..4	unknown function	orf19.1298	9132	CaSAP6	PROTEIN FATE [folding modification destination] ""CELL FATE SUBCELLULAR LOCALISATION Other virulence attributes	
CA0970	0.9	0.9	0.9	IPF10894	complemer	unknown function	orf19.1034	10894	IPF9132	UNCLASSI	molecular_function unknown
CA0971	1.1	1.1	0.9	IPF11428	complemer	unknown function	orf19.6470	11428	IPF10894	CELLULAF	protein binding
CA0972	1.5	1.7	1.1	CYP1	4559821..4	cyclophilin (peptidylpr	orf19.6472	17713	IPF11428	CELL RESCUE DEFENSE AND VIRULENCE ""REGULATION OF/INTERACTION WITH CELLULAR ENVIRONMENT SUBCELLULAR L	
CA0973	1.0	1.0	1.0	IPF12662	complemer	unknown function	orf19.6474	12662	CaCYP1	PROTEIN I	isomerase activity
CA0975	1.0	1.1	1.0	IPF18811	complemer	unknown function		18811	IPF12662	No significant S.c. match	
CA0976	0.7	0.6	1.0	PEX17	4567179..4	Peroxisomal periphera	orf19.1108	17165	IPF18811	No significant S.c. match	
CA0977	1.0	0.9	0.9	IPF18810	4568339..4	unknown function	orf19.1108	18810	CaPEX17	No significant S.c. match	
CA0978	1.0	1.0	1.0	IPF16222	4570643..4	unknown function	orf19.3603	16222	IPF18810	UNCLASSI	molecular_function unknown

CA0979	0.7	0.5	0.9	IPF19713	4573238..4	unknown function	orf19.3601	19713	IPF16222	UNCLASSI	molecular_function unknown
CA0980	1.0	0.9	0.9	IPF17655	4575038..4	unknown function	orf19.3600	17655	IPF19713	CELL CYCLE AND DNA PROCESSING CONTROL OF CELLULAR ORGANIZATION	
CA0981	1.0	0.9	0.9	GAP4	complemen	general amino acid per	orf19.1799	19644	IPF17655	No significant S.c. match	
CA0982	1.0	1.0	1.0	IPF7397	complemen	unknown function	orf19.1800	7397	CaGAP4	Amino acid metabolism	CELLULAR TRANSPORT AND TRANSPORT MECHANISMS SUBCELLULAR LOCALISATION TRANSPORT FA
CA0983	1.0	1.1	1.0	CBR1	complemen	Cytochrome-b5 reducta	orf19.1801	7398	IPF7397	UNCLASSI	molecular_function unknown
CA0984	1.0	1.0	0.9	IPF7400	complemen	unknown function	orf19.1802	7400	CaCBR1	ENERGY S	transporter activity
CA0985	1.0	1.0	1.1	IPF4815	4598955..4	unknown Function	orf19.3351	4815	IPF7400	UNCLASSI	molecular_function unknown
CA0986	1.1	1.1	1.0	IPF4814	complemen	similar to Saccharomyc	orf19.3350	4814	IPF4815	No significant S.c. match	
CA0987	1.1	1.1	1.1	RPB140	4601325..4	DNA-dependent RNA p	orf19.3349	4811	IPF4814	PROTEIN S	structural molecule activity
CA0988	1.1	1.2	1.3	PMI40	complemen	mannose-6-phosphate	orf19.8968	19894	CaRPB140	TRANSCR	nucleotidyltransferase activity
CA0989	1.0	1.0	1.0	IPF11069	4610365..4	unknown function	orf19.1391	11069	CaPMI40	C-compour	isomerase activity
CA0990	1.0	0.8	1.1	IPF11068	complemen	unknown function	orf19.1392	11068	IPF11069	No significant S.c. match	
CA0991	0.8	0.7	1.0	IPF11065	complemen	unknown function	orf19.8971	11065	IPF11068	PROTEIN I	oxidoreductase activity,isomerase activity
CA0992	1.0	0.9	1.0	IPF11063	4615600..4	unknown function	orf19.8972	11063	IPF11065	Metabolism	transporter activity
CA0993	1.1	1.1	1.0	IPF11059	4618933..4	unknown function	orf19.8973	11059	IPF11063	UNCLASSI	molecular_function unknown
CA0994	1.0	1.0	1.1	IPF10077	complemen	3-oxoacid CoA-transfe	orf19.2281	10077	IPF11059	No significant S.c. match	
CA0995	0.9	1.0	1.0	IPF10074	complemen	unknown function	orf19.9822	10074	IPF10077	No significant S.c. match	
CA0996	1.0	1.0	1.0	IPF10071	complemen	catabolic 3-dehydroqui	orf19.9823	10071	IPF10074	No significant S.c. match	
CA0997	1.0	1.0	0.9	IPF9998	4626016..4	unknown function	orf19.9824	9998	IPF10071	No significant S.c. match	
CA0998	0.9	0.9	1.0	IPF12369	4630065..4	Putative dipeptidase (b	orf19.1184	12369	IPF9998	No significant S.c. match	
CA0999	1.0	1.1	1.1	IPF12368	complemen	unknown function	orf19.1184	12368	IPF12369	UNCLASSIFIED PROTEINS	
CA1000	0.4	0.6	0.4	IPF6011	complemen	unknown function	orf19.9061	6011	IPF12368	TRANSCRIPTION	
CA1001	1.0	1.0	1.1	IPF6006	complemen	unknown function	orf19.9065	6006	IPF6011	No significant S.c. match	
CA1002	0.8	1.0	0.8	ROK1.3	4651226..4	RNA helicase, 3-prime	orf19.3756	8903	IPF6006	No significant S.c. match	
CA1003	0.9	1.0	1.0	ATP20	complemen	F1F0-ATPase complex	orf19.3757	8902	CaROK1.3	TRANSCR	RNA binding,helicase activity
CA1004	1.0	1.0	0.9	IPF8901	complemen	unknown function	orf19.3758	8901	CaATP20	ENERGY S	structural molecule activity
CA1005	0.9	0.8	0.9	LPG7	4656011..4	probable membrane pr	orf19.3759	8898	IPF8901	UNCLASSI	molecular_function unknown
CA1007	1.0	1.0	1.0	DLH1.3F	4657823..4	meiotic recombination	orf19.3760	8895	CaLPG7	TRANSCR	transcription regulator activity
CA1008	1.0	1.0	1.0	IPF14083	complemen	similarity to Saccharon	orf19.6254	14083	CaDLH1.3f	CELL CYC	DNA binding
CA1009	0.9	0.9	1.0	IPF14084	complemen	unknown function	orf19.6255	14084	IPF14083	UNCLASSI	transporter activity
CA1010	0.8	0.8	0.7	GLT1.3EO	complemen	glutamate synthase (N	orf19.6257	19715	IPF14084	UNCLASSI	molecular_function unknown
CA1011	1.1	1.1	1.1	SRP101	4673540..4	signal recognition parti	orf19.1143	19895	CaGLT1.3e	Amino acid	oxidoreductase activity
CA1012	1.1	1.0	1.1	PSD2.5F	4675908..4	phosphatidylserine dec	orf19.1143	14208	CaSRP101	PROTEIN FATE [folding modification destination] ""SUBCELLULAR LOCALISATION	
CA1014	0.9	1.1	1.0	MES1	complemen	methionyl-tRNA synthe	orf19.1143	15550	CaPSD2.5f	Lipid fatty-a	lyase activity
CA1015	0.9	0.9	1.0	IPF15547	4682821..4	putative glutamyl-tRNA	orf19.1143	15547	CaMES1	PROTEIN S	ligase activity
CA1016	1.0	1.0	0.9	IPF11142	complemen	unknown function	orf19.473	11142	IPF15547	Nitrogen ar	hydrolase activity
CA1017	1.0	1.0	1.1	IPF11144	complemen	unknown function	orf19.8105	11144	IPF11142	CELL RES	transporter activity
CA1018	0.9	0.9	0.9	IPF13479.3	4696683..4	unknown function, 3-pr	orf19.3768	13479	IPF11144	UNCLASSI	molecular_function unknown
CA1019	1.0	1.1	1.2	IPF13485	4700804..4	unknown function	orf19.3769	13485	IPF13479.3	C-compound and carbohydrate metabolism TRANSCRIPTION SUBCELLULAR LOCALISATION	
CA1020	1.3	1.0	1.1	IPF4824	complemen	unknown function	orf19.1086	4824	IPF13485	TRANSPO	molecular_function unknown
CA1022	0.9	1.2	0.6	IPF4820	complemen	putativecomplex I inter	orf19.1086	4820	IPF4824	UNCLASSI	molecular_function unknown
CA1023	1.0	1.0	1.1	IPF4817	4722193..4	unknown Function	orf19.1086	4817	IPF4820	No significant S.c. match	
CA1024	0.9	0.9	0.9	CAR1	complemen	arginase by homology	orf19.3934	10187	IPF4817	CLASSIFICATION NOT YET CLEAR-CUT	
CA1025	0.5	0.3	0.8	IPF10184	4728318..4	unknown function	orf19.3936	10184	CaCAR1	Amino acid	hydrolase activity
CA1026	1.1	0.9	1.0	IPF19645.E	4732034..4	unknown function, exo	orf19.3937	19646	IPF10184	UNCLASSI	molecular_function unknown
CA1027	0.8	0.9	0.8	IPF19645.E	4732648..4	unknown function, exon 2		19645	IPF19645.e	UNCLASSIFIED PROTEINS	
CA1028	1.0	0.9	0.9	IPF6488	4743360..4	unknown function	orf19.1539	6488	IPF19645.e	UNCLASSI	molecular_function unknown
CA1029	0.1	0.6	0.2	TLG2	4746077..4	Syntaxin family of t-SN	orf19.9112	14397	IPF6488	No significant S.c. match	
CA1030	1.1	1.4	1.2	ZRC1	complemen	Zinc and cadmium resi	orf19.9111	14395	CaTLG2	PROTEIN I	transporter activity
CA1031	0.9	1.0	1.0	IPF17469	4752283..4	unknown function	orf19.3848	17469	CaZRC1	CELLULAF	transporter activity
CA1032	1.0	0.9	1.1	IPF19896	complemen	unknown function	orf19.3852	19896	IPF17469	No significant S.c. match	
CA1033	1.0	0.9	1.0	MAD2	complemen	spindle checkpoint con	orf19.8642	17891	IPF19896	No significant S.c. match	
CA1034	1.0	1.0	1.0	BET4.EXO	4776433..4	alpha subunit of geran	orf19.8641	17890	CaMAD2	CELL CYC	molecular_function unknown
CA1035	1.0	0.9	1.0	IFI2.3F	complemen	unknown function, 3-pr	orf19.1038	13751	CaBET4.ex	Lipid fatty-a	transferase activity
CA1037	1.0	1.0	1.1	MNS1	complemen	Alpha1,2-mannosidase	orf19.8638	6163	CaIFI2.3f	No significant S.c. match	
CA1038	0.9	0.7	1.0	IPF6159	4784798..4	unknown function	orf19.8637	6159	CaMNS1	C-compour	hydrolase activity
CA1039	1.0	1.0	1.0	IPF4012	4791807..4	Unknown Function	orf19.2501	4012	IPF6159	TRANSCR	DNA binding,transcription regulator activity
CA1040	0.9	1.0	0.9	IPF4010	complemen	unknown function	orf19.2503	4010	IPF4012	UNCLASSI	molecular_function unknown
CA1041	1.0	0.9	0.8	BMS1	4795951..4	probable membrane pr	orf19.2504	4009	IPF4010	No significant S.c. match	
CA1042	1.1	1.2	1.0	IPF18784	4802829..4	unknown function	orf19.2506	18784	CaBMS1	TRANSCRIPTION	
CA1043	0.9	0.9	0.9	TOM6	complemen	mitochondrial outer me	orf19.9219	15801	IPF18784	No significant S.c. match	
CA1044	1.1	1.1	1.1	RNA1	4804962..4	GTPase activating prot	orf19.9218	11621	CaTOM6	PROTEIN I	transporter activity
CA1045	0.9	0.9	0.9	IPF11620	4806328..4	similar to Saccharomyc	orf19.1648	11620	CaRNA1	TRANSCR	enzyme regulator activity
CA1046	0.9	0.9	0.9	IPF11617	complemen	unknown function	orf19.1647	11617	IPF11620	CELL CYC	protein binding
CA1047	1.1	1.0	1.1	IPF11615	4811687..4	RNA-binding proteins (	orf19.9215	11615	IPF11617	CELL CYC	transferase activity
CA1048	1.0	1.0	1.1	IPF15394	complemen	unknown function	orf19.3899	15394	IPF11615	CLASSIFIC	RNA binding
CA1049	1.0	0.9	0.9	TLG1	4818309..4	tSNARE that affects a	orf19.3898	15393	IPF15394	UNCLASSI	DNA binding
CA1050	1.0	0.9	0.9	IPF19897	complemen	unknown function	orf19.3897	19897	CaTLG1	PROTEIN I	transporter activity
CA1051	1.3	1.0	1.1	CHT2	4824049..4	chitinase 2 precursor	orf19.3895	15860	IPF19897	No significant S.c. match	
CA1052	0.9	1.0	1.0	IPF15861	4826085..4	unknown function	orf19.3894	15861	CaCHT2	C-compound and carbohydrate metabolism CELL CYCLE AND DNA PROCESSING SUBCELLULAR LOCALISATION	
CA1053	0.9	1.0	1.0	SCW11.3E	complemen	glucanase gene family	orf19.3893	15862	IPF15861	No significant S.c. match	
CA1054	0.9	0.9	1.0	SAS3	4831154..4	silencing protein (by hc	orf19.2540	16878	CaSCW11.	CLASSIFIC	hydrolase activity

CA1055	1.1	1.0	1.0	IPF17504	complemer	unknown function	orf19.2541	17504	CaSAS3	TRANSCR	transferase activity
CA1056	1.0	1.0	0.9	IPF17503	4835212..4	protein folding and stal	orf19.2542	17503	IPF17504	UNCLASSI	molecular_function unknown
CA1057	0.7	0.5	0.9	IPF17031	4836330..4	unknown function	orf19.2544	17031	IPF17503	PROTEIN FATE [folding modification destination]	
CA1058	1.1	1.0	1.0	DOT6	complemer	involved in derepressic	orf19.2545	19898	IPF17031	UNCLASSI	hydrolase activity
CA1059	1.0	1.0	1.0	TRP2	complemer	anthranilate synthase c	orf19.1008(	13684	CaDOT6	TRANSCR	molecular_function unknown
CA1060	0.9	1.0	1.0	IPF11460	complemer	unknown function	orf19.267	11460	CaTRP2	Amino acid	lyase activity
CA1061	1.0	1.1	1.0	IPF19720.3	4860128..4	unknown function, 3-pr	orf19.1211	19720	IPF11460	TRANSCR	DNA binding
CA1062	1.0	1.0	1.0	IPF14165	complemer	unknown function	orf19.4642	14165	IPF19720.3	No significant S.c. match	
CA1063	0.9	0.9	1.0	NMT1	4866803..4	N-myristoyltransferase	orf19.4641	15592	IPF14165	No significant S.c. match	
CA1064	0.9	1.1	0.9	PWP1	4868492..4	beta-transducin superf	orf19.1211(	15595	CaNMT1	Lipid fatty-a	transferase activity
CA1065	1.0	1.0	1.0	IPF9002	complemer	unknown function	orf19.1210	9002	CaPWP1	UNCLASSI	molecular_function unknown
CA1066	1.1	1.1	1.0	IPF9001	4871411..4	unknown function	orf19.1210	9001	IPF9002	No significant S.c. match	
CA1067	2.3	2.5	2.9	HXT62	4876157..4	sugar transporter	orf19.2023	4109	IPF9001	UNCLASSI	molecular_function unknown
CA1069	1.2	1.4	1.2	HXT5.3F	4880221..4	sugar transporter, 3-pr	orf19.2021	18773	CaHXT62	C-compour	transporter activity
CA1070	1.5	2.5	1.1	HXT61	4881908..4	sugar transporter	orf19.2020	4104	CaHXT5.3f	C-compound and carbohydrate metabolism CELLULAR TRANSPORT AND TRANSPORT MECHANISMS SUBCELLULAR LOCALISATIO	
CA1071	1.0	0.9	0.9	IPF16624	complemer	unknown function	orf19.2534	16624	CaHXT61	C-compour	transporter activity
CA1072	1.0	1.0	1.1	SBH1	4891168..4	involved in translocation into the E		4995	IPF16624	UNCLASSI	molecular_function unknown
CA1073	1.0	1.0	1.0	PRORS.3F	complemer	prolyl-tRNA synthetase	orf19.2533	4994	CaSBH1	PROTEIN I	transporter activity
CA1074	1.0	1.0	1.0	PRORS.5F	complemer	prolyl-tRNA synthetase	orf19.2532	4992	CaPRORS	PROTEIN S	ligase activity
CA1075	1.9	1.6	1.0	IPF4991	complemer	putative membrane pro	orf19.2531	4991	CaPRORS	PROTEIN SYNTHESIS	
CA1076	0.7	0.4	1.1	IPF19721	complemer	similar to Saccharomy	orf19.2239	19721	IPF4991	CELLULAR TRANSPORT AND TRANSPORT MECHANISMS SUBCELLULAR LOCALISATION	
CA1077	1.0	1.0	1.0	IPF13694	complemer	unknown function	orf19.2238	13694	IPF19721	CELL CYC	enzyme regulator activity
CA1078	0.9	0.9	1.0	SPR1	complemer	exo-1,3-beta-glucanas	orf19.2237	19900	IPF13694	CELL CYCLE AND DNA PROCESSING	
CA1079	1.0	0.9	0.9	LIP1	4913733..4	Secretory lipase	orf19.4821	14712	CaSPR1	C-compound and carbohydrate metabolism CELL FATE SUBCELLULAR LOCALISATION	
CA1080	1.0	1.0	1.0	IPF14710	complemer	unknown function	orf19.4820	14710	CaLIP1	Other virulence attributes	
CA1081	1.0	1.0	1.1	IPF14797.3	complemer	unknown function, 3-prime end		14799	IPF14710	UNCLASSI	molecular_function unknown
CA1082	0.9	0.9	1.0	IPF14797	complemer	unknown function	orf19.4818	14797	IPF14797.3	No significant S.c. match	
CA1083	1.0	0.9	1.0	IPF1034	complemer	Similarity to transcriptic	orf19.1204	1034	IPF14797	No significant S.c. match	
CA1084	0.9	0.8	0.9	IPF1036	complemer	unknown function	orf19.4571	1036	IPF1034	TRANSCRIPTION	
CA1085	0.8	0.9	0.8	IPF1038	complemer	unknown function	orf19.4570	1038	IPF1036	No significant S.c. match	
CA1086	0.9	1.0	1.0	IPF1039	4932510..4	unknown function	orf19.1203	1039	IPF1038	No significant S.c. match	
CA1087	1.0	1.0	0.9	MYO5	complemer	Myosin I (by homology	orf19.8357	14461	IPF1039	No significant S.c. match	
CA1088	0.9	1.1	0.9	IPF13319	complemer	unknown function	orf19.740	13319	CaMYO5	CELLULAF	motor activity
CA1089	1.0	0.9	0.9	IPF13799	4947538..4	unknown function	orf19.3945	13799	IPF13319	TRANSCRIPTION SUBCELLULAR LOCALISATION	
CA1090	0.8	0.9	0.9	COX18	complemer	protein required for act	orf19.3946	13798	IPF13799	UNCLASSI	molecular_function unknown
CA1091	1.0	0.8	0.9	SPT4	4950243..4	transcription elongatior	orf19.3947	13796	CaCOX18	ENERGY "	molecular_function unknown
CA1092	1.1	1.1	1.0	YTA7	complemer	26S proteasome subur	orf19.3949	13795	CaSPT4	CELL CYC	transcription regulator activity
CA1093	1.1	1.1	1.0	MSM1	complemer	mitochondrial methiony	orf19.3950	19722	CaYTA7	PROTEIN I	hydrolase activity
CA1094	0.9	0.8	1.0	YIP1	complemer	golgi membrane protei	orf19.3951	16770	CaMSM1	PROTEIN S	ligase activity
CA1095	1.1	1.0	1.0	SMC1	4962077..4	Chromosomal ATPase	orf19.4367	7580	CaYIP1	CELLULAF	molecular_function unknown
CA1096	0.9	1.0	0.9	IPF7578	complemer	unknown function	orf19.4366	7578	CaSMC1	CELL CYC	DNA binding
CA1097	1.0	1.0	1.0	IPF7575	4969468..4	putative endo-exonucle	orf19.4365	7575	IPF7578	No significant S.c. match	
CA1098	1.1	1.0	1.0	IPF12316	complemer	unknown function		12316	IPF7575	TRANSCRIPTION SUBCELLULAR LOCALISATION	
CA1099	1.1	1.1	1.0	IPF12312	complemer	unknown function	orf19.1253(	12312	IPF12316	No significant S.c. match	
CA1100	1.3	1.1	1.0	IPF5681	complemer	unknown function	orf19.1241	5681	IPF12312	No significant S.c. match	
CA1101	1.1	1.1	1.0	IPF5678	complemer	Unknown function	orf19.1241	5678	IPF5681	No significant S.c. match	
CA1102	0.9	1.0	1.0	AKR1	4994071..4	ankyrin repeat-containi	orf19.4950	5675	IPF5678	No significant S.c. match	
CA1103	0.9	0.9	0.9	IPF5673	4997664..4	similar to Saccharomy	orf19.1241	5673	CaAKR1	REGULATI	transferase activity
CA1104	1.1	1.0	1.1	IPF18761	complemer	unknown function		18761	IPF5673	Nucleotide	hydrolase activity
CA1105	1.0	1.0	1.0	IPF18760.3	complemer	unknown function, 3-prime end		18760	IPF18761	UNCLASSI	molecular_function unknown
CA1106	0.9	1.1	0.9	IPF11270	complemer	unknown function	orf19.1269	11270	IPF18760.3	No significant S.c. match	
CA1107	1.0	1.0	0.9	IPF11271	complemer	by homology to S. cerevisiae: ATI		11271	IPF11270	UNCLASSI	RNA binding
CA1108	1.0	1.0	1.0	IPF11273	5006572..5	unknown function	orf19.1269	11273	IPF11271	ENERGY (	transporter activity
CA1109	0.9	0.9	1.1	MRPS9	complemer	ribosomal protein S9 s	orf19.5230	11274	IPF11273	No significant S.c. match	
CA1110	0.8	0.8	0.8	DIS3	5009476..5	3 --5 exoribonuclease	orf19.5229	10406	CaMRPS9	PROTEIN S	structural molecule activity
CA1111	1.8	1.9	1.5	RIB3	complemer	3,4-dihydroxy-2-butanc	orf19.1269	10407	CaDIS3	CELL CYC	RNA binding
CA1112	1.0	1.0	0.9	IPF18758.5	complemer	unknown function, 5-pr	orf19.2282	18758	CaRIB3	Metabolism of vitamins cofactors and prosthetic groups	
CA1113	0.9	0.9	0.9	IPF9996	5018610..5	unknown function	orf19.2285	9996	IPF18758.5	No significant S.c. match	
CA1114	1.0	0.9	1.0	IPF9995	5020524..5	unknown function	orf19.2286	9995	IPF9996	C-compound and carbohydrate metabolism	
CA1115	0.9	0.9	1.0	RPA12	complemer	DNA-directed RNA pol	orf19.2287	9993	IPF9995	UNCLASSI	molecular_function unknown
CA1116	1.1	1.1	1.0	CCT5	5022582..5	T-complex protein 1, e	orf19.2288	9991	CaRPA12	TRANSCR	nucleotidyltransferase activity
CA1117	1.0	0.9	0.9	ARP3	5024605..5	actin related protein (b	orf19.2289	9988	CaCCT5	PROTEIN I	chaperone activity
CA1118	0.7	0.5	0.9	IPF9987	5026113..5	similar to phosphatidyli	orf19.2290	9987	CaARP3	CELLULAF	structural molecule activity
CA1119	1.0	0.9	1.1	IPF7669.3	5028852..5	unknown function, 3-prime end		7669	IPF9987	Lipid fatty-acid and isoprenoid metabolism ""CELL CYCLE AND DNA PROCESSING CELLULAR COMMUNICATION/SIGNAL TRANSDUC	
CA1120	1.3	1.7	1.2	IPF7666	complemer	unknown function	orf19.4056	7666	IPF7669.3	UNCLASSIFIED PROTEINS	
CA1121	1.0	1.1	1.1	IPF6730	complemer	Unknown function	orf19.1604	6730	IPF7666	UNCLASSIFIED PROTEINS	
CA1122	1.1	1.1	1.1	PMS1.3	complemer	DNA mismatch repair p	orf19.1605	6724	IPF6730	UNCLASSI	molecular_function unknown
CA1123	0.8	1.0	0.8	IPF19723	complemer	similar to Saccharomy	orf19.6261	19723	CaPMS1.3	CELL CYC	DNA binding
CA1124	0.9	1.0	1.0	UBP12	complemer	ubiquitin C-terminal hy	orf19.6260	12859	IPF19723	CELLULAF	molecular_function unknown
CA1125	1.0	1.0	1.0	RRP43	complemer	rRNA processing prote	orf19.6259	12854	CaUBP12	PROTEIN I	peptidase activity
CA1126	1.1	1.1	1.0	ECM18.3E	5081283..5	cell wall biogenesis, 3-	orf19.3607	5203	CaRRP43	TRANSCR	RNA binding
CA1127	1.0	1.1	1.1	MSH3	complemer	DNA mismatch repair t	orf19.3608	5201	CaECM18.	UNCLASSIFIED PROTEINS	

CA1128	1.2	1.1	1.0	IPF5198	5086718..5	unknown function	orf19.3609	5198	CaMSH3	CELL CYC	DNA binding
CA1129	1.0	1.0	1.1	IPF5197	5088566..5	unknown function	orf19.3610	5197	IPF5198	UNCLASSI	RNA binding
CA1130	1.0	0.9	1.0	NAG1	complemer	Glucosamine-6-phospl	orf19.9703	2719	IPF5197	No significant S.c. match	
CA1131	1.0	1.0	1.1	NAG2	5093929..5	N-acetyl-glucosamine-	orf19.9704	20073	CaNAG1	No significant S.c. match	
CA1132	1.0	1.0	1.0	IPF2710.RI	complemer	putative permease (by	orf19.9705	2717	CaNAG2	No significant S.c. match	
CA1133	0.9	0.9	1.0	IPF2710.RI	complemer	putative permease (by	orf19.9706	2710	IPF2710.re	CELL RESCUE DEFENSE AND VIRULENCE ""TRANSPORT FACILITATION	
CA1134	1.1	1.2	1.0	IPF2702	5100522..5	unknown function	orf19.2163	2702	IPF2710.re	CELL RESCUE DEFENSE AND VIRULENCE ""TRANSPORT FACILITATION	
CA1135	1.0	1.0	1.0	IMP2	5107351..5	mitochondrial inner me	orf19.1981	8699	IPF2702	UNCLASSI	molecular_function unknown
CA1136	1.0	1.0	0.9	IFN2	complemer	glycerophosphoinosito	orf19.1980	8698	CaIMP2	CELL CYC	peptidase activity
CA1137	1.0	1.0	1.0	IFN1	complemer	glycerophosphoinosito	orf19.1979	8694	CaIFN2	Lipid fatty-acid and isoprenoid metabolism ""SUBCELLULAR LOCALISATION TRANSPORT FACILITATION	
CA1138	1.1	0.9	0.9	IFN3	complemer	glycerophosphoinosito	orf19.1978	8692	CaIFN1	Lipid fatty-acid and isoprenoid metabolism ""SUBCELLULAR LOCALISATION TRANSPORT FACILITATION	
CA1139	1.0	1.1	1.0	IPF9466	complemer	unknown function	orf19.4066	9466	CaIFN3	Lipid fatty-acid and isoprenoid metabolism ""SUBCELLULAR LOCALISATION TRANSPORT FACILITATION	
CA1140	0.9	1.0	1.0	IPF17322.3	complemer	unknown function, 3-pr	orf19.4068	9462	IPF9466	PROTEIN FATE [folding modification destination] ""CELLULAR TRANSPORT AND TRANSPORT MECHANISMS SUBCELLULAR LOCAL	
CA1141	1.0	1.0	1.0	IPF17322.5	complemer	unknown function, 5-pr	orf19.4069	9461	IPF17322.3	C-compound and carbohydrate metabolism TRANSCRIPTION CELL FATE SUBCELLULAR LOCALISATION	
CA1142	0.8	0.5	1.0	IPF9459	complemer	unknown function	orf19.4070	9459	IPF17322.5	No significant S.c. match	
CA1144	1.0	1.0	0.9	SPS19	5135926..5	peroxisomal 2,4-dienoy	orf19.1116	19650	IPF9459	No significant S.c. match	
CA1145	1.0	1.0	1.0	GCS1	5137053..5	ADP-ribosylation facto	orf19.1116	10382	CaSPS19	ENERGY S	oxidoreductase activity
CA1146	1.0	1.0	1.0	CWH8	complemer	putative required prote	orf19.3682	10381	CaGCS1	CELL CYC	protein binding
CA1147	0.9	1.0	1.0	KAP95	complemer	karyopherin-beta prote	orf19.1116	10376	CaCWH8	PROTEIN I	hydrolase activity
CA1148	1.1	1.1	1.1	CAN1	5149963..5	amino acid permease (	orf19.97	10466	CaKAP95	PROTEIN I	protein binding
CA1149	1.1	1.0	1.1	MET223	complemer	protein ser/thr phospha	orf19.99	10464	CaCAN1	Amino acid metabolism CELLULAR TRANSPORT AND TRANSPORT MECHANISMS SUBCELLULAR LOCALISATION TRANSPORT FA	
CA1150	0.9	1.0	1.0	RIM9	5154830..5	regulator for sporulatio	orf19.101	10461	CaMET223	Amino acid metabolism ""CELL RESCUE DEFENSE AND VIRULENCE ""	
CA1151	1.1	1.0	1.0	IPF10459	complemer	unknown function	orf19.102	10459	CaRIM9	CELL FATE	
CA1152	1.0	1.0	1.0	IPF10457	5157592..5	nuclear fusion protein-l	orf19.103	10457	IPF10459	CELL CYCLE AND DNA PROCESSING SUBCELLULAR LOCALISATION	
CA1153	0.9	1.0	1.0	IPF10455	5158194..5	unknown function	orf19.104	10455	IPF10457	CELL FATE SUBCELLULAR LOCALISATION	
CA1154	1.0	1.0	1.1	MET221	5159447..5	protein ser/thr phospha	orf19.105	10454	IPF10455	CELL CYCLE AND DNA PROCESSING	
CA1155	1.0	1.0	1.0	JA2	complemer	ATP-dependent RNA h	orf19.107	10452	CaMET221	Amino acid	hydrolase activity
CA1156	0.8	0.9	0.9	RLR1	5165198..5	hypothetical regulatory	orf19.4123	10133	CaJA2	TRANSCR	RNA binding,helicase activity
CA1157	0.9	1.0	1.0	PZF1	complemer	TFIIIA (transcription ini	orf19.4125	13278	CaRLR1	TRANSCRIPTION PROTEIN SYNTHESIS	
CA1158	1.1	1.2	1.0	IPF13275	5172792..5	unknown function	orf19.4127	13275	CaPZF1	TRANSCR	transcription regulator activity
CA1159	1.0	1.0	1.1	IPF13423	complemer	unknown function	orf19.4128	13423	IPF13275	SUBCELLU	molecular_function unknown
CA1160	1.1	1.2	1.0	IPF10262	5182142..5	unknown function	orf19.2726	10262	IPF13423	UNCLASSIFIED PROTEINS	
CA1161	0.9	1.0	1.0	GRX3	5183340..5	glutaredoxin-like protei	orf19.2727	10259	IPF10262	UNCLASSI	molecular_function unknown
CA1162	1.0	1.0	1.0	IPF10258	complemer	similar to Saccharomyc	orf19.2728	10258	CaGRX3	PROTEIN I	oxidoreductase activity
CA1163	0.9	0.9	1.0	IPF15357	complemer	unknown function	orf19.2730	20076	IPF10258	CELL CYC	DNA binding
CA1164	1.0	1.0	1.0	ARP8	5191111..5	actin-related protein (b	orf19.1086	14479	IPF15357	TRANSCR	transcription regulator activity
CA1165	0.6	0.7	0.5	IPF19902	5196466..5	unknown function	orf19.1086	19902	CaARP8	SUBCELLU	molecular_function unknown
CA1166	1.1	1.0	1.1	IFO1	5204897..5	Unknown function	orf19.1780	16648	IPF19902	No significant S.c. match	
CA1167	1.0	1.0	1.0	IPF16646	5206503..5	unknown function	orf19.1782	16646	CaIFO1	UNCLASSIFIED PROTEINS	
CA1168	0.9	1.9	0.9	IPF13017	complemer	unknown function	orf19.1785	13017	IPF16646	UNCLASSI	molecular_function unknown
CA1169	0.9	1.1	0.9	IPF16120.3	5217924..5	unknown function, 3-pr	orf19.2751	13270	IPF13017	No significant S.c. match	
CA1170	0.9	1.0	1.0	IPF13268	complemer	unknown function	orf19.2749	13268	IPF16120.3	TRANSPORT FACILITATION	
CA1171	1.1	1.0	1.0	IPF13264	5222162..5	zinc-finger transcriptio	orf19.2748	13264	IPF13268	UNCLASSI	molecular_function unknown
CA1172	1.1	0.9	0.7	RGT1	5228722..5	Regulator of glucose-ir	orf19.2747	14445	IPF13264	Amino acid metabolism Nitrogen and sulphur metabolism TRANSCRIPTION SUBCELLULAR LOCALISATION	
CA1173	1.0	1.0	1.0	IPF14254	complemer	unknown function	orf19.4768	14254	CaRGT1	C-compour	DNA binding
CA1174	0.9	1.0	0.9	IPF14255	complemer	unknown function	orf19.4767	14255	IPF14254	No significant S.c. match	
CA1175	0.9	0.8	0.9	ARG81	5242145..5	transcription factor pos	orf19.4766	14033	IPF14255	TRANSCRIPTION	
CA1176	1.0	1.2	1.0	CHS5	5250988..5	Chitin biosynthesis pro	orf19.807	5058	CaARG81	Amino acid	transcription regulator activity
CA1177	1.0	1.0	1.0	VMA7	complemer	vacuolar ATPase (by h	orf19.806	5061	CaCHS5	C-compour	molecular_function unknown
CA1178	0.9	1.0	1.0	IPF5062	5253582..5	unknown function	orf19.805	5062	CaVMA7	CELLULAF	transporter activity
CA1179	0.9	1.0	1.0	IPF5064	complemer	ADP/ATP carrier prote	orf19.804	5064	IPF5062	UNCLASSI	molecular_function unknown
CA1180	1.1	1.0	1.4	UGA12.3F	complemer	4-aminobutyrate aminc	orf19.803	19652	IPF5064	Nucleotide	transporter activity
CA1181	0.9	0.9	1.0	UGA12.5F	complemer	4-aminobutyrate aminc	orf19.802	19651	CaUGA12.	Amino acid metabolism Nitrogen and sulphur metabolism	
CA1182	1.0	1.0	1.1	IPF19724	5258815..5	similar to Saccharomyc	orf19.801	19724	CaUGA12.	Amino acid metabolism Nitrogen and sulphur metabolism	
CA1183	1.0	1.0	1.0	IPF14805	complemer	acetyl-coenzyme A trai	orf19.1126	14805	IPF19724	SUBCELLU	DNA binding
CA1184	1.1	1.0	1.1	IPF14802	5268529..5	unknown function		14802	IPF14805	UNCLASSI	molecular_function unknown
CA1185	1.1	1.1	1.0	IPF19903	complemer	unknown function	orf19.1126	19903	IPF14802	UNCLASSI	molecular_function unknown
CA1186	0.8	0.8	0.9	IPF6930	5272287..5	unknown function	orf19.3785	6930	IPF19903	No significant S.c. match	
CA1187	1.1	1.1	1.0	QRI7	complemer	putative glycoprotease	orf19.1126	19904	IPF6930	PROTEIN FATE [folding modification destination]	
CA1188	1.2	1.8	1.3	RPL30.3	complemer	RNA binding, 3-prime end (by hor		6920	CaQRI7	PROTEIN I	molecular_function unknown
CA1189	1.0	1.5	1.2	RPL24A	5276914..5	ribosomal protein L24 (	orf19.1126	18743	CaRPL30.3	PROTEIN S	structural molecule activity
CA1190	1.1	1.4	1.3	CIP1	5280520..5	Cadmium induced prot	orf19.7761	11929	CaRPL24A	PROTEIN S	RNA binding
CA1191	0.4	0.5	0.3	CAN2	5283094..5	amino acid permease (	orf19.111	11927	CaCIP1	No significant S.c. match	
CA1192	1.0	0.9	0.9	IPF11926	complemer	unknown function		11926	CaCAN2	Amino acid	transporter activity
CA1193	0.9	1.0	0.9	MSY1	complemer	tyrosyl-tRNA synthetas	orf19.7756	11923	IPF11926	No significant S.c. match	
CA1194	1.0	1.0	1.0	MET222	complemer	protein ser/thr phospha	orf19.7752	17216	CaMSY1	PROTEIN S	ligase activity
CA1195	0.9	0.9	0.9	KAR5	complemer	nuclear fusion protein-l	orf19.7750	17213	CaMET222	Amino acid metabolism ""CELL RESCUE DEFENSE AND VIRULENCE ""	
CA1196	1.0	0.8	0.9	IPF10394	complemer	unknown function		10394	CaKAR5	CELL FATI	molecular_function unknown
CA1197	0.9	0.9	1.0	IFG1	complemer	probable d-amino acid	orf19.1087	10395	IPF10394	No significant S.c. match	
CA1198	1.0	1.0	1.1	IPF10399	5299982..5	unknown function	orf19.1087	10399	CaIFG1	No significant S.c. match	
CA1199	1.1	1.0	1.0	IPF10404	5304747..5	unknown function	orf19.1087	10404	IPF10399	UNCLASSI	structural molecule activity
CA1200	1.0	0.9	1.0	IPF10223	complemer	putative serine/threonii	orf19.1233	10223	IPF10404	UNCLASSI	molecular_function unknown

CA1201	1.0	1.0	1.0	IPF19906	5315004..5	unknown function	orf19.4869	19906	IPF10223	CELL CYC protein kinase activity
CA1202	0.9	0.9	0.9	DBP3	5316825..5	ATP-dependent RNA h	orf19.1233	10215	IPF19906	Nitrogen and sulphur metabolism TRANSCRIPTION SUBCELLULAR LOCALISATION
CA1203	1.0	1.2	1.0	IPF3937	complemer	Unknown function	orf19.8487	3937	CaDBP3	TRANSCR RNA binding,helicase activity
CA1204	0.8	0.6	0.9	IPF14524	5328163..5	unknown function	orf19.8486	14524	IPF3937	No significant S.c. match
CA1205	0.9	1.0	0.9	SSK2	complemer	MAP kinase kinase kin	orf19.1125	13431	IPF14524	REGULATI oxidoreductase activity
CA1206	1.1	1.0	1.0	HUB1	5339823..5	Ubiquitin-like modifier (by homolog		15404	CaSSK2	CELL RES protein kinase activity,signal transducer activity
CA1207	0.9	1.0	1.1	PPG1	complemer	Phosphoprotein phosp	orf19.1125	18739	CaHUB1	UNCLASSIFIED PROTEINS
CA1208	1.0	1.0	1.0	IPF17195	5342795..5	unknown function	orf19.1125	17195	CaPPG1	ENERGY protein phosphatase activity
CA1209	0.9	0.7	1.1	ARG8	complemer	acetylornithine aminotr	orf19.1125	13486	IPF17195	TRANSCRIPTION SUBCELLULAR LOCALISATION
CA1210	1.1	1.2	0.9	IPF7030	5350146..5	unknown function	orf19.7778	7030	CaARG8	Amino acid transferase activity
CA1211	1.2	1.0	1.1	IPF7031.3	complemer	unknown function, 3-prime end		7031	IPF7030	TRANSCR hydrolase activity
CA1212	1.1	1.1	1.1	IPF7033	complemer	unknown function	orf19.7777	7033	IPF7031.3	UNCLASSI molecular_function unknown
CA1213	1.0	1.1	1.0	VPS15.5F	5353439..5	serine/threonine protei	orf19.7776	7035	IPF7033	TRANSCR RNA binding
CA1214	1.0	1.0	1.1	VPS15.53F	5355345..5	serine/threonine protei	orf19.129	7038	CaVPS15.5	PROTEIN I protein kinase activity
CA1215	0.9	0.7	1.0	VPS15.3F	5356875..5	serine/threonine protei	orf19.7773	7041	CaVPS15.5	PROTEIN FATE [folding modification destination] ""CELLULAR TRANSPORT AND TRANSPORT MECHANISMS SUBCELLULAR LOCAL
CA1216	1.0	0.9	1.0	EBP1	5359651..5	NADPH dehydrogenas	orf19.7772	7044	CaVPS15.3	PROTEIN FATE [folding modification destination] ""CELLULAR TRANSPORT AND TRANSPORT MECHANISMS SUBCELLULAR LOCAL
CA1217	1.1	1.0	1.1	IPF13909	complemer	Unknown function	orf19.7770	13909	CaEBP1	ENERGY
CA1218	0.9	0.9	0.9	CDC20	complemer	anaphase promoting c	orf19.7769	13908	IPF13909	No significant S.c. match
CA1219	1.0	1.1	1.1	PRC2	complemer	carboxypeptidase y pre	orf19.4135	14986	CaCDC20	CELL CYC enzyme regulator activity
CA1220	1.0	1.1	1.0	IPF14985	5372398..5	unknown function	orf19.4134	14985	CaPRC2	PROTEIN FATE [folding modification destination] ""SUBCELLULAR LOCALISATION
CA1221	1.0	1.0	0.9	IPF14872	5373766..5	unknown function	orf19.4133	14872	IPF14985	UNCLASSI molecular_function unknown
CA1222	0.9	1.0	1.1	IPF14871	complemer	unknown function	orf19.4132	14871	IPF14872	UNCLASSI molecular_function unknown
CA1223	1.0	1.1	1.0	IPF14870	5376671..5	unknown function	orf19.4131	14870	IPF14871	No significant S.c. match
CA1224	1.0	1.1	1.0	IPF15662	complemer	unknown function	orf19.4156	15662	IPF14870	UNCLASSI molecular_function unknown
CA1225	0.8	0.9	0.7	SPS20	complemer	peroxisomal 2,4-dienoy	orf19.4157	15661	IPF15662	No significant S.c. match
CA1226	1.0	1.0	1.0	IPF15660	complemer	putative mitochondrial	orf19.4159	15660	CaSPS20	ENERGY SUBCELLULAR LOCALISATION
CA1227	0.9	1.0	1.0	IPF16082	5392948..5	unknown function	orf19.4160	16082	IPF15660	CELLULAF transporter activity
CA1228	1.0	0.9	0.9	IPF16081	complemer	unknown function	orf19.4161	16081	IPF16082	PROTEIN I molecular_function unknown
CA1229	1.0	1.0	1.0	MLH1	5395673..5	DNA mismatch repair p	orf19.4162	15970	IPF16081	UNCLASSI molecular_function unknown
CA1230	1.2	1.6	0.7	SSA4	5401884..5	cahsp70 mRNA for hea	orf19.4980	11819	CaMLH1	CELL CYC DNA binding
CA1231	1.0	1.1	0.9	IPF11817	complemer	unknown function	orf19.4981	11817	CaSSA4	CELL RES chaperone activity
CA1232	1.0	1.0	0.9	IPF11815	complemer	similar to Saccharomy	orf19.4982	11815	IPF11817	TRANSPO molecular_function unknown
CA1233	1.0	1.1	0.9	IPF11814	complemer	unknown function	orf19.4983	11814	IPF11815	Lipid fatty-acid and isoprenoid metabolism
CA1234	1.1	1.1	1.3	IPF18732	5413276..5	histidine-rich glycoprot	orf19.8330	18732	IPF11814	No significant S.c. match
CA1235	1.1	1.2	1.2	IPF20079	complemer	unknown function	orf19.8332	20079	IPF18732	No significant S.c. match
CA1236	1.0	0.9	0.9	YPT6	5418379..5	GTP-binding protein of	orf19.8333	15785	IPF20079	UNCLASSI molecular_function unknown
CA1237	1.6	1.7	1.2	IPF15784	complemer	unknown function	orf19.8334	15784	CaYPT6	CELLULAF hydrolase activity
CA1238	1.7	1.4	1.5	IPF15781	5420640..5	unknown function	orf19.8335	15781	IPF15784	No significant S.c. match
CA1239	1.1	1.0	0.9	HSP60	5422161..5	Heat Shock Protein 60	orf19.717	14025	IPF15781	No significant S.c. match
CA1240	1.0	1.0	1.0	IPF14026	5424324..5	similar to Saccharomy	orf19.8337	14026	CaHSP60	PROTEIN I chaperone activity
CA1241	1.2	1.1	1.2	LIP8	complemer	Secretory lipase	orf19.8925	7914	IPF14026	TRANSCR transcription regulator activity
CA1242	1.0	0.9	1.0	IPF19908	5452014..5	unknown function	orf19.1344	19908	CaLIP8	Other virulence attributes
CA1243	0.9	0.9	1.1	IPF7922	complemer	unknown function	orf19.8923	7922	IPF19908	No significant S.c. match
CA1244	1.0	1.1	1.1	IPF12767	5465047..5	unknown function	orf19.2624	12767	IPF7922	Amino acid transcription regulator activity
CA1245	1.3	1.6	1.1	IMH3.EXOI	complemer	IMP dehydrogenase, e	orf19.7689	3873	IPF12767	No significant S.c. match
CA1246	1.1	1.4	1.0	IMH3.EXOI	complemer	IMP dehydrogenase, e	orf19.19	3872	CaIMH3.ex	Nucleotide oxidoreductase activity
CA1247	1.1	1.2	1.1	IPF3870	complemer	similar to Saccharomy	orf19.20	3870	CaIMH3.ex	Nucleotide metabolism Purine ribonucleotide metabolism
CA1248	1.0	1.1	1.1	IPF3866	complemer	unknown function	orf19.7692	3866	IPF3870	CELL RES protein phosphatase activity
CA1249	1.0	1.0	1.1	SPE4	5482001..5	spermine synthase (by	orf19.4960	9628	IPF3866	SUBCELLU molecular_function unknown
CA1250	0.9	1.0	1.0	IPF9626	5483727..5	unknown function	orf19.1242	9626	CaSPE4	Secondary transferase activity
CA1251	0.9	1.0	1.0	IPF9624	complemer	similar to Saccharomy	orf19.1242	9624	IPF9626	No significant S.c. match
CA1252	0.9	0.9	0.9	RPN1	5489006..5	26S proteasome regula	orf19.1242	9623	IPF9624	CONTROL molecular_function unknown
CA1253	1.0	1.0	0.9	FDH3.3F	complemer	formate dehydrogenas	orf19.1774	9034	CaRPN1	PROTEIN I peptidase activity,signal transducer activity
CA1255	1.0	0.9	0.9	IPF9030	5497238..5	unknown function	orf19.1776	9030	CaFDH3.3f	ENERGY
CA1256	1.2	1.0	1.0	UBP15	complemer	ubiquitin-specific prote	orf19.1777	9029	IPF9030	UNCLASSI nucleotidyltransferase activity
CA1257	1.0	1.0	1.1	IPF18725	complemer	unknown function	orf19.1067	18725	CaUBP15	PROTEIN I peptidase activity
CA1258	1.0	0.9	1.3	RPN8	5510003..5	26S proteasome regula	orf19.1067	18724	IPF18725	UNCLASSI molecular_function unknown
CA1259	1.0	1.0	1.1	IPF3174	5511244..5	Farnesyl transferase (t	orf19.1067	3174	CaRPN8	PROTEIN I peptidase activity
CA1260	1.1	1.0	1.0	IPF3170	5512965..5	unknown function	orf19.3166	3170	IPF3174	Lipid fatty-a transferase activity
CA1261	0.9	1.0	1.0	IPF9887.3f	5519470..5	unknown function, 3-pr	orf19.1240	9887	IPF3170	UNCLASSI molecular_function unknown
CA1262	1.0	1.0	1.0	MDR1	5527606..5	Mac1p interacting prot	orf19.1244	9881	IPF9887.3e	UNCLASSI molecular_function unknown
CA1263	1.0	1.3	1.1	IPF9880	complemer	unknown function	orf19.1246	9880	CaMDR1	TRANSCR enzyme regulator activity
CA1264	0.8	0.7	0.8	STH1	complemer	helicase related protei	orf19.239	10096	IPF9880	No significant S.c. match
CA1265	1.0	0.8	1.2	IPF10510	5542480..5	unknown function	orf19.240	10510	CaSTH1	CELL CYC DNA binding,helicase activity
CA1266	1.0	1.0	1.0	SAP8	complemer	aspartic protease	orf19.242	10508	IPF10510	UNCLASSI molecular_function unknown
CA1267	0.9	1.0	1.0	YSY6	5546802..5	protein involved in the secretory pa		10503	CaSAP8	PROTEIN FATE [folding modification destination] ""SUBCELLULAR LOCALISATION Other virulence attributes
CA1268	1.0	0.9	1.0	IPF14693	complemer	unknown function	orf19.3159	14693	CaYSY6	PROTEIN FATE [folding modification destination] ""CELLULAR TRANSPORT AND TRANSPORT MECHANISMS
CA1269	1.0	0.9	1.0	IPF19726	5552779..5	unknown function	orf19.1066	19726	IPF14693	UNCLASSI molecular_function unknown
CA1270	1.0	1.1	1.2	IPF13504	5558228..5	unknown function	orf19.1066	13504	IPF19726	UNCLASSI molecular_function unknown
CA1271	1.0	0.9	0.9	HIT1	5569225..5	required for growth at h	orf19.2723	6402	IPF13504	No significant S.c. match
CA1272	1.0	1.0	1.1	CGR1	complemer	Cell growth protein (by	orf19.2722	18718	CaHIT1	CELL RES molecular_function unknown
CA1273	1.0	1.0	1.0	CCT4	5572601..5	Component of chaperc	orf19.2720	6406	CaCGR1	UNCLASSI molecular_function unknown

CA1274	0.9	0.9	0.8	SAS10	complemer	Involved in silencing (b	orf19.2717	6410	CaCCT4	PROTEIN I	chaperone activity
CA1275	1.0	1.1	1.0	RPC53	5576302..5	DNA-directed RNA pol	orf19.2715	19910	CaSAS10	TRANSCR	RNA binding
CA1277	1.0	1.0	1.0	IPF10837	5581353..5	unknown function	orf19.3630	10837	CaRPC53	TRANSCR	nucleotidyltransferase activity
CA1278	0.9	0.9	1.0	IPF10835	5584841..5	unknown function	orf19.3629	10835	IPF10837	TRANSCR	transferase activity
CA1279	1.0	1.1	1.0	RSP5	5588206..5	ubiquitin-protein ligase	orf19.3628	10833	IPF10835	UNCLASSI	molecular_function unknown
CA1280	0.9	1.0	0.9	IPF10828	5591405..5	unknown function	orf19.3627	10828	CaRSP5	PROTEIN I	ligase activity
CA1281	1.1	0.8	0.9	IPF10171.E	complemer	unknown function, exo	orf19.645	10174	IPF10828	UNCLASSIFIED PROTEINS	
CA1282	0.9	0.9	1.0	IPF10171.E	complemer	unknown function, exon 2		10173	IPF10171.e	C-compound and carbohydrate metabolism CELLULAR TRANSPORT AND TRANSPORT MECHANISMS SUBCELLULAR LOCALISATIO	
CA1283	1.1	1.1	1.1	IPF10171.E	complemer	unknown function, exo	orf19.644	10171	IPF10171.e	C-compound and carbohydrate metabolism CONTROL OF CELLULAR ORGANIZATION TRANSPORT FACILITATION	
CA1284	1.0	0.9	1.0	IPF10168.3	5603150..5	unknown function, 3-pr	orf19.8257	10168	IPF10171.e	C-compound and carbohydrate metabolism CELLULAR TRANSPORT AND TRANSPORT MECHANISMS REGULATION OF/INTERACTIC	
CA1285	1.0	1.0	1.6	SKP1	5610067..5	kinetochore protein co	orf19.1190	5549	IPF10168.3	CELL CYC	molecular_function unknown
CA1286	1.0	1.0	1.0	IPF5546	5611387..5	unknown function	orf19.1190	5546	CaSKP1	Amino acid	protein binding
CA1287	1.0	1.0	1.1	IPF5545	complemer	unknown function	orf19.4430	5545	IPF5546	CELL CYCLE AND DNA PROCESSING TRANSCRIPTION SUBCELLULAR LOCALISATION	
CA1288	0.9	1.2	1.0	KSP1	5616957..5	SERINE/THREONINE-	orf19.4432	5540	IPF5545	PROTEIN I	molecular_function unknown
CA1289	0.7	1.0	0.7	ZUO1	5623854..5	Zuotin, a putative Z-DN	orf19.1022	16213	CaKSP1	SUBCELLU	protein kinase activity
CA1290	1.1	0.9	1.0	IPF16212	complemer	unknown function	orf19.2710	16212	CaZUO1	SUBCELLU	chaperone activity
CA1291	1.0	1.0	1.0	IPF16752	complemer	unknown function	orf19.2711	16752	IPF16212	C-compour	enzyme regulator activity
CA1292	0.9	0.9	0.9	SRB2.3	5629009..5	DNA-directed RNA polymerase II I		6419	IPF16752	TRANSCR	transcription regulator activity
CA1293	0.9	1.0	1.0	HCA4	5630031..5	Can suppress the U14	orf19.2712	6418	CaSRB2.3	TRANSCR	transcription regulator activity
CA1294	1.0	1.0	0.9	MSH5.3F	complemer	Meiosis-specific protei	orf19.1022	6415	CaHCA4	TRANSCR	RNA binding,helicase activity
CA1296	1.0	1.0	1.0	IPF18712	5635451..5	unknown function		18712	CaMSH5.3	CELL CYC	molecular_function unknown
CA1297	1.1	1.1	1.0	ACF2	5637459..5	endo-1,3-beta-glucana	orf19.3417	14734	IPF18712	UNCLASSIFIED PROTEINS	
CA1298	1.4	2.4	1.2	RPL32	5640699..5	ribosomal protein L32		12449	CaACF2	PROTEIN I	hydrolase activity
CA1299	1.0	1.0	1.0	PTK2	complemer	serine /threonine prote	orf19.3415	12448	CaRPL32	PROTEIN S	structural molecule activity
CA1300	0.5	0.3	1.0	SLY41	5650076..5	vesicular transport (by	orf19.4199	12346	CaPTK2	CELLULAF	protein kinase activity
CA1301	0.9	0.9	1.0	YHM2	complemer	mtDNA stabilizing prot	orf19.4197	12348	CaSLY41	CELLULAF	molecular_function unknown
CA1302	1.1	1.0	1.1	FCA1.3	5654565..5	cytosine deaminase, 3-prime end		13513	CaYHM2	CELL CYC	transporter activity
CA1303	1.1	1.0	1.0	TFB4	complemer	component of RNA pol	orf19.4194	13511	CaFCA1.3	Nucleotide	hydrolase activity
CA1304	2.1	2.2	1.5	RPS13.3	complemer	ribosomal protein, 3-prime end (by		13509	CaTFB4	CELL CYC	transcription regulator activity
CA1305	1.0	1.0	1.1	IPF13508	complemer	unknown function	orf19.4193	13508	CaRPS13.3	PROTEIN S	structural molecule activity
CA1306	1.1	1.1	1.0	CDC14.3	5659135..5	protein phosphatase, 3	orf19.4192	13507	IPF13508	UNCLASSI	molecular_function unknown
CA1307	0.9	0.8	1.0	IPF13506.3	complemer	unknown function, 3-prime end		13506	CaCDC14.	CELL CYC	protein phosphatase activity
CA1308	1.0	1.0	1.1	IPF8642	5663134..5	unknown function	orf19.1145	8642	IPF13506.3	No significant S.c. match	
CA1309	1.0	1.1	1.1	IPF8627	5671739..5	unknown function	orf19.1145	8627	IPF8642	UNCLASSIFIED PROTEINS	
CA1310	1.0	1.0	0.9	IPF14232	5675956..5	unknown function	orf19.1111	14232	IPF8627	TRANSCRIPTION SUBCELLULAR LOCALISATION	
CA1311	1.0	1.0	1.0	IPF14233	5678043..5	Putative transthyretin p	orf19.3633	14233	IPF14232	No significant S.c. match	
CA1312	0.9	0.9	1.0	IPF14241	complemer	unknown function	orf19.1111	14241	IPF14233	UNCLASSIFIED PROTEINS	
CA1313	1.0	0.9	0.9	IPF14126	5686766..5	unknown function	orf19.3638	14126	IPF14241	No significant S.c. match	
CA1314	0.9	0.9	1.0	MAG1	complemer	3-methyladenine DNA	orf19.3639	14127	IPF14126	No significant S.c. match	
CA1315	1.1	1.0	1.0	URA2.5EO	complemer	multifunctional pyrimidi	orf19.9896	10801	CaMAG1	CELL CYC	DNA binding
CA1316	1.0	1.0	1.0	IPF14587.3	5701736..5	unknown function, 3-prime end		14587	CaURA2.5	Nucleotide	transferase activity
CA1318	1.0	1.0	0.9	IPF3147	complemer	Nuclear valosin-contai	orf19.4219	3147	IPF14587.3	No significant S.c. match	
CA1319	0.8	0.7	0.8	IPF3144	complemer	unknown function	orf19.4220	3144	IPF3147	CLASSIFIC	helicase activity
CA1320	1.0	0.9	1.0	IPF3143	complemer	similar to Saccharomy	orf19.4221	3143	IPF3144	UNCLASSI	molecular_function unknown
CA1321	0.9	0.9	1.0	IPF3141	5715614..5	similar to Saccharomy	orf19.4222	3141	IPF3143	CELL CYC	DNA binding
CA1322	0.6	0.5	0.8	GCD11	complemer	Translation initiation fa	orf19.4223	3140	IPF3141	PROTEIN I	enzyme regulator activity
CA1323	0.7	0.5	1.0	IPF6675	complemer	unknown function	orf19.1309	6675	CaGCD11	TRANSCR	translation regulator activity
CA1324	0.7	0.5	1.0	IPF6676	5723687..5	polytopic membrane pr	orf19.1308	6676	IPF6675	No significant S.c. match	
CA1325	0.9	0.8	1.0	IPF6678	complemer	unknown function	orf19.1307	6678	IPF6676	CELL RES	molecular_function unknown
CA1326	1.0	1.0	1.0	IPF6679	complemer	unknown function	orf19.1306	6679	IPF6678	UNCLASSIFIED PROTEINS	
CA1327	0.8	0.8	0.9	IPF6680	complemer	unknown function	orf19.1305	6680	IPF6679	No significant S.c. match	
CA1328	0.9	0.7	0.9	RRP4	5730636..5	3 ->5 exoribonuclease	orf19.1304	6682	IPF6680	UNCLASSI	transferase activity
CA1329	0.9	0.8	0.9	MRF2	complemer	peptide chain release f	orf19.1303	19626	CaRRP4	No significant S.c. match	
CA1330	1.0	0.9	0.9	IPF11713	5738047..5	unknown function	orf19.8744	11713	CaMRF2	PROTEIN S	translation regulator activity
CA1331	0.9	1.0	1.0	IPF11714	complemer	unknown function		11714	IPF11713	Lipid fatty-acid and isoprenoid metabolism """"Metabolism of vitamins cofactors and prosthetic groups """"PROTEIN FATE [folding modifica	
CA1332	0.9	0.9	0.9	IPF11716	5740711..5	unknown function	orf19.1150	11716	IPF11714	No significant S.c. match	
CA1333	0.9	0.5	1.4	MRF1	complemer	mitochondrial respirato	orf19.8742	11717	IPF11716	Nitrogen and sulphur metabolism TRANSCRIPTION SUBCELLULAR LOCALISATION	
CA1334	1.1	0.9	1.0	IPF19912	complemer	unknown function	orf19.8740	19912	CaMRF1	TRANSCRIPTION SUBCELLULAR LOCALISATION	
CA1335	1.0	0.9	1.0	TRA1	5749821..5	phosphatidylinositol kir	orf19.3451	5342	IPF19912	CELL RESCUE DEFENSE AND VIRULENCE ""CELL FATE	
CA1336	1.0	0.9	0.9	IPF5334.E	complemer	unknown function, exon 2		5335	CaTRA1	TRANSCR	transferase activity
CA1338	1.0	1.1	1.0	IPF5333	5758841..5	unknown function	orf19.1095	5333	IPF5334.e	UNCLASSI	molecular_function unknown
CA1339	1.0	1.1	1.0	IPF5330	5762965..5	unknown function	orf19.1095	5330	IPF5333	UNCLASSI	molecular_function unknown
CA1340	1.1	1.0	1.1	VPS4	5770457..5	vacuolar sorting protei	orf19.4339	11343	IPF5330	No significant S.c. match	
CA1341	0.9	0.9	1.0	IPF11344	complemer	unknown function	orf19.4340	11344	CaVPS4	PROTEIN I	hydrolase activity
CA1342	1.1	1.1	0.9	IPF11347	complemer	unknown function		11347	IPF11344	CELLULAR TRANSPORT AND TRANSPORT MECHANISMS REGULATION OF/INTERACTION WITH CELLULAR ENVIRONMENT	
CA1343	1.0	1.0	1.0	IPF14624	5779872..5	unknown function		14624	IPF11347	No significant S.c. match	
CA1344	0.9	0.9	0.9	IPF14623	5780307..5	unknown function	orf19.4342	14623	IPF14624	No significant S.c. match	
CA1345	1.1	1.2	1.0	IPF6003	5788850..5	similar to Saccharomy	orf19.1490	6003	IPF14623	CELLULAF	transcription regulator activity
CA1346	1.0	1.0	1.0	KRE2.3F	complemer	secretory pathway protein, 3-prime		18700	IPF6003	CELL FATE	signal transducer activity
CA1349	0.9	1.1	1.0	PRP12	5804530..5	involved in early matur	orf19.4351	12922	CaKRE2.3	No significant S.c. match	
CA1350	1.0	1.1	1.1	MCM2	complemer	replication licensing fa	orf19.4354	15306	CaPRP12	TRANSCR	hydrolase activity
CA1351	1.0	1.0	1.1	PCL2	complemer	G1/S specific cyclin	orf19.403	8834	CaMCM2	CELL CYC	DNA binding

CA1352	1.0	1.0	1.0	VCX1	complemer	Ca2+-transport by hon	orf19.405	16576	CaPCL2	CELL CYC	protein kinase activity,enzyme regulator activity
CA1353	0.9	0.8	1.4	ERG1	complemer	squalene epoxidase	orf19.406	19729	CaVCX1	CELLULAF	transporter activity
CA1354	0.9	1.0	0.9	GCD6	complemer	translation initiation fac	orf19.407	10142	CaERG1	Lipid fatty-a	oxidoreductase activity
CA1355	1.2	1.1	1.0	IPF10138.3	complemer	unknown function, 3-pr	orf19.408	10140	CaGCD6	PROTEIN S	translation regulator activity
CA1356	0.9	0.9	1.0	IPF10138.5	complemer	unknown function, 5-pr	orf19.409	10138	IPF10138.3	No significant S.c. match	
CA1357	1.0	1.1	1.1	ARH1	5829588..5	adrenodoxin reductase	orf19.410	10134	IPF10138.5	UNCLASSI	molecular_function unknown
CA1358	1.0	1.0	1.0	NIF3	complemer	Ngg1p-interacting facto	orf19.4406	11837	CaARH1	Lipid fatty-a	transporter activity
CA1359	1.0	0.9	1.0	IPF11842	complemer	unknown function	orf19.4405	11842	CaNIF3	TRANSCR	molecular_function unknown
CA1360	1.1	1.0	1.0	IPF14598	5844203..5	unknown function	orf19.4404	14598	IPF11842	CELL CYCLE AND DNA PROCESSING	
CA1361	1.0	1.0	1.0	PEP5	5846748..5	vacuolar biogenesis pr	orf19.4403	11853	IPF14598	No significant S.c. match	
CA1362	0.9	0.9	1.0	PEX3	complemer	PEROXISOMAL MEMI	orf19.4426	5552	CaPEP5	PROTEIN I	molecular_function unknown
CA1363	1.0	0.9	1.0	IPF5556	5857554..5	acid phosphatase (by I	orf19.4424	5556	CaPEX3	CELLULAF	molecular_function unknown
CA1364	1.1	1.0	0.9	IPF5561	5859512..5	unknown function	orf19.4423	5561	IPF5556	No significant S.c. match	
CA1365	0.9	0.9	1.1	IPF11849	5867837..5	unknown function	orf19.4459	11849	IPF5561	UNCLASSIFIED PROTEINS	
CA1366	1.0	0.9	1.1	IPF11847	5871971..5	unknown function	orf19.4457	11847	IPF11849	UNCLASSIFIED PROTEINS	
CA1367	1.0	1.0	1.1	IPF16755	5882980..5	unknown function	orf19.4966	16755	IPF11847	PROTEIN I	protein binding
CA1368	0.9	0.9	0.9	IPF15969	complemer	unknown function	orf19.4965	15969	IPF16755	C-compour	transporter activity
CA1369	1.0	1.0	1.0	IPF15968	complemer	unknown function	orf19.4964	15968	IPF15969	No significant S.c. match	
CA1370	1.0	1.0	0.9	TCI1	complemer	protein phosphatase T	orf19.4963	15966	IPF15968	UNCLASSI	molecular_function unknown
CA1371	0.9	0.9	0.9	IPF19731	5890450..5	unknown function	orf19.4962	19731	CaTCI1	UNCLASSI	molecular_function unknown
CA1372	0.8	0.9	0.6	IPF15844	complemer	similar to Saccharomy	orf19.4961	15844	IPF19731	UNCLASSI	molecular_function unknown
CA1373	1.2	1.6	1.2	AGP1	complemer	asparagine and glutam	orf19.8784	7152	IPF15844	TRANSCR	transcription regulator activity
CA1374	1.1	1.0	1.1	MIP1	complemer	Mitochondrial intermed	orf19.1195	7154	CaAGP1	Amino acid	transporter activity
CA1375	1.0	1.0	1.0	IPF7158	5903699..5	putative serine/threoni	orf19.8787	7158	CaMIP1	PROTEIN I	peptidase activity
CA1376	0.9	0.9	1.0	IPF7159	complemer	unknown function	orf19.8789	7159	IPF7158	CLASSIFIC	protein kinase activity
CA1377	1.1	1.0	1.0	IPF18690	complemer	unknown function	orf19.461	18690	IPF7159	UNCLASSI	molecular_function unknown
CA1378	1.0	1.0	1.0	ERK2	5912192..5	mitogen-activated prot	orf19.460	2568	IPF18690	No significant S.c. match	
CA1380	1.1	1.0	1.0	BCS1	complemer	mitochondrial protein c	orf19.458	2574	CaERK2	CELL CYCLE AND DNA PROCESSING REGULATION OF/INTERACTION WITH CELLULAR ENVIRONMENT CELL FATE SUBCELLUL	
CA1381	0.9	0.9	0.9	IPF8420	complemer	unknown function	orf19.929	8420	CaBCS1	PROTEIN I	hydrolase activity
CA1382	0.9	0.8	0.9	IPF8421	complemer	unknown function	orf19.928	8421	IPF8420	CELL RESCUE DEFENSE AND VIRULENCE ""TRANSPORT FACILITATION	
CA1383	1.0	0.9	1.0	IPF8422	5926369..5	unknown function	orf19.927	8422	IPF8421	No significant S.c. match	
CA1384	1.0	0.9	1.0	IPF8423	complemer	similar to Saccharomy	orf19.926	8423	IPF8422	UNCLASSI	molecular_function unknown
CA1385	0.9	0.8	1.0	IPF8424	complemer	unknown function	orf19.925	8424	IPF8423	Nucleotide	hydrolase activity,DNA binding
CA1386	1.0	1.0	1.0	THR1	complemer	homoserine kinase	orf19.923	8426	IPF8424	TRANSCRIPTION	
CA1387	1.0	1.0	1.0	ERG16	complemer	cytochrome P450 lano	orf19.922	8427	CaTHR1	Amino acid	transferase activity
CA1388	1.2	1.0	1.1	IPF16514	complemer	unknown function	orf19.921	16514	CaERG16	Lipid fatty-a	oxidoreductase activity
CA1389	1.0	1.0	1.0	PLP2	5939493..5	Might regulate Ste4p ir	orf19.9338	17476	IPF16514	No significant S.c. match	
CA1390	1.0	1.0	0.9	IPF17474	5940747..5	unknown function	orf19.1768	17474	CaPLP2	CELL FATE	enzyme regulator activity
CA1391	0.9	0.9	1.0	IPF6151	5942778..5	unknown function		6151	IPF17474	CELL CYCLE AND DNA PROCESSING ""PROTEIN FATE [folding modification destination] ""SUBCELLULAR LOCALISATION	
CA1392	0.9	0.9	0.9	IPF6149	5944903..5	similar to Saccharomy	orf19.1767	6149	IPF6151	CELL CYCLE AND DNA PROCESSING SUBCELLULAR LOCALISATION	
CA1393	1.0	1.0	1.0	IFO3	5948144..5	Similar to Streptomyce	orf19.1766	6146	IPF6149	PROTEIN I	peptidase activity
CA1394	1.0	1.1	1.0	IFO2	5952440..5	unknown function	orf19.9334	19560	CaIFO3	CELL FATE	
CA1395	0.9	1.0	1.0	IPF17558	5956966..5	unknown function	orf19.9332	17558	CaIFO2	UNCLASSIFIED PROTEINS	
CA1396	1.0	0.9	1.0	IPF8075	5961207..5	unknown function	orf19.3701	8075	IPF17558	SUBCELLULAR LOCALISATION	
CA1397	1.2	1.2	1.0	TOM72	5963697..5	mitochondrial import re	orf19.3700	8073	IPF8075	UNCLASSI	molecular_function unknown
CA1398	1.0	0.9	1.0	IPF19913	5965945..5	unknown function	orf19.3699	19913	CaTOM72	PROTEIN I	transporter activity
CA1399	1.0	0.9	1.1	IPF8069	complemer	unknown function	orf19.3698	8069	IPF19913	CELLULAF	hydrolase activity
CA1400	1.1	1.0	1.0	IPF8067	5968669..5	unknown function	orf19.3697	8067	IPF8069	UNCLASSI	molecular_function unknown
CA1401	1.1	1.1	1.0	TOM22	5971836..5	mitochondrial outer me	orf19.3696	8066	IPF8067	UNCLASSI	molecular_function unknown
CA1402	11.3	5.6	5.4	ECE1	5996321..5	Cell Elongation Proteir	orf19.3374	14152	CaTOM22	PROTEIN I	transporter activity
CA1403	0.9	0.8	0.9	IPF14155	5997783..5	similar to Saccharomy	orf19.3373	14155	CaECE1	Hypha-specific	No significant S.c. match
CA1404	1.0	0.9	0.9	IPF19554.5	5999907..6	unknown function, 5-pr	orf19.3372	19554	IPF14155	CELL CYC	RNA binding
CA1405	0.9	1.0	0.9	IPF19554.3	6000571..6	unknown function, 3-pr	orf19.3371	15627	IPF19554.5	UNCLASSI	peptidase activity
CA1406	0.9	0.9	1.0	DOT4	6002515..6	derepression of telome	orf19.3370	15624	IPF19554.3	No significant S.c. match	
CA1407	0.9	1.0	1.0	UAPC	6017274..6	purine permease (by h	orf19.2882	10516	CaDOT4	TRANSCR	peptidase activity
CA1408	1.0	1.0	1.0	IPF10513	6020500..6	unknown function	orf19.2883	10513	CaUAPC	No significant S.c. match	
CA1409	1.0	1.0	1.0	SPT16	complemer	general chromatin facto	orf19.2884	10512	IPF10513	No significant S.c. match	
CA1410	1.0	1.0	0.9	GAT1	6031344..6	nitrogen regulation (by	orf19.1275	12290	CaSPT16	CELL CYC	transcription regulator activity
CA1411	1.3	1.1	1.0	IPF11858	complemer	unknown function	orf19.1277	11858	CaGAT1	Nitrogen ar	transcription regulator activity
CA1412	1.1	1.0	1.1	IPF11854	complemer	unknown function	orf19.1278	11854	IPF11858	No significant S.c. match	
CA1413	1.2	1.1	1.1	MTR3	6040777..6	Involved in mRNA tran	orf19.168	16693	IPF11854	No significant S.c. match	
CA1414	1.0	1.0	0.9	CHO2	complemer	phosphatidylethanolam	orf19.169	19594	CaMTR3	TRANSCR	RNA binding
CA1415	1.2	1.2	1.0	DBP2.EXO	complemer	ATP-dependent RNA h	orf19.170	18681	CaCHO2	Lipid fatty-a	transferase activity
CA1416	1.0	1.0	1.0	DBP2.EXO	complemer	ATP-dependent RNA h	orf19.171	17362	CaDBP2.ex	TRANSCRIPTION SUBCELLULAR LOCALISATION	
CA1417	1.0	0.9	1.0	IPF16126	complemer	similar to Saccharomy	orf19.172	16126	CaDBP2.ex	TRANSCR	RNA binding,helicase activity
CA1418	0.9	1.0	1.0	IPF16124	complemer	zinc-finger containing p	orf19.173	16124	IPF16126	TRANSCR	nucleotidyltransferase activity
CA1419	0.7	0.5	1.0	IPF17296	complemer	unknown function	orf19.175	17296	IPF16124	TRANSCR	DNA binding
CA1420	1.1	1.0	1.1	RNT1	complemer	Ribonuclease III (by hc	orf19.1127	14250	IPF17296	CELL CYCLE AND DNA PROCESSING SUBCELLULAR LOCALISATION	
CA1421	1.1	1.0	1.1	MRPL11	6063038..6	Mitochondrial ribosoma	orf19.1127	14249	CaRNT1	TRANSCR	RNA binding
CA1422	0.9	0.9	0.9	IPF14248	6064228..6	putative methyltransfer	orf19.3798	14248	CaMRPL11	PROTEIN S	structural molecule activity
CA1423	1.1	1.2	1.0	IPF14247	complemer	unknown function	orf19.3799	14247	IPF14248	UNCLASSI	protein binding
CA1424	0.9	1.0	1.0	IPF9090	complemer	unknown function	orf19.1128	9090	IPF14247	SUBCELLU	molecular_function unknown

CA1426	1.0	0.8	1.0	ALS11.3F	6081393..6	agglutinin-like protein,	orf19.1316	9917	IPF9090	No significant S.c. match	
CA1427	1.0	0.9	1.0	IPF9914	6084925..6	alanyl-tRNA synthetas	orf19.5746	9914	CaALS11.3	SUBCELLULAR LOCALISATION Other virulence attributes	
CA1428	1.1	0.9	1.0	MRP4	complemer	Ribosomal protein of th	orf19.1317	15124	IPF9914	PROTEIN S	ligase activity
CA1429	1.0	1.0	1.1	IPF15123	6089555..6	unknown function	orf19.1317	15123	CaMRP4	PROTEIN S	RNA binding,structural molecule activity
CA1430	1.1	0.9	1.0	IPF14331	6096149..6	Probable extracellular	orf19.3380	14331	IPF15123	No significant S.c. match	
CA1431	4.7	5.6	2.1	FET34.3EC	6109997..6	iron transport multicop	orf19.1206	12467	IPF14331	No significant S.c. match	
CA1432	0.7	0.5	0.8	APM3	6111900..6	AP-3 complex subunit,	orf19.1204	12465	CaFET34.3	CELLULAR TRANSPORT AND TRANSPORT MECHANISMS REGULATION OF/INTERACTION WITH CELLULAR ENVIRONMENT SUB	
CA1433	0.9	1.0	1.0	IPF12464	complemer	unknown function		12464	CaAPM3	CELLULAR	molecular_function unknown
CA1434	1.0	1.0	1.0	SNI2	6114195..6	Sec9 interacting protei	orf19.1203	12463	IPF12464	No significant S.c. match	
CA1435	1.0	1.1	1.0	IPF7166	6117655..6	unknown function	orf19.1202	7166	CaSNI2	CELLULAR	molecular_function unknown
CA1436	0.9	1.0	1.1	IPF7165	complemer	similar to Saccharomy	orf19.1201	7165	IPF7166	CELLULAR TRANSPORT AND TRANSPORT MECHANISMS SUBCELLULAR LOCALISATION	
CA1437	1.0	0.9	0.9	IPF7163	6120582..6	unknown function	orf19.1200	7163	IPF7165	PROTEIN S	ligase activity
CA1438	1.0	1.2	0.9	NOP58	6122042..6	nucleolar protein requi	orf19.1199	7161	IPF7163	No significant S.c. match	
CA1439	1.1	1.1	1.2	IPF19602	complemer	similar to Saccharomy	orf19.4488	19602	CaNOP58	TRANSCR	molecular_function unknown
CA1440	2.3	1.6	1.3	RPL17B	6132795..6	RPL17B ribosomal pro	orf19.4490	4772	IPF19602	C-compour	transcription regulator activity
CA1441	1.0	1.1	1.0	QCR8	6134541..6	ubiquinol-cytochrome-c reductase		4774	CaRPL17B	PROTEIN S	structural molecule activity
CA1442	1.1	1.0	0.9	ERG20	complemer	farnesyl-pyrophosphat	orf19.4491	4775	CaQCR8	ENERGY S	transporter activity,oxidoreductase activity
CA1443	1.0	1.1	1.0	IPF4776	complemer	unknown Function	orf19.4492	4776	CaERG20	Lipid fatty-a	transferase activity
CA1444	1.0	1.0	0.9	KTR2	complemer	mannosyltransferase (	orf19.4494	4778	IPF4776	TRANSCR	molecular_function unknown
CA1445	1.0	1.0	1.0	MRP51	6142233..6	Mitochondrial ribosoma	orf19.185	13417	CaKTR2	C-compour	transferase activity
CA1446	1.0	0.9	1.0	YAL011	complemer	mitochondrial transit pe	orf19.190	15052	CaMRP51	PROTEIN S	structural molecule activity
CA1447	1.0	0.9	1.1	NRK1	complemer	Cdc31p-interacting ser	orf19.191	15055	CaYAL011	SUBCELLULAR LOCALISATION	
CA1448	1.0	1.1	1.1	IPF14728	complemer	unknown function	orf19.192	14728	CaNRK1	CELL CYCLE AND DNA PROCESSING CONTROL OF CELLULAR ORGANIZATION	
CA1449	1.0	1.0	1.1	IPF14730.5	6153650..6	unknown function, 5-pr	orf19.193	14730	IPF14728	No significant S.c. match	
CA1450	1.0	1.0	1.1	IPF6474	complemer	unknown function	orf19.4414	6474	IPF14730.5	No significant S.c. match	
CA1452	1.1	1.2	1.1	REV1	6158529..6	DNA repair protein (by	orf19.4412	6472	IPF6474	TRANSCRIPTION SUBCELLULAR LOCALISATION	
CA1453	1.0	1.0	1.1	HOS1	6162044..6	Putative histon deacety	orf19.4411	6469	CaREV1	CELL CYC	nucleotidyltransferase activity
CA1454	1.0	1.0	1.0	ALG1	complemer	beta-1,4-mannosyltran	orf19.4410	19918	CaHOS1	TRANSCR	hydrolase activity
CA1455	1.0	0.9	1.0	IPF15081	6165189..6	phophodiesterase (by	orf19.4409	15081	CaALG1	C-compour	transferase activity
CA1456	1.0	0.9	1.0	IPF11835.3	complemer	unknown function		15083	IPF15081	UNCLASSI	molecular_function unknown
CA1457	0.9	1.1	1.0	IPF8287	6171083..6	unknown function	orf19.612	8287	IPF11835.3	No significant S.c. match	
CA1458	5.2	1.5	1.7	IPF6342	6191478..6	unknown function	orf19.1106	6342	IPF8287	UNCLASSI	molecular_function unknown
CA1459	0.9	0.9	0.9	IPF6340	6194741..6	unknown function	orf19.1107	6340	IPF6342	No significant S.c. match	
CA1460	1.1	1.0	1.0	HAM1	complemer	Controls 6-N-hydroxyla	orf19.1108	6339	IPF6340	UNCLASSIFIED PROTEINS	
CA1461	1.0	1.0	1.0	IPF6338	complemer	unknown function	orf19.1109	6338	CaHAM1	CELL RES	molecular_function unknown
CA1462	1.0	0.9	0.9	THI80	complemer	Thiamin pyrophosphok	orf19.8707	6335	IPF6338	No significant S.c. match	
CA1464	0.9	0.9	0.8	PYC2.EXO	6201408..6	Pyruvate carboxylase 2	orf19.789	15167	CaTHI80	Metabolism	transferase activity
CA1465	1.0	1.0	1.0	RIM11	complemer	Ser/thr protein kinase (	orf19.791	18668	CaPYC2.ex	C-compour	ligase activity
CA1466	0.2	0.1	0.2	IPF13324	complemer	unknown function	orf19.8411	13324	CaRIM11	CELL CYCLE AND DNA PROCESSING TRANSCRIPTION SUBCELLULAR LOCALISATION	
CA1467	1.2	0.9	0.9	IPF5082	complemer	similar to Saccharomy	orf19.8412	5082	IPF13324	No significant S.c. match	
CA1468	1.0	1.0	1.0	SRB10	complemer	cyclin-dependent kinas	orf19.794	5080	IPF5082	CELL CYC	protein kinase activity
CA1469	0.9	1.0	1.0	IPF5078	complemer	unknown function	orf19.8414	5078	CaSRB10	C-compour	protein kinase activity,transcription regulator activity
CA1470	1.0	1.0	1.1	IPF17942	6214621..6	unknown function	orf19.8415	17942	IPF5078	PROTEIN I	signal transducer activity
CA1471	0.9	0.9	1.1	ALS2.3FEC	complemer	agglutinin-like protein,	orf19.1098	15450	IPF17942	CELL FATE	transcription regulator activity
CA1474	0.9	1.0	1.0	IPF10208	complemer	chloride channel-like p	orf19.1096	10208	CaALS2.3f	SUBCELLULAR LOCALISATION Other virulence attributes	
CA1475	0.9	0.9	0.9	GLE2	6231541..6	nuclear pore complex s	orf19.1095	10209	IPF10208	CELLULAR TRANSPORT AND TRANSPORT MECHANISMS REGULATION OF/INTERACTION WITH CELLULAR ENVIRONMENT SUB	
CA1476	1.0	1.0	1.0	IPF10214	6235149..6	unknown function	orf19.1093	10214	CaGLE2	TRANSCR	structural molecule activity
CA1477	0.5	1.0	0.4	YME1	complemer	family of ATPases	orf19.1252	14644	IPF10214	No significant S.c. match	
CA1478	1.0	0.9	1.0	IPF16426	6245915..6	similar to human BRRN	orf19.1251	16426	CaYME1	PROTEIN I	peptidase activity
CA1479	1.0	0.9	1.0	IPF16428	complemer	unknown function	orf19.1250	16428	IPF16426	CELL CYC	molecular_function unknown
CA1480	1.0	0.9	0.9	HIS6	6249378..6	5 Pro-FAR isomerase	orf19.1249	15577	IPF16428	UNCLASSI	molecular_function unknown
CA1481	1.0	1.1	1.0	RPB3	complemer	DNA-directed RNA-pol	orf19.8832	15576	CaHIS6	Amino acid	isomerase activity
CA1482	1.0	0.9	1.0	IPF15575	6251477..6	unknown function	orf19.8831	15575	CaRPB3	TRANSCR	nucleotidyltransferase activity
CA1483	1.0	1.0	1.0	YHC3	complemer	involved in cellular pH	orf19.4059	10526	IPF15575	No significant S.c. match	
CA1484	1.0	1.0	1.0	ARO4	6259877..6	3-dehydro-deoxyphosp	orf19.4060	10524	CaYHC3	CLASSIFIC	molecular_function unknown
CA1485	1.0	1.0	1.1	IPF20082	complemer	unknown function	orf19.4061	20082	CaARO4	Amino acid	transferase activity
CA1486	1.0	1.0	1.1	IPF10521	complemer	unknown function	orf19.4062	10521	IPF20082	UNCLASSI	molecular_function unknown
CA1487	0.9	0.9	1.0	GPT1	complemer	polyamine transporter	orf19.4063	10519	IPF10521	No significant S.c. match	
CA1488	0.9	1.0	0.9	IPF17402	6267628..6	unknown function	orf19.4064	17402	CaGPT1	No significant S.c. match	
CA1489	1.2	1.6	1.0	ATP3.3	complemer	F1F0-ATPase complex	orf19.1073	163	IPF17402	SUBCELLU	transferase activity
CA1490	0.9	1.0	0.9	IPF165	complemer	unknown function		165	CaATP3.3	ENERGY C	transporter activity
CA1491	1.1	1.1	1.1	IPF166	6273822..6	unknown function	orf19.3225	166	IPF165	No significant S.c. match	
CA1492	1.2	1.0	1.2	IPF168	6277773..6	unknown function	orf19.3226	168	IPF166	TRANSPO	molecular_function unknown
CA1493	1.1	1.0	1.0	FTH2	6279115..6	iron transporter (by ho	orf19.3227	170	IPF168	UNCLASSI	molecular_function unknown
CA1494	1.2	1.1	1.2	IPF171	complemer	unknown function	orf19.3228	171	CaFTH2	REGULATION OF/INTERACTION WITH CELLULAR ENVIRONMENT TRANSPORT FACILITATION Other virulence attributes	
CA1495	1.0	1.0	1.0	IPF9686	6289847..6	similar to Saccharomy	orf19.3647	9686	IPF171	UNCLASSI	molecular_function unknown
CA1496	1.7	1.6	2.1	CTR1	complemer	copper transport protei	orf19.3646	9685	IPF9686	CELLULAR	protein binding
CA1497	0.9	1.0	1.0	IPF9683	complemer	unknown function	orf19.3644	9683	CaCTR1	CELLULAR	transporter activity
CA1498	0.9	0.9	1.0	IPF12371	complemer	extracellular alpha-1,4-	orf19.3643	12371	IPF9683	TRANSCRIPTION	
CA1499	0.8	0.8	1.0	IPF6521.3E	6303710..6	similar to Saccharomyces cerevisi		13224	IPF12371	TRANSCRIPTION	
CA1500	0.8	0.9	0.8	IPF13221	6308626..6	unknown function	orf19.1697	13221	IPF6521.3e	TRANSCRIPTION SUBCELLULAR LOCALISATION	
CA1501	1.0	1.0	1.0	IPF13217	complemer	unknown function	orf19.9265	13217	IPF13221	UNCLASSI	molecular_function unknown

CA1502	0.4	0.7	0.6	RPS7A	complemer	ribosomal protein (by h	orf19.9267	13662	IPF13217	UNCLASSI	molecular_function unknown
CA1503	1.0	1.0	1.0	RKI1	6315794..6	D-ribose-5-phosphate	orf19.9268	13663	CaRPS7A	PROTEIN S	structural molecule activity
CA1504	1.4	1.3	1.3	ARF3	complemer	GTP-binding protein of	orf19.1702	13664	CaRKI1	C-compour	isomerase activity
CA1505	1.0	0.9	1.0	POT1	6319848..6	Acetyl-CoA C-acyltrans	orf19.9271	17552	CaARF3	PROTEIN FATE [folding modification destination] ""CELLULAR TRANSPORT AND TRANSPORT MECHANISMS	
CA1506	0.9	1.1	0.9	HGT11	6323821..6	hexose transporter	orf19.4527	9983	CaPOT1	Lipid fatty-acid and isoprenoid metabolism ""ENERGY SUBCELLULAR LOCALISATION	
CA1507	1.1	1.1	1.0	HSP30	6328488..6	heat shock protein (by	orf19.4526	9978	CaHGT11	C-compour	molecular_function unknown
CA1508	1.0	0.8	1.1	IPF9977	6329966..6	unknown function	orf19.4525	9977	CaHSP30	CELL RES	chaperone activity
CA1509	0.9	0.9	0.9	IPF19920.3	complemer	unknown function, 3-pr	orf19.4524	19920	IPF9977	UNCLASSI	molecular_function unknown
CA1510	0.9	1.0	0.9	IPF9973	6333605..6	similar to Saccharomy	orf19.4523	9973	IPF19920.3	Amino acid metabolism TRANSCRIPTION SUBCELLULAR LOCALISATION	
CA1511	1.0	1.0	1.0	IPF9972.3	complemer	unknown function, 3-pr	orf19.4522	9972	IPF9973	Metabolism	ligase activity
CA1512	1.0	1.0	1.0	IPF15255	6341070..6	unknown function	orf19.4884	15255	IPF9972.3	ENERGY	molecular_function unknown
CA1513	1.1	1.4	1.1	MIR1	6346913..6	phosphate transport pr	orf19.4885	7536	IPF15255	CLASSIFIC	molecular_function unknown
CA1514	1.0	0.8	1.1	IPF7539	6349709..6	unknown function	orf19.4886	7539	CaMIR1	Phosphate	transporter activity
CA1515	0.7	0.5	0.8	ECM21.3	complemer	Involved in cell wall bio	orf19.1235	18663	IPF7539	No significant S.c. match	
CA1516	1.0	1.0	1.0	IPF16764	6355294..6	unknown function	orf19.4796	16764	CaECM21.	CONTROL	molecular_function unknown
CA1517	1.0	1.3	0.9	IPF11045	complemer	unknown function	orf19.4798	11045	IPF16764	PROTEIN SYNTHESIS SUBCELLULAR LOCALISATION	
CA1518	1.0	1.0	1.1	IPF11040	6360312..6	similar to Schizosacch	orf19.4799	11040	IPF11045	UNCLASSI	molecular_function unknown
CA1519	1.0	1.1	1.0	RIM20	6362353..6	Rim101 activating prot	orf19.4800	11037	IPF11040	CELL CYC	RNA binding
CA1520	1.0	1.0	1.0	IPF11035	6364914..6	similar to Saccharomy	orf19.4801	11035	CaRIM20	UNCLASSI	molecular_function unknown
CA1521	1.0	1.0	1.1	FTH1	complemer	iron transporter	orf19.4802	11034	IPF11035	TRANSCR	transferase activity
CA1522	1.0	0.9	1.0	IPF6916	complemer	unknown function	orf19.3791	6916	CaFTH1	REGULATI	molecular_function unknown
CA1523	1.0	1.0	1.0	PAT1	6372742..6	Topoisomerase II-assc	orf19.3792	6915	IPF6916	No significant S.c. match	
CA1524	1.0	1.1	1.1	SUR1	6380868..6	Suppressor of ROK1	orf19.3794	6909	CaPAT1	CELL CYC	molecular_function unknown
CA1525	1.0	0.9	1.0	AGP3	6383816..6	amino acid-permease	orf19.3795	6906	CaSUR1	TRANSCR	DNA binding
CA1526	1.0	1.3	1.0	IPF13815	6398814..6	unknown function	orf19.4553	13815	CaAGP3	Amino acid	transporter activity
CA1528	1.0	1.2	1.1	ALS4.3F	6408890..6	agglutinin-like protein,	orf19.4556	13009	IPF13815	No significant S.c. match	
CA1529	1.0	1.0	1.0	SPC105	6411436..6	Spindle pole body prot	orf19.4557	15446	CaALS4.3f	C-compound and carbohydrate metabolism SUBCELLULAR LOCALISATION Other virulence attributes	
CA1530	1.1	1.0	1.0	ALG2.5	6418468..6	mannosyltransferase, S	orf19.1221	9503	CaSPC105	SUBCELLU	structural molecule activity
CA1531	1.1	1.3	1.0	RVS167	complemer	(putative) cytoskeletal	orf19.1220	9505	CaALG2.5	C-compour	transferase activity
CA1532	0.9	0.9	0.8	IPF9507	6421659..6	unknown function	orf19.8806	9507	CaRVS167	CELL FATE	protein binding
CA1533	1.0	0.9	0.9	IPF9510	complemer	unknown function	orf19.8803	9510	IPF9507	UNCLASSI	molecular_function unknown
CA1534	0.9	1.0	1.1	MAP2	6428609..6	methionine aminopepti	orf19.1214	9511	IPF9510	No significant S.c. match	
CA1535	1.0	1.0	1.0	IPF9515	6432481..6	similar to Saccharomy	orf19.8800	9515	CaMAP2	PROTEIN S	peptidase activity
CA1536	1.0	1.1	1.0	SOK1	6443209..6	high copy suppressor c	orf19.451	13595	IPF9515	C-compour	protein binding
CA1537	1.0	1.0	1.0	IPF17483	complemer	unknown function	orf19.450	17483	CaSOK1	TRANSCR	molecular_function unknown
CA1538	1.1	1.2	1.1	IDI1.3EOC	complemer	Isopentenyl-diphospha	orf19.4558	1053	IPF17483	No significant S.c. match	
CA1539	1.0	1.0	1.0	BFR1	6456504..6	Similar to Saccharomy	orf19.4560	1051	CaIDI1.3ec	Lipid fatty-acid and isoprenoid metabolism ""SUBCELLULAR LOCALISATION	
CA1540	1.0	0.9	1.1	IPF1047	6458249..6	unknown function	orf19.4563	1047	CaBFR1	CELL CYC	RNA binding
CA1541	1.2	1.1	1.1	BGL21	6460091..6	endo-beta-1,3-glucana	orf19.4565	1046	IPF1047	UNCLASSI	molecular_function unknown
CA1542	1.0	1.1	1.0	ROT11	6461381..6	Putative membrane pro	orf19.4566	1044	CaBGL21	C-compour	hydrolase activity
CA1543	1.1	1.1	1.0	IPF1043	6462725..6	Similar to hydroxyquin	orf19.4567	1043	CaROT11	CELL CYCLE AND DNA PROCESSING	
CA1544	0.9	0.8	0.9	IPF1040	6464496..6	Similar to transcription	orf19.4568	1040	IPF1043	No significant S.c. match	
CA1545	0.9	0.8	1.0	CDR3.5EO	complemer	ABC transporter, multi	orf19.1313	14709	IPF1040	C-compound and carbohydrate metabolism TRANSCRIPTION SUBCELLULAR LOCALISATION	
CA1546	0.9	1.1	1.0	IPF6671	6475739..6	unknown function	orf19.8891	6671	CaCDR3.5	Lipid fatty-acid and isoprenoid metabolism """"CELL RESCUE DEFENSE AND VIRULENCE ""REGULATION OF/INTERACTION WITH CE	
CA1547				IPF6672	complement(6478315..6480216)				IPF6671	UNCLASSI	molecular_function unknown
CA1548	1.5	1.4	1.4	IPF8746	6485521..6	putative alpha-1,3-mar	orf19.4279	8746			
CA1549	1.1	1.0	1.0	IPF8744	6488638..6	unknown function	orf19.4278	8744	IPF8746	C-compound and carbohydrate metabolism ""PROTEIN FATE [folding modification destination] ""SUBCELLULAR LOCALISATION	
CA1550	0.9	0.9	1.0	IPF8741.5f	6491170..6	unknown function, 5-pr	orf19.4276	8742	IPF8744	TRANSCR	molecular_function unknown
CA1551	1.0	0.9	1.0	IPF8741.3f	6492327..6	unknown function, 3-pr	orf19.4275	8741	IPF8741.5f	No significant S.c. match	
CA1552	0.5	0.5	0.4	PUT1	complemer	proline oxidase (by hor	orf19.4274	8739	IPF8741.3f	CELL CYC	protein binding
CA1553	1.0	1.0	1.0	IPF14081	complemer	unknown function	orf19.1714	14081	CaPUT1	Amino acid	oxidoreductase activity
CA1554	1.0	1.0	1.1	GEA2.3F	complemer	GTP/GDP exchange fa	orf19.1713	14077	IPF14081	No significant S.c. match	
CA1556	1.0	1.1	0.9	END3	6505531..6	required for endocytos	orf19.1711	15676	CaGEA2.3f	CELLULAR	enzyme regulator activity
CA1557	1.0	1.0	0.9	IPF15677	complemer	probable NADH-ubiqui	orf19.1710	15677	CaEND3	CELLULAR	protein binding
CA1558	0.6	0.4	0.6	IPF15679	complemer	lipid transfer protein (b	orf19.1709	15679	IPF15677	No significant S.c. match	
CA1559	0.8	0.7	0.8	IPF16533	6510839..6	unknown function	orf19.1708	16533	IPF15679	No significant S.c. match	
CA1560	0.9	0.9	0.9	MET18	complemer	Involved in NER repair	orf19.1706	16088	IPF16533	No significant S.c. match	
CA1561	1.0	0.9	0.9	POT12	complemer	peroxysomal 3-ketoac	orf19.1704	16087	CaMET18	CELL CYC	transcription regulator activity
CA1563	0.7	0.4	0.9	IPF13855	6535766..6	unknown function	orf19.4713	13855	CaPOT12	Lipid fatty-acid and isoprenoid metabolism ""ENERGY SUBCELLULAR LOCALISATION	
CA1564	1.9	3.0	2.6	GAD1	complemer	Glutamate decarboxyla	orf19.1153	7609	IPF13855	No significant S.c. match	
CA1565	1.0	1.3	1.1	EGD1	complemer	GAL4 DNA-binding enl	orf19.1154	7610	CaGAD1	Amino acid	lyase activity
CA1566	0.8	0.7	1.0	DPP2	6543266..6	Diacylglycerol pyropho	orf19.1155	7611	CaEGD1	C-compour	chaperone activity
CA1567	0.9	0.9	1.0	IPF7613	6544566..6	unknown function	orf19.1156	7613	CaDPP2	Lipid fatty-acid and isoprenoid metabolism ""CELL CYCLE AND DNA PROCESSING CELL FATE	
CA1568	1.0	1.0	0.9	IPF7615	complemer	unknown function	orf19.1158	7615	IPF7613	CELL RESCUE DEFENSE AND VIRULENCE ""SUBCELLULAR LOCALISATION	
CA1569	1.0	1.1	1.0	IPF7616	6546850..6	putative homoserine O	orf19.1159	7616	IPF7615	UNCLASSIFIED PROTEINS	
CA1570	1.0	1.0	1.0	IPF7617	complemer	unknown function	orf19.1160	7617	IPF7616	Amino acid metabolism SUBCELLULAR LOCALISATION	
CA1571	1.0	1.1	1.0	SPO14.3E	complemer	phospholipase D, 3-prime end		7619	IPF7617	UNCLASSI	molecular_function unknown
CA1572	0.4	0.6	0.4	POX4	6553045..6	peroxisomal fatty acyl-	orf19.9221	15088	CaSPO14.3	Lipid fatty-acid and isoprenoid metabolism ""CELL CYCLE AND DNA PROCESSING CELL FATE	
CA1573	1.0	1.0	0.9	IPF15087	6555631..6	unknown function	orf19.1653	15087	CaPOX4	Lipid fatty-a	oxidoreductase activity
CA1574	0.5	0.7	0.4	PXP2	complemer	acyl-CoA oxidase pero	orf19.1655	13474	IPF15087	UNCLASSIFIED PROTEINS	
CA1576	2.0	1.5	5.5	HYR1	6570537..6	hyphally regulated prot	orf19.1244	19734	CaPXP2	Lipid fatty-acid and isoprenoid metabolism ""ENERGY SUBCELLULAR LOCALISATION	
CA1577	0.9	0.9	0.9	KNS1	6578007..6	Ser/thr protein kinase (	orf19.1244	11410	CaHYR1	Hypha-specific	No significant S.c. match

CA1578	1.0	0.9	0.9	NUM11	complemer	nuclear migration prote	orf19.4715	8243	CaKNS1	CLASSIFIC	protein kinase activity
CA1579	1.0	1.0	1.1	GDH3	complemer	NADP-glutamate dehy	orf19.4716	8236	CaNUM11	CELL CYC	protein binding
CA1580	0.9	1.0	1.0	TRP5	6591781..6	tryptophan synthase (b	orf19.4718	8232	CaGDH3	Amino acid	oxidoreductase activity
CA1581	1.0	0.8	0.9	CWH41.3E	complemer	ER glucosidase I, 3-pri	orf19.4719	8229	CaTRP5	Amino acid	lyase activity
CA1582	1.1	1.0	1.5	CLN21	6600806..6	G1 cyclin (by homolog	orf19.6028	15332	CaCWH41.	C-compour	hydrolase activity
CA1583	1.0	1.1	1.0	ROT1	6605304..6	Suppressor of TOR2 m	orf19.6029	18655	CaCLN21	CELL CYC	protein kinase activity,enzyme regulator activity
CA1584	1.0	1.0	1.0	IPF15824	complemer	unknown function	orf19.6030	15824	CaROT1	CELL CYC	molecular_function unknown
CA1585	0.8	0.8	0.9	VPS27	6607805..6	Vacuolar protein sortin	orf19.6031	15825	IPF15824	No significant S.c. match	
CA1586	1.0	1.0	1.0	ODC1	6611062..6	Ornithine decarboxylas	orf19.6032	1466	CaVPS27	PROTEIN I	protein binding
CA1587	0.5	0.4	1.0	CMP2	6613123..6	Calcineurin B, catalytic	orf19.6033	1465	CaODC1	Secondary	lyase activity
CA1588	0.9	1.0	1.0	SPT8	6619516..6	transcriptional adaptor	orf19.1178	16990	CaCMP2	TRANSCR	protein phosphatase activity
CA1589	0.9	0.9	0.9	IPF16988	complemer	unknown function	orf19.1178	16988	CaSPT8	TRANSCR	transcription regulator activity
CA1590	1.0	0.8	1.0	IPF14665	complemer	unknown function	orf19.1179	14665	IPF16988	No significant S.c. match	
CA1591	1.0	0.9	1.0	IPF14663	6625398..6	unknown function	orf19.1179	14663	IPF14665	UNCLASSI	enzyme regulator activity
CA1592	1.1	1.1	1.1	IPF14662	complemer	D-xylose reductase (by	orf19.4317	14662	IPF14663	UNCLASSIFIED PROTEINS	
CA1593	0.9	0.9	0.9	MIG1	complemer	transcriptional regulato	orf19.4318	11048	IPF14662	C-compour	oxidoreductase activity
CA1594	1.0	0.9	1.0	IPF11051	complemer	unknown function	orf19.4321	11051	CaMIG1	C-compour	transcription regulator activity
CA1595	0.9	0.9	1.0	IPF9544	6636402..6	unknown function	orf19.7905	9544	IPF11051	No significant S.c. match	
CA1596	0.9	1.0	1.0	FAA21	6640140..6	long-chain-fatty-acid-C	orf19.272	9539	IPF9544	UNCLASSI	molecular_function unknown
CA1597	1.0	0.9	0.9	IPF9538	complemer	unknown function	orf19.271	9538	CaFAA21	Lipid fatty-a	ligase activity
CA1598	0.9	1.0	1.0	SES1	complemer	seryl-tRNA synthetase	orf19.7901	9534	IPF9538	ENERGY SUBCELLULAR LOCALISATION	
CA1599	1.0	0.9	0.9	IPF11452	6646907..6	unknown function	orf19.7900	11452	CaSES1	No significa	ligase activity
CA1600	1.0	1.2	1.1	CTR2	6650213..6	copper transport protei	orf19.4720	15729	IPF11452	CELL CYC	molecular_function unknown
CA1601	0.9	0.8	0.9	IPF15728	6651071..6	unknown function	orf19.4721	15728	CaCTR2	CELLULAF	transporter activity
CA1602	1.0	1.0	1.1	RTG1	6652812..6	basic helix-loop-helix tı	orf19.4722	13658	IPF15728	No significant S.c. match	
CA1603	0.9	0.9	0.9	FAD1	complemer	flavin adenine dinuclec	orf19.4723	13656	CaRTG1	C-compour	transcription regulator activity
CA1604	1.0	1.0	1.0	IPF13653	complemer	unknown function	orf19.4724	13653	CaFAD1	Metabolisr	nucleotidyltransferase activity
CA1605	1.1	1.1	1.0	SWI6	6656250..6	Transcription factor (by	orf19.4725	17885	IPF13653	TRANSCR	RNA binding
CA1606	0.9	0.9	0.9	NCS1	complemer	Calcium binding protei	orf19.4726	16484	CaSWI6	CELL CYC	protein binding
CA1607	1.1	1.0	0.9	IPF14916	6659506..6	unknown function	orf19.4727	16483	CaNCS1	CELLULAR COMMUNICATION/SIGNAL TRANSDUCTION MECHANISM	
CA1608	0.9	0.9	1.0	TES12	complemer	Thiosterase (by homol	orf19.4122	5877	IPF14916	UNCLASSI	molecular_function unknown
CA1609	0.9	0.9	1.0	TES11	complemer	Thiosterase (by homol	orf19.4121	5879	CaTES12	Lipid fatty-a	hydrolase activity
CA1610	1.1	1.1	1.1	LAS1	6667577..6	cell morphogenesis, cy	orf19.4120	5882	CaTES11	Lipid fatty-acid and isoprenoid metabolism ""SUBCELLULAR LOCALISATION	
CA1611	0.7	0.8	0.6	SPO72	6669692..6	required for sporulatior	orf19.4119	12198	CaLAS1	CELL CYC	molecular_function unknown
CA1612	1.0	1.0	1.0	IFJ6	complemer	unknown function	orf19.3214	12814	CaSPO72	CELL CYC	molecular_function unknown
CA1613	1.1	1.0	1.1	IPF19735	complemer	unknown function	orf19.3215	19735	CaIFJ6	No significant S.c. match	
CA1614	0.9	1.0	1.1	IPF18645	complemer	unknown function	orf19.1072	18645	IPF19735	UNCLASSIFIED PROTEINS	
CA1615	0.9	0.9	1.0	IPF14540	6689569..6	putative multidrug prot	orf19.3218	14540	IPF18645	UNCLASSIFIED PROTEINS	
CA1616	1.1	1.1	1.1	IPF14538	6693378..6	unknown function	orf19.3219	14538	IPF14540	TRANSPO	molecular_function unknown
CA1617	1.0	0.9	0.9	IPF14536	6695614..6	unknown function	orf19.3220	14536	IPF14538	UNCLASSIFIED PROTEINS	
CA1618	1.2	1.2	1.1	RRP45	complemer	Protein component of t	orf19.1155	2068	IPF14536	UNCLASSI	molecular_function unknown
CA1619	1.1	1.1	1.1	IPF2067	complemer	Required for mannosyl	orf19.1155	2067	CaRRP45	TRANSCR	RNA binding
CA1620	1.0	1.0	1.1	MET10	6704455..6	Sulfite reductase flavin	orf19.4076	13194	IPF2067	Lipid fatty-a	transferase activity
CA1621	1.0	0.9	1.0	IFF6	6711242..6	unknown function	orf19.1155	19647	CaMET10	Amino acid	transporter activity
CA1622	1.0	1.1	1.0	IPF10045	6715191..6	similar to Saccharomy	orf19.470	10045	CaIFF6	No significant S.c. match	
CA1623	0.8	0.6	0.8	STE7	complemer	MAP Kinase Kinase	orf19.469	10048	IPF10045	TRANSCR	transcription regulator activity
CA1624	1.9	2.4	1.3	IPF10055	complemer	unknown function	orf19.467	10055	CaSTE7	REGULATI	protein kinase activity
CA1625	1.0	1.0	0.9	IPF6156	6732538..6	similar to C.elegans LI	orf19.1034	6156	IPF10055	No significant S.c. match	
CA1626	1.0	1.0	1.0	STR2	complemer	O-succinylhomoserine	orf19.1033	6155	IPF6156	UNCLASSIFIED PROTEINS	
CA1627	0.9	0.9	0.9	SKO1.3	6736495..6	Cre-binding bzip protei	orf19.1032	6153	CaSTR2	Amino acid	transferase activity
CA1628	1.0	1.0	1.0	HMG1	complemer	3-hydroxy-3-methylglu	orf19.1031	19736	CaSKO1.3	TRANSCR	transcription regulator activity
CA1629	1.0	1.1	0.9	NPI46	complemer	proline cis-trans isome	orf19.1030	14314	CaHMG1	Lipid fatty-a	oxidoreductase activity
CA1630	0.9	0.9	0.9	RPP1	6745236..6	required for processing	orf19.1029	14315	CaNPI46	PROTEIN I	isomerase activity
CA1631	1.0	1.0	0.9	IPF19582	complemer	unknown function	orf19.1028	19582	CaRPP1	TRANSCR	RNA binding
CA1632	1.0	1.0	1.0	IPF10181	6750184..6	similar to Saccharomy	orf19.1142	10823	IPF19582	UNCLASSI	transcription regulator activity
CA1633	0.7	0.7	1.0	IPF10180	complemer	unknown function	orf19.1142	10822	IPF10181	UNCLASSI	structural molecule activity
CA1634	0.9	1.0	1.0	IPF10179	6752178..6	unknown function	orf19.1142	10820	IPF10180	No significant S.c. match	
CA1635	1.0	1.0	0.9	URA7	complemer	CTP synthase 1 (by hc	orf19.3941	10815	IPF10179	UNCLASSI	molecular_function unknown
CA1636	1.0	1.0	1.0	IPF20086	complemer	unknown function	orf19.3942	20086	CaURA7	Nucleotide	ligase activity
CA1637	2.2	1.9	1.4	RPL43A.3	complemer	ribosomal protein, 3-prime end (by	orf19.3943	20087	IPF20086	UNCLASSI	molecular_function unknown
CA1638	0.9	1.0	0.8	GRR1	complemer	Required for glucose r	orf19.3944	14608	CaRPL43A	PROTEIN S	structural molecule activity
CA1639	1.0	0.9	1.1	IPF18641.E	6763994..6	unknown function, exo	orf19.1733	18641	CaGRR1	C-compour	protein binding
CA1640	1.0	0.9	0.9	IPF18641.E	6765639..6	unknown function, exo	orf19.1732	18640	IPF18641.e	UNCLASSIFIED PROTEINS	
CA1641	1.1	1.2	1.1	NHP10.3F	complemer	unknown function, 3-pr	orf19.1731	9350	IPF18641.e	UNCLASSIFIED PROTEINS	
CA1642	1.1	1.1	1.0	NHP1.5F	complemer	unknown function, 5-pr	orf19.1730	9349	CaNHP10.3	CLASSIFIC	molecular_function unknown
CA1643	1.0	1.0	1.0	IPF9347	6769829..6	unknown function	orf19.1729	9347	CaNHP1.5f	CLASSIFICATION NOT YET CLEAR-CUT	
CA1644	1.1	1.0	0.9	IPF9345	complemer	unknown function	orf19.1728	9345	IPF9347	No significant S.c. match	
CA1645	0.8	0.8	0.6	PMC1	6774913..6	Ca2+-transporting P-ty	orf19.1727	9340	IPF9345	No significant S.c. match	
CA1646	1.0	1.3	1.0	IPF9336.3E	complemer	unknown function, 3-pr	orf19.1725	9336	CaPMC1	CELLULAF	transporter activity
CA1647	1.0	1.0	0.9	IPF16663	6783732..6	unknown function	orf19.5568	16663	IPF9336.3e	No significant S.c. match	
CA1648	1.0	1.1	1.0	IPF16662	complemer	unknown function	orf19.5567	16662	IPF16663	UNCLASSI	protein binding
CA1649	1.0	1.0	1.0	LAB1	6785702..6	Lipoate biosynthesis b	orf19.5566	5392	IPF16662	TRANSCR	RNA binding

CA1650	1.1	1.0	1.0	IPF5389	6788276..6	3-hydroxyisobutyrate d	orf19.5565	5389	CaLAB1	UNCLASSI	molecular_function unknown
CA1651	0.8	0.9	0.9	RNH1.EXC	complemer	Ribonuclease H, exon	orf19.5564	18639	IPF5389	No significant S.c. match	
CA1652	1.0	1.1	1.0	RNH1.EXC	complemer	Ribonuclease H, exon	orf19.5563	5387	CaRNH1.e	Nucleotide metabolism CONTROL OF CELLULAR ORGANIZATION	
CA1653	1.1	1.2	1.1	STE23	complemer	protease involved in a-	orf19.5561	5385	CaRNH1.e	Nucleotide	RNA binding
CA1654	1.1	1.1	1.0	IPF19660	complemer	unknown function	orf19.5559	19661	CaSTE23	PROTEIN I	peptidase activity
CA1655	1.1	1.0	1.0	CCC2	complemer	putative copper-transp	orf19.4328	12705	IPF19660	UNCLASSI	molecular_function unknown
CA1656	0.9	1.1	0.9	IPF16019	complemer	unknown function	orf19.4326	16019	CaCCC2	REGULATI	transporter activity
CA1657	0.9	0.9	1.0	IPF16022	6810308..6	unknown function	orf19.4325	16022	IPF16019	TRANSCR	RNA binding
CA1658	1.0	1.0	1.0	IPF19924	complemer	unknown function	orf19.4324	19924	IPF16022	UNCLASSIFIED PROTEINS	
CA1659	0.9	1.0	1.0	IPF11054	6814609..6	unknown function	orf19.4323	11054	IPF19924	No significant S.c. match	
CA1660	1.0	1.1	1.0	DAP2	complemer	dipeptidyl aminopeptid	orf19.4322	11053	IPF11054	UNCLASSI	molecular_function unknown
CA1662	1.1	0.9	1.0	RPL28.3F	6820892..6	Ribosomal protein, 3-prime end (b		7308	CaDAP2	PROTEIN I	peptidase activity
CA1663	0.6	0.3	1.1	IPF7309	6821859..6	unknown function	orf19.2864	7309	CaRPL28.3	PROTEIN S	RNA binding
CA1664	0.9	1.0	1.0	ERV1.3	6823051..6	Mitochondrial biogenesis and regu		7312	IPF7309	UNCLASSI	molecular_function unknown
CA1665	1.0	1.0	1.0	RIB1	6826003..6	GTP cyclohydrolase II	orf19.2862	18635	CaERV1.3	CELL CYC	oxidoreductase activity
CA1666	1.0	1.0	1.0	SRP40	6827174..6	RNA I and II supressor	orf19.2859	19740	CaRIB1	Metabolism	hydrolase activity
CA1667	1.0	1.0	0.9	SSL2	6828822..6	by homology to S. cere	orf19.2857	11402	CaSRP40	TRANSCR	chaperone activity
CA1668	1.0	1.0	1.0	IPF11396	complemer	unknown function	orf19.2853	11396	CaSSL2	CELL CYC	DNA binding,helicase activity
CA1669	1.0	1.1	1.0	IPF11393	6832844..6	unknown function	orf19.2852	11393	IPF11396	UNCLASSIFIED PROTEINS	
CA1670	1.0	0.9	1.0	IPF11392	6834063..6	unknown function	orf19.2851	11392	IPF11393	PROTEIN S	structural molecule activity
CA1671	1.0	1.0	1.0	IPF11391	complemer	unknown function		11391	IPF11392	Amino acid metabolism CELLULAR TRANSPORT AND TRANSPORT MECHANISMS SUBCELLULAR LOCALISATION TRANSPORT FA	
CA1672	1.3	1.2	1.0	IPF5196	complemer	unknown function	orf19.1109	5196	IPF11391	No significant S.c. match	
CA1673	3.2	2.0	1.5	PST2	6840216..6	1,4-benzoquinone redu	orf19.3612	5195	IPF5196	No significant S.c. match	
CA1674	1.1	1.2	1.1	PAF1	6841165..6	DNA-directed RNA pol	orf19.3613	5194	CaPST2	UNCLASSI	molecular_function unknown
CA1675	0.9	0.9	0.9	IPF5192	complemer	unknown function	orf19.3615	5192	CaPAF1	TRANSCR	transcription regulator activity
CA1676	1.0	1.1	1.1	ERG9	complemer	farnesyl-diphosphate fa	orf19.3616	5191	IPF5192	UNCLASSI	molecular_function unknown
CA1677	1.0	1.0	0.9	GTR1	complemer	GTP-binding protein by	orf19.3617	5189	CaERG9	Lipid fatty-a	transferase activity
CA1678	1.2	1.0	1.2	IPF5185	complemer	putative cell wall protei	orf19.3618	5185	CaGTR1	Phosphate	hydrolase activity
CA1679	1.2	1.0	1.2	IPF5180.3E	complemer	unknown function, 3-prime end		18631	IPF5185	SUBCELLULAR LOCALISATION	
CA1680	1.1	0.9	1.0	IPF15649	complemer	unknown function	orf19.4913	15649	IPF5180.3e	No significant S.c. match	
CA1681	0.2	0.0	0.9	IPF12676	6863796..6	unknown function	orf19.4914	12676	IPF15649	UNCLASSI	molecular_function unknown
CA1682	0.9	0.9	0.8	CIRT3	complemer	Putative transposase	orf19.4918	18629	IPF12676	No significa	molecular_function unknown
CA1683	1.2	1.1	1.1	CIRT	6871560..6	Probable transposase	orf19.4919	18628	CaCirt3	No significant S.c. match	
CA1684	1.0	1.0	1.0	IPF6070	6873866..6	unknown function	orf19.4921	6070	CaCirt	No significant S.c. match	
CA1685	1.0	1.0	1.0	IPF12270	6878314..6	unknown function	orf19.3660	12270	IPF6070	No significant S.c. match	
CA1686	1.0	1.1	1.0	IPF12272	complemer	unknown function	orf19.3659	12272	IPF12270	No significant S.c. match	
CA1687	1.0	1.1	1.0	IPF12275	6880909..6	unknown function	orf19.3658	12275	IPF12272	UNCLASSI	molecular_function unknown
CA1688	1.0	1.0	1.0	COX15	complemer	cytochrome oxidase as	orf19.3656	13028	IPF12275	UNCLASSI	molecular_function unknown
CA1689	0.9	0.9	1.0	IPF13030	6884979..6	unknown function	orf19.3655	13030	CaCOX15	ENERGY "	molecular_function unknown
CA1690	1.0	0.9	1.0	FAT1	6888232..6	very long-chain fatty ac	orf19.3653	13033	IPF13030	No significant S.c. match	
CA1691	3.9	2.4	2.7	PGK1	complemer	Phosphoglycerate kina	orf19.3651	17009	CaFAT1	Lipid fatty-a	ligase activity,transporter activity
CA1692	1.0	1.1	1.1	IPF17055.3	6893728..6	unknown function, 3-pr	orf19.5036	17055	CaPGK1	C-compour	transferase activity
CA1693	1.0	0.9	1.0	IPF17054	complemer	unknown function	orf19.5037	17054	IPF17055.3	UNCLASSI	transporter activity
CA1694	1.1	1.1	1.0	TRM3	6897030..6	2 -O-ribose methyltran	orf19.5038	12305	IPF17054	No significant S.c. match	
CA1695	0.9	0.9	0.9	RRP42	complemer	rRNA processing prote	orf19.5039	12306	CaTRM3	TRANSCR	transferase activity
CA1696	1.1	1.0	0.9	ASM4	6903004..6	similar to Saccharmoyc	orf19.5040	12310	CaRRP42	TRANSCR	RNA binding
CA1697	1.0	1.0	0.9	IPF16758	6907195..6	unknown function	orf19.5043	16758	CaASM4	CELL CYC	structural molecule activity
CA1698	0.9	1.0	0.9	IPF15681	6911160..6	unknown function	orf19.5045	15681	IPF16758	CELLULAR TRANSPORT AND TRANSPORT MECHANISMS SUBCELLULAR LOCALISATION	
CA1699	1.0	0.8	1.0	RAM1.3F	complemer	protein farnesyltransferase, beta s		19535	IPF15681	No significant S.c. match	
CA1700	1.0	1.0	1.0	RAM1.5F	complemer	protein farnesyltransfe	orf19.5046	19534	CaRAM1.3	Lipid fatty-acid and isoprenoid metabolism """"PROTEIN FATE [folding modification destination] ""CELLULAR COMMUNICATION/SIGNAL	
CA1701	1.0	1.1	1.0	CFL11	6919083..6	Ferric reductase (by hc	orf19.701	7992	CaRAM1.5	Lipid fatty-a	transferase activity
CA1702	1.0	1.0	0.9	HEL1	complemer	DNA helicase I (by hor	orf19.702	7990	CaCFL11	REGULATION OF/INTERACTION WITH CELLULAR ENVIRONMENT Other virulence attributes	
CA1703	1.1	1.0	1.0	IPF7987	complemer	unknown function	orf19.703	7987	CaHEL1	CELL CYC	DNA binding,helicase activity,RNA binding
CA1704	1.0	1.1	1.1	SOL3	complemer	weak multicopy suppre	orf19.704	19927	IPF7987	UNCLASSI	hydrolase activity
CA1705	1.1	1.1	1.0	GCN5	complemer	Histone acetyltransfera	orf19.705	7983	CaSOL3	TRANSCR	molecular_function unknown
CA1706	1.1	1.1	0.9	NMD3	6928772..6	RNA binding (by homo	orf19.706	7981	CaGCN5	CELL CYC	transferase activity
CA1708	1.1	0.9	1.0	IPF7543	6936155..6	unknown function	orf19.4888	7543	CaNMD3	TRANSCR	protein binding,RNA binding
CA1709	0.9	0.9	0.9	HOL2	complemer	Multidrug-resistance pi	orf19.4889	7544	IPF7543	No significant S.c. match	
CA1710	1.0	1.0	1.0	CLA4	complemer	protein kinase homolog	orf19.4890	16297	CaHOL2	CELL RES	transporter activity
CA1711	0.9	1.0	1.0	POX18	complemer	Lipid-transfer protein (l	orf19.1084	17142	CaCLA4	CELL CYC	protein kinase activity
CA1712	1.0	1.1	1.0	ABC1	6950284..6	ubiquinol--cytochrome-	orf19.1084	14939	CaPOX18	No significant S.c. match	
CA1713	1.0	1.1	1.0	IPF19743	6952488..6	unknown function	orf19.3332	19743	CaABC1	ENERGY "	chaperone activity
CA1714	1.0	1.0	1.1	NAB2	6955882..6	Nuclear poly(A)-RNA-b	orf19.3333	14410	IPF19743	UNCLASSI	molecular_function unknown
CA1715	0.9	1.2	1.4	RPS21	complemer	ribosomal protein (by h	orf19.3334	14407	CaNAB2	TRANSCR	RNA binding
CA1716	2.1	1.3	1.1	IPF17237	6959696..6	unknown function	orf19.3335	17237	CaRPS21	PROTEIN S	structural molecule activity
CA1717	1.0	0.9	1.0	IPF9315	complemer	putative CCAAT-bindir	orf19.4647	9315	IPF17237	No significant S.c. match	
CA1718	0.9	0.9	1.0	IPF9312	complemer	unknown function	orf19.4649	9312	IPF9315	TRANSCRIPTION SUBCELLULAR LOCALISATION	
CA1719	0.4	0.1	1.0	ILV6	complemer	acetolactate synthase,	orf19.1211	9306	IPF9312	TRANSCRIPTION SUBCELLULAR LOCALISATION	
CA1720	1.2	1.2	1.3	IPF15581	complemer	unknown function	orf19.1212	15581	CaILV6	Amino acid	enzyme regulator activity
CA1722	1.0	1.0	1.0	TEF41	6987263..6	Probable translation el	orf19.2652	16861	IPF15581	No significant S.c. match	
CA1723	0.9	0.9	1.0	TEF4	6988340..6	translation elongation f	orf19.2651	16860	CaTEF41	PROTEIN SYNTHESIS SUBCELLULAR LOCALISATION	
CA1724	1.0	1.0	1.0	MRP10	complemer	Mitochondrial ribosomal protein (b		18618	CaTEF4	PROTEIN S	translation regulator activity

CA1725	0.9	1.0	0.9	IPF15985	complemer	unknown function	orf19.2650	15985	CaMRP10	PROTEIN S	structural molecule activity
CA1726	1.1	1.0	1.0	IPF13021	complemer	unknown function	orf19.2647	13021	IPF15985	No significant S.c. match	
CA1727	0.9	1.0	1.0	IPF8448	complemer	unknown function	orf19.1384	8448	IPF13021	TRANSCRIPTION SUBCELLULAR LOCALISATION	
CA1728	0.9	0.8	0.9	WHI3	complemer	Putative RNA binding p	orf19.6494	8447	IPF8448	No significant S.c. match	
CA1729	1.0	1.1	1.1	IPF8440	7008772..7	similar to Saccharomy	orf19.6496	8440	CaWHI3	CELL FATE	RNA binding
CA1730	1.0	0.9	0.9	IPF8439	7009648..7	unknown function	orf19.6498	8439	IPF8440	CELLULAR	molecular_function unknown
CA1731	1.0	1.1	1.0	IPF8437	complemer	putative DNA-directed	orf19.6499	8437	IPF8439	UNCLASSI	molecular_function unknown
CA1732	0.9	1.0	1.0	ECM42	7014851..7	Acetylornithine acetyltr	orf19.6500	3933	IPF8437	CELL CYCLE AND DNA PROCESSING TRANSCRIPTION SUBCELLULAR LOCALISATION	
CA1733	1.0	1.0	1.0	IPF3931	complemer	Unknown function	orf19.1385	3931	CaECM42	Amino acid	transferase activity
CA1734	1.2	1.2	1.1	IPF4988	complemer	unknown function	orf19.2529	4988	IPF3931	UNCLASSIFIED PROTEINS	
CA1735	1.0	1.0	0.9	IPF4986	complemer	similar to Saccharomy	orf19.2528	4986	IPF4988	No significant S.c. match	
CA1736	1.0	1.0	1.0	IPF4983	7024104..7	unknown function	orf19.2527	4983	IPF4986	TRANSCR	transcription regulator activity
CA1737	1.3	1.3	1.1	LYS12	complemer	homo-isocitrate dehydr	orf19.2525	4979	IPF4983	UNCLASSI	molecular_function unknown
CA1738	0.8	0.9	0.6	MGE1	complemer	heat shock protein (by	orf19.2524	4977	CaLYS12	Amino acid	oxidoreductase activity
CA1739	1.0	1.0	1.0	IPF4976	complemer	unknown function	orf19.1005	4976	CaMGE1	PROTEIN F	chaperone activity
CA1740	0.9	1.1	0.8	SMI1	7034065..7	beta-1,3-glucan synthe	orf19.5058	12215	IPF4976	No significant S.c. match	
CA1741	1.1	1.0	1.0	IPF12213	complemer	unknown function	orf19.5057	12213	CaSMI1	C-compour	molecular_function unknown
CA1742	1.0	1.0	0.9	IPF12210	complemer	quinolinate phosphorib	orf19.5054	12210	IPF12213	No significant S.c. match	
CA1743	1.0	0.8	0.9	IPF12209	7040457..7	similar to Saccharomy	orf19.5053	12209	IPF12210	Metabolism	transferase activity
CA1744	0.9	0.9	0.9	IPF10889	complemer	unknown function	orf19.5052	10889	IPF12209	CELL CYC	transferase activity
CA1745	1.0	0.9	1.0	IPF10888	7042767..7	unknown function	orf19.5051	10888	IPF10889	TRANSPO	transporter activity
CA1746	1.0	1.0	1.0	IPF10886	complemer	unknown function	orf19.5050	10886	IPF10888	No significant S.c. match	
CA1747	0.8	0.7	0.7	IPF10884	7047421..7	unknown function	orf19.5049	10884	IPF10886	PROTEIN S	molecular_function unknown
CA1748	1.0	0.9	0.9	IPF8257	7052065..7	unknown function	orf19.1522	8257	IPF10884	UNCLASSI	molecular_function unknown
CA1749	1.0	0.9	1.0	IPF8257.3F	7055194..7	unknown function, 3-prime end		8253	IPF8257	No significant S.c. match	
CA1750	1.0	0.9	0.9	IPF8252	complemer	unknown function	orf19.1519	8252	IPF8257.3f	No significant S.c. match	
CA1751	1.1	1.0	1.1	ARO3.EXC	complemer	3-deoxy-D-arabinohep	orf19.1517	8248	IPF8252	CELL CYCLE AND DNA PROCESSING SUBCELLULAR LOCALISATION	
CA1752	0.9	0.8	1.0	UBP14	complemer	Ubiquitin-specific prote	orf19.1516	8246	CaARO3.e	Amino acid	transferase activity
CA1753	1.0	1.0	1.0	IPF8245	7064004..7	putative chitinase	orf19.1515	8245	CaUBP14	Lipid fatty-a	peptidase activity
CA1754	1.1	1.0	1.0	IPF16320	7068168..7	Unknown function	orf19.5131	16320	IPF8245	C-compour	molecular_function unknown
CA1755	1.1	1.2	1.2	PDI1	complemer	protein disulfide-isome	orf19.5130	16217	IPF16320	UNCLASSI	molecular_function unknown
CA1756	1.0	1.0	1.0	IPF14562	complemer	unknown function	orf19.5129	14562	CaPDI1	Lipid fatty-a	oxidoreductase activity,isomerase activity
CA1757	1.0	1.1	1.1	IPF14559.5	7075780..7	unknown function, 5-pr	orf19.5128	14559	IPF14562	No significant S.c. match	
CA1758	0.9	0.9	0.8	IPF14559.3	7076649..7	unknown function, 3-pr	orf19.5126	14556	IPF14559.5	No significant S.c. match	
CA1759	0.8	1.0	0.6	IPF14744	complemer	unknown function	orf19.5125	14744	IPF14559.3	TRANSCR	RNA binding
CA1760	1.0	1.0	1.0	IPF17914.3	complemer	unknown function	orf19.5124	17914	IPF14744	UNCLASSI	molecular_function unknown
CA1761	1.2	1.1	1.2	IPF7602	7085435..7	oxidoreductase (by hor	orf19.5193	7602	IPF17914.3	CONTROL OF CELLULAR ORGANIZATION	
CA1762	1.1	1.0	1.1	IPF20014	7086420..7	oxidoreductase by homology		19928	IPF7602	Lipid fatty-acid and isoprenoid metabolism	
CA1763	0.9	1.1	1.0	URA6	7087454..7	Uridine-monophosphat	orf19.5195	7599	IPF20014	Lipid fatty-a	molecular_function unknown
CA1764	1.0	1.0	1.0	IPF7596	7088486..7	similar to Saccharomy	orf19.5196	7596	CaURA6	Nucleotide	transferase activity
CA1765	1.0	1.2	1.0	APE2	7089585..7	aminopeptidase yscII	orf19.5197	7593	IPF7596	TRANSCR	isomerase activity
CA1766	0.9	0.9	0.9	NOP4	7092793..7	Nucleolar protein	orf19.5198	7589	CaAPE2	Amino acid	peptidase activity
CA1767	0.9	1.0	1.0	SIT4	complemer	Ser/thr protein phospha	orf19.5200	7586	CaNOP4	TRANSCR	RNA binding
CA1768	0.9	0.9	1.0	IPF7585	7096998..7	unknown function	orf19.5201	7585	CaSIT4	CELL CYC	protein phosphatase activity
CA1769	0.9	1.1	1.0	IPF7581	7099629..7	unknown function	orf19.5203	19744	IPF7585	PROTEIN S	structural molecule activity
CA1770	1.0	1.0	0.9	IPF12457	7104740..7	unknown function	orf19.2185	12457	IPF7581	No significant S.c. match	
CA1771	0.9	0.9	0.9	SEH1	complemer	nuclear pore protein (b	orf19.2186	12458	IPF12457	UNCLASSI	molecular_function unknown
CA1772	0.9	0.8	1.1	ALG7	7107522..7	UDP-N-acetylglucosan	orf19.2187	12460	CaSEH1	CELLULAR	structural molecule activity
CA1773	0.9	1.0	1.0	VRP1	7109211..7	verprolin (by homology	orf19.2190	12461	CaALG7	Phosphate	transferase activity
CA1774	1.0	1.1	1.1	IPF7498	complemer	unknown function	orf19.2191	7498	CaVRP1	CELLULAR	protein binding
CA1775	0.2	0.6	0.2	GDH2	complemer	NAD-specific glutamat	orf19.9738	19745	IPF7498	UNCLASSI	molecular_function unknown
CA1776	0.9	1.0	1.0	IPF14704	complemer	unknown function	orf19.1823	14704	CaGDH2	Amino acid	oxidoreductase activity
CA1777	1.1	1.1	1.1	IPF14706	complemer	unknown function	orf19.1824	14706	IPF14704	UNCLASSIFIED PROTEINS	
CA1778	1.0	0.9	1.1	IPF13810.3	complemer	unknown function, 3-pr	orf19.1825	13810	IPF14706	No significant S.c. match	
CA1779	1.0	0.9	0.9	IPF14510	7131977..7	unknown function	orf19.1826	14510	IPF13810.3	No significant S.c. match	
CA1780	1.0	0.9	1.1	IPF14509	7134782..7	unknown function	orf19.1827	14509	IPF14510	UNCLASSI	molecular_function unknown
CA1781	1.0	0.9	1.0	IPF14508	complemer	unknown function	orf19.1828	14508	IPF14509	UNCLASSIFIED PROTEINS	
CA1782	0.7	1.1	0.4	PHO84.3E	7138851..7	Inorganic phosphate tr	orf19.1172	7638	IPF14508	UNCLASSI	molecular_function unknown
CA1783	1.1	1.1	1.1	IPF7635	complemer	unknown function	orf19.1171	7635	CaPHO84.	Phosphate	transporter activity
CA1784	1.0	0.9	1.0	ARO7	complemer	chorismate mutase (by	orf19.1170	7632	IPF7635	No significant S.c. match	
CA1785	1.0	1.0	0.9	IPF7631	complemer	unknown function	orf19.1169	7631	CaARO7	Amino acid	isomerase activity
CA1786	1.4	1.1	1.2	IPF7629	complemer	unknown function	orf19.1168	7629	IPF7631	UNCLASSI	molecular_function unknown
CA1787	0.4	0.1	0.9	IFH3	complemer	Dioxygenase (by homo	orf19.1167	19929	IPF7629	No significant S.c. match	
CA1788	1.0	0.9	1.0	USO1.3	complemer	Cytoskeletal-related tra	orf19.1166	7623	CaIFH3	CELL RES	oxidoreductase activity
CA1789	1.0	1.3	1.0	GAR1	7154269..7	Nucleolar rRNA proces	orf19.1164	7621	CaUSO1.3	UNCLASSI	molecular_function unknown
CA1790	1.2	1.1	1.1	IPF13971	7156384..7	unknown function	orf19.5204	13971	CaGAR1	TRANSCR	RNA binding
CA1791	1.0	1.0	0.9	IPF13967	7158856..7	unknown function	orf19.5205	13967	IPF13971	UNCLASSI	molecular_function unknown
CA1792	1.1	1.1	1.0	IPF13966	complemer	unknown function	orf19.5206	13966	IPF13967	UNCLASSIFIED PROTEINS	
CA1793	1.0	1.0	0.9	IPF18608.5	7162049..7	unknown function, 5-pr	orf19.5207	18608	IPF13966	UNCLASSI	molecular_function unknown
CA1794	1.0	1.0	0.9	IPF18608.3	7162608..7	unknown function, 3-pr	orf19.5208	11262	IPF18608.5	CLASSIFICATION NOT YET CLEAR-CUT	
CA1795	1.0	1.0	1.0	IPF11261	complemer	unknown function	orf19.5209	11261	IPF18608.3	CLASSIFIC	molecular_function unknown
CA1796	1.0	0.9	1.1	IPF11259	complemer	unknown function	orf19.5210	11259	IPF11261	UNCLASSI	molecular_function unknown

CA1797	1.3	1.3	1.2	NUM12	7171839..7	nuclear migration prote	orf19.2924	15988	IPF11259	TRANSCR DNA binding,transcription regulator activity
CA1798	1.1	1.0	1.1	IPF11448	complemer	unknown function	orf19.2923	11448	CaNUM12	CELL CYCLE AND DNA PROCESSING
CA1799	1.0	1.0	1.0	IPF11446	complemer	unknown function	orf19.2922	11446	IPF11448	UNCLASSIFIED PROTEINS
CA1800	1.0	1.0	1.0	IPF11445	7179826..7	similar to Saccharomy	orf19.2921	11445	IPF11446	No significant S.c. match
CA1801	1.1	0.9	1.0	IPF11444	complemer	unknown function	orf19.2920	11444	IPF11445	CELL CYC chaperone activity
CA1802	1.0	1.0	1.0	IPF11443	complemer	unknown function	orf19.2919	11443	IPF11444	UNCLASSI RNA binding
CA1803	0.8	0.9	0.9	IPF16748	complemer	unknown function	orf19.2917	16748	IPF11443	CELL CYC RNA binding,helicase activity
CA1804	0.9	0.9	1.0	TAF17	7190946..7	TFIID and SAGA subu	orf19.1111	6333	IPF16748	CELLULAF molecular_function unknown
CA1805	1.0	1.1	1.1	IPF6332	7191994..7	similar to Saccharomy	orf19.1112	6332	CaTAF17	TRANSCR transcription regulator activity
CA1806	1.0	1.0	0.9	IPF6329	7194761..7	unknown function	orf19.1113	6329	IPF6332	C-compour molecular_function unknown
CA1807	1.2	1.0	1.1	IPF6328	complemer	unknown function	orf19.1114	6328	IPF6329	No significant S.c. match
CA1808	0.9	0.8	1.0	GUK1	complemer	Guanylate kinase (by h	orf19.1115	6326	IPF6328	UNCLASSI molecular_function unknown
CA1809	1.0	1.1	1.0	IPF6325	7198001..7	unknown function	orf19.1116	6325	CaGUK1	Nucleotide transferase activity
CA1810	1.0	1.1	1.0	FDH2	7199805..7	Formate dehydrogenas	orf19.1117	6323	IPF6325	No significant S.c. match
CA1811	1.0	1.1	1.0	MTR10	7201350..7	Involved in nuclear pro	orf19.1119	6321	CaFDH2	ENERGY
CA1812	1.0	1.1	0.9	IPF13879	complemer	unknown function	orf19.1120	13879	CaMTR10	TRANSCRIPTION CELLULAR TRANSPORT AND TRANSPORT MECHANISMS SUBCELLULAR LOCALISATION
CA1814	0.9	0.9	0.9	TRA1.5EO	complemer	ATM/Mec1/TOR1+2-re	orf19.139	14787	IPF13879	No significant S.c. match
CA1815	1.1	1.0	1.2	IPF18606	complemer	unknown function	orf19.138	18606	CaTRA1.5	TRANSCRIPTION
CA1816	1.0	1.0	1.1	IPF7020	7219916..7	unknown function	orf19.136	7020	IPF18606	CELL FATI molecular_function unknown
CA1817	1.0	1.1	1.0	IPF7021	complemer	similar to Saccharomy	orf19.135	7021	IPF7020	CELL RES molecular_function unknown
CA1818	1.1	1.0	1.1	IPF7023.3	complemer	unknown function, 3-pr	orf19.134	7023	IPF7021	CELLULAF molecular_function unknown
CA1819	1.0	0.9	0.9	IPF16549	7233471..7	Unknown function	orf19.4273	16549	IPF7023.3	No significant S.c. match
CA1820	0.9	1.0	1.0	IPF9529	complemer	probable mannosyltrar	orf19.4270	9529	IPF16549	TRANSCR molecular_function unknown
CA1821	0.5	0.4	0.8	IPF9527	7240345..7	unknown function	orf19.4269	9527	IPF9529	C-compound and carbohydrate metabolism ""PROTEIN FATE [folding modification destination] ""
CA1822	0.9	0.8	1.0	IPF9525	7242308..7	unknown function	orf19.4268	9525	IPF9527	UNCLASSIFIED PROTEINS
CA1823	1.1	0.9	0.9	IPF9522.5F	7244770..7	septin, 5-prime end (by	orf19.4267	9524	IPF9525	UNCLASSI RNA binding
CA1825	1.0	0.9	1.0	IPF9522.3F	7245596..7	septin, 3-prime end (by	orf19.4266	9522	IPF9522.5f	C-compound and carbohydrate metabolism CELL CYCLE AND DNA PROCESSING CELL FATE SUBCELLULAR LOCALISATION
CA1826	1.0	1.0	1.0	UAP1	complemer	UDP-N-acetylglucosan	orf19.4265	9521	IPF9522.3f	CELL CYCLE AND DNA PROCESSING CELL FATE SUBCELLULAR LOCALISATION
CA1827	1.1	0.9	1.2	IPF9520	complemer	unknown function	orf19.1174	9520	CaUAP1	CONTROL nucleotidyltransferase activity
CA1828	1.2	0.9	1.1	CDC22.3E	7252360..7	DNA Polymerase III, 3-prime end		17179	IPF9520	No significant S.c. match
CA1829	1.0	1.1	1.0	IPF17177.3	complemer	similar to Saccharomy	orf19.5184	17178	CaCDC22.	CELL CYCLE AND DNA PROCESSING SUBCELLULAR LOCALISATION
CA1830	1.0	1.2	1.0	IPF17177.5	complemer	similar to Saccharomy	orf19.5185	17177	IPF17177.3	TRANSCR signal transducer activity
CA1831	1.0	1.0	1.0	CHS1	complemer	Chitin synthase (by hc	orf19.5188	6129	IPF17177.5	TRANSCRIPTION CELLULAR TRANSPORT AND TRANSPORT MECHANISMS CELL FATE SUBCELLULAR LOCALISATION
CA1832	1.2	1.0	1.0	IPF17727	complemer	unknown function	orf19.5190	17727	CaCHS1	C-compour transferase activity
CA1834	1.4	2.2	1.0	PFK1	complemer	6-phosphofructokinase	orf19.3967	5769	IPF17727	CELL CYCLE AND DNA PROCESSING CELLULAR TRANSPORT AND TRANSPORT MECHANISMS CELL FATE SUBCELLULAR LOC
CA1835	0.9	1.0	1.0	CRH12	complemer	Cell wall protein (by hc	orf19.3966	5772	CaPFK1	C-compour transferase activity
CA1836	1.0	0.9	1.0	IPF5773	7279135..7	unknown function	orf19.3965	5773	CaCRH12	SUBCELLULAR LOCALISATION
CA1837	1.0	1.0	1.0	IPF5776	complemer	ash2-trithorax family pi	orf19.3964	5776	IPF5773	No significant S.c. match
CA1838	1.0	1.0	1.0	IPF5777	complemer	unknown function	orf19.3963	5777	IPF5776	UNCLASSI transcription regulator activity
CA1839	0.9	1.0	0.8	IPF4435	7284774..7	unknown function	orf19.4711	4435	IPF5777	UNCLASSI molecular_function unknown
CA1840	0.9	0.9	0.9	IFP3	complemer	Unknown Function	orf19.4707	4440	IPF4435	UNCLASSI molecular_function unknown
CA1841	1.0	1.0	1.0	CCA1	7291739..7	tRNA nucleotidyltransf	orf19.4705	4444	CaIFP3	No significant S.c. match
CA1842	0.9	1.1	1.0	ARO1	7293668..7	arom pentafunctional e	orf19.4704	4446	CaCCA1	TRANSCR nucleotidyltransferase activity
CA1843	0.9	0.9	1.0	IPF11364	complemer	unknown function	orf19.635	11384	CaARO1	Amino acid transferase activity
CA1844	1.0	1.0	1.1	IFL5.3	complemer	unknown function, 3-pr	orf19.654	19930	IPF11364	No significant S.c. match
CA1845	0.9	0.7	0.9	IPF11123	7311134..7	similar to Saccharomy	orf19.637	11123	CaIFL5.3	CELL RESCUE DEFENSE AND VIRULENCE ""CELL FATE
CA1846	1.0	1.2	0.6	FDH12	7313358..7	Formate dehydrogenas	orf19.638	11124	IPF11123	ENERGY S oxidoreductase activity
CA1847	0.9	1.0	0.9	IPF11127	7314688..7	unknown function	orf19.639	11127	CaFDH12	ENERGY oxidoreductase activity
CA1848	1.1	0.9	0.9	IPF11128	complemer	unknown function		11128	IPF11127	UNCLASSIFIED PROTEINS
CA1849	1.0	1.1	1.0	IPF19932	complemer	unknown function	orf19.640	19932	IPF11128	PROTEIN S structural molecule activity
CA1850	1.0	1.0	1.0	IPF14630	7317320..7	unknown function	orf19.641	14630	IPF19932	UNCLASSIFIED PROTEINS
CA1851	0.6	0.6	0.5	SAP155	complemer	Cell cycle protein, inter	orf19.642	14629	IPF14630	PROTEIN I molecular_function unknown
CA1852	1.0	1.1	1.0	IPF13885.F	7322082..7	unknown function, 5-prime end		14628	CaSAP155	CELL CYCLE AND DNA PROCESSING CELL FATE
CA1853	0.9	1.0	1.0	LYP1	7325015..7	lysine-specific high-affi	orf19.651	16499	IPF13885.r	No significant S.c. match
CA1854	1.0	1.1	0.9	IPF12611	7327934..7	unknown function	orf19.649	12611	CaLYP1	Amino acid metabolism CELLULAR TRANSPORT AND TRANSPORT MECHANISMS SUBCELLULAR LOCALISATION TRANSPORT FA
CA1855	0.4	0.6	0.4	GLN1	7336170..7	glutamate-ammonia lig	orf19.646	15149	IPF12611	UNCLASSI molecular_function unknown
CA1856	1.0	1.0	1.0	IPF13689	7343526..7	unknown function	orf19.9081	13689	CaGLN1	Amino acid ligase activity
CA1857	1.0	1.0	1.1	IPF15348	7345494..7	unknown function	orf19.9080	15348	IPF13689	No significant S.c. match
CA1858	1.0	1.0	1.0	IPF15344	7349681..7	unknown function	orf19.9076	15344	IPF15348	UNCLASSIFIED PROTEINS
CA1859	1.0	1.0	1.0	IPF19614	7358890..7	putative transcription fa	orf19.9073	19614	IPF15344	UNCLASSIFIED PROTEINS
CA1860	1.0	1.0	0.9	UTR4	7361856..7	unknown function	orf19.9072	16638	IPF19614	TRANSCRIPTION
CA1861	1.0	1.0	0.9	RAD32	7364965..7	DNA repair protein (by	orf19.866	15212	CaUTR4	Amino acid molecular_function unknown
CA1862	1.0	1.0	0.9	NMD2	complemer	Nonsense-mediated m	orf19.864	7641	CaRAD32	CELL CYC nucleotidyltransferase activity
CA1863	1.0	1.1	1.0	MRPL35	complemer	Ribosomal protein of tl	orf19.863	7643	CaNMD2	Nucleotide protein binding
CA1864	1.0	0.7	1.0	IPF7644	complemer	unknown function	orf19.862	7644	CaMRPL35	PROTEIN S structural molecule activity
CA1865	0.9	0.8	0.8	IPF7646	complemer	putative transcription fa	orf19.861	7646	IPF7644	No significant S.c. match
CA1866	0.9	0.8	0.9	IPF7647	7374630..7	unknown function	orf19.860	7647	IPF7646	TRANSCRIPTION SUBCELLULAR LOCALISATION
CA1868	1.1	1.1	1.0	IPF14501.3	7380194..7	putative G-protein, -tra	orf19.9992	18597	IPF7647	No significant S.c. match
CA1869	0.8	0.8	0.9	IPF15822	complemer	unknown function	orf19.9993	15822	IPF14501.3	UNCLASSI molecular_function unknown
CA1870	0.9	1.0	0.9	IPF16981.E	complemer	unknown function, exo	orf19.9994	16981	IPF15822	No significant S.c. match
CA1871	0.9	1.0	0.9	IPF16981.E	complemer	unknown function, exo	orf19.9995	18595	IPF16981.e	UNCLASSIFIED PROTEINS
CA1872	1.1	1.2	1.1	IPF6700	complemer	unknown function	orf19.2459	6700	IPF16981.e	UNCLASSI molecular_function unknown

CA1873	1.0	1.0	1.0	IPF6696	complemer	unknown function	orf19.9997	6696	IPF6700	No significant S.c. match
CA1874	1.0	1.2	1.1	TPK2	7401744..7	cAMP-dependent prote	orf19.2277	10304	IPF6696	No significant S.c. match
CA1875	1.0	1.1	0.9	RPB9	complemer	DNA-directed RNA pol	orf19.2276	10302	CaTPK2	TRANSCR protein kinase activity
CA1876	1.0	1.0	1.0	IPF10301	7404401..7	putative 60S ribosoma	orf19.2275	10301	CaRPB9	TRANSCR nucleotidyltransferase activity
CA1877	1.1	1.1	1.0	IPF10300	complemer	unknown function	orf19.2274	10300	IPF10301	PROTEIN ! molecular_function unknown
CA1878	1.0	1.0	1.0	IPF10298	7407778..7	unknown function	orf19.2272	10298	IPF10300	No significant S.c. match
CA1879	1.1	1.2	1.2	SMF12	complemer	manganese transporte	orf19.2270	19747	IPF10298	UNCLASSI transcription regulator activity
CA1880	1.0	1.0	1.0	IPF13316	complemer	unknown function	orf19.2269	13316	CaSMF12	PROTEIN I transporter activity
CA1881	1.0	1.0	0.9	RCK2	7416783..7	Ca/calmodulin-depend	orf19.9808	19933	IPF13316	UNCLASSI molecular_function unknown
CA1882	1.2	0.9	1.1	IPF19934	complemer	unknown function	orf19.9755	19934	CaRCK2	ENERGY (protein kinase activity
CA1883	1.2	1.3	1.1	IPF5479	7424011..7	unknown function	orf19.2209	5479	IPF19934	No significant S.c. match
CA1884	1.0	1.1	1.0	IPF5486	7427257..7	unknown function	orf19.2208	5486	IPF5479	UNCLASSIFIED PROTEINS
CA1886	1.1	1.1	1.0	RHO2.3F	7432655..7	GTP-binding protein of the RHO su		5490	IPF5486	No significant S.c. match
CA1887	1.1	1.0	1.0	IPF5496	7434209..7	unknown function	orf19.2204	5496	CaRHO2.3	C-compound and carbohydrate metabolism CELLULAR COMMUNICATION/SIGNAL TRANSDUCTION MECHANISM CELL FATE SUBCE
CA1888	1.1	0.9	1.1	IPF18594	complemer	unknown function	orf19.9748	18594	IPF5496	UNCLASSI molecular_function unknown
CA1889	1.0	1.0	1.1	CBP6	7437555..7	Apo-cytochrome B pre-mRNA proc		18593	IPF18594	C-compound and carbohydrate metabolism ENERGY
CA1891	0.3	0.4	0.2	IPF10196	7443681..7	unknown function	orf19.2752	10196	CaCBP6	TRANSCR molecular_function unknown
CA1892	0.9	0.9	0.9	IPF10197	complemer	Similarity to transcriptio	orf19.2753	10197	IPF10196	C-compour DNA binding,transcription regulator activity
CA1893	0.9	0.9	0.9	TIM13	7452428..7	subunit of mitochondria	orf19.2754	10200	IPF10197	TRANSCRIPTION
CA1894	1.1	1.1	1.0	PRE7.EXO	7453387..7	putative subunit of 20S	orf19.2755	10202	CaTIM13	PROTEIN I transporter activity
CA1895	1.0	1.0	1.0	PRE7.EXO	7453894..7	subunit of 20S proteasome, exon 2		12004	CaPRE7.e:	PROTEIN I peptidase activity
CA1896	1.0	1.0	1.0	ERD2	complemer	ER lumen protein retai	orf19.2756	12003	CaPRE7.e:	PROTEIN FATE [folding modification destination] ""SUBCELLULAR LOCALISATION
CA1897	1.0	1.0	1.0	IPF12002	complemer	unknown function	orf19.1027	12002	CaERD2	PROTEIN FATE [folding modification destination] ""CELLULAR TRANSPORT AND TRANSPORT MECHANISMS SUBCELLULAR LOCAL
CA1898	1.0	1.0	1.0	IPF11998	7457401..7	unknown function	orf19.1027	11998	IPF12002	CELL CYCLE AND DNA PROCESSING SUBCELLULAR LOCALISATION
CA1899	1.0	1.1	0.9	HDA1	7461699..7	Histone deacetylase (b	orf19.2606	15480	IPF11998	No significant S.c. match
CA1900	0.9	1.0	0.9	ARK1	complemer	actin regulating serine/	orf19.2605	14133	CaHDA1	CELL CYC hydrolase activity
CA1901	0.8	0.7	0.8	IPF14135	7467022..7	unknown function	orf19.2604	14135	CaARK1	CELL CYCLE AND DNA PROCESSING SUBCELLULAR LOCALISATION
CA1902	0.9	0.8	0.9	OPT1	7470380..7	oligopeptide transporte	orf19.2602	14139	IPF14135	UNCLASSI molecular_function unknown
CA1903	0.7	0.4	1.0	IPF2754	complemer	unknown function		2754	CaOPT1	CELL FATI transporter activity
CA1904	1.0	1.0	1.0	GRX5	complemer	Glutaredoxin	orf19.1029	7275	IPF2754	No significant S.c. match
CA1905	1.0	1.0	1.0	IPF7274	7478916..7	unknown function	orf19.2783	7274	CaGRX5	PROTEIN I oxidoreductase activity
CA1906	0.9	0.9	1.0	IPF7271	7480500..7	unknown function	orf19.2784	7271	IPF7274	CELL RESCUE DEFENSE AND VIRULENCE ""SUBCELLULAR LOCALISATION
CA1907	1.6	1.8	1.2	ATP7	complemer	F1F0-ATPase complex	orf19.2785	7270	IPF7271	No significant S.c. match
CA1908	1.0	1.0	1.0	APL3	7482636..7	AP-2 complex subunit,	orf19.2786	7268	CaATP7	ENERGY (structural molecule activity
CA1909	1.0	0.9	1.0	IPF20091	7487008..7	unknown function	orf19.1030	20091	CaAPL3	PROTEIN I molecular_function unknown
CA1910	1.0	1.1	1.0	IPF7260	complemer	unknown function	orf19.1030	7260	IPF20091	CELL FATE
CA1911	1.1	1.4	0.9	SSE1	7500200..7	heat shock protein of H	orf19.2435	13776	IPF7260	UNCLASSI transferase activity
CA1912	1.1	1.1	1.1	SKY1	7503972..7	SRPK1 like protein kin	orf19.2436	13772	CaSSE1	CELL RES chaperone activity
CA1913	1.0	1.0	1.0	ARC35	7506384..7	subunit of the Arp2/3 c	orf19.2437	16563	CaSKY1	UNCLASSI protein kinase activity
CA1914	1.0	0.9	1.0	IPF16564	complemer	putative mitochondrial	orf19.2438	16564	CaARC35	CELLULAF structural molecule activity
CA1915	0.9	1.0	1.0	IPF16565	7508297..7	unknown function	orf19.2439	16565	IPF16564	PROTEIN ! molecular_function unknown
CA1916	1.0	0.9	1.0	IPF16566.3	7509360..7	unknown function, 3-prime end		16566	IPF16565	Lipid fatty-acid and isoprenoid metabolism ""SUBCELLULAR LOCALISATION TRANSPORT FACILITATION
CA1917	1.1	1.1	1.0	IPF7479.3f	complemer	unknown function, 3-pr	orf19.2440	7479	IPF16566.3	No significa transporter activity,oxidoreductase activity
CA1918	1.0	0.9	1.0	IPF7479.5f	complemer	unknown function, 5-pr	orf19.2441	7478	IPF7479.3f	UNCLASSIFIED PROTEINS
CA1919	0.9	0.9	1.1	IPF7477	7512539..7	unknown function	orf19.2442	7477	IPF7479.5f	No significant S.c. match
CA1920	1.1	1.0	1.1	IPF7476	complemer	similar to Saccharomy	orf19.2443	7476	IPF7477	No significant S.c. match
CA1921	1.0	1.0	1.5	IPF7475	complemer	similar to Saccharomy	orf19.2444	7475	IPF7476	CELLULAF enzyme regulator activity
CA1922	1.1	1.0	1.1	DIP52	complemer	Dicarboxylic amino aci	orf19.2445	7474	IPF7475	CELLULAF molecular_function unknown
CA1923	1.0	1.0	1.1	IPF9130	complemer	unknown function	orf19.5539	9130	CaDIP52	Amino acid metabolism SUBCELLULAR LOCALISATION TRANSPORT FACILITATION
CA1924	1.1	1.7	1.2	IPF9126	7531935..7	unknown function	orf19.5537	9126	IPF9130	No significant S.c. match
CA1925	1.2	1.2	1.2	IPF18587	7540351..7	putative methyltransfer	orf19.8372	18587	IPF9126	CELL RES signal transducer activity
CA1926	0.9	0.9	1.1	IPF13407	complemer	Unknown function	orf19.8373	13407	IPF18587	CELL CYCLE AND DNA PROCESSING SUBCELLULAR LOCALISATION
CA1927	1.0	1.1	1.0	YBN5	complemer	Putative purine nucleo	orf19.754	13405	IPF13407	C-compound and carbohydrate metabolism ""PROTEIN FATE [folding modification destination] ""
CA1928	1.0	0.9	0.9	MRPL37	7545977..7	Mitochondrial ribosoma	orf19.755	13404	CaYBN5	UNCLASSI molecular_function unknown
CA1929	1.0	1.0	1.0	SAP7	complemer	secreted aspartyl prote	orf19.8376	19935	CaMRPL37	PROTEIN ! structural molecule activity
CA1930	1.0	1.0	1.0	IPF3906.3	7553342..7	unknown function, 3-pr	orf19.758	3906	CaSAP7	PROTEIN FATE [folding modification destination] ""Other virulence attributes
CA1931	1.1	1.0	1.1	IPF3905	complemer	similar to Saccharomy	orf19.759	3905	IPF3906.3	UNCLASSI signal transducer activity
CA1932	1.1	1.0	1.0	IPF3903	7557161..7	unknown function	orf19.760	3903	IPF3905	CELLULAF molecular_function unknown
CA1933	1.0	0.9	0.9	IPF18586	complemer	Unknown function	orf19.8381	18586	IPF3903	PROTEIN ! structural molecule activity
CA1934	1.1	1.1	0.9	YAP1802.3	7561674..7	cytoskeletal adaptor, member of A		8015	IPF18586	No significant S.c. match
CA1935	1.0	1.1	1.0	IPF19936	complemer	unknown function	orf19.4185	19936	CaYAP180	PROTEIN FATE [folding modification destination] ""CELLULAR TRANSPORT AND TRANSPORT MECHANISMS SUBCELLULAR LOCAL
CA1936	1.0	0.9	1.0	PCT1	7564083..7	cholinephosphate cytic	orf19.4186	8007	IPF19936	UNCLASSI protein phosphatase activity
CA1937	1.0	1.0	1.0	MMM1	7565694..7	mitochondrial outer me	orf19.4187	8006	CaPCT1	Lipid fatty-a nucleotidyltransferase activity
CA1938	0.9	1.0	1.0	NMD5	7567805..7	putative Nam7p/Upf1p	orf19.4188	8002	CaMMM1	SUBCELLU molecular_function unknown
CA1939	1.0	1.0	1.0	IPF8000	7572102..7	unknown function	orf19.4189	8000	CaNMD5	Nucleotide protein binding
CA1940	0.9	1.0	0.9	IPF7999	complemer	unknown function	orf19.4190	7999	IPF8000	No significant S.c. match
CA1941	1.0	1.0	0.9	IPF7998	7574570..7	Ribosomal protein L24	orf19.4191	7998	IPF7999	UNCLASSI molecular_function unknown
CA1942	0.9	0.9	1.0	HCT5.3EO	complemer	#N/A		7996	IPF7998	PROTEIN ! molecular_function unknown
CA1943				IPF11759	7577262..7579019				CaHCT5.3	TRANSCRIPTION
CA1944	0.8	0.8	0.9	IPF10645	7579486..7	unknown function	orf19.4738	10645		
CA1945	0.9	0.9	0.8	MSS116	complemer	RNA helicase of the DI	orf19.4739	10643	IPF10645	UNCLASSI molecular_function unknown
CA1946	0.9	0.9	1.0	IPF19937	complemer	putative peptidyl-tRNA	orf19.4740	19937	CaMSS116	TRANSCR RNA binding,helicase activity

CA1947	1.0	0.9	1.1	IPF10637	7585182..7	starvation protein -like	orf19.1220	10637	IPF19937	PROTEIN	hydrolase activity
CA1948	1.0	5.6	0.9	IPF14022	complemer	ATPase family gene (b	orf19.1220	14022	IPF10637	SUBCELLULAR LOCALISATION	
CA1949	0.8	0.9	0.9	IPF14021	complemer	dual specificity phosph	orf19.1220	14021	IPF14022	CLASSIFICATION NOT YET CLEAR-CUT	
CA1950	0.9	0.9	0.8	IPF14019	complemer	unknown function	orf19.1220	14019	IPF14021	CLASSIFIC	hydrolase activity
CA1951	1.0	0.9	0.9	HEM14	7593736..7	Mitochondrial protopor	orf19.1220	19663	IPF14019	UNCLASSI	molecular_function unknown
CA1952	0.8	0.7	0.9	IFP1	7599449..7	Unknown function	orf19.762	3901	CaHEM14	Metabolism	oxidoreductase activity
CA1953	1.0	1.0	1.1	IPF3899	complemer	similar to Saccharomy	orf19.763	3899	CaIFP1	No significant S.c. match	
CA1954	1.0	1.0	1.0	IPF3897.5	7602644..7	unknown function, 5-pr	orf19.764	18581	IPF3899	CELL CYC	RNA binding
CA1955	1.0	1.1	1.1	IPF3897.3	7603593..7	unknown function, 3-pr	orf19.765	3897	IPF3897.5f	UNCLASSI	molecular_function unknown
CA1956	1.1	0.9	2.1	ERG3	7606896..7	C5,6 desaturase	orf19.767	3890	IPF3897.3f	UNCLASSIFIED PROTEINS	
CA1957	1.1	1.0	1.0	IPF3887	7609190..7	similar to Saccharomy	orf19.768	3887	CaERG3	Lipid fatty-a	oxidoreductase activity
CA1958	1.0	1.1	1.1	IFE1	complemer	Unknown function	orf19.769	3885	IPF3887	REGULATI	molecular_function unknown
CA1959	1.1	1.0	1.2	IFD2	complemer	putative oxidoreductas	orf19.771	3883	CaIFE1	C-compour	oxidoreductase activity
CA1960	1.0	1.0	1.0	IPF15232	7617490..7	unknown function	orf19.2400	15232	CaIFD2	C-compour	oxidoreductase activity
CA1961	1.0	0.9	0.9	IPF14284	complemer	putative dnaJ-like prot	orf19.2399	15229	IPF15232	UNCLASSI	molecular_function unknown
CA1962	0.8	1.0	0.8	IPF14285	complemer	unknown function	orf19.2398	14285	IPF14284	PROTEIN I	molecular_function unknown
CA1963	1.0	0.9	1.0	IPF14040	7627840..7	probable transporter (b	orf19.2397	14040	IPF14285	No significant S.c. match	
CA1964	0.9	1.1	1.0	IFR2	7630467..7	unknown function	orf19.2396	14036	IPF14040	TRANSPORT FACILITATION	
CA1965	1.0	1.0	1.0	IPF14035	complemer	Similar to serine/threor	orf19.2395	14035	CaIFR2	SUBCELLULAR LOCALISATION	
CA1966	0.9	0.9	1.0	IFR4	complemer	unknown function	orf19.2394	11494	IPF14035	CELL CYC	protein kinase activity
CA1967	0.9	1.3	1.0	IPF10866	7638111..7	similar to Saccharomy	orf19.5219	10866	CaIFR4	SUBCELLU	oxidoreductase activity
CA1968	0.9	0.9	1.0	IPF10864	complemer	similar to Saccharomy	orf19.5220	10864	IPF10866	Nucleotide	enzyme regulator activity
CA1969	1.0	1.0	1.0	IPF18579.3	complemer	unknown function, 3-pr	orf19.5221	10863	IPF10864	CELL CYC	hydrolase activity
CA1970	0.7	0.5	0.9	IPF18579.5	complemer	unknown function, 5-pr	orf19.5222	18579	IPF18579.3	UNCLASSI	molecular_function unknown
CA1971	1.0	1.0	0.9	PKH2	7651993..7	Ser/Thr protein kinase	orf19.5224	10418	IPF18579.5	No significant S.c. match	
CA1972	0.8	0.9	0.9	RPL27A	7656496..7	ribosomal protein L27		10412	CaPKH2	UNCLASSI	protein kinase activity
CA1973	1.0	0.9	1.0	WRS1	7657314..7	tryptophan--tRNA ligas	orf19.5226	10411	CaRPL27A	PROTEIN	structural molecule activity
CA1974	1.0	1.2	1.1	IPF10410	complemer	unknown function	orf19.5227	10410	CaWRS1	PROTEIN	ligase activity
CA1975	1.0	1.0	1.0	PLB1	complemer	phospholipase B	orf19.689	9584	IPF10410	UNCLASSI	molecular_function unknown
CA1976	1.1	1.0	1.0	IPF9582	7666057..7	similar to Saccharomy	orf19.688	9582	CaPLB1	Lipid fatty-acid and isoprenoid metabolism ""Other virulence attributes	
CA1977	1.5	1.6	1.1	RPL25.3	7667499..7	ribosomal protein L23a, 3-prime er		9581	IPF9582	PROTEIN	structural molecule activity
CA1978	1.1	1.1	1.0	IPF9580	complemer	unknown function	orf19.687	9580	CaRPL25.3	PROTEIN	RNA binding
CA1979	0.8	0.7	0.8	IPF9577	7670060..7	unknown function	orf19.686	9577	IPF9580	No significant S.c. match	
CA1980	1.0	1.0	1.0	YHM1	complemer	member of the mitocho	orf19.685	16978	IPF9577	UNCLASSI	molecular_function unknown
CA1981	1.0	0.9	1.0	IPF9470	7674421..7	similar to Saccharomy	orf19.684	9470	CaYHM1	CELLULAF	transporter activity
CA1982	0.8	0.9	0.9	ADK1	complemer	adenylate kinase, cyto	orf19.683	20093	IPF9470	TRANSCR	protein binding
CA1983	1.5	1.6	1.3	ILV5	complemer	ketol-acid reducto-ison	orf19.7733	9595	CaADK1	Nucleotide	transferase activity
CA1984	1.0	1.0	1.1	IPF9592	7685772..7	unknown function	orf19.7736	9592	CaILV5	Amino acid	oxidoreductase activity
CA1985	1.0	1.0	1.0	IPF9591	complemer	unknown function	orf19.91	9591	IPF9592	UNCLASSIFIED PROTEINS	
CA1986	0.8	0.9	0.9	IPF14899	complemer	unknown function	orf19.92	14899	IPF9591	UNCLASSI	molecular_function unknown
CA1987	0.9	1.0	0.9	IPF14895	7693851..7	unknown function	orf19.7739	14895	IPF14899	UNCLASSI	molecular_function unknown
CA1988	1.1	1.0	1.0	IPF19749	complemer	unknown function	orf19.94	19749	IPF14895	UNCLASSI	molecular_function unknown
CA1989	1.1	0.9	1.0	IPF16596	complemer	unknown function	orf19.95	16596	IPF19749	No significant S.c. match	
CA1990	1.0	1.0	1.0	TOP1	complemer	CANAL DNA TOPOIS	orf19.7742	17557	IPF16596	No significant S.c. match	
CA1991	1.1	1.0	1.1	IPF6617.R	7702053..7	unknown function, rep	orf19.1383	6619	CaTOP1	CELL CYC	isomerase activity
CA1992	1.0	1.0	1.0	IPF6617.R	7703699..7	unknown function, rep	orf19.6484	6617	IPF6617.re	UNCLASSIFIED PROTEINS	
CA1993	1.1	1.0	1.1	IPF6614	7704943..7	unknown function	orf19.6482	6614	IPF6617.re	No significant S.c. match	
CA1994	1.0	0.8	1.1	IPF6613	7705947..7	unknown function	orf19.6481	6613	IPF6614	PROTEIN SYNTHESIS SUBCELLULAR LOCALISATION	
CA1995	1.0	1.1	1.0	IPF6612	complemer	unknown function	orf19.6480	6612	IPF6613	CLASSIFIC	peptidase activity
CA1996	1.1	1.0	1.2	SEC1	7710748..7	transport protein	orf19.6479	6609	IPF6612	UNCLASSI	transporter activity
CA1997	1.1	1.0	1.0	YCF1	7713550..7	Glutathione S-conjuga	orf19.6478	6607	CaSEC1	CELLULAF	protein binding
CA1998	1.0	1.1	1.0	IPF6605	complemer	unknown function	orf19.6477	6605	CaYCF1	CELLULAF	transporter activity
CA1999	0.9	1.0	1.0	IPF19513	complemer	unknown function	orf19.1383	19513	IPF6605	UNCLASSI	protein binding
CA2000	1.1	1.0	1.0	IPF11796	7724290..7	unknown function	orf19.2791	11796	IPF19513	UNCLASSI	molecular_function unknown
CA2001	1.5	1.5	1.1	IPF17074	7729908..7	unknown function	orf19.2792	17074	IPF11796	No significant S.c. match	
CA2002	1.0	0.9	1.0	IPF16470	complemer	unknown function	orf19.2794	16470	IPF17074	UNCLASSI	molecular_function unknown
CA2003	1.0	1.0	0.9	IPF16471	7734019..7	unknown function	orf19.2795	16471	IPF16470	UNCLASSI	isomerase activity
CA2004	1.0	0.9	0.9	POL12	7735550..7	DNA-directed DNA pol	orf19.2796	15718	IPF16471	TRANSCR	RNA binding
CA2005	1.1	1.0	1.1	IPF11802	7737793..7	unknown function	orf19.2797	11802	CaPOL12	CELL CYC	nucleotidyltransferase activity
CA2006	1.0	1.0	1.0	IPF11801	7739903..7	unknown function	orf19.2798	11801	IPF11802	UNCLASSIFIED PROTEINS	
CA2007	1.0	1.1	1.0	GPI8	7742136..7	essential for GPI anch	orf19.2799	11799	IPF11801	UNCLASSI	helicase activity
CA2008	0.9	1.0	1.0	IPF1742.3	7744454..7	unknown function, 3-prime end		1740	CaGPI8	Lipid fatty-a	hydrolase activity
CA2009	1.0	1.1	1.0	IPF1732	7747497..7	intramitochondrial prot	orf19.3089	1732	IPF1742.3f	No significant S.c. match	
CA2010	1.1	1.0	1.0	IPF1731	7748288..7	unknown function	orf19.3088	1731	IPF1732	PROTEIN I	molecular_function unknown
CA2011	1.9	2.8	1.5	RPS31	7749911..7	Ubiquitin fusion protein	orf19.3087	1727	IPF1731	REGULATI	molecular_function unknown
CA2012	1.0	1.0	1.0	SEC10	7750545..7	Required for exocytosi	orf19.3086	1725	CaRPS31	PROTEIN	structural molecule activity
CA2013	1.0	1.1	1.0	CDC1	complemer	Cell division control pr	orf19.3083	1720	CaSEC10	CELLULAF	protein binding
CA2014	1.1	1.0	0.9	IPF1717	complemer	similar to Saccharomy	orf19.1059	1717	CaCDC1	CELL CYC	molecular_function unknown
CA2015	0.9	0.9	1.0	IPF3416	7762798..7	unknown function	orf19.8468	14806	IPF1717	CELL CYC	protein kinase activity
CA2016	1.0	1.2	0.9	IPF3415	7764256..7	similar to Saccharomy	orf19.8467	3415	IPF3416	No significant S.c. match	
CA2017	1.1	1.0	0.9	IPF3414	complemer	putative serine/threoni	orf19.846	3414	IPF3415	PROTEIN I	peptidase activity
CA2018	1.1	1.1	1.0	STE11	complemer	ser/thr protein kinase c	orf19.844	3412	IPF3414	CLASSIFIC	protein kinase activity

CA2019	1.1	1.0	1.0	IPF3409	complemer	unknown function	orf19.8463	3409	CaSTE11	REGULATI	protein kinase activity,signal transducer activity
CA2020	2.2	0.9	2.9	IPF16901	complemer	unknown function	orf19.8462	16901	IPF3409	UNCLASSI	molecular_function unknown
CA2021	1.0	1.2	1.0	IPF17283	complemer	unknown function	orf19.4907	17283	IPF16901	No significant S.c. match	
CA2022	0.2	0.1	0.9	CBK1	complemer	serine/threonine protei	orf19.4909	9611	IPF17283	UNCLASSI	molecular_function unknown
CA2023	1.4	2.2	1.4	RPL42.3	complemer	ribosomal protein L36a, 3-prime er		9613	CaCBK1	CLASSIFIC	protein kinase activity
CA2024	1.0	1.0	1.0	IPF9616	complemer	unknown function	orf19.4910	9616	CaRPL42.3	PROTEIN S	structural molecule activity
CA2025	0.9	1.0	1.0	IPF9618	7791388..7	unknown function	orf19.4911	9618	IPF9616	No significant S.c. match	
CA2026	1.0	1.0	1.0	IPF9777	7796233..7	unknown function	orf19.1096	9777	IPF9618	No significant S.c. match	
CA2027	1.1	1.0	1.1	MCK1	complemer	ser/thr/tyr protein kinas	orf19.1096	9776	IPF9777	UNCLASSI	molecular_function unknown
CA2028	1.1	1.0	1.0	IPF18561.3	complemer	unknown function, , 3-prime end		18561	CaMCK1	CELL CYC	protein kinase activity
CA2029	1.0	1.1	1.1	SAR1.3	7804767..7	GTP-binding protein of	orf19.1096	13117	IPF18561.3	No significant S.c. match	
CA2030	0.9	0.8	0.9	IPF13116	7806274..7	unknown function	orf19.3463	13116	CaSAR1.3	CELLULAF	hydrolase activity
CA2031	1.8	2.3	1.6	RPL10A	complemer	L10A ribosomal protein	orf19.3465	13114	IPF13116	UNCLASSI	hydrolase activity
CA2032	1.0	1.7	1.1	IPF13112	7809533..7	unknown function	orf19.3466	13112	CaRPL10A	PROTEIN S	structural molecule activity
CA2033	1.0	0.8	1.0	SEC27	complemer	coatomer complex beta	orf19.1097	16985	IPF13112	UNCLASSIFIED PROTEINS	
CA2034	0.9	0.9	1.0	SGD1.3F	complemer	Involved in HOG pathway, 3-prime		18558	CaSEC27	CELLULAF	molecular_function unknown
CA2035	0.9	1.0	0.9	SGD1.5F	complemer	Involved in HOG pathw	orf19.1184	7574	CaSGD1.3	REGULATION OF/INTERACTION WITH CELLULAR ENVIRONMENT	
CA2036	1.0	1.0	1.0	MSP1	7823690..7	40 kDa putative memb	orf19.4362	7571	CaSGD1.5	REGULATI	molecular_function unknown
CA2037	1.1	1.0	1.0	IFF3	complemer	unknown function	orf19.1183	20094	CaMSP1	PROTEIN I	hydrolase activity
CA2038	1.1	1.0	1.1	IPF17510	complemer	unknown function	orf19.1183	17510	CaIFF3	UNCLASSIFIED PROTEINS	
CA2039	0.9	0.9	0.9	IPF12947	7830501..7	unknown function	orf19.1183	12947	IPF17510	UNCLASSI	molecular_function unknown
CA2040	1.0	1.0	1.1	IPF12946	complemer	unknown function	orf19.4356	12946	IPF12947	UNCLASSI	molecular_function unknown
CA2041	1.0	0.9	1.0	IPF12944	7836414..7	unknown function		12944	IPF12946	C-compour	molecular_function unknown
CA2042	0.9	0.9	1.1	IPF14219	complemer	probable membrane pr	orf19.1044	14219	IPF12944	No significant S.c. match	
CA2043	1.0	1.0	1.0	GSL21	complemer	1,3-beta-D-glucan synt	orf19.2929	10681	IPF14219	UNCLASSI	molecular_function unknown
CA2044	0.8	0.7	0.9	IPF10668	complemer	unknown function	orf19.1044	10668	CaGSL21	C-compour	transferase activity
CA2045	0.8	0.9	1.0	IPF8107	complemer	unknown function	orf19.1045	10665	IPF10668	UNCLASSI	translation regulator activity
CA2046	1.1	1.0	1.1	IPF8108	complemer	unknown function	orf19.1045	8108	IPF8107	UNCLASSI	molecular_function unknown
CA2047	1.6	2.5	1.3	RPL10	7856908..7	Ribosomal protein L10	orf19.1045	8109	IPF8108	UNCLASSI	molecular_function unknown
CA2049	0.9	0.9	1.0	IPF12982	complemer	unknown function	orf19.1314	12982	CaRPL10	CELL CYC	structural molecule activity
CA2050	0.9	1.0	1.0	IPF11023	complemer	unknown function	orf19.1314	11023	IPF12982	No significant S.c. match	
CA2051	1.0	1.0	1.0	PXP5	complemer	acyl-coenzyme A oxida	orf19.5723	11025	IPF11023	No significant S.c. match	
CA2052	1.0	1.0	1.0	IPF11027	7867877..7	unknown function	orf19.5722	11027	CaPXP5	Lipid fatty-acid and isoprenoid metabolism ""ENERGY SUBCELLULAR LOCALISATION	
CA2053	1.1	1.1	1.0	IPF11029	complemer	unknown function	orf19.5720	11029	IPF11027	TRANSCR	molecular_function unknown
CA2055	0.9	0.8	1.0	SAP4	7877137..7	secreted aspartyl prote	orf19.5716	8099	IPF11029	TRANSPORT FACILITATION	
CA2056	0.9	0.9	1.0	URA5	7881609..7	Orotate phosphoribosy	orf19.2555	7845	CaSAP4	PROTEIN FATE [folding modification destination] ""Other virulence attributes	
CA2057	0.9	0.9	0.9	SEC65	complemer	Recognition particle su	orf19.2557	7847	CaURA5	Nucleotide	transferase activity
CA2058	1.1	0.9	1.1	IPF7848	7883632..7	unknown function	orf19.2558	7848	CaSEC65	PROTEIN I	molecular_function unknown
CA2059	1.0	1.4	1.1	CDC4	complemer	CANAL CELL DIVISIO	orf19.2559	7849	IPF7848	No significant S.c. match	
CA2060	1.0	1.1	1.0	CDC61.3F	complemer	Cytosolic leucyl-tRNA s	orf19.2560	7853	CaCDC4	CELL CYC	protein binding
CA2061	0.9	1.1	0.9	CDC61.5F	complemer	Cytosolic leucyl-tRNA s	orf19.2562	7856	CaCDC61.	PROTEIN SYNTHESIS SUBCELLULAR LOCALISATION	
CA2062	0.9	1.0	1.0	IPF7858	complemer	similar to Saccharomy	orf19.2563	7858	CaCDC61.	PROTEIN S	ligase activity
CA2063	1.0	1.0	1.0	IPF7859	7895146..7	unknown function	orf19.2564	7859	IPF7858	TRANSCR	RNA binding
CA2064	0.9	1.0	0.9	IPF13024	complemer	unknown function	orf19.1016	13024	IPF7859	No significant S.c. match	
CA2065	0.7	0.8	0.5	QCR2	7905191..7	Ubiquinol--cytochrome	orf19.2644	6978	IPF13024	TRANSCRIPTION SUBCELLULAR LOCALISATION	
CA2066	0.9	0.8	0.9	RPO26	7906716..7	DNA-directed RNA pol	orf19.2643	6977	CaQCR2	ENERGY " transporter activity,oxidoreductase activity	
CA2067	1.0	1.0	0.9	IPF6976	complemer	unknown function	orf19.2642	6976	CaRPO26	TRANSCR	nucleotidyltransferase activity
CA2068	0.9	0.9	1.1	ARP1	7908123..7	centractin (by homolog	orf19.2641	6975	IPF6976	CELL CYCLE AND DNA PROCESSING CELLULAR TRANSPORT AND TRANSPORT MECHANISMS CELL FATE SUBCELLULAR LOCA	
CA2069	1.0	1.0	1.0	FUR1	complemer	Uracil phosphoribosylt	orf19.2640	6973	CaARP1	CELL CYC	structural molecule activity
CA2070	1.0	1.0	1.0	IPF6971.3	complemer	unknown function, 3-prime end		6972	CaFUR1	Nucleotide	transferase activity
CA2071	0.8	1.0	1.1	IPF6971.5	complemer	unknown function, internal fragmer		6971	IPF6971.3	No significant S.c. match	
CA2072	1.1	0.9	1.0	IPF6970	complemer	unknown function	orf19.2639	6970	IPF6971.5	TRANSCR	RNA binding
CA2073	1.0	1.0	0.9	IPF6967	7915229..7	unknown function	orf19.1016	6967	IPF6970	PROTEIN S	structural molecule activity
CA2074	1.0	1.0	0.9	IPF3733	7918556..7	unknown function	orf19.5287	3733	IPF6967	No significant S.c. match	
CA2075	1.0	1.8	1.0	IFE2	7921959..7	Unknown function	orf19.5288	3735	IPF3733	C-compound and carbohydrate metabolism TRANSCRIPTION CELL FATE SUBCELLULAR LOCALISATION	
CA2076	1.2	1.4	1.2	ERO1	7930054..7	Required for protein di	orf19.4871	13354	CaIFE2	C-compound and carbohydrate metabolism	
CA2077	1.1	0.9	1.0	IPF13353	7932518..7	unknown function	orf19.4872	13353	CaERO1	PROTEIN I	transporter activity
CA2078	1.1	1.1	1.0	IPF13352	complemer	unknown function	orf19.4873	13352	IPF13353	No significant S.c. match	
CA2079	1.0	1.1	1.0	MNN3	complemer	Golgi alpha-1,2-mannc	orf19.4874	13350	IPF13352	No significant S.c. match	
CA2080	0.9	1.0	0.9	IPF9693	7938813..7	unknown function	orf19.4875	9693	CaMNN3	CELL FATE	
CA2081	1.0	1.1	1.1	IPF9699	complemer	unknown function	orf19.4878	9699	IPF9693	UNCLASSI	molecular_function unknown
CA2082	1.1	1.1	1.1	NTF2	complemer	nuclear transport factor (by homolo		9700	IPF9699	UNCLASSI	molecular_function unknown
CA2083	1.0	0.9	0.9	IPF3384.5F	7950724..7	similar to Saccharomy	orf19.832	3384	CaNTF2	CELLULAF	protein binding
CA2084	1.1	1.1	1.1	IPF3384.3F	7953392..7	similar to Saccharomy	orf19.833	3385	IPF3384.5f	Secondary	transferase activity
CA2085	0.9	0.9	1.0	IPF3388	7954273..7	unknown function	orf19.834	3388	IPF3384.3f	Secondary metabolism	
CA2086	1.0	1.0	0.9	IPF3392	7957232..7	unknown function	orf19.835	3392	IPF3388	C-compound and carbohydrate metabolism	
CA2087	1.0	0.9	1.1	IPF3393	complemer	unknown function	orf19.836	3393	IPF3392	UNCLASSI	molecular_function unknown
CA2088	0.9	0.8	1.0	IPF3394	7961096..7	unknown function		3394	IPF3393	No significant S.c. match	
CA2089	1.0	1.0	1.1	GNA1	complemer	Acetyltransferase	orf19.837	3395	IPF3394	TRANSCR	RNA binding
CA2090	0.9	1.1	1.0	IPF3398.3	7963718..7	unknown function, 3-prime end		3398	CaGNA1	CELL CYC	transferase activity
CA2091	0.9	0.8	0.9	IPF3401	complemer	unknown function	orf19.839	3401	IPF3398.3	No significant S.c. match	
CA2092	1.7	1.3	1.4	RPL21A.3	7968235..7	Ribosomal protein, 3-p	orf19.840	3404	IPF3401	UNCLASSI	molecular_function unknown

CA2093	1.1	1.2	1.0	IPF3406	7969031..7	unknown function	orf19.841	3406	CaRPL21A	PROTEIN S	structural molecule activity
CA2094	1.0	1.0	1.0	IPF11493	complemer	unknown function	orf19.9929	11493	IPF3406	CELL CYC	molecular_function unknown
CA2095	0.9	0.8	0.9	IPF11492	complemer	unknown function	orf19.2392	11492	IPF11493	TRANSCRIPTION	
CA2096	1.0	1.0	1.0	IPF11491	7975446..7	unknown function	orf19.2391	11491	IPF11492	No significant S.c. match	
CA2097	0.9	0.9	0.9	IPF11489	complemer	unknown function	orf19.2389	11489	IPF11491	UNCLASSI	molecular_function unknown
CA2098	1.1	1.0	1.0	IPF11487	complemer	unknown function	orf19.2387	11487	IPF11489	CELL CYC	molecular_function unknown
CA2099	0.9	0.9	0.9	IPF11484	7979812..7	unknown function	orf19.2386	11484	IPF11487	UNCLASSI	molecular_function unknown
CA2100	1.1	1.0	1.0	KTI12	complemer	involved in resistance t	orf19.2385	9366	IPF11484	UNCLASSI	RNA binding
CA2101	1.0	1.2	1.0	IPF9364	7981669..7	unknown function	orf19.2384	9364	CaKTI12	CELL RES	enzyme regulator activity
CA2102	1.2	1.1	1.1	IPF9363	complemer	similar to Saccharomy	orf19.2383	9363	IPF9364	UNCLASSI	hydrolase activity
CA2103	0.9	0.9	1.1	ISM1	7984853..7	isoleucyl-tRNA synthet	orf19.9918	9361	IPF9363	CELL CYC	DNA binding
CA2104	1.0	0.9	1.0	IPF19939	7988103..7	unknown function	orf19.9917	19939	CaISM1	PROTEIN S	ligase activity
CA2105	1.0	1.0	1.1	IPF10711	7993256..7	unknown function	orf19.3694	10711	IPF19939	No significant S.c. match	
CA2106	1.1	1.1	1.1	IPF10714	7997299..7	similar to pH-regulated	orf19.3693	10714	IPF10711	UNCLASSIFIED PROTEINS	
CA2107	1.0	1.0	1.0	IPF10716	complemer	unknown function	orf19.3691	10716	IPF10714	CONTROL	molecular_function unknown
CA2108	1.5	1.1	1.5	RPL26A.3	8001738..8	ribosomal protein, 3-prime end (by		10719	IPF10716	UNCLASSI	molecular_function unknown
CA2109	0.9	0.9	0.8	IPF13749.5	8003001..8	unknown function, 5-pr	orf19.3690	13749	CaRPL26A	PROTEIN S	RNA binding
CA2110	0.9	0.9	0.8	IPF13749.3	8004053..8	unknown function, 3-pr	orf19.3689	18544	IPF13749.5	C-compour	molecular_function unknown
CA2111	0.9	0.9	1.0	IPF13748	8004941..8	Unknown function	orf19.3688	13748	IPF13749.3	C-compound and carbohydrate metabolism ENERGY	
CA2112	1.1	1.1	1.0	PFD1	complemer	Prefoldin subunit 1 (by	orf19.3687	13747	IPF13748	UNCLASSI	molecular_function unknown
CA2113	1.1	1.1	1.1	ATP12	8005890..8	F1F0-ATPase comple›	orf19.3686	13746	CaPFD1	PROTEIN I	chaperone activity
CA2114	1.0	0.9	1.0	IPF13744	8007912..8	protein involved in regu	orf19.3685	13744	CaATP12	PROTEIN I	chaperone activity
CA2115	1.0	1.1	1.1	IPF8030	8014628..8	unknown function	orf19.8911	8030	IPF13744	C-compour	molecular_function unknown
CA2116	1.0	1.0	1.1	SNG4	complemer	Drug transporter (by hc	orf19.1332	8028	IPF8030	CELL CYC	molecular_function unknown
CA2117	1.0	1.0	1.0	SNG3	8018926..8	Drug transporter (by hc	orf19.1333	8025	CaSNG4	CELL RESCUE DEFENSE AND VIRULENCE	
CA2118	1.1	1.0	1.0	IPF8024	8023334..8	unknown function	orf19.1334	8024	CaSNG3	CELL RESCUE DEFENSE AND VIRULENCE ""TRANSPORT FACILITATION	
CA2119	0.9	1.0	0.9	MTR4	8025570..8	RNA Helicase (by hom	orf19.8915	8020	IPF8024	No significant S.c. match	
CA2120	1.0	1.0	1.1	PUP3	8029039..8	PRCT yeast proteasome compone		8017	CaMTR4	TRANSCR	RNA binding,helicase activity
CA2121	1.1	1.1	1.0	IPF7930	8030927..8	unknown function	orf19.8918	7930	CaPUP3	PROTEIN I	peptidase activity
CA2122	1.1	1.1	1.1	CPY1.3F	complemer	Carboxypeptidase Y pi	orf19.8919	18540	IPF7930	UNCLASSI	molecular_function unknown
CA2124	1.1	1.1	1.0	IPF10490	complemer	unknown function	orf19.8049	10490	CaCPY1.3f	PROTEIN FATE [folding modification destination] ""SUBCELLULAR LOCALISATION	
CA2125	0.9	1.1	1.0	IPF4893	8040201..8	unknown function	orf19.418	4893	IPF10490	No significant S.c. match	
CA2126	1.1	1.0	1.1	IPF4896	8045299..8	unknown function	orf19.417	4896	IPF4893	UNCLASSI	protein binding
CA2127	0.8	0.8	1.0	IPF4897	complemer	unknown function	orf19.416	4897	IPF4896	CELLULAF	transporter activity
CA2128	1.0	1.2	1.0	IPF4898	complemer	unknown function	orf19.415	4898	IPF4897	No significant S.c. match	
CA2129	0.9	1.0	1.0	IPF4899	8048921..8	unknown function	orf19.414	4899	IPF4898	UNCLASSI	molecular_function unknown
CA2130	1.1	1.0	1.1	RPS27A	complemer	ribosomal protein S27.e (by homol		4901	IPF4899	UNCLASSI	structural molecule activity
CA2131	1.1	0.9	1.0	IDP1	complemer	isocitrate dehydrogena	orf19.5211	5863	CaRPS27A	PROTEIN SYNTHESIS SUBCELLULAR LOCALISATION	
CA2132	0.9	0.8	1.0	IPF5865	8057765..8	unknown function	orf19.5212	5865	CaIDP1	C-compound and carbohydrate metabolism ENERGY SUBCELLULAR LOCALISATION	
CA2133	1.2	1.2	1.2	IPF5866	8059498..8	unknown function	orf19.5213	5866	IPF5865	CELL CYC	DNA binding
CA2134	0.8	0.6	1.0	COX8	complemer	CYTOCHROME C OX	orf19.5213	5867	IPF5866	TRANSCRIPTION SUBCELLULAR LOCALISATION	
CA2135	1.1	1.2	1.1	COX9	8062863..8	CYTOCHROME C OXIDASE (by h		5868	CaCOX8	ENERGY S	oxidoreductase activity
CA2136	1.0	0.9	1.0	VPS33	complemer	VACUOLAR PROTEIN	orf19.5214	5870	CaCOX9	ENERGY "	oxidoreductase activity
CA2137	1.0	1.0	1.0	ARP9	8076550..8	actin-related protein (b	orf19.2507	15652	CaVPS33	PROTEIN FATE [folding modification destination] ""CELLULAR TRANSPORT AND TRANSPORT MECHANISMS CONTROL OF CELLUL	
CA2139	1.0	0.9	1.0	IPF15654.E	complemer	unknown function, exo	orf19.2509	15654	CaARP9	SUBCELLU	transcription regulator activity
CA2140	0.9	3.9	0.8	IPF19665	complemer	unknown function	orf19.1004	19665	IPF15654.e	No significant S.c. match	
CA2141	0.9	0.9	0.8	IPF19664	complemer	unknown function	orf19.1004	19664	IPF19665	CELL CYC	chaperone activity
CA2142	0.9	1.1	1.0	MRPL33	8084879..8	ribosomal protein of the large subu		15872	IPF19664	CELL CYCLE AND DNA PROCESSING SUBCELLULAR LOCALISATION	
CA2143	1.1	1.1	1.1	IPF12803	8087503..8	unknown function	orf19.1004	12803	CaMRPL33	PROTEIN S	structural molecule activity
CA2144	1.1	1.0	1.0	IPF12802	complemer	unknown function	orf19.2513	12802	IPF12803	No significant S.c. match	
CA2145	1.1	1.1	1.0	IPF12800	8090630..8	unknown function	orf19.2514	12800	IPF12802	PROTEIN FATE [folding modification destination] ""SUBCELLULAR LOCALISATION	
CA2146	0.8	0.8	0.6	IPF12799	complemer	unknown function	orf19.2515	12799	IPF12800	PROTEIN FATE [folding modification destination]	
CA2147	1.1	1.0	1.0	IPF18533	8095958..8	unknown function	orf19.2516	18533	IPF12799	No significant S.c. match	
CA2148	1.0	1.0	1.0	AUT1	complemer	similar to Saccharomy	orf19.6020	10762	IPF18533	Lipid fatty-acid and isoprenoid metabolism """"PROTEIN FATE [folding modification destination] ""SUBCELLULAR LOCALISATION	
CA2149	1.1	1.5	1.4	IPF10761	complemer	unknown function	orf19.6021	10761	CaAUT1	PROTEIN I	molecular_function unknown
CA2150	1.0	0.9	0.9	IPF19940	8108317..8	unknown function	orf19.6022	19940	IPF10761	No significant S.c. match	
CA2151	1.0	1.0	1.0	ERC2	complemer	ethionine resistance pr	orf19.6023	7898	IPF19940	No significant S.c. match	
CA2152	0.9	0.9	1.0	IPF7899	complemer	unknown function	orf19.6024	7899	CaERC2	UNCLASSIFIED PROTEINS	
CA2153	1.0	1.0	1.1	IPF7900	complemer	unknown function	orf19.6025	7900	IPF7899	No significant S.c. match	
CA2154	1.0	1.0	1.0	ERG2	8115702..8	C-8 sterol isomerase	orf19.6026	7901	IPF7900	UNCLASSI	molecular_function unknown
CA2155	1.1	1.1	1.0	IPF7903	complemer	unknown function	orf19.6027	7903	CaERG2	Lipid fatty-a	isomerase activity
CA2156	1.2	1.1	1.1	IPF8535	complemer	unknown function	orf19.5302	8535	IPF7903	CELL CYC	structural molecule activity
CA2157	1.2	1.1	1.1	IPF8537	complemer	similar to Saccharomy	orf19.5300	8537	IPF8535	No significant S.c. match	
CA2158	1.0	1.0	0.9	ECM1	complemer	involved in cell wall bic	orf19.5299	8538	IPF8537	SUBCELLU	molecular_function unknown
CA2159	1.0	0.9	1.0	IPF19753	complemer	similar to Saccharomy	orf19.5297	19753	CaECM1	CONTROL	molecular_function unknown
CA2160	1.0	1.0	1.1	IPF15664	8131953..8	unknown function	orf19.5296	15664	IPF19753	CELL CYC	transcription regulator activity
CA2161	1.0	1.0	0.9	IPF11876	8133201..8	unknown function	orf19.5295	11876	IPF15664	UNCLASSI	molecular_function unknown
CA2162	1.3	1.4	1.1	PDB1	complemer	pyruvate dehydrogena	orf19.5294	11877	IPF11876	No significant S.c. match	
CA2163	1.0	1.1	1.0	IPF11879	8137348..8	unknown function	orf19.5293	11879	CaPDB1	C-compour	oxidoreductase activity
CA2164	1.1	1.1	1.1	AXL2	complemer	similar to saccharomyc	orf19.5292	11881	IPF11879	No significant S.c. match	
CA2165	1.0	1.0	1.0	IPF17024	complemer	unknown function	orf19.5291	17024	CaAXL2	CELL FATI	molecular_function unknown
CA2166	1.1	1.1	0.9	FEN12	8148907..8	Probable subunit of 1,3	orf19.908	4547	IPF17024	Lipid fatty-a	molecular_function unknown

CA2167	1.1	1.1	1.0	IPF4553	8153414..8	unknown function	orf19.909	4553	CaFEN12	C-compound and carbohydrate metabolism ""Lipid fatty-acid and isoprenoid metabolism ""CELL FATE SUBCELLULAR LOCALISATION	
CA2168	1.0	1.0	1.0	IPF4558	complemer	similar to Saccharomy	orf19.910	4558	IPF4553	Amino acid	molecular_function unknown
CA2169	1.0	1.0	1.0	IPF4563.5I	8159313..8	similar to saccharomy	orf19.911	4563	IPF4558	TRANSCR	RNA binding
CA2170	1.0	0.9	0.9	IPF4563.3I	8164556..8	similar to saccharomy	orf19.912	18528	IPF4563.5f	CELL CYCLE AND DNA PROCESSING	
CA2171	1.3	1.1	1.0	PEP1.3	8166543..8	Vacuolar protein sortin	orf19.3767	15204	IPF4563.3f	CELL CYCLE AND DNA PROCESSING	
CA2172	1.0	0.9	1.1	IPF15377	8173047..8	probable membrane pr	orf19.3765	15377	CaPEP1.3	PROTEIN FATE [folding modification destination] ""CELLULAR TRANSPORT AND TRANSPORT MECHANISMS SUBCELLULAR LOCAL	
CA2173	0.9	0.8	1.0	IPF18527	8177798..8	unknown function	orf19.3764	18527	IPF15377	UNCLASSI	molecular_function unknown
CA2174	1.0	1.0	1.1	IPF20096	complemer	unknown function	orf19.3763	20096	IPF18527	CELL CYC	molecular_function unknown
CA2175	0.9	1.0	1.0	IPF8892	8181927..8	unknown function	orf19.3762	8892	IPF20096	No significant S.c. match	
CA2176	1.0	0.9	1.0	CDC54	complemer	cell division control pro	orf19.1124	8893	IPF8892	No significant S.c. match	
CA2177	1.1	1.0	1.0	ROD1	complemer	O-dinitrobenzene,calci	orf19.9084	19576	CaCDC54	CELL CYC	DNA binding
CA2178	1.0	0.9	1.1	IPF9282	complemer	unknown function	orf19.1510	9282	CaROD1	CELL RES	molecular_function unknown
CA2179	0.9	1.0	1.0	FAB1	complemer	phosphatidylinositol 3-|	orf19.9088	9288	IPF9282	UNCLASSI	molecular_function unknown
CA2180				IPF9290	8207665..8208687				CaFAB1	Lipid fatty-a	transferase activity
CA2181	1.0	1.1	1.0	ECM331	8218869..8	Involved in cell wall bic	orf19.4255	6869			
CA2182	1.0	1.0	1.0	IPF6871	complemer	unknown function	orf19.4253	6871	CaECM331	CELL CYC	molecular_function unknown
CA2183	0.9	0.8	0.9	IPF6872	8223014..8	serine/threonine protei	orf19.4252	6872	IPF6871	Nucleotide metabolism SUBCELLULAR LOCALISATION	
CA2184	1.0	0.9	1.0	IPF6874.3	complemer	unknown function, 3-pr	orf19.4251	6874	IPF6872	CLASSIFIC	protein kinase activity
CA2185	0.9	0.8	0.9	IPF6880	8228949..8	unknown function	orf19.4247	6880	IPF6874.3	No significant S.c. match	
CA2186	0.8	0.7	1.1	IPF6881	complemer	putative phosphatidyl s	orf19.4246	6881	IPF6880	No significant S.c. match	
CA2187	1.0	1.0	1.1	IPF11667	8241456..8	unknown function	orf19.4771	11667	IPF6881	UNCLASSIFIED PROTEINS	
CA2188	1.3	1.9	1.5	SSU81	complemer	protein involved in the	orf19.4772	19612	IPF11667	PROTEIN FATE [folding modification destination]	
CA2189	2.2	1.9	1.4	AOX2	8249622..8	alternative oxidase (by	orf19.4773	9420	CaSSU81	CELL RES	signal transducer activity
CA2190	1.2	1.0	1.3	AOX1	8252054..8	alternative oxidase (by	orf19.4774	9418	CaAOX2	No significant S.c. match	
CA2191	0.7	0.7	0.8	IPF9417	8255108..8	similar to Saccharomy	orf19.4775	9417	CaAOX1	No significant S.c. match	
CA2193	1.0	0.9	0.9	IPF14273	8268453..8	Probable ser/thr protei	orf19.1182	14273	IPF9417	CELL CYC	DNA binding,transcription regulator activity
CA2194	1.0	1.0	1.0	RGR1	8271407..8	DNA-directed RNA pol	orf19.1182	14271	IPF14273	CLASSIFIC	protein kinase activity,signal transducer activity
CA2195	1.0	1.0	1.0	IPF18517.3	complemer	unknown function, 3-pr	orf19.1182	16819	CaRGR1	C-compour	transcription regulator activity
CA2196	1.0	1.0	0.9	IPF18517.5	complemer	unknown function, 5-pr	orf19.1182	18517	IPF18517.3	UNCLASSI	molecular_function unknown
CA2197	1.0	0.9	1.0	IPF8110	8290521..8	unknown function	orf19.2936	8110	IPF18517.5	No significant S.c. match	
CA2198	1.2	1.4	1.3	PMM1	8291882..8	phosphomannomutase	orf19.2937	8112	IPF8110	No significant S.c. match	
CA2199	1.0	1.0	1.0	IPF8113	complemer	unknown function	orf19.2938	8113	CaPMM1	C-compour	isomerase activity
CA2200	1.0	1.0	0.9	IPF8114	8295006..8	unknown function	orf19.2939	8114	IPF8113	UNCLASSI	molecular_function unknown
CA2201	0.9	1.0	1.1	BOS1	8295935..8	ER-to-Golgi v-SNARE	orf19.2940	19943	IPF8114	UNCLASSI	molecular_function unknown
CA2202	0.9	0.9	0.9	SCW4	8298078..8	cell wall glucanase (by	orf19.2941	8122	CaBOS1	CELLULAF	transporter activity
CA2203	0.9	0.9	0.8	DIP51.3F	complemer	dicarboxylic amino aci	orf19.2942	8123	CaSCW4	CLASSIFICATION NOT YET CLEAR-CUT	
CA2204	0.8	1.0	0.7	DIP51.5F	complemer	dicarboxylic amino aci	orf19.2943	10236	CaDIP51.3	Amino acid	transporter activity
CA2205	0.8	0.5	1.0	SEO2	complemer	suppressor of sulfoxyd	orf19.8319	12176	CaDIP51.5	Amino acid metabolism SUBCELLULAR LOCALISATION TRANSPORT FACILITATION	
CA2206	0.8	1.0	0.8	IPF12173	8311220..8	unknown function	orf19.698	12173	CaSEO2	TRANSPO	transporter activity
CA2207	1.1	1.0	1.0	IPF12169	complemer	unknown function	orf19.8316	12169	IPF12173	UNCLASSI	molecular_function unknown
CA2208	1.0	1.0	0.9	IPF17255	complemer	similar to Saccharomy	orf19.696	17255	IPF12169	UNCLASSI	molecular_function unknown
CA2209	0.8	0.8	0.6	IPF18512	8327154..8	unknown function	orf19.695	18512	IPF17255	CELLULAF	signal transducer activity
CA2210	1.1	1.0	1.1	MSL1	complemer	U2 snRNA-associated	orf19.4748	13925	IPF18512	CELLULAF	enzyme regulator activity
CA2211	1.0	1.1	1.0	IPF13921	8337732..8	Unknown function	orf19.4749	13921	CaMSL1	TRANSCR	RNA binding
CA2212	1.0	1.0	1.0	IPF13919	complemer	unknown function	orf19.4750	13919	IPF13921	Lipid fatty-acid and isoprenoid metabolism """"PROTEIN FATE [folding modification destination] ""CELLULAR TRANSPORT AND TRANSF	
CA2213	1.1	1.0	1.0	IPF16405	8342422..8	similar to saccharomy	orf19.4751	16405	IPF13919	UNCLASSIFIED PROTEINS	
CA2214	1.1	1.3	1.0	IPF9939	complemer	similar to Saccharomy	orf19.4752	9939	IPF16405	UNCLASSI	structural molecule activity
CA2215	0.9	1.0	0.8	PFK26	8352093..8	6-phosphofructose-2-k	orf19.4753	9943	IPF9939	C-compour	DNA binding
CA2216	0.7	1.0	0.7	IPF6235	8359756..8	Candida albicans Tca2	orf19.5372	6235	CaPFK26	C-compour	transferase activity
CA2217	0.6	0.5	1.0	POL0	8360866..8	pol polyprotein, revers	orf19.5373	6237	IPF6235	No significant S.c. match	
CA2218	0.9	1.0	1.0	IPF18508	complemer	unknown function	orf19.5375	18508	CaPOL0	UNCLASSIFIED PROTEINS	
CA2219	1.0	1.0	1.0	CAT8	complemer	transcription factor inv	orf19.1256	9674	IPF18508	No significant S.c. match	
CA2220	1.1	1.0	1.0	NTG1	8371878..8	endonuclease III-like g	orf19.5098	9671	CaCAT8	C-compour	transcription regulator activity
CA2221	1.1	1.2	1.0	IPF9670	complemer	membrane transporter	orf19.5100	9670	CaNTG1	CELL CYC	DNA binding
CA2222	1.0	1.0	0.9	CCR4	8379374..8	glucose-repressible alc	orf19.5101	13962	IPF9670	CELL RESCUE DEFENSE AND VIRULENCE ""TRANSPORT FACILITATION	
CA2223	1.1	1.2	1.0	PLB5	complemer	putative phospholipase	orf19.1256	13965	CaCCR4	Amino acid	RNA binding
CA2224	1.0	1.0	0.9	UGA6.3EO	8387122..8	GABA-specific transport protein, 3		19944	CaPLB5	Lipid fatty-acid and isoprenoid metabolism ""Other virulence attributes	
CA2225	1.2	0.7	1.1	SUR2	8389970..8	Hydroxylation of C-4 o	orf19.5818	5688	CaUGA6.3	No significant S.c. match	
CA2226	0.9	0.9	1.0	BET3.EXO	8391526..8	targeting and fusion of	orf19.5817	5686	CaSUR2	Lipid fatty-a	oxidoreductase activity
CA2227	0.9	1.0	0.9	EBP7	complemer	NADPH DEHYDROGE	orf19.5816	5684	CaBET3.ex	CELLULAF	molecular_function unknown
CA2228	1.0	0.9	1.0	SCT12	8394867..8	Suppresses a choline-t	orf19.5815	5683	CaEBP7	ENERGY	
CA2229	0.9	0.9	0.9	IPF5682	8397606..8	unknown function	orf19.5814	5682	CaSCT12	TRANSPORT FACILITATION	
CA2230	1.0	1.0	1.0	IPF10936	8403208..8	unknown function	orf19.3928	10936	IPF5682	No significant S.c. match	
CA2231	1.0	1.0	1.0	IPF10934	complemer	similar to Saccharomy	orf19.3926	10934	IPF10936	TRANSCRIPTION SUBCELLULAR LOCALISATION	
CA2232	1.0	1.0	1.1	IPF10929	8406777..8	unknown function	orf19.3925	10929	IPF10934	Nucleotide	RNA binding
CA2233	1.1	1.0	1.1	IFJ3	8409384..8	unknown function	orf19.3924	10924	IPF10929	CELL CYCLE AND DNA PROCESSING CELL FATE SUBCELLULAR LOCALISATION	
CA2234	1.1	1.0	1.0	IPF10922	8411631..8	unknown function	orf19.3923	10922	CaIFJ3	UNCLASSIFIED PROTEINS	
CA2235	0.8	0.7	1.0	MPD1	complemer	disulfide isomerase rel	orf19.3920	12689	IPF10922	No significant S.c. match	
CA2236	1.0	1.2	1.0	IFA23	8417177..8	unknown function	orf19.3919	12694	CaMPD1	PROTEIN I	oxidoreductase activity,isomerase activity
CA2237	0.9	1.0	0.9	IPF10032.3	complemer	unknown function, 3-pr	orf19.3917	12695	CaIFA23	CELL CYCLE AND DNA PROCESSING SUBCELLULAR LOCALISATION	
CA2238	1.0	0.9	1.1	IPF10032.5	complemer	unknown function, 5-pr	orf19.3916	10032	IPF10032.3	TRANSCRIPTION SUBCELLULAR LOCALISATION	
CA2240	1.0	1.0	1.1	CRD2	8423295..8	Cu-binding metallothionein		17996	IPF10032.5	UNCLASSI	molecular_function unknown

CA2241	1.0	1.0	1.1	IFQ1.3F	complemer	Unknown function, 3-p	orf19.4674	7422	CaCRD2	No significant S.c. match	
CA2242	1.1	1.0	1.1	IFQ1.5F	complemer	Unknown function, 5-p	orf19.4673	7421	CaIFQ1.3f	No significant S.c. match	
CA2243	0.9	1.1	1.0	IPF7414	8431030..8	putative transcription fa	orf19.4670	7414	CaIFQ1.5f	No significant S.c. match	
CA2244	0.0	0.0	0.3	AAT22	complemer	aspartate aminotransfe	orf19.4669	7410	IPF7414	C-compound and carbohydrate metabolism TRANSCRIPTION	
CA2245	1.0	0.9	1.0	IPF7409	complemer	unknown function	orf19.4668	7409	CaAAT22	Amino acid metabolism Nitrogen and sulphur metabolism SUBCELLULAR LOCALISATION	
CA2246	0.9	1.0	1.1	IPF7405	complemer	unknown function	orf19.4666	7405	IPF7409	C-compound and carbohydrate metabolism SUBCELLULAR LOCALISATION	
CA2247	1.1	1.0	1.0	IPF7404	complemer	unknown function	orf19.1213	7404	IPF7405	No significant S.c. match	
CA2248	1.0	1.1	1.0	IPF11469	8445367..8	unknown function	orf19.9902	11469	IPF7404	No significant S.c. match	
CA2249	0.9	0.9	1.0	POL2	complemer	DNA-directed DNA pol	orf19.2365	14265	IPF11469	No significant S.c. match	
CA2250	1.1	1.2	1.1	MIS11	8455731..8	mitochondrial C1-tetral	orf19.2364	12851	CaPOL2	CELL CYC	nucleotidyltransferase activity
CA2251	1.0	0.9	1.0	IPF12845	8459213..8	unknown function	orf19.2363	12845	CaMIS11	Amino acid	ligase activity
CA2252	1.0	1.0	1.0	IPF12844	complemer	unknown function	orf19.9898	12844	IPF12845	PROTEIN I	molecular_function unknown
CA2253	1.0	1.0	1.0	IPF15485	8469219..8	unknown function	orf19.1959	15485	IPF12844	UNCLASSI	molecular_function unknown
CA2254	1.0	1.0	1.1	IPF15487	8470616..8	unknown function	orf19.1958	15487	IPF15485	UNCLASSI	molecular_function unknown
CA2255	0.9	1.1	0.9	CYC3	8477757..8	cytochrome C heme ly	orf19.1957	13303	IPF15487	No significant S.c. match	
CA2256	1.1	0.9	1.0	IPF14757	complemer	unknown function	orf19.1956	14757	CaCYC3	Metabolism	lyase activity
CA2257	1.0	1.0	1.0	SHR5	8481948..8	RAS suppressor (by hc	orf19.1955	14758	IPF14757	UNCLASSI	molecular_function unknown
CA2258	0.9	1.0	1.0	IFR1	8486422..8	Unknown function	orf19.1763	17956	CaSHR5	Nucleotide	transferase activity
CA2259	1.0	0.9	1.1	IPF6845	complemer	unknown function	orf19.1762	6845	CaIFR1	SUBCELLULAR LOCALISATION	
CA2260	1.1	1.0	1.0	OST2	8488973..8	Oligosaccharyltransfer	orf19.1761	6848	IPF6845	UNCLASSI	protein phosphatase activity
CA2261	0.9	1.0	0.9	RAS1	complemer	GTP-binding protein (b	orf19.1760	6849	CaOST2	C-compour	transferase activity
CA2262	1.0	1.0	1.0	IPF6857	8495988..8	putative transcriptional	orf19.1757	6857	CaRAS1	Nucleotide	hydrolase activity
CA2263	1.1	1.1	1.2	GPD1	8500901..8	Glycerol-3-phosphate (	orf19.1756	6860	IPF6857	Amino acid	DNA binding
CA2265	0.9	1.1	1.0	CMK2	complemer	Ca2+/calmodulin-depe	orf19.1754	6866	CaGPD1	C-compour	oxidoreductase activity
CA2266	1.0	1.0	1.0	HEM1	8508965..8	5-aminolevulinic acid s	orf19.1013	2755	CaCMK2	CELLULAF	protein kinase activity
CA2267	1.0	1.1	1.0	IFU1.5F	8511139..8	Unknown function, 5-p	orf19.2600	2757	CaHEM1	Metabolism	transferase activity
CA2268	1.0	1.1	1.0	IFU1.3F	8513229..8	Unknown function, 3-prime end		18497	CaIFU1.5f	CELL CYC	structural molecule activity
CA2269	1.0	0.9	1.0	YOR100	8514491..8	Putative mitochondrial	orf19.2599	2761	CaIFU1.3f	No significant S.c. match	
CA2270	1.3	1.0	0.8	VMA4	8515783..8	H+-transporting ATPas	orf19.2598	2763	CaYOR100	CELLULAF	transporter activity
CA2271	1.0	1.1	1.0	MRS2	8516787..8	Mitochondrial RNA spli	orf19.2597	2765	CaVMA4	PROTEIN I	transporter activity
CA2272	1.0	1.0	0.9	RPA43	complemer	DNA-directed RNA pol	orf19.2594	19945	CaMRS2	TRANSCR	transporter activity
CA2273	1.0	1.0	1.0	BIO2	8520374..8	biotin synthetase (by h	orf19.2593	2771	CaRPA43	TRANSCR	nucleotidyltransferase activity
CA2274	1.0	1.0	1.0	BIO3	complemer	DAPA aminotransferas	orf19.2591	2774	CaBIO2	Metabolism	transferase activity
CA2275	1.0	1.0	1.0	BIO4	8523604..8	dethiobiotin synthetase	orf19.2590	2776	CaBIO3	Metabolism	transferase activity
CA2276	1.0	1.1	1.0	HNM3	8524466..8	Choline permease (by	orf19.2587	2781	CaBIO4	Metabolism	ligase activity
CA2277	1.0	1.0	0.9	DRS21	complemer	Membrane-spanning C	orf19.783	10810	CaHNM3	CELLULAR TRANSPORT AND TRANSPORT MECHANISMS SUBCELLULAR LOCALISATION TRANSPORT FACILITATION	
CA2278	1.0	0.9	1.0	IPF10806	8533188..8	unknown function	orf19.782	10806	CaDRS21	TRANSPO	transporter activity
CA2279	1.0	0.9	1.0	DUR31	8536333..8	Urea transport protein	orf19.781	10804	IPF10806	Lipid fatty-a	molecular_function unknown
CA2280	1.1	1.0	1.1	DUR1,2	8539570..8	urea amidolyase (by hc	orf19.780	9766	CaDUR31	REGULATI	transporter activity
CA2281	1.0	0.9	0.9	HAT1	8545321..8	histone acetyltransfera	orf19.779	9767	CaDUR1,2	Amino acid	hydrolase activity,ligase activity
CA2282	1.0	0.9	1.0	IPF14438	8548850..8	unknown function	orf19.1299	12637	CaHAT1	PROTEIN I	transferase activity
CA2283	1.1	1.0	1.0	MRT4	8549818..8	required for mRNA dec	orf19.1299	12634	IPF14438	No significant S.c. match	
CA2284	0.9	0.9	1.0	MIF2	complemer	required for normal chr	orf19.5551	12632	CaMRT4	Nucleotide	molecular_function unknown
CA2285	1.0	0.9	1.0	IPF12629	complemer	unknown function	orf19.5552	12629	CaMIF2	CELL CYC	DNA binding
CA2286	1.0	0.9	0.9	IPF5369	8555844..8	unknown function	orf19.5553	5369	IPF12629	UNCLASSI	molecular_function unknown
CA2287	0.9	1.0	1.0	IPF5373	complemer	unknown function	orf19.5555	5373	IPF5369	UNCLASSI	molecular_function unknown
CA2288	1.0	0.9	1.0	IPF5376	8563102..8	unknown function	orf19.5557	5376	IPF5373	C-compound and carbohydrate metabolism SUBCELLULAR LOCALISATION	
CA2289	1.2	1.1	1.1	RBF1.3	complemer	RPG-BOX-BINDING F	orf19.1300	5380	IPF5376	PROTEIN FATE [folding modification destination]	
CA2290	0.9	0.9	0.9	IPF16300	complemer	putative aldehyde dehy	orf19.742	16300	CaRBF1.3	No significant S.c. match	
CA2291	1.1	1.4	1.4	IPF9740	complemer	oligo-1,4 -1,4-glucantra	orf19.744	9743	IPF16300	CLASSIFICATION NOT YET CLEAR-CUT	
CA2292	0.8	1.0	0.8	VAC8	8585630..8	required for vacuole inl	orf19.745	9747	IPF9740	C-compour	transferase activity
CA2293	0.9	0.9	0.9	IPF9748	complemer	unknown function	orf19.746	9748	CaVAC8	PROTEIN I	protein binding
CA2294	1.0	0.9	1.0	IPF19946	complemer	similar to Saccharomy	orf19.747	19946	IPF9748	UNCLASSI	molecular_function unknown
CA2295	1.0	1.0	1.1	HOS2	complemer	putative histone deace	orf19.5377	8228	IPF19946	SUBCELLU	hydrolase activity
CA2296	1.1	1.0	1.0	SCL1	8601515..8	Proteasome subunit Y	orf19.5378	8226	CaHOS2	CELL CYC	hydrolase activity
CA2297	1.1	1.0	0.9	ERG4	complemer	sterol C-24 reductase (	orf19.5379	8225	CaSCL1	PROTEIN I	peptidase activity
CA2298	0.9	0.9	1.0	IPF8224	complemer	unknown function	orf19.5380	8224	CaERG4	Lipid fatty-a	oxidoreductase activity
CA2299	1.1	1.0	1.0	IPF8222	complemer	unknown function	orf19.5381	8222	IPF8224	Amino acid metabolism TRANSCRIPTION SUBCELLULAR LOCALISATION UNCLASSIFIED PROTEINS	
CA2300	1.0	1.9	1.0	PMA1	complemer	plasma membrane H+-	orf19.5383	8215	IPF8222	TRANSCR	RNA binding
CA2302	1.1	0.9	1.2	IPF6518	8629553..8	unknown function	orf19.1691	6518	CaPMA1	REGULATI	transporter activity
CA2303	1.3	1.6	1.3	TOS1	complemer	putative Anchor subun	orf19.1690	6517	IPF6518	UNCLASSIFIED PROTEINS	
CA2304	0.9	1.0	1.0	PRP43	8636365..8	RNA-dependent ATPa	orf19.1687	6512	CaTOS1	UNCLASSI	molecular_function unknown
CA2305	1.0	0.9	1.0	MPA43	8639114..8	Unknown function	orf19.1686	6511	CaPRP43	TRANSCR	RNA binding,helicase activity
CA2306	1.0	0.9	1.0	IPF6510	8641469..8	unknown function	orf19.1685	6510	CaMPA43	UNCLASSI	molecular_function unknown
CA2307	1.0	0.9	0.9	IPF18488.5	8642940..8	unknown function, 5-prime end		18488	IPF6510	TRANSCRIPTION SUBCELLULAR LOCALISATION	
CA2308	1.0	0.9	1.0	IPF9874.3	8649277..8	similar to Saccharomy	orf19.8953	9874	IPF18488.5	No significant S.c. match	
CA2309	0.9	1.0	1.0	IPF9875	complemer	unknown function	orf19.1374	9875	IPF9874.3	Lipid fatty-a	hydrolase activity
CA2310	1.0	1.1	1.0	LEU42	complemer	2-isopropylmalalate sy	orf19.1375	9877	IPF9875	PROTEIN FATE [folding modification destination]	
CA2311	0.9	1.0	1.1	SSO2	complemer	syntaxin (by homology	orf19.1376	16993	CaLEU42	Amino acid metabolism SUBCELLULAR LOCALISATION	
CA2312	1.0	1.0	1.0	IPF16498	8658174..8	similar to Saccharomy	orf19.1377	16498	CaSSO2	CELLULAF	transporter activity
CA2313	1.0	1.0	1.0	ERF3	complemer	translation release fact	orf19.1378	19754	IPF16498	Amino acid	transferase activity
CA2314	0.6	0.6	0.7	IPF14545	8662058..8	unknown function	orf19.1381	14545	CaERF3	CELL CYC	translation regulator activity

CA2315	0.9	1.0	1.0	YEA4	complemer	Golgi uridine diphosph	orf19.8962	14544	IPF14545	SUBCELLU	molecular_function unknown
CA2316	0.9	0.9	1.0	IPF14542	8664812..8	unknown function	orf19.8963	14542	CaYEA4	C-compour	transporter activity
CA2317	0.9	1.0	1.0	IPF13586	complemer	unknown function	orf19.5479	13586	IPF14542	No significant S.c. match	
CA2318	1.0	1.0	1.0	ILV1	8672919..8	Threonine dehydratase	orf19.5480	16147	IPF13586	No significant S.c. match	
CA2319	1.0	1.0	1.0	GUF1	complemer	GTP-binding protein (b	orf19.5483	19755	CaILV1	Amino acid	lyase activity
CA2320	1.1	1.1	1.0	SER1	complemer	phosphoserine transam	orf19.5484	10985	CaGUF1	PROTEIN S	hydrolase activity
CA2321	0.9	1.0	1.0	MEC3	complemer	G2-specific checkpoint	orf19.5485	10984	CaSER1	Amino acid	transferase activity
CA2322	0.8	0.9	0.9	YSH1	8679334..8	component of pre-mRN	orf19.5486	10981	CaMEC3	CELL CYC	DNA binding
CA2323	0.8	0.9	0.9	SMD2	8682305..8	U1 snRNP protein of the Sm class	orf19.5487	10980	CaYSH1	TRANSCR	RNA binding
CA2324	1.1	1.0	1.0	CDC46	complemer	cell division control pro	orf19.5487	10979	CaSMD2	TRANSCR	RNA binding
CA2325	1.0	0.9	0.9	IPF10977	8685237..8	unknown function	orf19.5488	10977	CaCDC46	CELL CYC	DNA binding
CA2326	1.0	1.0	1.0	NUBM	8689792..8	nucleotide-binding resp	orf19.1197	4781	IPF10977	CLASSIFIC	molecular_function unknown
CA2327	1.0	1.0	1.1	IPF4782	8692371..8	probable membrane pr	orf19.1197	4782	CaNUBM	No significant S.c. match	
CA2328	1.0	1.0	1.0	MED8	complemer	transcriptional regulatio	orf19.1197	4783	IPF4782	UNCLASSI	molecular_function unknown
CA2329	1.1	1.1	1.1	IPF4784	8694636..8	unknown Function	orf19.4498	4784	CaMED8	TRANSCR	transcription regulator activity
CA2330	0.9	0.8	1.0	RIM2	complemer	mitochondrial carrier pr	orf19.4499	4785	IPF4784	No significant S.c. match	
CA2331	1.0	1.0	1.0	MOT1	complemer	transcriptional accesso	orf19.4502	4789	CaRIM2	ENERGY C	transporter activity
CA2332	1.2	1.0	1.1	IPF4792	complemer	unknown Function	orf19.1197	4792	CaMOT1	TRANSCR	hydrolase activity
CA2333	0.9	0.9	0.9	ADH4	complemer	probable alcohol dehyd	orf19.1198	4794	IPF4792	No significant S.c. match	
CA2334	0.9	1.0	1.0	ADH3	complemer	probable alcohol dehyd	orf19.1198	4795	CaADH4	C-compound and carbohydrate metabolism ENERGY SUBCELLULAR LOCALISATION UNCLASSIFIED PROTEINS	
CA2335	1.0	1.0	1.1	LYS21	complemer	homocitrate synthase (	orf19.1198	18482	CaADH3	C-compound and carbohydrate metabolism ENERGY SUBCELLULAR LOCALISATION UNCLASSIFIED PROTEINS	
CA2336	1.0	1.0	1.1	IPF13383.5	complemer	unknown function, inte	orf19.2353	13383	CaLYS21	Amino acid metabolism C-compound and carbohydrate metabolism SUBCELLULAR LOCALISATION	
CA2337	1.0	1.0	1.0	IPF13379	8716095..8	unknown function	orf19.2352	13379	IPF13383.5	UNCLASSI	molecular_function unknown
CA2338	1.0	0.9	1.0	NIT3	complemer	nitrilase (by homology)	orf19.2351	13378	IPF13379	UNCLASSI	molecular_function unknown
CA2339	1.0	1.1	1.0	IPF13377	complemer	unknown function	orf19.2350	13377	CaNIT3	Nitrogen ar	hydrolase activity
CA2340	0.9	0.9	1.0	MNN5	complemer	Golgi alpha-1,2-manno	orf19.2347	15847	IPF13377	CELL RESCUE DEFENSE AND VIRULENCE ""TRANSPORT FACILITATION	
CA2341	1.0	1.0	1.0	IPF13838	8723633..8	unknown function	orf19.2346	13838	CaMNN5	CELL FATE	
CA2342	1.5	1.5	1.5	IPF13836	complemer	probable heat shock pr	orf19.2344	13836	IPF13838	UNCLASSI	molecular_function unknown
CA2343	1.0	0.9	1.1	IPF8671	8728909..8	unknown function	orf19.2343	8671	IPF13836	No significant S.c. match	
CA2344	1.0	1.0	1.0	SFT2	8730585..8	similar to Saccharomyc	orf19.2342	8674	IPF8671	PROTEIN I	protein binding
CA2345	1.0	1.0	1.1	HNT1	complemer	similarity to protein kin	orf19.2341	8675	CaSFT2	SUBCELLU	molecular_function unknown
CA2346	0.9	0.9	1.0	SEF1	complemer	Putative transcription f	orf19.3753	7790	CaHNT1	Nucleotide	hydrolase activity
CA2347	1.0	1.1	1.0	RAD51	complemer	DNA repair protein by I	orf19.3752	7787	CaSEF1	TRANSCR	molecular_function unknown
CA2348	1.3	1.2	1.2	IPF12811	8745975..8	putative serine/threonin	orf19.3751	12811	CaRAD51	CELL CYC	DNA binding
CA2349	0.9	1.1	1.0	IFC3	complemer	Unknown function	orf19.3749	12812	IPF12811	CLASSIFIC	protein kinase activity
CA2350	0.9	0.9	1.0	CWH41.5E	complemer	ER glucosidase I, 5-pri	orf19.4421	15907	CaIFC3	TRANSPO	transporter activity
CA2351	1.0	0.9	0.9	IPF19947	8754287..8	unknown function	orf19.4420	19947	CaCWH41.	C-compound and carbohydrate metabolism CONTROL OF CELLULAR ORGANIZATION SUBCELLULAR LOCALISATION	
CA2352	0.9	0.9	0.9	FMT1	8757080..8	Methionyl-tRNA Transf	orf19.4418	6482	IPF19947	TRANSCR	transcription regulator activity
CA2353	1.0	1.0	1.0	VPS13	complemer	involved in regulating r	orf19.4416	19756	CaFMT1	TRANSCR	transferase activity
CA2354	1.1	0.9	1.0	IPF18480	complemer	unknown function	orf19.1189	18480	CaVPS13	PROTEIN I	molecular_function unknown
CA2355	0.9	0.8	0.9	TPK1	complemer	cAMP-dependent prote	orf19.1235	12285	IPF18480	No significant S.c. match	
CA2356	0.8	0.8	0.8	IPF12282	complemer	unknown function	orf19.4893	12282	CaTPK1	TRANSCRIPTION REGULATION OF/INTERACTION WITH CELLULAR ENVIRONMENT	
CA2357	0.9	0.7	1.0	IPF8957	complemer	unknown function	orf19.4894	8957	IPF12282	No significant S.c. match	
CA2358	1.1	1.1	1.1	IPF8953	complemer	unknown function	orf19.4895	8953	IPF8957	UNCLASSI	molecular_function unknown
CA2359	0.7	0.5	0.7	IPF8952	complemer	similar to Saccharomyc	orf19.4896	8952	IPF8953	No significant S.c. match	
CA2360	1.1	1.0	1.0	IPF8951	complemer	unknown function	orf19.4897	8951	IPF8952	TRANSCR	nucleotidyltransferase activity
CA2361	0.8	0.9	1.8	IPF8950	complemer	unknown function	orf19.1236	8950	IPF8951	Lipid fatty-a	transporter activity
CA2362	0.5	0.4	0.5	IPF10595	8796680..8	unknown function	orf19.1287	10595	IPF8950	UNCLASSI	molecular_function unknown
CA2363	0.9	0.9	0.9	IPF10590	8804440..8	unknown function	orf19.1285	10590	IPF10595	No significant S.c. match	
CA2364	1.0	0.9	1.0	MEC1	complemer	cell cycle checkpoint p	orf19.8870	19757	IPF10590	UNCLASSI	molecular_function unknown
CA2365	0.9	0.9	0.9	CKS1	complemer	cyclin-dependent kinas	orf19.1282	10371	CaMEC1	CELL CYC	transferase activity
CA2366	1.0	1.0	1.0	IPF10373	complemer	unknown function	orf19.1281	10373	CaCKS1	CELL CYC	enzyme regulator activity
CA2367	1.0	1.4	1.3	SUI1	complemer	translation initiation fac	orf19.8867	10374	IPF10373	UNCLASSI	molecular_function unknown
CA2368	0.8	1.0	1.0	RPA190	8820119..8	DNA-directed RNA pol	orf19.1839	10842	CaSUI1	PROTEIN S	translation regulator activity
CA2369	1.1	1.1	1.0	TBP1	complemer	TATA-binding protein	orf19.1837	10845	CaRPA190	TRANSCR	nucleotidyltransferase activity
CA2370	1.0	0.9	0.9	APN2	complemer	AP endonuclease, exo	orf19.1836	10847	CaTBP1	TRANSCR	DNA binding
CA2371	1.0	1.0	1.0	IPF10333	8830210..8	unknown function	orf19.1835	10333	CaAPN2	CELL CYC	hydrolase activity
CA2372	0.9	1.0	1.0	IPF10335	8832527..8	unknown function	orf19.1834	10335	IPF10333	CELL FATE	protein binding
CA2373	1.0	1.0	1.0	CBF5	complemer	centromere/ microtubu	orf19.1833	10336	IPF10335	CELLULAR TRANSPORT AND TRANSPORT MECHANISMS SUBCELLULAR LOCALISATION	
CA2374	1.0	1.0	1.0	FCY23	complemer	Putative purine-cytosin	orf19.9390	10337	CaCBF5	CELL CYC	lyase activity
CA2375	1.0	1.0	1.0	IPF15504	complemer	unknown function	orf19.2211	15504	CaFCY23	Nucleotide	transporter activity
CA2376	1.0	0.9	1.1	IPF15506	complemer	unknown function	orf19.2213	15506	IPF15504	UNCLASSI	molecular_function unknown
CA2377	1.2	0.9	1.0	MRPL7	complemer	Ribosomal protein of th	orf19.2214	15259	IPF15506	UNCLASSI	molecular_function unknown
CA2378	0.9	1.0	0.9	GLE1	8849231..8	RNA export mediator (	orf19.2215	15261	CaMRPL7	PROTEIN S	structural molecule activity
CA2379	1.1	0.9	0.9	IPF4073	complemer	similar to Saccharomyc	orf19.2216	4073	CaGLE1	TRANSCR	molecular_function unknown
CA2380	1.0	1.1	1.0	IPF4072	8855132..8	unknown function	orf19.2217	4072	IPF4073	CELL CYC	structural molecule activity
CA2381	1.0	1.0	1.0	YCK3.3F	complemer	casein kinase I, 3-prim	orf19.2221	11642	IPF4072	No significant S.c. match	
CA2383	1.0	0.9	1.0	IPF18474	complemer	unknown function		18474	CaYCK3.3f	No significant S.c. match	
CA2384	1.0	0.9	1.0	MDL1	complemer	ATP-DEPENDENT PE	orf19.2615	7801	IPF18474	No significant S.c. match	
CA2385	1.0	1.0	1.0	RSR1.3	complemer	GTP-binding protein, 3	orf19.2614	8507	CaMDL1	TRANSPO	transporter activity
CA2386	1.7	1.4	1.2	ECM41.3	complemer	involved in cell wall bic	orf19.2613	8505	CaRSR1.3	CELL FATE	signal transducer activity
CA2387	1.1	1.1	1.2	IPF8504	8879599..8	unknown function	orf19.2612	8504	CaECM41.	CONTROL	molecular_function unknown

CA2388	1.1	1.1	1.1	MCM6	complemer	component of MCM ini	orf19.2611	8502	IPF8504	TRANSCRIPTION SUBCELLULAR LOCALISATION	
CA2389	0.9	0.9	1.1	IPF8500	8883552..8	unknown function	orf19.2610	8500	CaMCM6	CELL CYC	DNA binding
CA2390	1.0	1.0	0.9	CET1	8884285..8	mRNA 5 -triphosphata	orf19.2609	8499	IPF8500	TRANSCRIPTION SUBCELLULAR LOCALISATION	
CA2391	2.2	3.6	1.9	ADH5	8887692..8	probable alcohol dehy	orf19.2608	15671	CaCET1	TRANSCR	hydrolase activity
CA2392	1.1	1.1	1.0	IPF15672	complemer	unknown function	orf19.1013	15672	CaADH5	C-compound and carbohydrate metabolism ENERGY SUBCELLULAR LOCALISATION	
CA2393	1.0	1.0	1.0	RHO3	complemer	GTP-binding protein of	orf19.1101	6422	IPF15672	ENERGY	
CA2394	1.0	1.0	1.1	IPF6424	complemer	unknown function	orf19.1101	6424	CaRHO3	CELL FATI	signal transducer activity
CA2395	1.0	1.0	1.0	IPF6425	complemer	unknown function	orf19.3536	6425	IPF6424	UNCLASSIFIED PROTEINS	
CA2396	0.9	1.0	1.0	IPF6428	complemer	unknown function	orf19.3537	6428	IPF6425	UNCLASSI	transferase activity
CA2397	1.1	0.9	1.2	CFL12	8896757..8	Strong similarity to ferr	orf19.3538	6430	IPF6428	UNCLASSI	molecular_function unknown
CA2398	1.0	0.9	0.9	IPF6431	complemer	unknown function	orf19.3539	6431	CaCFL12	REGULATION OF/INTERACTION WITH CELLULAR ENVIRONMENT Other virulence attributes	
CA2399	0.9	0.9	0.8	MAK5	8900492..8	ATP-dependent RNA h	orf19.3540	6433	IPF6431	UNCLASSI	molecular_function unknown
CA2400	1.0	1.0	1.0	SUP45	complemer	Translational release fa	orf19.3541	6434	CaMAK5	TRANSCR	RNA binding,helicase activity
CA2401	1.1	1.1	1.0	LEM3	8904584..8	cell division cycle muta	orf19.3542	6435	CaSUP45	PROTEIN S	translation regulator activity
CA2402	1.0	0.9	1.0	IPF6437	complemer	unknown function	orf19.3543	6437	CaLEM3	UNCLASSI	transcription regulator activity
CA2403	1.0	0.9	0.9	IPF6438	8906519..8	unknown function	orf19.3544	6438	IPF6437	No significant S.c. match	
CA2405	1.0	1.0	1.0	IPF3844	8915133..8	unknown function	orf19.301	3844	IPF6438	Lipid fatty-a	molecular_function unknown
CA2406	0.9	1.0	0.9	AIP2	complemer	actin interacting protein	orf19.300	3840	IPF3844	No significant S.c. match	
CA2407	1.1	1.4	0.9	ECM14	8920404..8	carboxypeptidase invo	orf19.299	3838	CaAIP2	SUBCELLU	oxidoreductase activity
CA2408	1.0	1.1	1.0	IPF3833	complemer	unknown function	orf19.296	3833	CaECM14	CONTROL	molecular_function unknown
CA2409	0.9	1.0	1.0	IPF19948	8925564..8	unknown function	orf19.291	19948	IPF3833	No significant S.c. match	
CA2411	0.9	0.9	0.9	IFI3.3	complemer	Unknown function, 3-p	orf19.4483	17915	IPF19948	UNCLASSI	molecular_function unknown
CA2412	1.0	1.0	1.0	IPF13755	8932229..8	unknown function	orf19.4481	13755	CaIFI3.3	CLASSIFICATION NOT YET CLEAR-CUT	
CA2413	0.8	0.8	0.8	IPF13756.5	8933487..8	unknown function, 5-pr	orf19.4480	13756	IPF13755	No significant S.c. match	
CA2414	1.0	1.1	0.9	IPF13756.3	8934104..8	unknown function, 3-pr	orf19.4479	13757	IPF13756.5	UNCLASSIFIED PROTEINS	
CA2415	1.0	1.0	1.0	MSD1	complemer	Aspartyl-tRNA syntheta	orf19.4478	17203	IPF13756.3	UNCLASSI	molecular_function unknown
CA2416	2.6	4.4	3.3	IFD4	8938685..8	Putative aryl-alcohol de	orf19.4477	14374	CaMSD1	PROTEIN S	ligase activity
CA2418	1.0	1.0	0.9	MNT4	8942665..8	putative mannosyltrans	orf19.4475	14377	CaIFD4	C-compound and carbohydrate metabolism ENERGY	
CA2419	1.0	0.9	1.1	IPF14379	complemer	unknown function	orf19.4474	14379	CaMNT4	C-compour	transferase activity
CA2420	1.0	1.0	1.0	SPC19	complemer	spindle pole body prote	orf19.4473	15002	IPF14379	UNCLASSI	molecular_function unknown
CA2421	0.9	0.8	0.9	IPF14998	8947627..8	unknown function	orf19.4471	14998	CaSPC19	SUBCELLU	structural molecule activity
CA2422	0.9	0.7	0.9	IPF14997	8950699..8	unknown function	orf19.1195	14997	IPF14998	UNCLASSI	molecular_function unknown
CA2423	1.0	1.1	1.0	SDH41	complemer	succinate dehydrogena	orf19.1194	19574	IPF14997	No significant S.c. match	
CA2424	0.9	1.0	1.0	IPF18468	complemer	unknown function	orf19.1194	18468	CaSDH41	C-compour	oxidoreductase activity
CA2425	1.0	1.1	1.0	IPF9907	8956615..8	similar to Saccharomy	orf19.1091	9907	IPF18468	No significant S.c. match	
CA2426	1.0	1.0	0.9	IPF9901	8959480..8	similar to Saccharomy	orf19.3407	9901	IPF9907	CELLULAF	molecular_function unknown
CA2427	1.0	1.0	1.0	IPF9898	complemer	probable formate dehy	orf19.3406	9898	IPF9901	CELL CYC	DNA binding
CA2428	1.0	1.0	0.9	IPF9894.3	complemer	unknown function, 3-pr	orf19.3405	9894	IPF9898	ENERGY	transporter activity
CA2429	1.0	1.0	1.0	IPF9890	8965711..8	unknown function	orf19.3404	9890	IPF9894.3	No significant S.c. match	
CA2430	1.0	0.9	0.9	IPF12950	complemer	unknown function	orf19.3402	12950	IPF9890	UNCLASSIFIED PROTEINS	
CA2431	1.0	1.0	1.2	IPF12951	8968605..8	unknown function	orf19.3401	12951	IPF12950	No significant S.c. match	
CA2432	1.0	0.9	1.0	COQ3	complemer	3,4-dihydroxy-5-hexap	orf19.3400	12953	IPF12951	UNCLASSI	molecular_function unknown
CA2433	0.9	0.9	1.0	IPF12959	8970442..8	unknown function	orf19.3399	12959	CaCOQ3	Metabolism	transferase activity
CA2434	1.0	1.0	1.0	IPF9484	complemer	unknown function	orf19.3396	9484	IPF12959	UNCLASSI	molecular_function unknown
CA2435	1.0	0.9	0.9	IPF9483	complemer	probable permease (by	orf19.1089	9483	IPF9484	UNCLASSIFIED PROTEINS	
CA2436	1.0	0.9	1.0	IPF5500	8979991..8	unknown function	orf19.2200	5500	IPF9483	CELL RES	transporter activity
CA2437	1.0	1.0	1.1	PHO86	complemer	inorganic phospate tra	orf19.2199	5501	IPF5500	UNCLASSI	molecular_function unknown
CA2438	0.9	0.9	1.1	IPF5505	8983829..8	unknown function	orf19.2198	5505	CaPHO86	Phosphate	molecular_function unknown
CA2439	0.9	0.9	0.9	IPF7514	8986955..8	unknown function	orf19.2197	7514	IPF5505	UNCLASSIFIED PROTEINS	
CA2440	1.0	1.1	1.0	IPF7513	8989114..8	unknown function	orf19.2196	7513	IPF7514	Nitrogen and sulphur metabolism	
CA2441	1.0	0.8	0.9	APM4	8990760..8	AP-2 complex subunit,	orf19.2194	7509	IPF7513	UNCLASSI	molecular_function unknown
CA2442	0.9	0.9	1.0	PRS5	8992474..8	Phosphoribosylpyroph	orf19.2193	7507	CaAPM4	PROTEIN I	molecular_function unknown
CA2443				YAF9	8997489..8998253				CaPRS5	Amino acid	transferase activity
CA2444	0.9	1.0	0.9	IPF13883	complemer	Unknown function	orf19.5502	13883			
CA2445	1.1	1.0	1.0	IPF13885	8999951..9	unknown function	orf19.5503	13885	IPF13883	No significant S.c. match	
CA2446	0.9	0.8	1.0	IPF2582	9002148..9	unknown function	orf19.5504	2582	IPF13885	No significant S.c. match	
CA2447	1.1	1.2	1.0	HIS7	9005393..9	Histidine biosynthesis	orf19.5505	2583	IPF2582	UNCLASSI	molecular_function unknown
CA2448	0.9	0.9	1.0	PLC1	9007674..9	1-phosphatidylinositol-	orf19.5506	2586	CaHIS7	Amino acid	transferase activity
CA2449	0.9	0.7	0.9	ENP1	9011266..9	Essential nuclear prote	orf19.5507	2587	CaPLC1	Lipid fatty-a	hydrolase activity
CA2450	1.0	1.0	1.0	IPF2589	complemer	unknown function	orf19.5508	2589	CaENP1	PROTEIN I	RNA binding
CA2451	1.0	1.0	0.9	IFB2	complemer	unknown function	orf19.5509	6648	IPF2589	UNCLASSIFIED PROTEINS	
CA2452	2.1	1.5	2.1	IPF9955	complemer	unknown function	orf19.1099	9955	CaIFB2	TRANSPORT FACILITATION	
CA2453	0.9	1.0	0.9	IPF9950	9026533..9	unknown function	orf19.1099	9950	IPF9955	No significant S.c. match	
CA2454	1.2	1.2	1.2	RPL23B.3	9030804..9	ribosomal protein L23.	orf19.3504	9946	IPF9950	UNCLASSI	molecular_function unknown
CA2455	1.0	1.0	0.9	IPF12228	complemer	unknown function	orf19.3505	12228	CaRPL23B	PROTEIN S	structural molecule activity
CA2456	1.0	1.0	1.0	DBR1	complemer	lariat-debranching enz	orf19.3506	12230	IPF12228	UNCLASSI	molecular_function unknown
CA2457	1.7	1.7	1.4	MCR1	complemer	NADH-cytochrome-b5	orf19.3507	12231	CaDBR1	Nucleotide	RNA binding
CA2458	1.0	1.0	1.0	IPF12233	9038767..9	unknown function	orf19.3508	12233	CaMCR1	ENERGY S	transporter activity
CA2459	1.0	0.9	1.0	IPF12234.5	9040121..9	unknown function, 5-pr	orf19.3509	12234	IPF12233	UNCLASSI	molecular_function unknown
CA2460	1.0	1.0	1.1	LYS1.3EO	9040950..9	Saccharopine dehydrogenase, 3-p		18465	IPF12234.5	No significant S.c. match	
CA2461	1.0	1.1	0.9	MAK11	complemer	involved in cell growth	orf19.1791	8738	CaLYS1.3	Amino acid metabolism SUBCELLULAR LOCALISATION	
CA2462	1.0	1.0	0.9	CDC16	9042988..9	subunit of anaphase-p	orf19.1792	8736	CaMAK11	CELL FATI	molecular_function unknown

CA2463	1.0	1.0	1.1	IPF8730	9048173..9	unknown function	orf19.1793	8730	CaCDC16	CELL CYC protein binding
CA2464	1.0	1.0	1.0	IPF8727	complemer	unknown function	orf19.1794	8727	IPF8730	UNCLASSI molecular_function unknown
CA2465	1.0	1.0	1.0	IPF8726	9052094..9	unknown function	orf19.1795	8726	IPF8727	No significant S.c. match
CA2466	1.0	1.0	1.0	IPF8725	complemer	unknown function		8725	IPF8726	Nucleotide RNA binding
CA2467	0.9	0.9	1.0	IPF8724	9055895..9	unknown function	orf19.1796	8724	IPF8725	No significant S.c. match
CA2468	0.9	0.9	1.0	IPF8723	complemer	unknown function	orf19.1797	8723	IPF8724	C-compour oxidoreductase activity
CA2469	1.0	1.0	1.0	IPF7393	complemer	unknown function	orf19.9364	7393	IPF8723	No significant S.c. match
CA2470	0.9	1.0	1.1	SDH12	9069926..9	Succinate dehydrogen	orf19.1038	3247	IPF7393	UNCLASSIFIED PROTEINS
CA2471	1.1	1.1	1.1	TOP2	complemer	Topoisomerase II	orf19.2873	3245	CaSDH12	C-compound and carbohydrate metabolism ENERGY SUBCELLULAR LOCALISATION
CA2472	1.0	1.0	1.0	IPF3239	complemer	unknown function	orf19.2875	3239	CaTOP2	CELL CYC isomerase activity
CA2473	0.9	1.0	1.0	CBF1	complemer	putative centromere bi	orf19.2876	3238	IPF3239	PROTEIN FATE [folding modification destination] ""CELLULAR TRANSPORT AND TRANSPORT MECHANISMS SUBCELLULAR LOCAL
CA2474	5.1	3.2	3.0	PDC11	9080267..9	Pyruvate decarboxylas	orf19.2877	3234	CaCBF1	Amino acid DNA binding
CA2475	1.0	1.1	1.0	IPF3233	complemer	unknown function	orf19.2878	3233	CaPDC11	C-compour lyase activity
CA2476	0.9	1.0	0.9	IFF5	9085007..9	unknown function	orf19.1039	15521	IPF3233	No significant S.c. match
CA2477	1.0	1.1	0.9	IPF16016	complemer	unknown function	orf19.1039	16016	CaIFF5	C-compound and carbohydrate metabolism SUBCELLULAR LOCALISATION
CA2478	1.0	1.0	0.9	DAL52	9098997..9	allantoate permease (t	orf19.3208	14177	IPF16016	UNCLASSIFIED PROTEINS
CA2479	1.1	0.9	1.0	CCN1	complemer	G1 cyclin	orf19.3207	15597	CaDAL52	CELLULAR TRANSPORT AND TRANSPORT MECHANISMS SUBCELLULAR LOCALISATION TRANSPORT FACILITATION
CA2480	1.0	1.1	1.0	CCT7	complemer	component of chapero	orf19.1071	9837	CaCCN1	CELL CYCLE AND DNA PROCESSING ""CELL RESCUE DEFENSE AND VIRULENCE ""CELL FATE SUBCELLULAR LOCALISATION
CA2481	1.0	0.9	1.0	MRPL36	9107958..9	ribosomal protein YmL	orf19.3205	9838	CaCCT7	PROTEIN I chaperone activity
CA2482	1.0	0.9	0.9	IPF9841	complemer	unknown function	orf19.3204	9841	CaMRPL36	PROTEIN S structural molecule activity
CA2483	1.0	1.1	1.0	IPF19758	complemer	unknown function	orf19.3203	19758	IPF9841	CELL CYCLE AND DNA PROCESSING CONTROL OF CELLULAR ORGANIZATION SUBCELLULAR LOCALISATION
CA2484	1.0	1.0	1.0	IPF9846	9114239..9	unknown function	orf19.1071	9846	IPF19758	UNCLASSI protein binding
CA2485	1.0	1.0	1.0	PAP12	9117478..9	poly(A) polymerase	orf19.1071	9850	IPF9846	UNCLASSIFIED PROTEINS
CA2486	1.0	0.9	0.9	IPF9851	complemer	unknown function	orf19.1071	9851	CaPAP12	TRANSCRIPTION SUBCELLULAR LOCALISATION
CA2487	0.8	0.8	0.9	PIK1	complemer	phosphatidylinositol 4-l	orf19.1071	9853	IPF9851	TRANSCR transcription regulator activity
CA2488	0.9	1.0	0.9	IPF17706	9125155..9	Unknown function	orf19.1070	17706	CaPIK1	Lipid fatty-a transferase activity
CA2489	1.1	1.2	0.9	IPF7217	complemer	unknown function	orf19.1152	7217	IPF17706	No significant S.c. match
CA2490	1.0	1.1	1.1	MUM2	complemer	ubiquitin C-terminal hy	orf19.4044	7220	IPF7217	TRANSCRIPTION
CA2491	1.0	1.0	1.0	IPF7221	complemer	unknown function	orf19.4046	7221	CaMUM2	CELL CYC molecular_function unknown
CA2492	0.7	0.5	0.9	IPF7224	9132913..9	putative telomere elong	orf19.4045	7224	IPF7221	No significant S.c. match
CA2493	1.5	1.2	1.2	IPF7227	9134845..9	putative fatty acid desa	orf19.4048	7227	IPF7224	CELL CYC molecular_function unknown
CA2494	1.0	1.2	1.0	HTS1	complemer	histidine tRNA syntheta	orf19.4051	7230	IPF7227	Lipid fatty-acid and isoprenoid metabolism """"CELL RESCUE DEFENSE AND VIRULENCE ""REGULATION OF/INTERACTION WITH CE
CA2495	0.9	1.0	1.0	CTA24	complemer	transcriptional regulatio	orf19.4054	7233	CaHTS1	PROTEIN S ligase activity
CA2496	1.2	1.0	1.1	IPF3468	complemer	unknown function	orf19.4055	3468	CaCTA24	No significant S.c. match
CA2497	0.9	1.0	1.0	TEL1.3EO	9147927..9	Putative phosphatidylir	orf19.5580	15888	IPF3468	No significant S.c. match
CA2498	1.0	1.0	1.0	VPS181	9153846..9	vacuolar membrane pr	orf19.5584	19951	CaTEL1.3e	Lipid fatty-a transferase activity
CA2499	0.9	1.0	0.9	SAP5	9158571..9	secreted aspartyl prote	orf19.5585	6816	CaVPS181	PROTEIN I protein binding
CA2500	1.0	1.0	1.0	FIG4	complemer	suppressor of sac1 mu	orf19.5586	6814	CaSAP5	PROTEIN FATE [folding modification destination] ""Other virulence attributes
CA2501	1.0	0.9	0.9	IPF6812	complemer	unknown function	orf19.5587	6812	CaFIG4	CELLULAF hydrolase activity
CA2502	1.0	1.0	1.0	IPF20103	9166929..9	unknown function	orf19.5588	20103	IPF6812	UNCLASSI protein binding
CA2503	1.0	1.0	0.9	IPF6803.5f	9171868..9	unknown function, 5-pr	orf19.5592	6803	IPF20103	No significant S.c. match
CA2504	1.0	0.9	0.9	IPF6803.3f	9173034..9	unknown function, 3-pr	orf19.5593	6801	IPF6803.5f	UNCLASSIFIED PROTEINS
CA2505	1.2	1.1	1.1	IPF6796	9175569..9	unknown function	orf19.5595	6796	IPF6803.3f	No significant S.c. match
CA2506	1.0	0.9	1.0	IPF6794	complemer	unknown function	orf19.5596	6794	IPF6796	CELL FATI RNA binding
CA2507	1.0	0.9	1.0	IPF18459.3	9179867..9	aldehyde dehydrogenase, 3-prime		18459	IPF6794	TRANSCRIPTION
CA2508	1.0	1.0	1.1	BET5	9181124..9	targeting and fusion of	orf19.302	1468	IPF18459.3	CLASSIFICATION NOT YET CLEAR-CUT
CA2509	1.1	1.1	1.0	IPF1471	9182324..9	aminotriazole resistanc	orf19.304	1471	CaBET5	CELLULAF molecular_function unknown
CA2510	1.0	0.9	1.0	IPF1472	9184056..9	unknown function	orf19.305	1472	IPF1471	CELL RES molecular_function unknown
CA2511	1.0	0.9	1.0	IPF1474	9185635..9	myosin-like protein	orf19.306	1474	IPF1472	TRANSPORT FACILITATION
CA2512	1.1	1.0	1.0	HFI1	complemer	Putative transcriptional	orf19.307	1476	IPF1474	CELLULAR TRANSPORT AND TRANSPORT MECHANISMS SUBCELLULAR LOCALISATION
CA2513	1.0	1.0	0.9	SNG1	9190417..9	Involved in nitroguanid	orf19.308	1478	CaHFI1	TRANSCR transcription regulator activity
CA2514	0.9	0.9	1.0	FUR4	complemer	Probable uracile or alla	orf19.313	1484	CaSNG1	CELL RES molecular_function unknown
CA2515	0.9	1.1	0.9	IPF19759	9200705..9	Unknown Function	orf19.5510	19759	CaFUR4	CELLULAF transporter activity
CA2516	1.0	1.0	0.9	IPF4301	complemer	unknown function	orf19.5513	4310	IPF19759	TRANSCR molecular_function unknown
CA2517	0.9	0.9	0.9	IPF4311	9205609..9	unknown function	orf19.5514	4311	IPF4301	TRANSCR transcription regulator activity
CA2518	0.9	1.0	1.0	CBP3	complemer	involved in cytochrome	orf19.5515	4312	IPF4311	No significant S.c. match
CA2519	0.9	0.9	0.9	SRP72	9207788..9	signal recognition parti	orf19.5516	4313	CaCBP3	PROTEIN I molecular_function unknown
CA2520	1.1	1.1	1.1	IPF20104	9211001..9	alcohol dehydrogenase	orf19.5517	20104	CaSRP72	PROTEIN FATE [folding modification destination] ""SUBCELLULAR LOCALISATION
CA2521	1.1	1.1	1.2	IPF4317	complemer	unknown function	orf19.5518	4317	IPF20104	C-compour oxidoreductase activity
CA2522	0.7	0.7	0.8	GCV1	complemer	glycine cleavage T pro	orf19.5519	4318	IPF4317	No significant S.c. match
CA2523	1.0	1.0	1.0	IPF4319	complemer	unknown function	orf19.5520	4319	CaGCV1	Amino acid oxidoreductase activity
CA2524	0.8	0.7	0.6	IPF4322	9217494..9	unknown function	orf19.5521	4322	IPF4319	No significant S.c. match
CA2525	0.9	1.0	1.1	IPF4324.3	complemer	unknown function, 3-pr	orf19.5522	4324	IPF4322	REGULATI molecular_function unknown
CA2526	1.0	1.1	1.0	SEC20	complemer	secretory pathway prot	orf19.5526	4329	IPF4324.3	No significant S.c. match
CA2527	1.0	1.1	1.0	IPF4331	9223521..9	unknown function	orf19.5527	4331	CaSEC20	PROTEIN I transporter activity
CA2528	1.0	1.0	1.1	MOB1	complemer	required for completion	orf19.5528	4332	IPF4331	ENERGY RNA binding
CA2530	1.0	1.1	1.1	IPF12782	9229267..9	unknown function	orf19.3176	12782	CaMOB1	CELL CYC enzyme regulator activity
CA2531	1.0	1.2	1.1	RIB2	complemer	DRAP deaminase (by l	orf19.3177	12784	IPF12782	UNCLASSI molecular_function unknown
CA2532	1.0	1.0	1.0	PRP9	complemer	pre-mRNA splicing fac	orf19.3178	20105	CaRIB2	Metabolism lyase activity
CA2533	0.9	1.1	1.0	IPF12790	9234610..9	inositol polyphosphate	orf19.3180	12790	CaPRP9	TRANSCR RNA binding
CA2534	1.2	1.2	1.0	IPF12793	complemer	similar to Saccharomy	orf19.3182	12793	IPF12790	Lipid fatty-a hydrolase activity
CA2535	1.0	1.0	1.0	NCE11	9237807..9	involved in non-classical protein ex		20106	IPF12793	TRANSCR DNA binding,transcription regulator activity

CA2536	1.0	1.1	1.0	IPF20107	complemer	unknown function	orf19.3183	20107	CaNCE11	CELLULAR TRANSPORT AND TRANSPORT MECHANISMS
CA2537	1.0	1.0	1.0	IPF20108	9238983..9	similar to Saccharomy	orf19.3184	20108	IPF20107	PROTEIN I molecular_function unknown
CA2538	0.9	1.1	0.9	NAT1	complemer	Protein N-acetyltransfe	orf19.3185	15324	IPF20108	SUBCELLULAR LOCALISATION
CA2539	1.2	1.2	1.1	IPF9188	9243131..9	unknown function	orf19.3187	9188	CaNAT1	Lipid fatty-a transferase activity
CA2541	1.0	1.1	1.0	IPF9191.3f	9247480..9	unknown function, 3-pr	orf19.3188	9191	IPF9188	TRANSCRIPTION ""CELL RESCUE DEFENSE AND VIRULENCE ""SUBCELLULAR LOCALISATION
CA2542	0.8	0.9	0.8	IPF16067	9251849..9	unknown function	orf19.3190	16067	IPF9191.3f	TRANSCRIPTION ""CELL RESCUE DEFENSE AND VIRULENCE ""SUBCELLULAR LOCALISATION
CA2543	1.0	1.0	1.0	IPF16925	9257179..9	unknown function	orf19.2828	16925	IPF16067	TRANSCR transcription regulator activity
CA2544	0.9	0.9	1.0	IPF16924	9258342..9	unknown function	orf19.2829	16924	IPF16925	PROTEIN I chaperone activity
CA2545	1.0	1.0	1.0	RRP9	9259154..9	U3 small nucleolar ribo	orf19.2830	15036	IPF16924	UNCLASSI molecular_function unknown
CA2546	1.0	1.0	1.0	RPC31	complemer	DNA-directed RNA pol	orf19.2831	15034	CaRRP9	TRANSCR RNA binding
CA2547	1.0	1.0	1.0	IPF14911	9265854..9	unknown function	orf19.2832	14911	CaRPC31	TRANSCR nucleotidyltransferase activity
CA2548	1.1	1.0	0.9	IPF9101	complemer	unknown function	orf19.2833	9101	IPF14911	UNCLASSI molecular_function unknown
CA2549	1.1	1.1	1.1	RPD31	complemer	histone deacetylase B	orf19.2834	9100	IPF9101	No significant S.c. match
CA2550	0.9	1.2	1.0	IPF9099	complemer	similar to Saccharomy	orf19.2835	9099	CaRPD31	Phosphate metabolism CELL CYCLE AND DNA PROCESSING TRANSCRIPTION CELL FATE SUBCELLULAR LOCALISATION
CA2551	1.0	1.0	1.0	IPF9098	9275206..9	unknown function	orf19.2836	9098	IPF9099	PROTEIN FATE [folding modification destination]
CA2552	1.1	1.1	1.0	ALG5	complemer	dolichol-P-glucose syn	orf19.2837	9097	IPF9098	No significant S.c. match
CA2553	1.0	1.0	1.0	IPF9096	complemer	probable mannosidase	orf19.2838	9096	CaALG5	C-compour transferase activity
CA2554	0.9	1.0	1.0	CIRT4B	9280201..9	probable transposase	orf19.2839	9095	IPF9096	No significant S.c. match
CA2555	1.1	1.0	0.9	IPF12105	complemer	unknown function	orf19.5633	12105	CaCirt4b	CELL CYCLE AND DNA PROCESSING SUBCELLULAR LOCALISATION
CA2556	1.3	1.5	1.2	FRE5	complemer	ferric reductase transm	orf19.5634	12102	IPF12105	C-compound and carbohydrate metabolism ENERGY SUBCELLULAR LOCALISATION
CA2557	1.4	1.7	1.3	IPF12101	9287725..9	mycelial surface antige	orf19.5635	12101	CaFRE5	REGULATION OF/INTERACTION WITH CELLULAR ENVIRONMENT Other virulence attributes
CA2558	1.5	1.5	1.4	RBT5	9289637..9	repressed by TUP1 pro	orf19.5636	19952	IPF12101	No significant S.c. match
CA2559	0.9	0.9	1.1	HIS4	complemer	Histidine biosynthesis	orf19.5639	12359	CaRBT5	No significant S.c. match
CA2560	1.0	1.0	1.0	PEX5	complemer	peroxisomal targeting s	orf19.5640	12357	CaHIS4	Amino acid oxidoreductase activity
CA2561	0.9	1.0	1.0	CAR2	complemer	ornithine aminotransfe	orf19.5641	12355	CaPEX5	PROTEIN I signal transducer activity
CA2562	1.3	2.1	1.3	IPF19953	9297454..9	unknown function	orf19.5642	19953	CaCAR2	Amino acid transferase activity
CA2563	0.9	1.0	1.1	ECM7	9298071..9	cell wall biogenesis an	orf19.5643	12129	IPF19953	No significant S.c. match
CA2564	0.8	0.9	0.9	IPF12127	9299670..9	unknown function	orf19.5644	12127	CaECM7	CONTROL molecular_function unknown
CA2565	1.1	1.8	1.3	MET15	complemer	O-acetylhomoserine O	orf19.5645	12125	IPF12127	UNCLASSI molecular_function unknown
CA2566	1.0	0.9	0.9	IPF12122	9302509..9	unknown function	orf19.5646	12122	CaMET15	Amino acid transferase activity
CA2567	1.0	1.1	0.9	SUB2.3F	complemer	Involved in pre-mRNA	orf19.5647	12120	IPF12122	UNCLASSI molecular_function unknown
CA2569	1.0	0.8	0.9	IPF12117	complemer	unknown function	orf19.5648	12117	CaSUB2.3f	TRANSCR protein binding
CA2570	1.0	1.0	0.9	PRO3	complemer	delta 1-pyrroline-5-carb	orf19.5650	12114	IPF12117	No significant S.c. match
CA2571	0.9	0.9	0.9	IPF13769	9309333..9	unknown function	orf19.1359	13769	CaPRO3	Amino acid oxidoreductase activity
CA2572	1.1	1.1	1.0	IPF13766	9311002..9	unknown function	orf19.6211	13766	IPF13769	TRANSPO transporter activity
CA2573	0.9	1.0	0.9	SUI2	complemer	translation initiation fac	orf19.6213	13764	IPF13766	CELL CYCLE AND DNA PROCESSING ""PROTEIN FATE [folding modification destination] ""SUBCELLULAR LOCALISATION
CA2574	1.0	0.9	1.0	ATH1	9315495..9	acid trehalase, vacuola	orf19.6214	19760	CaSUI2	PROTEIN S translation regulator activity
CA2575	1.0	0.9	1.0	SEC31	complemer	Component of the COF	orf19.6217	7439	CaATH1	C-compour hydrolase activity
CA2576	1.0	0.9	1.1	IPF7432	9323625..9	unknown function	orf19.6219	7432	CaSEC31	CELLULAF structural molecule activity
CA2577	1.0	1.0	1.0	IPF7430	9325294..9	unknown function	orf19.6220	7430	IPF7432	No significant S.c. match
CA2578	1.2	1.4	1.1	MMD1.3	9328479..9	Maintenance of mitochondrial DNA		7427	IPF7430	UNCLASSI molecular_function unknown
CA2579	1.0	2.6	1.1	RPL34B.3	9329645..9	Ribosomal protein L34.e, 3-prime		7424	CaMMD1.3	CONTROL molecular_function unknown
CA2581	1.1	1.0	1.0	IPF9450	9330479..9	unknown function	orf19.4370	9450	CaRPL34B	PROTEIN S structural molecule activity
CA2582	3.2	1.5	3.2	TAL1	9335898..9	transaldolase (by homo	orf19.4371	9448	IPF9450	No significant S.c. match
CA2583	1.0	1.0	1.1	IPF9445	complemer	unknown function	orf19.4372	9445	CaTAL1	C-compour transferase activity
CA2584	0.9	1.0	1.0	IPF9440	complemer	similar to Saccharomy	orf19.4373	9440	IPF9445	TRANSPORT FACILITATION
CA2585	1.0	1.0	0.9	IPF9438	complemer	similar to Saccharomy	orf19.4374	9438	IPF9440	CLASSIFIC transferase activity
CA2586	0.9	1.2	1.0	IPF9435	9343175..9	unknown function	orf19.4375	9435	IPF9438	TRANSCR RNA binding
CA2587	1.3	1.4	1.3	RPS30.3	complemer	40S ribosomal protein S30, 3-prim		15100	IPF9435	UNCLASSI transferase activity
CA2588	1.0	0.9	1.0	IPF15098	complemer	Unknown function	orf19.4376	15098	CaRPS30.3	PROTEIN S structural molecule activity
CA2589	1.1	1.0	1.1	KRE1	complemer	secretory pathway prot	orf19.4377	13702	IPF15098	No significant S.c. match
CA2590	1.0	0.9	1.0	PPH3.3B	complemer	protein serine/threonin	orf19.4378	12918	CaKRE1	No significant S.c. match
CA2591	0.9	1.0	0.9	IPF18448.3	9357618..9	unknown function, 3-pr	orf19.60	18448	CaPPH3.3I	CELL FATE
CA2592	1.0	1.0	1.0	IPF18447	complemer	putative zinc-finger pro	orf19.59	18447	IPF18448.3	SUBCELLULAR LOCALISATION UNCLASSIFIED PROTEINS
CA2593	1.0	1.1	1.0	RRP6	9359514..9	involved in 5.8S rRNA	orf19.58	12827	IPF18447	UNCLASSI molecular_function unknown
CA2594	0.9	1.0	1.0	IPF12824	9362616..9	unknown function	orf19.57	12824	CaRRP6	UNCLASSI RNA binding
CA2595	1.1	1.1	1.0	ARG2	9363451..9	acetylglutamate syntha	orf19.56	12823	IPF12824	UNCLASSI DNA binding
CA2596	1.1	1.0	1.0	IPF19546	9365758..9	unknown function	orf19.55	19546	CaARG2	Amino acid transferase activity
CA2597	1.1	1.0	1.1	IFQ3	complemer	unknown function	orf19.54	13148	IPF19546	UNCLASSI molecular_function unknown
CA2598	1.1	1.0	1.1	IPF13142	9371688..9	unknown function	orf19.53	13142	CaIFQ3	No significant S.c. match
CA2599	1.1	1.0	1.3	IPF11176	9373658..9	similar to Saccharomy	orf19.52	11176	IPF13142	No significant S.c. match
CA2600	1.0	1.0	1.1	IPF11177	complemer	similar to Saccharomy	orf19.51	11177	IPF11176	CELLULAF molecular_function unknown
CA2601	1.0	1.0	1.0	IPF11181	9381355..9	unknown function	orf19.50	11181	IPF11177	PROTEIN S molecular_function unknown
CA2602	1.0	1.0	1.1	IPF5473	complemer	unknown function	orf19.5679	5473	IPF11181	PROTEIN I ligase activity
CA2603	1.1	1.0	0.9	IPF5471	complemer	unknown function	orf19.5680	5471	IPF5473	PROTEIN FATE [folding modification destination]
CA2604	1.0	1.1	1.0	IPF5469	complemer	unknown function	orf19.5681	5469	IPF5471	No significant S.c. match
CA2605	1.1	1.2	1.0	SRP1	9388143..9	alpha importin by homo	orf19.5682	5468	IPF5469	No significant S.c. match
CA2606	1.1	1.2	1.0	IPF5466	9389917..9	unknown function	orf19.5683	5466	CaSRP1	PROTEIN I protein binding
CA2607	1.1	1.2	1.1	MRPL38	9390697..9	ribosomal protein of th	orf19.5684	5464	IPF5466	UNCLASSI molecular_function unknown
CA2608	1.0	1.2	1.0	THS1	9391498..9	threonyl tRNA syntheta	orf19.5685	5462	CaMRPL38	PROTEIN S structural molecule activity
CA2609	1.0	0.9	0.9	IPF5457	9395086..9	similar to Saccharomy	orf19.5689	5457	CaTHS1	Nucleotide ligase activity
CA2610	1.3	1.1	1.0	CDC11	complemer	septin by homology	orf19.5691	5456	IPF5457	PROTEIN I molecular_function unknown

CA2611	0.9	0.9	0.9	IPF5453	9397365..9	unknown function	orf19.5692	5453	CaCDC11	C-compour	structural molecule activity
CA2612	1.1	1.0	1.0	GAA1	9399699..9	required for attachmen	orf19.5693	5450	IPF5453	No significant S.c. match	
CA2613	1.1	1.0	1.0	IPF5446	complemer	putative ribosomal prot	orf19.5698	5446	CaGAA1	Lipid fatty-a	hydrolase activity
CA2614	0.5	0.7	0.4	CTA241.E)	complemer	transcriptional activator, exon 2		5444	IPF5446	PROTEIN	structural molecule activity
CA2615	1.0	0.9	1.1	CTA241.E)	complemer	transcriptional activato	orf19.5700	5443	CaCTA241	No significant S.c. match	
CA2616	1.1	0.9	1.0	IPF3540	9407379..9	unknown function	orf19.2814	3540	CaCTA241	No significant S.c. match	
CA2617	0.9	0.9	0.8	IPF3539	complemer	unknown function	orf19.2813	3539	IPF3540	No significant S.c. match	
CA2618	1.1	1.0	1.1	SNG2	complemer	drug transporter (by hc	orf19.2812	19762	IPF3539	No significant S.c. match	
CA2619	0.9	1.0	0.9	IPF9490	complemer	amino acid permease (	orf19.2810	9490	CaSNG2	CELL RESCUE DEFENSE AND VIRULENCE ""TRANSPORT FACILITATION	
CA2620	0.7	0.7	0.5	IPF9496	complemer	carnitine O-acetyltrans	orf19.2809	9496	IPF9490	Amino acid metabolism CELLULAR TRANSPORT AND TRANSPORT MECHANISMS SUBCELLULAR LOCALISATION TRANSPORT FA	
CA2621	1.0	1.0	1.0	IPF9499	complemer	probable transcription	orf19.2808	9499	IPF9496	C-compour	transferase activity
CA2622	0.9	0.8	1.0	PER3.EXO	complemer	peroxisomal import prc	orf19.2806	11531	IPF9499	C-compound and carbohydrate metabolism ENERGY TRANSCRIPTION SUBCELLULAR LOCALISATION	
CA2623	0.9	0.9	0.9	PER3.EXO	complemer	peroxisomal import prc	orf19.1032	19631	CaPER3.e	No significant S.c. match	
CA2624	1.0	1.0	1.0	ARG81.3E	9436131..9	transcription factor possibly involve		17734	CaPER3.e	No significant S.c. match	
CA2625	1.1	1.0	1.2	IPF10919	9438459..9	Similar to Flo1p (by ho	orf19.1222	10919	CaARG81.	Amino acid metabolism Nitrogen and sulphur metabolism TRANSCRIPTION SUBCELLULAR LOCALISATION	
CA2626	0.9	0.9	1.1	PAN2	complemer	component of Pab1p-s	orf19.4764	10918	IPF10919	CONTROL	molecular_function unknown
CA2627	1.0	1.0	1.0	IPF10916	9444414..9	unknown function	orf19.4763	10916	CaPAN2	TRANSCR	RNA binding
CA2628	1.0	1.0	1.0	SIR22	complemer	canal regulatory protei	orf19.1222	10913	IPF10916	UNCLASSI	molecular_function unknown
CA2629	1.0	1.0	0.9	IPF10911	9448317..9	unknown function	orf19.1222	18440	CaSIR22	TRANSCR	hydrolase activity
CA2630	0.7	1.1	0.5	COX5A	9450350..9	cytochrome-c oxidase	orf19.1222	10910	IPF10911	UNCLASSI	transferase activity
CA2631	0.6	0.8	0.4	IPF14389	complemer	ubiquinone oxidoreduc	orf19.4758	14389	CaCOX5A	ENERGY	oxidoreductase activity
CA2632	1.0	0.9	0.9	NAR1	complemer	Yeast nuclear architect	orf19.4757	14388	IPF14389	No significant S.c. match	
CA2633	1.2	1.1	1.3	KEX2	9457875..9	Kexin precursor (KEX2	orf19.4755	13063	CaNAR1	UNCLASSI	oxidoreductase activity
CA2634	6.1	3.3	2.4	ZWF1	9462347..9	glucose-6-phosphate c	orf19.1221	13060	CaKEX2	PROTEIN	peptidase activity
CA2635	1.0	1.0	1.0	CKB22	9466876..9	Casein kinase II, beta	orf19.4297	2946	CaZWF1	C-compour	oxidoreductase activity
CA2636	1.0	1.0	1.0	MSW1	9468015..9	Mitochondrial tryptopha	orf19.4299	2944	CaCKB22	TRANSCR	protein kinase activity
CA2637	0.9	0.9	1.0	IPF19954	9470178..9	unknown function	orf19.4301	19954	CaMSW1	PROTEIN	ligase activity
CA2638	1.1	1.1	1.1	GAP3	complemer	General amino acid pe	orf19.4304	2936	IPF19954	No significant S.c. match	
CA2639	1.0	1.1	1.0	IPF2932	complemer	unknown function	orf19.4305	2932	CaGAP3	Amino acid metabolism CELLULAR TRANSPORT AND TRANSPORT MECHANISMS SUBCELLULAR LOCALISATION TRANSPORT FA	
CA2640	0.9	0.8	1.0	IPF2930	9476075..9	Suppressor of PAB1 (by homology		2930	IPF2932	CELLULAR TRANSPORT AND TRANSPORT MECHANISMS SUBCELLULAR LOCALISATION	
CA2641	1.0	1.0	0.9	IPF2929	complemer	unknown function	orf19.4306	2929	IPF2930	TRANSCRIPTION	
CA2642	1.0	0.9	1.0	FTI1	9477448..9	Rad52 inhibitor (by hor	orf19.4307	2927	IPF2929	UNCLASSI	molecular_function unknown
CA2643	0.8	0.8	0.9	HSL1	complemer	Ser/thr protein kinase t	orf19.4308	2926	CaFTI1	PROTEIN I	molecular_function unknown
CA2644	2.7	1.5	2.1	GRP2	complemer	Reductase (by homolo	orf19.4309	2918	CaHSL1	CELL CYC	protein kinase activity
CA2645	1.0	1.4	1.2	YNK1	9488422..9	Nucleoside diphosphat	orf19.4311	2914	CaGRP2	Metabolism of vitamins cofactors and prosthetic groups	
CA2646	1.0	1.0	0.9	IPF13089	9493426..9	unknown function	orf19.1185	13899	CaYNK1	Nucleotide	transferase activity
CA2647	1.1	1.0	1.2	IPF13088	9494709..9	unknown function	orf19.1186	13088	IPF13089	UNCLASSI	molecular_function unknown
CA2648	1.0	1.4	1.1	IPF9062	9497177..9	unknown function	orf19.1187	9062	IPF13088	UNCLASSIFIED PROTEINS	
CA2649	1.0	1.1	1.1	IPF9057	9504384..9	unknown function	orf19.1189	9057	IPF9062	CLASSIFIC	DNA binding,transcription regulator activity
CA2650	0.9	1.1	1.0	STV1	complemer	H+-ATPase V0 domair	orf19.1190	13604	IPF9057	CELL CYCLE AND DNA PROCESSING SUBCELLULAR LOCALISATION	
CA2651	1.0	1.0	1.1	HRD3	9511782..9	involved in HMG-CoA	orf19.1191	13605	CaSTV1	PROTEIN FATE [folding modification destination] ""CELLULAR TRANSPORT AND TRANSPORT MECHANISMS REGULATION OF/INTE	
CA2652	1.0	1.0	1.0	DNA2	complemer	DNA helicase (by hom	orf19.1192	15025	CaHRD3	PROTEIN I	ligase activity
CA2653	0.5	0.2	0.9	IPF8472.3	9519932..9	unknown function, 3-pr	orf19.5701	8472	CaDNA2	CELL CYC	DNA binding,helicase activity
CA2654	0.9	1.0	1.0	IPF8474	complemer	unknown function	orf19.5702	8474	IPF8472.3	UNCLASSI	protein binding
CA2655	0.9	0.8	0.9	IPF8477	9526218..9	unknown function	orf19.5704	8477	IPF8474	UNCLASSI	molecular_function unknown
CA2656	1.1	1.0	0.9	NAM2	complemer	mitochondrial leucine--	orf19.5705	19955	IPF8477	UNCLASSI	molecular_function unknown
CA2657	0.9	0.9	0.8	IPF8486	9531702..9	unknown function	orf19.5710	8486	CaNAM2	TRANSCR	RNA binding
CA2658	1.1	1.0	1.0	IPF8105	9538183..9	unknown function	orf19.5711	8105	IPF8486	TRANSCRIPTION CELLULAR TRANSPORT AND TRANSPORT MECHANISMS SUBCELLULAR LOCALISATION	
CA2659	1.1	1.0	1.1	NDH2	9540741..9	NADH dehydrogenase	orf19.5713	8102	IPF8105	Lipid fatty-a	transporter activity
CA2660	1.0	0.9	1.0	SAP1	complemer	secreted aspartyl prote	orf19.5714	8101	CaNDH2	ENERGY SUBCELLULAR LOCALISATION	
CA2661	1.1	1.0	1.2	AAT1	9551732..9	aspartate aminotransfe	orf19.3554	14258	CaSAP1	PROTEIN FATE [folding modification destination] ""Other virulence attributes	
CA2662	1.0	0.9	1.0	IPF17139	9553708..9	unknown function	orf19.3553	17139	CaAAT1	Amino acid metabolism Nitrogen and sulphur metabolism SUBCELLULAR LOCALISATION	
CA2663	0.9	0.9	0.9	NUP133	9555106..9	nuclear pore protein (b	orf19.3552	13373	IPF17139	UNCLASSI	RNA binding
CA2664	0.9	0.9	1.1	IPF13370	complemer	unknown function	orf19.3551	13370	CaNUP133	TRANSCR	structural molecule activity
CA2665	1.0	1.0	1.0	CDC21	complemer	thymidylate synthase (	orf19.3549	13364	IPF13370	UNCLASSI	structural molecule activity
CA2666	1.0	1.1	1.0	IPF16995	complemer	unknown function	orf19.3548	16995	CaCDC21	Nucleotide	transferase activity
CA2667	1.0	1.0	1.0	IPF16996	9563566..9	unknown function	orf19.3547	16996	IPF16995	UNCLASSI	molecular_function unknown
CA2668	1.2	1.0	1.2	IPF17553	complemer	similar to Saccharomy	orf19.3546	17553	IPF16996	UNCLASSI	molecular_function unknown
CA2669	1.0	1.0	1.0	IPF17494.3	complemer	unknown function, 3-prime end		17494	IPF17553	CELLULAF	protein binding
CA2670	1.1	1.2	1.2	MAK16	9569686..9	nuclear viral propagati	orf19.5500	5859	IPF17494.3	No significant S.c. match	
CA2671	0.9	0.6	1.0	IPF5856	9571354..9	4-hydroxyphenylpyruva	orf19.5499	5856	CaMAK16	CELL CYC	molecular_function unknown
CA2672	0.9	0.8	1.0	EFH1	complemer	Transcription regulator	orf19.5498	5855	IPF5856	No significant S.c. match	
CA2673	0.9	1.0	0.9	IPF5849	9580118..9	unknown function	orf19.5496	5849	CaEFH1	TRANSCRIPTION CELLULAR COMMUNICATION/SIGNAL TRANSDUCTION MECHANISM CELL FATE SUBCELLULAR LOCALISATIOI	
CA2674	1.0	0.9	0.9	IPF5846	complemer	unknown function	orf19.5495	5846	IPF5849	TRANSPORT FACILITATION	
CA2675	1.2	0.9	1.7	GSP1	9589303..9	GTP-binding protein (b	orf19.5493	5839	IPF5846	UNCLASSIFIED PROTEINS	
CA2676	1.1	1.1	1.1	YHC1	complemer	SMALL NUCLEAR RIE	orf19.5492	5838	CaGSP1	TRANSCR	hydrolase activity
CA2677	1.1	1.3	1.1	ATP14.EX	9591635..9	F1F0-ATPase complex, subunit h,		5835	CaYHC1	TRANSCR	RNA binding
CA2678	1.0	1.0	0.9	IPF5834	9592274..9	unknown function	orf19.1294	5834	CaATP14.e	ENERGY (	transporter activity
CA2679	1.1	1.3	1.1	IFU5	9594892..9	Unknown function	orf19.2568	19956	IPF5834	CELL CYCLE AND DNA PROCESSING CONTROL OF CELLULAR ORGANIZATION SUBCELLULAR LOCALISATION	
CA2680	1.0	1.0	0.9	MCI4	9595886..9	NADH dehydrogenase	orf19.2570	2728	CaIFU5	UNCLASSI	molecular_function unknown
CA2681	1.0	1.2	1.1	SEC4	complemer	GTP-binding protein	orf19.2571	2730	CaMCI4	No significant S.c. match	
CA2682	1.0	0.9	1.0	FRS1	complemer	Phenylalanyl-tRNA syr	orf19.2573	2731	CaSEC4	CELLULAF	hydrolase activity

CA2683	1.0	1.1	1.0	IFU4	complemer	Unknown function	orf19.2574	2734	CaFRS1	PROTEIN !	ligase activity
CA2684	1.0	1.0	1.0	IFU3	9603952..9	Unknown function	orf19.2575	2736	CaIFU4	UNCLASSI	molecular_function unknown
CA2685	0.9	0.9	0.9	MSH4	complemer	DNA mismatch repair	orf19.2579	2741	CaIFU3	CLASSIFIC	molecular_function unknown
CA2686	1.0	0.8	0.9	HST2	complemer	Transcription regulator	orf19.2580	2743	CaMSH4	CELL CYC	DNA binding
CA2687	1.0	1.0	1.1	IFU6.3F	complemer	Unknown function, 3-p	orf19.1011	18435	CaHST2	TRANSCR	hydrolase activity
CA2688	1.1	1.1	1.1	IFU6.5F	complemer	Putative ortholog of S.	orf19.2581	2744	CaIFU6.3f	No significant S.c. match	
CA2689	1.0	0.9	1.0	IFU2	9610078..9	Unknown function	orf19.2582	2746	CaIFU6.5f	Metabolism of vitamins cofactors and prosthetic groups	
CA2690	1.0	1.0	1.0	PTR2.EXO	complemer	Peptide transporter for di- and tripe		2747	CaIFU2	UNCLASSI	molecular_function unknown
CA2691	0.9	0.8	1.0	PTR2.EXO	complemer	Peptide transporter for	orf19.2583	2749	CaPTR2.e	No significant S.c. match	
CA2692	1.0	1.1	1.0	IPF9173.3	complemer	similar to Saccharomy	orf19.1024	10250	CaPTR2.e	CELLULAR TRANSPORT AND TRANSPORT MECHANISMS SUBCELLULAR LOCALISATION TRANSPORT FACILITATION	
CA2693	1.1	1.1	1.1	IPF9173.5	complemer	similar to Saccharomy	orf19.1024	9173	IPF9173.3f	PROTEIN FATE [folding modification destination] ""CELLULAR TRANSPORT AND TRANSPORT MECHANISMS	
CA2694	1.1	1.0	1.0	IPF9171	complemer	unknown function	orf19.1024	9171	IPF9173.5f	PROTEIN I	molecular_function unknown
CA2695	1.1	1.2	1.0	IPF9070	9624821..9	similar to Saccharomy	orf19.2735	9170	IPF9171	CLASSIFICATION NOT YET CLEAR-CUT	
CA2696	1.1	1.0	1.1	IPF9169	9626418..9	similar to Saccharomy	orf19.2736	9169	IPF9070	TRANSCR	RNA binding
CA2697	2.3	2.1	1.5	IPF9167	9627997..9	unknown function	orf19.2737	9167	IPF9169	TRANSCR	transcription regulator activity
CA2698	1.0	0.9	1.0	SUL1	9632399..9	High-affinity sulfate tra	orf19.1025	14417	IPF9167	UNCLASSIFIED PROTEINS	
CA2699	1.0	1.0	1.0	RLF2	9635300..9	chromatin assembly c	orf19.1025	19958	CaSUL1	Amino acid	transporter activity
CA2700	1.0	0.9	1.0	IPF14414.E	9637300..9	unknown function, exo	orf19.1025	17364	CaRLF2	CELL CYC	molecular_function unknown
CA2701	1.0	0.9	0.9	IPF14414.E	9638870..9	unknown function, exo	orf19.1025	15360	IPF14414.e	No significant S.c. match	
CA2702	1.0	1.0	1.1	EMP70	9644624..9	Endosomal protein (by	orf19.1026	14444	IPF14414.e	TRANSCRIPTION SUBCELLULAR LOCALISATION	
CA2703	1.0	1.0	1.0	IPF12193	9653983..9	unknown function	orf19.4655	12193	CaEMP70	CELLULAF	transporter activity
CA2704	0.9	0.8	1.0	IPF12195	complemer	unknown function	orf19.4656	12195	IPF12193	TRANSPORT FACILITATION	
CA2705	1.1	1.0	0.9	NEM1	complemer	required for nuclear m	orf19.4657	12196	IPF12195	No significant S.c. match	
CA2706	0.9	0.8	0.9	IPF11006	complemer	unknown function	orf19.4658	11006	CaNEM1	CELL CYC	molecular_function unknown
CA2707	1.0	1.0	1.0	IPF11003	9666525..9	by homology pre-mrna	orf19.4659	11003	IPF11006	UNCLASSIFIED PROTEINS	
CA2708	1.9	2.0	1.2	RPS6A	complemer	ribosomal protein S6 (t	orf19.4660	11001	IPF11003	TRANSCR	RNA binding
CA2709	1.0	1.0	1.0	IPF4828	complemer	by homology to S. cere	orf19.4662	4828	CaRPS6A	PROTEIN !	structural molecule activity
CA2710	0.9	1.0	1.1	IPF7403	complemer	unknown function	orf19.4664	7403	IPF4828	TRANSCR	DNA binding
CA2711	1.1	1.0	0.9	TFB3	complemer	Transcription/repair fa	orf19.8198	18427	IPF7403	UNCLASSI	molecular_function unknown
CA2712	0.8	0.7	1.1	SPE2	complemer	by homology to S. cere	orf19.8199	11572	CaTFB3	CELL CYC	transcription regulator activity
CA2713	0.9	0.9	1.0	IFF8	9680874..9	unknown function	orf19.570	11578	CaSPE2	Secondary	lyase activity
CA2714	1.1	1.1	1.0	IFF2	9687390..9	unknown function	orf19.575	7360	CaIFF8	No significant S.c. match	
CA2715	1.0	1.0	1.0	CTF8	9691912..9	putative) kinetochore p	orf19.576	7354	CaIFF2	UNCLASSIFIED PROTEINS	
CA2716	1.0	1.1	0.9	IPF7353	complemer	unknown function	orf19.577	7353	CaCTF8	UNCLASSI	molecular_function unknown
CA2717	1.0	1.0	1.1	MSB3	9696404..9	GTPase-activating pro	orf19.8209	7351	IPF7353	UNCLASSI	molecular_function unknown
CA2718	1.0	1.1	1.0	IPF7374	9704761..9	unknown function	orf19.1084	7374	CaMSB3	UNCLASSI	enzyme regulator activity
CA2719	1.0	1.1	1.0	SOD2	complemer	Manganese-superoxid	orf19.3340	7368	IPF7374	No significant S.c. match	
CA2720	0.9	1.0	1.0	IPF7366	complemer	Arginyl-tRNA syntheta	orf19.3341	7366	CaSOD2	CELL RES	oxidoreductase activity
CA2721	1.0	1.1	0.9	IPF4799	9714328..9	unknown Function	orf19.3342	4799	IPF7366	PROTEIN !	ligase activity
CA2722	1.0	1.0	1.0	IPF4801	9716406..9	similar to Saccharomy	orf19.3344	4801	IPF4799	UNCLASSI	molecular_function unknown
CA2723	1.0	1.0	1.0	IPF4805	9718368..9	unknown Function	orf19.3345	4805	IPF4801	PROTEIN I	molecular_function unknown
CA2724	1.0	1.0	1.0	RPB7	complemer	DNA-directed RNA pol	orf19.1085	4809	IPF4805	PROTEIN I	ligase activity
CA2725	1.1	1.2	1.1	MRPL23A	9724136..9	mitochondrial ribosom	orf19.1085	4810	CaRPB7	TRANSCR	nucleotidyltransferase activity
CA2727	1.2	1.2	1.1	IPF10318	complemer	similar to Saccharomy	orf19.2119	10318	CaMRPL2	PROTEIN !	structural molecule activity
CA2728	0.9	1.0	1.0	IPF10322	9728491..9	putative mitochondrial	orf19.9665	10322	IPF10318	CELL CYC	DNA binding,transcription regulator activity
CA2729	0.9	1.0	1.0	NAT2	complemer	N-acetyltransferase fo	orf19.9664	10323	IPF10322	Nucleotide	transporter activity
CA2730	1.0	1.0	1.0	IPF10325	complemer	molybdopterin-converti	orf19.9663	10325	CaNAT2	PROTEIN !	transferase activity
CA2731	0.9	0.9	0.9	URIC	complemer	uricase (urate oxidase	orf19.2114	10326	IPF10325	Metabolism	molecular_function unknown
CA2732	1.0	1.1	1.0	IPF10327	complemer	unknown function	orf19.2113	10327	CaURIC	No significant S.c. match	
CA2733	1.0	1.0	1.0	PRP18	9734529..9	U5 snRNA-associated	orf19.2112	10329	IPF10327	UNCLASSI	molecular_function unknown
CA2734	1.0	1.2	1.1	RPL38	9735515..9	ribosomal protein L38 (by homolog		10330	CaPRP18	TRANSCR	protein binding
CA2735	1.1	1.1	1.0	TFG2	complemer	transcription initiation f	orf19.2111	19765	CaRPL38	PROTEIN !	structural molecule activity
CA2736	1.1	1.2	1.1	ATE1	9737124..9	arginyl tRNA transfera	orf19.2110	15421	CaTFG2	TRANSCR	transcription regulator activity
CA2737	1.0	0.8	1.0	IPF15423	complemer	putative superoxide dis	orf19.2108	15423	CaATE1	Amino acid	transferase activity
CA2738	0.8	0.5	0.5	STF2	9740300..9	ATP synthase regulatory factor (by		15425	IPF15423	No significant S.c. match	
CA2739	0.9	0.8	1.2	MUQ1	complemer	choline phosphate cyti	orf19.2107	10634	CaSTF2	PROTEIN I	molecular_function unknown
CA2740	0.9	0.9	0.8	IPF10633	complemer	unknown function	orf19.2106	10633	CaMUQ1	Lipid fatty-a	nucleotidyltransferase activity
CA2741	1.0	1.0	0.8	IPF10632	complemer	unknown function	orf19.2105	10632	IPF10633	UNCLASSI	molecular_function unknown
CA2742	0.9	0.9	0.8	JAC1	9743500..9	molecular chaperone (	orf19.2104	10631	IPF10632	UNCLASSI	molecular_function unknown
CA2743	1.0	1.1	1.0	CKB21	complemer	Casein kinase II, beta	orf19.2102	10630	CaJAC1	ENERGY "	chaperone activity
CA2744	1.1	1.0	1.0	IPF10626	9745477..9	unknown function	orf19.2101	10626	CaCKB21	TRANSCR	protein kinase activity
CA2745	0.8	0.8	0.7	HIR1	complemer	Histone transcription r	orf19.9647	10625	IPF10626	UNCLASSI	molecular_function unknown
CA2746	0.9	1.0	0.9	IPF19766	9749207..9	unknown function	orf19.9646	19766	CaHIR1	TRANSCR	transcription regulator activity
CA2747	0.9	0.8	0.9	IPF11467	complemer	unknown function	orf19.2367	11467	IPF19766	Lipid fatty-acid and isoprenoid metabolism ""CONTROL OF CELLULAR ORGANIZATION	
CA2748	1.0	1.0	0.9	IPF11466	9756151..9	unknown function	orf19.2368	11466	IPF11467	UNCLASSI	molecular_function unknown
CA2749	0.9	0.9	1.0	IPF11465	complemer	unknown function	orf19.2369	11465	IPF11466	CELLULAF	protein binding
CA2750	1.0	1.0	1.0	ATX1	9759522..9	antioxidant protein and metal hom		11464	IPF11465	CELL CYC	DNA binding
CA2751	1.0	0.9	0.9	IPF19558	complemer	unknown function	orf19.2370	19558	CaATX1	CELL RES	chaperone activity
CA2752	1.0	1.0	0.9	IPF6238	9768682..9	GAG protein of retrotra	orf19.2374	6238	IPF19558	UNCLASSI	molecular_function unknown
CA2753	1.0	1.0	1.0	IPF16806	9772787..9	unknown function	orf19.1306	16806	IPF6238	No significant S.c. match	
CA2754	0.9	0.7	0.9	VPS45	complemer	vacuolar protein sortin	orf19.1306	12432	IPF16806	UNCLASSI	protein binding
CA2755	1.0	0.9	1.1	IFA12	9777121..9	unknown function	orf19.1306	12435	CaVPS45	PROTEIN I	chaperone activity

CA2756	1.0	1.2	1.0	IPF18418	9779713..9	unknown function	orf19.1306	18418	CaIFA12	No significant S.c. match	
CA2757	1.1	1.0	1.1	IPF19767	complemer	unknown function	orf19.1306	19767	IPF18418	UNCLASSI	molecular_function unknown
CA2758	1.4	1.3	1.2	GLC3	complemer	1,4-glucan branching e	orf19.1306	16449	IPF19767	UNCLASSI	molecular_function unknown
CA2759	0.9	0.9	0.9	ARP4	complemer	actin-related protein (b	orf19.1306	13789	CaGLC3	C-compour	transferase activity
CA2760	1.0	1.9	1.1	IPF13790	complemer	Unknown function	orf19.1307	13790	CaARP4	CONTROL	DNA binding
CA2761	0.9	1.0	0.7	IPF15013	9793468..9	pyruvate decarboxylas	orf19.1307	15013	IPF13790	No significant S.c. match	
CA2762	1.1	1.0	1.0	IPF15012	9795542..9	pre mRNA splicing fac	orf19.1307	15012	IPF15013	No significant S.c. match	
CA2763	1.0	1.1	1.1	DIC1.3	9797980..9	dicarboxylate carrier p	orf19.5628	16773	IPF15012	UNCLASSI	RNA binding
CA2764	0.9	1.0	0.6	QCR7	complemer	ubiquinol--cytochrome-	orf19.5629	16775	CaDIC1.3	Phosphate	transporter activity
CA2765	1.0	1.1	1.0	APA2	complemer	ATP adenylyltransfera	orf19.1307	18417	CaQCR7	ENERGY "	transporter activity,oxidoreductase activity
CA2766	0.9	1.0	1.0	IPF18416	9801246..9	unknown function	orf19.1307	18416	CaAPA2	Nucleotide	hydrolase activity
CA2767	0.8	1.0	1.0	TOA1	complemer	transcription initiation f	orf19.2682	14884	IPF18416	No significant S.c. match	
CA2768	1.0	0.9	0.9	IPF17011	complemer	similar to Saccharomy	orf19.2684	17011	CaTOA1	TRANSCR	transcription regulator activity
CA2769	1.9	3.0	2.9	IPF15870	complemer	unknown function	orf19.2685	15870	IPF17011	CELL CYC	molecular_function unknown
CA2770	1.0	1.1	1.0	CPS1	9815697..9	Carboxypeptidase YS	orf19.2686	7980	IPF15870	No significant S.c. match	
CA2771	1.0	1.0	1.0	RPB10	complemer	DNA-directed RNA polymerase II (		7977	CaCPS1	Nitrogen ar	peptidase activity
CA2772	1.0	1.1	0.9	IPF20112	9818217..9	unknown function	orf19.2688	20112	CaRPB10	TRANSCR	nucleotidyltransferase activity
CA2773	0.9	1.0	0.9	MGM1	complemer	GTPase	orf19.2690	7974	IPF20112	CELL CYC	RNA binding
CA2774	1.1	1.0	1.1	TFC4	9827896..9	transcription factor IIIC	orf19.274	10122	CaMGM1	SUBCELLU	hydrolase activity
CA2775	0.9	0.9	1.0	POP5	9831324..9	subunit of RNase P- lik	orf19.275	10123	CaTFC4	TRANSCR	transcription regulator activity
CA2776	0.8	0.9	0.8	IPF10124	complemer	Alcohol acetyltransfera	orf19.276	10124	CaPOP5	CELL CYC	RNA binding
CA2777	1.0	1.1	1.1	THI6	9833961..9	thiamin-phosphate pyr	orf19.277	10126	IPF10124	UNCLASSI	molecular_function unknown
CA2778	0.9	0.9	0.9	MTR	complemer	neutral amino acid per	orf19.278	10127	CaTHI6	Metabolism	transferase activity
CA2779	1.1	1.0	1.0	IPF3836	9837627..9	unknown function	orf19.279	19768	CaMTR	UNCLASSIFIED PROTEINS	
CA2780	1.0	1.0	1.1	IPF3831	9838807..9	unknown function	orf19.281	3798	IPF3836	UNCLASSI	hydrolase activity
CA2781	1.0	0.9	1.2	IPF3821	9840046..9	unknown function	orf19.284	3805	IPF3831	No significant S.c. match	
CA2782	0.8	1.8	0.6	IPF3806	9840952..9	unknown function	orf19.285	3806	IPF3821	No significant S.c. match	
CA2783	1.0	0.9	0.9	IPF3808	9841819..9	unknown function	orf19.286	3808	IPF3806	UNCLASSI	molecular_function unknown
CA2784	1.0	1.0	1.0	IPF3810	complemer	unknown function	orf19.287	3810	IPF3808	PROTEIN FATE [folding modification destination]	
CA2785	1.0	1.2	1.1	MET13	complemer	Methylene tetrahydrofo	orf19.288	3814	IPF3810	No significant S.c. match	
CA2786	1.0	0.9	1.0	KRE5.3EO	complemer	UDP-glucose:glycopro	orf19.290	3818	CaMET13	Amino acid	structural molecule activity
CA2787	1.3	1.3	1.0	EFG1	9858262..9	Enhanced filamentous	orf19.8243	3577	CaKRE5.3	C-compour	transferase activity
CA2788	0.7	0.4	0.9	RAD26	complemer	DNA repair and recom	orf19.8240	3569	CaEFG1	TRANSCR	DNA binding,transcription regulator activity
CA2789	0.6	0.7	0.5	IPF3567	9865948..9	unknown function	orf19.606	3567	CaRAD26	CELL CYC	hydrolase activity
CA2790	1.0	0.9	1.0	IPF3562	9869139..9	Unknown function	orf19.604	3562	IPF3567	UNCLASSI	molecular_function unknown
CA2791	1.1	0.9	0.9	IMP4	9871684..9	Ribonucleoprotein (by	orf19.603	3560	IPF3562	Metabolism of vitamins cofactors and prosthetic groups	
CA2792	1.0	1.0	1.0	TRK1.3F	complemer	Potassium transporter,	orf19.602	3559	CaIMP4	TRANSCR	RNA binding
CA2793	0.9	0.9	1.0	TRK1.5F	complemer	Potassium transporter,	orf19.8233	3557	CaTRK1.3	CELLULAR TRANSPORT AND TRANSPORT MECHANISMS SUBCELLULAR LOCALISATION TRANSPORT FACILITATION	
CA2794	1.0	0.9	0.9	IPF8408	9887619..9	unknown function	orf19.1724	8408	CaTRK1.5	CELLULAF	transporter activity
CA2795	1.0	0.9	1.0	IPF8407	9888306..9	unknown function	orf19.1723	8407	IPF8408	No significant S.c. match	
CA2796	0.8	0.8	0.9	IPF8405	complemer	similar to Saccharomy	orf19.1721	8405	IPF8407	UNCLASSI	molecular_function unknown
CA2797	1.0	1.0	1.1	IPF8404	9891254..9	putative helicase (by h	orf19.1720	8404	IPF8405	CELLULAF	molecular_function unknown
CA2798	1.0	0.9	1.0	IPF8402	complemer	similar to Saccharomy	orf19.1719	8402	IPF8404	CELL CYC	helicase activity
CA2799	0.9	0.7	1.0	IPF19769	9899194..9	unknown function	orf19.1718	19769	IPF8402	C-compour	hydrolase activity
CA2800	1.1	1.0	1.1	IPF11366	9902499..9	unknown function	orf19.1717	11366	IPF19769	No significant S.c. match	
CA2801	1.0	1.0	1.0	URA3	complemer	orotidine-5 -monophos	orf19.1716	11364	IPF11366	No significant S.c. match	
CA2802	1.3	1.2	1.5	IPF11363	complemer	unknown function	orf19.9283	11363	CaURA3	Nucleotide	lyase activity
CA2803	1.1	1.0	0.9	IPF11711	9909449..9	ubiquitin-protein ligase	orf19.5776	11711	IPF11363	No significant S.c. match	
CA2804	0.9	0.9	1.0	IPF19961	complemer	unknown function	orf19.5777	19961	IPF11711	CELL CYC	ligase activity
CA2805	1.3	1.0	1.5	RNR1	9914611..9	ribonucleoside-diphos	orf19.5779	11704	IPF19961	UNCLASSI	molecular_function unknown
CA2806	1.0	0.9	0.9	IPF11702	9918043..9	unknown function	orf19.5780	11702	CaRNR1	Nucleotide	oxidoreductase activity
CA2807	0.8	0.8	0.8	IPF11965	9920339..9	unknown function	orf19.5782	11965	IPF11702	UNCLASSI	molecular_function unknown
CA2808	0.9	1.0	0.9	IPF11966	complemer	unknown function	orf19.5783	11966	IPF11965	UNCLASSI	hydrolase activity
CA2809	0.9	1.0	0.9	AMO1	complemer	amine oxidase (by hon	orf19.5784	11969	IPF11966	UNCLASSI	molecular_function unknown
CA2810	1.1	1.3	1.3	EFT2	complemer	translation elongation f	orf19.5788	6840	CaAMO1	No significant S.c. match	
CA2811	0.9	1.0	1.2	RPS10	complemer	Ribosomal protein 10	orf19.1052	5347	CaEFT2	PROTEIN S	translation regulator activity
CA2812	0.9	1.0	1.0	TEM1	9937411..9	GTP-binding protein of	orf19.1051	5346	CaRPS10	PROTEIN S	structural molecule activity
CA2813	1.0	1.1	1.0	ORC1	9938442..9	Origin recognition com	orf19.1051	5612	CaTEM1	CELL CYC	protein binding
CA2814	1.1	1.2	1.0	IPF5607	9941036..9	unknown function	orf19.2998	5607	CaORC1	CELL CYC	DNA binding
CA2815	1.0	1.0	1.0	IPF5604	complemer	unknown function	orf19.2996	5604	IPF5607	UNCLASSI	molecular_function unknown
CA2816	1.0	1.1	1.0	IPF5601	complemer	unknown function	orf19.2995	5601	IPF5604	CONTROL	molecular_function unknown
CA2817	0.9	2.2	1.2	RPS16.3	complemer	ribosomal protein, 3-prime end		5598	IPF5601	UNCLASSI	molecular_function unknown
CA2818	1.2	1.8	1.1	RPL13	9948383..9	Ribosomal protein	orf19.2994	5596	CaRPS16.	PROTEIN S	structural molecule activity
CA2819	2.1	1.2	1.8	RPA1	complemer	60S ribosomal protein	orf19.2992	5594	CaRPL13	PROTEIN S	structural molecule activity
CA2820	0.9	0.9	0.9	HOL1	complemer	member of major facilit	orf19.2991	5592	CaRPA1	PROTEIN S	structural molecule activity
CA2821	0.9	0.9	1.0	CDR3.3EO	9955198..9	Opaque-specific ABC transporter,		17152	CaHOL1	CELL RESCUE DEFENSE AND VIRULENCE ""TRANSPORT FACILITATION	
CA2822	0.9	1.0	0.9	IPF14550	9957534..9	unknown function	orf19.1314	17153	CaCDR3.3	SUBCELLULAR LOCALISATION TRANSPORT FACILITATION	
CA2823	1.0	1.0	1.0	IPF14554	9960135..9	similar to Saccharomy	orf19.1317	14554	IPF14550	UNCLASSIFIED PROTEINS	
CA2824	1.0	0.9	0.9	IPF17888	complemer	unknown function	orf19.1318	17888	IPF14554	Lipid fatty-acid and isoprenoid metabolism	
CA2825	1.0	0.9	1.1	HWP1	complemer	Hyphal wall protein	orf19.1321	12916	IPF17888	UNCLASSI	molecular_function unknown
CA2826	1.0	1.0	1.0	APL6	complemer	AP-3 complex subunit,	orf19.1323	12906	CaHWP1	C-compound and carbohydrate metabolism SUBCELLULAR LOCALISATION Hypha-specific	
CA2827	0.9	0.9	1.0	RAD2	complemer	structure-specific nucle	orf19.1324	19770	CaAPL6	CELLULAF	molecular_function unknown

CA2828	1.0	1.0	0.9	IPF17026	9974890..9	unknown function	orf19.1325	17026	CaRAD2	CELL CYC DNA binding
CA2829	1.0	1.1	1.0	IFA7	complemen	unknown function	orf19.1326	14593	IPF17026	Amino acid metabolism CELL CYCLE AND DNA PROCESSING CONTROL OF CELLULAR ORGANIZATION
CA2830	0.9	0.7	0.9	RBT1	9982188..9	repressed by TUP1 pro	orf19.1327	15385	CaIFA7	Nucleotide metabolism CELL CYCLE AND DNA PROCESSING CELLULAR COMMUNICATION/SIGNAL TRANSDUCTION MECHANISM
CA2831	1.0	0.9	0.9	IPF15706	complemen	unknown function	orf19.4783	15706	CaRBT1	Hypha-specific No significant S.c. match
CA2832	0.9	1.0	1.0	CRD1	9988871..9	Cu-transporting P1-typ	orf19.4784	19962	IPF15706	No significant S.c. match
CA2833	0.8	0.7	0.9	PTC1	complemen	protein serine/threonin	orf19.4785	19963	CaCRD1	REGULATION OF/INTERACTION WITH CELLULAR ENVIRONMENT SUBCELLULAR LOCALISATION TRANSPORT FACILITATION
CA2834	0.9	1.0	1.0	IPF12093	9994618..9	unknown function	orf19.4786	12093	CaPTC1	C-compour protein phosphatase activity
CA2835	1.0	1.0	1.0	IPF12091	9996734..9	Unknown function	orf19.4787	12091	IPF12093	UNCLASSIFIED PROTEINS
CA2836	1.1	1.1	1.1	ARG5,6	complemen	acetylglutamate kinase	orf19.4788	12088	IPF12091	CELL CYCLE AND DNA PROCESSING ""PROTEIN FATE [folding modification destination] ""CELLULAR TRANSPORT AND TRANSPOR
CA2837	1.0	1.0	1.0	IPF8340	complemen	unknown function	orf19.4789	8340	CaARG5,6	Amino acid oxidoreductase activity,transferase activity
CA2838	1.2	1.1	1.1	IPF8339	complemen	unknown function	orf19.4791	8339	IPF8340	No significant S.c. match
CA2839	1.2	1.2	1.1	IPF8336	complemen	unknown function	orf19.4792	8336	IPF8339	No significant S.c. match
CA2841	0.9	1.0	1.0	IPF12981	10012740..	unknown function	orf19.5727	12981	IPF8336	No significant S.c. match
CA2842	1.0	0.9	1.0	ALK5.3F	complemen	n-alkane-inducible cytochrome P-4		18405	IPF12981	No significant S.c. match
CA2843	1.0	1.1	0.9	ALK5.5F	complemen	n-alkane-inducible cyto	orf19.5728	13156	CaALK5.3f	CELL RESCUE DEFENSE AND VIRULENCE ""CELL FATE CONTROL OF CELLULAR ORGANIZATION
CA2844	0.9	1.0	0.9	IPF13158	10021410..	unknown function	orf19.5729	13158	CaALK5.5f	CELL RESCUE DEFENSE AND VIRULENCE ""CELL FATE CONTROL OF CELLULAR ORGANIZATION
CA2845	1.0	0.9	1.0	IPF10782	complemen	unknown function	orf19.5730	10782	IPF13158	Amino acid metabolism TRANSCRIPTION SUBCELLULAR LOCALISATION
CA2846	1.0	1.0	0.9	PAD1	10025559..	phenylacrylic acid deca	orf19.5731	10783	IPF10782	UNCLASSI molecular_function unknown
CA2847	0.9	1.0	1.0	IPF10785.E	10026553..	unknown function, exo	orf19.5732	10785	CaPAD1	CELL RES lyase activity
CA2848	1.0	1.1	1.0	IPF10785.E	10028105..	unknown function, exo	orf19.5733	10786	IPF10785.e	UNCLASSI hydrolase activity
CA2849	1.0	1.0	1.0	POP2	complemen	required for glucose de	orf19.5734	10787	IPF10785.e	UNCLASSIFIED PROTEINS
CA2850	1.0	1.1	1.0	CDC50	complemen	cell division cycle muta	orf19.5735	10789	CaPOP2	C-compour RNA binding
CA2852	1.0	0.9	0.9	ALS5	complemen	agglutinin-like protein	orf19.5736	19771	CaCDC50	CELL CYC transcription regulator activity
CA2853	0.7	0.5	0.9	IPF17057	10043344..	unknown function	orf19.1070	17057	CaALS5	CELL FATE SUBCELLULAR LOCALISATION Other virulence attributes
CA2854	1.3	1.8	1.0	RPN4	complemen	26S proteasome subur	orf19.1069	7070	IPF17057	UNCLASSI molecular_function unknown
CA2855	1.0	1.0	1.0	GPM2	complemen	phosphoglycerate muta	orf19.1067	7065	CaRPN4	PROTEIN I peptidase activity
CA2856	1.1	1.0	1.1	IPF7062	10050750..	unknown function	orf19.1066	7062	CaGPM2	C-compound and carbohydrate metabolism ENERGY SUBCELLULAR LOCALISATION
CA2857	1.0	1.0	1.0	SSA1	10052114..	Heat shock protein of I	orf19.1065	7060	IPF7062	UNCLASSI molecular_function unknown
CA2858	0.4	0.6	0.7	ACS2	10056401..	acetyl-coenzyme-A syr	orf19.1064	7057	CaSSA1	CELL RESCUE DEFENSE AND VIRULENCE ""SUBCELLULAR LOCALISATION
CA2859	1.0	1.0	1.0	IPF7056	10058692..	unknown function	orf19.1063	7056	CaACS2	C-compour ligase activity
CA2860	1.0	1.0	1.1	IPF7054	10060272..	unknown function	orf19.1062	7054	IPF7056	UNCLASSI molecular_function unknown
CA2861	1.7	2.4	2.3	HHT21	complemen	Histone H3	orf19.1061	7053	IPF7054	No significant S.c. match
CA2862	2.7	1.5	3.1	HHF21	10063170..	histone H4	orf19.1059	7050	CaHHT21	TRANSCR DNA binding
CA2863	1.3	1.2	1.1	IPF20117	complemen	unknown function	orf19.1058	20117	CaHHF21	TRANSCR DNA binding
CA2864	1.1	1.0	1.1	IPF17234.3	10065092..	unknown function, 3-pr	orf19.1057	17234	IPF20117	UNCLASSI peptidase activity
CA2866	1.2	1.2	1.1	RHO1	complemen	GTP-binding protein of	orf19.2843	11690	IPF17234.3	No significant S.c. match
CA2867	1.0	0.9	1.0	IPF11688	10079064..	similar to Saccharomyc	orf19.2844	11688	CaRHO1	C-compour signal transducer activity
CA2868	1.0	1.0	1.2	IPF20118	10082128..	unknown function	orf19.2846	20118	IPF11688	PROTEIN I protein binding
CA2869	1.1	1.0	1.0	RPC82	complemen	DNA-directed RNA pol	orf19.2847	15457	IPF20118	No significant S.c. match
CA2870	0.9	1.0	1.0	OPT2.53F	10084983..	Oligopeptide transporter, internal f		17923	CaRPC82	TRANSCR nucleotidyltransferase activity
CA2871	0.9	0.9	1.1	OPT2.3F	10085454..	Oligopeptide transporte	orf19.2847	17924	CaOPT2.53	TRANSPORT FACILITATION
CA2872	0.9	1.1	1.0	APG13	10088575..	probable component o	orf19.2848	10707	CaOPT2.3f	TRANSPORT FACILITATION
CA2873	1.1	1.2	1.0	AQY1	complemen	similarity to plasma me	orf19.2849	10705	CaAPG13	PROTEIN I protein binding
CA2874	1.1	1.1	1.1	IPF4999	10101550..	unknown function	orf19.5843	4999	CaAQY1	TRANSPO transporter activity
CA2875	0.9	0.9	1.0	MEI5	10105914..	meiotic protein (by hon	orf19.5844	17969	IPF4999	No significant S.c. match
CA2876	1.1	1.1	1.0	RNR2	10106787..	Ribonucleotide reducta	orf19.5845	3774	CaMEI5	CELL CYC molecular_function unknown
CA2877	0.9	0.8	0.9	TFB2	complemen	Transcription/repair fac	orf19.5846	3776	CaRNR2	Nucleotide metabolism CELL CYCLE AND DNA PROCESSING
CA2878	0.9	0.9	0.8	RET1	10111192..	DNA-directed RNA pol	orf19.5847	3777	CaTFB2	CELL CYC transcription regulator activity
CA2879	1.5	1.5	1.1	IPF3779	10115248..	unknown function	orf19.5848	3779	CaRET1	TRANSCR nucleotidyltransferase activity
CA2880	0.9	0.9	1.0	IPF3781	10117617..	unknown function	orf19.5849	3781	IPF3779	UNCLASSIFIED PROTEINS
CA2881	1.0	1.0	0.9	RAD4	complemen	Excision repair protein	orf19.5850	3784	IPF3781	TRANSCR DNA binding,transcription regulator activity
CA2882	1.1	1.0	1.0	STE13	complemen	type IV dipeptidyl amin	orf19.5851	3786	CaRAD4	CELLULAF molecular_function unknown
CA2883	0.9	0.8	0.9	IPF3790	10125186..	unknown function	orf19.5852	3790	CaSTE13	PROTEIN I peptidase activity
CA2884	1.0	1.0	1.0	IPF7201	complemen	similar to Saccharomyc	orf19.2476	7201	IPF3790	CELLULAR TRANSPORT AND TRANSPORT MECHANISMS SUBCELLULAR LOCALISATION
CA2885	0.9	0.9	0.9	IPF7204	complemen	unknown function	orf19.2475	7204	IPF7201	CONTROL molecular_function unknown
CA2886	1.1	1.0	1.0	IPF7207	10141237..	unknown function	orf19.2473	7207	IPF7204	No significant S.c. match
CA2887	1.0	0.9	1.0	IPF13631	complemen	unknown function	orf19.2472	13631	IPF7207	UNCLASSI DNA binding
CA2888	0.9	0.8	1.0	GIM5	10144589..	Gim complex compone	orf19.2471	13630	IPF13631	No significant S.c. match
CA2889	1.0	1.0	0.9	IPF13628	complemen	putative DNA repair pro	orf19.2469	13628	CaGIM5	PROTEIN I protein binding
CA2890	1.0	1.0	1.0	IPF13626	10146672..	Putative methyltransfe	orf19.2468	13626	IPF13628	CELL CYC DNA binding
CA2891	1.1	1.0	1.2	IFS4	10148151..	Pirin protein (by homol	orf19.2467	6686	IPF13626	UNCLASSI transferase activity
CA2892	1.0	0.9	0.9	IPF6688	10149496..	unknown function	orf19.2465	6688	CaIFS4	No significant S.c. match
CA2893	1.0	0.9	1.0	IFS3	10151006..	Unknown function	orf19.2463	6690	IPF6688	No significant S.c. match
CA2894	0.9	0.7	1.0	IFS2	10152524..	Unknown function	orf19.2462	6691	CaIFS3	UNCLASSIFIED PROTEINS
CA2895	1.0	1.1	1.1	IFS1	10154280..	Unknown function	orf19.2461	6694	CaIFS2	No significant S.c. match
CA2896	0.9	0.8	1.0	IPF14981	complemen	unknown function	orf19.3483	14981	CaIFS1	UNCLASSIFIED PROTEINS
CA2897	1.0	1.1	1.1	IPF14979	complemen	similar to Saccharomyc	orf19.3482	14979	IPF14981	UNCLASSI molecular_function unknown
CA2898	0.8	0.8	0.9	IPF15646	complemen	putative ATP-depende	orf19.3481	15646	IPF14979	Nucleotide hydrolase activity
CA2899	1.0	1.0	1.0	IPF17681	10164782..	similar to Saccharomyc	orf19.3480	17681	IPF15646	UNCLASSI RNA binding,helicase activity
CA2900	1.0	1.0	0.9	NIP7	complemen	required for efficient 60	orf19.3478	11733	IPF17681	CLASSIFIC structural molecule activity
CA2901	0.9	0.9	0.9	PUS1	10167784..	pseudouridine synthas	orf19.3477	11730	CaNIP7	TRANSCR molecular_function unknown
CA2902	0.9	1.2	0.8	HRR25	complemen	casein kinase I (by hor	orf19.3476	11729	CaPUS1	Nucleotide lyase activity

CA2903	1.2	0.9	1.2	IPF11725	complemer	unknown function	orf19.1097	11725	CaHRR25	CELL CYC	protein kinase activity
CA2904	0.9	1.0	1.0	IPL1	10177676..	Ser/thr protein kinase (	orf19.3474	12051	IPF11725	No significant S.c. match	
CA2905	1.0	1.0	1.0	IPF12049	complemer	unknown function	orf19.1097	12049	CaIPL1	CELL CYC	protein kinase activity
CA2906	1.0	1.1	1.1	IPF15523	complemer	unknown function	orf19.4521	15523	IPF12049	UNCLASSI	molecular_function unknown
CA2907	0.9	0.9	1.4	IPF15525	complemer	putative gluconokinase	orf19.4520	15525	IPF15523	CLASSIFIC	oxidoreductase activity
CA2908	1.0	1.0	1.1	SUV3	complemer	ATP-dependent RNA h	orf19.4519	15527	IPF15525	C-compour	molecular_function unknown
CA2909	1.1	1.0	0.9	IPF11424	10190600..	unknown function	orf19.4518	11424	CaSUV3	Nucleotide	RNA binding,helicase activity
CA2910	1.1	1.0	1.0	IPF11421	10193868..	unknown function	orf19.4517	11421	IPF11424	UNCLASSI	protein kinase activity
CA2911	0.9	0.7	0.9	MET7	complemer	folylpolyglutamate synt	orf19.4516	11419	IPF11421	No significant S.c. match	
CA2912	1.1	1.0	1.1	IPF11206	10197825..	unknown function	orf19.4515	11206	CaMET7	Metabolism	ligase activity
CA2913	1.0	1.0	1.0	IPF11205	10199936..	unknown function	orf19.4513	11205	IPF11206	CELL RESCUE DEFENSE AND VIRULENCE ""CELL FATE	
CA2914	1.0	1.0	1.0	IFA17.5F	10201694..	unknown function, 5-pr	orf19.4512	11203	IPF11205	Lipid fatty-acid and isoprenoid metabolism ""CELL CYCLE AND DNA PROCESSING CELLULAR COMMUNICATION/SIGNAL TRANSDUC	
CA2915	0.9	0.9	1.0	IFA17.3F	10202423..	unknown function, 3-pr	orf19.4511	11201	CaIFA17.5	No significant S.c. match	
CA2916	1.0	1.0	1.1	IFA4	10204695..	unknown function	orf19.4510	11199	CaIFA17.3	Nucleotide metabolism CELL CYCLE AND DNA PROCESSING CELLULAR COMMUNICATION/SIGNAL TRANSDUCTION MECHANISM	
CA2917	1.1	0.8	1.0	IPF16173.5	10207753..	Unknown function, 5-p	orf19.4509	16177	CaIFA4	Nucleotide metabolism CELL CYCLE AND DNA PROCESSING CELLULAR COMMUNICATION/SIGNAL TRANSDUCTION MECHANISM	
CA2918	1.0	1.0	1.0	IPF16173.3	10208060..	unknown function, 3-pr	orf19.4508	16174	IPF16173.5	No significant S.c. match	
CA2919	1.0	1.0	0.9	IFA18.3	10208768..	unknown function, 3-pr	orf19.4507	16173	IPF16173.3	No significant S.c. match	
CA2920	2.1	3.1	1.4	FET5	complemer	multicopy oxidase (by l	orf19.4215	17039	CaIFA18.3	Nucleotide metabolism CELL CYCLE AND DNA PROCESSING CELLULAR COMMUNICATION/SIGNAL TRANSDUCTION MECHANISM	
CA2921	1.0	0.9	1.0	IPF17037	complemer	unknown function	orf19.4214	17037	CaFET5	CELLULAR TRANSPORT AND TRANSPORT MECHANISMS REGULATION OF/INTERACTION WITH CELLULAR ENVIRONMENT SUB	
CA2922	1.0	1.0	1.1	FET31	complemer	cell surface ferroxidase	orf19.1168	17035	IPF17037	ENERGY	
CA2923	1.2	1.2	1.3	FET32	complemer	cell surface ferroxidase	orf19.1168	11905	CaFET31	CELLULAR TRANSPORT AND TRANSPORT MECHANISMS REGULATION OF/INTERACTION WITH CELLULAR ENVIRONMENT SUB	
CA2924	1.5	1.4	1.2	FET33	complemer	cell surface ferroxidase	orf19.1168	11908	CaFET32	CELLULAR TRANSPORT AND TRANSPORT MECHANISMS REGULATION OF/INTERACTION WITH CELLULAR ENVIRONMENT SUB	
CA2925	1.0	1.0	1.0	IPF7827	10229638..	unknown function	orf19.4210	7827	CaFET33	CELLULAR	oxidoreductase activity
CA2926	1.0	1.0	1.1	UBA3	complemer	Ubiquitin-like protein a	orf19.4209	7830	IPF7827	UNCLASSI	molecular_function unknown
CA2927	0.9	0.9	1.0	RAD52	complemer	Nuclear ribonucleoprot	orf19.4208	7833	CaUBA3	PROTEIN FATE [folding modification destination]	
CA2928	1.0	1.0	0.9	SME1	complemer	Nuclear ribonucleoprotein E		7835	CaRAD52	CELL CYC	DNA binding
CA2929	0.9	1.1	1.0	IPF7838	10235491..	similar to Saccharomy	orf19.4206	7838	CaSME1	TRANSCR	RNA binding
CA2930	0.9	0.9	0.7	IPF7840	10237526..	similar to Saccharomy	orf19.4204	7840	IPF7838	CELL CYC	hydrolase activity
CA2931	1.0	0.9	0.8	IPF7841	10238778..	similar to Saccharomy	orf19.4203	7841	IPF7840	PROTEIN S	structural molecule activity
CA2932	1.0	1.1	1.1	NHX1	complemer	NA+-H+ antiporter	orf19.1167	7842	IPF7841	CELL CYC	structural molecule activity
CA2934	1.0	0.9	1.0	IPF11515	10249547..	similar to Saccharomy	orf19.3329	11515	CaNHX1	CELLULAR	transporter activity
CA2935	0.9	0.9	0.9	IPF11521	10254612..	unknown function	orf19.3328	11521	IPF11515	Lipid fatty-a	hydrolase activity
CA2936	1.1	1.1	1.1	IPF8326	10256835..	similar to Saccharomy	orf19.3327	8326	IPF11521	UNCLASSI	transcription regulator activity
CA2937	1.0	1.2	1.1	RPS21B.3	10259199..	ribosomal protein S21, 3-prime en		8325	IPF8326	TRANSCR	transferase activity
CA2938	1.8	1.4	1.3	IPF8321	10261346..	similar to Saccharomy	orf19.3325	8321	CaRPS21B	PROTEIN S	structural molecule activity
CA2939	1.1	1.2	1.0	TIF1	10264139..	translation initiation fac	orf19.3324	8319	IPF8321	C-compour	transferase activity
CA2940	1.0	1.0	1.1	IPF8318	10265728..	unknown function	orf19.3323	8318	CaTIF1	PROTEIN S	translation regulator activity
CA2941	1.1	0.9	1.0	CVB1	complemer	vacuole biogenesis co	orf19.1970	6289	IPF8318	SUBCELLULAR LOCALISATION	
CA2942	1.0	0.9	1.1	IPF6291	10270806..	Secretory Stress Resp	orf19.1969	6291	CaCVB1	No significant S.c. match	
CA2943	0.9	1.0	1.0	MAK31	complemer	Involved in stability of L-A dsRNA-		6293	IPF6291	No significant S.c. match	
CA2944	1.0	3.8	1.0	IPF6294	10272504..	unknown function	orf19.1968	6294	CaMAK31	No significa	transferase activity
CA2945	1.0	1.0	1.0	IMG1	complemer	Ribosomal protein, mit	orf19.1967	6295	IPF6294	No significant S.c. match	
CA2946	1.0	1.0	1.0	IPF6296	complemer	putative methyltransfer	orf19.1966	6296	CaIMG1	ENERGY F	structural molecule activity
CA2947	1.1	1.0	1.0	IPF6298	complemer	unknown function	orf19.1964	6298	IPF6296	CLASSIFIC	transferase activity
CA2948	1.1	1.1	1.0	GDS1	complemer	nam9-1 suppressor (by	orf19.1963	6301	IPF6298	No significant S.c. match	
CA2949	1.0	1.0	1.0	IPF14506	complemer	unknown function	orf19.9516	14506	CaGDS1	CLASSIFIC	molecular_function unknown
CA2950	1.0	1.1	1.0	CLN2	complemer	G1/S-SPECIFIC CYCL	orf19.9515	10874	IPF14506	No significant S.c. match	
CA2951	1.1	1.9	1.0	SBP1	10299689..	RNA binding protein-lik	orf19.5854	10692	CaCLN2	CELL CYC	protein kinase activity,enzyme regulator activity
CA2952	1.1	1.0	1.1	IPF19671	10301502..	unknown function		19671	CaSBP1	TRANSCR	RNA binding
CA2953	1.0	1.0	1.0	MBP1	complemer	transcription factor (by	orf19.5855	10688	IPF19671	Lipid fatty-acid and isoprenoid metabolism ""SUBCELLULAR LOCALISATION TRANSPORT FACILITATION	
CA2954	0.9	0.9	1.0	IPF10685	complemer	unknown function	orf19.5856	10685	CaMBP1	CELL CYC	DNA binding
CA2955	1.0	1.1	1.1	IPF15201	10306785..	Unknown function	orf19.5857	15201	IPF10685	SUBCELLULAR LOCALISATION	
CA2956	1.0	1.2	1.1	EGD2	complemer	Nascent polypeptide a	orf19.5858	15200	IPF15201	No significant S.c. match	
CA2957	0.9	1.1	1.0	DAL53	10309893..	allantoate permease (b	orf19.5859	8598	CaEGD2	C-compour	chaperone activity
CA2958	1.0	1.1	1.0	KRE9	10313399..	cell wall synthesis prot	orf19.5861	8595	CaDAL53	CELLULAR TRANSPORT AND TRANSPORT MECHANISMS SUBCELLULAR LOCALISATION TRANSPORT FACILITATION	
CA2959	0.9	0.9	0.8	IPF8591	10315498..	putative arginase famil	orf19.5862	8591	CaKRE9	C-compour	molecular_function unknown
CA2960	1.0	0.9	0.9	IPF8590	10317643..	unknown function	orf19.5863	8590	IPF8591	Amino acid metabolism Nitrogen and sulphur metabolism SUBCELLULAR LOCALISATION	
CA2961	1.0	0.9	0.8	URK1	complemer	uridine kinase (by hom	orf19.5864	8589	IPF8590	No significant S.c. match	
CA2962	1.0	0.9	1.0	PRP2	10320357..	RNA-dependent ATPa	orf19.5865	8587	CaURK1	Nucleotide	transferase activity
CA2963	1.0	1.0	1.0	GRP3	complemer	dihydroflavonol-4-redu	orf19.5611	13178	CaPRP2	TRANSCR	RNA binding,helicase activity
CA2964	1.1	1.1	1.0	IPF13176.3	complemer	ornithine carbamoyltra	orf19.5610	13176	CaGRP3	Metabolism	oxidoreductase activity
CA2965	1.0	0.9	1.0	IPF13174	complemer	unknown function	orf19.5609	13174	IPF13176.3	Amino acid	transferase activity
CA2966	0.9	1.0	1.0	RPC34	10334066..	DNA-directed RNA pol	orf19.5608	16279	IPF13174	No significant S.c. match	
CA2967	1.1	1.1	1.0	IPF12513	10338043..	unknown function	orf19.5605	12513	CaRPC34	TRANSCR	nucleotidyltransferase activity
CA2968	1.1	1.1	1.0	BMR1	10342240..	benomyl/methothrexat	orf19.5604	16346	IPF12513	UNCLASSI	protein binding
CA2969	1.1	1.1	1.1	IPF19772	10345160..	unknown function	orf19.5602	19772	CaBMR1	CELL RES	transporter activity
CA2970	0.9	1.0	1.0	IPF14448	10347311..	unknown function	orf19.5601	14448	IPF19772	No significant S.c. match	
CA2971	1.1	1.1	1.0	MDL2.5F	10348887..	ATP-binding transporte	orf19.5600	14449	IPF14448	No significant S.c. match	
CA2972	0.9	1.0	1.1	MDL2.3F	10350636..	ATP-binding transporte	orf19.5599	20121	CaMDL2.5	TRANSPORT FACILITATION	
CA2973	1.2	1.2	1.2	IPF14452.F	complemer	F1-ATPase epsilon subunit (by ho		14452	CaMDL2.3	TRANSPORT FACILITATION	
CA2974	0.9	0.9	1.0	IPF12540	10359969..	unknown function	orf19.8824	12540	IPF14452.r	No significant S.c. match	
CA2975	1.2	1.1	1.2	ARO9	10363904..	aromatic amino acid ar	orf19.8822	14677	IPF12540	No significant S.c. match	

CA2976	1.2	1.4	1.0	IPF14676	10365923..	unknown function	orf19.8821	14676	CaARO9	Amino acid	transferase activity
CA2977	1.1	1.2	1.1	HOM3	complemer	Aspartokinase (by hom	orf19.1235	14675	IPF14676	UNCLASSI	molecular_function unknown
CA2979	1.2	1.0	1.1	ADE4	complemer	amidophosphoribosyltr	orf19.1233	13283	CaHOM3	Amino acid	transferase activity
CA2980	0.9	0.9	1.2	GOG5	10390348..	GDP-mannose transpo	orf19.1232	13284	CaADE4	Nucleotide	transferase activity
CA2981	1.1	1.0	0.9	CSE1.5F	10392640..	Importin-beta-like prot	orf19.1231	13285	CaGOG5	CELLULAF	transporter activity
CA2982	1.0	0.9	0.9	CSE1.3F	10394151..	Importin-beta-like prot	orf19.8815	16520	CaCSE1.5I	PROTEIN I	protein binding
CA2983	1.0	1.1	0.9	IPF18396	complemer	unknown function	orf19.8814	18396	CaCSE1.3I	PROTEIN FATE [folding modification destination] ""CELLULAR TRANSPORT AND TRANSPORT MECHANISMS CONTROL OF CELLUL	
CA2984	1.0	1.0	1.0	IPF12606.3	10397204..	unknown function, 3-prime end		5109	IPF18396	No significant S.c. match	
CA2985	1.0	1.0	1.0	SET1	10399009..	Chromatin regulatory p	orf19.6009	5105	IPF12606.3	UNCLASSIFIED PROTEINS	
CA2986	1.0	1.1	1.1	CDC5	10403402..	Cell-cycle protein kina	orf19.6010	5102	CaSET1	CELL CYC	transferase activity
CA2987	1.0	1.0	1.1	RPB11.3	complemer	DNA-directed RNA polymerase II s		5100	CaCDC5	CELL CYC	protein kinase activity
CA2988	1.0	1.0	1.0	SIN3.EXOI	10407146..	Histone deacetylase by homology		5097	CaRPB11.3	TRANSCR	nucleotidyltransferase activity
CA2989	0.9	0.9	0.7	SIN3.EXOI	10407748..	Histone deacetylase b	orf19.6011	5096	CaSIN3.ex	Lipid fatty-acid and isoprenoid metabolism ""TRANSCRIPTION CELL FATE SUBCELLULAR LOCALISATION	
CA2990	1.0	1.1	1.0	IPF5092	complemer	unknown function	orf19.6012	5092	CaSIN3.ex	Lipid fatty-a	hydrolase activity
CA2991	0.9	1.1	1.0	IPF5088.3	complemer	unknown function, 3-pr	orf19.6013	5088	IPF5092	UNCLASSI	molecular_function unknown
CA2992	0.8	0.7	0.9	RRS1	complemer	Regulator for ribosome	orf19.6014	5085	IPF5088.3	UNCLASSI	molecular_function unknown
CA2993	1.0	1.1	1.0	IPF9118	complemer	unknown function	orf19.6118	9118	CaRRS1	TRANSCR	molecular_function unknown
CA2994	1.0	1.0	1.1	IPF9116	10425599..	unknown function	orf19.6119	9116	IPF9118	CELLULAF	enzyme regulator activity
CA2995	1.0	1.0	1.0	IPF9113	10428585..	unknown function	orf19.6121	9113	IPF9116	No significant S.c. match	
CA2996	0.9	1.0	1.0	IPF9108	complemer	similar to Saccharomy	orf19.6124	9108	IPF9113	TRANSCR	molecular_function unknown
CA2997	0.9	0.9	0.8	KGD2	complemer	2-oxoglutarate dehydr	orf19.6126	8851	IPF9108	C-compour	transcription regulator activity
CA2998	1.0	3.4	1.0	LPD1	10438250..	dihydrolipoamide dehy	orf19.6127	8847	CaKGD2	C-compour	molecular_function unknown
CA2999	1.0	0.9	1.0	MRPL8	10440104..	mitochondrial 60s ribo	orf19.6129	8844	CaLPD1	Amino acid	transporter activity
CA3000	1.0	1.0	1.5	TSC1.5	10441073..	3-ketosphinganine red	orf19.6131	8842	CaMRPL8	PROTEIN S	structural molecule activity
CA3001	0.9	1.1	1.0	IPF8841	complemer	unknown function	orf19.6132	8841	CaTSC1.5	Lipid fatty-a	oxidoreductase activity
CA3002	0.9	0.8	1.0	PIF1	complemer	mitochondrial DNA hel	orf19.6133	19673	IPF8841	UNCLASSI	molecular_function unknown
CA3003	0.9	0.9	0.9	IPF15741	10448869..	similar to Saccharomy	orf19.1362	15741	CaPIF1	CELL CYC	DNA binding,helicase activity
CA3004	1.0	0.9	1.0	KIN28	10452289..	cyclin-dependent ser/t	orf19.1361	15293	IPF15741	UNCLASSI	molecular_function unknown
CA3005	1.2	1.2	1.1	CC43	10456995..	Cell Division Control -l	orf19.1361	19967	CaKIN28	CELL CYC	protein kinase activity,transcription regulator activity
CA3006	0.9	1.0	1.0	IPF12152	complemer	Unknown function	orf19.1361	12152	CaCC43	CELL CYCLE AND DNA PROCESSING CELLULAR COMMUNICATION/SIGNAL TRANSDUCTION MECHANISM REGULATION OF/INTI	
CA3007	0.9	1.0	1.0	IPF12148	complemer	Unknown function	orf19.1361	12148	IPF12152	UNCLASSI	molecular_function unknown
CA3008	0.9	0.9	0.9	IPF12147	10461455..	unknown function	orf19.1361	12147	IPF12148	UNCLASSI	molecular_function unknown
CA3009	1.0	1.0	1.1	MRPL19	complemer	Ribosomal protein (by	orf19.1361	14569	IPF12147	UNCLASSI	oxidoreductase activity
CA3010	0.9	0.9	1.0	IPF14568	10467495..	unknown function	orf19.1361	14568	CaMRPL19	PROTEIN S	structural molecule activity
CA3011	0.9	1.1	0.8	CTA1	10470262..	catalase A, peroxisom	orf19.6229	20124	IPF14568	UNCLASSI	enzyme regulator activity
CA3012	1.0	1.0	1.1	IPF14013	10472451..	unknown function	orf19.6227	14013	CaCTA1	CELL RES	oxidoreductase activity
CA3013	1.1	1.2	1.0	PET117	complemer	cytochrome c oxidase assembly fa		20125	IPF14013	No significant S.c. match	
CA3014	0.8	0.8	0.7	IPF11915	10476184..	similar to Saccharomy	orf19.1360	11915	CaPET117	PROTEIN I	molecular_function unknown
CA3015	1.1	0.9	1.0	IPF10029	10480426..	unknown function	orf19.1139	10029	IPF11915	CLASSIFIC	protein kinase activity,enzyme regulator activity
CA3016	1.1	1.1	1.0	IPF10027	10482517..	unknown function	orf19.1139	10027	IPF10029	UNCLASSI	molecular_function unknown
CA3017	1.0	1.0	1.0	IPF10021	10486002..	unknown function	orf19.1139	10021	IPF10027	PROTEIN SYNTHESIS CELL FATE SUBCELLULAR LOCALISATION	
CA3018	1.1	1.2	1.1	SAH1	10488537..	S-adenosyl-L-homocys	orf19.3911	10018	IPF10021	Nitrogen ar	DNA binding,transcription regulator activity
CA3019	1.2	1.0	1.1	IPF15494	complemer	putative ribonuclease (	orf19.3910	15494	CaSAH1	Metabolism	hydrolase activity
CA3020	1.7	1.1	1.4	IPF15492	complemer	unknown function	orf19.3908	15492	IPF15494	Nucleotide metabolism	
CA3021	1.1	1.0	1.0	IPF17640	complemer	unknown function	orf19.3906	17640	IPF15492	CELL RESCUE DEFENSE AND VIRULENCE ""SUBCELLULAR LOCALISATION TRANSPORT FACILITATION UNCLASSIFIED PROTEI	
CA3022				IPF17642	complement(10495936..10496577)				IPF17640	No significant S.c. match	
CA3023	0.9	0.9	0.9	IPF11508	10497887..	unknown function	orf19.3904	11508			
CA3024	0.9	0.9	1.0	IPF11506	complemer	unknown function	orf19.3903	11506	IPF11508	CELL RESCUE DEFENSE AND VIRULENCE	
CA3025	0.9	0.9	0.8	IPF11503	complemer	unknown function	orf19.3902	11503	IPF11506	Lipid fatty-acid and isoprenoid metabolism ""SUBCELLULAR LOCALISATION	
CA3026	1.0	1.0	0.9	IPF11499.F	complemer	unknown function	orf19.1138	11499	IPF11503	CELLULAR TRANSPORT AND TRANSPORT MECHANISMS	
CA3027	1.0	1.2	1.1	IPF20126	complemer	putative chromosome s	orf19.1138	20126	IPF11499.r	No significant S.c. match	
CA3028	0.9	1.0	1.0	IPF18393	complemer	unknown function	orf19.1138	18393	IPF20126	UNCLASSI	transporter activity
CA3029	1.0	1.0	1.0	IPF13825	10509253..	similarity to serine/thre	orf19.223	13825	IPF18393	UNCLASSIFIED PROTEINS	
CA3030	2.2	1.5	1.8	IPF19968	complemer	putative cell wall prote	orf19.220	19968	IPF13825	CLASSIFIC	protein kinase activity
CA3031	1.1	0.9	1.0	IPF12324	10520768..	unknown function	orf19.217	12324	IPF19968	CELL RES	structural molecule activity
CA3032	1.0	1.0	0.9	IPF12319	complemer	unknown function	orf19.216	12319	IPF12324	TRANSCR	molecular_function unknown
CA3033	1.1	1.1	1.0	IPF13202	10527690..	unknown function	orf19.215	13202	IPF12319	CELL RESCUE DEFENSE AND VIRULENCE	
CA3034	1.2	1.0	1.1	RPN12	10529829..	26S proteasome regul	orf19.213	13198	IPF13202	UNCLASSI	molecular_function unknown
CA3035	1.0	1.0	1.0	VPS28	complemer	involved in vacuolar tr	orf19.212	13197	CaRPN12	UNCLASSI	peptidase activity
CA3036	1.0	1.0	0.9	IPF13443	10531753..	unknown function	orf19.211	13443	CaVPS28	CELLULAF	molecular_function unknown
CA3037	1.0	1.0	1.0	IPF13442	complemer	unknown function	orf19.210	13442	IPF13443	No significant S.c. match	
CA3038	1.0	0.9	0.9	IPF13438	complemer	unknown function	orf19.209	13438	IPF13442	UNCLASSI	molecular_function unknown
CA3040	1.0	1.0	1.1	IPF7306	complemer	putative permease (by	orf19.2425	7306	IPF13438	CELL FATI	molecular_function unknown
CA3041	0.9	0.9	1.1	IPF7303	complemer	unknown function	orf19.2423	7303	IPF7306	C-compour	molecular_function unknown
CA3042	1.1	1.1	1.0	ARC1	10548031..	G4 nucleic acid binding	orf19.2422	7302	IPF7303	TRANSCR	molecular_function unknown
CA3043	1.0	1.1	0.9	DOM34	10549385..	probable involvement	orf19.2419	7299	CaARC1	TRANSCR	RNA binding
CA3044	1.0	1.0	0.9	IPF7298	complemer	unknown function	orf19.2418	7298	CaDOM34	CELL CYC	molecular_function unknown
CA3045	1.1	1.0	1.1	IPF7297.3	complemer	similar to Saccharomyces cerevisi		7297	IPF7298	No significant S.c. match	
CA3046	1.1	1.0	1.0	IPF7295	10552547..	unknown function	orf19.2417	7295	IPF7297.3	CELL CYC	motor activity
CA3047	1.0	1.1	1.1	MSE1	10555973..	Mitochondrial glutamyl	orf19.9953	7294	IPF7295	CELL CYC	molecular_function unknown
CA3048	1.0	1.1	1.0	IPF14991	10559150..	unknown function	orf19.9950	14991	CaMSE1	PROTEIN S	ligase activity
CA3049	1.1	1.1	1.0	IPF14990	complemer	unknown function	orf19.9949	14990	IPF14991	UNCLASSI	molecular_function unknown

CA3050	1.0	0.9	1.0	IPF13042	10561005..	similar to Saccharomy	orf19.9948	13042	IPF14990	UNCLASSI	transporter activity
CA3051	1.0	1.0	0.9	IPF13043	10563863..	unknown function	orf19.2408	13043	IPF13042	PROTEIN I	molecular_function unknown
CA3052	0.9	1.1	1.0	DPS1	10564964..	aspartyl-tRNA syntheta	orf19.9945	13045	IPF13043	UNCLASSI	molecular_function unknown
CA3053	0.9	0.9	1.3	GTR2	10567059..	GTP-binding protein (b	orf19.9944	13046	CaDPS1	PROTEIN S	ligase activity
CA3055	1.0	1.1	1.0	HIR2	complemer	Histone transcription re	orf19.1177	2951	CaGTR2	UNCLASSI	hydrolase activity
CA3056	1.0	1.0	1.0	IPF2953	complemer	similar to Saccharomy	orf19.4294	2953	CaHIR2	TRANSCR	transcription regulator activity
CA3057	1.0	1.0	1.0	IPF2954	10576198..	unknown function	orf19.4293	2954	IPF2953	CELLULAF	molecular_function unknown
CA3058	1.0	1.0	1.1	IPF2955	complemer	unknown function	orf19.4292	2955	IPF2954	UNCLASSI	molecular_function unknown
CA3059	1.0	1.0	1.0	TRR1	complemer	Thioredoxin reductase	orf19.1176	2959	IPF2955	PROTEIN FATE [folding modification destination] ""CELLULAR TRANSPORT AND TRANSPORT MECHANISMS SUBCELLULAR LOCAL	
CA3061	0.9	1.0	0.9	IPF2965	complemer	unknown function	orf19.1176	2965	CaTRR1	Nitrogen ar	transporter activity
CA3062	1.0	1.0	1.0	IPF2968	complemer	unknown function	orf19.1176	2968	IPF2965	C-compound and carbohydrate metabolism	
CA3063	1.0	1.1	1.0	IPF2971	complemer	unknown function	orf19.4284	2971	IPF2968	No significant S.c. match	
CA3064	0.9	1.1	1.0	IPF2973	10589587..	unknown function	orf19.4283	2973	IPF2971	CELL CYC	protein kinase activity,enzyme regulator activity
CA3065	0.9	1.0	0.9	IPF19775	10593296..	unknown function	orf19.1175	19775	IPF2973	PROTEIN FATE [folding modification destination] ""SUBCELLULAR LOCALISATION	
CA3066	1.0	1.2	1.0	IPF15813	10595073..	unknown function	orf19.4281	15813	IPF19775	No significant S.c. match	
CA3067	1.0	1.0	0.9	IPF15811	complemer	unknown function	orf19.1175	15811	IPF15813	TRANSCR	molecular_function unknown
CA3068	1.0	1.0	1.0	LIP2	10604170..	Secretory lipase	orf19.4804	6120	IPF15811	No significant S.c. match	
CA3069	1.0	1.1	0.9	IPF6117	10606420..	unknown function	orf19.4805	6117	CaLIP2	Other virulence attributes	
CA3070	1.0	0.9	0.9	PPA2	10609643..	Mitochondrial inorganic	orf19.4807	6114	IPF6117	UNCLASSI	molecular_function unknown
CA3071	1.0	1.0	0.9	NUP188	complemer	Nucleoporin	orf19.4808	6113	CaPPA2	Phosphate	hydrolase activity
CA3072	1.0	1.0	1.1	ERG12	10616174..	Mevalonate kinase (by	orf19.4809	6109	CaNUP188	CELLULAF	structural molecule activity
CA3073	1.0	1.0	0.9	IPF6108	complemer	putative tricarboxylate	orf19.4811	6108	CaERG12	Lipid fatty-a	transferase activity
CA3074	1.0	0.9	1.0	IPF6106	10618742..	similar to Bacillus halo	orf19.4812	6106	IPF6108	TRANSPO	molecular_function unknown
CA3075	0.9	1.0	1.0	IPF6105	complemer	similar to Saccharomy	orf19.4813	6105	IPF6106	No significant S.c. match	
CA3076	1.0	1.0	1.0	IPF6101.3	10621306..	unknown function, 3-pr	orf19.4814	6101	IPF6105	Nucleotide	ligase activity
CA3077				YTM1	10622006..10623406				IPF6101.3	No significant S.c. match	
CA3078	0.9	5.3	1.0	IPF19970	complemer	unknown function	orf19.4816	19970			
CA3079	0.9	0.9	1.0	RAM2	10625422..	geranylgeranyltransfer	orf19.4817	15519	IPF19970	UNCLASSI	molecular_function unknown
CA3080	0.9	0.9	1.0	IPF14634	complemer	APP-binding protein 1	orf19.4153	14634	CaRAM2	Lipid fatty-a	transferase activity
CA3081	1.2	1.6	1.0	EFT3	10638188..	translation elongation f	orf19.1162	11638	IPF14634	PROTEIN FATE [folding modification destination]	
CA3082	1.0	0.9	1.0	IPF11627	10644145..	unknown function	orf19.1162	11627	CaEFT3	PROTEIN S	translation regulator activity
CA3083	1.0	0.9	1.0	IPF11262	10644491..	unknown function	orf19.1162	11626	IPF11627	No significant S.c. match	
CA3084	1.9	3.2	1.5	IPF11625	10645394..	unknown function	orf19.4149	11625	IPF11262	Nucleotide	molecular_function unknown
CA3085	1.1	1.2	1.2	IPF17086	10647065..	unknown function	orf19.4148	17086	IPF11625	No significant S.c. match	
CA3086	1.1	1.1	1.1	GLR1	10648979..	by similarity to S. cere	orf19.1162	14902	IPF17086	No significant S.c. match	
CA3087	1.0	1.1	1.1	SMD3	complemer	core snRNP protein (by	orf19.1162	14903	CaGLR1	CELL RES	transporter activity
CA3088	1.0	1.0	0.9	IPF9826	10652887..	unknown function	orf19.1162	9826	CaSMD3	TRANSCR	RNA binding
CA3089	0.8	0.9	0.8	IPF9825	10656457..	unknown function		9825	IPF9826	TRANSCR	transcription regulator activity
CA3090	1.1	1.0	1.0	IPF18385	complemer	unknown function	orf19.1161	18385	IPF9825	UNCLASSI	molecular_function unknown
CA3091	1.0	0.9	1.0	IPF9821.5f	10659481..	unknown function, 5-pr	orf19.1161	19600	IPF18385	UNCLASSI	molecular_function unknown
CA3092	0.9	1.0	1.0	IPF9821.3f	10660040..	unknown function, 3-pr	orf19.1161	9821	IPF9821.5f	TRANSPORT FACILITATION	
CA3093	1.1	1.0	1.0	TOF1	complemer	Topoisomerase I intera	orf19.1161	9818	IPF9821.3f	TRANSPO	transporter activity
CA3094	0.5	0.6	0.6	IPF11548	10671694..	serine/threonine protei	orf19.1133	11548	CaTOF1	CELL CYC	molecular_function unknown
CA3095	1.2	1.3	1.2	CDC28	10674380..	CELL DIVISION CONT	orf19.3856	11549	IPF11548	CELL RES	protein kinase activity
CA3096	1.1	1.0	1.0	IPF11551	10675988..	unknown function	orf19.3858	11551	CaCDC28	CELL CYC	protein kinase activity
CA3097	1.1	1.1	1.2	IPF6600	10676828..	unknown function	orf19.3859	6600	IPF11551	UNCLASSIFIED PROTEINS	
CA3098	1.1	2.0	1.1	SIS1	10678451..	heat shock protein (by	orf19.3861	6598	IPF6600	Lipid fatty-a	oxidoreductase activity
CA3099	1.0	1.0	1.0	LST8	10679824..	required for transport c	orf19.3862	6595	CaSIS1	CELL CYC	chaperone activity
CA3100	1.0	0.9	0.9	IPF6594	complemer	unknown function	orf19.3863	6594	CaLST8	CELLULAF	protein binding
CA3101	1.0	0.9	1.0	IPF6593	complemer	similar to Saccharomy	orf19.3865	6593	IPF6594	TRANSCRIPTION SUBCELLULAR LOCALISATION	
CA3102	0.9	0.9	0.9	RLP7	10689617..	ribosomal-like proteins	orf19.3867	6586	IPF6593	TRANSCR	transcription regulator activity
CA3103	1.0	0.9	0.9	IPF12086	10696183..	unknown function	orf19.1390	12086	CaRLP7	CLASSIFIC	RNA binding
CA3104	0.7	0.7	0.5	IPF12084	10697034..	unknown function	orf19.1390	12084	IPF12086	UNCLASSI	molecular_function unknown
CA3105	0.5	0.4	1.1	IPF12083	10698135..	unknown function	orf19.6553	12083	IPF12084	UNCLASSI	molecular_function unknown
CA3106	0.8	0.9	0.8	IPF11821	10699783..	unknown function	orf19.6552	11821	IPF12083	CELLULAR TRANSPORT AND TRANSPORT MECHANISMS SUBCELLULAR LOCALISATION	
CA3107	1.0	1.0	0.9	GOS1	complemer	SNARE protein of Golg	orf19.6551	11824	IPF11821	SUBCELLU	oxidoreductase activity
CA3108	0.6	0.4	0.9	IPF11826	10702398..	unknown function	orf19.6550	11826	CaGOS1	PROTEIN I	transporter activity
CA3109	0.9	1.0	0.6	IPF11829	complemer	unknown function	orf19.6548	11829	IPF11826	UNCLASSI	molecular_function unknown
CA3110	1.5	1.5	1.0	LPI9	10706896..	Microtubule-associatec	orf19.6544	15433	IPF11829	Nitrogen ar	molecular_function unknown
CA3111	1.0	1.1	1.1	RPL5	complemer	ribosomal protein (by h	orf19.6541	10601	CaLPI9	CLASSIFIC	molecular_function unknown
CA3112	0.5	1.2	0.4	PFK2	complemer	6-phosphofructokinase	orf19.6540	10598	CaRPL5	PROTEIN S	RNA binding
CA3113	0.9	0.9	1.0	IPF15737	10717001..	simailar to Saccharomy	orf19.1389	15737	CaPFK2	C-compour	transferase activity
CA3114	1.0	1.0	1.1	IPF18384	10719606..	unknown function		18384	IPF15737	CELL CYC	hydrolase activity
CA3115	1.6	1.2	1.9	ECM33.3	10725212..	cell wall biogenesis, 3-prime end (		5366	IPF18384	No significant S.c. match	
CA3116	0.8	0.7	0.9	LAB2	complemer	LIPOATE BIOSYNTHE	orf19.3010	5364	CaECM33.	CELL CYC	molecular_function unknown
CA3117	0.9	0.9	0.9	IPF5363	10727856..	unknown function	orf19.3009	5363	CaLAB2	Metabolism	ligase activity
CA3118	1.0	1.0	1.1	COQ4	10730084..	ubiquinone biosynthes	orf19.3008	5361	IPF5363	UNCLASSI	molecular_function unknown
CA3119	1.1	0.9	1.1	IPF5360.3	10731552..	unknown function, 3-prime end		5360	CaCOQ4	Metabolism	molecular_function unknown
CA3120	1.0	0.9	0.9	IPF5358	complemer	unknown function	orf19.3007	5358	IPF5360.3	UNCLASSIFIED PROTEINS	
CA3121	0.9	1.0	0.9	GGA1	10733426..	Arf-binding protein	orf19.3006	5357	IPF5358	UNCLASSI	molecular_function unknown
CA3122	0.9	1.1	1.0	IPF5356	complemer	unknown function	orf19.3004	5356	CaGGA1	CELLULAF	molecular_function unknown
CA3123	1.0	2.0	1.1	RPL6.3	complemer	ribosomal protein, 3-prime end		5354	IPF5356	UNCLASSI	molecular_function unknown

CA3124	1.0	1.1	1.1	IPF5353.3	complemer	unknown function, 3-pr	orf19.3003	5353	CaRPL6.3	PROTEIN !	RNA binding
CA3125	1.1	1.0	1.0	IPF8921.5E	complemer	unknown function, 5-prime end		8921	IPF5353.3	UNCLASSI	molecular_function unknown
CA3126	0.6	1.1	0.8	GCN4	complemer	transcriptional activato	orf19.1358	8919	IPF8921.5€	No significant S.c. match	
CA3127	0.8	0.9	0.8	IPF8915	complemer	unknown function	orf19.1359	8915	CaGCN4	Amino acid	DNA binding
CA3128	0.9	1.0	0.9	IPF8914	complemer	unknown function	orf19.1360	8914	IPF8915	UNCLASSI	molecular_function unknown
CA3129	1.0	0.8	0.9	OST4	10756389..	oligosaccharyltransferase subunit		8912	IPF8914	CELL CYC	molecular_function unknown
CA3130	1.0	1.0	1.0	TIM23	10757192..	mitochondrial inner me	orf19.1361	8911	CaOST4	No significant S.c. match	
CA3131	1.1	1.0	1.0	IPF8910	complemer	unknown function	orf19.1362	8910	CaTIM23	PROTEIN I	transporter activity
CA3132	0.6	0.9	0.5	IPF8904	10761903..	unknown function	orf19.1363	8904	IPF8910	CLASSIFICATION NOT YET CLEAR-CUT	
CA3133	1.1	1.0	1.0	IPF16939	complemer	unknown function	orf19.1364	16939	IPF8904	UNCLASSI	molecular_function unknown
CA3134	0.9	0.9	0.9	IPF11499.F	10765987..	unknown function	orf19.1365	19972	IPF16939	UNCLASSIFIED PROTEINS	
CA3135	1.0	0.9	1.0	IPF8990	10767918..	unknown function	orf19.1366	8990	IPF11499.r	No significant S.c. match	
CA3136	1.0	1.0	1.0	IPF8989	complemer	unknown function	orf19.1367	8989	IPF8990	No significant S.c. match	
CA3137	1.1	1.1	1.0	IPF8976	10779028..	unknown function	orf19.8949	8976	IPF8989	No significa	molecular_function unknown
CA3138	0.9	1.0	0.9	SAP2	10790905..	aspartic protease	orf19.3708	9220	IPF8976	No significant S.c. match	
CA3139	1.1	1.1	1.1	YHB3	10795143..	flavohemoglobin (by hc	orf19.3710	9216	CaSAP2	PROTEIN FATE [folding modification destination] ""Other virulence attributes	
CA3140	1.1	1.1	1.0	IPF9214	10798230..	unknown function	orf19.3711	9214	CaYHB3	CELL RESCUE DEFENSE AND VIRULENCE ""SUBCELLULAR LOCALISATION	
CA3141	1.0	1.1	1.0	IPF9211.3f	complemer	unknown function, 3-pr	orf19.3712	9213	IPF9214	Metabolism of vitamins cofactors and prosthetic groups	
CA3142	0.9	0.9	0.7	IPF9211.5f	complemer	unknown function, 3-pr	orf19.3713	9211	IPF9211.3f	No significant S.c. match	
CA3143	1.0	1.0	1.0	IPF8295	10805134..	unknown function	orf19.3714	8295	IPF9211.5f	No significant S.c. match	
CA3144	1.0	0.9	1.0	ASF1	complemer	anti-silencing protein (t	orf19.3715	8293	IPF8295	No significant S.c. match	
CA3145	1.1	1.0	1.0	CDC9	complemer	DNA ligase (by homolc	orf19.6155	9796	CaASF1	TRANSCR	protein binding
CA3146	1.1	0.9	1.0	IPF9797	complemer	unknown function	orf19.6156	9797	CaCDC9	CELL CYC	ligase activity
CA3147	1.2	2.2	1.0	IPF9803	complemer	unknown function	orf19.6160	9803	IPF9797	No significa	molecular_function unknown
CA3148	1.0	1.0	0.9	IPF9808	10820281..	similar to Saccharomy	orf19.6163	9808	IPF9803	UNCLASSI	molecular_function unknown
CA3149	1.0	1.1	1.0	KGD1	10822581..	2-oxoglutarate dehydrc	orf19.6165	19778	IPF9808	CELL CYC	DNA binding
CA3150	1.0	1.0	1.0	IPF4035	complemer	unknown function	orf19.6166	4035	CaKGD1	C-compour	oxidoreductase activity
CA3151	1.0	1.1	1.0	IPF4033	complemer	similar to Saccharomy	orf19.6167	4033	IPF4035	Lipid fatty-acid and isoprenoid metabolism	
CA3152	1.2	1.1	1.2	IPF4032	10830274..	unknown function	orf19.6168	4032	IPF4033	C-compour	oxidoreductase activity
CA3153	0.5	0.5	0.3	FRP2	10834721..	member of the FRP fai	orf19.6169	4028	IPF4032	CELL CYCLE AND DNA PROCESSING CONTROL OF CELLULAR ORGANIZATION SUBCELLULAR LOCALISATION	
CA3154	0.9	1.0	1.0	FUN34.5E(	10836621..	unknown function, 5-prime end		18376	CaFRP2	C-compound and carbohydrate metabolism	
CA3156	1.0	1.0	1.0	IPF14455	10837414..	similar to Saccharomy	orf19.1589	14455	CaFUN34.!	C-compound and carbohydrate metabolism	
CA3157	1.2	1.1	1.1	IPF14456	complemer	unknown function	orf19.1588	14456	IPF14455	TRANSCR	transcription regulator activity
CA3158	1.0	1.1	0.9	IPF13131.3	10841576..	unknown function, 3-pr	orf19.1587	13131	IPF14456	UNCLASSI	molecular_function unknown
CA3159	1.0	0.9	1.1	PLC3	10843487..	phosphatidylinositol ph	orf19.1586	13130	IPF13131.3	C-compound and carbohydrate metabolism TRANSPORT FACILITATION	
CA3160	1.3	1.8	1.5	ZRT2	complemer	zinc transport protein (	orf19.1585	13128	CaPLC3	TRANSCRIPTION SUBCELLULAR LOCALISATION	
CA3161	1.1	1.1	1.0	IPF13121.3	complemer	unknown function, 3-pr	orf19.1584	13121	CaZRT2	REGULATI	transporter activity
CA3162	0.9	1.0	1.0	HOL5.3F	complemer	member of major facilit	orf19.1583	13181	IPF13121.3	TRANSPORT FACILITATION	
CA3163	0.9	0.9	1.0	HOL5.5F	complemer	member of major facilit	orf19.1582	13183	CaHOL5.3I	CELL RESCUE DEFENSE AND VIRULENCE ""TRANSPORT FACILITATION	
CA3164	0.9	0.8	1.0	IPF13187	10852889..	unknown function	orf19.1580	13187	CaHOL5.5I	CELL RESCUE DEFENSE AND VIRULENCE ""TRANSPORT FACILITATION	
CA3165	0.9	0.9	0.9	FMI1	complemer	processing of pre-ribos	orf19.1578	9638	IPF13187	No significant S.c. match	
CA3166	1.0	0.9	1.0	IPF9634	complemer	probable GATA zinc fir	orf19.1577	9634	CaFMI1	TRANSCR	RNA binding
CA3167	0.8	1.0	0.9	IPF9632	complemer	unknown function	orf19.1576	9632	IPF9634	UNCLASSI	DNA binding,transcription regulator activity
CA3168	1.0	0.8	1.0	PRS3	complemer	ribose-phosphate pyro	orf19.1575	9631	IPF9632	UNCLASSIFIED PROTEINS	
CA3169	1.6	1.0	1.5	IPF7686	10873121..	putative mitochondrial	orf19.1395	7686	CaPRS3	Amino acid	transferase activity
CA3170	0.9	0.9	1.0	IPF7685	complemer	putative GTPase activa	orf19.1396	7685	IPF7686	Phosphate	molecular_function unknown
CA3171	0.9	0.9	1.0	IPF7681	10877654..	unknown function	orf19.1397	7681	IPF7685	UNCLASSI	enzyme regulator activity
CA3172	1.0	0.9	0.9	IPF7676	10879160..	unknown function	orf19.1400	7679	IPF7681	Lipid fatty-acid and isoprenoid metabolism	
CA3173	1.0	0.9	1.0	IPF14145	complemer	unknown function	orf19.1401	14145	IPF7676	No significant S.c. match	
CA3174	1.0	1.0	1.0	CCT2	10888438..	chaperonin of the TCP	orf19.1402	11584	IPF14145	No significant S.c. match	
CA3175	1.1	1.0	1.1	IPF11587	10890905..	unknown function	orf19.1403	11587	CaCCT2	UNCLASSI	chaperone activity
CA3176	1.0	1.0	0.9	IPF11588	complemer	unknown function	orf19.1404	11588	IPF11587	CELLULAF	transporter activity
CA3177	1.1	1.0	1.1	IPF11589	complemer	similar to Saccharomy	orf19.1405	11589	IPF11588	UNCLASSIFIED PROTEINS	
CA3178	1.0	0.9	0.9	IPF19974	complemer	unknown function	orf19.1406	19974	IPF11589	Phosphate metabolism SUBCELLULAR LOCALISATION	
CA3179	1.0	1.0	1.1	YLF2	10901693..	GTP-binding protein	orf19.2128	12246	IPF19974	CELL CYCLE AND DNA PROCESSING	
CA3180	0.9	1.0	1.1	IPF12244	complemer	unknown function	orf19.2131	12244	CaYLF2	UNCLASSI	molecular_function unknown
CA3181	0.9	0.9	1.0	IPF12241	complemer	unknown function	orf19.2132	12241	IPF12244	UNCLASSI	ligase activity
CA3182	1.0	1.0	1.0	LIP4	10908164..	secretory lipase	orf19.2133	15657	IPF12241	UNCLASSIFIED PROTEINS	
CA3183	0.9	0.9	0.9	TSM1.3F	complemer	component of TFIID cc	orf19.2135	15656	CaLIP4	Other virulence attributes	
CA3184	1.1	1.0	1.0	TSM1.5F	complemer	component of TFIID cc	orf19.2136	13636	CaTSM1.3I	TRANSCR	transcription regulator activity
CA3185	1.0	1.0	1.0	IPF13637	complemer	unknown function	orf19.2137	13637	CaTSM1.5I	TRANSCRIPTION SUBCELLULAR LOCALISATION	
CA3186	1.2	0.9	1.0	ILS1	10915617..	isoleucyl-tRNA synthet	orf19.2138	13644	IPF13637	No significant S.c. match	
CA3187	1.0	1.0	1.0	IPF17094	complemer	unknown function	orf19.2143	17094	CaILS1	PROTEIN !	ligase activity
CA3188	1.0	1.0	1.0	IPF11610	complemer	similar to Saccharomy	orf19.2146	11610	IPF17094	UNCLASSI	molecular_function unknown
CA3189	0.9	0.8	0.8	IPF11607	complemer	unknown function	orf19.2149	11607	IPF11610	TRANSCR	transferase activity
CA3190	1.0	1.0	0.9	IPF11603	10926058..	unknown function	orf19.2150	11603	IPF11607	REGULATI	molecular_function unknown
CA3191	1.0	1.3	1.0	IPF11601	10927146..	unknown function	orf19.2151	11601	IPF11603	ENERGY !	transporter activity
CA3192	1.0	1.0	1.0	IPF11598	10929664..	by homology to S. cerv	orf19.2154	11598	IPF11601	CLASSIFIC	molecular_function unknown
CA3193	1.1	0.9	1.1	IPF3418	complemer	unknown function	orf19.6170	3418	IPF11598	C-compound and carbohydrate metabolism ENERGY SUBCELLULAR LOCALISATION	
CA3194	1.1	1.2	1.1	IPF3425	complemer	unknown function	orf19.6171	3423	IPF3418	CELL CYC	protein binding
CA3195	1.0	1.0	1.1	IPF3426	10936551..	unknown function	orf19.6173	3426	IPF3425	CELLULAF	structural molecule activity
CA3196	1.1	1.0	1.0	IPF3428	complemer	unknown function	orf19.6175	3428	IPF3426	C-compour	enzyme regulator activity

CA3197	1.1	1.1	1.1	SEC61	complemer	ER protein-translocatic	orf19.6176	3431	IPF3428	UNCLASSI	molecular_function unknown
CA3198	1.0	1.1	0.9	IPF3432	10939919..	Unknown function	orf19.6177	3432	CaSEC61	PROTEIN I	transporter activity
CA3199	1.1	1.0	0.9	FBP1	10940393..	Fructose-1,6-bisphosp	orf19.6178	3435	IPF3432	No significant S.c. match	
CA3200	1.0	1.0	1.0	IPF3439	10941463..	unknown function	orf19.6180	3439	CaFBP1	C-compour	hydrolase activity
CA3201	1.0	1.0	1.1	IPF3444.3f	complemer	unknown function, 3-pr	orf19.6182	3443	IPF3439	UNCLASSI	transporter activity
CA3202	1.0	0.9	1.0	IPF3444.5f	complemer	unknown function, 5-pr	orf19.6184	3444	IPF3444.3f	Lipid fatty-acid and isoprenoid metabolism ""TRANSCRIPTION SUBCELLULAR LOCALISATION	
CA3203	0.9	0.7	1.1	IPF3445	10945425..	Unknown function	orf19.6183	3445	IPF3444.5f	No significant S.c. match	
CA3204	1.0	1.0	1.0	IPF3446	complemer	Unknown function	orf19.6186	3446	IPF3445	PROTEIN I	transporter activity
CA3205	1.1	1.0	1.0	IPF3448	10946634..	Unknown function	orf19.6185	3448	IPF3446	UNCLASSIFIED PROTEINS	
CA3206	1.0	0.9	1.0	IPF3454	10947640..	unknown function	orf19.6187	3454	IPF3448	No significant S.c. match	
CA3207	0.9	0.9	1.0	IPF3456	10948707..	unknown function	orf19.6189	3456	IPF3454	No significant S.c. match	
CA3208	1.3	1.6	1.4	PSA1	complemer	GDP-mannose pyroph	orf19.6190	3458	IPF3456	UNCLASSI	molecular_function unknown
CA3209	1.3	1.8	0.9	CTA2.5.3F	complemer	transcriptional activator, 3-prime e		3460	CaPSA1	C-compour	nucleotidyltransferase activity
CA3211	1.0	1.0	1.0	IPF19977	complemer	unknown function	orf19.6192	19977	CaCTA2.5.	No significant S.c. match	
CA3212	0.9	0.9	0.9	MEF2	10966922..	translation elongation f	orf19.6208	8163	IPF19977	Lipid fatty-acid and isoprenoid metabolism ""SUBCELLULAR LOCALISATION	
CA3213	1.0	1.0	0.9	IPF8160	10969537..	unknown function	orf19.6205	8160	CaMEF2	PROTEIN S	translation regulator activity
CA3214	0.9	1.0	0.9	PUT3	complemer	putative positive activa	orf19.6203	8158	IPF8160	UNCLASSI	molecular_function unknown
CA3215	1.0	1.0	0.8	HCS1	complemer	putative DNA helicase	orf19.6199	13040	CaPUT3	Amino acid	transcription regulator activity
CA3216	1.0	1.0	1.2	IPF13038	complemer	unknown function		13038	CaHCS1	CELL CYC	DNA binding,helicase activity
CA3217	1.1	1.0	0.9	DHH1	complemer	RNA helicase by homc	orf19.6197	19979	IPF13038	No significa	molecular_function unknown
CA3218	1.0	1.1	1.0	IPF10005	10989742..	unknown function	orf19.6196	10005	CaDHH1	TRANSCR	protein binding
CA3219	1.0	1.0	1.0	IPF10003	complemer	similar to Saccharomyc	orf19.6195	10003	IPF10005	No significant S.c. match	
CA3220	0.9	0.9	0.9	IPF10001	complemer	unknown function	orf19.6194	10001	IPF10003	Nucleotide	RNA binding,hydrolase activity
CA3221	1.0	1.0	1.0	IPF10000.5	10994663..	unknown function, 5-pr	orf19.6193	10000	IPF10001	No significant S.c. match	
CA3222	1.0	0.9	1.1	IPF15883	complemer	unknown function	orf19.6283	17135	IPF10000.5	CELL CYC	protein kinase activity
CA3223	1.0	1.1	1.0	SRP102	10999078..	Signal recognition part	orf19.6284	17133	IPF15883	Nitrogen and sulphur metabolism REGULATION OF/INTERACTION WITH CELLULAR ENVIRONMENT	
CA3224	1.3	1.0	1.1	GLC7	complemer	Ser/thr phosphoproteir	orf19.6285	18366	CaSRP102	PROTEIN FATE [folding modification destination] ""SUBCELLULAR LOCALISATION	
CA3225	1.1	1.1	1.0	IPF9329	11003785..	unknown function	orf19.6286	9329	CaGLC7	C-compour	protein phosphatase activity
CA3226	0.9	1.0	1.2	RPS27	11005478..	ribosomal protein S27 (by homolog		9327	IPF9329	UNCLASSI	molecular_function unknown
CA3227	0.8	0.7	0.8	AAT21	complemer	aspartate aminotransfe	orf19.6287	9326	CaRPS27	PROTEIN S	structural molecule activity
CA3228	1.0	1.1	1.1	IPF9325	11007926..	unknown function	orf19.6288	9325	CaAAT21	Amino acid	transferase activity
CA3229	1.0	1.0	0.9	FUN30	complemer	helicases of the Snf2/F	orf19.6291	9324	IPF9325	No significant S.c. match	
CA3230	1.0	1.0	0.9	EMP24	complemer	component of the COF	orf19.6293	9318	CaFUN30	CELL CYC	molecular_function unknown
CA3231	0.9	1.0	0.9	IPF14348.3	complemer	unknown function, 3-prime end		14348	CaEMP24	CELLULAF	molecular_function unknown
CA3232	1.0	1.0	0.9	IPF17727.3	11016729..	unknown function, 3-prime end		14351	IPF14348.3	No significant S.c. match	
CA3233	0.9	0.9	1.1	MYO1	11018670..	myosin-1 isoform (type	orf19.6294	10851	IPF17727.3	No significant S.c. match	
CA3234	1.0	1.0	1.0	MAS2	complemer	processing peptidase,	orf19.6295	10850	CaMYO1	CELL CYC	motor activity
CA3235	1.0	1.1	1.0	SNF8	11027752..	involved in glucose der	orf19.6296	15696	CaMAS2	PROTEIN I	peptidase activity
CA3236	1.0	1.1	1.1	DEG1	complemer	pseudouridine synthas	orf19.6297	15695	CaSNF8	C-compour	molecular_function unknown
CA3237	1.0	1.0	1.0	SPB4	11030097..	ATP-dependent RNA h	orf19.6298	15694	CaDEG1	TRANSCR	lyase activity
CA3239	1.1	1.0	1.5	IPF10558	complemer	unknown function		10558	CaSPB4	TRANSCR	RNA binding,helicase activity
CA3240	1.1	1.0	0.9	IPF10559	11038512..	myosin-like protein (by	orf19.4683	10559	IPF10558	PROTEIN SYNTHESIS ""PROTEIN FATE [folding modification destination] ""SUBCELLULAR LOCALISATION	
CA3241	0.9	1.0	1.0	IPF7493	11046547..	putative permease (by	orf19.4682	7493	IPF10559	CELL CYC	molecular_function unknown
CA3242	1.0	1.0	1.0	RAT1	11048860..	5 -3 Exoribonuclease	orf19.4681	7491	IPF7493	C-compound and carbohydrate metabolism CELLULAR TRANSPORT AND TRANSPORT MECHANISMS SUBCELLULAR LOCALISATIO	
CA3243	1.0	1.1	1.0	IPF7489	complemer	unknown function	orf19.4680	7489	CaRAT1	TRANSCR	RNA binding
CA3244	1.0	0.9	1.0	AGP2	complemer	amino-acid permease (	orf19.4679	7487	IPF7489	No significant S.c. match	
CA3245	1.0	0.8	1.0	IPF19980	complemer	putative lipase (by hon	orf19.4678	19980	CaAGP2	Amino acid	transporter activity
CA3246	0.9	0.9	0.8	IPF15830	complemer	unknown function	orf19.4677	15830	IPF19980	Lipid fatty-a	hydrolase activity
CA3247	0.9	0.9	1.0	IPF15832	complemer	unknown function	orf19.4676	15832	IPF15830	UNCLASSI	molecular_function unknown
CA3248	1.0	0.9	1.0	IPF15834	11060569..	unknown function	orf19.4675	15834	IPF15832	UNCLASSI	molecular_function unknown
CA3249	0.9	0.9	0.9	GEF2	11068295..	Putative voltage-gated	orf19.1121	9175	IPF15834	No significant S.c. match	
CA3250	1.0	0.9	1.0	IPF4716	complemer	unknown Function	orf19.1122	4716	CaGEF2	CELLULAR TRANSPORT AND TRANSPORT MECHANISMS REGULATION OF/INTERACTION WITH CELLULAR ENVIRONMENT SUB	
CA3251	1.0	1.1	1.0	IPF4721	11076782..	unknown Function	orf19.3737	4721	IPF4716	Nucleotide metabolism CELL CYCLE AND DNA PROCESSING CELLULAR COMMUNICATION/SIGNAL TRANSDUCTION MECHANISM	
CA3252	1.0	1.1	0.9	IPF4722	complemer	unknown Function	orf19.3738	4722	IPF4721	UNCLASSIFIED PROTEINS	
CA3253	1.1	1.1	1.1	IPF4724	complemer	unknown Function	orf19.1122	4724	IPF4722	No significant S.c. match	
CA3254	1.0	1.0	1.0	IPF4728	11082297..	unknown Function	orf19.1122	4728	IPF4724	No significant S.c. match	
CA3255	1.1	1.0	1.1	IPF4729	11083186..	unknown Function	orf19.3743	4729	IPF4728	CELL CYCLE AND DNA PROCESSING SUBCELLULAR LOCALISATION	
CA3256	1.0	1.0	1.0	IPF4730	11084640..	unknown Function	orf19.1122	4730	IPF4729	No significant S.c. match	
CA3257	0.9	1.0	0.9	IFC1	11089056..	Unknown Function	orf19.3746	4732	IPF4730	UNCLASSI	molecular_function unknown
CA3258	1.0	1.0	0.9	IFC2	11095342..	Unknown Function	orf19.1123	4737	CaIFC1	TRANSPORT FACILITATION	
CA3259	0.9	1.0	1.1	IPF7970	11100373..	unknown function	orf19.1020	7970	CaIFC2	TRANSPORT FACILITATION	
CA3260	0.9	1.0	1.0	IPF7968	11101858..	unknown function	orf19.2693	7968	IPF7970	No significant S.c. match	
CA3261	1.0	1.1	0.9	TYS1	complemer	tyrosyl-tRNA synthetas	orf19.2694	5023	IPF7968	Nitrogen and sulphur metabolism	
CA3262	1.0	0.8	1.0	UBR11.3	11104759..	ubiquitin-protein ligase	orf19.2695	5026	CaTYS1	PROTEIN S	ligase activity
CA3263	1.1	1.0	1.0	UBR12	complemer	ubiquitin-protein ligase	orf19.2697	19781	CaUBR11.3	PROTEIN I	ligase activity
CA3264	0.9	0.9	1.0	IPF5035	11116385..	unknown function	orf19.2698	5035	CaUBR12	UNCLASSI	ligase activity
CA3265	1.1	1.6	1.1	ABP1	complemer	actin-binding protein (b	orf19.2699	5041	IPF5035	UNCLASSI	molecular_function unknown
CA3266	1.2	1.0	1.0	IPF5045	complemer	unknown function	orf19.2703	5045	CaABP1	CELL FATE	protein binding
CA3267	1.1	1.5	1.0	RPS620A	complemer	unknown function	orf19.6300	20129	IPF5045	UNCLASSI	molecular_function unknown
CA3268	1.1	0.9	1.1	RPS620B	complemer	unknown function	orf19.6301	19981	RPS620a	No significant S.c. match	
CA3269	1.0	0.9	1.1	IPF12221	11134151..	unknown function	orf19.6302	12221	RPS620b	No significant S.c. match	
CA3270	1.1	0.9	1.1	LYS5	complemer	L-aminoadipate-semial	orf19.6304	12222	IPF12221	No significant S.c. match	

CA3271	1.0	1.1	1.1	GLY2	complemen	L-threonine aldolase, l	orf19.6305	12223	CaLYS5	Amino acid	transferase activity
CA3272	1.0	0.9	1.1	ALD4	11138987..	aldehyde dehydrogena	orf19.6306	12224	CaGLY2	Amino acid	lyase activity
CA3273	1.0	1.0	1.0	IPF12227.5	11141508..	unknown function, 5-pr	orf19.6307	19982	CaALD4	CLASSIFICATION NOT YET CLEAR-CUT	
CA3274	1.0	1.0	1.1	IPF12227.3	11142865..	unknown function, 3-pr	orf19.6308	12227	IPF12227.5	TRANSCRIPTION SUBCELLULAR LOCALISATION	
CA3275	1.0	1.0	1.1	IPF17068	11144794..	unknown function	orf19.6309	17068	IPF12227.3	No significant S.c. match	
CA3276	1.0	1.0	1.1	IPF6263	11148141..	unknown function	orf19.6310	6263	IPF17068	No significant S.c. match	
CA3277	0.7	0.5	0.6	IPF6266	complemen	unknown function	orf19.6311	6266	IPF6263	No significant S.c. match	
CA3278	1.1	1.4	1.2	RPS3	complemen	Ribosomal protein S3.(	orf19.6312	6267	IPF6266	No significant S.c. match	
CA3279	1.1	1.0	1.0	IPF6268	complemen	unknown function	orf19.6313	6268	CaRPS3	PROTEIN S	structural molecule activity
CA3280	0.9	0.9	0.8	IPF6269.3	complemen	unknown function, 3-prime end		6269	IPF6268	C-compound and carbohydrate metabolism ""PROTEIN FATE [folding modification destination] ""	
CA3281	1.0	1.0	0.9	RPB8	complemen	DNA-directed RNA pol	orf19.6314	6271	IPF6269.3	No significant S.c. match	
CA3282	1.2	1.1	1.2	IPF6272	11159454..	unknown function	orf19.6315	6272	CaRPB8	TRANSCR	nucleotidyltransferase activity
CA3283	1.1	0.9	1.1	IPF6274	11160497..	unknown function	orf19.6316	6274	IPF6272	No significant S.c. match	
CA3284	1.0	1.1	1.1	ADE6	11163386..	5 -phosphoribosylform	orf19.6317	6279	IPF6274	UNCLASSI	molecular_function unknown
CA3285	1.0	1.0	1.1	IPF6280	complemen	unknown function	orf19.6318	6280	CaADE6	Nucleotide	ligase activity
CA3286	0.9	1.0	1.0	UBP3.3EO	11169385..	Ubiquitin-specific prote	orf19.6319	14298	IPF6280	UNCLASSI	molecular_function unknown
CA3287	1.0	1.0	1.0	IPF14295	complemen	putative Sed1p-like cel	orf19.6321	14295	CaUBP3.3e	TRANSCR	peptidase activity
CA3288	1.1	1.3	0.9	ARD8	complemen	D-arabinitol dehydroge	orf19.6322	14294	IPF14295	No significant S.c. match	
CA3289	0.9	0.9	1.0	HPA3	11173021..	histone and other prot	orf19.6323	14291	CaARD8	ENERGY SUBCELLULAR LOCALISATION	
CA3290	1.1	1.0	1.1	IPF5734	11173579..	unknown function	orf19.6324	5734	CaHPA3	CELL CYC	transferase activity
CA3291	1.0	0.9	0.9	IPF5730	complemen	unknown function		5730	IPF5734	UNCLASSI	molecular_function unknown
CA3292	1.0	0.9	1.0	IPF5729	11176175..	unknown function	orf19.6326	5729	IPF5730	UNCLASSI	molecular_function unknown
CA3293	1.1	1.1	1.0	IPF5726	11177065..	unknown function	orf19.6327	5726	IPF5729	No significant S.c. match	
CA3294	1.0	0.9	1.0	IPF5725	complemen	unknown function	orf19.6328	5725	IPF5726	No significant S.c. match	
CA3295	1.0	1.0	1.1	IPF5723.E	11178263..	cell surface GPI-ancho	orf19.6329	5723	IPF5725	UNCLASSI	molecular_function unknown
CA3296	1.0	1.0	1.0	IPF5723.E	11179639..	cell surface GPI-ancho	orf19.6336	5720	IPF5723.ex	No significant S.c. match	
CA3299	1.0	1.0	1.1	IPF13777	complemen	unknown function	orf19.2317	13777	IPF5723.ex	No significant S.c. match	
CA3300	1.0	0.9	1.0	RTG3	complemen	Probable bHLH/zip tra	orf19.2315	13780	IPF13777	No significant S.c. match	
CA3301	0.9	0.9	0.9	IPF13782	complemen	unknown function	orf19.2314	13782	CaRTG3	C-compour	transcription regulator activity
CA3302	0.9	1.0	1.0	IPF13784	complemen	unknown function	orf19.2313	13784	IPF13782	CONTROL	molecular_function unknown
CA3303	1.0	1.2	1.0	FRE42	11198079..	ferric reductase (by ho	orf19.2312	15397	IPF13784	UNCLASSIFIED PROTEINS	
CA3304	1.6	1.2	1.4	RPL82	11200750..	60S ribosomal protein	orf19.2311	11736	CaFRE42	REGULATION OF/INTERACTION WITH CELLULAR ENVIRONMENT Other virulence attributes	
CA3305	0.9	1.2	1.3	RPL29	11202094..	ribosomal protein, cytosolic(by hor		11737	CaRPL82	PROTEIN SYNTHESIS SUBCELLULAR LOCALISATION	
CA3306	0.9	1.0	1.0	IPF11738	11202754..	unknown function	orf19.2310	11738	CaRPL29	No significa	structural molecule activity
CA3307	0.9	1.1	1.6	RPL2.3	complemen	ribosomal protein L8, 3-prime end		11739	IPF11738	PROTEIN S	molecular_function unknown
CA3308	0.8	0.8	0.9	PET127	11206167..	component of mitochor	orf19.9845	11742	CaRPL2.3	PROTEIN S	structural molecule activity
CA3309	1.0	1.1	1.0	IPF8806	11210004..	6-phosphofructose-2-k	orf19.9844	8806	CaPET127	PROTEIN S	molecular_function unknown
CA3310	1.0	0.9	1.0	PFS2	complemen	Polyadenylation Facto	orf19.2307	8808	IPF8806	C-compound and carbohydrate metabolism SUBCELLULAR LOCALISATION	
CA3311	1.0	1.0	1.0	IPF8809	11213581..	unknown function	orf19.2306	8809	CaPFS2	TRANSCR	RNA binding
CA3312	1.1	0.9	1.0	IPF8810	complemen	unknown function	orf19.2305	8810	IPF8809	UNCLASSI	molecular_function unknown
CA3313	1.1	1.1	1.1	IPF8811	complemen	unknown function	orf19.2304	8811	IPF8810	PROTEIN I	protein binding
CA3314	1.0	0.8	0.9	IPF8812	11216686..	unknown function	orf19.2303	8812	IPF8811	UNCLASSIFIED PROTEINS	
CA3315	0.9	0.9	1.0	IPF8814	11217693..	unknown function	orf19.2302	8814	IPF8812	TRANSCR	RNA binding
CA3316	1.1	1.1	1.0	IPF8817	11219488..	putative proteasome su	orf19.2301	8817	IPF8814	No significant S.c. match	
CA3317	1.1	1.0	1.1	URM1	complemen	ubiquitin like protein (b	orf19.2299	8818	IPF8817	UNCLASSI	molecular_function unknown
CA3318	1.1	1.1	1.1	WBP1	11221040..	Oligosaccharyl transfer	orf19.2298	8820	CaURM1	PROTEIN FATE [folding modification destination]	
CA3319	1.0	1.0	1.0	DRS25.3E	11223914..	Probable ATPase, 3-pr	orf19.2680	6900	CaWBP1	C-compour	transferase activity
CA3320	0.9	1.0	1.0	IPF6898	complemen	similar to Saccharomy	orf19.2678	6898	CaDRS25.3	TRANSPO	transporter activity
CA3321	1.0	1.1	1.0	IPF6896.5F	11231331..	unknown function, 5-pr	orf19.2677	6896	IPF6898	CELL CYC	protein binding,protein kinase activity
CA3322	1.0	1.0	1.0	IPF6895.3F	11232939..	unknown function, 3-prime end		6895	IPF6896.5f	ENERGY	hydrolase activity
CA3323	0.8	0.9	0.9	IKI1	complemen	killer toxin insensitive p	orf19.2676	6894	IPF6895.3f	ENERGY	
CA3324	1.0	0.9	1.1	IPF6893	11234357..	unknown function	orf19.2675	6893	CaIKI1	CLASSIFIC	transcription regulator activity
CA3325	0.9	1.0	1.1	IPF6890	complemen	unknown function	orf19.2674	6890	IPF6893	TRANSCR	RNA binding
CA3326	0.9	1.0	1.0	IPF6889	11239385..	unknown function	orf19.2673	6889	IPF6890	No significant S.c. match	
CA3327	0.9	0.9	1.2	NCP1	11242005..	NADPH-cytochrome P.	orf19.2672	10561	IPF6889	UNCLASSI	molecular_function unknown
CA3328	1.0	1.1	1.0	IPF10564	11245061..	unknown function	orf19.2671	10564	CaNCP1	Lipid fatty-a	transporter activity
CA3329	0.9	0.9	1.0	IPF12162	complemen	Unknown function	orf19.2670	12162	IPF10564	UNCLASSIFIED PROTEINS	
CA3330	1.0	0.9	1.0	POL21.3	complemen	pol polyprotein, revers	orf19.2669	12161	IPF12162	UNCLASSI	molecular_function unknown
CA3331	0.9	1.0	1.0	POL.3	complemen	Pol part of pCal retrotransposon		12159	CaPOL21.3	No significant S.c. match	
CA3332	1.0	1.0	1.0	GAG	complemen	GAG protein of pCal re	orf19.2668	12158	CaPOL.3	No significant S.c. match	
CA3333	1.0	1.3	0.9	CDC48	11256925..	microsomal ATPase (b	orf19.9876	8680	CaGAG	No significant S.c. match	
CA3334	1.3	1.1	1.5	ALP1	complemen	amino-acid permease	orf19.2337	8681	CaCDC48	CELL CYC	hydrolase activity
CA3335	1.0	0.9	1.0	IPF8682	11262451..	unknown function	orf19.2336	8682	CaALP1	Amino acid metabolism	CELLULAR TRANSPORT AND TRANSPORT MECHANISMS SUBCELLULAR LOCALISATION TRANSPORT FA
CA3336	0.8	0.7	0.9	IPF19983	complemen	unknown function	orf19.2335	19983	IPF8682	CELL FATE	
CA3337	0.9	0.9	0.9	IPF13583	11264759..	unknown function	orf19.2334	13583	IPF19983	PROTEIN I	molecular_function unknown
CA3338	1.3	1.2	1.1	IPF13582	complemen	unknown function	orf19.2333	13582	IPF13583	C-compour	molecular_function unknown
CA3339	0.9	1.0	1.0	IPF20131	11269757..	unknown function	orf19.2332	20131	IPF13582	UNCLASSI	molecular_function unknown
CA3340	1.0	0.9	0.9	ADA2	complemen	general transcriptional	orf19.2331	15288	IPF20131	CONTROL	molecular_function unknown
CA3341	1.1	1.4	1.1	RPS17.3	11273133..	Ribosomal protein S17, 3-prime er		15290	CaADA2	TRANSCR	transcription regulator activity
CA3342	1.0	1.0	1.0	IPF13361	11274016..	unknown function	orf19.2330	13361	CaRPS17.3	PROTEIN S	structural molecule activity
CA3343	1.0	1.1	1.0	IPF13360	complemen	unknown function	orf19.2328	13360	IPF13361	UNCLASSI	RNA binding
CA3344	0.9	0.9	0.9	IPF13359	complemen	unknown function	orf19.2326	13359	IPF13360	No significant S.c. match	

CA3345	1.0	1.1	1.0	IPF13357	11278824..	unknown function	orf19.2325	13357	IPF13359	CELL CYC	molecular_function unknown
CA3346	0.9	0.9	1.0	IPF13356	complemer	molybdopterin biosynth	orf19.2324	13356	IPF13357	TRANSCR	transcription regulator activity
CA3347	1.2	1.2	1.1	ERP5	complemer	protein involved in membrane traff		15563	IPF13356	Metabolism of vitamins cofactors and prosthetic groups	
CA3348	0.9	0.9	0.9	RIO1	complemer	unknown function	orf19.2320	15559	CaERP5	PROTEIN I	molecular_function unknown
CA3349	1.0	1.0	1.0	IPF19588	11284820..	unknown function		19588	CaRIO1	UNCLASSI	protein kinase activity
CA3350	1.0	0.9	0.9	RPL18A.5	complemer	ribosomal protein S18.e, 5-prime e		18348	IPF19588	UNCLASSI	molecular_function unknown
CA3351	1.0	1.0	0.9	IPF18347	11287619..	unknown function	orf19.9503	18347	CaRPL18A	PROTEIN SYNTHESIS SUBCELLULAR LOCALISATION	
CA3352	0.9	0.9	1.0	IPF16194	complemer	unknown function	orf19.9501	16195	IPF18347	No significant S.c. match	
CA3353	1.1	1.0	1.1	AUR1	11290569..	aureobasidin-resistanc	orf19.9500	11286	IPF16194	CLASSIFIC	molecular_function unknown
CA3354	0.9	1.0	0.9	IPF11281	11294397..	similar to Saccharomy	orf19.9499	11281	CaAUR1	Lipid fatty-a	transferase activity
CA3355	0.9	1.0	0.9	IPF11277	11297435..	unknown function	orf19.9498	11277	IPF11281	CELLULAF	signal transducer activity
CA3356	1.1	1.0	1.0	IPF9560	11300320..	unknown function	orf19.9497	9560	IPF11277	Nucleotide metabolism CELL CYCLE AND DNA PROCESSING CELLULAR COMMUNICATION/SIGNAL TRANSDUCTION MECHANISM	
CA3357	0.9	1.0	1.0	IPF9562	complemer	similar to Saccharomy	orf19.1941	9562	IPF9560	CELL RESCUE DEFENSE AND VIRULENCE ""TRANSPORT FACILITATION	
CA3358	1.1	1.2	1.0	IPF19984	complemer	similar to Saccharomy	orf19.1940	19984	IPF9562	CELL CYC	structural molecule activity
CA3359	0.8	0.8	0.8	IPF9566	complemer	unknown function	orf19.1939	9566	IPF19984	C-compour	molecular_function unknown
CA3360	0.9	0.9	0.9	IPF9568	11307329..	unknown function	orf19.9493	9568	IPF9566	UNCLASSI	molecular_function unknown
CA3361	1.0	1.0	0.9	SNF1	11308238..	serine/threonine protei	orf19.9491	9571	IPF9568	No significant S.c. match	
CA3362	1.0	1.1	1.1	IPF9789	11313145..	unknown function	orf19.9489	9789	CaSNF1	C-compour	protein kinase activity
CA3363	1.1	1.1	1.0	FRE32	11316949..	ferric reductase (by ho	orf19.9488	9791	IPF9789	UNCLASSI	molecular_function unknown
CA3365	0.9	0.8	1.1	ALG6	11322070..	glucosyltransferase (by	orf19.1843	4661	CaFRE32	REGULATI	oxidoreductase activity
CA3366	0.9	0.9	0.9	FRE43	11332963..	ferric reductase-like (b	orf19.9403	4664	CaALG6	C-compour	transferase activity
CA3367	1.0	1.0	1.0	IPF4667	11336168..	unknown Function	orf19.9405	4667	CaFRE43	REGULATION OF/INTERACTION WITH CELLULAR ENVIRONMENT Other virulence attributes	
CA3368	0.9	0.9	0.9	IPF4671	complemer	unknown Function	orf19.1849	4671	IPF4667	C-compour	lyase activity
CA3369	1.0	1.0	1.0	IPF4672	11341348..	unknown Function	orf19.1850	4672	IPF4671	UNCLASSI	molecular_function unknown
CA3370	1.0	0.9	1.0	IPF4674	complemer	unknown Function	orf19.1852	4674	IPF4672	UNCLASSI	protein phosphatase activity
CA3372	3.2	2.9	2.7	HHF22	complemer	histone H4 (by homolo	orf19.1854	4677	IPF4674	UNCLASSI	molecular_function unknown
CA3373	0.9	1.0	1.0	SEO1	11346465..	suppressor of sulfoxyd	orf19.1855	19985	CaHHF22	TRANSCRIPTION SUBCELLULAR LOCALISATION	
CA3374	0.9	0.9	1.2	IPF4683	complemer	unknown Function	orf19.1856	4683	CaSEO1	TRANSPORT FACILITATION	
CA3375	0.9	1.0	1.1	IPF4684	11351101..	unknown Function	orf19.1857	4684	IPF4683	TRANSCR	protein binding
CA3376	1.4	1.3	1.1	LSC2.3EO	11353073..	succinate-CoA ligase beta subunit		4686	IPF4684	TRANSCRIPTION SUBCELLULAR LOCALISATION	
CA3377	1.0	1.0	1.0	CLF1	11357226..	pre-mRNA splicing fac	orf19.7964	8347	CaLSC2.3e	C-compound and carbohydrate metabolism ENERGY	
CA3378	1.0	1.0	1.0	RUB1	complemer	ubiquitin-like protein (by homology		8348	CaCLF1	CELL CYC	molecular_function unknown
CA3379	1.0	0.9	1.0	IPF8350	11360004..	putative methyltransfer	orf19.331	8350	CaRUB1	PROTEIN SYNTHESIS ""PROTEIN FATE [folding modification destination] """"CELL RESCUE DEFENSE AND VIRULENCE ""CELL FATE	
CA3380	1.0	1.0	0.9	MSL5	11360855..	branch point bridging p	orf19.329	8351	IPF8350	PROTEIN I	transferase activity
CA3381	0.9	0.9	0.9	NPR2	11362697..	nitrogen permease reg	orf19.328	8357	CaMSL5	TRANSCR	RNA binding
CA3382	1.1	1.2	1.2	HTA3	11364932..	histone H2A F/Z variar	orf19.327	8358	CaNPR2	Nitrogen ar	molecular_function unknown
CA3383	1.1	1.0	1.0	IPF8359	complemer	unknown function	orf19.326	8359	CaHTA3	SUBCELLU	DNA binding
CA3384	1.0	0.9	0.9	IPF8362	complemer	similar to Saccharomy	orf19.325	8362	IPF8359	No significant S.c. match	
CA3385	1.1	1.0	1.1	DRS23	complemer	Membrane-spanning C	orf19.7955	6764	IPF8362	TRANSCR	RNA binding
CA3386	1.0	1.0	1.0	IPF6758	11374192..	unknown function	orf19.7954	6758	CaDRS23	TRANSPORT FACILITATION	
CA3387	1.1	1.1	1.1	MUP3	11376288..	Very low affinity methic	orf19.7953	6757	IPF6758	No significant S.c. match	
CA3388	1.4	1.2	1.2	IPF6755	complemer	unknown function	orf19.320	6755	CaMUP3	Amino acid	transporter activity
CA3389	1.1	1.0	1.0	IPF6754	11380356..	unknown function	orf19.7951	6754	IPF6755	CLASSIFICATION NOT YET CLEAR-CUT	
CA3390	0.9	0.9	1.0	CAF17	11381000..	Component of CCR4 transcriptiona		6752	IPF6754	UNCLASSI	molecular_function unknown
CA3391	1.0	1.1	0.9	PNP1	11382811..	Purine Nucleoside Phc	orf19.7949	6750	CaCAF17	TRANSCR	molecular_function unknown
CA3392	1.1	1.2	1.1	SEC13	complemer	Protein transport prote	orf19.316	6749	CaPNP1	Nucleotide	transferase activity
CA3393	1.0	0.9	1.0	IPF6748	11385023..	unknown function	orf19.315	6748	CaSEC13	CELLULAF	molecular_function unknown
CA3394	1.0	0.9	1.0	IPF6747	complemer	similar to Saccharomy	orf19.7946	6747	IPF6748	CELL CYC	molecular_function unknown
CA3395	1.0	1.1	1.0	IPF13616	complemer	unknown function	orf19.3970	13616	IPF6747	TRANSCR	transcription regulator activity
CA3396	1.0	0.9	1.0	IPF13617	11394387..	unknown function	orf19.3971	13617	IPF13616	UNCLASSI	molecular_function unknown
CA3397	1.0	1.0	1.1	IPF13618	complemer	unknown function	orf19.3972	13618	IPF13617	No significant S.c. match	
CA3398	1.1	1.0	1.0	IPF13621	11396437..	unknown function	orf19.3973	13621	IPF13618	PROTEIN I	molecular_function unknown
CA3399	1.0	0.9	1.0	PUT2	complemer	1-pyrroline-5-carboxyla	orf19.3974	13622	IPF13621	UNCLASSI	molecular_function unknown
CA3400	1.0	1.0	0.9	JNM1	complemer	mitosis protein, involve	orf19.3976	13623	CaPUT2	Amino acid	oxidoreductase activity
CA3401	1.0	0.9	1.1	IPF8652	complemer	putative GTP-binding p	orf19.3977	8652	CaJNM1	CELL CYC	structural molecule activity
CA3402	1.0	0.9	1.0	IPF8651	11402277..	unknown function	orf19.3978	8651	IPF8652	UNCLASSI	molecular_function unknown
CA3403	1.0	0.9	1.0	IPF8650	complemer	RNA helicase (by hom	orf19.3980	8650	IPF8651	UNCLASSI	molecular_function unknown
CA3404	1.0	1.0	0.9	MAL31	complemer	maltose permease (by	orf19.3981	8647	IPF8650	CELL RES	molecular_function unknown
CA3405	1.0	0.9	0.9	IPF8644	11414245..	maltase (by homology)	orf19.3982	8644	CaMAL31	C-compour	molecular_function unknown
CA3406	1.0	1.0	1.0	IPF9711	complemer	unknown function	orf19.3983	9711	IPF8644	C-compour	hydrolase activity
CA3407	0.8	0.6	0.9	IPF9406	11426364..	unknown function	orf19.1357	9406	IPF9711	UNCLASSI	molecular_function unknown
CA3408	1.2	1.1	1.1	IPF9407	11428214..	similar to Saccharomy	orf19.6151	9407	IPF9406	No significant S.c. match	
CA3409	1.0	0.8	1.0	IPF9410	complemer	similar to Saccharomy	orf19.6148	9410	IPF9407	CELLULAF	structural molecule activity
CA3410	1.2	1.2	1.0	IPF9411	11433417..	unknown function	orf19.6147	9411	IPF9410	CELL CYC	structural molecule activity
CA3411	1.3	2.0	1.2	IPF9413	complemer	unknown function	orf19.1356	9413	IPF9411	UNCLASSI	molecular_function unknown
CA3412	1.0	1.0	1.0	IPF9238	complemer	long chain fatty alcoho	orf19.1356	9238	IPF9413	CELL CYC	protein kinase activity,enzyme regulator activity
CA3413	0.9	1.0	1.1	IPF9239	complemer	unknown function	orf19.6142	9239	IPF9238	No significant S.c. match	
CA3414	1.0	0.9	1.0	IPF9240	complemer	probable permease	orf19.6141	9240	IPF9239	No significant S.c. match	
CA3415	1.2	1.1	1.0	FRE30.53	11443642..	Strong similarity to ferr	orf19.6140	17765	IPF9240	C-compound and carbohydrate metabolism CELLULAR TRANSPORT AND TRANSPORT MECHANISMS SUBCELLULAR LOCALISATIO	
CA3416	1.3	1.3	1.1	FRE30.3	11444320..	Strong similarity to ferr	orf19.6139	17763	CaFRE30.5	REGULATION OF/INTERACTION WITH CELLULAR ENVIRONMENT Other virulence attributes	
CA3417	1.0	1.0	0.9	FRE41	11446783..	ferric reductase transm	orf19.6138	12773	CaFRE30.3	REGULATION OF/INTERACTION WITH CELLULAR ENVIRONMENT Other virulence attributes	
CA3418	1.0	0.9	1.0	IPF12777	11449360..	unknown function	orf19.6137	12777	CaFRE41	REGULATION OF/INTERACTION WITH CELLULAR ENVIRONMENT Other virulence attributes	

CA3419	0.9	1.0	1.0	IPF12778	complemer	ribosomal protein, mitc	orf19.6136	12778	IPF12777	TRANSCRIPTION SUBCELLULAR LOCALISATION	
CA3420	1.1	1.1	1.1	SMX4	11452544..	U6 snRNA-associated	Sm-like pro	12779	IPF12778	PROTEIN !	structural molecule activity
CA3421	1.0	1.0	1.0	IPF19782	complemer	unknown function	orf19.1355	19782	CaSMX4	TRANSCR	RNA binding
CA3422	0.9	1.0	0.9	IPF17289	11454560..	unknown function	orf19.1355	17289	IPF19782	CELL CYCLE AND DNA PROCESSING CONTROL OF CELLULAR ORGANIZATION SUBCELLULAR LOCALISATION	
CA3423	1.0	1.0	1.0	IPF3549	complemer	unknown function	orf19.597	3549	IPF17289	UNCLASSI	molecular_function unknown
CA3424	0.9	0.8	1.0	NOP10	complemer	Nucleolar rRNA processing protein		3544	IPF3549	Nitrogen and sulphur metabolism	
CA3425	1.0	1.0	0.9	IPF11873	complemer	similar to Saccharomy	orf19.593	11873	CaNOP10	No significa	RNA binding
CA3426	0.9	0.8	0.9	IPF11869	11469541..	unknown function	orf19.592	11869	IPF11873	CELLULAF	molecular_function unknown
CA3427	0.9	0.9	1.2	IPF11865	complemer	unknown function	orf19.590	11865	IPF11869	UNCLASSI	transferase activity
CA3428	1.1	1.0	1.2	VPS21	complemer	GTP-binding protein (b	orf19.589	11863	IPF11865	No significant S.c. match	
CA3429	0.9	0.9	1.0	IPF6380	11479667..	unknown function	orf19.588	6380	CaVPS21	PROTEIN FATE [folding modification destination] ""CELLULAR TRANSPORT AND TRANSPORT MECHANISMS SUBCELLULAR LOCAL	
CA3430	1.0	0.9	0.9	IPF6382	complemer	unknown function	orf19.587	6382	IPF6380	UNCLASSI	molecular_function unknown
CA3431	0.9	1.1	1.0	FUN9	11481359..	Protein involved in ves	orf19.586	6383	IPF6382	UNCLASSI	molecular_function unknown
CA3432	1.0	1.0	1.0	MRPL17	complemer	ribosomal protein of th	orf19.585	20133	CaFUN9	CELLULAF	molecular_function unknown
CA3433	1.0	0.9	1.0	IPF6387.3	11484272..	unknown function, 3-prime end		6387	CaMRPL17	PROTEIN !	structural molecule activity
CA3434	1.1	0.9	0.9	CCP1	11486211..	Cytochrome-c peroxida	orf19.584	6390	IPF6387.3	No significant S.c. match	
CA3435	1.0	1.2	1.1	IPF6391	11487140..	similarity to mammalia	orf19.583	6391	CaCCP1	CELL RES	oxidoreductase activity
CA3436	1.0	0.9	1.0	NRD1	11489739..	Involved in regulation c	orf19.581	6394	IPF6391	Amino acid	oxidoreductase activity
CA3437	1.1	1.2	1.2	IPF15925	complemer	unknown function	orf19.1050	15925	CaNRD1	TRANSCR	RNA binding
CA3438	0.6	0.3	1.0	IPF11330	11495433..	unknown function	orf19.2987	11330	IPF15925	REGULATION OF/INTERACTION WITH CELLULAR ENVIRONMENT SUBCELLULAR LOCALISATION	
CA3439	1.1	1.0	1.0	IPF11329	complemer	unknown function	orf19.2986	11329	IPF11330	UNCLASSI	molecular_function unknown
CA3440	0.9	0.8	1.2	IPF11328	11497940..	unknown function	orf19.2985	11328	IPF11329	No significant S.c. match	
CA3441	0.9	0.9	1.0	MST1	11498686..	mitochondrial threonyl	orf19.2984	11327	IPF11328	No significant S.c. match	
CA3442	0.8	0.6	0.9	IPF11326	complemer	similar to Saccharomy	orf19.2983	11326	CaMST1	SUBCELLU	ligase activity
CA3443	1.1	1.1	0.9	IPF11324	11501524..	unknown function	orf19.2982	11324	IPF11326	TRANSCR	transcription regulator activity
CA3444	0.9	0.7	1.0	IPF11323	11503175..	unknown function	orf19.2980	11323	IPF11324	PROTEIN I	enzyme regulator activity
CA3445	0.9	1.0	1.0	IPF7325	11505637..	unknown function	orf19.2978	7325	IPF11323	UNCLASSI	molecular_function unknown
CA3446	1.0	0.9	1.1	IPF7324	11506679..	unknown function	orf19.2977	7324	IPF7325	No significant S.c. match	
CA3447	1.0	1.0	1.1	YPT70	complemer	GTP-binding protein (b	orf19.2975	7322	IPF7324	UNCLASSI	molecular_function unknown
CA3448	0.9	0.9	0.9	YKT6	11509430..	Endoplasmic Reticulum	orf19.2974	7321	CaYPT70	CELLULAR TRANSPORT AND TRANSPORT MECHANISMS SUBCELLULAR LOCALISATION	
CA3449	1.0	1.0	1.0	IPF7320	complemer	unknown function	orf19.2973	7320	CaYKT6	PROTEIN I	transporter activity
CA3450	1.0	0.9	0.9	PDE2	complemer	Nucleotide phosphodie	orf19.2972	7318	IPF7320	UNCLASSI	transporter activity
CA3451	1.0	1.0	1.0	IPF7316	complemer	unknown function	orf19.1048	7316	CaPDE2	Nucleotide	hydrolase activity
CA3452	0.8	0.8	0.7	IPF10541	11523174..	unknown function	orf19.1253	10541	IPF7316	CELL CYCLE AND DNA PROCESSING CONTROL OF CELLULAR ORGANIZATION SUBCELLULAR LOCALISATION	
CA3453	1.0	1.1	1.1	SEC23	11525673..	Component of COPII c	orf19.1254	10536	IPF10541	Phosphate	DNA binding,transcription regulator activity
CA3454	0.9	0.9	0.9	IPF10533.E	11529937..	unknown function, exo	orf19.1255	10533	CaSEC23	CELLULAF	enzyme regulator activity
CA3455	1.0	0.9	0.9	IPF10533.E	11532466..	unknown function, exo	orf19.1256	19641	IPF10533.e	C-compound and carbohydrate metabolism TRANSCRIPTION SUBCELLULAR LOCALISATION	
CA3456	1.0	0.9	0.9	IPF19986	complemer	unknown function	orf19.1259	19986	IPF10533.e	No significant S.c. match	
CA3457	0.9	0.9	1.0	IPF8521	complemer	unknown function	orf19.1260	8521	IPF19986	SUBCELLU	molecular_function unknown
CA3459	0.9	1.0	1.0	CGT1.3F	11544397..	mRNA capping enzym	orf19.1261	8518	IPF8521	TRANSCR	RNA binding
CA3460	0.9	0.9	0.9	CFL1	complemer	ferric reductase	orf19.1263	8516	CaCGT1.3	TRANSCR	nucleotidyltransferase activity
CA3461	1.1	1.1	1.0	CFL2	complemer	ferric reductase (by ho	orf19.1264	8512	CaCFL1	REGULATION OF/INTERACTION WITH CELLULAR ENVIRONMENT SUBCELLULAR LOCALISATION Other virulence attributes	
CA3462	1.1	1.0	1.1	IPF14959	11556303..	similar to Saccharomy	orf19.1265	14959	CaCFL2	REGULATION OF/INTERACTION WITH CELLULAR ENVIRONMENT Other virulence attributes	
CA3463	1.1	0.9	0.9	ALG8	complemer	glucosyltransferase (by	orf19.1659	12530	IPF14959	CELLULAF	molecular_function unknown
CA3464	0.9	1.1	1.0	DBP5	11565513..	RNA helicase (by hom	orf19.1661	12528	CaALG8	C-compour	transferase activity
CA3465	1.0	1.1	0.9	MRP1.3F	complemer	Mitochondrial ribosomal protein of		18330	CaDBP5	CLASSIFIC	RNA binding,helicase activity
CA3466	0.9	0.8	1.0	MRP1.5F	complemer	mitochondrial ribosoma	orf19.1662	12526	CaMRP1.3	PROTEIN !	structural molecule activity
CA3467	1.2	1.0	1.0	MNT2	11570759..	Alpha-1,2-mannosyltra	orf19.1663	6319	CaMRP1.5	PROTEIN SYNTHESIS SUBCELLULAR LOCALISATION	
CA3468	1.0	1.0	1.0	IPF6318	complemer	beta-glucosidase (by h	orf19.1664	6318	CaMNT2	C-compour	transferase activity
CA3469	1.2	1.1	1.1	MNT1	11577042..	Mannosyltransferase ir	orf19.1665	6315	IPF6318	No significant S.c. match	
CA3470	1.1	1.0	1.1	IPF6310	11580716..	unknown function	orf19.1666	6310	CaMNT1	C-compour	transferase activity
CA3471	0.5	0.2	0.9	IPF6308	11582920..	unknown function	orf19.1667	6308	IPF6310	No significant S.c. match	
CA3472	1.0	0.9	1.1	IPF6305.E	11586885..	unknown function, exon 2		6306	IPF6308	UNCLASSIFIED PROTEINS	
CA3473	1.0	0.9	1.0	IPF6305.E	11587374..	unknown function, exo	orf19.1668	6305	IPF6305.e	UNCLASSI	molecular_function unknown
CA3474	1.0	1.1	1.0	AFG3	complemer	Member of the Sec18p	orf19.1669	8090	IPF6305.e	UNCLASSI	molecular_function unknown
CA3475	1.0	0.9	1.0	BRO1	11592591..	required for normal res	orf19.1670	8087	CaAFG3	PROTEIN I	peptidase activity
CA3476	0.9	0.7	1.0	NUP2.3EO	11599995..	nucleoporine involved	orf19.3583	3232	CaBRO1	CELL RES	transporter activity
CA3477	1.0	1.0	1.0	IPF3229	complemer	unknown function	orf19.3582	3229	CaNUP2.3	CELLULAF	structural molecule activity
CA3478	1.0	1.0	1.0	IPF3227	11602346..	similar to Saccharomy	orf19.3581	3227	IPF3229	UNCLASSI	transferase activity
CA3479	1.1	0.8	1.0	ATP4	complemer	F1F0-ATPase comple	orf19.3579	3225	IPF3227	CELL CYC	molecular_function unknown
CA3480	1.0	1.1	1.1	IPF3224	complemer	unknown function	orf19.3578	3224	CaATP4	ENERGY C	structural molecule activity
CA3481	1.1	1.0	0.9	IPF3223	11605324..	unknown function		3223	IPF3224	No significant S.c. match	
CA3482	1.0	1.0	1.0	COQ5	complemer	C-methyltransferase (b	orf19.3577	3222	IPF3223	No significant S.c. match	
CA3483	4.2	4.1	2.0	CDC19	complemer	pyruvate kinase (by ho	orf19.3575	19988	CaCOQ5	Metabolism	transferase activity
CA3484	1.0	1.0	1.0	IPF3214	11610094..	HSP-mitochondrial cha	orf19.3574	3214	CaCDC19	C-compour	transferase activity
CA3485	1.0	1.0	0.9	IPF3213	complemer	similar to Saccharomy	orf19.3573	3213	IPF3214	PROTEIN I	molecular_function unknown
CA3486	1.0	1.0	1.0	IPF3206	complemer	unknown function	orf19.3572	3206	IPF3213	PROTEIN I	hydrolase activity
CA3487	1.1	1.1	1.1	IPF3203	complemer	unknown function	orf19.3569	3203	IPF3206	No significant S.c. match	
CA3488	1.0	0.9	1.0	IPF3198	11623067..	unknown function	orf19.3568	3198	IPF3203	UNCLASSI	molecular_function unknown
CA3489	1.0	1.1	1.1	IPF3195	11625182..	unknown function	orf19.3567	3195	IPF3198	UNCLASSI	molecular_function unknown
CA3490	0.9	0.9	0.9	IPF3192	11626857..	unknown function	orf19.3565	3192	IPF3195	Metabolism of vitamins cofactors and prosthetic groups	
CA3491	0.9	1.0	1.0	RPC40	11628235..	RNA polymerase (by h	orf19.3564	3191	IPF3192	UNCLASSIFIED PROTEINS	

CA3492	0.9	1.0	0.9	IPF3189	11630027..	unknown function	orf19.3563	3189	CaRPC40	TRANSCR nucleotidyltransferase activity
CA3493	1.0	1.0	0.9	CDC7	complemer	serine/threonine protei	orf19.3561	19635	IPF3189	No significant S.c. match
CA3494	1.0	1.1	1.0	RPB5	11634820..	DNA-directed RNA pol	orf19.6340	17067	CaCDC7	CELL CYC protein kinase activity
CA3495	1.3	1.6	1.2	RIB7	complemer	HTP reductase (By hor	orf19.6341	17066	CaRPB5	TRANSCR nucleotidyltransferase activity
CA3496	1.0	1.0	0.9	IPF17064	complemer	unknown function	orf19.6342	17064	CaRIB7	Metabolism hydrolase activity
CA3497	1.0	0.9	1.0	FEN11	11638516..	Fatty acid elongase re	orf19.6343	18329	IPF17064	No significant S.c. match
CA3498	0.9	0.8	0.9	RBK1	complemer	Ribokinase (by homolc	orf19.6344	15253	CaFEN11	C-compour transferase activity
CA3499	1.1	1.1	0.9	RPG1	complemer	Translation initiation fa	orf19.6345	15252	CaRBK1	C-compour transferase activity
CA3500	1.0	1.0	1.0	IPF14487	complemer	unknown function	orf19.6346	14487	CaRPG1	CELL CYC translation regulator activity
CA3501	1.0	1.0	1.0	CDC40	11645515..	cell division control prc	orf19.6347	14486	IPF14487	UNCLASSI molecular_function unknown
CA3502	1.1	1.0	1.0	IPF14485	complemer	unknown function	orf19.6348	14485	CaCDC40	CELL CYC RNA binding
CA3503	0.0	0.0	0.7	IPF15890	complemer	cytoskeletal binding pr	orf19.6349	15890	IPF14485	No significant S.c. match
CA3504	1.0	1.0	1.1	IPF11369	11653630..	unknown function	orf19.6350	11369	IPF15890	CELLULAR TRANSPORT AND TRANSPORT MECHANISMS CELL FATE SUBCELLULAR LOCALISATION
CA3505	0.9	1.0	1.0	IPF11372	11654824..	unknown function	orf19.6351	11372	IPF11369	No significant S.c. match
CA3506	1.0	0.9	0.9	IFA5	complemer	unknown function	orf19.6353	11374	IPF11372	No significant S.c. match
CA3507	0.7	0.3	1.0	IPF11375	11659625..	unknown function	orf19.6354	11375	CaIFA5	Nucleotide metabolism CELL CYCLE AND DNA PROCESSING CELLULAR COMMUNICATION/SIGNAL TRANSDUCTION MECHANISM
CA3508	0.9	0.9	0.9	RSA2	complemer	Involved in ribosome b	orf19.6355	11376	IPF11375	TRANSCRIPTION SUBCELLULAR LOCALISATION
CA3509	0.9	1.0	1.0	PRP6	11662111..	snRNP(U4/U6)-associ	orf19.6356	19783	CaRSA2	TRANSCR molecular_function unknown
CA3510	1.1	1.0	1.0	MAD1	complemer	coiled-coil protein invol	orf19.6357	11251	CaPRP6	TRANSCR RNA binding
CA3511	1.1	1.0	1.0	MMS2	11667629..	ubiquitin-conjugating e	orf19.6358	11248	CaMAD1	CELL CYC molecular_function unknown
CA3512	1.0	1.0	0.9	IPF11247	complemer	unknown function	orf19.6359	11247	CaMMS2	CELL CYCLE AND DNA PROCESSING
CA3513	1.0	1.1	1.0	IPF11246	11668915..	unknown function	orf19.6360	11246	IPF11247	PROTEIN I molecular_function unknown
CA3514	1.1	1.1	1.0	IPF11245	complemer	similar to Saccharomy	orf19.6362	11245	IPF11246	No significant S.c. match
CA3515	1.0	0.9	1.0	MRPL15	complemer	mitochondrial ribosoma	orf19.6363	11656	IPF11245	REGULATI protein binding
CA3517	1.1	1.1	1.0	IFA22	11677479..	Unknown function	orf19.1002	15371	CaMRPL15	PROTEIN S structural molecule activity
CA3518	0.9	1.0	1.0	AMYG2	11686305..	glucoamylase	orf19.8614	6791	CaIFA22	CELL CYCLE AND DNA PROCESSING SUBCELLULAR LOCALISATION
CA3519	1.0	0.9	0.9	IPF6787	11689544..	unknown function	orf19.8613	6787	CaAMYG2	C-compound and carbohydrate metabolism ENERGY SUBCELLULAR LOCALISATION
CA3520	0.9	0.8	0.9	IPF6785	complemer	unknown function	orf19.997	6785	IPF6787	No significant S.c. match
CA3521	1.0	1.0	1.0	IFA15	complemer	unknown function	orf19.996	6784	IPF6785	No significant S.c. match
CA3522	0.9	1.0	0.9	IPF20134	complemer	unknown function	orf19.994	20134	CaIFA15	Nucleotide metabolism CELL CYCLE AND DNA PROCESSING CELLULAR COMMUNICATION/SIGNAL TRANSDUCTION MECHANISM
CA3523	1.1	1.0	1.0	IPF20135	complemer	unknown function	orf19.993	20135	IPF20134	No significant S.c. match
CA3524	1.0	1.1	0.9	LKH1.3	complemer	PROBABLE LEUKOTF	orf19.992	6778	IPF20135	TRANSCRIPTION SUBCELLULAR LOCALISATION
CA3525	1.0	1.0	0.9	DJP1	complemer	DnaJ-like protein involv	orf19.8606	6776	CaLKH1.3	Lipid fatty-a peptidase activity
CA3526	1.0	1.0	1.0	MRPS5	complemer	Probable ribosomal pr	orf19.8604	9936	CaDJP1	PROTEIN I chaperone activity
CA3527	1.2	1.0	0.9	IPF9934	complemer	unknown function	orf19.8603	9934	CaMRPS5	PROTEIN S structural molecule activity
CA3528	1.1	1.1	1.0	GLY1	complemer	L-threonine aldolase	orf19.8601	9931	IPF9934	UNCLASSI molecular_function unknown
CA3529	0.9	0.9	1.0	IPF9929	complemer	unknown function	orf19.8600	9929	CaGLY1	Amino acid metabolism
CA3530	0.9	1.0	0.9	IPF9926	complemer	alkaline phosphatase (	orf19.984	9926	IPF9929	UNCLASSI molecular_function unknown
CA3532	1.0	1.0	1.0	IPF13448	complemer	similar to Saccharomy	orf19.6365	13448	IPF9926	Phosphate metabolism SUBCELLULAR LOCALISATION
CA3533	0.6	0.5	1.0	IPF13450	11719589..	unknown function	orf19.6366	13450	IPF13448	CELLULAF protein phosphatase activity
CA3534	0.8	1.2	1.1	SSB1	11720670..	heat shock protein 70	orf19.6367	13454	IPF13450	No significant S.c. match
CA3535	1.0	0.9	0.9	IPF13458	complemer	unknown function	orf19.6369	13458	CaSSB1	PROTEIN S chaperone activity
CA3536	0.9	0.8	1.0	IPF5414	11724185..	unknown function	orf19.6371	5414	IPF13458	UNCLASSI protein kinase activity
CA3537	1.3	1.1	1.1	PSU1	complemer	suppressor of petit mut	orf19.6373	5418	IPF5414	No significant S.c. match
CA3538	1.0	1.0	1.1	ATP10	11728576..	F1F0 ATPase complex	orf19.6374	5420	CaPSU1	ENERGY hydrolase activity
CA3539	2.6	2.5	1.4	RPS22	11730432..	ribosomal protein by hc	orf19.6375	5422	CaATP10	PROTEIN I molecular_function unknown
CA3540	1.0	1.1	1.0	PTC5	11731874..	Type 2C Protein Phos	orf19.6376	5424	CaRPS22	PROTEIN S structural molecule activity
CA3541	0.8	0.8	0.9	IPF5425	complemer	similar to Saccharomy	orf19.6377	5425	CaPTC5	PROTEIN I hydrolase activity
CA3542	1.0	1.1	1.0	IPF5426	complemer	putative methyltransfer	orf19.6378	5426	IPF5425	PROTEIN I transferase activity
CA3543	1.0	1.0	1.0	IPF5428	11736291..	unknown function	orf19.6379	5428	IPF5426	UNCLASSI molecular_function unknown
CA3544	0.9	0.7	1.0	BPT1.3F	complemer	membrane transporter	orf19.6382	12646	IPF5428	No significant S.c. match
CA3545	0.9	1.0	1.0	BPT1.5F	complemer	membrane transporter	orf19.6383	12648	CaBPT1.3f	CELL RESCUE DEFENSE AND VIRULENCE ""TRANSPORT FACILITATION
CA3546	0.3	0.4	0.2	ACO1	complemer	aconitate hydratase (b	orf19.6385	12652	CaBPT1.5f	CELL RESCUE DEFENSE AND VIRULENCE ""TRANSPORT FACILITATION
CA3547	1.1	1.0	1.0	ADE13	complemer	adenylosuccinate lyase	orf19.3870	12183	CaACO1	C-compour lyase activity
CA3548	1.0	1.0	1.0	IPF12179	11754067..	unknown function	orf19.3872	12179	CaADE13	Nucleotide lyase activity
CA3549	1.0	0.9	1.1	ARC40	11755199..	subunit of the Arp2/3 c	orf19.3873	12178	IPF12179	UNCLASSI molecular_function unknown
CA3550	1.1	1.0	1.1	IPF13228	11758649..	unknown function	orf19.3874	13228	CaARC40	CELL FATI structural molecule activity
CA3551	0.9	0.9	0.9	IPF13229	complemer	unknown function	orf19.3876	13229	IPF13228	No significant S.c. match
CA3552	0.9	0.9	1.0	IPF13231	11763102..	unknown function	orf19.3877	13231	IPF13229	UNCLASSI molecular_function unknown
CA3553	0.9	0.8	1.1	IFA16.5	11764065..	unknown function, 5-pr	orf19.3878	13232	IPF13231	No significant S.c. match
CA3554	1.0	1.0	1.1	IPF7770	complemer	unknown function	orf19.3881	7770	CaIFA16.5	No significant S.c. match
CA3555	0.8	0.8	1.0	IPF7766	complemer	unknown function	orf19.3884	7766	IPF7770	UNCLASSI molecular_function unknown
CA3556	0.9	0.9	0.9	IPF7764	11771032..	unknown function	orf19.3885	7764	IPF7766	CELLULAR TRANSPORT AND TRANSPORT MECHANISMS
CA3557	0.9	1.0	1.0	IPF7763	complemer	unknown function	orf19.3886	7763	IPF7764	No significant S.c. match
CA3558	1.0	0.9	1.0	IPF7760	complemer	unknown function	orf19.3887	7760	IPF7763	CELL CYCLE AND DNA PROCESSING SUBCELLULAR LOCALISATION
CA3559	2.3	4.2	2.1	PGI1	complemer	Glucose-6-phosphate i	orf19.3888	7759	IPF7760	No significant S.c. match
CA3560	1.0	1.1	1.0	CZF1	11792323..	canal zinc finger protei	orf19.3127	10612	CaPGI1	C-compour isomerase activity
CA3561	1.6	1.8	2.2	SLY1	11794297..	hydrophilic suppressor	orf19.3128	10610	CaCZF1	No significant S.c. match
CA3562	1.0	1.0	1.0	IPF7823	complemer	similar to Saccharomy	orf19.3129	7823	CaSLY1	CELLULAF protein binding
CA3563	1.0	0.9	1.0	IPF7819	complemer	unknown function	orf19.3130	7819	IPF7823	CELL CYC hydrolase activity
CA3564	1.0	1.0	1.0	IPF7817	11799576..	putative NADH-depenc	orf19.3131	7817	IPF7819	UNCLASSI molecular_function unknown
CA3565	1.0	0.9	1.0	MSC2	complemer	Probable membrane pr	orf19.3132	7815	IPF7817	ENERGY

CA3566	1.0	1.0	1.0	GUT2	complemen	Glycerol-3-phosphate (	orf19.3133	7812	CaMSC2	UNCLASSI	transporter activity
CA3567	1.0	1.0	0.9	IPF7804.5f	11809479..	unknown function, 5-pr	orf19.3134	7804	CaGUT2	C-compour	oxidoreductase activity
CA3568	1.0	0.9	1.0	IPF7804.3f	11809962..	unknown function, 3-pr	orf19.3135	7802	IPF7804.5f	No significant S.c. match	
CA3569	1.0	1.2	0.9	IPF3268	11812168..	unknown function	orf19.3136	3268	IPF7804.3f	UNCLASSI	molecular_function unknown
CA3570	0.7	0.7	1.0	NOP1	complemen	Fibrillarin	orf19.3138	3267	IPF3268	CELL CYCLE AND DNA PROCESSING	
CA3571	1.0	1.0	1.0	IPF3264	complemen	unknown function	orf19.1065	3264	CaNOP1	TRANSCR	transferase activity
CA3572	1.0	0.9	1.0	IPF18321	complemen	unknown function		18321	IPF3264	Lipid fatty-acid and isoprenoid metabolism	
CA3573	1.2	0.9	1.4	RFA2	complemen	DNA replication factor	orf19.2267	13308	IPF18321	No significant S.c. match	
CA3574	0.9	1.0	1.0	IPF19584	11822330..	unknown function	orf19.2266	19584	CaRFA2	CELL CYC	DNA binding
CA3575	0.9	0.9	1.0	IPF11108	complemen	unknown function	orf19.2265	11108	IPF19584	SUBCELLU	protein binding
CA3576	0.9	1.0	0.9	IPF11107	11826725..	probably stearoyl-CoA	orf19.2264	11107	IPF11108	CELL CYC	molecular_function unknown
CA3577	1.0	0.9	1.0	IPF11106	11828594..	unknown function	orf19.2263	11106	IPF11107	Lipid fatty-acid and isoprenoid metabolism ""SUBCELLULAR LOCALISATION	
CA3578	0.9	0.9	0.9	IPF11105	complemen	probable quinone oxido	orf19.2262	11105	IPF11106	No significant S.c. match	
CA3579	1.0	1.1	0.9	HSH49	complemen	spliceosome-associate	orf19.2261	11104	IPF11105	CLASSIFIC	molecular_function unknown
CA3580	0.9	1.0	1.0	IPF16141	complemen	unknown function	orf19.2260	16141	CaHSH49	TRANSCR	RNA binding
CA3581	1.0	1.1	1.1	IPF16143	11833107..	unknown function	orf19.2259	16143	IPF16141	UNCLASSIFIED PROTEINS	
CA3582	1.0	1.1	1.1	IPF15466	11834150..	unknown function	orf19.2258	15466	IPF16143	No significant S.c. match	
CA3583	1.0	0.9	1.1	IPF20137	complemen	unknown function	orf19.2257	20137	IPF15466	No significant S.c. match	
CA3584	1.0	1.0	1.0	IPF15468	complemen	unknown function	orf19.2256	15468	IPF20137	UNCLASSI	molecular_function unknown
CA3585	0.9	1.0	1.0	IFT1	complemen	unknown function	orf19.2253	16720	IPF15468	UNCLASSI	molecular_function unknown
CA3586	1.0	1.0	1.0	IFT3	complemen	Unknown function	orf19.2252	15939	CaIFT1	No significant S.c. match	
CA3587	0.9	1.0	1.0	AAH1	complemen	adenosine deaminase	orf19.2251	10117	CaIFT3	No significant S.c. match	
CA3588	0.9	1.0	1.0	SPE3	complemen	putrescine aminopropy	orf19.2250	10115	CaAAH1	Nucleotide	hydrolase activity
CA3589	1.0	0.9	1.1	IPF10113	11847040..	unknown function	orf19.2249	10113	CaSPE3	Secondary	transferase activity
CA3590	0.9	1.1	1.0	ARE2	11849844..	acyl-CoA sterol acyltra	orf19.2248	10110	IPF10113	UNCLASSI	molecular_function unknown
CA3591	1.0	1.0	1.0	IFT2	11853104..	unknown function	orf19.2247	12902	CaARE2	Lipid fatty-a	transferase activity
CA3592	1.0	1.0	1.0	IPF12900	11854883..	unknown function	orf19.2246	12900	CaIFT2	TRANSPORT FACILITATION	
CA3593	1.1	1.0	1.1	YPT71	complemen	GTP-binding protein of	orf19.2245	12899	IPF12900	UNCLASSI	molecular_function unknown
CA3594	1.1	1.0	1.0	IPF6076.3	complemen	unknown function, 3-pr	orf19.4922	6076	CaYPT71	CELLULAR	hydrolase activity
CA3595	1.0	1.1	0.9	IPF6079	complemen	putative permease (by	orf19.4923	6079	IPF6076.3	UNCLASSI	enzyme regulator activity
CA3596	1.0	1.0	1.0	IPF6085	11863395..	unknown function	orf19.4924	6085	IPF6079	C-compound and carbohydrate metabolism TRANSPORT FACILITATION	
CA3597	1.2	1.4	1.1	BNI1	complemen	regulator of budding (b	orf19.4927	6091	IPF6085	No significant S.c. match	
CA3598	1.0	1.1	1.0	SEC2	complemen	GDP/GTP exchange fa	orf19.1239	6094	CaBNI1	CELL CYC	protein binding
CA3599	1.0	0.9	1.0	IPF19785	11879559..	unknown function	orf19.4929	19785	CaSEC2	CELLULAR	enzyme regulator activity
CA3600	1.0	0.9	1.3	SPC3	complemen	signal peptidase subur	orf19.4930	15621	IPF19785	TRANSCR	translation regulator activity
CA3601	1.0	1.0	1.1	IPF15618	complemen	cysteinyl-tRNA synthet	orf19.4931	15618	CaSPC3	PROTEIN I	peptidase activity
CA3602	1.6	2.3	1.4	RPL14B.3	11887878..	ribosomal protein L14B, 3-prime e		12937	IPF15618	PROTEIN S	ligase activity
CA3603	1.0	1.0	1.0	MEF1	complemen	mitochondrial translatio	orf19.4932	12940	CaRPL14B	PROTEIN S	RNA binding
CA3604	1.0	1.0	1.0	IPF12942	complemen	delta-12 fatty acid desa	orf19.4933	12942	CaMEF1	PROTEIN S	translation regulator activity
CA3605	1.0	1.0	1.0	IPF3865	complemen	unknown function	orf19.22	3865	IPF12942	No significant S.c. match	
CA3606	1.1	1.0	1.1	RTA3	11902679..	Unknown function	orf19.23	3859	IPF3865	SUBCELLULAR LOCALISATION	
CA3607	1.1	1.2	1.0	RTA2	11905078..	Unknown function	orf19.24	3858	CaRTA3	UNCLASSIFIED PROTEINS	
CA3608	1.1	1.1	1.1	IPF3857	11906652..	unknown function	orf19.25	3857	CaRTA2	UNCLASSI	transporter activity
CA3609	1.1	1.1	1.1	IPF3856	11907851..	similar to Saccharomy	orf19.26	3856	IPF3857	UNCLASSI	transferase activity
CA3610	1.0	1.1	1.0	IPF3854	11909848..	unknown function	orf19.27	3854	IPF3856	PROTEIN I	enzyme regulator activity
CA3611	1.1	1.1	1.0	IPF3853	complemen	unknown function	orf19.28	3853	IPF3854	No significant S.c. match	
CA3612	1.1	1.0	1.0	IPF3852	11912464..	unknown function	orf19.29	3852	IPF3853	Nucleotide	transporter activity
CA3613	1.0	1.0	1.0	SPF1	complemen	P-type ATPase	orf19.30	3849	IPF3852	CELL CYC	molecular_function unknown
CA3614	1.1	1.1	1.0	IPF8878	11918837..	unknown function	orf19.31	8878	CaSPF1	REGULATI	transporter activity
CA3615	1.0	0.9	1.0	GIT1	complemen	glycerophosphoinosito	orf19.34	8874	IPF8878	CELL RESCUE DEFENSE AND VIRULENCE ""CELL FATE	
CA3616	0.9	1.1	0.9	ASN1	11927767..	asparagine synthetase (by homolo		19994	CaGIT1	Lipid fatty-a	transporter activity
CA3617	1.0	1.0	1.0	IPF9069	11929978..	unknown function	orf19.199	9069	CaASN1	Amino acid	ligase activity
CA3618	1.1	1.0	0.9	THO1	complemen	suppressor of the hpr1	orf19.200	20138	IPF9069	ENERGY	
CA3619	1.0	1.0	1.0	CDC47.5	complemen	cell division control prc	orf19.202	9065	CaTHO1	UNCLASSI	molecular_function unknown
CA3620	1.0	1.1	1.1	IPF9063	complemen	similar to Saccharomy	orf19.203	9063	CaCDC47.	CELL CYC	DNA binding
CA3621	1.0	1.1	1.0	IPF8942	11940739..	unknown function	orf19.204	8942	IPF9063	TRANSCR	molecular_function unknown
CA3622	1.0	1.1	1.0	IPF12022	11947135..	extracellular alpha-1,4-	orf19.7836	12022	IPF8942	No significant S.c. match	
CA3623	0.9	0.8	1.0	IPF15911	11952810..	unknown function	orf19.7838	15911	IPF12022	No significant S.c. match	
CA3625	1.0	0.8	0.9	CYR1.3F	complemen	adenylate cyclase, 3-p	orf19.1261	12339	IPF15911	No significant S.c. match	
CA3626	1.0	0.9	0.9	CYR1.5F	complemen	adenylate cyclase, 5-p	orf19.1261	12336	CaCYR1.3	Nucleotide	lyase activity
CA3627	1.1	1.1	1.0	IPF18318	11965072..	unknown function	orf19.1261	18318	CaCYR1.5	Nucleotide metabolism CELL CYCLE AND DNA PROCESSING CELLULAR COMMUNICATION/SIGNAL TRANSDUCTION MECHANISM	
CA3628	1.1	0.9	1.0	IPF18316.3	complemen	unknown function, 3-pr	orf19.1261	18317	IPF18318	UNCLASSI	molecular_function unknown
CA3629	1.0	1.1	1.1	IPF18316.5	complemen	unknown function, 5-pr	orf19.1261	18316	IPF18316.3	Nitrogen and sulphur metabolism	
CA3630	1.0	1.0	1.0	IPF8129	11970757..	unknown function	orf19.1260	8129	IPF18316.5	No significant S.c. match	
CA3631	1.0	1.0	0.9	TIM54	11972073..	Translocase for the ins	orf19.1260	8130	IPF8129	No significant S.c. match	
CA3632	1.0	1.0	0.9	DFR1	complemen	dihydrofolate reductase	orf19.5142	8131	CaTIM54	PROTEIN I	transporter activity
CA3633	0.8	0.6	1.0	IFA19	11974620..	unknown function	orf19.5141	8136	CaDFR1	Amino acid	oxidoreductase activity
CA3634	1.1	1.1	1.1	IFA25	11977577..	unknown function	orf19.5140	8138	CaIFA19	UNCLASSIFIED PROTEINS	
CA3635	0.9	0.9	0.9	IFA20	11980400..	unknown function	orf19.5139	8140	CaIFA25	Nucleotide metabolism CELL CYCLE AND DNA PROCESSING CELLULAR COMMUNICATION/SIGNAL TRANSDUCTION MECHANISM	
CA3636	1.0	1.0	0.9	IFA21	11983197..	unknown function	orf19.1260	16529	CaIFA20	Nucleotide metabolism CELL CYCLE AND DNA PROCESSING CELLULAR COMMUNICATION/SIGNAL TRANSDUCTION MECHANISM	
CA3637	1.0	0.9	1.0	IPF9255	complemen	unknown function	orf19.5136	9255	CaIFA21	CELL RESCUE DEFENSE AND VIRULENCE	
CA3638	1.0	1.0	1.0	IPF9252	11990889..	unknown function	orf19.5134	9252	IPF9255	UNCLASSI	molecular_function unknown

CA3639	1.2	1.0	1.2	IPF9251	complemer	unknown function	orf19.5133	9251	IPF9252	UNCLASSIFIED PROTEINS	
CA3640	1.0	1.0	0.9	VPS24	12002153..	endosomal Vps protei	orf19.2031	4120	IPF9251	Lipid fatty-a	DNA binding
CA3641	1.0	1.0	0.9	IPF4122	complemer	unknown function	orf19.2032	4122	CaVPS24	PROTEIN I	molecular_function unknown
CA3642	0.9	1.0	1.0	IPF4123	complemer	unknown function	orf19.2033	4123	IPF4122	No significant S.c. match	
CA3643	0.9	0.9	0.9	IPF4124	complemer	unknown function	orf19.2034	4124	IPF4123	No significant S.c. match	
CA3644	1.0	1.0	1.1	IPF4126	complemer	unknown function	orf19.2035	4126	IPF4124	No significant S.c. match	
CA3645	1.0	1.0	0.9	IPF4127	complemer	putative dimeric dihydr	orf19.2036	4127	IPF4126	TRANSPORT FACILITATION	
CA3646	1.0	1.0	0.9	IPF4128	complemer	unknown function	orf19.2037	4128	IPF4127	No significant S.c. match	
CA3647	1.1	0.9	1.4	IPF4129	12009894..	unknown function	orf19.2038	4129	IPF4128	No significant S.c. match	
CA3648	0.9	0.9	1.0	MSF1	complemer	phenylalanine--tRNA li	orf19.2039	4131	IPF4129	UNCLASSIFIED PROTEINS	
CA3649	1.0	1.0	1.0	IPF4132	complemer	unknown function	orf19.2040	4132	CaMSF1	PROTEIN S	ligase activity
CA3650	1.0	0.9	1.0	IPF4134	12014907..	unknown function	orf19.2041	4134	IPF4132	Lipid fatty-a	molecular_function unknown
CA3651	0.9	0.9	1.1	MSS1	complemer	Mitochondrial GTPase	orf19.2042	7447	IPF4134	CELL CYC	molecular_function unknown
CA3652	1.0	1.1	1.1	NBP35	12018549..	Nucleotide-binding pro	orf19.2043	7450	CaMSS1	TRANSCRIPTION PROTEIN SYNTHESIS SUBCELLULAR LOCALISATION	
CA3653	0.9	0.9	0.9	IPF7451	12019654..	unknown function	orf19.2044	7451	CaNBP35	UNCLASSIFIED PROTEINS	
CA3654	1.0	1.0	1.0	IPF7452	12021656..	unknown function	orf19.2045	7452	IPF7451	No significant S.c. match	
CA3655	1.1	0.9	1.0	POT13	complemer	Acetyl-CoA C-acyltrans	orf19.2046	7453	IPF7452	UNCLASSI	molecular_function unknown
CA3656	1.4	1.2	1.1	IPF7456	complemer	unknown function	orf19.2047	7456	CaPOT13	Lipid fatty-acid and isoprenoid metabolism ""ENERGY SUBCELLULAR LOCALISATION	
CA3657	1.3	1.0	1.3	IPF7459	complemer	unknown function	orf19.2049	7459	IPF7456	UNCLASSI	molecular_function unknown
CA3658	1.0	1.1	1.0	TGL1	12028548..	Triacylglycerol lipase (l	orf19.2050	7463	IPF7459	No significant S.c. match	
CA3660	1.0	0.9	0.9	IPF17794	12030737..	unknown function		17994	CaTGL1	Lipid fatty-a	hydrolase activity
CA3661	0.9	1.0	1.0	IPF17991	complemer	unknown function	orf19.6465	17991	IPF17794	No significant S.c. match	
CA3662	1.0	1.1	1.0	IPF17021	12037174..	unknown function	orf19.6464	17021	IPF17991	C-compound and carbohydrate metabolism SUBCELLULAR LOCALISATION	
CA3663	0.9	0.9	1.0	IPF11432	complemer	unknown function	orf19.6463	11432	IPF17021	Nucleotide metabolism	
CA3664	1.0	1.0	1.0	IPF11433.3	complemer	unknown function, 3-pr	orf19.6462	11433	IPF11432	UNCLASSI	molecular_function unknown
CA3666	1.0	1.0	0.9	IPF11435	complemer	unknown function	orf19.6461	11435	IPF11433.3	UNCLASSI	molecular_function unknown
CA3667	1.0	1.0	1.0	IPF16323	12043118..	similar to Saccharomy	orf19.6460	16323	IPF11435	ENERGY C	chaperone activity
CA3668	1.1	1.0	1.0	DPP3	complemer	Diacylglycerol Pyrophc	orf19.6459	19996	IPF16323	PROTEIN I	hydrolase activity
CA3669	1.1	1.1	1.0	IPF19997	12048788..	by homology to S. cerevisiae: U6 s		19997	CaDPP3	Lipid fatty-a	hydrolase activity
CA3670	1.0	1.0	1.0	IPF13934	12049541..	unknown function	orf19.6458	13934	IPF19997	No significant S.c. match	
CA3671	1.0	1.0	1.0	IPF13933	complemer	unknown function	orf19.6457	13933	IPF13934	No significant S.c. match	
CA3672	0.9	1.0	0.9	IPF13669	12053915..	unknown function	orf19.6456	13669	IPF13933	C-compour	molecular_function unknown
CA3673	0.9	0.8	0.9	IPF13667	complemer	unknown function	orf19.6455	13667	IPF13669	No significant S.c. match	
CA3674	1.1	1.0	1.0	IPF19529.E	12056759..	unknown function, exo	orf19.6454	19529	IPF13667	UNCLASSI	molecular_function unknown
CA3675	1.0	1.0	0.9	IPF19529.E	12058245..	unknown function, exo	orf19.6453	18311	IPF19529.e	No significant S.c. match	
CA3676	1.0	1.5	1.2	RBP1	complemer	rapamycin-binding prot	orf19.6452	8077	IPF19529.e	No significant S.c. match	
CA3677	1.0	1.0	0.9	SOU3	12070103..	putative sorbitol utilizat	orf19.732	8391	CaRBP1	PROTEIN I	isomerase activity
CA3678	1.0	0.9	1.0	IPF8392	12071254..	unknown function	orf19.731	8392	CaSOU3	ENERGY SUBCELLULAR LOCALISATION	
CA3679	1.0	1.0	1.0	IPF20142	12073061..	unknown function	orf19.730	20142	IPF8392	CLASSIFIC	molecular_function unknown
CA3680	0.9	0.8	1.0	IPF8395	12076135..	unknown function	orf19.729	8395	IPF20142	CELL FATI	enzyme regulator activity
CA3681	0.9	0.8	0.9	IPF19787	complemer	unknown function	orf19.728	19787	IPF8395	No significant S.c. match	
CA3683	1.0	1.0	1.0	PPZ1	complemer	ser/thr phosphatase re	orf19.726	13615	IPF19787	UNCLASSI	protein binding
CA3684	1.0	0.9	1.0	IPF13613	12100562..	unknown function	orf19.725	13613	CaPPZ1	CELL RES	protein phosphatase activity
CA3685	1.1	1.2	1.1	IPF19540	complemer	unknown function	orf19.723	19540	IPF13613	No significant S.c. match	
CA3686	1.0	1.1	0.9	IPF4890	12111800..	unknown function	orf19.1364	4890	IPF19540	TRANSCR	molecular_function unknown
CA3687	0.9	0.9	0.9	IPF4889	complemer	unknown function	orf19.6264	4889	IPF4890	TRANSPO	transporter activity
CA3688	1.0	1.0	1.1	YIP3.3	12115214..	protein of unknown function, 3-prir		4885	IPF4889	C-compound and carbohydrate metabolism ENERGY TRANSCRIPTION CELL FATE SUBCELLULAR LOCALISATION	
CA3689	1.4	1.9	1.6	RPS22A	12116904..	ribosomal protein S15a	orf19.6265	4882	CaYIP3.3	CELLULAF	molecular_function unknown
CA3690	1.6	1.3	1.3	RPS14B	12118211..	ribosomal protein (by homology)		4881	CaRPS22A	PROTEIN S	structural molecule activity
CA3691	0.9	0.9	0.9	IPF4880	12118919..	unknown function	orf19.6266	4880	CaRPS14B	PROTEIN S	RNA binding
CA3692	1.0	1.0	1.0	IPF4876	12122185..	unknown function	orf19.6267	4876	IPF4880	TRANSCRIPTION CELL FATE SUBCELLULAR LOCALISATION	
CA3693	0.9	0.9	1.0	IPF4874	12125054..	unknown function	orf19.6268	4874	IPF4876	No significant S.c. match	
CA3694	1.0	0.9	1.0	IPF4872.3F	complemer	unknown function, 3-pr	orf19.6269	4873	IPF4874	UNCLASSI	molecular_function unknown
CA3695	1.0	1.1	1.0	IPF4872.5F	complemer	unknown function, 5-pr	orf19.6270	4872	IPF4872.3f	PROTEIN FATE [folding modification destination] ""CELLULAR TRANSPORT AND TRANSPORT MECHANISMS SUBCELLULAR LOCAL	
CA3696	1.0	1.1	1.0	IPF4868	12131198..	unknown function	orf19.1365	4868	IPF4872.5f	No significant S.c. match	
CA3697	0.9	0.8	1.0	IPF4866	complemer	similar to Saccharomy	orf19.1365	4866	IPF4868	SUBCELLU	molecular_function unknown
CA3698	1.0	0.8	1.0	IPF4861	12137078..	unknown function	orf19.6274	4861	IPF4866	PROTEIN I	peptidase activity
CA3699	1.0	1.0	1.0	IPF4860	complemer	similarity to ribosomal	orf19.6275	4860	IPF4861	No significant S.c. match	
CA3700	0.9	1.0	0.9	IPF4859	complemer	unknown function	orf19.6276	4859	IPF4860	CLASSIFIC	protein kinase activity
CA3701	1.1	1.1	1.0	IPF11936.3	complemer	unknown function, 5-pr	orf19.1365	19586	IPF4859	No significant S.c. match	
CA3703	0.8	0.8	0.8	IPF11945	complemer	unknown function	orf19.6281	11945	IPF11936.3	No significant S.c. match	
CA3704	0.9	0.9	1.0	IPF5671	complemer	unknown function	orf19.4946	5671	IPF11945	No significant S.c. match	
CA3705	1.1	0.9	1.2	MSH6	12160626..	DNA mismatch repair	orf19.4945	5670	IPF5671	No significant S.c. match	
CA3706	1.8	2.7	1.4	PSA2	complemer	mannose-1-phosphate	orf19.4943	5668	CaMSH6	CELL CYC	DNA binding
CA3707	0.8	0.8	0.5	TYE7	12170168..	Basic helix-loop-helix t	orf19.4941	5663	CaPSA2	C-compound and carbohydrate metabolism CONTROL OF CELLULAR ORGANIZATION	
CA3708	1.0	1.0	1.1	HIP1	12171574..	Histidine permease (by	orf19.4940	7876	CaTYE7	C-compour	DNA binding,transcription regulator activity
CA3709	0.9	1.0	1.0	IPF7880	12173743..	unknown function	orf19.4939	7880	CaHIP1	Amino acid metabolism CELLULAR TRANSPORT AND TRANSPORT MECHANISMS REGULATION OF/INTERACTION WITH CELLULA	
CA3710	0.8	0.8	0.9	CHS3.5F	12180005..	chitin-UDP acetyl-gluc	orf19.4938	7886	IPF7880	UNCLASSI	molecular_function unknown
CA3711	1.1	1.1	1.0	CHS3.3F	12180306..	chitin-UDP acetyl-gluc	orf19.4937	7889	CaCHS3.5	C-compound and carbohydrate metabolism CELL FATE CONTROL OF CELLULAR ORGANIZATION SUBCELLULAR LOCALISATION	
CA3712	1.0	1.1	1.1	IPF7891	complemer	unknown function	orf19.4936	7891	CaCHS3.3	C-compour	transferase activity
CA3713	1.1	1.1	1.0	OPS4	complemer	opaque - phase specifi	orf19.4934	15453	IPF7891	No significant S.c. match	
CA3714	1.0	0.9	1.0	IPF16223	12194080..	unknown function	orf19.1163	16223	CaOPS4	No significant S.c. match	

CA3715	1.0	1.0	1.0	IPF6205	12197699..	unknown function	orf19.4164	6205	IPF16223	UNCLASSI	molecular_function unknown
CA3716	0.9	1.0	0.9	IPF6203	complemer	unknown function	orf19.1164:	6203	IPF6205	UNCLASSI	RNA binding
CA3717	1.0	1.0	1.0	IPF6192	12205742..	unknown function	orf19.1164:	6192	IPF6203	TRANSCRIPTION	
CA3718	1.0	0.8	0.9	POP7	complemer	Nuclear RNase P subu	orf19.1164	6191	IPF6192	UNCLASSI	molecular_function unknown
CA3719	0.9	0.9	1.0	IPF6190	12207199..	unknown function	orf19.1164	6190	CaPOP7	TRANSCR	RNA binding
CA3720	0.9	0.8	1.0	IFL3	complemer	Unknown function	orf19.1164	6188	IPF6190	No significant S.c. match	
CA3721	0.9	0.9	0.9	IPF6186	complemer	unknown function	orf19.1164	6186	CaIFL3	CELL RESCUE DEFENSE AND VIRULENCE ""CELL FATE	
CA3722	1.0	1.1	1.0	PHO13	12212650..	4-nitrophenylphosphat	orf19.4172	6184	IPF6186	No significant S.c. match	
CA3723	1.0	1.1	0.9	DPH2	12213872..	Diphtheria toxin resista	orf19.4173	6183	CaPHO13	Phosphate metabolism SUBCELLULAR LOCALISATION	
CA3724	1.0	1.0	1.0	IPF6181	complemer	similar to Saccharomy	orf19.4174	6181	CaDPH2	Amino acid	molecular_function unknown
CA3725	0.9	1.1	0.9	TOK1.3	complemer	Outward-rectifier potas	orf19.4175	6179	IPF6181	Nucleotide	transporter activity
CA3726	0.9	1.1	0.9	IPF6175	complemer	unknown function	orf19.4176	6175	CaTOK1.3	CELLULAF	transporter activity
CA3727	0.9	1.1	0.9	HIS5.3F	complemer	Histidinol-phosphate a	orf19.4177	6173	IPF6175	PROTEIN !	structural molecule activity
CA3729	0.9	1.0	1.0	IPF11901	complemer	unknown function	orf19.4179	11901	CaHIS5.3f	Amino acid	transferase activity
CA3730	1.0	0.9	0.9	IPF11900	12223031..	unknown function	orf19.4180	11900	IPF11901	No significant S.c. match	
CA3731	1.0	1.0	1.1	SPC2	12224114..	signal peptidase 18 kD	orf19.4181	11899	IPF11900	PROTEIN I	transporter activity
CA3732	0.9	1.0	1.0	IPF11898	complemer	unknown function	orf19.4182	11898	CaSPC2	PROTEIN I	protein binding
CA3733	1.2	1.1	1.0	IPF11897	12225959..	unknown function	orf19.1165	11897	IPF11898	UNCLASSIFIED PROTEINS	
CA3735	1.0	1.0	0.9	IPF5534	12229437..	5-oxo-1,2,5-tricarboxil	orf19.2184	5534	IPF11897	C-compound and carbohydrate metabolism SUBCELLULAR LOCALISATION	
CA3736	0.9	1.3	1.1	IPF5533	complemer	ABC transporter (by hc	orf19.2183	5533	IPF5534	UNCLASSIFIED PROTEINS	
CA3737	1.0	1.0	1.0	BLM3	12235622..	bleomycin resistance (	orf19.2182	5529	IPF5533	TRANSPO	transporter activity,hydrolase activity
CA3738	1.1	1.0	1.0	IPF5526	complemer	unknown function	orf19.2180	5526	CaBLM3	UNCLASSI	molecular_function unknown
CA3739	1.3	1.4	1.2	RPS10.3	complemer	ribosomal protein, 3-prime end (by		5524	IPF5526	UNCLASSI	molecular_function unknown
CA3740	1.1	1.1	1.0	SIT1	complemer	Ferrioxamine B perme	orf19.2179	5522	CaRPS10.3	PROTEIN !	structural molecule activity
CA3741	1.0	0.9	1.0	MRS4	complemer	RNA splicing protein a	orf19.2178	5513	CaSIT1	CELL RES	transporter activity
CA3742	1.0	0.9	0.9	IFM3	complemer	2-hydroxyacid dehydrc	orf19.2176	5511	CaMRS4	TRANSCR	transporter activity
CA3743	1.1	1.5	1.1	IPF19998	12253720..	unknown function	orf19.2175	19998	CaIFM3	C-compound and carbohydrate metabolism	
CA3744	1.0	0.9	0.9	RAD57	12255054..	DNA-repair like protein	orf19.2174	10144	IPF19998	UNCLASSI	oxidoreductase activity
CA3745	1.0	1.0	0.9	MAF1	12257282..	nuclear protein by hom	orf19.2173	10146	CaRAD57	CELL CYC	protein binding
CA3746	1.0	1.0	1.0	ARA1	12258916..	D-arabinose dehydrogε	orf19.2172	10148	CaMAF1	PROTEIN I	molecular_function unknown
CA3747	1.1	1.5	1.0	IPF10153	complemer	membrane transporter	orf19.2170	10153	CaARA1	C-compour	oxidoreductase activity
CA3748	0.8	1.1	0.9	IPF7385	12269447..	unknown function	orf19.3430	7385	IPF10153	UNCLASSI	molecular_function unknown
CA3749	0.9	1.0	1.0	IPF7389	complemer	unknown function	orf19.3429	7389	IPF7385	No significant S.c. match	
CA3750	1.1	1.0	1.1	IPF7377	12278199..	unknown function	orf19.3428	7377	IPF7389	No significant S.c. match	
CA3751	1.1	1.0	0.9	IPF7378	12279864..	unknown function	orf19.3427	7378	IPF7377	UNCLASSIFIED PROTEINS	
CA3752	1.6	2.3	1.6	TIF51.3	12281670..	translation initiation fac	orf19.3426	20144	IPF7378	No significant S.c. match	
CA3753	1.0	1.1	1.0	IPF14743	12284650..	unknown function	orf19.3425	14743	CaTIF51.3	UNCLASSI	translation regulator activity
CA3754	0.6	0.8	0.6	TIF3	12286967..	translation initiation fac	orf19.3423	16643	IPF14743	UNCLASSIFIED PROTEINS	
CA3755	1.0	1.0	1.0	IPF17743	12289536..	Unknown function		17743	CaTIF3	PROTEIN !	translation regulator activity
CA3756	1.0	1.0	1.0	IPF8884	complemer	unknown function	orf19.3422	8884	IPF17743	TRANSCR	transcription regulator activity
CA3757	1.1	1.4	1.0	MAE1	12300384..	mitochondrial malic en	orf19.3419	8888	IPF8884	UNCLASSI	molecular_function unknown
CA3758	1.0	0.9	1.0	IPF8889	complemer	putative arginase (by h	orf19.1092:	8889	CaMAE1	C-compour	oxidoreductase activity
CA3759	0.9	0.9	1.0	IPF13139	12310473..	unknown function	orf19.2910	13139	IPF8889	Amino acid metabolism Nitrogen and sulphur metabolism SUBCELLULAR LOCALISATION	
CA3760	0.9	1.0	1.1	ERG26	complemer	C-3 sterol dehydrogena	orf19.2909	13138	IPF13139	No significant S.c. match	
CA3761	1.0	1.0	1.1	IFR3	complemer	unknown function	orf19.2908	13137	CaERG26	Lipid fatty-a	oxidoreductase activity
CA3762	1.0	0.9	1.0	IPF13135	complemer	unknown function	orf19.2907	13135	CaIFR3	SUBCELLULAR LOCALISATION	
CA3763	0.9	0.9	1.0	IPF13070	complemer	unknown function	orf19.2906	13070	IPF13135	No significant S.c. match	
CA3764	1.0	1.0	0.9	IPF13072	complemer	unknown function	orf19.2905	13072	IPF13070	No significant S.c. match	
CA3765	0.9	0.9	1.0	IPF18298	12321271..	unknown function	orf19.2904	18298	IPF13072	No significant S.c. match	
CA3766	1.6	1.6	1.0	IPF18298.3	12321970..	unknown function, 3-pr	orf19.2903	16014	IPF18298	Lipid fatty-acid and isoprenoid metabolism	
CA3767	0.9	1.1	0.9	NUP60	complemer	Putative nuclear pore p	orf19.2901	13471	IPF18298.3	No significant S.c. match	
CA3768	0.9	1.0	1.0	IPF13467	complemer	Putative peroxisomal 2	orf19.2899	13467	CaNUP60	CELLULAF	structural molecule activity
CA3769	1.0	0.9	1.0	IPF13465	complemer	Putative anion transpo	orf19.2898	13465	IPF13467	ENERGY SUBCELLULAR LOCALISATION	
CA3770	1.0	1.0	0.9	SOU2	12331220..	Sorbitol utilization prot	orf19.2897	13462	IPF13465	CLASSIFIC	transporter activity
CA3771	1.0	1.0	1.0	SOU1	12332930..	Sorbitol utilization prot	orf19.2896	11150	CaSOU2	ENERGY SUBCELLULAR LOCALISATION	
CA3772	0.9	1.0	0.9	VMA8	complemer	CANAL VACUOLAR A	orf19.2895	11151	CaSOU1	ENERGY SUBCELLULAR LOCALISATION	
CA3773	0.9	1.0	1.0	IPF11153	12335071..	unknown function	orf19.2893	11153	CaVMA8	CELLULAF	transporter activity
CA3774	1.0	1.0	1.0	IPF10437	12340660..	budding protein-like (b	orf19.2892	10437	IPF11153	CELL CYC	molecular_function unknown
CA3775	1.0	1.0	0.9	AFG1	complemer	ATPase family gene (b	orf19.2891	10436	IPF10437	CELL FATI	molecular_function unknown
CA3776	0.8	0.5	0.9	IPF10435	complemer	unknown function	orf19.2890	10435	CaAFG1	CLASSIFIC	hydrolase activity
CA3777	1.1	1.1	1.0	IPF10432	12347748..	unknown function	orf19.2889	10432	IPF10435	UNCLASSIFIED PROTEINS	
CA3778	0.9	0.9	1.0	IPF10431	complemer	unknown function	orf19.2888	10431	IPF10432	UNCLASSI	molecular_function unknown
CA3779	1.0	0.9	1.0	IPF11221	12353921..	unknown function	orf19.5841	11221	IPF10431	No significant S.c. match	
CA3780	1.0	1.0	1.0	IPF11222	complemer	unknown function	orf19.5840	11222	IPF11221	No significant S.c. match	
CA3781	1.0	1.0	1.0	IPF11224	12355232..	similar to Saccharomy	orf19.5839	11224	IPF11222	No significant S.c. match	
CA3782	1.1	1.1	1.1	SER2	12356659..	phosphoserine phosph	orf19.5838	11225	IPF11224	Lipid fatty-a	transporter activity
CA3783	1.0	1.0	1.0	IPF11226	complemer	unknown function	orf19.5837	11226	CaSER2	Amino acid	hydrolase activity
CA3784	1.0	1.0	1.0	IPF11229	complemer	similar to Saccharomy	orf19.5835	11229	IPF11226	UNCLASSI	molecular_function unknown
CA3785	0.9	1.0	1.0	IPF19788	12359996..	similar to Saccharomy	orf19.5834	19788	IPF11229	TRANSCR	molecular_function unknown
CA3786	1.2	1.1	1.2	UFD1	12363355..	Ubiquitin fusion degrac	orf19.5833	14267	IPF19788	TRANSCR	protein binding
CA3787	0.9	0.9	1.0	HPT1	complemer	hypoxanthine guanine	orf19.5832	14266	CaUFD1	PROTEIN I	protein binding
CA3788	1.1	1.0	1.0	IPF16445	complemer	unknown function	orf19.5831	16445	CaHPT1	Nucleotide	transferase activity

CA3789	1.0	2.4	1.0	IPF11217	12368047..	similar to Saccharomy	orf19.1325	11217	IPF16445	No significant S.c. match	
CA3790	1.0	1.0	0.9	IPF11215	12371174..	unknown function	orf19.1325	11215	IPF11217	PROTEIN I	chaperone activity
CA3791	1.0	0.9	1.1	IPF11212	complemer	similar to Saccharomy	orf19.5827	11212	IPF11215	TRANSCR	RNA binding
CA3792	1.1	1.1	1.1	UGA5	complemer	GABA-SPECIFIC TRA	orf19.5826	11211	IPF11212	CELL CYC	enzyme regulator activity
CA3793	1.0	1.0	1.1	IPF5701	12377945..	unknown function		5701	CaUGA5	No significant S.c. match	
CA3794	1.1	0.9	1.0	NCB2	complemer	Transcriptional repress	orf19.5825	5700	IPF5701	No significa	structural molecule activity
CA3795	0.9	0.9	0.9	IPF5699	complemer	Abhydrolase by homol	orf19.5824	5699	CaNCB2	TRANSCR	transcription regulator activity
CA3796	1.0	1.2	1.0	SGT2	complemer	small glutamine-rich te	orf19.5823	5697	IPF5699	UNCLASSIFIED PROTEINS	
CA3797	1.0	1.0	0.9	IPF5623	complemer	unknown function	orf19.5821	5693	CaSGT2	UNCLASSI	molecular_function unknown
CA3799	0.9	1.0	1.0	IPF7198	12387813..	unknown function	orf19.1001	7198	IPF5623	UNCLASSI	molecular_function unknown
CA3800	1.2	1.6	1.2	RPL7A.3	complemer	60S Ribosomal Protein	L7-A, 3-pri	7197	IPF7198	CELL CYCLE AND DNA PROCESSING SUBCELLULAR LOCALISATION	
CA3801	1.0	1.0	1.0	UGA4	12394943..	GABA-specific transpo	orf19.1001	7194	CaRPL7A.	PROTEIN !	structural molecule activity
CA3802	1.4	2.8	1.0	AUT7.EXO	complemer	microtubule-associated	protein ess	12408	CaUGA4	No significa	transporter activity
CA3803	0.9	1.0	0.9	IPF12407	12398139..	unknown function	orf19.2481	12407	CaAUT7.e	PROTEIN I	protein binding
CA3804	1.1	0.8	1.1	RIM1	12399164..	telomere-binding prote	orf19.2483	12405	IPF12407	No significant S.c. match	
CA3805	0.9	0.8	1.1	IPF12403	complemer	unknown function	orf19.2484	12403	CaRIM1	No significant S.c. match	
CA3806	1.1	1.1	1.0	IPF9224	12401531..	similar to Saccharomy	orf19.2485	9224	IPF12403	PROTEIN I	molecular_function unknown
CA3807	1.1	1.0	1.0	IPF9225	complemer	unknown function	orf19.2487	9225	IPF9224	CELL CYC	structural molecule activity
CA3808	0.9	0.9	0.9	FAL1	12406774..	ATP-dependent RNA h	orf19.2488	9226	IPF9225	Nucleotide	molecular_function unknown
CA3809	1.1	1.5	1.1	KAP123	12408257..	karyopherin-beta prote	orf19.2489	9227	CaFAL1	TRANSCR	RNA binding,helicase activity
CA3810	1.0	1.1	1.0	IPF9230	12412100..	similar to Saccharomy	orf19.2492	9230	CaKAP123	PROTEIN I	protein binding
CA3811	0.9	1.1	1.0	PET112	complemer	glutamyl-tRNA (GLN) a	orf19.2494	9233	IPF9230	CELLULAF	signal transducer activity
CA3812	0.9	1.0	1.0	GSL22	complemer	1,3-beta-D-glucan synt	orf19.2495	4913	CaPET112	PROTEIN !	molecular_function unknown
CA3813	1.0	1.1	1.0	FRP1	12421523..	member of the FRP fa	orf19.2496	4026	CaGSL22	C-compound and carbohydrate metabolism	
CA3814	1.0	0.9	1.0	IPF4023	12423340..	unknown function	orf19.2498	4023	CaFRP1	C-compound and carbohydrate metabolism	
CA3815	1.0	1.0	0.9	CYP7	complemer	peptidyl-prolyl cis-trans	orf19.2499	4018	IPF4023	CELL FATE	molecular_function unknown
CA3816	0.9	0.9	1.0	IPF4017	12426495..	unknown function	orf19.2500	4017	CaCYP7	TRANSCR	chaperone activity
CA3818	1.4	1.2	1.2	IPF6695	12443908..	unknown function	orf19.5773	6995	IPF4017	No significant S.c. match	
CA3819	1.0	0.9	0.9	IPF6993	12451889..	unknown function	orf19.5772	6993	IPF6695	UNCLASSI	molecular_function unknown
CA3820	1.0	1.0	1.0	PBP2	12453231..	PAB1 binding protein (	orf19.5771	6992	IPF6993	UNCLASSI	molecular_function unknown
CA3821	1.2	1.1	1.0	IPF6990	complemer	unknown function	orf19.5770	6990	CaPBP2	TRANSCR	molecular_function unknown
CA3822	0.9	1.1	0.9	CUS2	12459077..	cold sensitive U2 snRN	orf19.5767	20001	IPF6990	UNCLASSI	molecular_function unknown
CA3823	1.0	1.0	0.7	IPF10651	complemer	unknown function	orf19.5765	10651	CaCUS2	TRANSCR	RNA binding
CA3824	1.0	1.1	1.0	SKI8	12462546..	antiviral protein-like (by	orf19.5764	10653	IPF10651	TRANSCR	structural molecule activity
CA3825	1.0	1.0	0.9	IPF10654	complemer	D-arabinitol dehydroge	orf19.5763	10654	CaSKI8	CELL CYC	translation regulator activity
CA3826	1.0	1.0	1.1	IPF20148	12466008..	unknown function	orf19.5762	20148	IPF10654	ENERGY SUBCELLULAR LOCALISATION	
CA3827	1.0	1.3	1.0	IPF10662	12469047..	unknown function	orf19.5760	10662	IPF20148	No significant S.c. match	
CA3828	1.0	1.0	1.0	SNQ2	12471725..	multidrug resistance pr	orf19.5759	13913	IPF10662	No significant S.c. match	
CA3829	1.2	1.1	1.1	PPQ1	complemer	phosphoprotein phospl	orf19.5758	13917	CaSNQ2	Lipid fatty-a	transporter activity
CA3830	0.9	0.9	1.0	SSF1	12479848..	mating protein (by hom	orf19.6589	8195	CaPPQ1	PROTEIN SYNTHESIS	
CA3831	1.0	10.7	1.0	VMA22	complemer	vacuolar ATPase asse	orf19.6590	8194	CaSSF1	CELL FATE	RNA binding
CA3832	1.0	1.0	1.0	IPF8193	complemer	unknown function	orf19.6591	8193	CaVMA22	PROTEIN I	chaperone activity
CA3833	0.9	1.0	1.0	IPF8192	complemer	unknown function	orf19.6592	8192	IPF8193	Metabolism	hydrolase activity
CA3834	1.1	1.0	1.0	PLB3	12488494..	phospholipase B (by h	orf19.6594	8186	IPF8192	No significant S.c. match	
CA3835	1.1	1.1	1.0	RTA4	12492448..	Protein involved in 7-a	orf19.6595	8181	CaPLB3	Lipid fatty-a	hydrolase activity
CA3836	1.0	1.0	1.0	IPF8179	12494486..	putative esterase (by h	orf19.6596	8179	CaRTA4	UNCLASSIFIED PROTEINS	
CA3838	0.9	0.9	0.9	LAS17	complemer	actin assembly factor (	orf19.6598	12743	IPF8179	Lipid fatty-a	hydrolase activity
CA3839	1.0	1.0	1.0	IPF12744	12499739..	unknown function		12744	CaLAS17	CELL CYC	protein binding
CA3840	0.8	0.5	1.0	IPF12745	12501409..	unknown function	orf19.6600	12745	IPF12744	No significant S.c. match	
CA3841	1.0	1.0	1.0	IPF16640	12503398..	unknown function	orf19.6601	16640	IPF12745	CLASSIFIC	molecular_function unknown
CA3842	2.5	3.5	1.9	YKE2.3	12505413..	Gim complex component,	3-prime	7948	IPF16640	No significant S.c. match	
CA3843	0.9	1.0	0.9	IPF7947	complemer	unknown function	orf19.6602	7947	CaYKE2.3	PROTEIN I	protein binding
CA3844	1.2	1.7	1.1	IPF7945	12508388..	unknown function	orf19.6604	7945	IPF7947	UNCLASSI	molecular_function unknown
CA3845	1.0	0.9	1.1	IPF7944	complemer	unknown function	orf19.6605	7944	IPF7945	No significant S.c. match	
CA3846	0.9	1.0	0.9	IPF7943	12510810..	unknown function	orf19.6606	7943	IPF7944	UNCLASSI	molecular_function unknown
CA3847	1.0	1.0	1.0	IPF7942	12513243..	NADH-ubiquinone oxic	orf19.6607	7942	IPF7943	No significant S.c. match	
CA3848	2.1	2.1	1.5	IPF7940	12515029..	unknown function	orf19.6608	7940	IPF7942	No significant S.c. match	
CA3849	1.0	1.0	1.0	IPF7938	12517774..	similar to Saccharomy	orf19.6610	7938	IPF7940	No significant S.c. match	
CA3850	1.0	1.0	1.0	IPF7932	12520919..	similar to Saccharomy	orf19.6612	7932	IPF7938	SUBCELLU	structural molecule activity
CA3851	1.0	1.0	1.0	CTA21	12533211..	transcriptional activatic	orf19.6112	10779	IPF7932	ENERGY !	molecular_function unknown
CA3852	1.2	1.2	1.0	TUP1	12534656..	general transcription re	orf19.6109	4657	CaCTA21	No significant S.c. match	
CA3853	0.9	1.1	0.9	MVD1.3	12536361..	mevalonate pyrophosp	orf19.6105	4652	CaTUP1	TRANSCR	transcription regulator activity
CA3854	0.9	0.8	0.9	IPF4649	complemer	unknown Function	orf19.6102	4649	CaMVD1.3	Lipid fatty-a	lyase activity
CA3855	1.0	0.9	1.0	IPF4645	12538088..	unknown Function	orf19.6103	4645	IPF4649	CELL CYC	transcription regulator activity
CA3856	1.0	1.0	1.0	IPF4641	complemer	similar to Saccharomy	orf19.6100	4641	IPF4645	UNCLASSI	molecular_function unknown
CA3857	0.9	1.2	1.0	CCT8	12539831..	component of chapero	orf19.6099	4639	IPF4641	Lipid fatty-a	transferase activity
CA3858	0.9	0.9	1.0	TRP1	complemer	phosphoribosylanthran	orf19.6096	4635	CaCCT8	PROTEIN I	chaperone activity
CA3859	1.0	1.0	1.0	IPF4632	complemer	similar to Saccharomy	orf19.6094	4632	CaTRP1	Amino acid	isomerase activity
CA3860	0.9	1.0	1.0	KEL1	12544763..	involved in cell fusion a	orf19.6092	4628	IPF4632	CELLULAF	molecular_function unknown
CA3861	1.1	1.1	1.0	RIM8	complemer	regulator of PH respon	orf19.6091	4622	CaKEL1	REGULATI	molecular_function unknown
CA3862	1.0	1.0	0.9	NSR1	complemer	nuclear localization se	orf19.6090	4616	CaRIM8	UNCLASSIFIED PROTEINS	
CA3863	0.9	1.0	1.0	LEU41	12552360..	2-isopropylmalalate sy	orf19.6086	4611	CaNSR1	TRANSCR	RNA binding

CA3864	1.3	2.2	1.5	RPL16A	12554335..	ribosomal protein (by h	orf19.6085	4609	CaLEU41	Amino acid	transferase activity
CA3865	1.0	1.0	0.9	IPF4608	complemer	unknown Function	orf19.6084	4608	CaRPL16A	PROTEIN S	RNA binding
CA3866	1.1	1.2	1.1	IPF4606	complemer	unknown Function	orf19.6082	4606	IPF4608	No significant S.c. match	
CA3867	1.1	1.2	1.1	PHR2	12560345..	pH-regulated protein 2	orf19.1350	4604	IPF4606	C-compour	molecular_function unknown
CA3868	1.1	1.2	1.0	CCT1	complemer	component of chapero	orf19.401	8832	CaPHR2	Lipid fatty-a	transferase activity
CA3869	1.1	2.1	1.1	IPF8831	12568747..	unknown function	orf19.400	8831	CaCCT1	PROTEIN I	chaperone activity
CA3870	1.4	1.5	1.1	YPK1	12570292..	ser/thr-specific protein	orf19.399	8830	IPF8831	No significant S.c. match	
CA3871	1.0	1.0	1.1	IPF8828	complemer	unknown function	orf19.398	8828	CaYPK1	CELL CYC	protein kinase activity
CA3872	1.1	1.0	0.9	MRPL28	12573281..	mitochondrial ribosoma	orf19.397	8826	IPF8828	No significant S.c. match	
CA3873	1.1	1.1	1.0	IPF8825	complemer	unknown function	orf19.396	8825	CaMRPL28	PROTEIN S	structural molecule activity
CA3874	4.6	4.0	2.6	ENO1	12575400..	Enolase I (2-phosphog	orf19.395	14429	IPF8825	UNCLASSI	molecular_function unknown
CA3875	1.2	1.0	1.0	IPF14430	complemer	putative kynureninase	orf19.8024	14430	CaENO1	C-compour	lyase activity
CA3876	1.1	1.0	1.1	APS3	12578650..	AP-3 complex subunit,	orf19.8023	7291	IPF14430	Amino acid	hydrolase activity
CA3877	1.1	1.1	1.1	CBP4	complemer	Ubiquinol--cytochrome	orf19.392	7290	CaAPS3	CELLULAF	molecular_function unknown
CA3878	1.0	0.9	1.0	IPF7289	12580573..	similar to Saccharomy	orf19.391	7289	CaCBP4	ENERGY "	molecular_function unknown
CA3879	1.2	1.1	1.2	CDC42	12583487..	Cell Division Control	orf19.390	7286	IPF7289	Lipid fatty-a	transcription regulator activity
CA3880	1.1	1.2	1.1	CAF16	complemer	ABC ATPase (by homo	orf19.388	7283	CaCDC42	CELL CYC	signal transducer activity
CA3881	0.8	0.8	1.0	GCR3	complemer	Large subunit of the nu	orf19.387	7281	CaCAF16	TRANSCR	transporter activity,hydrolase activity
CA3882	1.1	1.3	1.1	IPF7279	12589543..	putative cobalamin-dep	orf19.386	7279	CaGCR3	TRANSCR	RNA binding
CA3883	1.0	1.1	1.0	GCV2	12591452..	Glycine decarboxylase	orf19.8015	19789	IPF7279	UNCLASSI	transferase activity
CA3884	0.9	0.8	1.0	IPF6367	12595057..	unknown function	orf19.8014	6367	CaGCV2	Amino acid	oxidoreductase activity
CA3885	1.2	1.0	1.0	IPF19790	12598744..	unknown function	orf19.5095	19790	IPF6367	UNCLASSI	molecular_function unknown
CA3886	1.0	1.0	1.0	IPF12963	12605161..	ubiquitin-mediated prot	orf19.5094	12963	IPF19790	Lipid fatty-acid and isoprenoid metabolism	
CA3887	0.9	1.0	1.0	IPF12964	complemer	similar to Saccharomy	orf19.5093	12964	IPF12963	PROTEIN FATE [folding modification destination]	
CA3888	1.0	0.9	1.0	IPF12967	12608994..	unknown function	orf19.5092	12967	IPF12964	CELL CYC	protein binding
CA3889	1.0	0.9	1.0	TAD3	complemer	tRNA-specific adenosi	orf19.5090	12969	IPF12967	No significant S.c. match	
CA3890	0.9	0.9	1.1	TERT2	12610665..	telomerase reverse tra	orf19.5089	19791	CaTAD3	TRANSCR	hydrolase activity
CA3891	1.0	1.0	0.9	BUD6	complemer	bud site selection prote	orf19.5087	8707	CaTERT2	CELL CYC	DNA binding,nucleotidyltransferase activity
CA3892	1.0	1.1	1.0	PSE1	complemer	karyopherin-beta prote	orf19.5085	8712	CaBUD6	CELL FATI	protein binding
CA3893	0.9	0.9	0.9	FUN11	complemer	putative GTP-binding p	orf19.5083	8715	CaPSE1	CELLULAF	protein binding
CA3894	1.0	1.0	1.0	YIF2	12621274..	general translation fact	orf19.5081	8722	CaFUN11	UNCLASSI	molecular_function unknown
CA3895	1.1	1.3	1.2	CDR4	complemer	Multidrug resistance pr	orf19.5079	10349	CaYIF2	PROTEIN S	translation regulator activity
CA3896	0.9	0.8	0.8	IPF10339	complemer	unknown function	orf19.5077	10339	CaCDR4	Lipid fatty-acid and isoprenoid metabolism """"CELL RESCUE DEFENSE AND VIRULENCE ""REGULATION OF/INTERACTION WITH CE	
CA3897	1.9	1.7	1.2	PFY1	complemer	BINDS TO ACTIN	orf19.5076	16724	IPF10339	No significant S.c. match	
CA3898	1.1	1.0	1.0	UBA2	complemer	ubiquitin-activating -like	orf19.5074	10088	CaPFY1	CELL FATI	protein binding
CA3899	1.0	1.0	1.0	DPM1	complemer	dolichol-phosphate (be	orf19.5073	10089	CaUBA2	TRANSCRIPTION ""PROTEIN FATE [folding modification destination] ""SUBCELLULAR LOCALISATION	
CA3900	0.9	1.2	0.8	IPF10092	complemer	unknown function	orf19.5071	10092	CaDPM1	C-compour	transferase activity
CA3901	1.1	1.0	1.0	IPF3282.3E	12643207..	hexose transporter, 3-prime end (b		14892	IPF10092	UNCLASSI	molecular_function unknown
CA3902	1.0	1.0	1.0	YVH1	12643889..	protein tyrosine phospl	orf19.4401	14891	IPF3282.3e	C-compound and carbohydrate metabolism CELLULAR TRANSPORT AND TRANSPORT MECHANISMS SUBCELLULAR LOCALISATIO	
CA3903	0.9	1.0	1.0	IPF14890	complemer	unknown function	orf19.4400	14890	CaYVH1	CELL CYC	protein phosphatase activity
CA3904	1.1	1.0	0.9	IPF14888	complemer	unknown function	orf19.1187	14888	IPF14890	No significant S.c. match	
CA3905	0.9	1.0	1.0	IPF19792	complemer	unknown function	orf19.1187	19792	IPF14888	PROTEIN S	molecular_function unknown
CA3906	1.1	1.1	1.0	IPF20149	complemer	unknown function	orf19.1187	20149	IPF19792	UNCLASSI	molecular_function unknown
CA3907	1.0	1.0	1.0	IPF3304	12651099..	similar to Saccharomy	orf19.1187	3304	IPF20149	ENERGY	molecular_function unknown
CA3908	1.0	1.0	0.9	IPF3301	complemer	unknown function	orf19.4394	3301	IPF3304	SUBCELLU	protein binding
CA3909	0.3	0.9	0.1	CIT1.EXON	complemer	Citrate synthase, exon	orf19.4393	3299	IPF3301	C-compound and carbohydrate metabolism ""PROTEIN FATE [folding modification destination] ""	
CA3910	1.1	0.9	1.0	IPF3293	complemer	unknown function	orf19.4392	3293	CaCIT1.ex	C-compour	transferase activity
CA3911	0.9	1.0	0.9	IPF3292	complemer	unknown function	orf19.4391	3292	IPF3293	UNCLASSI	molecular_function unknown
CA3912	1.0	0.9	1.0	IPF3288	complemer	unknown function	orf19.4390	3288	IPF3292	UNCLASSIFIED PROTEINS	
CA3913	0.9	1.0	1.0	IPF3283	complemer	unknown function	orf19.4388	3283	IPF3288	No significant S.c. match	
CA3914	1.1	1.0	0.9	IPF3282	complemer	hexose transporter (by	orf19.4386	3282	IPF3283	TRANSCR	transcription regulator activity
CA3915	1.0	1.0	1.0	IPF3277	complemer	unknown function	orf19.4384	3277	IPF3282	C-compound and carbohydrate metabolism CELLULAR TRANSPORT AND TRANSPORT MECHANISMS SUBCELLULAR LOCALISATIO	
CA3916	0.9	0.8	0.9	IPF3274	complemer	Unknown function	orf19.4383	3274	IPF3277	No significant S.c. match	
CA3917	1.1	1.1	1.2	IPF3273	complemer	similar to Saccharomy	orf19.4382	3273	IPF3274	UNCLASSI	molecular_function unknown
CA3918	1.0	0.9	1.0	VTC2	complemer	putative polyphosphate	orf19.4381	3271	IPF3273	CELLULAF	protein binding
CA3919	0.9	1.0	1.0	IFC5	complemer	unknown function	orf19.5121	9728	CaVTC2	Phosphate	molecular_function unknown
CA3920	1.1	1.0	1.0	SDS24	complemer	Similar to S. cerevisiae	orf19.5118	6359	CaIFC5	TRANSPORT FACILITATION	
CA3921	1.5	2.8	1.9	OLE1	12697174..	Stearoyl-CoA desatura	orf19.5117	6353	CaSDS24	PROTEIN I	molecular_function unknown
CA3922	0.9	0.9	1.0	GRD19	12700546..	Probable golgi membra	orf19.5114	6349	CaOLE1	Lipid fatty-a	oxidoreductase activity
CA3923	0.6	1.4	0.5	ADH2	12702654..	alcohol dehydrogenase	orf19.5113	16784	CaGRD19	PROTEIN I	protein binding
CA3924	1.0	1.1	1.5	TKL1	complemer	transketolase 1	orf19.5112	13260	CaADH2	C-compour	oxidoreductase activity
CA3925	0.9	1.0	0.9	IPF13257	complemer	unknown function	orf19.5110	13257	CaTKL1	Amino acid	transferase activity
CA3926	1.0	1.0	0.9	IPF16198	complemer	possible regulatory pro	orf19.5107	16198	IPF13257	No significant S.c. match	
CA3927	0.9	0.9	0.9	DIP2	12713649..	beta transducin	orf19.5106	14262	IPF16198	TRANSCR	RNA binding
CA3928	0.6	0.5	0.8	GAL11	12719361..	DNA-directed RNA pol	orf19.5105	12729	CaDIP2	CELL CYC	RNA binding
CA3930	0.9	1.0	1.0	LPT1.EXOI	12723309..	protein-tyrosine-phosp	orf19.5104	12722	CaGAL11	C-compour	transcription regulator activity
CA3931	0.9	0.9	0.8	IPF12719	12725059..	unknown function	orf19.5103	12719	CaLPT1.ex	CLASSIFIC	protein phosphatase activity
CA3932	0.9	0.9	1.0	IPF20150	complemer	unknown function	orf19.9561	20150	IPF12719	ENERGY	
CA3933	0.9	0.8	0.9	IPF18281	12730007..	similar to Saccharomy	orf19.9560	18281	IPF20150	No significant S.c. match	
CA3934	1.0	1.0	0.9	IPF17119	12731697..	unknown function	orf19.2008	17119	IPF18281	SUBCELLU	protein binding
CA3935	1.1	1.0	1.1	IPF14688	complemer	unknown function	orf19.2007	14688	IPF17119	UNCLASSI	molecular_function unknown
CA3936	1.0	1.0	1.1	COX17	12736721..	cysteine-rich cytoplasmic protein(b		14687	IPF14688	CELLULAF	molecular_function unknown

CA3937	1.0	1.1	1.1	IPF14686	complemer	unknown function	orf19.9557	14686	CaCOX17	ENERGY "	transporter activity
CA3938	0.5	0.5	0.4	IPF4764	complemer	unknown Function	orf19.9556	4764	IPF14686	UNCLASSI	molecular_function unknown
CA3939	1.0	1.0	1.0	HNM1	complemer	Choline permease (by	orf19.9554	4757	IPF4764	C-compour	protein phosphatase activity
CA3940	0.9	0.9	0.8	NIC96	12748259..	nuclear pore protein (b	orf19.9553	4755	CaHNM1	CELLULAR TRANSPORT AND TRANSPORT MECHANISMS SUBCELLULAR LOCALISATION TRANSPORT FACILITATION	
CA3941	0.9	1.0	0.9	IPF4754	12751373..	unknown Function	orf19.2001	4754	CaNIC96	CELLULAF	structural molecule activity
CA3942	0.9	0.9	0.9	CHL1	complemer	protein of the DEAH bo	orf19.2000	4753	IPF4754	No significant S.c. match	
CA3943	0.9	1.0	1.0	IPF4751	12754865..	unknown Function	orf19.1999	4751	CaCHL1	CELL CYC	DNA binding,helicase activity
CA3944	1.0	0.8	0.9	IPF4750	12755828..	unknown Function	orf19.1998	4750	IPF4751	No significant S.c. match	
CA3945	1.0	1.0	1.0	CHA12	12758897..	L-serine/L-threonine de	orf19.9548	4749	IPF4750	CELL CYCLE AND DNA PROCESSING	
CA3946	1.0	1.0	1.0	MNN2	complemer	Golgi alpha-1,2-mannc	orf19.9547	4742	CaCHA12	Amino acid metabolism	
CA3947	1.0	1.0	1.0	IPF14768	12766735..	unknown function	orf19.1994	14768	CaMNN2	CELL FATE	
CA3948	1.1	1.0	1.1	RPN9	12769144..	26S proteasome regula	orf19.1993	14766	IPF14768	UNCLASSIFIED PROTEINS	
CA3949	0.9	1.0	1.1	SIR21	complemer	regulatory protein (by h	orf19.9544	17303	CaRPN9	PROTEIN I	peptidase activity
CA3950	1.0	0.9	1.0	PTM1	12773621..	Possibly involved in the	orf19.9542	19793	CaSIR21	TRANSCRIPTION CELL FATE	
CA3951	1.1	0.9	0.9	VAC7.3	12796222..	Vacuolar protein, 3-prin	orf19.1409	6717	CaPTM1	ENERGY	molecular_function unknown
CA3952	1.1	1.0	1.0	IPF6716	complemer	Phenylacetate 2-hydro	orf19.1411	6716	CaVAC7.3	PROTEIN I	enzyme regulator activity
CA3953	0.9	0.9	0.9	IPF6714	complement(12800838..12801470		orf19.1412	6714	IPF6716	Lipid fatty-acid and isoprenoid metabolism """"CELL RESCUE DEFENSE AND VIRULENCE ""SUBCELLULAR LOCALISATION	
CA3954	0.9	1.0	1.1	YFH1	12801702..	Regulates mitochondri	orf19.1413	6713	IPF6714	No significant S.c. match	
CA3955	1.2	1.3	1.2	IPF6712.5f	12802958..	unknown function, 5-pr	orf19.1414	6712	CaYFH1	REGULATI	enzyme regulator activity
CA3956	1.1	0.9	1.1	IPF6712.3f	12803598..	unknown function, 3-prime end		6710	IPF6712.5f	UNCLASSIFIED PROTEINS	
CA3957	1.3	1.3	1.5	RBT2	complemer	Repressed by TUP1 pr	orf19.1415	6709	IPF6712.3f	No significant S.c. match	
CA3958	0.9	1.0	1.0	COX11	complemer	cytochrome-c oxidase	orf19.1416	11240	CaRBT2	REGULATION OF/INTERACTION WITH CELLULAR ENVIRONMENT Other virulence attributes	
CA3959	1.1	0.9	1.0	IPF11236.3	complemer	similar to Saccharomy	orf19.1418	11238	CaCOX11	Metabolism	molecular_function unknown
CA3960	1.0	0.9	1.0	IPF11236.5	complemer	similar to Saccharomy	orf19.1419	11236	IPF11236.3	CELLULAR TRANSPORT AND TRANSPORT MECHANISMS SUBCELLULAR LOCALISATION	
CA3961	0.9	0.9	1.0	IPF11235	12814339..	unknown function	orf19.1420	11235	IPF11236.5	CELLULAF	protein binding
CA3962	0.9	1.0	0.9	IPF11234	complemer	similar to saccharomyc	orf19.1421	11234	IPF11235	UNCLASSI	molecular_function unknown
CA3963	1.0	1.1	1.0	IPF11233	12816247..	similar to Saccharomy	orf19.1422	11233	IPF11234	Nitrogen ar	hydrolase activity
CA3964	1.1	1.0	1.1	IPF9370	complemer	unknown function	orf19.1424	9370	IPF11233	CONTROL	hydrolase activity
CA3965	1.2	1.3	1.2	IPF9375	12822546..	unknown function	orf19.1426	9375	IPF9370	UNCLASSI	molecular_function unknown
CA3966	1.0	1.0	1.0	IPF9376	complemer	unknown function	orf19.1427	9376	IPF9375	UNCLASSIFIED PROTEINS	
CA3967	0.9	1.0	1.0	IPF9377	complemer	unknown function	orf19.1428	9377	IPF9376	TRANSPO	transporter activity
CA3968	1.0	1.0	1.1	IPF9378	12827859..	similar to Saccharomy	orf19.1429	9378	IPF9377	No significant S.c. match	
CA3969	1.0	1.0	1.0	IPF9379	12829925..	unknown function	orf19.1430	9379	IPF9378	CELL CYC	molecular_function unknown
CA3970	1.1	1.0	1.0	IPF6497	complemer	unknown function	orf19.1542	6497	IPF9379	No significant S.c. match	
CA3971	1.1	1.1	1.1	IPF6498	complemer	unknown function	orf19.1543	6498	IPF6497	C-compour	DNA binding
CA3972	1.1	1.0	1.1	IPF6504	12839665..	unknown function	orf19.9118	6504	IPF6498	No significant S.c. match	
CA3973	1.0	1.0	1.0	IPF6505	complemer	unknown function	orf19.1545	6505	IPF6504	UNCLASSI	molecular_function unknown
CA3974	0.9	0.9	1.0	IPF6507	12841350..	unknown function	orf19.1546	6507	IPF6505	UNCLASSI	molecular_function unknown
CA3975	1.0	1.0	1.0	IPF20152	complemer	unknown function	orf19.1547	20152	IPF6507	UNCLASSI	molecular_function unknown
CA3976	0.9	0.9	0.9	SNU23	12843206..	RNA binding zinc finge	orf19.1548	15310	IPF20152	UNCLASSI	molecular_function unknown
CA3977	1.0	0.9	1.0	IPF20153	12844359..	unknown function	orf19.1549	20153	CaSNU23	TRANSCR	RNA binding
CA3978	1.2	1.1	1.1	CPR3	complemer	cyclophilin (peptidylprc	orf19.1552	20154	IPF20153	No significant S.c. match	
CA3979	1.2	1.3	1.1	ENT3.3F	complemer	putative endocytosis ai	orf19.1553	7127	CaCPR3	PROTEIN FATE [folding modification destination] ""SUBCELLULAR LOCALISATION	
CA3982	1.0	1.0	1.0	SAC3	complemer	Leucine permease trar	orf19.9129	7130	CaENT3.3f	SUBCELLU	protein binding
CA3983	1.0	0.9	0.9	IPF7133.3	complemer	unknown function, 3-pr	orf19.9130	7133	CaSAC3	Amino acid	protein binding
CA3984	1.2	1.2	1.1	HOM2	complemer	Aspartate-semialdehyc	orf19.9132	7136	IPF7133.3	UNCLASSI	molecular_function unknown
CA3985	1.1	1.0	1.0	POB3	12855324..	Binds DNA polymerase	orf19.1560	7138	CaHOM2	Amino acid	oxidoreductase activity
CA3986	1.5	1.6	1.2	IPF7141	12858766..	unknown function	orf19.1562	7141	CaPOB3	CELL CYC	DNA binding
CA3987	1.0	1.0	1.0	ECM3	12860734..	Involved in cell wall bic	orf19.1563	7144	IPF7141	No significant S.c. match	
CA3988	2.8	1.6	1.4	IPF7145	12862774..	unknown function	orf19.1564	7145	CaECM3	CONTROL	hydrolase activity
CA3989	1.0	1.1	1.0	IPF7147	complemer	unknown function	orf19.1565	7147	IPF7145	UNCLASSI	molecular_function unknown
CA3990	0.9	1.0	0.9	IPF14782	12865488..	beta-transducin (by ho	orf19.1566	14782	IPF7147	UNCLASSIFIED PROTEINS	
CA3991	0.9	0.9	1.0	VAM6.5F	12868640..	Vacuolar carboxypepti	orf19.1567	14783	IPF14782	CLASSIFIC	molecular_function unknown
CA3992	1.0	1.1	1.0	VAM6.3F	12870299..	Vacuolar carboxypepti	orf19.1568	19794	CaVAM6.5	No significant S.c. match	
CA3993	0.9	1.0	1.0	IPF8275	complemer	unknown function	orf19.9142	8275	CaVAM6.3	PROTEIN I	enzyme regulator activity
CA3994	0.9	1.0	0.9	ERG7	12875947..	lanosterol synthase	orf19.9143	8270	IPF8275	UNCLASSI	molecular_function unknown
CA3995	0.9	0.9	0.9	IPF11615.3	12879487..	RNA-binding protein (by homology		10438	CaERG7	Lipid fatty-a	isomerase activity
CA3996	0.9	0.6	1.1	IPF10440	12879925..	unknown function	orf19.1643	10440	IPF11615.3	CLASSIFICATION NOT YET CLEAR-CUT	
CA3997	1.0	1.0	1.0	LOC1	complemer	putative double-strand	orf19.1642	10442	IPF10440	UNCLASSI	peptidase activity
CA3998	1.0	1.0	1.0	IPF10443	12882405..	formamidase-like prote	orf19.1641	10443	CaLOC1	UNCLASSI	RNA binding
CA3999	1.0	1.1	1.1	IPF10447	complemer	unknown function	orf19.1637	10447	IPF10443	No significant S.c. match	
CA4000	1.1	1.0	1.1	IPF4262	12890326..	similar to Saccharomy	orf19.1636	4262	IPF10447	No significant S.c. match	
CA4001	2.4	1.4	1.8	RPL12	12892036..	ribosomal protein	orf19.1635	4260	IPF4262	REGULATI	enzyme regulator activity
CA4002	1.0	1.0	1.0	IPF4258	12893635..	unknown function	orf19.1634	4258	CaRPL12	PROTEIN S	structural molecule activity
CA4003	1.0	1.0	1.0	IPF4257	12895133..	unknown function	orf19.1633	4257	IPF4258	Lipid fatty-acid and isoprenoid metabolism	
CA4004	0.9	0.9	1.2	IPF4256	complemer	unknown function	orf19.1632	4256	IPF4257	UNCLASSI	RNA binding
CA4005	1.0	0.9	1.0	ERG6	complemer	sterol transmethylase	orf19.1631	4255	IPF4256	UNCLASSIFIED PROTEINS	
CA4006	1.0	1.0	1.0	IPF4253	complemer	unknown function	orf19.1630	4253	CaERG6	Lipid fatty-a	transferase activity
CA4007	1.0	1.2	1.1	LAP41	complemer	aminopeptidase yscI p	orf19.1628	4250	IPF4253	TRANSCR	molecular_function unknown
CA4008	1.0	1.0	1.0	DYS1	complemer	deoxyhypusine syntha	orf19.1626	4248	CaLAP41	PROTEIN I	peptidase activity
CA4009	1.0	1.0	1.0	IPF4247	complemer	unknown function	orf19.1625	4247	CaDYS1	Amino acid	transferase activity
CA4010	1.0	0.9	1.0	MAK10.3	complemer	glucose-repressible pro	orf19.1624	4245	IPF4247	No significant S.c. match	

CA4011	1.0	1.0	1.0	IPF4240	complemer	similar to Saccharomy	orf19.1622	4240	CaMAK10.	ENERGY	transferase activity
CA4012	1.1	1.0	1.0	GPA2	complemer	nucleotide-binding reg	orf19.1621	4239	IPF4240	CELL CYC	molecular_function unknown
CA4013	1.0	1.0	1.1	IPF4234	complemer	unknown function	orf19.1620	4234	CaGPA2	CELLULAF	hydrolase activity
CA4014	1.0	1.0	1.0	CTK1	complemer	probable cell division p	orf19.1619	4233	IPF4234	No significant S.c. match	
CA4015	1.0	1.3	1.1	IPF12268	12926743..	unknown function		12268	CaCTK1	TRANSCR	protein kinase activity
CA4016	1.1	1.3	1.3	GFA1	complemer	glutamine:fructose-6-p	orf19.1618	12267	IPF12268	No significant S.c. match	
CA4017	0.9	1.1	1.0	PBN1	12937376..	protease by homology	orf19.3447	5328	CaGFA1	C-compour	transferase activity
CA4018	1.0	1.0	1.0	MOG1	complemer	Ran-Binding Protein by	orf19.3446	5327	CaPBN1	PROTEIN I	molecular_function unknown
CA4019	0.9	1.1	1.0	HOC1	12939834..	GLYCOSYLTRANSFE	orf19.3445	5326	CaMOG1	PROTEIN I	protein binding
CA4020	1.0	1.0	1.1	IPF5324	12942128..	putative transporter (by	orf19.3444	5324	CaHOC1	C-compour	transferase activity
CA4021	1.0	1.1	1.0	EBP2	complemer	NADPH dehydrogenas	orf19.3442	5322	IPF5324	CELL RESCUE DEFENSE AND VIRULENCE ""TRANSPORT FACILITATION	
CA4022	1.1	1.0	1.0	FRP6	complemer	member of the FRP fa	orf19.3441	5321	CaEBP2	ENERGY	
CA4023	1.1	1.1	1.1	FRP5	complemer	member of the FRP fa	orf19.3440	7185	CaFRP6	C-compound and carbohydrate metabolism	
CA4024	0.9	0.9	1.0	IPF7182	complemer	unknown function	orf19.3439	7182	CaFRP5	C-compound and carbohydrate metabolism	
CA4025	1.1	1.0	1.0	SCJ1	complemer	Mitochondrial and ER i	orf19.3438	7178	IPF7182	No significant S.c. match	
CA4026	0.9	0.9	0.9	IPF7175	12957963..	unknown function	orf19.3437	7175	CaSCJ1	PROTEIN I	chaperone activity
CA4027	1.0	1.0	0.9	IPF7174	12960250..	unknown function	orf19.3436	7174	IPF7175	No significant S.c. match	
CA4028	1.0	1.0	1.0	IPF7171.3f	complemer	unknown function, 3-pr	orf19.3435	7173	IPF7174	CELL FATE SUBCELLULAR LOCALISATION PROTEIN ACTIVITY REGULATION	
CA4029	1.1	0.9	1.0	IPF7171.5f	complemer	unknown function, 5-pr	orf19.3434	7171	IPF7171.3f	No significant S.c. match	
CA4030	0.9	0.9	0.9	EBP4	12968102..	NADPH dehydrogenas	orf19.3433	19548	IPF7171.5f	TRANSCR	molecular_function unknown
CA4031	0.9	1.0	0.9	IPF11077	12970024..	membrane transporter	orf19.3432	11077	CaEBP4	ENERGY	transporter activity
CA4032	1.1	0.9	1.0	IPF11081	12975776..	unknown function		11081	IPF11077	CELL RESCUE DEFENSE AND VIRULENCE ""TRANSPORT FACILITATION	
CA4033	0.9	0.9	1.0	IPF19795	complemer	similar to Saccharomy	orf19.3431	19795	IPF11081	No significant S.c. match	
CA4034	1.0	1.0	0.9	HSP31	12983964..	heat shock protein (by	orf19.1114	12039	IPF19795	CELL CYC	nucleotidyltransferase activity
CA4035	1.0	1.0	1.0	IPF12040	12985492..	unknown function	orf19.3665	12040	CaHSP31	CELL RESCUE DEFENSE AND VIRULENCE ""REGULATION OF/INTERACTION WITH CELLULAR ENVIRONMENT SUBCELLULAR LC	
CA4036	1.0	1.0	1.0	IPF12042	complemer	unknown function	orf19.3666	12042	IPF12040	UNCLASSI	molecular_function unknown
CA4037	1.0	1.1	1.1	KIP31	12988346..	kinesin-related protein	orf19.3667	12044	IPF12042	UNCLASSI	molecular_function unknown
CA4038	1.1	1.2	1.0	HGT12	complemer	hexose transporter	orf19.3668	5315	CaKIP31	CELL CYCLE AND DNA PROCESSING CELLULAR TRANSPORT AND TRANSPORT MECHANISMS SUBCELLULAR LOCALISATION	
CA4039	1.0	1.0	1.0	SKS1	complemer	serine/threonine kinase	orf19.3669	5304	CaHGT12	C-compound and carbohydrate metabolism TRANSPORT FACILITATION	
CA4040	1.0	1.0	1.0	GAL1	complemer	galactokinase	orf19.3670	5296	CaSKS1	C-compour	protein kinase activity
CA4041	1.0	1.0	1.0	GAL10	13008284..	UDP-glucose 4-epimer	orf19.3672	5294	CaGAL1	C-compour	protein binding
CA4042	0.9	0.8	0.9	TRS23	13010516..	targeting and fusion of	orf19.3673	5292	CaGAL10	C-compour	molecular_function unknown
CA4043	0.9	0.9	0.9	IPF5291	complemer	UDP-glucose 4-epimer	orf19.3674	5291	CaTRS23	CELLULAF	molecular_function unknown
CA4044	1.0	1.0	1.0	GAL7	13012925..	UDP-glucose-hexose-1	orf19.3675	9203	IPF5291	C-compound and carbohydrate metabolism SUBCELLULAR LOCALISATION	
CA4045	1.1	1.1	1.1	ABP140	13014486..	putative methyltransfer	orf19.3676	9204	CaGAL7	C-compound and carbohydrate metabolism SUBCELLULAR LOCALISATION	
CA4046	1.0	0.9	1.0	IPF9205	complemer	similar to Saccharomy	orf19.3677	9205	CaABP140	UNCLASSIFIED PROTEINS	
CA4047	1.0	1.1	1.1	IPF9206	13017190..	unknown function	orf19.3678	9206	IPF9205	PROTEIN I	molecular_function unknown
CA4048	1.0	0.9	1.1	IPF9207	complemer	unknown function	orf19.1116	9207	IPF9206	No significant S.c. match	
CA4049	1.0	1.0	1.1	IPF10482.E	13024198..	unknown function, exo	orf19.8054	10482	IPF9207	UNCLASSI	molecular_function unknown
CA4050	1.0	1.0	1.0	IPF10482.E	13024939..	unknown function, exo	orf19.425	20007	IPF10482.e	CELL RESCUE DEFENSE AND VIRULENCE ""REGULATION OF/INTERACTION WITH CELLULAR ENVIRONMENT SUBCELLULAR LC	
CA4051	1.0	1.0	1.0	IPF10231.E	13025769..	similar to Saccharomy	orf19.426	10233	IPF10482.e	UNCLASSI	molecular_function unknown
CA4052	0.9	1.0	0.8	IPF10231.E	13027896..	similar to Saccharomy	orf19.427	10231	IPF10231.e	CELL CYCLE AND DNA PROCESSING TRANSCRIPTION SUBCELLULAR LOCALISATION	
CA4053	1.0	1.0	1.0	IKS1	13033171..	PROBABLE SERINE/T	orf19.428	7725	IPF10231.e	TRANSCR	molecular_function unknown
CA4054	0.9	0.9	0.9	TRF4	13035598..	Topoisomerase I-relate	orf19.429	7724	CaIKS1	UNCLASSI	molecular_function unknown
CA4055	1.0	1.0	1.1	YPT522	complemer	GTP-binding protein of	orf19.430	7723	CaTRF4	CELL CYC	nucleotidyltransferase activity
CA4056	1.0	0.9	0.9	IPF7721	13039034..	unknown function	orf19.431	7721	CaYPT522	CELLULAR TRANSPORT AND TRANSPORT MECHANISMS SUBCELLULAR LOCALISATION	
CA4057	1.1	1.0	1.1	IPF7719	13041664..	unknown function	orf19.432	7719	IPF7721	Amino acid metabolism TRANSCRIPTION SUBCELLULAR LOCALISATION UNCLASSIFIED PROTEINS	
CA4058	1.0	1.0	1.1	IPF7717	13043295..	unknown function	orf19.8063	7717	IPF7719	UNCLASSI	protein binding
CA4059	1.0	1.2	0.9	PRD1	13045920..	Proteinase (by homolo	orf19.8064	5940	IPF7717	C-compour	molecular_function unknown
CA4060	1.0	1.1	1.0	GRS1	complemer	glycine-tRNA ligase (by	orf19.437	5937	CaPRD1	PROTEIN I	peptidase activity
CA4061	1.1	1.1	1.0	IPF5935	complemer	Unknown function	orf19.438	5935	CaGRS1	PROTEIN S	ligase activity
CA4062	1.0	1.0	1.0	IPF5933	13052447..	similar to Saccharomy	orf19.439	5933	IPF5935	UNCLASSI	molecular_function unknown
CA4063	1.0	0.9	1.1	SDH11	13053735..	Succinate dehydrogen	orf19.440	5931	IPF5933	CELL CYC	transcription regulator activity
CA4064	0.8	0.8	0.9	RPT1	13055941..	26S PROTEASE REG	orf19.441	5928	CaSDH11	C-compour	oxidoreductase activity
CA4065	1.0	1.0	1.0	RPC25	complemer	DNA-direcred RNA pol	orf19.443	5926	CaRPT1	CELL CYC	peptidase activity
CA4066	1.0	1.0	1.0	IPF5925	13058238..	RNA-binding protein (b	orf19.444	5925	CaRPC25	TRANSCR	nucleotidyltransferase activity
CA4067	1.0	0.9	1.0	IPF5924	13058888..	unknown function	orf19.445	5924	IPF5925	TRANSCRIPTION SUBCELLULAR LOCALISATION	
CA4068	0.7	0.9	0.8	IPF5922	13061155..	unknown function	orf19.446	5922	IPF5924	UNCLASSI	molecular_function unknown
CA4069				MHP1	13066468..13068705				IPF5922	No significant S.c. match	
CA4070	1.0	1.1	1.0	IPF2562	complemer	unknown function	orf19.6623	2562			
CA4071	0.9	1.1	0.9	IPF2561	complemer	unknown function	orf19.6624	2561	IPF2562	UNCLASSI	molecular_function unknown
CA4072	0.8	0.7	0.9	IPF2560	13071318..	unknown function	orf19.6625	2560	IPF2561	CELLULAF	enzyme regulator activity
CA4073	0.9	0.9	1.0	IPF2559.3	complemer	unknown function, 3-pr	orf19.6626	2559	IPF2560	CELL CYC	molecular_function unknown
CA4074	1.0	1.0	1.0	IPF2557	complemer	unknown function	orf19.6627	2557	IPF2559.3	No significant S.c. match	
CA4075	1.0	0.9	1.0	IPF2555	complemer	unknown function	orf19.6628	2555	IPF2557	UNCLASSI	molecular_function unknown
CA4076	1.0	0.9	1.0	IPF2542	13075857..	putative neutral sphing	orf19.6629	2542	IPF2555	SUBCELLULAR LOCALISATION	
CA4077	1.0	0.9	1.1	ACO2	13079616..	aconitate hydratase (by	orf19.6632	2530	IPF2542	Lipid fatty-a	hydrolase activity
CA4078	1.0	1.2	1.0	VMA2	complemer	H+-transporting ATPas	orf19.6634	2528	CaACO2	ENERGY	lyase activity
CA4079	1.0	1.0	0.9	IPF2527	complemer	unknown function	orf19.6635	2527	CaVMA2	CELLULAF	transporter activity
CA4080	0.9	1.1	1.0	IPF2524	13085887..	unknown function	orf19.6636	2524	IPF2527	No significant S.c. match	
CA4081	1.1	1.1	1.0	IPF2523	complemer	unknown function	orf19.6637	2523	IPF2524	UNCLASSI	molecular_function unknown
CA4082	1.0	0.9	1.0	PTC4	13089027..	ser/thr protein phospha	orf19.6638	15571	IPF2523	No significant S.c. match	

CA4083	1.1	1.0	0.9	IPF5018	13091134..	unknown function	orf19.6639	5018	CaPTC4	CLASSIFIC	protein phosphatase activity
CA4084	1.8	2.3	1.4	TPS1	complemer	TREHALOSE-6-PHOS	orf19.6640	5016	IPF5018	UNCLASSIFIED PROTEINS	
CA4085	1.1	1.1	1.0	IPF5015	13095177..	unknown function	orf19.6641	5015	CaTPS1	C-compour	transferase activity
CA4086	1.1	1.1	1.0	IPF5014	13096360..	unknown function	orf19.6642	5014	IPF5015	No significant S.c. match	
CA4087	1.1	1.0	1.1	IPF5013	complemer	similar to Saccharomy	orf19.6643	5013	IPF5014	CELLULAF	molecular_function unknown
CA4088	1.8	1.3	1.3	HMO1	13099520..	High-mobility protein 1	orf19.6645	5011	IPF5013	Lipid fatty-a	transferase activity
CA4089	1.0	1.1	0.9	IPF5009	complemer	unknown function	orf19.6648	5009	CaHMO1	SUBCELLU	DNA binding,transcription regulator activity
CA4090	1.0	0.9	0.9	BRF1	complemer	TFIIIB subunit	orf19.6649	5007	IPF5009	CELL CYC	molecular_function unknown
CA4091	0.9	0.9	1.0	IPF5005	13104923..	unknown function	orf19.6650	5005	CaBRF1	TRANSCR	transcription regulator activity
CA4092	1.0	1.1	1.0	DBP8	complemer	DEAD box protein ATF	orf19.6652	5004	IPF5005	No significant S.c. match	
CA4093	1.0	1.0	1.0	IPF5002	13107433..	GTP binding protein (b	orf19.6653	5002	CaDBP8	TRANSCR	RNA binding,helicase activity
CA4094	1.0	0.9	1.0	IPF4004	13114300..	unknown function	orf19.658	4004	IPF5002	CLASSIFIC	molecular_function unknown
CA4095	0.9	0.8	0.9	IPF4002	complemer	unknown function	orf19.660	4002	IPF4004	UNCLASSI	molecular_function unknown
CA4096	1.0	1.0	1.0	KRR1	complemer	involved in cell division	orf19.661	4000	IPF4002	No significant S.c. match	
CA4097	1.0	0.9	1.1	GIN4	13124277..	ser/thr protein kinase (	orf19.663	3994	CaKRR1	SUBCELLU	molecular_function unknown
CA4098	1.0	1.0	1.0	IPF3988	complemer	unknown function	orf19.664	3988	CaGIN4	CELL CYC	protein kinase activity
CA4099	0.9	0.8	1.0	IPF3986	complemer	unknown function	orf19.665	3986	IPF3988	CELLULAR TRANSPORT AND TRANSPORT MECHANISMS CELL FATE CONTROL OF CELLULAR ORGANIZATION SUBCELLULAR	
CA4100	0.9	1.0	1.0	IPF3985	13131074..	unknown function	orf19.666	3985	IPF3986	UNCLASSI	molecular_function unknown
CA4101	1.0	0.9	1.0	IPF3984	13132115..	unknown function	orf19.667	3984	IPF3985	CELL CYC	protein binding
CA4102	1.9	1.7	1.2	RPL37B	complemer	Ribosomal protein		20156	IPF3984	No significant S.c. match	
CA4103	1.0	0.9	1.1	IPF3980	13134897..	unknown function	orf19.668	3980	CaRPL37B	PROTEIN S	structural molecule activity
CA4104	0.9	1.0	1.0	IPF20157	complemer	unknown function	orf19.669	20157	IPF3980	UNCLASSI	DNA binding,transcription regulator activity
CA4105	1.2	1.2	1.1	SMT3	13138818..	Ubiquitin-like protein (b	orf19.670	20158	IPF20157	UNCLASSI	molecular_function unknown
CA4107	1.0	0.9	0.9	IPF3970	13140769..	unknown function	orf19.671	3970	CaSMT3	PROTEIN FATE [folding modification destination]	
CA4108	0.9	0.9	0.9	IPF3968	13145290..	similar to Saccharomy	orf19.672	3968	IPF3970	UNCLASSI	molecular_function unknown
CA4109	1.0	1.0	0.9	IPF3967	complemer	unknown function	orf19.673	3967	IPF3968	TRANSCR	RNA binding,helicase activity
CA4110	1.0	1.0	1.0	IPF3965	complemer	unknown function	orf19.674	3965	IPF3967	ENERGY	
CA4111	1.3	1.5	1.4	IPF3964	complemer	unknown function	orf19.675	3964	IPF3965	UNCLASSI	molecular_function unknown
CA4112	1.0	0.9	1.0	BIM1	complemer	microtubule-binding pr	orf19.676	7699	IPF3964	No significant S.c. match	
CA4113	1.0	1.5	1.1	CHO1	13154895..	Phosphatidylserine syr	orf19.677	7703	CaBIM1	CELL CYC	structural molecule activity
CA4114	0.9	1.0	1.1	IPF7704	complemer	unknown function	orf19.679	7704	CaCHO1	Lipid fatty-a	transferase activity
CA4115	1.1	1.0	1.0	IPF7706	13156638..	putative plasma memb	orf19.680	7706	IPF7704	No significant S.c. match	
CA4116	1.0	1.0	0.9	IDI1	13165199..	isopentenyl-diphospha	orf19.2775	11947	IPF7706	UNCLASSI	molecular_function unknown
CA4117	0.9	0.9	0.9	LAB5	13166539..	lipoic acid synthase (b	orf19.2774	11949	CaIDI1	Lipid fatty-a	isomerase activity
CA4118	1.1	1.1	1.1	IPF11952	13168575..	similar to Saccharomy	orf19.2772	11952	CaLAB5	Metabolism of vitamins cofactors and prosthetic groups ""SUBCELLULAR LOCALISATION	
CA4119	1.0	0.9	1.0	BEM3	13172328..	GTPase-activating pro	orf19.2771	7085	IPF11952	CLASSIFIC	hydrolase activity
CA4120	1.4	1.4	1.3	SOD1.3	complemer	Cu,Zn-superoxide dismutase, 3-pr		7082	CaBEM3	CELL FATE	signal transducer activity
CA4121	1.0	1.0	0.9	IPF7081	complemer	unknown function	orf19.2770	7081	CaSOD1.3	CELL RES	oxidoreductase activity
CA4122	1.0	1.2	1.1	PBI2	13178853..	proteinase B inhibitor 2	orf19.2769	7079	IPF7081	No significant S.c. match	
CA4123	1.3	0.8	1.9	AMS1	13179983..	alpha-mannosidase (b	orf19.2768	7076	CaPBI2	PROTEIN I	enzyme regulator activity
CA4124	3.5	2.1	4.8	IPF20008	complemer	unknown function	orf19.2767	20008	CaAMS1	C-compour	hydrolase activity
CA4125	3.3	1.8	4.3	IPF20161	13195669..	unknown function	orf19.1028	20161	IPF20008	No significant S.c. match	
CA4126	1.0	1.0	1.0	IPF6631	complemer	unknown function	orf19.2763	6631	IPF20161	No significant S.c. match	
CA4127	2.3	2.0	4.0	IPF6629	complemer	unknown function	orf19.2762	6629	IPF6631	UNCLASSIFIED PROTEINS	
CA4128	1.1	1.0	1.0	IPF6876	13208708..	unknown function	orf19.1172	6876	IPF6629	CELL RES	oxidoreductase activity
CA4129	0.9	1.0	0.9	IPF6878	complemer	unknown function	orf19.1172	6878	IPF6876	No significant S.c. match	
CA4130	0.9	0.9	1.2	IPF20009	13216409..	Unknown function	orf19.1172	20009	IPF6878	CLASSIFIC	hydrolase activity
CA4131	1.0	0.9	0.9	IPF6886	13218620..	unknown function	orf19.1171	6886	IPF20009	REGULATI	molecular_function unknown
CA4132	1.0	1.0	1.0	STE20	13221889..	serine/threonine-specit	orf19.4242	7017	IPF6886	UNCLASSI	protein binding
CA4133	1.1	1.8	1.3	IPF7010.3	complemer	unknown function, 3-pr	orf19.4241	7011	CaSTE20	REGULATI	protein kinase activity
CA4134	1.1	1.2	1.0	COS162	13228250..	involved in manganese	orf19.4240	7009	IPF7010.3	No significant S.c. match	
CA4135	0.9	0.9	1.0	IPF20163	13229832..	unknown function	orf19.4239	20163	CaCOS162	REGULATI	molecular_function unknown
CA4136	1.2	1.0	1.2	RET2	13232107..	Coatomer complex del	orf19.4236	7005	IPF20163	No significant S.c. match	
CA4137	0.9	1.0	0.9	CNA1	complemer	cyclic nucleotide phosp	orf19.4235	7003	CaRET2	CELLULAF	protein binding
CA4138	0.9	0.9	0.9	IPF11954	13237155..	unknown function	orf19.4234	11954	CaCNA1	Nucleotide	hydrolase activity
CA4139	1.0	1.1	1.0	THR4	complemer	threonine synthase (by	orf19.4233	11957	IPF11954	UNCLASSI	molecular_function unknown
CA4140	0.8	0.9	0.8	IPF11959	13240577..	unknown function	orf19.4232	11959	CaTHR4	Amino acid	lyase activity
CA4141	1.0	1.0	1.0	PTH2	complemer	proline transport helpe	orf19.1170	16485	IPF11959	UNCLASSI	molecular_function unknown
CA4142	1.1	1.0	1.8	PRE4	complemer	20S proteasome subur	orf19.1170	13337	CaPTH2	CLASSIFICATION NOT YET CLEAR-CUT	
CA4143	0.9	0.5	1.0	DDP1	13251761..	diadenosine and dipho	orf19.4229	13339	CaPRE4	PROTEIN I	peptidase activity
CA4144	1.1	1.0	1.0	IPF13340	complemer	unknown function	orf19.4228	13340	CaDDP1	CELL RES	hydrolase activity
CA4145	0.9	1.0	1.1	IPF3130	complemer	unknown function	orf19.4227	3130	IPF13340	UNCLASSI	molecular_function unknown
CA4146	1.0	0.9	1.0	LEU3	13256314..	Binds to UASs in prom	orf19.1170	3138	IPF3130	No significant S.c. match	
CA4147	1.0	1.0	0.9	ADE8	13263190..	Phosphoribosylglycina	orf19.1321	17327	CaLEU3	Amino acid	transcription regulator activity
CA4148	0.9	0.9	1.0	IDH2	complemer	Isocitrate dehydrogena	orf19.1321	6833	CaADE8	Nucleotide	transferase activity
CA4149	0.9	1.0	1.0	SMP3	13266283..	Protein kinase C pathv	orf19.5792	6831	CaIDH2	C-compour	oxidoreductase activity
CA4150	1.5	1.2	1.3	RPT3	complemer	26S proteasome regul	orf19.5793	6830	CaSMP3	CELL CYC	molecular_function unknown
CA4151	0.9	1.0	1.0	SHE9	complemer	causes lethality when	orf19.5796	6829	CaRPT3	PROTEIN I	peptidase activity
CA4153	0.9	1.1	1.0	DNLI	complemer	CANAL DNA LIGASE	orf19.5798	6823	CaSHE9	UNCLASSI	molecular_function unknown
CA4154	0.5	0.3	0.8	IPF20010	13277763..	unknown function	orf19.5799	20010	CaDNLI	CELL CYC	ligase activity
CA4155	1.1	1.1	1.2	RNR21	13281287..	ribonucleoside-diphosp	orf19.5801	10991	IPF20010	UNCLASSIFIED PROTEINS	
CA4156	1.1	0.9	1.0	IPF10990	13282933..	unknown function	orf19.5802	10990	CaRNR21	Nucleotide	oxidoreductase activity

CA4157	0.9	1.0	1.0	HYU1	13284597..	hydantoin utilization pr	orf19.5804	10288	IPF10990	UNCLASSI	molecular_function unknown
CA4158	1.0	1.0	0.9	DLD3	13288923..	D-lactate ferrycytochro	orf19.5805	10287	CaHYU1	Amino acid	molecular_function unknown
CA4159	0.9	1.2	0.6	ALD5	13293088..	aldehyde dehydrogena	orf19.1322	17030	CaDLD3	C-compound and carbohydrate metabolism ENERGY SUBCELLULAR LOCALISATION	
CA4160	1.0	1.0	1.1	IPF9294	13294957..	unknown function	orf19.5808	9294	CaALD5	ENERGY S	oxidoreductase activity
CA4161	1.0	0.9	1.0	IPF20164	complemer	putative kynurenine an	orf19.5809	20164	IPF9294	UNCLASSI	transferase activity
CA4162	0.8	0.2	1.0	MET1	complemer	siroheme synthase (by	orf19.5811	9300	IPF20164	Nitrogen ar	hydrolase activity
CA4163	0.5	0.3	0.8	IPF9301	13300548..	unknown function	orf19.5812	9301	CaMET1	Metabolism	transferase activity
CA4164	0.9	1.0	1.2	IPF9302	complemer	unknown function	orf19.1323	9302	IPF9301	UNCLASSI	molecular_function unknown
CA4165	1.0	1.0	1.1	IPF7774	complemer	unknown function	orf19.1048	7774	IPF9302	UNCLASSI	molecular_function unknown
CA4166	1.0	1.2	1.0	TIF34	complemer	Translation initiation fa	orf19.2967	7775	IPF7774	No significant S.c. match	
CA4167	1.3	1.1	1.2	IPF7778	complemer	putative carboxymethy	orf19.2966	7778	CaTIF34	PROTEIN S	translation regulator activity
CA4168	0.9	1.0	0.9	IPF7781	complemer	putative pump-driving	orf19.2965	7781	IPF7778	UNCLASSI	hydrolase activity
CA4169	1.0	1.0	1.0	RSC2	13309517..	Member of RSC compl	orf19.2964	7783	IPF7781	CELL RES	molecular_function unknown
CA4170	0.9	0.9	1.0	IPF7784	13312164..	unknown function	orf19.2963	7784	CaRSC2	CELL CYC	molecular_function unknown
CA4171	1.0	1.0	1.0	IPF7785	complemer	unknown function	orf19.2962	7785	IPF7784	CELL CYCLE AND DNA PROCESSING TRANSCRIPTION ""PROTEIN FATE [folding modification destination] ""CELL FATE SUBCELLUL	
CA4172	1.0	1.0	1.0	IPF4432	complemer	unknown function	orf19.2961	4432	IPF7785	No significant S.c. match	
CA4173	1.0	1.2	0.9	FRS2	complemer	phenylalanine--tRNA li	orf19.2960	4431	IPF4432	CELL CYCLE AND DNA PROCESSING TRANSCRIPTION SUBCELLULAR LOCALISATION	
CA4174	0.9	1.1	0.9	IPF4119.5	13324134..	unknown function, 5-prime end		4430	CaFRS2	PROTEIN S	ligase activity
CA4175	1.0	1.0	1.0	IPF4425.RI	13327113..	unknown function	orf19.2958	4427	IPF4119.5	CLASSIFICATION NOT YET CLEAR-CUT	
CA4176	0.9	0.9	0.9	IPF4425.RI	13330464..	unknown function	orf19.2957	4425	IPF4425.re	UNCLASSIFIED PROTEINS	
CA4177	1.0	1.0	1.0	MGM101	13333161..	mitochondrial genome	orf19.2956	4423	IPF4425.re	UNCLASSIFIED PROTEINS	
CA4178	1.1	0.9	1.0	IPF4421	complemer	unknown function	orf19.2954	4421	CaMGM10	CELL CYC	DNA binding
CA4179	0.9	1.0	1.0	TOM20	13335192..	mitochondrial outer me	orf19.2953	4420	IPF4421	No significant S.c. match	
CA4180	1.0	1.0	1.0	EXG2	13336189..	glucan 1,3-beta-glucos	orf19.2952	4419	CaTOM20	PROTEIN I	transporter activity
CA4181	0.9	1.1	0.9	HOM6	13337992..	homoserine dehydroge	orf19.2951	4417	CaEXG2	C-compound and carbohydrate metabolism CELL FATE SUBCELLULAR LOCALISATION	
CA4182	1.1	1.0	0.9	IPF19797	complemer	similar to Saccharomy	orf19.1046	19797	CaHOM6	Amino acid	oxidoreductase activity
CA4183	1.0	1.0	1.1	SNO1	complemer	hisH-like protein (by hc	orf19.1046	4408	IPF19797	CELL CYC	molecular_function unknown
CA4184	1.1	1.0	1.1	SNZ1	13345964..	stationary phase protei	orf19.2947	10248	CaSNO1	CELL RES	protein binding
CA4185	0.9	0.8	0.8	HNM4	complemer	Choline permease-like	orf19.2946	10246	CaSNZ1	CELL CYC	protein binding
CA4186	1.0	0.9	1.0	PUT4	13349485..	proline permease	orf19.2945	10243	CaHNM4	CELLULAR TRANSPORT AND TRANSPORT MECHANISMS SUBCELLULAR LOCALISATION TRANSPORT FACILITATION	
CA4187	1.0	1.0	1.0	IPF7524	13356397..	unknown function	orf19.1200	7524	CaPUT4	Amino acid metabolism TRANSPORT FACILITATION	
CA4188	1.1	1.1	1.0	IPF7525	13357317..	unknown function	orf19.4529	7525	IPF7524	UNCLASSI	molecular_function unknown
CA4189	2.2	1.7	1.7	IPF7527	complemer	unknown function		7527	IPF7525	No significant S.c. match	
CA4190	1.0	1.0	1.1	IPF7530	complemer	ATP-binding-cassette	orf19.1200	7530	IPF7527	CELLULAR COMMUNICATION/SIGNAL TRANSDUCTION MECHANISM REGULATION OF/INTERACTION WITH CELLULAR ENVIRON	
CA4191	0.9	1.0	0.9	IPF7531	complemer	unknown function	orf19.1200	7531	IPF7530	TRANSPO	transporter activity,hydrolase activity
CA4192	1.0	1.0	1.0	IPF7533	13367323..	unknown function	orf19.4533	7533	IPF7531	UNCLASSI	molecular_function unknown
CA4193	1.0	0.8	0.9	IPF7535	13369287..	unknown function	orf19.4534	7535	IPF7533	PROTEIN I	RNA binding
CA4194	1.0	0.9	1.1	PTR3	13371163..	transcriptional regulatc	orf19.4535	19799	IPF7535	UNCLASSI	molecular_function unknown
CA4195	0.9	1.0	1.0	CYS4	13373807..	cystathionine beta-syn	orf19.4536	8061	CaPTR3	TRANSCRIPTION	
CA4196	1.1	0.8	1.0	DST1	complemer	RNA polymerase II elo	orf19.4537	8059	CaCYS4	Amino acid	lyase activity
CA4197	0.8	0.8	1.0	IPF8057	13376497..	similar to Saccharomy	orf19.4538	8057	CaDST1	CELL CYC	transcription regulator activity
CA4198	1.0	0.9	1.0	IPF8055	13377995..	unknown function	orf19.4539	8055	IPF8057	TRANSCR	RNA binding
CA4199	1.0	1.0	1.0	UBC8	13379302..	ubiquitin-conjugating e	orf19.4540	8054	IPF8055	CELL FATE SUBCELLULAR LOCALISATION PROTEIN ACTIVITY REGULATION	
CA4200	0.9	0.9	0.9	CCL1	13380436..	cyclin (by homology)	orf19.4542	8051	CaUBC8	PROTEIN FATE [folding modification destination]	
CA4201	1.1	0.9	1.1	IPF8048	13381912..	probable succinate-ser	orf19.4543	8048	CaCCL1	CELL CYC	transcription regulator activity
CA4202	0.9	0.9	1.0	IPF8047	complemer	unknown function	orf19.4544	8047	IPF8048	Amino acid metabolism	
CA4203	1.0	1.0	1.0	SWI4	complemer	transcription factor (by	orf19.1202	17386	IPF8047	No significant S.c. match	
CA4204	0.9	0.9	0.7	HOL4	13390282..	member of major facilit	orf19.1202	14750	CaSWI4	CELL CYCLE AND DNA PROCESSING TRANSCRIPTION SUBCELLULAR LOCALISATION	
CA4205	0.8	0.7	1.0	IFA10	complemer	unknown function	orf19.1202	9076	CaHOL4	CELL RESCUE DEFENSE AND VIRULENCE ""TRANSPORT FACILITATION	
CA4206	1.0	0.9	1.0	IPF9079	complemer	Membrane transporter	orf19.4550	9079	CaIFA10	Nucleotide metabolism CELL CYCLE AND DNA PROCESSING CELLULAR COMMUNICATION/SIGNAL TRANSDUCTION MECHANISM	
CA4207	0.5	0.6	0.4	YAT1	complemer	carnitine acetyltransfer	orf19.4551	9082	IPF9079	CELL RES	molecular_function unknown
CA4208	1.0	1.1	1.0	CHS5.5EO	complemer	chitin biosynthesis, 5-prime end		18240	CaYAT1	Lipid fatty-a	transferase activity
CA4209	1.1	0.9	0.9	DEP1.3F	complemer	Regulator of phospholi	orf19.8427	18239	CaCHS5.5	C-compound and carbohydrate metabolism CELL FATE SUBCELLULAR LOCALISATION	
CA4210	0.9	1.0	1.2	DEP1.5F	complemer	Regulator of phospholi	orf19.8428	18238	CaDEP1.3	Lipid fatty-a	molecular_function unknown
CA4211	0.9	1.0	0.9	IPF5052	13410471..	RNA-binding protein (b	orf19.809	5051	CaDEP1.5	No significant S.c. match	
CA4212	1.0	0.9	1.0	HFM1	complemer	DNA/RNA helicase by	orf19.810	5050	IPF5052	UNCLASSI	RNA binding
CA4213	1.1	0.9	1.0	IPF5795	complemer	unknown function	orf19.811	5795	CaHFM1	TRANSCR	DNA binding,helicase activity
CA4214	0.9	0.9	0.9	IPF5796	complemer	unknown function	orf19.812	5796	IPF5795	UNCLASSI	molecular_function unknown
CA4215	0.7	0.7	0.8	SSY1	13422639..	Regulator of transporte	orf19.8434	5802	IPF5796	No significant S.c. match	
CA4216	1.2	1.6	1.6	IPF5806	13426650..	unknown function	orf19.8435	5806	CaSSY1	Amino acid metabolism TRANSCRIPTION	
CA4217	0.9	0.9	0.9	IPF5809	13433312..	unknown function	orf19.816	5809	IPF5806	UNCLASSI	molecular_function unknown
CA4218	1.2	1.2	1.1	ACP1	13438905..	mitochondrial acyl carr	orf19.819	8766	IPF5809	UNCLASSIFIED PROTEINS	
CA4219	1.0	0.9	1.0	SDS22	13439771..	regulatory subunit for t	orf19.820	8765	CaACP1	Lipid fatty-acid and isoprenoid metabolism ""SUBCELLULAR LOCALISATION TRANSPORT FACILITATION	
CA4220	0.8	0.6	0.6	IPF8762	13441293..	unknown function	orf19.822	8762	CaSDS22	CELL CYC	enzyme regulator activity
CA4221	1.0	1.1	1.0	IPF8760	13442052..	unknown function	orf19.823	8760	IPF8762	No significant S.c. match	
CA4222	0.8	0.9	1.0	GCD7	complemer	translation initiation fac	orf19.825	8759	IPF8760	No significant S.c. match	
CA4223	1.1	1.2	1.0	IPF8757	13444107..	unknown function	orf19.826	8757	CaGCD7	PROTEIN S	translation regulator activity
CA4224	1.1	1.1	1.0	IPF8755	complemer	unknown function	orf19.827	8755	IPF8757	No significant S.c. match	
CA4225	1.0	1.3	1.1	RPL39.3	complemer	ribosomal protein L39, 3-prime enc		8753	IPF8755	No significant S.c. match	
CA4226	1.1	1.0	1.0	IPF8752	complemer	similar to Saccharomy	orf19.828	8752	CaRPL39.3	PROTEIN S	molecular_function unknown
CA4227	1.0	1.0	1.0	SCH9	13452163..	strong similarity to S.p	orf19.829	13426	IPF8752	PROTEIN S	structural molecule activity
CA4228	0.9	0.9	0.9	IPF16057	13455459..	unknown function	orf19.831	16057	CaSCH9	CELL CYC	protein kinase activity

CA4229	1.0	1.0	0.9	IPF12480.5	complemer	unknown function, 5-prime end		12480	IPF16057	C-compour	transcription regulator activity
CA4230	1.1	1.1	1.0	IPF12481	13459645..	unknown function	orf19.513	12481	IPF12480.5	CELLULAR COMMUNICATION/SIGNAL TRANSDUCTION MECHANISM	
CA4231	1.1	0.9	1.0	SNP3	13461408..	snRNP-related protein	orf19.514	12483	IPF12481	CELL CYCLE AND DNA PROCESSING	
CA4232	1.0	1.0	0.9	IPF12484	complemer	unknown function	orf19.515	12484	CaSNP3	TRANSCR	RNA binding
CA4233	1.0	0.9	1.0	RFT1	complemer	nuclear division proteir	orf19.516	12486	IPF12484	UNCLASSI	molecular_function unknown
CA4234	0.9	1.0	1.0	HAP3	13465254..	CCAAT-binding factor	orf19.517	12488	CaRFT1	CELL CYC	transporter activity
CA4235	1.0	1.0	1.0	NCL1	13466093..	Probable proliferating-(	orf19.518	8167	CaHAP3	TRANSCR	transcription regulator activity
CA4236	1.0	1.0	0.9	IPF8166	complemer	unknown function	orf19.519	8166	CaNCL1	TRANSCR	transferase activity
CA4237	1.1	1.0	1.0	IPF6375	13469527..	unknown function	orf19.520	6375	IPF8166	TRANSCR	DNA binding,transcription regulator activity
CA4238	1.0	1.0	1.0	PIM1	13471159..	mitochondrial ATP-dep	orf19.522	8173	IPF6375	No significant S.c. match	
CA4239	1.0	0.9	1.0	IPF8174	complemer	unknown function	orf19.524	8174	CaPIM1	TRANSCR	peptidase activity
CA4240	1.1	1.1	1.0	NHP2	complemer	nucleolar rRNA proces	orf19.526	8176	IPF8174	No significant S.c. match	
CA4241	1.0	1.0	1.0	IPF8177	13477042..	unknown function	orf19.527	8177	CaNHP2	TRANSCR	RNA binding
CA4242	0.9	0.9	0.8	SEC26	complemer	beta chain of secretory	orf19.528	15941	IPF8177	No significant S.c. match	
CA4243	1.0	1.0	1.0	IPF16491	complemer	unknown function	orf19.529	16491	CaSEC26	CELLULAF	molecular_function unknown
CA4244	0.9	0.9	1.0	IPF4305	13485139..	unknown function	orf19.530	4305	IPF16491	C-compound and carbohydrate metabolism SUBCELLULAR LOCALISATION	
CA4245	1.1	1.0	1.1	IPF4303	complemer	unknown function	orf19.532	4303	IPF4305	No significant S.c. match	
CA4246	1.4	0.8	1.4	IPF4299	complemer	unknown function	orf19.535	4299	IPF4303	No significant S.c. match	
CA4247	1.0	1.0	1.0	TAF90	complemer	Probable transcription-	orf19.536	4296	IPF4299	No significant S.c. match	
CA4248	0.9	1.0	1.0	IPF4294	complemer	unknown function	orf19.537	4294	CaTAF90	TRANSCR	transcription regulator activity
CA4249	1.1	1.0	1.0	IPF4293	13495440..	similar to Saccharomy(	orf19.538	4293	IPF4294	UNCLASSI	molecular_function unknown
CA4250	2.0	1.4	1.3	IPF4292	complemer	bleomycin Hydrolase	orf19.539	4292	IPF4293	Lipid fatty-a	molecular_function unknown
CA4251	1.0	1.0	1.0	IPF4291	complemer	unknown function	orf19.540	4291	IPF4292	PROTEIN I	transcription regulator activity
CA4252	1.0	1.0	1.0	IPF4290	complemer	unknown function	orf19.541	4290	IPF4291	CELL CYCLE AND DNA PROCESSING SUBCELLULAR LOCALISATION	
CA4253	0.9	0.9	1.0	IPF4288	complemer	unknown function		4288	IPF4290	No significant S.c. match	
CA4254	1.0	1.1	1.1	IPF18234.3	13504904..	Unknown Function, 3-prime end		18234	IPF4288	No significant S.c. match	
CA4255	1.0	1.3	1.0	FUM11	13505439..	fumarate hydratase	orf19.543	4283	IPF18234.3	UNCLASSIFIED PROTEINS	
CA4256	1.0	1.0	0.9	IPF4282	complemer	unknown function	orf19.544	4282	CaFUM11	ENERGY S	lyase activity
CA4257	1.4	1.0	1.3	PRE6	13507748..	20S proteasome subunit		4281	IPF4282	TRANSCR	RNA binding
CA4258	1.1	1.0	0.9	IPF4279	complemer	unknown function	orf19.547	4279	CaPRE6	PROTEIN I	molecular_function unknown
CA4259	0.9	1.1	1.1	CDC10	complemer	cell division control prc	orf19.548	4277	IPF4279	CELL CYC	DNA binding
CA4260	0.9	0.8	0.9	IPF4276	13511668..	similar to Saccharomy(	orf19.549	4276	CaCDC10	C-compour	structural molecule activity
CA4261	1.0	1.0	1.2	PDX3	complemer	pyridoxamine-phospha	orf19.550	4274	IPF4276	PROTEIN S	structural molecule activity
CA4262	1.1	1.1	1.0	IPF2275	complemer	unknown function	orf19.6654	2275	CaPDX3	Metabolism	oxidoreductase activity
CA4263	1.2	1.2	1.2	IPF2277	complemer	unknown function	orf19.6656	2277	IPF2275	UNCLASSIFIED PROTEINS	
CA4264	1.0	1.1	1.1	IPF2280	complemer	unknown function	orf19.6658	2280	IPF2277	REGULATION OF/INTERACTION WITH CELLULAR ENVIRONMENT SUBCELLULAR LOCALISATION TRANSPORT FACILITATION	
CA4265	1.3	1.2	1.1	GAP6	13521030..	General amino acid pe	orf19.6659	2282	IPF2280	No significant S.c. match	
CA4266	1.6	1.2	1.4	IPF2283	complemer	unknown function	orf19.6660	2283	CaGAP6	Amino acid metabolism CELLULAR TRANSPORT AND TRANSPORT MECHANISMS SUBCELLULAR LOCALISATION TRANSPORT FA	
CA4267	0.9	0.9	1.0	IPF2286	13526537..	unknown function	orf19.6661	2286	IPF2283	UNCLASSI	molecular_function unknown
CA4268	1.1	1.1	1.1	IPF2287	complemer	unknown function	orf19.6662	2287	IPF2286	No significant S.c. match	
CA4269	1.3	1.2	1.2	RPS25B	complemer	Cytosolic ribosomal pr	orf19.6663	2288	IPF2287	UNCLASSI	molecular_function unknown
CA4270	1.0	1.0	1.1	NUP2	13529973..	Nuclear pore protein (b	orf19.6665	2295	CaRPS25E	PROTEIN S	structural molecule activity
CA4271	1.0	0.9	1.0	SAP30	13532889..	Subunit of the histone	orf19.6667	2297	CaNUP2	CELLULAF	structural molecule activity
CA4272	1.1	1.1	1.1	CUE1	13533828..	Involved in ubiquitinatio	orf19.6668	2299	CaSAP30	TRANSCR	hydrolase activity
CA4273	1.0	1.0	1.0	CAC2	complemer	Chromatin assembly co	orf19.6670	2301	CaCUE1	PROTEIN I	protein binding
CA4274	1.0	1.0	1.0	LAP42	13536542..	Aminopeptidase yscI p	orf19.6671	2303	CaCAC2	CELL CYC	molecular_function unknown
CA4275	1.1	1.0	1.0	MDJ1	13538604..	Heat shock protein - cl	orf19.6672	2305	CaLAP42	PROTEIN FATE [folding modification destination] ""SUBCELLULAR LOCALISATION	
CA4276	0.9	1.0	0.9	HEX1	13540810..	ß-N-acetylglucosamini	orf19.6673	2308	CaMDJ1	PROTEIN I	chaperone activity
CA4277	0.9	0.9	1.1	BTS1	13543155..	Geranylgeranyl diphos	orf19.6674	2309	CaHEX1	No significant S.c. match	
CA4278	0.9	1.0	1.0	IPF2310	complemer	unknown function	orf19.6675	2310	CaBTS1	Lipid fatty-a	transferase activity
CA4279	0.9	1.0	0.9	DPH51	13544839..	Diphthamide methyltra	orf19.6676	2311	IPF2310	No significant S.c. match	
CA4280	1.0	1.1	1.1	IFJ5	complemer	Unknown function	orf19.6678	2313	CaDPH51	Amino acid	transferase activity
CA4281	1.0	0.9	1.0	IPF2314	complemer	unknown function	orf19.6679	2314	CaIFJ5	UNCLASSIFIED PROTEINS	
CA4282	1.0	1.1	1.0	IPF2319	13552041..	unknown function	orf19.6680	2319	IPF2314	No significant S.c. match	
CA4283	1.0	1.1	1.0	IFJ4	13555358..	Unknown function	orf19.6681	2321	IPF2319	TRANSCRIPTION	
CA4284	0.9	0.5	1.0	DPH52.3E(	complemer	Diphthamide methyltra	orf19.6682	2322	CaIFJ4	UNCLASSIFIED PROTEINS	
CA4287	0.9	1.0	0.9	IPF20011.3	13557688..	similar to Saccharomyces cerevisi		20012	CaDPH52.3	Amino acid metabolism	
CA4288	0.9	1.1	0.9	IPF6662	complemer	similar to Saccharomy(	orf19.6685	6662	IPF20011.3	UNCLASSIFIED PROTEINS	
CA4289	0.9	1.0	1.0	IPF6665	complemer	unknown function	orf19.6686	6665	IPF6662	TRANSCR	RNA binding
CA4290	0.9	0.8	1.0	IPF2617	complemer	unknown function	orf19.6687	2617	IPF6665	UNCLASSI	molecular_function unknown
CA4291	0.2	0.4	0.1	IPF2615	13568848..	unknown function	orf19.6688	2615	IPF2617	No significant S.c. match	
CA4293	1.1	1.1	1.1	IFA8	13571419..	Unknown function	orf19.6690	2611	IPF2615	No significant S.c. match	
CA4294	1.0	1.0	1.0	ERC1	complemer	ethionine resistance pr	orf19.6691	2610	CaIFA8	UNCLASSIFIED PROTEINS	
CA4295	0.9	1.0	1.0	MNN7	13579767..	putative Golgi alpha-1,	orf19.6692	2606	CaERC1	CELL RES	molecular_function unknown
CA4296	0.9	1.0	1.0	IPF2605	complemer	unknown function	orf19.6693	2605	CaMNN7	CELL FATE	
CA4297	0.9	0.9	0.9	IPF2603	complemer	unknown function	orf19.6694	2603	IPF2605	UNCLASSI	molecular_function unknown
CA4298	1.0	1.0	1.0	TIM9	complemer	Mitochondrial inner me	orf19.6696	2599	IPF2603	TRANSCRIPTION CELL FATE SUBCELLULAR LOCALISATION	
CA4299	1.0	1.0	1.0	IPF2598	complemer	unknown function	orf19.6698	2598	CaTIM9	PROTEIN I	chaperone activity
CA4300	1.0	0.9	1.0	HIS2	13590854..	Histidinol phosphatase	orf19.6699	2596	IPF2598	PROTEIN S	ligase activity
CA4301	1.0	1.0	1.0	IPF2593	complemer	amino acid-tRNA ligas	orf19.6701	2593	CaHIS2	Amino acid	hydrolase activity
CA4302	1.0	1.2	1.0	DED81	13594793..	Asparaginyl-tRNA synt	orf19.6702	2591	IPF2593	PROTEIN S	ligase activity
CA4303	1.0	1.0	1.1	IFB1	13596937..	unknown function	orf19.6703	2590	CaDED81	PROTEIN S	ligase activity

CA4304	1.0	1.1	1.1	IPF6649	13599850..	unknown function	orf19.6704	6649	CaIFB1	CELLULAR TRANSPORT AND TRANSPORT MECHANISMS SUBCELLULAR LOCALISATION	
CA4305	1.0	1.1	1.0	IPF6654	13605657..	unknown function	orf19.6705	6654	IPF6649	CELL CYCLE AND DNA PROCESSING PROTEIN SYNTHESIS SUBCELLULAR LOCALISATION	
CA4306	0.9	0.9	0.9	GYP7	13609552..	GTPase activating prot	orf19.6706	6656	IPF6654	UNCLASSI	molecular_function unknown
CA4307	1.0	0.9	1.0	IPF20013	13612322..	Unknown function	orf19.6707	20013	CaGYP7	CONTROL	enzyme regulator activity
CA4308	0.9	1.0	1.0	IPF6660	13613872..	unknown function	orf19.6708	6660	IPF20013	PROTEIN S	hydrolase activity
CA4309	1.1	1.1	1.1	IPF2127.3	13616446..	unknown function, 3-pr	orf19.4117	2127	IPF6660	UNCLASSIFIED PROTEINS	
CA4310	1.0	0.9	1.0	IPF2125	complemer	unknown function	orf19.4116	2125	IPF2127.3	No significant S.c. match	
CA4311	0.9	0.8	0.9	FAA23	13620672..	Long-chain-fatty-acid--	orf19.4114	2123	IPF2125	UNCLASSIFIED PROTEINS	
CA4312	1.1	1.1	1.0	IPF2122	complemer	similar to thiamin pyrop	orf19.4112	2122	CaFAA23	Lipid fatty-acid and isoprenoid metabolism ""CELLULAR TRANSPORT AND TRANSPORT MECHANISMS SUBCELLULAR LOCALISATIC	
CA4313	1.0	1.0	1.0	IPF2121	13624191..	unknown function	orf19.4110	2121	IPF2122	Metabolism	molecular_function unknown
CA4314	1.0	1.1	1.1	PMT4	13627689..	Mannosyltransferase (l	orf19.4109	2115	IPF2121	CELL CYCLE AND DNA PROCESSING SUBCELLULAR LOCALISATION	
CA4315	1.0	1.0	1.0	PPX1	13630268..	Exopolyphosphatase (l	orf19.4107	2112	CaPMT4	C-compour	transferase activity
CA4316	1.0	1.0	1.0	IPF2111	complemer	unknown function	orf19.4106	2111	CaPPX1	Phosphate	hydrolase activity
CA4317	1.1	1.1	1.1	IPF2109	13632715..	unknown function	orf19.4105	2109	IPF2111	UNCLASSI	molecular_function unknown
CA4318	1.0	1.0	1.0	IPF2106	complemer	unknown function	orf19.4104	2106	IPF2109	UNCLASSI	molecular_function unknown
CA4319	1.0	1.0	1.2	RPN10	complemer	Protein degradation (by	orf19.4102	2104	IPF2106	No significant S.c. match	
CA4320	1.0	1.1	1.0	ECM17	complemer	Putative sulfite reducta	orf19.4099	2102	CaRPN10	PROTEIN I	peptidase activity
CA4321	1.1	1.0	1.0	KAR3.53	13639735..	Kinesin-related protein	orf19.4100	2100	CaECM17	Amino acid	transporter activity
CA4322	1.0	1.2	1.1	IPF2097	complemer	unknown function	orf19.4097	2097	CaKAR3.5:	CELL CYC	motor activity
CA4323	1.0	0.9	1.0	IPF2096	complemer	putative acyltransferas	orf19.4096	2096	IPF2097	TRANSCRIPTION	
CA4324	1.1	1.0	1.0	IPF2095	complemer	unknown function	orf19.4095	2095	IPF2096	CLASSIFIC	transferase activity
CA4325	1.1	1.1	1.0	IPF2094	13646015..	unknown function	orf19.4094	2094	IPF2095	No significant S.c. match	
CA4326	0.9	1.1	0.9	IPF2093	13646785..	nuclear protein of unkr	orf19.4093	2093	IPF2094	No significant S.c. match	
CA4327	0.9	1.0	1.0	IPF2091	13648774..	unknown function	orf19.4092	2091	IPF2093	UNCLASSI	molecular_function unknown
CA4328	0.9	1.0	0.9	IPF2090	complemer	similar to Saccharomyc	orf19.4091	2090	IPF2091	UNCLASSI	molecular_function unknown
CA4329	1.0	0.9	1.0	VMA21	complemer	Vacuolar H+-ATPase assembly (b		2089	IPF2090	TRANSCR	transcription regulator activity
CA4330	0.9	1.0	0.9	IPF2087	13651418..	unknown function	orf19.4090	2087	CaVMA21	PROTEIN I	molecular_function unknown
CA4331	1.2	1.2	1.8	IPF2086	13653176..	unknown function	orf19.4089	2086	IPF2087	UNCLASSIFIED PROTEINS	
CA4332	1.1	1.1	1.1	GLO2	13654688..	Glyoxalase II (hydroxya	orf19.4088	2085	IPF2086	CELL CYC	chaperone activity
CA4333	0.9	0.9	1.0	IPF2083	complemer	unknown function	orf19.4086	2083	CaGLO2	Amino acid	hydrolase activity
CA4334	1.1	1.1	1.3	IPF2082	13656458..	unknown function	orf19.4085	2082	IPF2083	UNCLASSI	molecular_function unknown
CA4335	0.8	0.9	1.0	GAL83	13657376..	Glucose repression pro	orf19.4084	2079	IPF2082	CELL RESCUE DEFENSE AND VIRULENCE ""TRANSPORT FACILITATION	
CA4336	9.1	2.8	5.8	DDR48	complemer	stress protein (by hom	orf19.4082	2075	CaGAL83	C-compour	protein kinase activity
CA4337	1.1	1.1	1.0	IPF2071	complemer	unknown function	orf19.4079	2071	CaDDR48	CELL RESCUE DEFENSE AND VIRULENCE ""UNCLASSIFIED PROTEINS	
CA4338	1.1	1.1	1.0	IPF3616	13666944..	Unknown function	orf19.6709	3616	IPF2071	No significant S.c. match	
CA4339	1.2	1.2	1.2	IPF3618	complemer	Unknown function	orf19.6710	3618	IPF3616	UNCLASSI	molecular_function unknown
CA4340	1.4	1.4	1.0	IPF3621	complemer	unknown function	orf19.6712	3621	IPF3618	UNCLASSIFIED PROTEINS	
CA4341	1.1	1.2	1.0	IPF3624	complemer	unknown function	orf19.6713	3624	IPF3621	CLASSIFIC	molecular_function unknown
CA4342	0.8	0.7	0.7	IPF3629	complemer	RNA (guanine-N7-) me	orf19.6716	3629	IPF3624	No significant S.c. match	
CA4343	1.2	1.1	1.2	IPF3630	complemer	unknown function	orf19.6717	3630	IPF3629	TRANSCR	transferase activity
CA4344	1.1	1.1	1.1	IPF3631	13680189..	unknown function	orf19.6718	3631	IPF3630	UNCLASSI	molecular_function unknown
CA4345	1.0	1.1	1.0	IPF3633.3	complemer	similar to Saccharomyc	orf19.6719	3633	IPF3631	No significant S.c. match	
CA4346	1.2	1.3	1.5	IPF3634	complemer	unknown function	orf19.6720	3634	IPF3633.3	CELL CYC	transcription regulator activity
CA4347	1.0	0.9	0.9	IPF3636	complemer	similar to Saccharomyc	orf19.6722	3636	IPF3634	UNCLASSI	molecular_function unknown
CA4348	1.0	1.1	1.1	IPF3638	13689201..	unknown function	orf19.6723	3638	IPF3636	CELL CYC	DNA binding
CA4349	1.0	1.0	1.0	FUM12.5F	13691337..	Fumarate hydratase, 5	orf19.6724	3640	IPF3638	UNCLASSIFIED PROTEINS	
CA4350	1.0	0.7	1.1	FUM12.53I	13692040..	Fumarate hydratase, internal fragr		18225	CaFUM12.	ENERGY SUBCELLULAR LOCALISATION	
CA4351	0.9	1.0	0.9	FUM12.3F	13692234..	Fumarate hydratase, 3	orf19.6725	3641	CaFUM12.	ENERGY SUBCELLULAR LOCALISATION	
CA4352	1.1	0.9	1.0	IPF3642	13693048..	Unknown function	orf19.6726	3642	CaFUM12.	ENERGY SUBCELLULAR LOCALISATION	
CA4353	1.1	1.0	1.2	IPF3645	13694115..	similar to Saccharomyc	orf19.6727	3645	IPF3642	TRANSCR	DNA binding
CA4354	1.0	1.1	1.0	IPF3647	complemer	unknown function	orf19.6729	3647	IPF3645	TRANSCR	transferase activity
CA4355	1.0	1.1	1.0	IPF3649	complemer	unknown function	orf19.6730	3649	IPF3647	TRANSCRIPTION PROTEIN SYNTHESIS	
CA4356	0.9	0.9	1.0	IPF3651	complemer	unknown function		3651	IPF3649	UNCLASSI	molecular_function unknown
CA4357	0.9	0.9	0.9	IPF19800	13701331..	unknown function	orf19.6732	19800	IPF3651	No significant S.c. match	
CA4358	2.0	1.8	1.3	IPF6067	complemer	putative transcription fa	orf19.6734	6067	IPF19800	UNCLASSI	molecular_function unknown
CA4359	1.1	1.0	1.0	IPF6054	13713281..	unnown function	orf19.6736	6054	IPF6067	TRANSCRIPTION SUBCELLULAR LOCALISATION	
CA4360	1.1	0.9	1.0	IPF6050	13717654..	unknown function	orf19.6737	6050	IPF6054	UNCLASSI	molecular_function unknown
CA4361	1.0	1.0	1.0	IPF16104	13723251..	unknown function	orf19.1309	16104	IPF6050	No significant S.c. match	
CA4362	0.6	0.8	0.5	ATP2	13726192..	F1F0-ATPase complex	orf19.5653	5264	IPF16104	TRANSCR	molecular_function unknown
CA4363	1.1	1.1	1.1	STE24	13728012..	zinc metallo-protease t	orf19.5654	5265	CaATP2	ENERGY C	transporter activity
CA4364	1.0	1.0	1.1	IPF5268.E	complemer	choline monooxygenas	orf19.5655	5266	CaSTE24	PROTEIN I	peptidase activity
CA4365	1.0	0.9	0.9	IPF5268.E	complemer	choline monooxygenas	orf19.5656	5268	IPF5268.e	No significant S.c. match	
CA4366	1.0	1.0	0.9	SWI1	complemer	Transcription regulatio	orf19.5657	5271	IPF5268.e	No significant S.c. match	
CA4367	1.0	1.0	1.0	MNN10	complemer	galactosyltransferase (	orf19.5658	5272	CaSWI1	C-compour	transcription regulator activity
CA4368	1.1	1.0	1.0	PEX10.3	complemer	peroxisomal assembly	orf19.5660	5275	CaMNN10	C-compour	transferase activity
CA4369	1.0	1.1	1.1	TIM11	complemer	subunit e of mitochondrial F1F0-A		5277	CaPEX10.3	PROTEIN I	protein binding
CA4370	1.0	1.2	1.0	IPF5279	13737863..	unknown function	orf19.5661	5279	CaTIM11	ENERGY ""PROTEIN FATE [folding modification destination] ""CELLULAR TRANSPORT AND TRANSPORT MECHANISMS SUBCELLUL	
CA4371	1.0	1.0	1.1	PEP7	13739024..	vacuolar segregation p	orf19.5662	5281	IPF5279	ENERGY S	protein phosphatase activity
CA4372	0.9	1.0	1.0	IPF5282	complemer	unknown function	orf19.5663	5282	CaPEP7	PROTEIN I	molecular_function unknown
CA4373	0.9	0.8	0.9	IPF5287	13742925..	signal transduction pro	orf19.5664	5287	IPF5282	UNCLASSI	transporter activity
CA4374	1.0	1.0	0.9	IPF5288	13744929..	Unknown function	orf19.5665	5288	IPF5287	CELL CYC	protein binding
CA4375	1.1	1.1	1.1	IPF11309	13746058..	unknown function	orf19.5666	11309	IPF5288	UNCLASSI	molecular_function unknown

CA4376	0.9	0.9	1.0	IPF11307	13747288..	unknown function	orf19.5667	11307	IPF11309	TRANSCR transcription regulator activity
CA4377	1.0	1.0	0.9	IPF11301	13749902..	unknown function	orf19.5669	11301	IPF11307	CELL RES transporter activity
CA4378	0.9	0.8	0.9	IPF11299	complemer	unknown function	orf19.5671	11299	IPF11301	SUBCELLULAR LOCALISATION
CA4379	0.6	0.4	0.9	MEP2	13754336..	high affinity low capaci	orf19.5672	12738	IPF11299	UNCLASSI molecular_function unknown
CA4380	0.8	0.7	0.9	IPF12736	complemer	unknown function	orf19.5673	12736	CaMEP2	CELLULAF transporter activity
CA4381	2.4	2.3	1.6	IPF20169	complemer	unknown function	orf19.5674	20169	IPF12736	TRANSPORT FACILITATION
CA4382	1.0	1.0	1.1	IPF8576	complemer	similar to Saccharomy	orf19.5675	8576	IPF20169	No significant S.c. match
CA4383	1.0	0.9	1.0	IPF8573	complemer	unknown function	orf19.1312	8573	IPF8576	UNCLASSI hydrolase activity
CA4384	1.0	1.1	1.0	DUR34	13767917..	Urea transport protein	orf19.1312	8570	IPF8573	UNCLASSI molecular_function unknown
CA4385	1.3	1.1	1.0	CCT6	complemer	component of chapero	orf19.3126	14978	CaDUR34	REGULATION OF/INTERACTION WITH CELLULAR ENVIRONMENT SUBCELLULAR LOCALISATION TRANSPORT FACILITATION
CA4386	0.9	0.9	0.9	IPF12412	13777240..	unknown function	orf19.3125	12412	CaCCT6	PROTEIN I chaperone activity
CA4387	0.9	1.0	1.0	MAP1	complemer	methionine aminopepti	orf19.3124	12414	IPF12412	TRANSCRIPTION SUBCELLULAR LOCALISATION
CA4388	1.0	0.9	1.1	IPF12416.3	complemer	cytidine deaminase, 3-prime end (		12416	CaMAP1	PROTEIN I peptidase activity
CA4389	0.8	0.7	1.0	RPT5	13780287..	26S proteasome regul	orf19.3123	12419	IPF12416.3	Nucleotide hydrolase activity
CA4390	1.0	1.1	1.1	HOD1	complemer	regulator of G2/M progression (by		12420	CaRPT5	PROTEIN I peptidase activity
CA4391	0.9	0.8	0.9	ARR3	13782944..	involved in arsenite tra	orf19.3122	12422	CaHOD1	No significant S.c. match
CA4392	1.0	1.0	0.9	IPF12303	13784434..	glutathione-S-transfera	orf19.3121	12303	CaARR3	SUBCELLU transporter activity
CA4393	1.0	1.0	1.0	IPF12300	13785662..	unknown function	orf19.3120	12300	IPF12303	Nitrogen and sulphur metabolism
CA4394	1.1	1.1	1.2	IPF12297	complemer	mycelial surface antige	orf19.3117	12297	IPF12300	TRANSPORT FACILITATION
CA4395	1.0	1.0	1.1	EXM2	complemer	EXit from Mitosis (by h	orf19.3116	12295	IPF12297	No significant S.c. match
CA4396	1.0	1.0	1.0	IPF12294	complemer	unknown function	orf19.3115	12294	CaEXM2	TRANSCR RNA binding
CA4397	0.9	0.9	1.0	IPF14369	13791307..	unknown function	orf19.3114	14369	IPF12294	SUBCELLULAR LOCALISATION
CA4398	1.8	3.5	1.2	ZRT1	complemer	high-affinity zinc transp	orf19.3112	14367	IPF14369	Nucleotide lyase activity
CA4399	1.0	1.3	1.0	PRA1	13794736..	pH-regulated antigen	orf19.3111	14364	CaZRT1	REGULATION OF/INTERACTION WITH CELLULAR ENVIRONMENT TRANSPORT FACILITATION
CA4400	1.1	1.1	1.1	IPF14362	13796197..	unknown function	orf19.3110	8781	CaPRA1	UNCLASSI molecular_function unknown
CA4401	1.0	1.0	1.1	IPF8780	13798417..	unknown function	orf19.3109	8780	IPF14362	UNCLASSIFIED PROTEINS
CA4402	1.0	1.0	1.0	MGT1	complemer	O6-methylguanine DN	orf19.3108	8778	IPF8780	No significant S.c. match
CA4403	0.9	0.9	0.9	IPF8777	complemer	unknown function	orf19.3107	8777	CaMGT1	CELL CYC DNA binding
CA4404	1.1	1.0	1.0	MET16	13801316..	3 -phosphoadenylylsul	orf19.3106	8775	IPF8777	No significant S.c. match
CA4405	1.1	1.0	0.9	IPF8773	13802252..	putative cytochrome P	orf19.3105	8773	CaMET16	Amino acid oxidoreductase activity
CA4406	1.1	0.9	1.0	YDC1	13804150..	alkaline dihydrocerami	orf19.3104	8772	IPF8773	CELL RESCUE DEFENSE AND VIRULENCE ""CELL FATE CONTROL OF CELLULAR ORGANIZATION
CA4407	1.1	1.0	1.0	RPO31	13805409..	DNA-directed RNA pol	orf19.3103	13155	CaYDC1	Lipid fatty-a hydrolase activity
CA4408	0.9	0.9	0.9	CTA6.3	13809882..	unknown function, 3-pr	orf19.3102	13152	CaRPO31	TRANSCR nucleotidyltransferase activity
CA4409	1.0	1.0	1.0	IPF13151	complemer	unknown function	orf19.3100	13151	CaCTA6.3	No significant S.c. match
CA4410	0.9	0.9	1.0	TRP4	complemer	Anthranilate phosphori	orf19.3099	1754	IPF13151	CELLULAR TRANSPORT AND TRANSPORT MECHANISMS SUBCELLULAR LOCALISATION
CA4411	1.0	1.1	1.1	BRR2	complemer	RNA helicase-related	orf19.3098	1751	CaTRP4	Amino acid transferase activity
CA4412	1.5	2.4	1.9	PDA1	complemer	Pyruvate dehydrogena	orf19.3097	1749	CaBRR2	TRANSCR RNA binding,helicase activity
CA4413	1.0	0.9	1.2	MSH2	complemer	DNA mismatch repair	orf19.3093	1746	CaPDA1	C-compour oxidoreductase activity
CA4415	0.9	0.8	1.2	HIK1.3EOC	13826051..	histidine kinase, 3-prime end		11890	CaMSH2	CELL CYC DNA binding
CA4416	1.2	1.6	1.2	IPF11888	13830957..	unknown function	orf19.5180	11888	CaHIK1.3e	CELL CYCLE AND DNA PROCESSING TRANSCRIPTION CELLULAR COMMUNICATION/SIGNAL TRANSDUCTION MECHANISM ""CE
CA4417	1.0	0.9	1.0	LIP5	complemer	Secretory lipase	orf19.5179	5395	IPF11888	CELL RES oxidoreductase activity
CA4418	0.7	0.6	1.1	ERG5	complemer	C-22 sterol desaturase	orf19.5178	5398	CaLIP5	Other virulence attributes
CA4419	1.0	0.9	1.0	IFA6	13840496..	Unknown function	orf19.5177	5401	CaERG5	Lipid fatty-acid and isoprenoid metabolism """"CELL RESCUE DEFENSE AND VIRULENCE ""SUBCELLULAR LOCALISATION
CA4420	1.2	1.2	1.0	SSM4	complemer	involved in mRNA turn	orf19.5175	5404	CaIFA6	UNCLASSIFIED PROTEINS
CA4421	1.2	1.1	1.1	TAF19	complemer	TBP-associated factor	orf19.5174	5405	CaSSM4	TRANSCR ligase activity
CA4422	1.1	1.2	1.2	LIP9.EXON	complemer	secretory lipase 9, exo	orf19.5173	5406	CaTAF19	TRANSCR transcription regulator activity
CA4423	1.0	1.0	1.1	LIP9.EXON	complemer	secretory lipase 9, exo	orf19.5172	5407	CaLIP9.exo	Other virulence attributes
CA4424	1.1	1.0	1.1	PMT1	complemer	mannosyltransferase	orf19.5171	5408	CaLIP9.exo	Other virulence attributes
CA4425	0.9	2.0	1.0	ENA21.3	13855072..	P-type ATPase, 3-prim	orf19.5170	5411	CaPMT1	C-compour transferase activity
CA4426	1.0	0.9	1.0	AMD21	13858996..	amidase (by homology	orf19.5169	5587	CaENA21.3	REGULATION OF/INTERACTION WITH CELLULAR ENVIRONMENT TRANSPORT FACILITATION
CA4427	1.0	1.0	0.9	IPF5584	complemer	unknown function	orf19.5168	5584	CaAMD21	Nitrogen and sulphur metabolism
CA4428	1.2	1.1	1.1	IFM1	complemer	translation initiation fac	orf19.5167	5581	IPF5584	UNCLASSI molecular_function unknown
CA4429	0.9	0.9	1.1	DBF4	complemer	regulatory subunit for (	orf19.5166	5578	CaIFM1	PROTEIN S RNA binding
CA4430	1.1	1.0	1.0	IPF5577	complemer	unknown function	orf19.5165	5577	CaDBF4	CELL CYC protein kinase activity
CA4431	1.0	0.9	1.0	ECM39	13868170..	cell wall biogenesis by	orf19.5164	5575	IPF5577	UNCLASSI molecular_function unknown
CA4432	0.8	0.2	0.9	IPF5574	complemer	unknown function	orf19.5163	5574	CaECM39	CONTROL transferase activity
CA4433	0.9	0.9	1.0	BCK1	13873674..	serine/threonine protei	orf19.5162	5572	IPF5574	CELL CYC molecular_function unknown
CA4434	0.9	1.0	1.0	MRPL49	complemer	ribosomal protein mito	orf19.5161	5569	CaBCK1	CELL CYC protein kinase activity,signal transducer activity
CA4435	0.9	0.9	0.8	SAP190	13879174..	phosphatase associate	orf19.5160	5567	CaMRPL49	PROTEIN S structural molecule activity
CA4436	1.0	1.1	1.0	IPF13868	complemer	unknown function	orf19.5159	13868	CaSAP190	CELL CYC protein phosphatase activity
CA4437	1.2	1.5	1.8	IPF13867	13883597..	unknown function	orf19.5158	13867	IPF13868	UNCLASSI molecular_function unknown
CA4438	1.0	0.9	0.9	IPF13866	13884332..	unknown function	orf19.5157	13866	IPF13867	CELLULAR TRANSPORT AND TRANSPORT MECHANISMS SUBCELLULAR LOCALISATION
CA4439	1.0	1.1	1.0	IPF13865	13885085..	unknown function	orf19.5156	13865	IPF13866	UNCLASSIFIED PROTEINS
CA4440	1.0	1.0	0.9	IPF13864	complemer	similar to Saccharomy	orf19.5155	13864	IPF13865	UNCLASSI molecular_function unknown
CA4441	1.2	1.2	1.0	IPF2268.3	complemer	unknown function, 3-prime end		2268	IPF13864	C-compour molecular_function unknown
CA4442	1.0	1.0	0.9	IPF3537	complemer	unknown function	orf19.6838	3537	IPF2268.3	No significant S.c. match
CA4443	0.9	1.0	1.1	IPF3535	complemer	unknown function	orf19.6840	3535	IPF3537	ENERGY SUBCELLULAR LOCALISATION
CA4444	1.0	1.0	1.0	IPF3533	complemer	putative GDP/GTP exc	orf19.6842	3533	IPF3535	No significant S.c. match
CA4445	1.0	0.7	0.9	IPF3530	13913089..	unknown function	orf19.6843	3530	IPF3533	UNCLASSI enzyme regulator activity
CA4446	0.4	0.8	0.4	ICL1	13915305..	Isocitrate lyase	orf19.6844	3527	IPF3530	No significant S.c. match
CA4447	1.0	1.0	1.0	IPF3523	13917397..	unknown function	orf19.6845	3523	CaICL1	C-compour lyase activity
CA4448	0.9	0.9	1.0	PHO85	complemer	Negative regulator of F	orf19.6846	3522	IPF3523	UNCLASSIFIED PROTEINS

CA4449	0.9	0.9	0.9	IPF3520	complemer	unknown function	orf19.6847	3520	CaPHO85	Phosphate	protein kinase activity
CA4450	1.1	0.9	1.0	IPF3518	complemer	similar to Saccharomy	orf19.6848	3518	IPF3520	UNCLASSI	molecular_function unknown
CA4451	1.0	1.0	1.0	ELC1	13925378..	Transcription elongatic	orf19.6849	3516	IPF3518	PROTEIN I	molecular_function unknown
CA4452	1.0	0.9	1.0	IPF3514	13926226..	unknown function	orf19.6850	3514	CaELC1	CELL CYC	transcription regulator activity
CA4453	1.0	1.0	1.1	CHL4	complemer	chromosome segregat	orf19.6851	15162	IPF3514	UNCLASSI	molecular_function unknown
CA4454	0.9	0.9	1.1	IPF15160	complemer	unknown function	orf19.6852	15160	CaCHL4	CELL CYC	DNA binding
CA4455	1.0	1.0	1.1	IPF8454	complemer	unknown function	orf19.6853	8454	IPF15160	UNCLASSI	molecular_function unknown
CA4456	1.1	1.1	1.1	ATP1.EXO	13935906..	F1F0-ATPase complex, F1 alpha s		8458	IPF8454	No significant S.c. match	
CA4457	0.8	1.5	0.5	ATP1.EXO	13936228..	F1F0-ATPase complex	orf19.6854	8460	CaATP1.ex	ENERGY CELLULAR TRANSPORT AND TRANSPORT MECHANISMS REGULATION OF/INTERACTION WITH CELLULAR ENVIRONM	
CA4458	0.7	0.5	1.0	IPF8464	13938534..	unknown function	orf19.6855	8464	CaATP1.ex	ENERGY (	transporter activity
CA4459	0.9	1.0	0.9	NSP49.3F	complemer	nucleoporin, 3-prime e	orf19.6856	8467	IPF8464	No significant S.c. match	
CA4460	0.9	0.9	0.9	NSP49.5F	complemer	nuclear pore protein, 5	orf19.6857	8469	CaNSP49.3	TRANSCR	structural molecule activity
CA4461	1.0	1.0	0.9	IPF8470	complemer	unknown function	orf19.6858	8470	CaNSP49.5	No significant S.c. match	
CA4462	0.9	0.9	1.0	IPF17251	complemer	unknown function	orf19.6859	17251	IPF8470	UNCLASSI	molecular_function unknown
CA4463	1.0	1.0	1.0	PIS1	13947475..	CDP diacylglycerol--in	orf19.6860	9827	IPF17251	No significant S.c. match	
CA4464	1.1	1.0	1.1	IPF9828	complemer	similar to Saccharomy	orf19.6861	9828	CaPIS1	Lipid fatty-a	transferase activity
CA4465	0.9	0.9	0.9	IPF9829	complemer	unknown function	orf19.6862	9829	IPF9828	CELL CYC	protein binding
CA4466	1.2	1.3	1.1	VPH1	complemer	H+-ATPase V0 domair	orf19.6863	9830	IPF9829	UNCLASSI	molecular_function unknown
CA4467	1.0	1.0	1.0	IPF9833	13957835..	unknown function	orf19.6864	9833	CaVPH1	PROTEIN I	transporter activity
CA4468	0.9	0.9	0.8	TOR2.53E(	13960608..	phosphatidylinositol 3-l	orf19.1905	9721	IPF9833	No significant S.c. match	
CA4469	0.9	1.0	0.9	TOR2.3F	13962745..	phosphatidylinositol 3-l	orf19.1903	9719	CaTOR2.5	Lipid fatty-acid and isoprenoid metabolism ""CELL CYCLE AND DNA PROCESSING CELLULAR COMMUNICATION/SIGNAL TRANSDUC	
CA4470	1.0	1.0	1.0	IPF9717	13967770..	unknown function	orf19.1902	9717	CaTOR2.3	Lipid fatty-a	protein binding
CA4471	1.0	1.1	1.0	MCM3	13969807..	replication initiation prc	orf19.1901	9716	IPF9717	UNCLASSI	molecular_function unknown
CA4472	1.0	1.0	1.0	IPF6444	13972714..	putative methyltransfer	orf19.1900	6444	CaMCM3	CELL CYC	DNA binding
CA4473	1.0	0.9	1.0	IPF6447	13974538..	unknown function	orf19.1897	6447	IPF6444	UNCLASSI	transferase activity
CA4474	0.8	0.9	0.8	SSC1	13976149..	Mitochondrial heat sho	orf19.1896	6450	IPF6447	UNCLASSIFIED PROTEINS	
CA4475	1.2	1.2	1.0	IPF6455	complemer	unknown function	orf19.1893	6455	CaSSC1	PROTEIN I	enzyme regulator activity
CA4476	1.4	1.0	1.5	01-Apr	complemer	aspartyl protease	orf19.1891	6456	IPF6455	No significant S.c. match	
CA4477	0.9	1.1	1.0	IPF6459	13983877..	unknown function	orf19.1890	6459	CaAPR1	PROTEIN I	peptidase activity
CA4478	1.1	1.1	1.1	IPF6461	13985699..	unknown function	orf19.1889	6461	IPF6459	UNCLASSI	molecular_function unknown
CA4479	1.1	1.1	1.1	URH1	complemer	Uridine ribohydrolase (	orf19.1888	6462	IPF6461	C-compour	molecular_function unknown
CA4480	1.0	0.9	1.0	IPF6464	complemer	putative triacylglycerol	orf19.1887	6464	CaURH1	Nucleotide	hydrolase activity
CA4481	1.0	1.0	1.0	IPF9150	13992547..	similar to Saccharomy	orf19.9441	9150	IPF6464	Lipid fatty-a	molecular_function unknown
CA4482	1.0	1.0	1.0	IPF9154	13993921..	similar to Saccharomy	orf19.1883	9154	IPF9150	PROTEIN S	transcription regulator activity
CA4483	1.1	1.1	1.1	IPF9156	13997689..	unknown function	orf19.1881	9156	IPF9154	SUBCELLU	molecular_function unknown
CA4484	1.0	1.0	1.0	HEM15	14000215..	ferrochelatase precurs	orf19.1880	9158	IPF9156	UNCLASSI	molecular_function unknown
CA4485	0.9	0.9	1.0	IPF9160	complemer	similar to Saccharomy	orf19.1878	9160	CaHEM15	Metabolism	lyase activity
CA4486	1.0	1.0	1.0	IPF9162	14002693..	unknown function	orf19.1877	9162	IPF9160	TRANSCR	transcription regulator activity
CA4487	1.0	0.9	0.9	IPF4039	complemer	similar to Saccharomy	orf19.1876	4039	IPF9162	UNCLASSI	molecular_function unknown
CA4488	0.9	0.9	1.0	MEK1.3F	complemer	serine/threonine protei	orf19.1875	4041	IPF4039	CELL CYC	RNA binding
CA4490	0.8	0.9	0.7	IPF4045	complemer	unknown function	orf19.9428	4045	CaMEK1.3	CELL CYC	protein kinase activity
CA4491	1.1	1.1	1.0	SWR1	14009069..	putative DNA helicase	orf19.9427	4050	IPF4045	No significant S.c. match	
CA4492	1.0	0.9	1.1	RNR22	14015299..	ribonucleoside-diphosp	orf19.1868	4053	CaSWR1	TRANSCR	helicase activity
CA4493	1.0	1.0	1.0	IPF4055	14016968..	unknown function	orf19.1867	4055	CaRNR22	Nucleotide metabolism CELL CYCLE AND DNA PROCESSING SUBCELLULAR LOCALISATION	
CA4494	1.2	1.2	1.2	VMA1	14018455..	vacuolar ATPase V1 d	orf19.1866	4056	IPF4055	CELL RESCUE DEFENSE AND VIRULENCE ""SUBCELLULAR LOCALISATION	
CA4495	1.1	1.1	1.1	IPF4059	complemer	aldehyde dehydrogena	orf19.1865	4059	CaVMA1	No significa	transporter activity
CA4496	1.2	1.2	1.1	IPF4062	complemer	unknown function	orf19.9420	4062	IPF4059	ENERGY (	molecular_function unknown
CA4497	1.1	1.1	1.0	IPF4064	14023894..	unknown function	orf19.9419	4064	IPF4062	UNCLASSI	molecular_function unknown
CA4498	0.8	0.6	0.9	IPF11315	14026958..	unknown function	orf19.8143	11315	IPF4064	No significant S.c. match	
CA4499	1.0	1.0	1.0	IPF11316	complemer	unknown function	orf19.511	11316	IPF11315	CELLULAF	molecular_function unknown
CA4500	0.9	0.9	1.0	IPF11319	14033050..	unknown function	orf19.8141	11319	IPF11316	UNCLASSI	molecular_function unknown
CA4501	1.0	1.1	1.0	QDR1	14035767..	putative antibiotic resis	orf19.8138	16422	IPF11319	UNCLASSIFIED PROTEINS	
CA4502	0.9	1.1	1.0	IPF10391	complemer	Similar to dnaJ protein	orf19.8136	10391	CaQDR1	CELL RES	transporter activity
CA4503	1.1	1.0	1.0	SRV2	complemer	adenylate cyclase-ass	orf19.505	10389	IPF10391	CELL CYC	enzyme regulator activity
CA4504	0.9	0.9	0.9	ARP5	14041534..	actin-related protein (b	orf19.504	10388	CaSRV2	CELLULAF	protein binding
CA4505	1.0	1.2	1.0	NOP2	complemer	nucleolar protein (by h	orf19.501	15042	CaARP5	SUBCELLU	molecular_function unknown
CA4506	1.0	1.0	1.0	GCD10	14046116..	translation initiation fac	orf19.500	15038	CaNOP2	TRANSCR	transferase activity
CA4507	1.0	1.0	0.9	IPF20015	complemer	unknown function	orf19.499	20015	CaGCD10	PROTEIN S	transferase activity
CA4508	1.0	1.2	1.0	NAM9	complemer	mitochondrial ribosoma	orf19.498	8974	IPF20015	UNCLASSI	transferase activity
CA4509	0.9	1.0	1.0	IPF8973	14050477..	unknown function	orf19.497	8973	CaNAM9	PROTEIN S	structural molecule activity
CA4510	1.1	1.1	1.1	IPF8970	14052090..	similar to Saccharomy	orf19.496	8970	IPF8973	UNCLASSI	molecular_function unknown
CA4511	1.0	1.0	1.0	IPF8966	complemer	unknown function	orf19.8124	8966	IPF8970	CELL CYCLE AND DNA PROCESSING SUBCELLULAR LOCALISATION	
CA4512	1.2	2.2	1.6	RPL15B	14057436..	ribosomal protein L15.	orf19.8123	8963	IPF8966	UNCLASSI	transporter activity,RNA binding
CA4513	1.6	1.8	1.2	ADE17	14058839..	5-aminoimidazole-4-ca	orf19.492	14907	CaRPL15B	PROTEIN S	RNA binding
CA4514	0.6	0.4	1.0	IPF20016	14061041..	similar to Saccharomy	orf19.491	20016	CaADE17	Nucleotide	hydrolase activity
CA4515	0.8	0.8	0.9	IPF16652	complemer	unknown function	orf19.490	16652	IPF20016	PROTEIN I	transporter activity
CA4516	1.2	1.1	1.2	IPF18207	14064587..	unknown function	orf19.489	18207	IPF16652	UNCLASSI	transferase activity
CA4517	1.0	1.0	1.0	MEX67	14065491..	poly(A)+RNA binding p	orf19.488	7086	IPF18207	UNCLASSI	molecular_function unknown
CA4518	1.1	1.0	1.1	SPT14	14067741..	N-acetylglucosaminyl-p	orf19.487	7087	CaMEX67	TRANSCR	protein binding,RNA binding
CA4519	1.0	1.0	1.0	IPF7088	complemer	similar to Saccharomy	orf19.486	7088	CaSPT14	C-compour	transferase activity
CA4520	1.0	1.0	1.1	CDC31	14071993..	spindle pole body com	orf19.485	7089	IPF7088	CELL CYC	protein binding
CA4521	1.0	1.1	1.0	MRPL40	14073012..	Putative mitochondrial	orf19.484	7091	CaCDC31	CELL CYC	structural molecule activity

CA4522	1.1	1.0	1.0	RPT4	complemer	26S proteasome regul	orf19.482	7093	CaMRPL4(	PROTEIN !	structural molecule activity
CA4523	1.1	1.1	1.0	GCD1	14075715..	translation initiation fac	orf19.481	7094	CaRPT4	TRANSCR	peptidase activity
CA4524	1.0	1.0	1.0	IPF7097	complemer	unknown function	orf19.480	7097	CaGCD1	SUBCELLU	translation regulator activity
CA4525	1.1	1.0	1.1	SEC22	14078810..	synaptobrevin-type protein transpo		7098	IPF7097	UNCLASSI	molecular_function unknown
CA4526	0.9	0.9	1.0	MON1.3	14079483..	unknown function, 3-pr	orf19.8109	7100	CaSEC22	CELLULAF	transporter activity
CA4527	1.0	1.1	1.0	IPF4398	14082622..	unknown function	orf19.9054	4398	CaMON1.3	UNCLASSI	molecular_function unknown
CA4528	1.0	1.1	1.3	STT3	14085326..	oligosaccharyl transfer	orf19.1478	4396	IPF4398	UNCLASSIFIED PROTEINS	
CA4529	0.8	0.9	0.8	IPF4395	14088050..	unknown function	orf19.1477	4395	CaSTT3	C-compour	transferase activity
CA4530	1.0	0.9	1.0	IME4	complemer	positive transcription fa	orf19.1476	4394	IPF4395	UNCLASSI	molecular_function unknown
CA4531	0.9	1.0	0.9	SLA1	complemer	cytoskeleton assembly	orf19.1474	4392	CaIME4	TRANSCR	transferase activity
CA4532	0.9	0.9	0.9	IPF4386	complemer	unknown function	orf19.1473	4386	CaSLA1	PROTEIN I	protein binding
CA4533	1.0	1.6	0.7	COX4	complemer	cytochrome-c oxidase	orf19.1471	20017	IPF4386	CLASSIFICATION NOT YET CLEAR-CUT	
CA4534	1.5	1.4	2.1	RPS26A	14098607..	ribosomal protein S26.	orf19.1470	4380	CaCOX4	ENERGY S	oxidoreductase activity
CA4535	0.9	1.0	1.0	CDC55	14100236..	B subunit of protein ph	orf19.1468	4377	CaRPS26A	PROTEIN !	structural molecule activity
CA4536	0.7	0.8	0.8	COX13	complemer	cytochrome-c oxidase	orf19.1467	4376	CaCDC55	CELL CYC	protein phosphatase activity
CA4537	1.0	0.9	1.0	YNT2	complemer	suppressor of rna12/yr	orf19.1466	4375	CaCOX13	ENERGY S	enzyme regulator activity
CA4538	1.0	0.9	1.0	IPF20018	complemer	unknown function	orf19.1465	20018	CaYNT2	TRANSCR	hydrolase activity
CA4539	1.0	1.0	1.0	IFQ4	complemer	Unknown function	orf19.1464	6260	IPF20018	UNCLASSIFIED PROTEINS	
CA4540	1.1	1.2	1.1	SMP2	complemer	Involved in plasmid ma	orf19.1462	6259	CaIFQ4	UNCLASSIFIED PROTEINS	
CA4541	0.5	0.7	0.4	IPF6257	complemer	unknown function	orf19.1461	6257	CaSMP2	ENERGY (	molecular_function unknown
CA4542	1.0	1.1	0.9	IPF6255	14111834..	unknown function	orf19.1460	6255	IPF6257	No significant S.c. match	
CA4543	1.1	0.9	0.9	PPE1	complemer	Ribosomal protein of tl	orf19.1459	6254	IPF6255	UNCLASSI	hydrolase activity
CA4544	1.0	0.9	1.0	IPF6252	14115326..	similar to Saccharomy	orf19.1458	6252	CaPPE1	PROTEIN !	hydrolase activity
CA4545	0.9	1.0	1.0	SSL1	14116432..	TFIIH subunit (transcri	orf19.1457	6250	IPF6252	TRANSCR	hydrolase activity
CA4546	0.9	1.0	0.8	SPT5	complemer	Transcription elongatic	orf19.1453	6247	CaSSL1	CELL CYC	transcription regulator activity
CA4547	1.0	1.0	1.0	SRB9	complemer	DNA-directed RNA pol	orf19.1451	6242	CaSPT5	CELL CYC	transcription regulator activity
CA4548	1.0	1.0	1.0	IPF7862	14126680..	unknown function	orf19.1450	7862	CaSRB9	C-compour	transcription regulator activity
CA4549	0.9	0.9	0.9	IPF7863	14128068..	unknown function	orf19.1449	7863	IPF7862	PROTEIN FATE [folding modification destination]	
CA4550	0.9	0.9	1.0	DYN2.3	14129143..	Dynein light chain 1, cytosolic, 3-p		7865	IPF7863	Nitrogen ar	molecular_function unknown
CA4551	1.0	1.0	1.0	APT1	complemer	adenine phosphoribos	orf19.1448	7867	CaDYN2.3	CELLULAF	motor activity
CA4552	1.0	1.0	1.0	IPF7869	14130639..	unknown function	orf19.1447	7869	CaAPT1	Nucleotide	transferase activity
CA4553	1.0	1.0	1.0	CYB1	14136327..	G2/Mitotic-specific cyc	orf19.1446	7873	IPF7869	CONTROL OF CELLULAR ORGANIZATION	
CA4554	0.9	1.0	1.0	IPF7874	14138304..	similar to Saccharomy	orf19.1445	7874	CaCYB1	CELL CYC	protein kinase activity,enzyme regulator activity
CA4555	1.1	1.3	1.2	IPF17555.3	complemer	unknown function, 3-pr	orf19.9019	17555	IPF7874	TRANSCR	molecular_function unknown
CA4556	0.9	1.2	1.1	IPF8044	complemer	similar to Saccharomy	orf19.6866	8044	IPF17555.3	CELLULAF	protein binding
CA4557	0.9	1.0	1.0	IPF8043	14144722..	unknown function	orf19.6867	8043	IPF8044	TRANSCR	RNA binding
CA4558	0.9	0.5	1.0	GRP5	14145467..	dihydroflavonol-4-redu	orf19.6868	8042	IPF8043	UNCLASSIFIED PROTEINS	
CA4559	1.1	1.0	1.1	IPF8041	complemer	putative mitochondrial	orf19.6869	8041	CaGRP5	Metabolism of vitamins cofactors and prosthetic groups	
CA4560	0.9	0.9	0.9	IPF8038	14150732..	unknown function	orf19.6871	8038	IPF8041	PROTEIN FATE [folding modification destination] ""SUBCELLULAR LOCALISATION	
CA4561	1.0	0.9	0.9	IPF8031	complemer	unknown function	orf19.6872	8031	IPF8038	UNCLASSI	molecular_function unknown
CA4562	2.0	1.3	1.5	RPS8A	complemer	ribosomal protein (by h	orf19.6873	6565	IPF8031	SUBCELLU	molecular_function unknown
CA4563	0.9	0.9	1.0	IPF6566	14158941..	unknown function		6566	CaRPS8A	PROTEIN !	structural molecule activity
CA4564	0.7	0.5	1.0	IPF6572	14165740..	unknown function	orf19.6874	6572	IPF6566	UNCLASSI	molecular_function unknown
CA4565	0.7	0.6	0.8	VPS35	14168313..	Protein-sorting protein,	orf19.6875	6573	IPF6572	CELL CYCLE AND DNA PROCESSING	
CA4566	1.4	1.7	1.1	IPF19801	14172755..	unknown function	orf19.6877	19801	CaVPS35	PROTEIN I	molecular_function unknown
CA4567	1.0	0.9	1.0	IPF9555	complemer	unknown function	orf19.6879	9555	IPF19801	No significant S.c. match	
CA4568	1.0	1.1	1.0	IPF9554	14177046..	unknown function	orf19.6880	9554	IPF9555	TRANSCR	RNA binding
CA4569	0.9	1.0	1.0	IPF9552	complemer	unknown function	orf19.6881	9552	IPF9554	UNCLASSI	molecular_function unknown
CA4570	3.3	2.4	2.1	IPF9550	14181143..	similar to Saccharomy	orf19.6882	9550	IPF9552	TRANSCR	RNA binding
CA4571	1.0	1.2	1.1	RPL33.3	complemer	ribosomal protein L35a, 3-prime er		9548	IPF9550	ENERGY "	oxidoreductase activity
CA4572	1.2	1.1	1.1	KES1	complemer	involved in ergosterol t	orf19.6883	9434	CaRPL33.3	PROTEIN !	structural molecule activity
CA4573	1.1	1.0	1.0	IPF9431	complemer	unknown function	orf19.6884	9431	CaKES1	Lipid fatty-acid and isoprenoid metabolism ""CELLULAR TRANSPORT AND TRANSPORT MECHANISMS SUBCELLULAR LOCALISATIC	
CA4574	1.0	1.0	1.0	IPF9430	14188237..	similar to Saccharomy	orf19.6885	9430	IPF9431	UNCLASSI	molecular_function unknown
CA4575	0.9	0.9	0.9	IPF9428	14190289..	unknown function	orf19.6886	9428	IPF9430	CELL CYC	molecular_function unknown
CA4576	1.0	0.9	0.9	IPF9425.3	14194146..	unknown function, 3-pr	orf19.6888	9425	IPF9428	UNCLASSI	molecular_function unknown
CA4577	1.1	1.3	1.0	MKK2	complemer	Protein kinase of MEK	orf19.6889	14309	IPF9425.3	No significant S.c. match	
CA4578	0.9	0.9	1.0	RFC1	complemer	DNA replication factor	orf19.6891	14308	CaMKK2	CELL CYC	protein kinase activity
CA4579	1.0	0.9	1.0	IPF2234	14220274..	unknown function	orf19.6898	2234	CaRFC1	CELL CYCLE AND DNA PROCESSING SUBCELLULAR LOCALISATION	
CA4580	0.7	0.5	0.8	IPF2233	complemer	unknown function		2233	IPF2234	No significant S.c. match	
CA4581	1.1	1.1	1.0	IPF2232	complemer	unknown function	orf19.6899	2232	IPF2233	No significant S.c. match	
CA4582	1.0	0.9	1.0	MDM12	14224083..	Involved in mitochondr	orf19.6900	2231	IPF2232	UNCLASSIFIED PROTEINS	
CA4583	1.0	1.0	1.1	IPF2229	14225693..	unknown function	orf19.6901	2229	CaMDM12	SUBCELLU	molecular_function unknown
CA4584	1.1	1.2	1.0	IPF2228	complemer	similar to Saccharomy	orf19.6902	2228	IPF2229	UNCLASSI	molecular_function unknown
CA4585	0.8	0.8	0.9	IPF2227	complemer	unknown function	orf19.6903	2227	IPF2228	TRANSCR	RNA binding,helicase activity
CA4586	1.0	1.0	1.1	GCN3	14230237..	Translation initiation fa	orf19.6904	2224	IPF2227	TRANSCR	nucleotidyltransferase activity
CA4587	1.0	0.9	1.0	IPF2223	complemer	unknown function	orf19.6905	2223	CaGCN3	PROTEIN !	translation regulator activity
CA4588	1.0	1.1	1.0	BEL1.EXO	14233745..	protein of the 40S ribo	orf19.6906	2221	IPF2223	UNCLASSI	molecular_function unknown
CA4589	1.0	1.5	1.2	BEL1.EXO	14234532..	protein of the 40S ribosomal subu		2220	CaBEL1.ex	ENERGY F	molecular_function unknown
CA4590	0.9	0.9	1.0	IPF2218	complemer	unknown function	orf19.6907	2218	CaBEL1.ex	ENERGY PROTEIN SYNTHESIS SUBCELLULAR LOCALISATION	
CA4591	0.9	0.9	0.9	IPF2216	complemer	putative folylpolyglutan	orf19.6908	2216	IPF2218	UNCLASSI	molecular_function unknown
CA4592	0.9	1.1	1.0	IPF2215	14239185..	unknown function	orf19.6909	2215	IPF2216	Metabolisr	ligase activity
CA4593	0.9	0.9	1.0	IPF2214	complemer	unknown function	orf19.6910	2214	IPF2215	TRANSCR	transcription regulator activity

CA4594	1.0	0.9	0.9	IPF2212	14241952..	unknown function	orf19.6912	2212	IPF2214	UNCLASSI	DNA binding
CA4595	0.9	1.1	1.0	GCN2	complemer	Ser/thr protein kinase (	orf19.6913	2209	IPF2212	Lipid fatty-acid and isoprenoid metabolism ""SUBCELLULAR LOCALISATION	
CA4596	1.0	1.1	0.9	MRE11	14249502..	DNA repair and meioti	orf19.6915	2205	CaGCN2	PROTEIN	protein kinase activity
CA4597	1.0	0.9	0.9	ATP11	complemer	F1F0-ATPase comple	orf19.6916	2203	CaMRE11	CELL CYC	protein binding
CA4598	1.0	0.9	0.9	IPF20019	complemer	unknown function	orf19.6917	20019	CaATP11	PROTEIN I	chaperone activity
CA4599	0.9	0.9	1.0	IPF2200	14253886..	Unknown function	orf19.6918	2200	IPF20019	UNCLASSI	molecular_function unknown
CA4600	1.0	1.0	1.1	IPF2199	14257208..	unknown function	orf19.6919	2199	IPF2200	No significant S.c. match	
CA4601	1.0	1.0	1.0	IPF2195	14261733..	unknown function	orf19.6920	2195	IPF2199	No significant S.c. match	
CA4602	1.1	1.1	1.0	IPF6231	14266071..	unknown function	orf19.1080	6231	IPF2195	No significant S.c. match	
CA4603	1.0	1.1	1.1	IPF6230	complemer	unknown function	orf19.1080	6230	IPF6231	TRANSCR	oxidoreductase activity
CA4604	1.4	1.2	1.1	MBF1	14267840..	Multiprotein bridging fa	orf19.3294	6228	IPF6230	Lipid fatty-a	oxidoreductase activity
CA4605	1.0	1.0	1.0	IPF6226	complemer	unknown function	orf19.3295	6226	CaMBF1	TRANSCR	transcription regulator activity
CA4606	0.9	0.9	0.9	IPF6224	14270429..	unknown function	orf19.3296	6224	IPF6226	UNCLASSIFIED PROTEINS	
CA4607	0.9	1.0	1.0	IPF6223	complemer	unknown function	orf19.3297	6223	IPF6224	No significant S.c. match	
CA4608	1.0	1.0	1.0	CCH1	14273192..	Calcium channel prote	orf19.3298	6222	IPF6223	UNCLASSI	structural molecule activity
CA4609	1.0	0.9	1.0	ZPR1	complemer	Zinc finger protein (by	orf19.3300	6215	CaCCH1	REGULATI	transporter activity
CA4610	0.9	0.9	0.9	IPF19802	14284224..	similar to Saccharomy	orf19.3301	19802	CaZPR1	UNCLASSI	protein binding
CA4611	1.0	0.9	0.9	IPF10727	complemer	unknown function	orf19.3302	10727	IPF19802	Amino acid	protein binding
CA4612	1.1	0.9	1.1	PPM2	complemer	carboxy methyl transfe	orf19.3303	10724	IPF10727	C-compound and carbohydrate metabolism ENERGY SUBCELLULAR LOCALISATION	
CA4613	0.9	0.9	0.9	IPF7950	complemer	similar to Saccharomy	orf19.3304	7950	CaPPM2	PROTEIN I	transferase activity
CA4614	0.9	0.9	1.0	IPF7952	complemer	unknown function	orf19.3305	7952	IPF7950	CELL CYC	RNA binding
CA4615	1.0	1.0	0.9	IPF7955	complemer	DNA binding protein (b	orf19.3306	7955	IPF7952	Lipid fatty-acid and isoprenoid metabolism ""TRANSCRIPTION	
CA4616	0.8	0.9	1.1	IPF5761	complemer	flavin-containing mono	orf19.3307	5761	IPF7955	CELL CYC	DNA binding
CA4617	1.0	1.0	1.1	STB5	14302298..	SIN3 binding protein (t	orf19.3308	5760	IPF5761	Nitrogen ar	oxidoreductase activity
CA4618	1.0	1.0	1.0	IPF5757	14304763..	unknown function	orf19.3309	5757	CaSTB5	TRANSCR	DNA binding,transcription regulator activity
CA4619	1.0	1.0	0.9	IPF5756	complemer	unknown function	orf19.3310	5756	IPF5757	No significant S.c. match	
CA4620	1.0	1.0	1.0	IFD3	14308373..	OXIDOREDUCTASE (	orf19.3311	5754	IPF5756	No significant S.c. match	
CA4621	1.0	1.1	1.0	IPF5753	14309660..	unknown function	orf19.3312	5753	CaIFD3	C-compound and carbohydrate metabolism ENERGY	
CA4622	1.0	1.1	1.0	IPF5751	complemer	TRAPP subunit of 20 k	orf19.3314	5751	IPF5753	UNCLASSI	molecular_function unknown
CA4623	0.9	0.9	1.0	CTA9	14312715..	Putative transcriptional	orf19.3315	5750	IPF5751	CELLULAF	molecular_function unknown
CA4624	1.0	0.9	0.9	IPF5747	complemer	unknown function	orf19.3318	5747	CaCTA9	No significant S.c. match	
CA4625	1.1	1.2	1.0	IPF5742	14317478..	thioredoxin-like protein	orf19.3319	5742	IPF5747	UNCLASSI	molecular_function unknown
CA4626	1.0	1.0	1.0	MRS7	14319204..	suppressor splicing de	orf19.1083	5739	IPF5742	CELL CYCLE AND DNA PROCESSING ""PROTEIN FATE [folding modification destination] """"CELL RESCUE DEFENSE AND VIRULEN	
CA4627	1.1	1.0	1.0	DUT1	complemer	dUTP pyrophosphatas	orf19.1083	5736	CaMRS7	UNCLASSI	molecular_function unknown
CA4628	1.2	1.2	1.2	IPF13704	14324792..	unknown function		13704	CaDUT1	Nucleotide	hydrolase activity
CA4629	1.0	1.0	1.0	IPF13709	14329052..	unknown function	orf19.335	13709	IPF13704	No significant S.c. match	
CA4630	0.9	0.8	1.1	IPF1537	complemer	putative adrenodoxin a	orf19.336	1537	IPF13709	CELL CYCLE AND DNA PROCESSING ""PROTEIN FATE [folding modification destination] ""SUBCELLULAR LOCALISATION	
CA4631	1.0	1.1	0.9	VTI1	complemer	v-SNARE involved in C	orf19.337	1535	IPF1537	CLASSIFIC	transporter activity
CA4632	1.0	1.3	1.1	IPF1531	complemer	unknown function	orf19.338	1531	CaVTI1	PROTEIN I	transporter activity
CA4633	1.1	1.3	0.9	NDH1	complemer	Mitochondrial NADH d	orf19.339	1529	IPF1531	UNCLASSI	molecular_function unknown
CA4634	1.0	1.0	1.0	IPF1526	complemer	unknown function	orf19.340	1526	CaNDH1	ENERGY S	transporter activity
CA4635	1.0	0.9	1.0	IPF1524	complemer	putative multidrug resis	orf19.341	1524	IPF1526	TRANSCR	molecular_function unknown
CA4636	1.1	1.1	1.1	IPF1520	complemer	unknown function	orf19.342	1520	IPF1524	CELL RESCUE DEFENSE AND VIRULENCE ""TRANSPORT FACILITATION	
CA4637	1.0	1.1	1.1	IPF1514	complemer	unknown function	orf19.344	1514	IPF1520	No significant S.c. match	
CA4638	1.1	1.0	1.0	UGA2	14352333..	succinate-semialdehyc	orf19.345	1510	IPF1514	No significant S.c. match	
CA4639	1.0	0.9	0.9	IPF1509	complemer	putative alanine transa	orf19.346	1509	CaUGA2	Amino acid	oxidoreductase activity
CA4640	1.0	1.0	1.0	IPF1506	14358098..	unknown function	orf19.347	1506	IPF1509	Amino acid	transferase activity
CA4641	0.9	0.9	1.0	IPF1505	14360691..	similar to saccharomyc	orf19.348	1505	IPF1506	No significant S.c. match	
CA4642	1.0	1.0	1.0	IPF1500	complemer	similar to Saccharomy	orf19.349	1500	IPF1505	C-compound and carbohydrate metabolism CELL FATE CONTROL OF CELLULAR ORGANIZATION SUBCELLULAR LOCALISATION	
CA4643	1.0	1.0	1.0	PRE9	complemer	20S proteasome subur	orf19.350	1499	IPF1500	CONTROL	molecular_function unknown
CA4644	1.1	1.0	1.0	IPF1497	14366875..	unknown function	orf19.351	1497	CaPRE9	PROTEIN I	peptidase activity
CA4645	1.0	1.0	1.0	IPF1496	14367588..	unknown function	orf19.352	1496	IPF1497	UNCLASSI	molecular_function unknown
CA4646	1.1	1.1	1.0	IPF1495	complemer	unknown function	orf19.353	1495	IPF1496	UNCLASSIFIED PROTEINS	
CA4647	1.1	1.1	1.1	IPF1493	14370664..	similarity to E.coli X-Pr	orf19.354	1493	IPF1495	PROTEIN FATE [folding modification destination]	
CA4648	1.0	1.0	0.9	IPF9864	complemer	similar to Saccharomy	orf19.1191	9864	IPF1493	PROTEIN I	peptidase activity
CA4649	1.0	1.0	1.0	GPX4	14386219..	glutathione peroxidase	orf19.4436	9860	IPF9864	SUBCELLU	structural molecule activity
CA4650	1.0	1.0	1.0	ISW1.3	complemer	ATPase component of	orf19.1191	9859	CaGPX4	CELL RESCUE DEFENSE AND VIRULENCE	
CA4651	1.0	1.0	1.0	IPF3121	complemer	unknown function	orf19.1191	3121	CaISW1.3	TRANSCR	hydrolase activity
CA4652	1.0	0.9	0.9	IPF3105	complemer	Unknown function	orf19.4439	3105	IPF3121	CELL CYC	transcription regulator activity
CA4653	1.0	0.9	1.0	IPF3102	complemer	similar to Saccharomy	orf19.4440	3102	IPF3105	No significant S.c. match	
CA4654	1.0	0.9	1.0	IPF3101	14418332..	unknown function	orf19.4441	3101	IPF3102	CELLULAF	transporter activity
CA4655	0.9	0.9	1.0	IPF3098	14420313..	Putative mannosyltran	orf19.4442	3098	IPF3101	CELL CYC	DNA binding
CA4656	1.0	0.9	1.0	IPF3095	complemer	unknown function	orf19.4443	3095	IPF3098	C-compour	transferase activity
CA4657	1.1	1.0	1.0	IPF3094	complemer	4-nitrophenyl phospha	orf19.4444	3094	IPF3095	REGULATI	transferase activity
CA4658	1.0	1.0	0.9	IPF3092	complemer	unknown function	orf19.4445	3092	IPF3094	Phosphate	hydrolase activity
CA4659	0.9	1.0	1.0	IPF17754	14428084..	low affinity high capaci	orf19.4446	17754	IPF3092	No significant S.c. match	
CA4660	0.9	1.0	1.0	YMC1	complemer	mitochondrial carrier p	orf19.4447	6563	IPF17754	CELLULAR TRANSPORT AND TRANSPORT MECHANISMS SUBCELLULAR LOCALISATION TRANSPORT FACILITATION	
CA4661	1.0	1.0	1.0	IPF6561	complemer	unknown function	orf19.4448	6561	CaYMC1	CELLULAF	transporter activity
CA4662	1.1	1.3	1.1	LYS7	complemer	Copper chaperone for	orf19.4449	6557	IPF6561	UNCLASSI	molecular_function unknown
CA4663	1.0	1.0	1.0	IPF6554	14437834..	unknown function	orf19.4450	6554	CaLYS7	PROTEIN I	chaperone activity
CA4665	1.1	1.0	1.1	IPF6548	14440757..	translation elongation f	orf19.4451	6548	IPF6554	TRANSCR	molecular_function unknown
CA4666	0.9	1.0	0.9	IPF6542	14445472..	unknown function	orf19.4455	6542	IPF6548	PROTEIN	translation regulator activity

CA4667	1.2	1.6	1.5	GAP5	complemer	General amino acid pe	orf19.4456	6541	IPF6542	UNCLASSI	molecular_function unknown
CA4668	1.1	1.3	1.1	ROM2	complemer	GDP/GTP exchange fa	orf19.906	4544	CaGAP5	Amino acid metabolism CELLULAR TRANSPORT AND TRANSPORT MECHANISMS SUBCELLULAR LOCALISATION TRANSPORT FA	
CA4669	1.0	1.0	1.0	IPF4537	14458495..	putative permease (by	orf19.905	4537	CaROM2	C-compour	signal transducer activity
CA4670	1.1	1.0	1.0	IPF4536	14460038..	unknown function	orf19.904	4536	IPF4537	TRANSPO	transporter activity
CA4671	7.3	4.8	3.1	GPM1	14461584..	phosphoglycerate muta	orf19.903	4535	IPF4536	Amino acid metabolism SUBCELLULAR LOCALISATION	
CA4672	1.4	2.3	1.1	IPF3659	14462809..	similar to Saccharomy	orf19.900	3659	CaGPM1	C-compour	isomerase activity
CA4673	1.1	1.1	1.1	IPF3661	14465338..	unknown function	orf19.899	3661	IPF3659	TRANSCR	structural molecule activity
CA4674	0.9	1.0	0.8	HEM2	14466704..	Porphobilinogen synth	orf19.898	3663	IPF3661	No significant S.c. match	
CA4675	0.7	0.6	0.8	IPF3664	complemer	unknown function	orf19.897	3664	CaHEM2	Metabolism	lyase activity
CA4676	1.0	1.0	1.0	HK1	14469675..	Histidine kinase	orf19.896	3668	IPF3664	CLASSIFIC	molecular_function unknown
CA4677	0.5	0.3	0.9	HOG1	complemer	Ser/thr protein kinase (	orf19.895	3669	CaHK1	C-compound and carbohydrate metabolism CELLULAR COMMUNICATION/SIGNAL TRANSDUCTION MECHANISM ""CELL RESCUE DE	
CA4678	1.1	1.0	1.0	IPF3670	14478750..	unknown function	orf19.894	3670	CaHOG1	C-compour	protein kinase activity,signal transducer activity
CA4679	1.0	1.0	1.0	IPF3674	14481512..	unknown function	orf19.893	3674	IPF3670	CELL CYC	enzyme regulator activity
CA4680	1.1	1.2	1.1	AMD1	14483896..	AMP deaminase (by ho	orf19.891	3677	IPF3674	No significant S.c. match	
CA4681	1.1	1.0	1.1	IPF3679	14486567..	similar to Saccharomy	orf19.889	3679	CaAMD1	Nucleotide	hydrolase activity
CA4682	1.2	1.4	1.1	IPF3687	14488504..	similar to Saccharomy	orf19.886	3687	IPF3679	TRANSCR	transferase activity
CA4683	1.0	1.0	0.9	HSP78.3F	complemer	heat shock protein of c	orf19.884	9007	IPF3687	PROTEIN I	protein binding
CA4684	1.0	1.1	0.9	HSP78.5F	complemer	heat shock protein of c	orf19.882	9010	CaHSP78.3	PROTEIN FATE [folding modification destination] """"CELL RESCUE DEFENSE AND VIRULENCE ""SUBCELLULAR LOCALISATION	
CA4685	1.0	1.1	1.1	IPF9013	complemer	unknown function	orf19.881	9013	CaHSP78.5	PROTEIN FATE [folding modification destination] """"CELL RESCUE DEFENSE AND VIRULENCE ""SUBCELLULAR LOCALISATION	
CA4686	1.0	1.0	1.0	IPF9015	14498374..	unknown function	orf19.880	9015	IPF9013	CELL FATE	molecular_function unknown
CA4687	0.9	1.0	1.0	IPF9017	complemer	similar to Saccharomy	orf19.879	9017	IPF9015	UNCLASSI	molecular_function unknown
CA4688	1.0	1.1	1.0	NBN1	14503979..	involved in chromatin r	orf19.878	9018	IPF9017	CELLULAR	protein binding
CA4689	1.1	1.3	1.1	IPF9020	14505254..	unknown function	orf19.8495	9020	CaNBN1	SUBCELLU	enzyme regulator activity
CA4690	0.9	1.0	1.0	IPF3959	14506309..	unknown function	orf19.8494	3959	IPF9020	No significant S.c. match	
CA4691	0.9	0.8	0.9	IPF3958	complemer	unknown function	orf19.874	3958	IPF3959	No significant S.c. match	
CA4692	1.1	1.1	1.1	COX6.3	complemer	cytochrome-c oxidase subunit VI,		3955	IPF3958	UNCLASSIFIED PROTEINS	
CA4693	1.1	1.0	1.0	IPF3952	complemer	unknown function	orf19.872	3952	CaCOX6.3	ENERGY S	oxidoreductase activity
CA4694	1.0	1.0	1.0	IPF3950	14514082..	unknown function	orf19.8490	3950	IPF3952	No significant S.c. match	
CA4695	0.9	1.0	0.9	IPF4229	complemer	unknown function	orf19.6923	4229	IPF3950	CELL CYCLE AND DNA PROCESSING TRANSCRIPTION SUBCELLULAR LOCALISATION	
CA4696	1.1	1.0	1.7	HTA1	complemer	Histone H2A (by hom	orf19.6924	4226	IPF4229	TRANSCR	transcription regulator activity
CA4697	2.0	1.4	1.4	HTB1	14527408..	Histone H2B (by homo	orf19.6925	4223	CaHTA1	TRANSCR	DNA binding
CA4698	1.0	1.0	0.9	CDC25	complemer	cell division cycle prote	orf19.6926	4222	CaHTB1	TRANSCR	DNA binding
CA4699	0.9	1.0	0.9	IPF4220	14532785..	similar to Saccharomy	orf19.6927	4220	CaCDC25	Nitrogen ar	enzyme regulator activity
CA4700	1.1	1.2	1.0	SAP9	14535383..	aspartyl proteinase 9 (l	orf19.6928	4215	IPF4220	PROTEIN I	molecular_function unknown
CA4701	1.1	1.2	1.1	IPF4214	complemer	unknown function	orf19.6929	4214	CaSAP9	PROTEIN I	peptidase activity
CA4702	1.1	0.8	1.2	IPF4213	14538736..	unknown function	orf19.6930	4213	IPF4214	UNCLASSI	molecular_function unknown
CA4703	1.2	1.1	1.2	CLP1	14539673..	probable cleaveage/po	orf19.6931	4212	IPF4213	No significant S.c. match	
CA4704	1.1	1.2	1.0	RRD2	complemer	hosphotyrosyl phospha	orf19.6933	4207	CaCLP1	TRANSCR	RNA binding
CA4705	1.0	0.4	1.1	IPF4206	14542348..	unknown function	orf19.6934	4206	CaRRD2	UNCLASSI	protein phosphatase activity
CA4706	1.2	1.1	1.2	RAD53	complemer	protein kinase	orf19.6936	4205	IPF4206	CELL RESCUE DEFENSE AND VIRULENCE	
CA4707	0.9	1.2	1.0	PTR21	complemer	peptide transporter	orf19.6937	4202	CaRAD53	CELL CYC	protein kinase activity
CA4708	1.0	1.0	1.0	MEU1	complemer	regulator of ADH2 expr	orf19.6938	5890	CaPTR21	CELLULAR	transporter activity
CA4709	1.0	1.2	0.9	IPF5895	complemer	unknown function	orf19.6941	5895	CaMEU1	Nucleotide	molecular_function unknown
CA4710	1.1	1.1	1.1	ORC3	14556103..	Origin recognition com	orf19.6942	5897	IPF5895	UNCLASSI	transferase activity
CA4711	1.2	1.4	1.2	PHB1	14559208..	Prohibitin, antiprolifera	orf19.6944	5903	CaORC3	CELL CYC	DNA binding
CA4712	1.2	1.0	1.2	GTT1.3	complemer	glutathione S-transfera	orf19.6947	5904	CaPHB1	CELL CYC	molecular_function unknown
CA4713	1.1	1.0	1.0	CCC1	complemer	Transmembrane Ca2+	orf19.6948	5909	CaGTT1.3	CELL RES	transferase activity
CA4714	0.9	0.9	1.0	IPF5912	complemer	unknown function	orf19.6950	5912	CaCCC1	REGULATI	molecular_function unknown
CA4715	0.7	0.7	0.8	DPL1	complemer	dihydrosphingosine ph	orf19.6951	16613	IPF5912	UNCLASSI	molecular_function unknown
CA4716	1.1	1.0	1.0	IPF8666	complemer	unknown function	orf19.6952	8666	CaDPL1	Lipid fatty-a	lyase activity
CA4717	1.0	0.8	0.9	IPF8663	complemer	Unknown function	orf19.6953	8663	IPF8666	UNCLASSI	molecular_function unknown
CA4718	1.1	1.0	1.0	IPF8661	complemer	unknown function	orf19.6955	8661	IPF8663	UNCLASSI	molecular_function unknown
CA4719	1.0	0.9	1.1	DAL51	complemer	allantoate permease (b	orf19.6956	8658	IPF8661	UNCLASSI	chaperone activity
CA4720	1.0	1.0	1.0	ECM18	complemer	Involved in cell wall bic	orf19.6958	8656	CaDAL51	CELLULAR	transporter activity
CA4721	1.0	1.1	1.0	CPP1	14583857..	probable protein-tyrosi	orf19.1233	15616	CaECM18	CONTROL	molecular_function unknown
CA4722	0.9	1.0	1.0	SAC1	complemer	integral membrane pro	orf19.1232	15617	CaCPP1	CELL CYC	protein phosphatase activity
CA4723	1.1	1.1	1.0	IPF18177	14589045..	Unknown function	orf19.1232	18177	CaSAC1	CELLULAR	hydrolase activity
CA4724	0.8	0.9	0.8	PDC2	14590781..	pyruvate decarboxylas	orf19.1232	16151	IPF18177	UNCLASSI	molecular_function unknown
CA4725	0.9	1.0	1.0	PET100	complemer	cytochrome-c oxidase assembly p		16152	CaPDC2	C-compour	transcription regulator activity
CA4726	1.0	1.0	1.0	IPF12255	14594135..	unknown function	orf19.1232	12255	CaPET100	ENERGY "	chaperone activity
CA4727	1.1	0.9	1.0	IPF12253	complemer	unknown function		12253	IPF12255	CLASSIFIC	molecular_function unknown
CA4728	1.0	1.0	1.0	VPS41.3F	complemer	required for the vacuol	orf19.1232	12250	IPF12253	No significant S.c. match	
CA4729	0.9	0.9	0.9	VPS41.5F	complemer	required for the vacuol	orf19.1232	12249	CaVPS41.3	PROTEIN I	enzyme regulator activity
CA4730	1.1	1.0	1.0	IPF7737	14600420..	unknown function	orf19.1232	7737	CaVPS41.5	PROTEIN FATE [folding modification destination] ""SUBCELLULAR LOCALISATION	
CA4731	0.9	0.9	1.0	LIP3	complemer	Secretory lipase	orf19.4856	7736	IPF7737	No significant S.c. match	
CA4732	0.9	1.0	1.0	IPF7733	14604926..	unknown function	orf19.4855	7733	CaLIP3	Other virulence attributes	
CA4733	1.0	0.9	1.0	IPF7732	complemer	similar to Saccharomy	orf19.4853	7732	IPF7733	CLASSIFIC	molecular_function unknown
CA4734	1.1	1.1	1.1	TFA1	14608968..	Large subunit of transc	orf19.4851	7729	IPF7732	TRANSCR	transcription regulator activity
CA4735	1.1	1.1	1.1	IPF7726	14610999..	unknown function	orf19.4850	7726	CaTFA1	TRANSCR	transcription regulator activity
CA4736	1.0	1.1	1.1	IPF4503	14613866..	unknown function	orf19.4849	4503	IPF7726	UNCLASSI	molecular_function unknown
CA4737	0.9	0.9	1.0	SKI3	complemer	antiviral protein	orf19.4848	4502	IPF4503	TRANSCR	molecular_function unknown
CA4738	0.8	0.8	0.9	IPF4500	complemer	putative GTP-binding p	orf19.4846	4500	CaSKI3	CELL RES	translation regulator activity

CA4739	1.0	1.0	1.2	IPF4498	14621356..	unknown function	orf19.4845	4498	IPF4500	UNCLASSI	molecular_function unknown
CA4740	1.0	0.9	0.8	IPF4497	14622263..	unknown function	orf19.4844	4497	IPF4498	UNCLASSI	molecular_function unknown
CA4741	0.9	1.0	0.9	IPF4496	14623971..	unknown function	orf19.4843	4496	IPF4497	UNCLASSI	molecular_function unknown
CA4742	0.8	1.0	1.0	SHY1	14627032..	SURF homologue prot	orf19.4841	4494	IPF4496	UNCLASSI	oxidoreductase activity
CA4743	1.0	1.0	1.0	IPF4491	complemer	unknown function	orf19.4839	4491	CaSHY1	ENERGY S	chaperone activity
CA4744	0.9	0.9	1.0	IPF4489	complemer	unknown function	orf19.4837	4489	IPF4491	UNCLASSI	molecular_function unknown
CA4745	1.0	0.9	1.0	URA1	complemer	dihydroorotate dehydro	orf19.4836	4487	IPF4489	CELL CYC	structural molecule activity
CA4746	0.9	1.0	0.9	IPF4485	14632338..	unknown function	orf19.4835	4485	CaURA1	No significant S.c. match	
CA4747	1.0	0.9	1.8	IPF4484	complemer	unknown function	orf19.4834	4484	IPF4485	UNCLASSI	RNA binding
CA4748	0.2	0.2	0.2	MLS1	complemer	malate synthase	orf19.4833	4483	IPF4484	C-compound and carbohydrate metabolism	
CA4749	1.0	0.8	1.2	IPF4481	complemer	unknown function	orf19.4831	4481	CaMLS1	C-compour	transferase activity
CA4750	1.0	1.0	0.9	IPF4477	complemer	similar to Saccharomy	orf19.4829	4477	IPF4481	No significant S.c. match	
CA4751	0.9	1.0	1.0	IPF11473	complemer	unknown function	orf19.4828	11473	IPF4477	Lipid fatty-a	molecular_function unknown
CA4752	0.8	0.8	0.8	ADE12	complemer	adenylosuccinate synt	orf19.4827	11474	IPF11473	No significant S.c. match	
CA4753	0.9	1.6	1.1	IDH1.3	complemer	isocitrate dehydrogena	orf19.4826	11475	CaADE12	Nucleotide	ligase activity
CA4754	0.8	0.8	0.8	IPF11479	14649594..	unknown function	orf19.4825	11479	CaIDH1.3	C-compour	oxidoreductase activity
CA4755	0.9	1.0	1.1	IPF11480	complemer	unknown function	orf19.4824	11480	IPF11479	Metabolism	molecular_function unknown
CA4756	0.9	1.0	1.0	LIP6	14653322..	Secretory lipase	orf19.4823	11788	IPF11480	No significant S.c. match	
CA4757	1.0	0.9	1.0	LIP10	14655761..	Secretory lipase	orf19.4822	11790	CaLIP6	Other virulence attributes	
CA4758	1.0	0.9	0.9	PPR1	complemer	transcription factor reg	orf19.3986	9661	CaLIP10	Other virulence attributes	
CA4759	1.0	1.0	1.0	IPF9655	14694521..	unknown function	orf19.3988	9655	CaPPR1	Nucleotide	DNA binding,transcription regulator activity
CA4760	1.0	0.9	0.9	IPF9652	14696524..	unknown function	orf19.3990	9652	IPF9655	No significant S.c. match	
CA4761	0.9	1.1	1.0	IPF9650	complemer	lipase family protein cc	orf19.3991	9651	IPF9652	UNCLASSI	nucleotidyltransferase activity
CA4762	1.1	1.0	1.0	IPF9647	complemer	similar to Saccharomy	orf19.3994	9647	IPF9650	Lipid fatty-a	hydrolase activity
CA4763	0.9	1.0	1.0	IPF9645	complemer	similar to Saccharomy	orf19.3995	9645	IPF9647	C-compour	transferase activity
CA4764	1.0	0.9	0.9	GPI10	14703600..	required for Glycosyl P	orf19.3996	19803	IPF9645	PROTEIN I	peptidase activity
CA4765	2.0	0.8	2.6	ADH1	14706501..	alcohol dehydrogenase	orf19.3997	17060	CaGPI10	Lipid fatty-a	molecular_function unknown
CA4766	1.1	1.1	1.0	IPF13056	complemer	unknown function	orf19.3998	13056	CaADH1	C-compound and carbohydrate metabolism SUBCELLULAR LOCALISATION	
CA4767	1.0	1.0	1.0	IPF13054	14708705..	unknown function	orf19.3999	13054	IPF13056	No significant S.c. match	
CA4768	1.0	1.1	1.0	IPF9385	14717907..	similar to Saccharomy	orf19.4000	9385	IPF13054	UNCLASSIFIED PROTEINS	
CA4769	1.0	1.0	0.9	IPF9384	14720421..	similar to Saccharomy	orf19.4001	9384	IPF9385	Nucleotide	DNA binding,transcription regulator activity
CA4770	1.0	1.0	1.0	IPF9382.3	complemer	similar to Saccharomy	orf19.4002	9382	IPF9384	ENERGY	
CA4771	1.0	0.9	1.0	IPF3336	complemer	unknown function	orf19.4003	3336	IPF9382.3	CELL CYC	protein kinase activity
CA4772	0.7	0.6	0.9	CCT3	14726652..	Chaperonin (by homol	orf19.4004	3339	IPF3336	CELLULAF	molecular_function unknown
CA4773	0.9	0.9	1.0	IPF3340	complemer	unknown function	orf19.4005	3340	CaCCT3	PROTEIN I	chaperone activity
CA4774	1.0	1.0	1.1	IPF3341	14729142..	Unknown function	orf19.4006	3341	IPF3340	UNCLASSI	transferase activity
CA4775	1.0	1.0	1.0	IPF3342	complemer	Unknown function	orf19.4007	3342	IPF3341	Metabolism	oxidoreductase activity
CA4776	1.1	1.1	1.1	CNB1	complemer	Protein phosphatase, (	orf19.4009	3344	IPF3342	UNCLASSI	molecular_function unknown
CA4777	1.0	0.9	0.9	PAN3	14732698..	component of the Pab	orf19.4010	3345	CaCNB1	TRANSCR	protein phosphatase activity
CA4778	1.1	1.1	1.0	IFJ1	14736026..	Unknown function	orf19.4011	3348	CaPAN3	TRANSCR	RNA binding
CA4779	1.0	1.0	1.1	IPF3351	14738137..	unknown function	orf19.4012	3351	CaIFJ1	UNCLASSIFIED PROTEINS	
CA4780	0.9	1.0	1.0	IPF3352	complemer	unknown function	orf19.4013	3352	IPF3351	UNCLASSI	protein kinase activity,enzyme regulator activity
CA4781	0.9	0.9	1.0	IPF3355	14740761..	similar to Saccharomy	orf19.4014	3355	IPF3352	UNCLASSI	molecular_function unknown
CA4783	0.9	0.9	0.6	IPF3358	14742404..	ubiquinol-cytochrome-c	orf19.4016	3358	IPF3355	CELL CYCLE AND DNA PROCESSING REGULATION OF/INTERACTION WITH CELLULAR ENVIRONMENT CELL FATE SUBCELLULA	
CA4784	1.0	1.0	0.9	IPF3359	complemer	Unknown function	orf19.4017	3359	IPF3358	ENERGY S	transporter activity,oxidoreductase activity
CA4785	1.2	1.0	1.2	IPF3361	complemer	putative mitochondrial	orf19.4018	3361	IPF3359	No significant S.c. match	
CA4786	0.9	0.8	1.0	IPF3362	14747411..	Unknown function	orf19.4019	3362	IPF3361	PROTEIN S	structural molecule activity
CA4787	0.9	0.9	0.8	IPF3364	complemer	Unknown function	orf19.4021	3364	IPF3362	UNCLASSI	molecular_function unknown
CA4788	1.0	1.0	1.1	SDH42	complemer	succinate dehydrogena	orf19.4022	3365	IPF3364	No significant S.c. match	
CA4789	1.0	1.0	1.0	IPF3366	complemer	Mitochondrial ribosoma	orf19.4023	3366	CaSDH42	C-compound and carbohydrate metabolism ENERGY SUBCELLULAR LOCALISATION	
CA4790	1.1	0.8	1.2	IPF3367	14750556..	Riboflavin synthase (by	orf19.4024	3367	IPF3366	PROTEIN S	structural molecule activity
CA4791	1.1	0.8	1.8	IPF3370	14751414..	similar to Saccharomy	orf19.4025	3370	IPF3367	Metabolism	transferase activity
CA4792	0.9	1.0	1.1	HIS1	complemer	ATP phosphoribosyltra	orf19.4026	3372	IPF3370	PROTEIN I	peptidase activity
CA4793	0.9	0.8	1.0	IPF3375	complemer	similar to Saccharomy	orf19.4028	3375	CaHIS1	Amino acid	transferase activity
CA4794	0.6	0.5	0.8	SQT1	14754614..	suppresses dominant-r	orf19.4029	3377	IPF3375	PROTEIN I	transferase activity
CA4795	1.1	1.0	1.0	IPF3378	14756058..	similar to Saccharomy	orf19.4030	3378	CaSQT1	PROTEIN I	molecular_function unknown
CA4796	0.9	0.9	1.0	IPF3380	complemer	unknown function	orf19.4031	3380	IPF3378	CELL CYC	nucleotidyltransferase activity
CA4797	1.0	0.9	1.0	PRP22	complemer	RNA-dependent ATPa	orf19.4033	8801	IPF3380	UNCLASSI	molecular_function unknown
CA4798	1.1	1.1	0.9	RPN5.3F	14763195..	subunit of the regulator	orf19.4032	8798	CaPRP22	TRANSCR	RNA binding,helicase activity
CA4799	1.0	1.0	1.0	RPN5.5F	14763967..	subunit of the regulator	orf19.4034	8797	CaRPN5.3I	PROTEIN I	peptidase activity
CA4800	1.6	2.2	1.4	IPF8796	complemer	putative GPI-anchhore	orf19.4035	8796	CaRPN5.5I	PROTEIN FATE [folding modification destination] ""SUBCELLULAR LOCALISATION	
CA4801	1.0	1.0	1.0	APM1	14766973..	AP-1 complex subunit,	orf19.4036	8795	IPF8796	SUBCELLU	molecular_function unknown
CA4802	0.9	1.0	0.9	ILV3	complemer	dihydroxyacid dehydra	orf19.4040	8790	CaAPM1	PROTEIN I	protein binding
CA4803	0.9	1.0	0.9	PEX4	14770965..	E2 ubiquitin-conjugatin	orf19.4041	7214	CaILV3	Amino acid	lyase activity
CA4804	0.9	0.9	1.1	ARO8	complemer	aromatic amino acid ar	orf19.2098	1147	CaPEX4	PROTEIN FATE [folding modification destination] ""SUBCELLULAR LOCALISATION	
CA4805	1.0	0.9	1.0	RAD5	14775461..	DNA helicase (by hom	orf19.2097	1153	CaARO8	Amino acid	transferase activity
CA4806	0.8	0.9	0.8	IPF1155	complemer	Putative dipeptidase (t	orf19.2095	1155	CaRAD5	CELL CYC	hydrolase activity
CA4807	1.0	1.1	0.9	PDR6	14781435..	Pleiotropic drug resista	orf19.2094	1158	IPF1155	UNCLASSI	molecular_function unknown
CA4808	1.6	1.0	1.4	RFA1	complemer	DNA replication factor	orf19.2093	1160	CAPDR6	PROTEIN I	protein binding
CA4809	1.0	1.0	1.0	IPF1162	complemer	Cystathionine beta-lya	orf19.2092	1162	CaRFA1	CELL CYC	DNA binding
CA4810	0.9	0.9	0.8	IPF1164	complemer	Subunit NUHM of NAD	orf19.2091	1164	IPF1162	Amino acid	lyase activity
CA4811	1.1	1.0	1.0	ECM16	complemer	RNA helicase (by hom	orf19.2090	1169	IPF1164	No significant S.c. match	

CA4812	1.0	1.0	1.0	IPF1171	14794532..	Putative synaptobrevin	orf19.2089	1171	CaECM16	CONTROL	RNA binding,helicase activity
CA4813	1.0	0.9	1.0	DPB4	complemer	DNA-directed DNA pol	orf19.2088	1174	IPF1171	PROTEIN I	transporter activity
CA4814	0.7	0.3	0.9	SAS2	14796407..	Zinc finger protein invo	orf19.2087	1176	CaDPB4	TRANSCR	nucleotidyltransferase activity
CA4815	1.0	0.9	0.9	CDH1	complemer	Substrate-specific acti	orf19.2084	20022	CaSAS2	CELL CYC	transferase activity
CA4816	1.0	0.9	1.0	IPF1183	14800278..	putative aspartyl prote	orf19.2082	1183	CaCDH1	CELL CYC	enzyme regulator activity
CA4817	1.0	1.0	1.1	POM152	14801845..	Nuclear pore membrar	orf19.2081	1191	IPF1183	PROTEIN FATE [folding modification destination]	
CA4818	1.0	1.2	1.0	IPF1193	complemer	unknown function	orf19.2079	1193	CaPOM152	CELLULAF	structural molecule activity
CA4819	0.9	0.9	1.0	IPF1194	14806771..	Similar to clathrin coat	orf19.2078	1194	IPF1193	Metabolism	molecular_function unknown
CA4820	1.0	1.0	1.0	IPF1196	14809301..	unknown function Hyp	orf19.2077	1196	IPF1194	CELLULAF	protein binding
CA4821	1.1	0.9	0.9	IPF1197	14812123..	unknown function	orf19.2076	1197	IPF1196	Amino acid metabolism Nitrogen and sulphur metabolism TRANSCRIPTION SUBCELLULAR LOCALISATION	
CA4822	1.0	1.0	1.1	DFG5	14814010..	Required for filamento	orf19.2075	1199	IPF1197	UNCLASSI	molecular_function unknown
CA4823	1.0	0.9	1.0	ERC3	14815987..	ethionine resistance pr	orf19.2073	1203	CaDFG5	CELL FATE	molecular_function unknown
CA4824	0.9	1.0	0.9	HNM2	14819106..	Choline permease (by	orf19.2072	1204	CaERC3	UNCLASSI	molecular_function unknown
CA4825	1.0	1.0	1.1	IPF1205	14821039..	unknown function	orf19.2071	1205	CaHNM2	CELLULAF	transporter activity
CA4826	0.8	0.9	0.9	IPF1206	14822042..	unknown function	orf19.2070	1206	IPF1205	No significant S.c. match	
CA4827	1.1	0.9	1.0	SMF2	complemer	Manganese transporte	orf19.2069	1207	IPF1206	UNCLASSI	molecular_function unknown
CA4828	1.1	0.9	0.9	IPF1209	14826300..	unknown function	orf19.2068	1209	CaSMF2	REGULATI	transporter activity
CA4829	1.1	1.1	1.0	IPF1210	complemer	similar to Saccharomy	orf19.2067	1210	IPF1209	UNCLASSIFIED PROTEINS	
CA4830	1.0	1.1	1.0	ATP8.EXO	complemer	F1F0-ATPase complex, Atp8 subu		1211	IPF1210	Nitrogen ar	molecular_function unknown
CA4831	0.8	0.9	0.9	IPF1212	complemer	unknown function	orf19.2066	1212	CaATP8.ex	ENERGY S	transporter activity
CA4832	0.9	0.8	0.9	DAL2	complemer	Allantoinase	orf19.2065	1213	IPF1212	UNCLASSI	transferase activity
CA4833	1.1	1.1	1.0	IPF1216	14832576..	unknown function	orf19.2064	1216	CaDAL2	Nitrogen ar	hydrolase activity
CA4834	1.1	1.0	1.0	IPF1217	14836185..	unknown function	orf19.2063	1217	IPF1216	CELL FATE UNCLASSIFIED PROTEINS	
CA4835	0.9	0.9	1.1	IPF1218	complemer	Similar to superoxide c	orf19.2062	1218	IPF1217	CELL FATE UNCLASSIFIED PROTEINS	
CA4836	0.1	0.0	0.3	IPF1222	complemer	Similar to superoxide c	orf19.2060	1222	IPF1218	CELL RESCUE DEFENSE AND VIRULENCE ""SUBCELLULAR LOCALISATION	
CA4837	0.9	1.0	1.0	IPF1228	14846438..	unknown function	orf19.2059	1228	IPF1222	CELL RESCUE DEFENSE AND VIRULENCE ""SUBCELLULAR LOCALISATION	
CA4838	1.0	1.1	1.0	YTA12	14848221..	Protease of the SEC18	orf19.2057	1231	IPF1228	No significant S.c. match	
CA4839	0.9	0.9	0.9	NPL6	14850998..	Nuclear protein localiz	orf19.2055	1234	CaYTA12	ENERGY "	peptidase activity
CA4840	0.9	1.0	1.0	IPF1235	complemer	unknown function	orf19.2054	1235	CaNPL6	PROTEIN I	molecular_function unknown
CA4841	1.0	1.2	1.0	IPF10355	complemer	unknown function	orf19.3809	10355	IPF1235	PROTEIN FATE [folding modification destination] ""SUBCELLULAR LOCALISATION	
CA4842	1.0	1.1	1.0	MTD1	complemer	methylenetetrahydrofo	orf19.3810	6938	IPF10355	Amino acid	transcription regulator activity
CA4843	1.1	1.0	1.0	GYP1	complemer	GTPase activating prot	orf19.3811	6940	CaMTD1	Nucleotide	oxidoreductase activity
CA4844	0.8	0.9	0.8	PDR13	complemer	Drug resistance	orf19.3812	6943	CaGYP1	CELLULAF	enzyme regulator activity
CA4845	0.8	0.8	0.9	IPF6945.5f	14865030..	unknown function, 5-pr	orf19.3813	6945	CaPDR13	CELL RES	chaperone activity
CA4846	1.0	1.0	0.9	IPF6945.3f	14865835..	unknown function, 3-pr	orf19.3814	6946	IPF6945.5f	No significant S.c. match	
CA4847	1.0	1.0	1.1	IPF6951	14867398..	similar to Saccharomy	orf19.3815	6951	IPF6945.3f	No significant S.c. match	
CA4848	0.9	0.8	0.9	RTS2	complemer	Unknown function	orf19.3817	6953	IPF6951	PROTEIN I	peptidase activity
CA4849	1.0	0.9	1.0	IPF6954	14871578..	unknown function	orf19.3818	6954	CaRTS2	CELL CYC	molecular_function unknown
CA4850	1.0	1.0	1.0	CIRT2	14873961..	Transposase	orf19.3820	3257	IPF6954	No significant S.c. match	
CA4851	1.0	1.0	0.9	IPF5818	14881168..	unknown function	orf19.3821	5818	CaCirt2	CLASSIFICATION NOT YET CLEAR-CUT	
CA4852	1.5	1.3	1.2	SCS7	complemer	Required for hydroxyla	orf19.3822	5819	IPF5818	No significant S.c. match	
CA4853	1.0	0.8	1.0	IPF5823	complemer	similar to Saccharomy	orf19.3823	5823	CaSCS7	Lipid fatty-a	oxidoreductase activity
CA4854	1.2	1.2	1.2	RCE1	complemer	CAAX PRENYL PROT	orf19.3825	5825	IPF5823	CELL CYC	protein binding
CA4855	1.0	0.9	1.0	IPF5830	14894227..	unknown function	orf19.3826	5830	CaRCE1	PROTEIN I	peptidase activity
CA4856	1.0	1.0	1.0	IPF15950	14896097..	unknown function	orf19.3827	15950	IPF5830	UNCLASSIFIED PROTEINS	
CA4857	1.4	1.1	1.1	PHR1	14899415..	GPI-anchored pH resp	orf19.3829	15932	IPF15950	UNCLASSI	molecular_function unknown
CA4858	0.9	1.1	1.0	IPF15927.3	complemer	similar to Saccharomy	orf19.3831	15929	CaPHR1	Lipid fatty-acid and isoprenoid metabolism ""CONTROL OF CELLULAR ORGANIZATION SUBCELLULAR LOCALISATION	
CA4859	1.0	1.1	1.1	IPF15927.5	complemer	similar to Saccharomy	orf19.3833	15927	IPF15927.3	UNCLASSI	RNA binding
CA4860	1.0	0.9	0.9	IPF19804	complemer	unknown Function	orf19.3835	19804	IPF15927.5	TRANSCR	transcription regulator activity
CA4861	1.0	0.9	1.0	IPF4094	14906755..	unknown function	orf19.3836	4094	IPF19804	TRANSCRIPTION SUBCELLULAR LOCALISATION	
CA4862	1.2	2.1	1.2	EFB1	14908222..	translation elongation f	orf19.3838	4091	IPF4094	UNCLASSI	molecular_function unknown
CA4863	1.0	1.0	1.0	IPF4089	complemer	secretory aspartyl prot	orf19.3839	4089	CaEFB1	PROTEIN S	translation regulator activity
CA4864	1.0	0.9	1.0	IPF4087	14912663..	similar to Saccharomy	orf19.3840	4087	IPF4089	PROTEIN FATE [folding modification destination] ""SUBCELLULAR LOCALISATION	
CA4865	1.0	0.9	1.0	IPF4085	14917806..	similar to Saccharomy	orf19.3841	4085	IPF4087	TRANSCR	protein kinase activity
CA4866	0.9	1.0	0.9	IPF4083	complemer	similar to Saccharomy	orf19.3843	4083	IPF4085	PROTEIN I	protein kinase activity
CA4867	1.1	1.0	1.1	IPF4080	complemer	similar to Saccharomy	orf19.3844	4080	IPF4083	PROTEIN I	transporter activity
CA4868	0.9	1.5	1.0	IPF4078	complemer	Unknown Function	orf19.3845	4078	IPF4080	PROTEIN S	structural molecule activity
CA4869	1.1	1.0	1.0	LYS4	14925418..	homoaconitate hydrata	orf19.3846	4077	IPF4078	No significant S.c. match	
CA4870	0.9	1.1	1.0	IPF19568	14935736..	unknown function	orf19.6556	19568	CaLYS4	Amino acid	lyase activity
CA4871	1.0	0.9	0.9	IPF1680	complemer	probable amidase	orf19.6557	1680	IPF19568	No significant S.c. match	
CA4872	1.0	1.0	1.0	SEC231	14941589..	Component of COPII c	orf19.6558	1677	IPF1680	Nitrogen ar	hydrolase activity
CA4873	1.1	1.0	1.0	IPF1674.3	complemer	putative transcription initiation fact		1675	CaSEC231	CELLULAR TRANSPORT AND TRANSPORT MECHANISMS SUBCELLULAR LOCALISATION	
CA4874	1.1	1.1	0.9	IPF1674	complemer	putative transcription i	orf19.6559	1674	IPF1674.3	No significant S.c. match	
CA4875	1.3	2.0	1.1	LAT1	complemer	Dihydrolipoamide S-ac	orf19.6561	1673	IPF1674	UNCLASSI	molecular_function unknown
CA4876	1.0	0.9	1.0	RNH35	complemer	RNase H (by homolog)	orf19.6562	1669	CaLAT1	C-compour	transferase activity
CA4877	1.0	1.0	1.1	IPF1667	14950419..	unknown function	orf19.6563	1667	CaRNH35	Nucleotide	RNA binding
CA4878	1.0	0.9	1.0	OXA1	complemer	Cytochrome oxidase b	orf19.6565	1663	IPF1667	UNCLASSI	molecular_function unknown
CA4879	1.0	0.9	1.0	IPF1660	14954636..	unknown function	orf19.6566	1660	CaOXA1	ENERGY "	transporter activity
CA4880	1.0	0.9	1.0	RHC18	complemer	Recombination repair p	orf19.6568	1656	IPF1660	UNCLASSIFIED PROTEINS	
CA4881	1.0	1.0	1.0	IPF1652	14960328..	putative purine nucleos	orf19.6569	1652	CaRHC18	CELL CYC	molecular_function unknown
CA4882	0.9	0.9	1.0	IPF1651	14962314..	purine nucleoside perm	orf19.6570	1651	IPF1652	No significant S.c. match	
CA4883	1.6	1.7	1.2	IPF1649	complemer	similar to Saccharomy	orf19.6573	1649	IPF1651	No significant S.c. match	

CA4884	1.0	0.9	1.0	ALK6	complemer	n-alkane inducible cytc	orf19.6574	1640	IPF1649	CELL CYC	signal transducer activity
CA4885	1.1	1.1	1.1	IPF1636	complemer	similar to Saccharomy	orf19.6577	1636	CaALK6	CELL RESCUE DEFENSE AND VIRULENCE ""CELL FATE CONTROL OF CELLULAR ORGANIZATION	
CA4886	1.0	1.0	0.9	IPF1634	complemer	unknown function	orf19.6578	1634	IPF1636	CELL RES	transporter activity
CA4887	1.0	1.0	1.1	IPF1632	complemer	unknown function	orf19.6579	1632	IPF1634	Phosphate metabolism CELLULAR TRANSPORT AND TRANSPORT MECHANISMS REGULATION OF/INTERACTION WITH CELLULAF	
CA4888	1.0	1.0	1.0	IPF1631	complemer	unknown function	orf19.6580	1631	IPF1632	No significant S.c. match	
CA4889	0.9	0.9	0.9	IPF1629	complemer	unknown function	orf19.6581	1629	IPF1631	No significant S.c. match	
CA4890	1.1	0.9	1.1	PRE10	14990263..	20S proteasome subui	orf19.6582	1628	IPF1629	UNCLASSI	molecular_function unknown
CA4891	0.9	0.9	1.0	IPF1627	14991363..	unknown function	orf19.6583	1627	CaPRE10	PROTEIN I	peptidase activity
CA4892	1.2	1.0	1.0	PRT1	14994841..	Translation initiation fa	orf19.6584	1623	IPF1627	CELLULAR TRANSPORT AND TRANSPORT MECHANISMS SUBCELLULAR LOCALISATION	
CA4893	1.0	0.9	0.9	IPF1621	complemer	unknown function	orf19.6585	1621	CaPRT1	CELL CYC	translation regulator activity
CA4894	0.9	0.9	0.8	IPF1617	15003438..	unknown function	orf19.6586	1617	IPF1621	UNCLASSI	molecular_function unknown
CA4895	1.0	0.9	1.0	IPF18161	complemer	unknown function	orf19.6587	18161	IPF1617	No significant S.c. match	
CA4896	0.9	0.9	1.0	IPF18160	15009072..	unknown function	orf19.6588	18160	IPF18161	UNCLASSI	molecular_function unknown
CA4897	1.3	1.5	2.0	TUB2.3	15014118..	Beta-tubulin, 3-prime e	orf19.6034	1463	IPF18160	PROTEIN I	molecular_function unknown
CA4898	0.7	0.8	0.6	IPF1461	complemer	putative NADH dehydr	orf19.6035	1461	CaTUB2.3	CELL CYC	structural molecule activity
CA4899	0.9	0.9	1.0	IPF1460	complemer	unknown function	orf19.6036	1460	IPF1461	No significant S.c. match	
CA4900	1.0	1.1	1.0	IFP2	complemer	unknown function	orf19.6037	1459	IPF1460	TRANSCR	enzyme regulator activity
CA4901	1.0	0.9	0.9	IPF1457	15023409..	putative transcription a	orf19.6038	1457	CaIFP2	No significant S.c. match	
CA4902	1.0	1.1	1.0	SED5	15025486..	Syntaxin (by homology	orf19.6039	1455	IPF1457	Nitrogen and sulphur metabolism TRANSCRIPTION SUBCELLULAR LOCALISATION	
CA4903	0.8	0.7	0.7	SNF7	15027036..	Class E Vps protein (b	orf19.6040	1454	CaSED5	CELLULAF	transporter activity
CA4904	1.1	1.0	1.0	RPO41	15028260..	Mitochondrial DNA-dire	orf19.6041	1453	CaSNF7	C-compour	molecular_function unknown
CA4905	1.0	0.8	0.9	DLD1	complemer	D-lactate ferricytochror	orf19.6043	1451	CaRPO41	CELL CYC	nucleotidyltransferase activity
CA4906	0.9	1.1	1.0	MOB2	complemer	Required for maintenai	orf19.6044	1449	CaDLD1	C-compound and carbohydrate metabolism ENERGY SUBCELLULAR LOCALISATION	
CA4907	0.9	0.9	1.0	PSD1	complemer	Phosphatidylserine dec	orf19.6045	1448	CaMOB2	No significa	structural molecule activity
CA4908	1.1	1.0	0.9	IPF1445	complemer	similar to Saccharomy	orf19.6046	1445	CaPSD1	Lipid fatty-a	lyase activity
CA4909	1.0	1.0	1.0	TUF1	complemer	Translation elongation	orf19.6047	1439	IPF1445	CELL CYC	protein binding
CA4910	0.9	1.0	1.0	IPF1437	complemer	unknown function	orf19.6048	1437	CaTUF1	PROTEIN S	translation regulator activity
CA4911	1.0	0.9	1.0	IPF1435	15045593..	unknown function	orf19.6049	1435	IPF1437	C-compound and carbohydrate metabolism ""PROTEIN FATE [folding modification destination] ""SUBCELLULAR LOCALISATION	
CA4912	0.9	1.0	0.9	CNS1	complemer	Cyclophilin Seven Sup	orf19.6052	1432	IPF1435	CELL CYC	enzyme regulator activity
CA4913	1.0	1.0	1.0	CIS2	complemer	Gamma-glutamyltransf	orf19.6053	1431	CaCNS1	UNCLASSI	chaperone activity
CA4914	1.1	1.0	0.9	IPF1428	15052039..	Similar to ubiquitinatior	orf19.6054	1428	CaCIS2	Amino acid	transferase activity
CA4915	0.9	1.0	1.0	IPF1427	15054729..	Similar to ubiquitinatior	orf19.6055	1427	IPF1428	PROTEIN FATE [folding modification destination]	
CA4916	1.0	0.9	0.9	IPF1425	complemer	Hypothetical phosphog	orf19.6056	1425	IPF1427	PROTEIN FATE [folding modification destination]	
CA4917	0.8	0.8	0.9	ECM31	complemer	Involved in cell wall bic	orf19.6057	1424	IPF1425	C-compour	molecular_function unknown
CA4918	1.0	1.0	0.8	GLO1	15059618..	Glyoxalase I (by homo	orf19.6058	1422	CaECM31	Metabolism	transferase activity
CA4919	1.4	1.2	1.5	TTR1	15060920..	Glutaredoxin (by homc	orf19.6059	1421	CaGLO1	Amino acid	lyase activity
CA4920	1.1	1.1	1.1	GCN20	15061459..	Positive effector of Gcı	orf19.6060	1420	CaTTR1	Nucleotide	transferase activity,oxidoreductase activity
CA4921	1.0	1.0	1.0	IPF1416	15063906..	unknown function	orf19.6061	1416	CaGCN20	PROTEIN S	molecular_function unknown
CA4922	1.1	1.0	0.9	IPF1415	complemer	unknown function	orf19.6062	1415	IPF1416	UNCLASSI	molecular_function unknown
CA4923	0.8	0.8	0.8	IPF1413.3	15066196..	unknown function, 3-prime end		1413	IPF1415	UNCLASSI	molecular_function unknown
CA4924	0.9	0.8	1.0	UBP6.3	complemer	Ubiquitin-specific prote	orf19.6063	1412	IPF1413.3	CLASSIFIC	molecular_function unknown
CA4925	0.9	1.0	1.0	IPF1408	complemer	unknown function	orf19.6064	1408	CaUBP6.3	PROTEIN I	peptidase activity
CA4926	1.0	1.0	1.1	IPF1404	complemer	unknown function	orf19.6065	1404	IPF1408	UNCLASSI	molecular_function unknown
CA4927	0.9	0.9	0.8	IPF1401	15072963..	similarity to aldehyde c	orf19.6066	1401	IPF1404	C-compound and carbohydrate metabolism ENERGY TRANSCRIPTION CELL FATE SUBCELLULAR LOCALISATION	
CA4928	1.0	0.9	1.1	IPF1399	complemer	unknown function	orf19.6068	1399	IPF1401	CLASSIFIC	molecular_function unknown
CA4929	0.4	0.4	0.3	ENA22	complemer	P-type ATPase involve	orf19.6070	1398	IPF1399	UNCLASSI	molecular_function unknown
CA4930	1.0	1.1	1.1	IPF8381	15082213..	similar to Saccharomy	orf19.6071	8381	CaENA22	REGULATI	transporter activity
CA4931	0.9	0.8	1.0	IPF8378	complemer	unknown function	orf19.6072	8378	IPF8381	CELL CYC	transferase activity
CA4932	2.1	2.1	1.9	IPF8374	15087606..	unknown function	orf19.6073	8374	IPF8378	No significant S.c. match	
CA4933	1.0	0.8	0.9	IPF8372	complemer	unknown function	orf19.6074	8372	IPF8374	Metabolism	oxidoreductase activity
CA4934	0.9	1.1	1.0	CDC36	15089444..	transcription factor (by	orf19.6075	8371	IPF8372	UNCLASSI	molecular_function unknown
CA4935	1.0	1.0	0.9	VPS29	complemer	vacuolar protein sorting	orf19.6076	8370	CaCDC36	CELL CYC	RNA binding
CA4936	1.3	1.4	1.4	IPF8369	complemer	unknown function	orf19.1349	8369	CaVPS29	PROTEIN I	molecular_function unknown
CA4937	1.2	1.1	1.1	TFP3	complemer	H+-ATPase by homolo	orf19.6538	5167	IPF8369	UNCLASSI	molecular_function unknown
CA4938	1.2	1.0	1.2	IPF5166	15094986..	unknown function	orf19.6537	5166	CaTFP3	PROTEIN I	transporter activity
CA4939	1.1	1.1	1.1	IQG1	15096398..	RAS GTPase-activatin	orf19.6536	5165	IPF5166	UNCLASSI	molecular_function unknown
CA4940	0.8	0.8	0.7	CRN1.3F	complemer	actin-binding protein, 3	orf19.6535	5163	CaIQG1	CELL CYC	protein binding
CA4941	0.9	0.9	1.0	CRN1.53F	complemer	actin-binding protein, 5-prime end		5160	CaCRN1.3	No significant S.c. match	
CA4943	0.9	0.9	0.8	IPF5158	complemer	unknown function	orf19.6534	5158	CaCRN1.5	SUBCELLU	protein binding
CA4944	0.9	1.0	1.0	MSK1	15104388..	lysyl-tRNA synthetase	orf19.6533	5156	IPF5158	No significant S.c. match	
CA4945	0.8	0.7	0.9	FLX1	complemer	MITOCHONDRIAL FA	orf19.6532	5154	CaMSK1	PROTEIN S	ligase activity
CA4946	0.9	1.1	1.1	TOM71	complemer	Translocase of the outer mitochon		5151	CaFLX1	Metabolism	transporter activity
CA4947	0.9	0.9	0.8	NUC2	15108686..	NADH-UBIQUINONE (	orf19.6531	5150	CaTOM71	PROTEIN I	transporter activity
CA4948	0.9	0.8	0.8	IPF5149	complemer	unknown function	orf19.6530	5149	CaNUC2	No significant S.c. match	
CA4949	1.0	1.3	0.9	CDC34	15113037..	Ubiquitin-conjugating e	orf19.6529	5145	IPF5149	PROTEIN FATE [folding modification destination] ""CELLULAR TRANSPORT AND TRANSPORT MECHANISMS SUBCELLULAR LOCAL	
CA4950	1.0	1.0	1.1	IPF5143	complemer	Unknown function	orf19.6528	5143	CaCDC34	Amino acid	ligase activity
CA4951	1.0	0.9	1.2	IPF13607	complemer	unknown function	orf19.6527	13607	IPF5143	No significant S.c. match	
CA4952	1.0	0.9	1.1	IPF13609	15120425..	unknown function	orf19.6526	13609	IPF13607	UNCLASSI	molecular_function unknown
CA4953	1.0	1.0	1.0	IPF4567.3	15121764..	unknown function, 3-pr	orf19.6525	4567	IPF13609	UNCLASSI	molecular_function unknown
CA4954	0.9	0.9	0.8	TOM40	complemer	mitochondrial import re	orf19.6524	4568	IPF4567.3	No significant S.c. match	
CA4955	0.8	0.9	0.7	IPF4580	complemer	putative allantoate peri	orf19.6522	4580	CaTOM40	PROTEIN I	transporter activity
CA4956	0.9	0.9	1.0	IPF4583	complemer	putative allantoate peri	orf19.6520	4583	IPF4580	TRANSPORT FACILITATION	

CA4957	1.0	1.0	0.9	IPF4588	complemer	putative aldehyde dehy	orf19.6518	4588	IPF4583	TRANSPO	transporter activity
CA4958	1.0	1.0	1.0	RAD14	15140020..	nucleotide excision rep	orf19.6517	4591	IPF4588	CLASSIFICATION NOT YET CLEAR-CUT	
CA4959	0.5	0.4	0.5	HSP90	15141513..	heat shock protein	orf19.6515	4596	CaRAD14	CELL CYC	DNA binding
CA4960	1.1	1.1	0.8	IPF3912	complemer	unknown function	orf19.6514	3912	CaHSP90	CELL RES	chaperone activity
CA4961	1.1	1.1	1.0	IPF3916	complemer	similar to Saccharomy	orf19.6512	3916	IPF3912	TRANSCR	transcription regulator activity
CA4962	1.1	1.0	1.0	TRL1	15160208..	tRNA ligase	orf19.6511	3918	IPF3916	CELLULAF	protein binding
CA4963	0.9	1.0	1.0	IPF3919	15162982..	unknown function	orf19.6510	3919	CaTRL1	TRANSCR	ligase activity
CA4964	1.2	1.0	1.1	IPF3920	15163666..	unknown function	orf19.6509	3920	IPF3919	CELL RESCUE DEFENSE AND VIRULENCE	
CA4965	1.0	1.0	0.9	IPF3921	complemer	unknown function	orf19.6508	3921	IPF3920	Nucleotide metabolism ""CELL RESCUE DEFENSE AND VIRULENCE ""SUBCELLULAR LOCALISATION	
CA4966	1.9	1.3	1.4	IPF3923	15165775..	unknown function	orf19.6507	3923	IPF3921	CELLULAF	transporter activity
CA4967	1.1	1.1	1.0	IPF3927	15167052..	unknown function	orf19.6506	3927	IPF3923	PROTEIN SYNTHESIS ""PROTEIN FATE [folding modification destination] ""SUBCELLULAR LOCALISATION UNCLASSIFIED PROTEINS	
CA4968	1.0	1.0	1.0	IPF3928	complemer	unknown function	orf19.6503	3928	IPF3927	UNCLASSI	molecular_function unknown
CA4969	1.0	0.9	1.0	IPF3930	15170109..	unknown function	orf19.6502	3930	IPF3928	No significant S.c. match	
CA4970	1.0	0.9	1.0	IPF4696	15175492..	unknown Function	orf19.5282	4696	IPF3930	CELL CYC	molecular_function unknown
CA4971	1.0	0.9	1.2	IPF4697	15177526..	similar to Saccharomy	orf19.5281	4697	IPF4696	UNCLASSIFIED PROTEINS	
CA4972	1.0	1.0	1.1	MUP1	15182420..	High affinity methionine	orf19.5280	4701	IPF4697	CELL CYC	RNA binding
CA4973	1.0	0.9	1.0	IPF4703	15184424..	unknown Function	orf19.5279	4703	CaMUP1	Amino acid	transporter activity
CA4974	1.0	0.9	1.0	IPF4704	15185345..	unknown Function	orf19.5278	4704	IPF4703	UNCLASSI	structural molecule activity
CA4975	1.0	1.0	1.1	IPF4706	complemer	unknown Function	orf19.5277	4706	IPF4704	UNCLASSI	molecular_function unknown
CA4976	0.9	1.0	1.0	IPF4708	15188026..	unknown Function	orf19.5276	4708	IPF4706	Nucleotide	molecular_function unknown
CA4977	1.0	0.9	1.1	IPF4710	15189601..	unknown Function	orf19.5275	4710	IPF4708	UNCLASSI	protein binding
CA4978	1.0	0.9	1.0	IPF2065	15193003..	unknown function	orf19.5274	2065	IPF4710	UNCLASSI	molecular_function unknown
CA4979	1.1	1.0	1.0	IPF2062	complemer	unknown function	orf19.5271	2062	IPF2065	UNCLASSI	molecular_function unknown
CA4980	1.1	1.1	1.0	IPF2059	15199221..	unknown function	orf19.5270	2059	IPF2062	Nucleotide metabolism CELL CYCLE AND DNA PROCESSING CELLULAR COMMUNICATION/SIGNAL TRANSDUCTION MECHANISM	
CA4981	0.8	0.5	1.0	IPF2057	complemer	unknown function	orf19.5269	2057	IPF2059	UNCLASSIFIED PROTEINS	
CA4982	1.0	1.0	1.0	NUT2	15203161..	Negative transcription	orf19.5268	2054	IPF2057	No significant S.c. match	
CA4983	0.9	0.8	1.0	IPF2053	15206221..	unknown function	orf19.5267	2053	CaNUT2	TRANSCR	transcription regulator activity
CA4984	0.9	1.0	1.0	IPF2052	complemer	unknown function	orf19.5266	2052	IPF2053	No significant S.c. match	
CA4985	1.0	1.1	1.0	IPF2050	complemer	similar to Saccharomy	orf19.5265	2050	IPF2052	No significant S.c. match	
CA4986	1.0	0.9	1.0	SER33	15215166..	Phosphoglycerate deh	orf19.5263	2046	IPF2050	CELL CYC	motor activity,structural molecule activity
CA4987	0.9	0.9	1.0	IPF2045	15216737..	unknown function	orf19.5262	2045	CaSER33	Amino acid	oxidoreductase activity
CA4988	1.1	1.0	1.1	RPN2	15217609..	Proteasome regulatory	orf19.5260	2043	IPF2045	No significant S.c. match	
CA4989	1.0	0.9	0.9	IPF2041	complemer	unknown function	orf19.5259	2041	CaRPN2	TRANSCR	peptidase activity,signal transducer activity
CA4990	1.0	0.9	1.0	IPF2039	complemer	unknown function	orf19.5258	2039	IPF2041	No significant S.c. match	
CA4991	1.0	1.0	1.0	LCB4	15224113..	Sphingolipid long chair	orf19.5257	2037	IPF2039	No significant S.c. match	
CA4992	1.0	0.9	0.9	IPF2036	complemer	unknown function	orf19.5256	2036	CaLCB4	Lipid fatty-acid and isoprenoid metabolism	
CA4993	1.0	0.9	0.9	PXA2	complemer	ABC transporter, perox	orf19.5255	2034	IPF2036	CELLULAR TRANSPORT AND TRANSPORT MECHANISMS SUBCELLULAR LOCALISATION	
CA4994	1.0	0.8	1.0	IPF2033	complemer	unknown function	orf19.5254	2033	CaPXA2	Lipid fatty-a	transporter activity,hydrolase activity
CA4995	1.0	1.0	0.9	YAK1	15230847..	Ser/thr protein kinase (	orf19.5253	2032	IPF2033	No significant S.c. match	
CA4996	0.9	1.0	1.0	IPF2029	complemer	unknown function	orf19.5251	2029	CaYAK1	CELL CYCLE AND DNA PROCESSING SUBCELLULAR LOCALISATION	
CA4997	1.0	1.0	0.9	IPF2027	15235858..	unknown function	orf19.5250	2027	IPF2029	TRANSCRIPTION ""CELL RESCUE DEFENSE AND VIRULENCE ""SUBCELLULAR LOCALISATION	
CA4998	1.1	0.8	1.0	IPF2026	complemer	unknown function	orf19.5249	2026	IPF2027	No significant S.c. match	
CA4999	1.0	1.0	1.1	IPF2024	15237770..	unknown function	orf19.5248	2024	IPF2026	No significant S.c. match	
CA5000	0.9	0.8	1.0	IPF2023	complemer	unknown function	orf19.5247	2023	IPF2024	CELLULAF	molecular_function unknown
CA5001	0.8	0.9	0.9	IPF2022	complemer	unknown function	orf19.5246	2022	IPF2023	UNCLASSI	molecular_function unknown
CA5002	1.0	1.0	1.0	IPF2021	15241490..	unknown function	orf19.5245	2021	IPF2022	No significant S.c. match	
CA5003	1.0	1.0	1.0	MCD4	15243972..	Sporulation protein (by	orf19.5244	2019	IPF2021	PROTEIN FATE [folding modification destination]	
CA5004	1.0	1.0	1.1	TRP3	15247113..	Anthranilate synthase /	orf19.5243	2018	CaMCD4	CELL FATI	molecular_function unknown
CA5005	1.2	1.2	1.1	CDC62	15249204..	Cell division control protein (by ho		18149	CaTRP3	Amino acid	lyase activity
CA5006	1.1	1.2	1.1	IPF12584	15251820..	unknown function	orf19.5241	12584	CaCDC62	No significant S.c. match	
CA5007	0.8	0.8	1.0	IPF12579	complemer	putative phospholipase	orf19.5239	12579	IPF12584	UNCLASSI	hydrolase activity
CA5008	1.0	1.0	1.0	IPF12577	complemer	unknown function	orf19.5238	12577	IPF12579	Lipid fatty-a	molecular_function unknown
CA5009	0.9	1.0	1.1	VPH2	15259729..	H+-ATPase assembly	orf19.5237	13946	IPF12577	No significant S.c. match	
CA5010	1.1	1.0	1.0	IPF13945	15260525..	Unknown function		13945	CaVPH2	PROTEIN I	molecular_function unknown
CA5011	1.0	1.0	1.0	IPF13944	15260959..	unknown function	orf19.5236	13944	IPF13945	PROTEIN FATE [folding modification destination] ""CONTROL OF CELLULAR ORGANIZATION	
CA5012	0.9	1.0	1.0	IPF13943	complemer	Unknown function	orf19.5235	13943	IPF13944	PROTEIN FATE [folding modification destination] ""CONTROL OF CELLULAR ORGANIZATION	
CA5013	1.1	1.2	1.0	IPF13941	complemer	Unknown function	orf19.5234	13941	IPF13943	No significant S.c. match	
CA5014	0.9	1.0	1.0	IPF11120	complemer	similar to Saccharomy	orf19.6966	11120	IPF13941	SUBCELLULAR LOCALISATION TRANSPORT FACILITATION	
CA5015	1.1	1.1	1.0	IPF11118	15275852..	unknown function	orf19.6967	11118	IPF11120	Lipid fatty-a	transferase activity
CA5016	1.0	0.9	1.2	IPF7561	15282767..	unknown function	orf19.6968	7561	IPF11118	CELLULAR TRANSPORT AND TRANSPORT MECHANISMS SUBCELLULAR LOCALISATION	
CA5017	1.0	1.1	1.0	IPF7559	complemer	unknown function	orf19.6970	7559	IPF7561	No significant S.c. match	
CA5018	1.0	0.9	0.9	IPF7558	15287768..	unknown function	orf19.6971	7558	IPF7559	UNCLASSIFIED PROTEINS	
CA5019	1.0	0.9	0.9	IPF7557	complemer	similar to Saccharomy	orf19.6972	7557	IPF7558	C-compour	transferase activity
CA5020	1.0	0.9	0.9	IPF7556	complemer	similar to Saccharomy	orf19.6973	7556	IPF7557	C-compound and carbohydrate metabolism SUBCELLULAR LOCALISATION	
CA5021	1.5	1.9	1.1	YST1.EXO	complemer	Ribosomal protein, exo	orf19.6975	7549	IPF7556	TRANSCRIPTION ""PROTEIN FATE [folding modification destination] ""SUBCELLULAR LOCALISATION	
CA5023	1.0	1.0	0.9	IPF7547	complemer	unknown function	orf19.6976	7547	CaYST1.ex	PROTEIN S	structural molecule activity
CA5024	0.8	0.8	1.0	IPF3009	complemer	similar to Saccharomy	orf19.6977	3009	IPF7547	UNCLASSI	molecular_function unknown
CA5025	1.2	1.0	1.1	AMI3	15306272..	protein required for no	orf19.6979	3003	IPF3009	Lipid fatty-a	molecular_function unknown
CA5026	1.0	0.9	1.0	IPF3001	15310230..	serine/threonine protei	orf19.6980	3001	CaAMI3	UNCLASSI	molecular_function unknown
CA5027	1.0	0.9	1.0	IPF2999	15312312..	unknown function	orf19.6981	2999	IPF3001	CELL RESCUE DEFENSE AND VIRULENCE	
CA5028	1.0	1.0	1.1	IPF2998	complemer	unknown function	orf19.6982	2998	IPF2999	UNCLASSI	molecular_function unknown
CA5029	1.1	1.1	0.9	IPF2997	complemer	unknown function	orf19.6983	2997	IPF2998	UNCLASSI	molecular_function unknown

CA5030	1.0	1.0	1.0	IPF2988	15331189..	unknown function	orf19.6984	2988	IPF2997	C-compound and carbohydrate metabolism TRANSCRIPTION SUBCELLULAR LOCALISATION	
CA5031	1.0	1.0	1.0	IPF20023	15333873..	similar to Saccharomy	orf19.6985	20023	IPF2988	UNCLASSIFIED PROTEINS	
CA5032	1.0	1.0	1.0	IPF2982	15338446..	unknown function	orf19.6986	2982	IPF20023	TRANSCR	DNA binding
CA5033	1.0	1.2	1.1	DNM1	complemer	Dynamin-related protei	orf19.6987	2980	IPF2982	UNCLASSIFIED PROTEINS	
CA5034	1.2	1.0	1.1	OST1	15344570..	oligosaccharyltransfera	orf19.6988	4532	CaDNM1	SUBCELLU	hydrolase activity
CA5035	1.0	1.0	1.0	IPF4531	complemer	unknown function	orf19.6989	4531	CaOST1	C-compour	transferase activity
CA5036	1.0	1.0	1.0	CYP52	complemer	Peptidyl-prolyl cis-trans	orf19.6990	4530	IPF4531	CLASSIFICATION NOT YET CLEAR-CUT	
CA5037	1.4	1.3	1.2	PRE3	15347635..	20S proteasome subur	orf19.6991	4529	CaCYP52	PROTEIN FATE [folding modification destination] ""SUBCELLULAR LOCALISATION	
CA5038	1.0	1.0	1.0	QDR2	15349554..	putative antibiotic resis	orf19.6992	4526	CaPRE3	PROTEIN I	peptidase activity
CA5039	0.6	0.6	0.4	GAP2	complemer	general amino acid per	orf19.6993	4523	CaQDR2	CELL RESCUE DEFENSE AND VIRULENCE ""TRANSPORT FACILITATION	
CA5040	1.1	1.1	1.1	BAT22	complemer	branched chain amino	orf19.6994	4517	CaGAP2	Amino acid	transporter activity
CA5041	1.0	1.0	1.0	FRP7	15357526..	member of the FRP fa	orf19.6995	4516	CaBAT22	Amino acid metabolism SUBCELLULAR LOCALISATION	
CA5042	1.1	1.0	0.9	IPF4514	15359151..	putative alpha-1,3-mar	orf19.6996	4514	CaFRP7	C-compound and carbohydrate metabolism	
CA5043	1.0	1.1	1.1	FRP4	15361837..	member of the FRP fa	orf19.6997	4513	IPF4514	C-compound and carbohydrate metabolism ""PROTEIN FATE [folding modification destination] ""	
CA5044				GTT2	15363476..15364279				CaFRP4	C-compound and carbohydrate metabolism	
CA5045	1.1	0.9	0.9	IPF4510	15364750..	unknown function	orf19.6999	4510			
CA5046	1.1	1.3	1.0	YCK2	15368249..	casein kinase I (by hor	orf19.7001	4506	IPF4510	CELL RESCUE DEFENSE AND VIRULENCE	
CA5047	0.9	1.0	1.0	IPF4504	complemer	unknown function	orf19.7002	4504	CaYCK2	CELL CYC	protein kinase activity
CA5048	1.0	1.0	1.0	IPF20024	complemer	unknown function	orf19.1053	20024	IPF4504	No significant S.c. match	
CA5049	1.2	1.2	1.0	IPF3714	complemer	similar to Saccharomy	orf19.3013	3714	IPF20024	TRANSCR	transcription regulator activity
CA5050	1.7	3.0	1.5	BMH2	complemer	similar to Saccharomy	orf19.3014	3712	IPF3714	C-compour	structural molecule activity
CA5051	0.9	0.9	0.7	IPF3709	complemer	unknown function	orf19.3015	3709	CaBMH2	CELL CYC	protein binding,DNA binding
CA5052	1.0	1.0	1.1	IPF3708	15385817..	unknown function	orf19.3016	3708	IPF3709	UNCLASSI	molecular_function unknown
CA5053	0.9	1.0	1.0	IPF3707	complemer	unknown function	orf19.3018	3707	IPF3708	No significant S.c. match	
CA5054	0.9	0.9	1.0	IPF3704	15387676..	unknown function	orf19.3019	3704	IPF3707	UNCLASSI	DNA binding,transcription regulator activity
CA5055	0.8	0.9	0.8	IPF3701	15390160..	unknown function	orf19.3021	3701	IPF3704	CELL CYC	helicase activity
CA5056	0.9	1.0	1.0	IPF3698	15391444..	similar to Saccharomy	orf19.3022	3698	IPF3701	UNCLASSI	molecular_function unknown
CA5057	0.9	0.9	1.0	IPF3695	15392660..	similar to Saccharomy	orf19.3023	3695	IPF3698	PROTEIN S	structural molecule activity
CA5058				MAS1	complement(15394649..15396052)				IPF3695	C-compour	transcription regulator activity
CA5059	1.0	1.0	1.0	IPF3691	15396512..	unknown function	orf19.3027	3691			
CA5060	0.9	0.9	0.9	IPF3690	complemer	unknown function	orf19.3029	3690	IPF3691	SUBCELLULAR LOCALISATION	
CA5061	1.0	0.8	0.9	IPF8495	complemer	unknown function	orf19.3030	8495	IPF3690	CLASSIFIC	hydrolase activity
CA5062	1.1	1.0	1.1	SEC62	complemer	subunit of ER protein-t	orf19.3031	8494	IPF8495	UNCLASSIFIED PROTEINS	
CA5063	0.9	0.9	0.9	IPF8493	complemer	putative member of no	orf19.3034	8493	CaSEC62	PROTEIN I	protein binding
CA5064	0.7	0.7	0.7	CHD1	15407747..	transcriptional regulato	orf19.3035	8489	IPF8493	TRANSPO	transporter activity
CA5065	0.6	0.9	0.6	IPF3584	15412520..	similar to Saccharomy	orf19.3037	3584	CaCHD1	TRANSCR	transcription regulator activity
CA5066	1.0	1.4	0.7	TPS2	15415493..	Threalose-6-phosphate	orf19.3038	3588	IPF3584	TRANSCR	RNA binding
CA5067	1.0	1.0	1.0	IPF3589	15418999..	putative alcohol acyl tr	orf19.3040	3589	CaTPS2	C-compour	hydrolase activity
CA5068	1.0	1.0	1.1	IPF3592	15421573..	unknown function	orf19.3041	3592	IPF3589	CLASSIFIC	molecular_function unknown
CA5069	1.0	0.9	0.9	IPF3593	15423778..	unknown function	orf19.3042	3593	IPF3592	UNCLASSI	molecular_function unknown
CA5070	0.9	0.9	1.0	IPF3594	15425647..	triglyceride lipase (by h	orf19.3043	3594	IPF3593	No significant S.c. match	
CA5071	0.9	0.9	1.0	IPF3597	complemer	similar to Saccharomy	orf19.3045	3597	IPF3594	Lipid fatty-a	hydrolase activity
CA5072	1.0	1.1	1.1	IPF3598	complemer	similar to Saccharomy	orf19.3047	3598	IPF3597	CLASSIFICATION NOT YET CLEAR-CUT	
CA5073	0.8	0.7	0.9	IPF3603	complemer	unknown function	orf19.3048	3603	IPF3598	C-compour	transcription regulator activity
CA5074	1.1	1.0	1.1	IPF3607	complemer	putative serine/threoni	orf19.3049	3607	IPF3603	No significant S.c. match	
CA5075	1.1	1.1	1.2	IPF3610	complemer	unknown function	orf19.3050	3610	IPF3607	CELL CYC	protein kinase activity
CA5076	1.1	0.9	1.0	IPF15301	15442825..	unknown function	orf19.3051	15301	IPF3610	UNCLASSI	enzyme regulator activity
CA5077	1.0	1.2	1.2	YPT1	15444263..	GTP-binding protein of	orf19.3052	15299	IPF15301	UNCLASSI	molecular_function unknown
CA5078	1.0	1.1	1.1	IPF15297	complemer	unknown function	orf19.3053	15297	CaYPT1	CELLULAF	hydrolase activity
CA5079	1.1	1.1	1.1	RPN3	15446623..	26S proteasome regul	orf19.3054	9265	IPF15297	No significant S.c. match	
CA5080	0.9	0.9	0.9	IPF9268.3	15448391..	similar to Saccharomy	orf19.3055	9268	CaRPN3	PROTEIN I	peptidase activity
CA5081	1.2	1.4	1.4	COQ6	complemer	monooxygenase (by h	orf19.3058	9273	IPF9268.3	TRANSCR	transcription regulator activity
CA5082	1.1	1.1	1.1	SUA70	complemer	TFIIB subunit (transcri	orf19.3059	9274	CaCOQ6	Metabolism	oxidoreductase activity
CA5083	1.0	1.1	1.1	IPF9278	15455618..	similar to Saccharomy	orf19.3060	9278	CaSUA70	TRANSCR	transcription regulator activity
CA5084	1.0	0.9	1.0	IMP1	complemer	protease, mitochondria	orf19.3061	9279	IPF9278	C-compour	transferase activity
CA5085	1.2	1.0	1.1	RPS22.EX	complemer	ribosomal protein S15a, exon 2 (by		9280	CaIMP1	PROTEIN I	peptidase activity
CA5087	0.5	0.3	0.8	IPF20025	complemer	unknown function	orf19.3062	20025	CaRPS22.(	PROTEIN SYNTHESIS SUBCELLULAR LOCALISATION	
CA5088	1.0	1.2	1.0	IPF9758	complemer	similar to Saccharomy	orf19.3063	9758	IPF20025	CELL RES	molecular_function unknown
CA5089	1.0	1.1	1.0	MRPL27	complemer	ribosomal protein (by h	orf19.3064	9757	IPF9758	CELL CYC	nucleotidyltransferase activity
CA5090	0.7	0.6	0.9	IFG4	complemer	probable d-amino acid	orf19.3065	9755	CaMRPL27	PROTEIN S	structural molecule activity
CA5091	0.9	0.7	1.0	ACF3	15467452..	endo-1,3-beta-glucana	orf19.1058	9751	CaIFG4	No significant S.c. match	
CA5092	1.0	1.1	1.0	IPF1394	complemer	unknown function	orf19.6450	1394	CaACF3	CLASSIFIC	hydrolase activity
CA5093	1.0	0.9	1.0	IPF1390	complemer	unknown function	orf19.6449	1390	IPF1394	No significant S.c. match	
CA5094	1.1	1.0	1.0	IPF1387	complemer	unknown function	orf19.6448	1387	IPF1390	No significant S.c. match	
CA5095	1.1	1.1	1.1	ARF21	15479458..	GTP-binding protein of	orf19.6447	1386	IPF1387	No significant S.c. match	
CA5096	1.0	0.9	0.9	IPF1384	15480233..	similar to Saccharomy	orf19.6445	1384	CaARF21	PROTEIN FATE [folding modification destination] ""CELLULAR TRANSPORT AND TRANSPORT MECHANISMS CONTROL OF CELLUL	
CA5097	0.8	0.9	0.8	IPF1382	15481253..	unknown function	orf19.6444	1382	IPF1384	Lipid fatty-a	isomerase activity
CA5098	1.0	1.1	1.0	IPF1380	15483540..	delta3-cis-delta2-trans	orf19.6443	1380	IPF1382	UNCLASSI	molecular_function unknown
CA5099	0.9	1.1	1.0	PRP8	complemer	U5 snRNP protein, pre	orf19.6442	1378	IPF1380	Lipid fatty-acid and isoprenoid metabolism ""ENERGY SUBCELLULAR LOCALISATION	
CA5100	1.0	1.1	1.0	IPF1372	15492089..	unknown function	orf19.6440	1372	CaPRP8	CELL CYC	RNA binding
CA5101	1.1	1.1	1.0	LCB1	15494654..	Serine C-palmitoyltrans	orf19.6438	1370	IPF1372	UNCLASSI	ligase activity
CA5102	0.9	0.9	1.0	CDC23	complemer	Subunit of anaphase-p	orf19.6437	1368	CaLCB1	Lipid fatty-a	transferase activity

CA5103	1.0	0.9	1.0	IPF1367	15498434..	unknown function	orf19.6436	1367	CaCDC23	CELL CYC	protein binding
CA5104	1.0	1.1	1.1	IPF1364	complemer	unknown function	orf19.6435	1364	IPF1367	No significant S.c. match	
CA5105	0.9	1.1	0.9	PEX19	15500780..	Required for biogenesi	orf19.6434	1363	IPF1364	UNCLASSI	molecular_function unknown
CA5106	0.9	0.7	1.0	AFG2	complemer	Member of the Sec18p	orf19.6432	1361	CaPEX19	SUBCELLU	molecular_function unknown
CA5107	1.1	1.0	1.1	IPF1358	15504327..	unknown function	orf19.6431	1358	CaAFG2	UNCLASSI	hydrolase activity
CA5108	1.1	1.2	1.1	SKI2	complemer	Antiviral protein and pu	orf19.6425	1354	IPF1358	No significant S.c. match	
CA5109	0.9	0.9	1.1	UBC9	15508929..	E2 ubiquitin-conjugatin	orf19.6424	1348	CaSKI2	CELL RES	RNA binding,helicase activity,translation regulator activity
CA5110	0.9	1.0	1.1	FBP26	complemer	Fructose-2,6-bisphosp	orf19.6423	1347	CaUBC9	CELL CYCLE AND DNA PROCESSING ""PROTEIN FATE [folding modification destination] ""SUBCELLULAR LOCALISATION	
CA5111	1.0	1.0	1.1	SSY5	15511240..	Involved in sulfonylurea	orf19.6422	1345	CaFBP26	C-compour	hydrolase activity
CA5112	1.3	2.0	1.3	IPF1341	complemer	Similarity to mucin prot	orf19.6420	1341	CaSSY5	REGULATION OF/INTERACTION WITH CELLULAR ENVIRONMENT	
CA5113	1.1	1.1	1.0	IPF20026	complemer	unknown function	orf19.6418	20026	IPF1341	No significant S.c. match	
CA5114	1.0	1.0	1.0	IPF1334	complemer	Conserved hypothetica	orf19.6417	1334	IPF20026	UNCLASSI	molecular_function unknown
CA5115	1.1	1.1	1.1	IPF1331	complemer	unknown function	orf19.6416	1331	IPF1334	UNCLASSI	molecular_function unknown
CA5116	0.7	0.4	1.1	TPM2.3	complemer	Tropomyosin, 3-prime end		1325	IPF1331	UNCLASSI	molecular_function unknown
CA5117	1.1	1.1	1.0	IPF1323	15530494..	unknown function	orf19.6414	1323	CaTPM2.3	CELL FATI	protein binding
CA5118	1.0	1.0	1.0	IPF1321	complemer	unknown function	orf19.6413	1321	IPF1323	No significant S.c. match	
CA5119	0.9	1.0	1.0	IPF1320	complemer	unknown function	orf19.6411	1320	IPF1321	No significant S.c. match	
CA5120	1.1	1.2	1.0	YDJ1	complemer	Mitochondrial and ER i	orf19.6408	1317	IPF1320	UNCLASSI	enzyme regulator activity
CA5121	1.0	1.0	0.9	IPF1310	complemer	unknown function	orf19.6407	1310	CaYDJ1	CELL CYCLE AND DNA PROCESSING ""PROTEIN FATE [folding modification destination] ""CELLULAR TRANSPORT AND TRANSPOR	
CA5122	1.2	1.0	1.4	IPF1308	15541942..	Similarity to ribosomal	orf19.6406	1308	IPF1310	PROTEIN I	molecular_function unknown
CA5123	1.0	1.0	1.0	IPF1306	15543459..	unknown function	orf19.6405	1306	IPF1308	PROTEIN S	molecular_function unknown
CA5124	0.9	1.1	1.0	GSH2	complemer	Glutathione synthetase	orf19.6404	1304	IPF1306	UNCLASSI	molecular_function unknown
CA5125	1.9	1.2	1.5	RPP2	15546934..	acidic ribosomal protein by homolo		5111	CaGSH2	Metabolism	ligase activity
CA5126	1.1	0.9	1.1	SLS1	complemer	Endoplasmic transloca	orf19.6403	5113	CaRPP2	PROTEIN S	structural molecule activity
CA5127	1.1	1.2	1.0	CYS3	complemer	cystathionine gamma-l	orf19.6402	5115	CaSLS1	PROTEIN I	molecular_function unknown
CA5128	0.9	0.9	1.0	IPF5118	complemer	unknown function	orf19.1375	5118	CaCYS3	Amino acid	lyase activity
CA5129	1.0	0.9	1.1	ATS1	complemer	similar to Saccharomy	orf19.1375	5119	IPF5118	UNCLASSI	molecular_function unknown
CA5130	0.9	0.7	1.0	IFH2	15553854..	Dioxygenase (by homo	orf19.1375	5121	CaATS1	SUBCELLU	molecular_function unknown
CA5131				IPF5124	15555334..15559494				CaIFH2	CELL RESCUE DEFENSE AND VIRULENCE	
CA5132	1.0	1.1	1.0	GTS1	complemer	Transcription factor by	orf19.6393	5128			
CA5133	0.9	0.9	1.0	IPF5129	complemer	unknown function	orf19.6392	5129	CaGTS1	CELL CYC	molecular_function unknown
CA5134	0.9	0.9	1.0	IPF5131	complemer	unknown function	orf19.6391	5131	IPF5129	No significant S.c. match	
CA5135	0.9	1.6	0.7	HSP104	15564334..	Heat shock protein (by	orf19.1374	5137	IPF5131	No significant S.c. match	
CA5136	0.9	0.8	1.0	IPF5139	15568890..	unknown function	orf19.1374	5139	CaHSP104	PROTEIN I	chaperone activity
CA5137	1.0	0.9	0.9	IPF1032	15572535..	similar to probable mei	orf19.4574	1032	IPF5139	No significant S.c. match	
CA5138	1.1	1.0	0.9	IPF1031	15574679..	Similar to aminoglycos	orf19.4575	1031	IPF1032	UNCLASSI	molecular_function unknown
CA5139	1.0	1.0	0.9	IPF1027	15577328..	unknown function	orf19.4577	1027	IPF1031	CLASSIFIC	molecular_function unknown
CA5140	1.0	1.0	1.1	TIM10	15580869..	Subunit of the Tim22-complex (by		1024	IPF1027	UNCLASSI	signal transducer activity
CA5141	0.9	1.0	0.9	CYT2	complemer	holocytochrome-c1 syr	orf19.4578	1023	CaTIM10	No significant S.c. match	
CA5142	0.9	1.0	1.0	IPF1022	complemer	similar to Saccharomy	orf19.4579	1022	CaCYT2	Metabolism	lyase activity
CA5143	0.9	1.0	1.0	IPF1020	complemer	Weak similarity to N. c	orf19.4580	1020	IPF1022	UNCLASSI	molecular_function unknown
CA5144	1.1	0.9	0.9	IPF1019	15585165..	unknown function	orf19.4581	1019	IPF1020	UNCLASSIFIED PROTEINS	
CA5145	0.9	0.9	1.0	SKI6	15586468..	3 ->5 exoribonuclease	orf19.4582	1018	IPF1019	UNCLASSI	transferase activity
CA5146	1.0	1.0	0.9	YMC2	complemer	Carnitine/acylcarnitine	orf19.4583	1017	CaSKI6	No significant S.c. match	
CA5147	1.1	1.0	1.0	PHO12	complemer	Acid phosphatase, sec	orf19.4584	1016	CaYMC2	CELLULAR TRANSPORT AND TRANSPORT MECHANISMS SUBCELLULAR LOCALISATION	
CA5148	0.9	0.9	1.0	TFG1	15590768..	RNA pol.II transcriptior	orf19.4585	1015	CaPHO12	Phosphate metabolism SUBCELLULAR LOCALISATION	
CA5149	1.0	1.0	1.0	HGH1	complemer	Similar to human HMG	orf19.4587	1013	CaTFG1	TRANSCR	transcription regulator activity
CA5150	1.0	1.1	1.0	FMS1	complemer	Similar to corticosteroi	orf19.4589	1011	CaHGH1	UNCLASSI	molecular_function unknown
CA5151	1.0	1.0	1.1	IPF1009	complemer	Weak similarity to S. c	orf19.4590	1009	CaFMS1	Metabolism	oxidoreductase activity
CA5152	1.0	1.0	1.0	CAT2	complemer	carnitine O-acetyltrans	orf19.4591	1006	IPF1009	TRANSCRIPTION SUBCELLULAR LOCALISATION	
CA5153	0.9	0.9	1.0	IPF1003	15607847..	unknown function	orf19.4592	1003	CaCAT2	Lipid fatty-a	transferase activity
CA5154	1.1	1.1	1.0	RGA2	15612572..	rho-GTPase activating	orf19.4593	1000	IPF1003	No significant S.c. match	
CA5155	1.0	1.0	1.0	IPF995	complemer	unknown function		995	CaRGA2	CELL FATI	signal transducer activity
CA5156	1.1	1.0	1.0	CLC1	15616949..	clathrin light chain (by	orf19.4594	994	IPF995	UNCLASSI	molecular_function unknown
CA5157	0.9	1.0	0.9	IPF993	complemer	unknown function	orf19.4595	993	CaCLC1	CELLULAF	structural molecule activity
CA5158	1.0	1.0	1.0	IPF992	15619879..	unknown function	orf19.4596	992	IPF993	No significant S.c. match	
CA5159	1.0	1.1	1.0	CAP2	complemer	F-actin capping proteir	orf19.4597	990	IPF992	No significant S.c. match	
CA5160	0.8	0.8	0.7	PHO89	complemer	Na+-coupled phosphat	orf19.4599	988	CaCAP2	CELL FATI	protein binding
CA5161	1.0	0.9	1.0	IPF983	15628986..	unknown function	orf19.4600	983	CaPHO89	Phosphate	transporter activity
CA5162	0.9	1.1	1.0	IPF982	complemer	Weakly similar to human dolichol-p		982	IPF983	UNCLASSI	molecular_function unknown
CA5163	1.0	1.0	0.9	TFC1	complemer	Transcription initiation	orf19.4601	6022	IPF982	No significant S.c. match	
CA5164	1.1	1.2	0.9	MDH1	15632903..	Mitochondrial malate d	orf19.4602	6025	CaTFC1	TRANSCR	transcription regulator activity
CA5165	0.9	1.1	1.0	ARL1	15634415..	GTP-binding protein of	orf19.4603	6027	CaMDH1	C-compour	oxidoreductase activity
CA5166	0.8	0.9	0.9	TYR1	complemer	Prephenate dehydroge	orf19.4605	6029	CaARL1	PROTEIN I	hydrolase activity
CA5167	1.0	1.0	1.1	ERG8	15636640..	Phosphomevalonate ki	orf19.4606	6030	CaTYR1	Amino acid	oxidoreductase activity
CA5168	0.9	1.1	1.0	IPF6032	15638410..	unknown function	orf19.4607	6032	CaERG8	Lipid fatty-a	transferase activity
CA5170	1.0	0.9	1.0	PDC12.EX	15640027..	Pyruvate decarboxylas	orf19.4608	6036	IPF6032	UNCLASSIFIED PROTEINS	
CA5171	0.9	0.8	1.0	IPF6037	15642439..	Similar to Legionella pr	orf19.4609	6037	CaPDC12.e	C-compound and carbohydrate metabolism ENERGY SUBCELLULAR LOCALISATION	
CA5172	1.0	1.1	1.0	CPS2.5F	15643464..	Carboxypeptidase YSC	orf19.4610	6038	IPF6037	UNCLASSI	molecular_function unknown
CA5173	1.0	1.1	1.1	CPS2.3F	15644533..	Carboxypeptidase YSCS precurso		18130	CaCPS2.5F	PROTEIN FATE [folding modification destination] ""SUBCELLULAR LOCALISATION	
CA5174	0.9	1.0	1.0	PRS4	15645480..	Ribose-phosphate pyro	orf19.4611	6040	CaCPS2.3F	PROTEIN FATE [folding modification destination] ""SUBCELLULAR LOCALISATION	
CA5175	0.9	1.1	0.9	IPF6041	complemer	Similar to Legionella pr	orf19.4612	6041	CaPRS4	Nucleotide	transferase activity

CA5176	1.0	1.0	1.0	IPF6045	complemer	unknown function	orf19.4614	6045	IPF6041	UNCLASSIFIED PROTEINS	
CA5177	0.8	0.9	0.8	IPF11101	15653368..	Weak similarity to C. c	orf19.4615	11101	IPF6045	UNCLASSI	molecular_function unknown
CA5178	1.2	1.3	1.4	POL30	complemer	Proliferating Cell Nucle	orf19.4616	20027	IPF11101	UNCLASSI	molecular_function unknown
CA5179	1.0	1.0	0.9	MAK3	complemer	N-acetyltransferase (by	orf19.4617	11098	CaPOL30	CELL CYC	nucleotidyltransferase activity
CA5180	1.3	3.5	1.1	FBA1	15657313..	fructose-bisphosphate	orf19.4618	11096	CaMAK3	PROTEIN I	transferase activity
CA5181	1.1	1.0	1.1	TIM12	complemer	subunit of the TIM22-c	orf19.4620	11094	CaFBA1	C-compour	lyase activity
CA5182	1.0	0.9	1.0	IPF11093	15659318..	weak similarity to pig t	orf19.4621	11093	CaTIM12	PROTEIN I	transporter activity
CA5183	0.9	1.0	1.0	IPF11090.E	15662288..	weak similarity to glute	orf19.4622	11090	IPF11093	UNCLASSI	molecular_function unknown
CA5184	0.9	1.2	1.0	IPF11090.E	15663575..	weak similarity to glutenin, exon 2		11086	IPF11090.e	UNCLASSI	molecular_function unknown
CA5185	2.9	1.0	3.4	NHP6A	15666811..	nonhistone chromosomal protein r		10163	IPF11090.e	No significant S.c. match	
CA5186	0.9	0.9	0.9	HRT2	15668014..	Similar to ScHRT2 (by	orf19.4624	10162	CaNHP6A	CELL FATE	DNA binding
CA5187	0.9	0.9	1.0	TOA2	complemer	TFIIA subunit 13.5 kD	orf19.4625	10160	CaHRT2	CLASSIFIC	molecular_function unknown
CA5188	0.9	1.0	0.9	TAP42	15670275..	Component of the Tor	orf19.4626	10159	CaTOA2	TRANSCR	transcription regulator activity
CA5189	0.8	0.9	0.9	IPF10158	complemer	Weak similarity to ScN	orf19.4627	10158	CaTAP42	CELL CYC	protein binding
CA5190	1.0	1.0	1.0	IPF10155	15675194..	unknown function	orf19.4628	10155	IPF10158	TRANSCR	structural molecule activity
CA5191	1.1	1.2	1.1	IPF19538	complemer	partially similar to Isocitrate dehyd		19538	IPF10155	UNCLASSI	molecular_function unknown
CA5192	0.9	0.9	0.9	HOK	15690495..	unknown function	orf19.7004	17447	IPF19538	C-compound and carbohydrate metabolism ENERGY TRANSCRIPTION SUBCELLULAR LOCALISATION	
CA5194	0.8	1.0	0.8	IPF11756	15695156..	unknown function	orf19.7006	11756	CaHOK	No significant S.c. match	
CA5195	1.0	0.9	1.0	GRP8	complemer	Similarity to dihydroflav	orf19.7009	2336	IPF11756	No significant S.c. match	
CA5196	1.1	1.0	1.0	IPF2338	15706958..	unknown function	orf19.7010	2338	CaGRP8	Metabolism of vitamins cofactors and prosthetic groups	
CA5197	1.0	1.0	1.0	IPF2342	15708991..	unknown function	orf19.7011	2342	IPF2338	UNCLASSI	molecular_function unknown
CA5198	1.0	0.8	1.0	LPA4	15712803..	Similar to ribosomal pr	orf19.7012	2343	IPF2342	UNCLASSI	molecular_function unknown
CA5199	1.0	1.0	1.0	IPF2334	complemer	unknown function	orf19.7013	2344	CaLPA4	PROTEIN S	structural molecule activity
CA5200	1.6	1.4	1.2	RPL10E	complemer	Ribosomal protein L10	orf19.7015	2347	IPF2334	CELL CYCLE AND DNA PROCESSING SUBCELLULAR LOCALISATION	
CA5201	0.5	0.7	0.4	IPF2349	complemer	similar to human sphin	orf19.7016	2349	CaRPL10E	PROTEIN S	structural molecule activity
CA5202	1.2	0.9	1.3	YOX1	15721529..	Similar to homoeodom	orf19.7017	2351	IPF2349	Lipid fatty-a	hydrolase activity
CA5203	1.4	2.1	1.2	RPS18	complemer	Ribosomal protein S18	orf19.7018	2353	CaYOX1	TRANSCR	DNA binding
CA5204	1.0	1.0	1.0	YML6	complemer	Ribosomal protein, mit	orf19.7019	2354	CaRPS18	PROTEIN S	structural molecule activity
CA5205	1.1	1.1	1.0	KEX1	complemer	Carboxypeptidase-alph	orf19.7020	2356	CaYML6	PROTEIN S	structural molecule activity
CA5206	3.7	2.8	2.8	GPH1	complemer	Glycogen phosphoryla	orf19.7021	2357	CaKEX1	PROTEIN I	peptidase activity
CA5207	1.0	0.9	1.0	IPF2359	15735088..	unknown function	orf19.7022	2359	CaGPH1	C-compour	transferase activity
CA5208	0.9	0.9	1.0	IPF2361	15736314..	unknown function	orf19.7023	2361	IPF2359	No significant S.c. match	
CA5209	1.1	0.9	0.9	MCM1	15738758..	Transcription factor of	orf19.7025	2363	IPF2361	UNCLASSI	molecular_function unknown
CA5210	0.9	1.0	1.0	IPF2373	15748251..	unknown function	orf19.7027	2373	CaMCM1	Amino acid	DNA binding
CA5211	0.7	0.6	0.7	IPF18125	15750117..	similar to glutenin and	orf19.7028	18125	IPF2373	No significant S.c. match	
CA5212	1.0	1.0	0.9	IPF3050	complemer	unknown function	orf19.7029	3050	IPF18125	No significant S.c. match	
CA5213	1.0	1.3	1.1	SSR1	complemer	Secretory Stress Resp	orf19.7030	3054	IPF3050	CLASSIFIC	hydrolase activity
CA5214	1.2	1.0	1.0	IPF19807	complemer	unknown function	orf19.7032	19807	CaSSR1	No significa	structural molecule activity
CA5215	1.1	0.9	1.0	PPS1	15764307..	protein tyrosine phospl	orf19.7033	3063	IPF19807	Amino acid metabolism SUBCELLULAR LOCALISATION	
CA5216	0.9	0.9	1.0	IPF3069	15767764..	unknown function	orf19.7034	3069	CaPPS1	CELL CYC	protein phosphatase activity
CA5217	1.0	0.9	0.9	RFC2	complemer	Replication factor (by h	orf19.7035	3070	IPF3069	No significant S.c. match	
CA5218	0.9	1.0	1.0	WHI2	complemer	Growth regulation facto	orf19.7036	3073	CaRFC2	CELL CYC	DNA binding
CA5219	0.9	0.9	0.9	YAE1	15773683..	Essential protein	orf19.7037	3074	CaWHI2	CELL FATE	enzyme regulator activity
CA5220	1.0	1.1	1.1	MVP1.EXO	complemer	Required for vacuolar p	orf19.7038	3075	CaYAE1	UNCLASSI	molecular_function unknown
CA5221	1.0	1.0	1.0	MVP1.EXO	complemer	Required for vacuolar p	orf19.7039	3076	CaMVP1.e	PROTEIN I	molecular_function unknown
CA5222	0.9	0.9	0.9	IPF3079	complemer	similar to Saccharomyc	orf19.7041	3079	CaMVP1.e	PROTEIN FATE [folding modification destination] ""CELLULAR TRANSPORT AND TRANSPORT MECHANISMS SUBCELLULAR LOCAL	
CA5223	0.8	0.9	0.9	IPF3080	complemer	unknown function (by h	orf19.7042	3080	IPF3079	TRANSCR	RNA binding
CA5224	1.0	1.0	1.0	IPF3081	15783003..	unknown function	orf19.7043	3081	IPF3080	No significant S.c. match	
CA5225	1.0	1.2	1.0	ACB1.EXO	complemer	acyl-coenzyme-A-binding protein,		3082	IPF3081	UNCLASSI	molecular_function unknown
CA5226	1.0	0.9	1.0	RIM15	complemer	Protein kinase involvec	orf19.7044	3086	CaACB1.e	Lipid fatty-a	transporter activity
CA5227	0.9	1.1	1.0	IPF3087	complemer	unknown function	orf19.7046	3087	CaRIM15	TRANSCR	protein kinase activity
CA5228	0.9	0.9	0.8	RTF1.3EO	complemer	Regulates DNA binding	orf19.7047	3088	IPF3087	Amino acid	DNA binding
CA5229	0.9	0.7	1.0	IPF10425	15795157..	unknown function	orf19.5035	10425	CaRTF1.3e	TRANSCR	transcription regulator activity
CA5230	1.0	1.0	0.9	IPF10424	complemer	unknown function	orf19.5034	10424	IPF10425	CELL CYC	transcription regulator activity
CA5231	0.9	0.7	0.9	IPF10422	15799197..	Similar to APG12, com	orf19.5033	10422	IPF10424	UNCLASSI	molecular_function unknown
CA5232	1.0	1.0	1.0	SUN42	complemer	Putative cell wall beta-	orf19.5032	10421	IPF10422	PROTEIN I	molecular_function unknown
CA5233	1.1	1.1	1.0	SSK1	complemer	Putative reponse regul	orf19.5031	3012	CaSUN42	CELL CYCLE AND DNA PROCESSING CELL FATE	
CA5234	1.0	0.9	0.9	IPF3014	complemer	weak similarity to S. ce	orf19.5030	3014	CaSSK1	C-compour	signal transducer activity,transcription regulator activity,enzyme regulator activity
CA5235	0.8	0.9	0.9	IPF3015	15809952..	Similar to E.coli modF	orf19.5029	3015	IPF3014	UNCLASSI	molecular_function unknown
CA5236	1.0	0.9	1.0	LCB2	15812272..	Palmitoyl transferase (	orf19.5027	3017	IPF3015	CELL CYC	transporter activity,hydrolase activity
CA5237	1.0	1.0	1.0	ZMS1	15818471..	Zinc Finger Protein C2	orf19.5026	3021	CaLCB2	Lipid fatty-a	transferase activity
CA5238	1.0	1.0	1.0	MET3	complemer	ATP sulfurylase	orf19.5025	3022	CaZMS1	TRANSCR	molecular_function unknown
CA5239	1.8	1.8	1.3	GND1	15827975..	6-phosphogluconate de	orf19.5024	3028	CaMET3	Amino acid	nucleotidyltransferase activity
CA5240	0.9	0.9	0.9	IPF3032	15830354..	allantoate permease (t	orf19.5023	3032	CaGND1	C-compour	oxidoreductase activity
CA5241	1.0	1.0	1.0	SMF3	complemer	Probable manganese t	orf19.5022	3034	IPF3032	TRANSPORT FACILITATION	
CA5242	0.9	1.1	1.0	PDX1	15835165..	Pyruvate dehydrogena	orf19.5021	3038	CaSMF3	PROTEIN FATE [folding modification destination] ""CELLULAR TRANSPORT AND TRANSPORT MECHANISMS REGULATION OF/INTE	
CA5243	1.0	0.9	1.0	IPF3040	15836861..	unknown function	orf19.5020	3040	CaPDX1	C-compour	protein binding
CA5244	1.0	1.0	1.2	IPF3043	complemer	unknown function	orf19.5019	3043	IPF3040	No significant S.c. match	
CA5245	0.9	0.9	0.9	DUR32	15841924..	Urea transport protein	orf19.5017	3048	IPF3043	No significant S.c. match	
CA5246	0.9	1.0	1.0	IPF19808	15844117..	unknown function	orf19.5016	19808	CaDUR32	REGULATION OF/INTERACTION WITH CELLULAR ENVIRONMENT SUBCELLULAR LOCALISATION TRANSPORT FACILITATION	
CA5247	1.1	1.0	1.1	MYO2	complemer	Myosin heavy chain (b	orf19.5015	13531	IPF19808	No significant S.c. match	
CA5248	1.0	1.1	1.0	IPF1828	15851987..	unknown function	orf19.5014	1828	CaMYO2	CELLULAF	motor activity

CA5249	1.0	1.0	1.0	PCM1	complemer	phosphoacetylglucosar	orf19.5013	1827	IPF1828	UNCLASSI	molecular_function unknown
CA5250	0.9	0.9	0.9	IPF1826	15854644..	unknown function	orf19.5012	1826	CaPCM1	C-compour	isomerase activity
CA5251	1.0	1.1	1.0	IPF1824	complemer	unknown function	orf19.5011	1824	IPF1826	No significant S.c. match	
CA5252	1.0	1.0	1.0	DIM1	15858440..	rRNA (adenine-N6,N6-	orf19.5010	1822	IPF1824	CELL CYC	molecular_function unknown
CA5253	1.0	1.1	1.1	KEL3	complemer	Kelch-repeat protein	orf19.5009	1821	CaDIM1	TRANSCR	transferase activity
CA5254	1.0	1.0	1.0	IPF1820	15861698..	unknown function		1820	CaKEL3	UNCLASSI	molecular_function unknown
CA5255	0.7	0.7	1.7	ACT1	complemer	actin (by homology)	orf19.5007	1819	IPF1820	UNCLASSI	structural molecule activity
CA5257	1.1	0.9	1.1	SNC2.EXC	15865513..	Strong similarity to synaptobrevin,		1811	CaACT1	CELL CYC	structural molecule activity
CA5258	1.2	1.1	1.1	GCV3	15866499..	Glycine decarboxylase	orf19.5006	1809	CaSNC2.e:	PROTEIN I	transporter activity
CA5259	1.0	1.2	1.1	OSM2	complemer	Osmotic growth protein	orf19.5005	1808	CaGCV3	Amino acid	oxidoreductase activity
CA5260	1.0	1.0	1.1	RAD54	complemer	DNA-dependent ATPa	orf19.5004	1807	CaOSM2	ENERGY ""CELL RESCUE DEFENSE AND VIRULENCE ""SUBCELLULAR LOCALISATION	
CA5261	1.0	1.0	1.1	IPF1805	15872431..	unknown function	orf19.5003	1805	CaRAD54	CELL CYC	DNA binding
CA5262	0.9	1.0	0.9	IPF1804	complemer	putative transcription fa	orf19.5001	1804	IPF1805	UNCLASSI	enzyme regulator activity,protein binding
CA5263	0.9	0.9	0.9	CYB3	complemer	Lactate dehydrogenase	orf19.5000	1801	IPF1804	TRANSCR	transcription regulator activity
CA5264	0.9	2.0	0.7	IPF1798	complemer	unknown function	orf19.4998	1798	CaCYB3	C-compour	oxidoreductase activity
CA5265	1.0	1.1	1.0	IPF1792	15885491..	unknown function	orf19.4997	1792	IPF1798	Amino acid metabolism TRANSCRIPTION SUBCELLULAR LOCALISATION	
CA5266	0.9	1.0	0.9	IPF1787.3f	complemer	unknown function, 3-pr	orf19.4996	1789	IPF1792	C-compound and carbohydrate metabolism TRANSCRIPTION	
CA5267	1.0	0.9	1.0	IPF1787.5f	complemer	unknown function, 5-pr	orf19.4995	1787	IPF1787.3f	UNCLASSI	molecular_function unknown
CA5268	0.9	1.0	1.1	SEC18.3F	complemer	vesicular fusion protein	orf19.4994	1786	IPF1787.5f	No significant S.c. match	
CA5270	1.0	0.9	1.0	SEC18.5F	complemer	vesicular fusion protein	orf19.4993	1784	CaSEC18.3	CELLULAF	hydrolase activity
CA5271	1.0	1.1	1.0	IPF1777	complemer	similar to Saccharomyc	orf19.4991	1777	CaSEC18.5	CELLULAR TRANSPORT AND TRANSPORT MECHANISMS SUBCELLULAR LOCALISATION	
CA5272	1.0	1.0	1.0	IPF1770	complemer	unknown function	orf19.4988	1770	IPF1777	CELL CYC	RNA binding
CA5273	0.9	1.1	1.0	NUP49	15905616..	nuclear pore protein (b	orf19.4987	1766	IPF1770	No significant S.c. match	
CA5274	0.9	0.9	1.0	IPF1764	complemer	similar to Saccharomyc	orf19.4985	1764	CaNUP49	TRANSCR	structural molecule activity
CA5275	1.0	1.1	1.0	IPF1760.3f	complemer	unknown function, 3-pr	orf19.1245	1760	IPF1764	TRANSPO	transporter activity
CA5276	1.1	1.0	1.1	IPF1759.53	complemer	unknown function, similar to endoc		1759	IPF1760.3f	C-compound and carbohydrate metabolism	
CA5277	1.1	1.1	1.0	CTA29.EXC	15918562..	Protein with putative tr	orf19.7127	1892	IPF1759.53	No significant S.c. match	
CA5278	0.5	0.8	0.3	CTA29.EXC	15919539..	Protein with putative transcription a		1893	CaCTA29.e	No significant S.c. match	
CA5279	1.0	0.8	1.1	SYS1	15920247..	Similar to ypt6 suppres	orf19.7128	1895	CaCTA29.e	No significant S.c. match	
CA5280	1.0	0.9	0.9	IPF1899	15921443..	unknown function	orf19.7131	1899	CaSYS1	CELLULAF	molecular_function unknown
CA5281	0.9	0.9	0.9	SPT6	complemer	Transcription elongatio	orf19.7136	1910	IPF1899	UNCLASSI	molecular_function unknown
CA5282	1.0	1.1	1.0	IPF1911	complemer	unknown function	orf19.7139	1911	CaSPT6	CELL CYC	transcription regulator activity
CA5283	1.0	0.9	1.4	IPF1912	15927356..	putative catechol o-me	orf19.7140	1912	IPF1911	No significant S.c. match	
CA5284	1.1	1.1	1.0	UFE1	15928155..	Endoplasmic reticulum	orf19.7141	1914	IPF1912	No significant S.c. match	
CA5285	1.0	1.2	0.9	HBS1.3F	complemer	Translation elongation	orf19.7144	1918	CaUFE1	CELLULAF	transporter activity
CA5287	0.9	1.0	1.0	IPF1922	complemer	similar to multidrug res	orf19.7148	1922	CaHBS1.3f	PROTEIN S	molecular_function unknown
CA5288	0.9	1.0	0.9	IPF1928	15935369..	unknown function	orf19.7149	1928	IPF1922	CELL RESCUE DEFENSE AND VIRULENCE ""TRANSPORT FACILITATION	
CA5289	0.9	1.3	1.0	NRG1	complemer	similar to transcriptiona	orf19.7150	1932	IPF1928	UNCLASSI	molecular_function unknown
CA5290	1.3	1.9	1.4	IPF1943	15953097..	similar to Aspergillus (E	orf19.7152	1943	CaNRG1	UNCLASSI	transcription regulator activity
CA5291	1.0	0.9	1.1	LOS1	15954470..	pre-tRNA splicing prote	orf19.7153	1945	IPF1943	Amino acid metabolism	
CA5292	1.0	1.1	0.9	IPF1948	15957726..	unknown function	orf19.7154	1948	CaLOS1	TRANSCR	RNA binding,protein binding
CA5293	1.0	1.0	1.0	FAA24	complemer	Long-chain-fatty-acid--	orf19.7156	1949	IPF1948	UNCLASSI	molecular_function unknown
CA5294	0.9	1.0	1.0	IPF1952	complemer	unknown function	orf19.7157	1952	CaFAA24	Lipid fatty-acid and isoprenoid metabolism ""CELLULAR TRANSPORT AND TRANSPORT MECHANISMS SUBCELLULAR LOCALISATIC	
CA5295	0.9	0.9	1.0	IPF1954	complemer	putative transporter (by	orf19.7158	1954	IPF1952	No significant S.c. match	
CA5296	1.0	0.9	0.9	IPF1956	complemer	unknown function	orf19.7159	1956	IPF1954	TRANSPORT FACILITATION	
CA5297	1.0	1.0	0.9	YAR1	15967930..	Ankyrin repeat-contain	orf19.7160	1957	IPF1956	UNCLASSI	molecular_function unknown
CA5298	0.8	0.9	0.9	SUI3	15968805..	Translation initiation fa	orf19.7161	19809	CaYAR1	CELL CYC	molecular_function unknown
CA5299	1.0	1.0	1.0	IPF2190	complemer	putative serine/threonin	orf19.7164	2190	CaSUI3	PROTEIN S	translation regulator activity
CA5300	1.0	1.0	1.0	IPF2189	15971542..	unknown function	orf19.7165	2189	IPF2190	CLASSIFIC	protein kinase activity
CA5301	1.1	1.0	1.0	IPF2186	15973161..	unknown function	orf19.7166	2186	IPF2189	No significant S.c. match	
CA5302	1.1	0.9	1.0	IPF20175	15975761..	unknown function	orf19.7167	20175	IPF2186	UNCLASSIFIED PROTEINS	
CA5303	1.0	0.9	1.0	IPF2180	15977893..	unknown function	orf19.7170	2180	IPF20175	No significant S.c. match	
CA5304	1.1	1.1	1.2	IPF2178	complemer	unknown function	orf19.7173	2178	IPF2180	No significant S.c. match	
CA5305	1.0	1.0	1.0	IPF2175	complemer	similar to Saccharomyc	orf19.7175	2175	IPF2178	No significant S.c. match	
CA5306	0.9	1.0	1.0	NPT1	15981895..	Nicotinate phosphoribo	orf19.7176	2173	IPF2175	PROTEIN I	molecular_function unknown
CA5307	0.9	1.0	0.9	IPF2172	15983406..	similar to Saccharomyc	orf19.7177	2172	CaNPT1	Metabolism	transferase activity
CA5308	0.9	1.0	1.1	PRE5	15986569..	20S proteasome subur	orf19.7178	2171	IPF2172	PROTEIN I	structural molecule activity
CA5309	0.9	0.9	0.9	IPF2170	complemer	similar to saccharomyc	orf19.7179	2170	CaPRE5	PROTEIN I	peptidase activity
CA5310	0.9	1.1	1.0	IPF2167	complemer	unknown function	orf19.7181	2167	IPF2170	PROTEIN FATE [folding modification destination] ""CELLULAR TRANSPORT AND TRANSPORT MECHANISMS ""CELL RESCUE DEFE	
CA5311	1.0	1.0	1.0	IPF2166	15991528..	unknown function	orf19.7182	2166	IPF2167	PROTEIN FATE [folding modification destination] ""SUBCELLULAR LOCALISATION	
CA5312	0.9	0.9	1.0	IPF2165	complemer	unknown function	orf19.7183	2165	IPF2166	UNCLASSI	molecular_function unknown
CA5313	0.9	1.0	1.0	IPF2163	15993216..	unknown function	orf19.7184	2163	IPF2165	UNCLASSI	molecular_function unknown
CA5314	1.0	0.9	1.0	HAT2	15993846..	Subunit of the major ye	orf19.7185	2161	IPF2163	UNCLASSIFIED PROTEINS	
CA5315	1.1	1.1	1.0	CYB2	15996981..	B-type cyclin	orf19.7186	2159	CaHAT2	TRANSCRIPTION ""PROTEIN FATE [folding modification destination] ""SUBCELLULAR LOCALISATION	
CA5316	1.0	1.1	1.0	MAM33	15998865..	Mitochondrial acidic ma	orf19.7187	2157	CaCYB2	CELL CYC	protein kinase activity,enzyme regulator activity
CA5317	1.8	2.9	1.6	RPP1B	16000013..	Acidic ribosomal protei	orf19.7188	2156	CaMAM33	SUBCELLU	molecular_function unknown
CA5318	1.0	1.0	1.1	OGG1	complemer	8-oxoguanine DNA gly	orf19.7190	2154	CaRPP1B	PROTEIN SYNTHESIS SUBCELLULAR LOCALISATION	
CA5319	1.0	1.1	1.0	IPF2150	16002602..	similar to protein involv	orf19.7193	2150	CaOGG1	CELL CYC	DNA binding
CA5320	1.0	0.9	1.0	IPF2147	16004783..	unknown function	orf19.7194	2147	IPF2150	Amino acid	molecular_function unknown
CA5321	1.1	0.9	1.1	RAD6.3	complemer	Ubiquitin protein ligase	orf19.7195	2145	IPF2147	No significant S.c. match	
CA5322	1.4	1.5	1.1	PRB1	complemer	Protease B, vacuolar (I	orf19.7196	2144	CaRAD6.3	CELL CYCLE AND DNA PROCESSING TRANSCRIPTION ""PROTEIN FATE [folding modification destination] ""CELL FATE SUBCELLUL	
CA5323	0.9	1.0	1.0	IPF2142	complemer	unknown function	orf19.7197	2142	CaPRB1	PROTEIN I	peptidase activity

CA5324	0.9	0.6	1.0	IPF2140	complemer	unknown function	orf19.7198	2140	IPF2142	PROTEIN I	protein binding
CA5325	1.0	1.0	0.9	IPF2138	16013645..	unknown function	orf19.7199	2138	IPF2140	UNCLASSI	molecular_function unknown
CA5326	1.4	1.1	1.3	IPF2137	16014422..	unknown function	orf19.7200	2137	IPF2138	UNCLASSI	molecular_function unknown
CA5327	1.1	1.0	1.0	SLA2	complemer	Cytoskeleton assembly	orf19.7201	2136	IPF2137	No significant S.c. match	
CA5328	1.0	1.1	1.0	RER1	complemer	Required for correct lo	orf19.7202	2132	CaSLA2	Nucleotide	structural molecule activity
CA5329	1.0	0.9	1.0	MRP7	complemer	Mitochondrial ribosoma	orf19.7203	2131	CaRER1	PROTEIN I	molecular_function unknown
CA5330	1.0	1.0	1.0	IPF2130	16022187..	similar to 2-nitropropar	orf19.7204	2130	CaMRP7	PROTEIN S	structural molecule activity
CA5331	1.0	1.0	1.0	DUR33	complemer	Urea transport protein	orf19.7205	901	IPF2130	Nitrogen ar	molecular_function unknown
CA5332	1.0	1.0	0.9	IPF900.3	16026752..	unknown function, , 3-p	orf19.7206	900	CaDUR33	REGULATION OF/INTERACTION WITH CELLULAR ENVIRONMENT SUBCELLULAR LOCALISATION TRANSPORT FACILITATION	
CA5333	1.0	1.1	1.0	DOA4	complemer	ubiquitin-specific isope	orf19.7207	899	IPF900.3	UNCLASSI	molecular_function unknown
CA5334	1.1	1.0	1.1	SMK1	16031171..	MAP kinase (by homol	orf19.7208	895	CaDOA4	PROTEIN I	peptidase activity
CA5335	1.0	1.2	1.1	IPF894	16032418..	unknown function	orf19.7209	894	CaSMK1	CELLULAF	protein kinase activity,signal transducer activity
CA5336	1.1	0.8	1.1	IPF893	16033135..	unknown function	orf19.7210	893	IPF894	CELL FATE SUBCELLULAR LOCALISATION PROTEIN ACTIVITY REGULATION	
CA5337	1.0	0.9	1.0	APL1	16034302..	AP-2 complex subunit,	orf19.7212	891	IPF893	UNCLASSI	molecular_function unknown
CA5338	1.0	1.0	1.0	IPF889	complemer	ATP-dependent RNA h	orf19.7213	889	CaAPL1	PROTEIN I	molecular_function unknown
CA5339	1.1	1.4	1.3	IPF885	16040381..	glucan 1,3-beta-glucos	orf19.7214	885	IPF889	CLASSIFIC	helicase activity
CA5340	1.0	1.0	0.9	IPF883	16042494..	unknown function	orf19.7215	883	IPF885	C-compour	molecular_function unknown
CA5341	1.2	1.1	1.1	HSP10.3	16048241..	10 kDa mitochondrial heat shock c		878	IPF883	UNCLASSI	RNA binding
CA5342	1.2	1.3	1.2	YPT521	16048974..	GTP-binding protein of	orf19.7216	877	CaHSP10.3	PROTEIN I	chaperone activity
CA5343	1.9	2.0	1.3	RPL4B	complemer	Ribosomal protein L4B	orf19.7217	875	CaYPT521	PROTEIN I	hydrolase activity
CA5344	0.8	2.6	0.9	PRY2	complemer	putative pathogen relat	orf19.7218	872	CaRPL4B	PROTEIN S	structural molecule activity
CA5345	2.6	4.2	2.2	FTR1	16056888..	high affinity iron perme	orf19.7219	868	CaPRY2	CELL FATI	molecular_function unknown
CA5346	1.0	0.9	1.1	IPF867	complemer	unknown function	orf19.7221	867	CaFTR1	REGULATI	transporter activity
CA5347	0.9	0.9	1.0	IPF864	complemer	unknown function	orf19.7222	864	IPF867	UNCLASSI	hydrolase activity
CA5348	1.0	1.0	1.1	IPF863	16064221..	involved in inositol bios	orf19.7223	863	IPF864	UNCLASSI	molecular_function unknown
CA5349	1.1	1.1	1.0	IPF861	16066337..	unknown function	orf19.7224	861	IPF863	C-compour	molecular_function unknown
CA5350	1.0	0.9	1.0	IPF859	16068549..	unknown function	orf19.7225	859	IPF861	UNCLASSI	molecular_function unknown
CA5351	1.0	0.9	0.8	IPF857	complemer	unknown function	orf19.7227	857	IPF859	No significant S.c. match	
CA5352	1.0	1.1	1.1	IPF856	16070891..	unknown function	orf19.7228	856	IPF857	UNCLASSI	molecular_function unknown
CA5353	1.0	0.9	1.1	IML2	complemer	unknown function	orf19.7229	855	IPF856	UNCLASSI	molecular_function unknown
CA5354	1.2	1.4	1.1	FTR2	complemer	high affinity iron perme	orf19.7231	853	CaIML2	UNCLASSI	molecular_function unknown
CA5355	1.1	0.9	1.0	IRR1.3F	complemer	cohesin complex subu	orf19.7232	850	CaFTR2	REGULATION OF/INTERACTION WITH CELLULAR ENVIRONMENT SUBCELLULAR LOCALISATION TRANSPORT FACILITATION UN	
CA5356	1.0	1.0	0.9	IRR1.5F	complemer	cohesin complex subu	orf19.7233	849	CaIRR1.3f	CELL CYCLE AND DNA PROCESSING SUBCELLULAR LOCALISATION	
CA5357	1.1	0.9	1.0	RSC8	16082295..	chromatin remodeling	orf19.7234	848	CaIRR1.5f	CELL CYC	protein binding
CA5358	1.0	0.9	1.0	IPF846	complemer	WD-repeat protein, bet	orf19.7235	846	CaRSC8	CELL CYC	molecular_function unknown
CA5359	1.1	1.1	1.0	TIF35	complemer	translation initiation fac	orf19.7236	842	IPF846	UNCLASSI	molecular_function unknown
CA5360	1.0	1.1	1.0	IPF839	16088591..	unknown function	orf19.7237	839	CaTIF35	PROTEIN S	translation regulator activity
CA5361	1.0	1.1	1.0	NPL3	complemer	nucleolar shuttling prot	orf19.7238	837	IPF839	UNCLASSI	molecular_function unknown
CA5362	1.3	1.6	0.9	IPF836.3	complemer	regulation of G-protein	orf19.7239	836	CaNPL3	TRANSCR	RNA binding
CA5363	0.9	0.7	1.2	NCR1	complemer	Polytopic membrane p	orf19.7242	829	IPF836.3	CELLULAF	DNA binding
CA5364	0.9	0.9	1.0	DCD1	16098318..	deoxycytidylate deamir	orf19.7243	825	CaNCR1	Lipid fatty-a	molecular_function unknown
CA5365	1.1	1.0	0.9	IPF824	complemer	2-hydroxyhepta-2,4-die	orf19.7244	824	CaDCD1	Nucleotide	hydrolase activity
CA5366	0.9	0.9	0.9	IPF823	16100311..	tRNA (5-methylaminon	orf19.7245	823	IPF824	UNCLASSI	molecular_function unknown
CA5367	0.9	1.0	1.0	RIM101	complemer	Zn finger transcription	orf19.7247	822	IPF823	UNCLASSI	transferase activity
CA5368	1.0	1.1	1.0	IPF5257	16105366..	unknown function	orf19.7250	5257	CaRIM101	CELL CYC	transcription regulator activity
CA5369	1.0	1.3	1.0	WSC4	complemer	Cell wall integrity by hc	orf19.7251	5256	IPF5257	UNCLASSIFIED PROTEINS	
CA5370	0.9	1.1	0.9	IPF5248	complemer	unknown function	orf19.7254	5248	CaWSC4	CELL RES	signal transducer activity
CA5371	1.0	0.8	1.1	RPC10	16110659..	DNA-directed RNA pol	orf19.7255	5246	IPF5248	UNCLASSI	molecular_function unknown
CA5372	1.0	1.1	1.0	MLH3	complemer	DNA mismatch repair I	orf19.7257	5245	CaRPC10	No significa	nucleotidyltransferase activity
CA5373	1.1	1.1	1.0	IPF5243	16112726..	snRNP (by homology)	orf19.7256	5243	CaMLH3	CELL CYC	molecular_function unknown
CA5374	1.0	1.0	1.0	DDI1	16113118..	Response to DNA alky	orf19.7258	5241	IPF5243	TRANSCR	RNA binding
CA5375	1.0	1.0	1.0	IPF5239	complemer	similarity to aldose red	orf19.7260	5239	CaDDI1	CELL RES	protein binding
CA5376	1.4	2.0	1.3	GDI1	complemer	GDP dissociation inhib	orf19.7261	5237	IPF5239	C-compound and carbohydrate metabolism	
CA5377	1.0	1.0	1.0	IPF5234	complemer	X-Pro dipeptidase (by I	orf19.7263	5234	CaGDI1	CELLULAF	enzyme regulator activity
CA5378	1.0	1.1	1.1	MPR1	16117776..	26S proteasome regula	orf19.7264	5231	IPF5234	PROTEIN I	peptidase activity
CA5379	1.0	1.1	1.0	IPF5228.5	16118769..	similar to Saccharomyc	orf19.7265	5228	CaMPR1	PROTEIN I	peptidase activity
CA5380	1.1	1.1	1.1	IPF5224	complemer	unknown function	orf19.7267	5224	IPF5228.5	TRANSCR	RNA binding
CA5381	1.0	1.1	1.1	IPF5222	complemer	arylalkylamine n-acetyl	orf19.7269	5222	IPF5224	No significant S.c. match	
CA5382	1.1	1.4	1.1	IPF5217	16122809..	unknown function	orf19.7270	5217	IPF5222	UNCLASSI	molecular_function unknown
CA5383	1.1	1.1	1.0	ZORRO1B	complemer	reverse transcriptase,	orf19.7273	14000	IPF5217	No significant S.c. match	
CA5384	0.9	0.9	1.0	ZORRO1B	complemer	reverse transcriptase,	orf19.7274	14001	CaZorro1b.	No significant S.c. match	
CA5385	1.1	1.0	1.0	ZORRO2B	complemer	reverse transcriptase,	orf19.7275	14003	CaZorro1b.	No significant S.c. match	
CA5386	0.9	1.0	1.0	ZORRO2B	complemer	Putative gag protein, 5	orf19.7277	14005	CaZorro2b.	No significant S.c. match	
CA5387	1.7	2.1	0.8	CTA24.3	complemer	transcriptional activator, 3-prime e		14006	CaZorro2b.	No significant S.c. match	
CA5388	0.9	1.0	0.7	PET9	complemer	ADP/ATP carrier prote	orf19.8545	6536	CaCTA24.3	No significant S.c. match	
CA5389	1.0	0.8	1.1	IFA13	complemer	unknown function	orf19.931	6533	CaPET9	Nucleotide	transporter activity
CA5390				DRS24	complement(16139225..16144375)				CaIFA13	C-compound and carbohydrate metabolism TRANSCRIPTION SUBCELLULAR LOCALISATION	
CA5391	1.0	1.0	1.0	IPF18109	16147634..	unknown function	orf19.934	18109			
CA5392	0.9	0.9	0.8	IPF10571	16158605..	Unknown function	orf19.935	10571	IPF18109	No significant S.c. match	
CA5393	1.0	1.9	1.0	IPF9398	complemer	unknown function	orf19.936	9398	IPF10571	No significant S.c. match	
CA5394	1.0	1.0	1.1	IPF9400	complemer	unknown function	orf19.937	9400	IPF9398	No significant S.c. match	
CA5395	1.0	0.9	1.0	IPF9401	complemer	unknown function	orf19.938	9401	IPF9400	UNCLASSIFIED PROTEINS	

CA5396	1.1	1.3	1.0	NAM7	complemer	nonsense-mediated m	orf19.939	9662	IPF9401	No significant S.c. match	
CA5397	1.0	1.1	1.0	IPF9663	16175902..	similar to Saccharomy	orf19.940	9663	CaNAM7	Nucleotide	helicase activity
CA5398	1.0	0.9	1.1	SEC14	16181246..	phosphatidylinositol(P	orf19.941	9666	IPF9663	CELL FATE	signal transducer activity
CA5399	1.0	0.8	1.0	KRE62.5F	16183259..	Glucan synthase subu	orf19.942	1538	CaSEC14	Lipid fatty-a	transporter activity
CA5400	1.0	1.0	1.0	KRE62.3F	16185108..	Glucan synthase subunit, 3-prime		1539	CaKRE62.5	C-compound and carbohydrate metabolism CELL FATE CONTROL OF CELLULAR ORGANIZATION SUBCELLULAR LOCALISATION	
CA5401	1.0	1.0	1.0	FET35.3	16185517..	Cell surface ferroxidas	orf19.943	1540	CaKRE62.3	No significant S.c. match	
CA5402	1.0	0.8	1.0	IFG3.3	16188612..	probable d-amino acid	orf19.944	1541	CaFET35.3	CELLULAR TRANSPORT AND TRANSPORT MECHANISMS REGULATION OF/INTERACTION WITH CELLULAR ENVIRONMENT SUB	
CA5403	0.9	0.9	0.9	IPF1542	16189271..	unknown function	orf19.945	1542	CaIFG3.3	No significant S.c. match	
CA5404	1.1	1.0	1.2	MET14	complemer	Adenylylsulfate kinase	orf19.946	1543	IPF1542	PROTEIN I	protein binding
CA5405	1.0	1.0	1.1	MRP17	16191360..	Mitochondrial ribosoma	orf19.947	1544	CaMET14	Amino acid	transferase activity
CA5406	1.0	1.1	1.1	IPF1547	16193043..	unknown function	orf19.949	1547	CaMRP17	PROTEIN S	structural molecule activity
CA5407	1.3	1.1	1.0	IPF1548	16197395..	unknown function	orf19.951	1548	IPF1547	PROTEIN FATE [folding modification destination] ""CELLULAR TRANSPORT AND TRANSPORT MECHANISMS CONTROL OF CELLUL	
CA5408	1.0	1.0	1.0	IPF1551	complemer	unknown function	orf19.952	1551	IPF1548	No significant S.c. match	
CA5409	0.5	1.0	1.0	COF1	complemer	cofilin (by homology)		1554	IPF1551	No significant S.c. match	
CA5410	1.0	1.0	1.0	IPF1557	16203444..	similar to dnaJ proteins	orf19.954	1557	CaCOF1	PROTEIN I	protein binding
CA5411	1.0	1.1	1.1	IPF1558	complemer	unknown function	orf19.955	1558	IPF1557	PROTEIN FATE [folding modification destination]	
CA5412	1.1	1.1	1.0	IPF1566	16206178..	unknown function	orf19.956	1566	IPF1558	No significant S.c. match	
CA5413	1.0	1.0	1.1	IPF1567	complemer	mitochondrial ribosomal protein L3		1567	IPF1566	UNCLASSI	molecular_function unknown
CA5414	1.0	1.0	1.0	IPF1568	16215716..	unknown function	orf19.962	1568	IPF1567	PROTEIN S	structural molecule activity
CA5415	1.0	1.0	0.9	SMC4	16216486..	Stable Maintenance of	orf19.964	1574	IPF1568	TRANSCR	nucleotidyltransferase activity
CA5416	1.0	0.9	1.0	IPF1576	complemer	unknown function	orf19.966	1576	CaSMC4	CONTROL	hydrolase activity
CA5417	1.1	1.0	1.1	NUC1	16222130..	Nuclease, mitochondri	orf19.967	1578	IPF1576	UNCLASSIFIED PROTEINS	
CA5418	1.1	1.8	1.1	IPF1580	complemer	unknown function	orf19.968	1580	CaNUC1	Nucleotide	RNA binding
CA5419	0.9	1.0	0.9	PRS1	complemer	Ribose-phosphate pyro	orf19.969	1582	IPF1580	UNCLASSI	molecular_function unknown
CA5420	1.1	1.0	1.0	IPF1583	16226306..	unknown function	orf19.970	1583	CaPRS1	Amino acid	transferase activity
CA5421	1.0	1.0	1.0	SKN7	complemer	Transcription factor (by	orf19.971	1586	IPF1583	UNCLASSI	molecular_function unknown
CA5422	1.0	1.0	1.0	IPF1588	16229370..	unknown function	orf19.972	1588	CaSKN7	CELL CYC	signal transducer activity,transcription regulator activity,DNA binding
CA5423	1.1	1.2	1.0	ROT2	complemer	Glucosidase II, catalyti	orf19.974	1591	IPF1588	UNCLASSI	molecular_function unknown
CA5424	1.2	1.3	1.0	IPF1598	16233612..	unknown function	orf19.976	1598	CaROT2	C-compour	hydrolase activity
CA5425	0.9	1.0	0.9	BDF1	16236647..	sporulation protein (by	orf19.978	1604	IPF1598	UNCLASSI	ligase activity
CA5426	1.2	1.2	1.7	FAS1	16239835..	Fatty-acyl-CoA synthas	orf19.979	1611	CaBDF1	TRANSCR	transcription regulator activity
CA5427				IPF1615.3	complement(16245992..16248238)				CaFAS1	Lipid fatty-a	lyase activity
CA5428	1.0	1.3	1.0	BOI2	16251930..	budding protein (by ho	orf19.3230	179			
CA5429	1.0	1.0	0.9	CDC27	16256263..	subunit of anaphase-p	orf19.3231	185	CaBOI2	CELL FATE	
CA5430	0.9	0.9	1.0	IPF191	complemer	putative permease (by	orf19.3232	191	CaCDC27	CELL CYC	protein binding
CA5431	0.6	0.3	0.8	IPF195	16266757..	unknown function	orf19.3233	195	IPF191	TRANSPORT FACILITATION	
CA5432	1.0	1.0	0.9	EBP5	complemer	NADPH dehydrogenas	orf19.3234	197	IPF195	CELLULAR	protein binding
CA5433	1.0	0.9	1.0	SFT1	16270617..	SNARE-like protein (by homology)		198	CaEBP5	ENERGY	transporter activity
CA5434	1.0	1.1	1.1	IPF199	complemer	F-actin capping protei	orf19.3235	199	CaSFT1	CELLULAR	transporter activity
CA5435	1.0	1.1	1.1	UFD4	complemer	ubiquitin fusion degrad	orf19.3237	204	IPF199	CELL FATE	protein binding
CA5436	1.0	1.0	1.0	CTF18	16284780..	chromosome transmiss	orf19.3239	217	CaUFD4	PROTEIN I	ligase activity
CA5437	0.9	1.0	1.0	ERG27	16287570..	3-keto sterol reductase	orf19.3240	218	CaCTF18	CELL CYC	molecular_function unknown
CA5438	1.1	1.2	1.1	IPF223	complemer	unknown function	orf19.3241	223	CaERG27	Lipid fatty-a	oxidoreductase activity
CA5439	1.1	1.1	1.0	TAF25	16293173..	transcription initiation f	orf19.3242	224	IPF223	UNCLASSI	molecular_function unknown
CA5440	1.0	0.9	0.8	SRP54	complemer	54 kD signal recognitio	orf19.3243	225	CaTAF25	TRANSCR	transcription regulator activity
CA5441	1.1	1.1	1.1	IPF227	16295956..	unknown function	orf19.3244	227	CaSRP54	PROTEIN FATE [folding modification destination] ""SUBCELLULAR LOCALISATION	
CA5442	1.2	1.1	1.1	IPF18105.3	complemer	unknown function, 3-pr	orf19.3245	228	IPF227	UNCLASSI	molecular_function unknown
CA5443	1.0	0.9	1.0	IPF18105.5	complemer	unknown function, 3-pr	orf19.3246	18105	IPF18105.3	No significant S.c. match	
CA5444	1.0	1.0	1.0	IPF230	complemer	unknown function	orf19.3247	230	IPF18105.5	No significant S.c. match	
CA5445	1.1	1.0	1.0	IPF232	complemer	unknown function	orf19.3248	232	IPF230	UNCLASSI	molecular_function unknown
CA5446	1.0	1.0	1.0	LAG1	complemer	longevity-assurance pr	orf19.3249	233	IPF232	UNCLASSIFIED PROTEINS	
CA5447	1.1	1.1	1.0	IPF234	16309970..	similar to Saccharomy	orf19.3250	234	CaLAG1	Lipid fatty-a	transporter activity
CA5448	1.0	1.0	1.0	ARC19.EX	16312095..	subunit of the Arp2/3 c	orf19.3251	235	IPF234	TRANSCR	RNA binding
CA5449	0.9	0.9	0.9	DAL81	complemer	Transcriptional activato	orf19.3252	236	CaARC19.e	CELLULAR	structural molecule activity
CA5450	1.0	0.9	1.1	IPF243	16316783..	unknown function	orf19.3254	243	CaDAL81	Amino acid	transcription regulator activity
CA5451	1.0	1.0	1.0	SLN1	16321174..	Two-component signal	orf19.3256	250	IPF243	No significant S.c. match	
CA5452	1.0	0.9	1.1	SEC11	complemer	signal peptidase subur	orf19.3259	252	CaSLN1	C-compour	signal transducer activity,protein kinase activity
CA5453	0.9	1.1	0.9	IPF256	16328198..	control of gene expres	orf19.3260	256	CaSEC11	PROTEIN I	peptidase activity
CA5454	1.1	1.0	1.1	IPF257.3	complemer	member of the FRP fa	orf19.3261	257	IPF256	CELL RES	lyase activity
CA5455	0.9	0.8	0.8	IPF263.3	complemer	member of the FRP fa	orf19.3263	263	IPF257.3	C-compound and carbohydrate metabolism	
CA5456	0.9	0.9	1.0	IPF267	complemer	unknown function	orf19.3264	267	IPF263.3	C-compound and carbohydrate metabolism	
CA5457	1.1	1.0	1.0	TRM1	complemer	N2,N2-dimethylguanin	orf19.3265	272	IPF267	CELL CYCLE AND DNA PROCESSING SUBCELLULAR LOCALISATION	
CA5458	0.8	0.8	0.8	IPF274	complemer	unknown function	orf19.3266	274	CaTRM1	TRANSCR	transferase activity
CA5459	1.0	1.0	1.0	IPF276	16347302..	unknown function	orf19.3267	276	IPF274	No significant S.c. match	
CA5460	1.2	1.4	1.1	IPF277	16348709..	human IgE-dependent	orf19.3268	277	IPF276	UNCLASSI	molecular_function unknown
CA5461	0.9	0.9	1.0	GSL23.5F	16350075..	1,3-beta-D-glucan synt	orf19.3269	279	IPF277	PROTEIN S	molecular_function unknown
CA5462	1.0	1.0	1.0	GSL23.3F	16352359..	1,3-beta-D-glucan synt	orf19.3270	282	CaGSL23.5	C-compound and carbohydrate metabolism SUBCELLULAR LOCALISATION	
CA5463	1.2	1.0	0.9	IPF284	complemer	unknown function	orf19.3272	284	CaGSL23.3	C-compound and carbohydrate metabolism CELL FATE SUBCELLULAR LOCALISATION	
CA5464	1.0	1.0	1.0	IPF285.5F	16356515..	unknown function, 5-pr	orf19.3273	285	IPF284	CELL RESCUE DEFENSE AND VIRULENCE ""CELL FATE	
CA5465	1.0	0.9	1.2	IPF285.3F	16357131..	unknown function, 3-pr	orf19.3274	286	IPF285.5f	UNCLASSI	molecular_function unknown
CA5466	1.0	1.0	1.0	PWP2	16358168..	periodic tryptophan pro	orf19.3276	290	IPF285.3f	UNCLASSIFIED PROTEINS	
CA5467	1.6	1.5	1.3	GSY1	16362931..	UDP glucose--starch g	orf19.3278	294	CaPWP2	CELL CYC	RNA binding

CA5468	0.7	0.7	1.0	IFF7	16365701..	unknown function	orf19.3279	296	CaGSY1	C-compour	transferase activity
CA5469	1.0	1.0	1.1	IPF298	16369927..	unknown function	orf19.3281	298	CaIFF7	UNCLASSIFIED PROTEINS	
CA5470	1.5	1.2	1.3	IPF300	16372420..	unknown function	orf19.3282	300	IPF298	UNCLASSI	molecular_function unknown
CA5471	0.9	0.9	0.9	IPF302	16374836..	short chain dehydroge	orf19.3283	302	IPF300	UNCLASSIFIED PROTEINS	
CA5472	0.9	0.9	1.1	IPF16948	complemer	unknown function	orf19.3285	16948	IPF302	Lipid fatty-a	oxidoreductase activity
CA5473	0.9	1.0	0.9	IPF16947	16377090..	unknown function	orf19.3286	16947	IPF16948	UNCLASSIFIED PROTEINS	
CA5474	1.0	0.9	0.9	IPF16944.3	complemer	unknown function, 3-pr	orf19.3287	16944	IPF16947	UNCLASSI	molecular_function unknown
CA5475	1.0	0.9	1.1	IPF8532	16385207..	unknown function	orf19.5303	8532	IPF16944.3	UNCLASSI	molecular_function unknown
CA5476	1.1	1.0	1.1	IPF8527	16388810..	unknown function	orf19.5305	8527	IPF8532	No significant S.c. match	
CA5477	1.2	1.1	1.1	IPF18101	complemer	unknown function	orf19.1276	18101	IPF8527	No significant S.c. match	
CA5478	1.0	1.0	1.0	JEN2	complemer	carboxylic acid transpc	orf19.1276	8196	IPF18101	No significant S.c. match	
CA5479	1.0	1.0	1.0	IPF8205	complemer	unknown function	orf19.5311	8205	CaJEN2	C-compound and carbohydrate metabolism TRANSPORT FACILITATION	
CA5480	1.0	1.1	1.0	IPF8210	complemer	unknown function	orf19.5312	8210	IPF8205	No significant S.c. match	
CA5481	1.0	1.0	1.0	IPF19810	complemer	unknown function	orf19.1277	19810	IPF8210	Amino acid	transcription regulator activity
CA5482	1.0	1.1	0.9	IPF14094.F	16424046..	repeated protein (10 tir	orf19.5315	7748	IPF19810	No significant S.c. match	
CA5483	1.0	0.9	1.0	IPF817	complemer	unknown function	orf19.5316	817	IPF14094.r	No significant S.c. match	
CA5484	1.1	1.0	1.0	RAD1.3F	complemer	UV endonuclease, con	orf19.5318	815	IPF817	UNCLASSIFIED PROTEINS	
CA5485	1.0	0.9	1.0	RAD1.53F	complemer	UV endonuclease, con	orf19.5319	18099	CaRAD1.3	CELL CYC	DNA binding
CA5486	1.0	1.0	0.9	IPF813	16434892..	unknown function	orf19.5320	813	CaRAD1.5	CELL CYCLE AND DNA PROCESSING SUBCELLULAR LOCALISATION	
CA5487	1.0	1.0	1.1	MET12	complemer	methylenetetrahydrofo	orf19.5321	812	IPF813	No significant S.c. match	
CA5488	1.1	1.1	1.0	IPF810	complemer	unknown function	orf19.5322	810	CaMET12	Metabolisr	oxidoreductase activity
CA5489	1.0	1.0	1.0	MDH12	16438979..	mitochondrial malate d	orf19.5323	807	IPF810	UNCLASSI	enzyme regulator activity
CA5490	1.1	0.9	1.0	KIN3	complemer	G2-specific serine/thre	orf19.5325	802	CaMDH12	C-compour	oxidoreductase activity
CA5491	1.0	1.1	1.0	IPF798	16443656..	transcriptional regulatc	orf19.5326	798	CaKIN3	CLASSIFIC	protein kinase activity
CA5492	1.2	1.1	1.0	GCN1.3F	complemer	translational activator,	orf19.5328	797	IPF798	C-compour	transcription regulator activity
CA5493	1.1	1.0	1.1	GCN1.5F	complemer	translational activator,	orf19.5333	793	CaGCN1.3	PROTEIN SYNTHESIS SUBCELLULAR LOCALISATION	
CA5494	1.0	1.1	1.1	IPF85	complemer	similar to Saccharomy	orf19.5334	785	CaGCN1.5	PROTEIN S	molecular_function unknown
CA5495	1.1	1.0	1.0	SGS1	16453723..	ATP-dependent DNA	orf19.5335	783	IPF85	TRANSCR	molecular_function unknown
CA5496	1.1	1.1	1.0	IPF779	16457414..	E2 ubiquitin conjugatin	orf19.5337	779	CaSGS1	CELL CYC	DNA binding,helicase activity
CA5497	2.0	1.6	1.4	IPF776	16459065..	transcriptional activato	orf19.5338	776	IPF779	PROTEIN FATE [folding modification destination]	
CA5498	1.2	1.1	1.1	IPF772	16460511..	unknown function	orf19.5340	772	IPF776	C-compound and carbohydrate metabolism TRANSCRIPTION SUBCELLULAR LOCALISATION	
CA5499	1.0	2.2	1.3	RPS4A	16462327..	ribosomal protein S4	orf19.5341	771	IPF772	PROTEIN S	enzyme regulator activity
CA5500	1.1	1.2	1.1	IPF768	16463208..	unknown function	orf19.5342	768	CaRPS4A	PROTEIN S	structural molecule activity
CA5501	1.2	1.0	1.1	MTR2.3	complemer	mRNA transport protein, 3-prime e		767	IPF768	UNCLASSI	molecular_function unknown
CA5502	0.7	0.7	1.0	IPF763	16467039..	putative transcription fa	orf19.5343	763	CaMTR2.3	TRANSCR	protein binding
CA5503	1.0	1.0	1.4	DSK2	16468574..	ubiquitin-like protein (b	orf19.5345	761	IPF763	TRANSCR	transcription regulator activity
CA5504	1.0	1.0	1.0	TAF65	16469753..	subunit of transcription	orf19.5346	758	CaDSK2	CELL CYCLE AND DNA PROCESSING SUBCELLULAR LOCALISATION	
CA5505	1.1	2.3	1.0	TPS3.3	16471311..	alpha,alpha-trehalose-	orf19.5348	753	CaTAF65	TRANSCR	transcription regulator activity
CA5506	0.9	0.8	1.1	IPF748	complemer	unknown function	orf19.5350	748	CaTPS3.3	C-compour	transferase activity
CA5507	0.8	0.8	1.0	TIF11	16475396..	translation initiation fac	orf19.5351	747	IPF748	TRANSCR	protein kinase activity
CA5508	0.7	0.6	1.2	IPF745	complemer	unknown function	orf19.5352	745	CaTIF11	PROTEIN S	translation regulator activity
CA5509	1.0	1.0	1.0	IPF743	16477379..	unknown function	orf19.5353	743	IPF745	UNCLASSIFIED PROTEINS	
CA5510	1.0	1.0	0.9	IPF741	complemer	unknown function	orf19.5356	741	IPF743	TRANSPORT FACILITATION	
CA5511	1.0	1.1	1.0	AKL1	complemer	serine/threonine protei	orf19.5357	737	IPF741	UNCLASSI	molecular_function unknown
CA5512	1.0	1.1	1.0	ORC2	complemer	origin recognition comp	orf19.5358	733	CaAKL1	CELL CYC	protein kinase activity
CA5513	1.0	1.0	1.0	RPC11	16485782..	RNA polymerase III C1	orf19.5360	731	CaORC2	CELL CYC	DNA binding
CA5514	1.0	0.9	1.0	IPF730	complemer	unknown function	orf19.5362	730	CaRPC11	TRANSCR	nucleotidyltransferase activity
CA5515	1.1	1.1	1.1	SAT2	complemer	putative glycosyl-transt	orf19.5363	728	IPF730	No significant S.c. match	
CA5516	0.9	1.1	0.9	IPF726	16488869..	rna binding protein (by	orf19.5364	726	CaSAT2	CELL RES	molecular_function unknown
CA5517	1.0	1.0	1.0	IPF721.3F	complemer	unknown function, 3-pr	orf19.5365	724	IPF726	CONTROL	molecular_function unknown
CA5518	1.0	1.1	1.0	IPF721.5F	complemer	unknown function, 5-pr	orf19.5366	721	IPF721.3f	UNCLASSIFIED PROTEINS	
CA5519	0.9	1.0	1.0	RDH54	16494038..	helicase required for m	orf19.5367	718	IPF721.5f	UNCLASSI	molecular_function unknown
CA5520	1.1	1.1	1.0	IPF714	complemer	unknown function	orf19.5368	714	CaRDH54	CELL CYC	DNA binding
CA5521	1.1	1.1	1.0	HEM12	complemer	uroporphyrinogen deca	orf19.5369	712	IPF714	UNCLASSI	molecular_function unknown
CA5522	1.1	1.3	1.1	IPF708	16499832..	unknown function	orf19.5370	708	CaHEM12	Metabolisr	lyase activity
CA5523	1.0	1.0	0.9	NIT2	complemer	Nitrilase (by homology	orf19.7279	2865	IPF708	UNCLASSIFIED PROTEINS	
CA5524	0.9	0.8	0.9	IPF2861	16509683..	putative pyruvate dehy	orf19.7281	2861	CaNIT2	Nitrogen ar	hydrolase activity
CA5525	0.9	1.0	0.9	PEX13	16512069..	Peroxisome import pro	orf19.7282	2859	IPF2861	Amino acid metabolism	
CA5526	0.8	0.8	1.1	IPF2857	complemer	unknown function	orf19.7284	2857	CaPEX13	PROTEIN I	protein binding
CA5527	0.9	0.9	0.9	IPF2856	complemer	unknown function	orf19.7285	2856	IPF2857	UNCLASSIFIED PROTEINS	
CA5528	1.0	1.0	1.2	RPN7	complemer	Subunit of the regulato	orf19.7286	2855	IPF2856	UNCLASSI	RNA binding
CA5529	0.6	0.8	0.6	IPF2852	16518952..	putative acetyl-coenzy	orf19.7288	2852	CaRPN7	PROTEIN I	peptidase activity
CA5530	1.1	1.1	1.0	SPB8	complemer	Suppressor of PAB1 (t	orf19.7290	2850	IPF2852	No significant S.c. match	
CA5531	0.9	1.0	1.0	GCD14	16521513..	Translational represso	orf19.7291	2848	CaSPB8	TRANSCR	RNA binding
CA5532	1.0	1.1	1.0	ARP2	16522913..	actin-like protein (by hc	orf19.7292	2847	CaGCD14	PROTEIN S	transferase activity
CA5533	1.0	1.1	0.9	IPF2846	complemer	similar to Saccharomy	orf19.7293	2846	CaARP2	CELL CYC	structural molecule activity
CA5534	0.9	1.0	0.9	IPF2843	complemer	unknown function	orf19.7295	2843	IPF2846	CELL CYC	protein kinase activity
CA5535	0.7	0.8	0.6	IPF2839	complemer	unknown function	orf19.7296	2839	IPF2843	PROTEIN I	protein binding
CA5536	1.0	1.1	1.0	IPF2837	complemer	putative cystathionine	orf19.7297	2837	IPF2839	No significant S.c. match	
CA5537	1.1	1.0	1.0	CHS2	complemer	Chitin synthase	orf19.7298	2836	IPF2837	Amino acid	lyase activity
CA5538	1.0	1.1	1.1	IPF2830	16539029..	unknown function	orf19.7300	2830	CaCHS2	C-compour	transferase activity
CA5541	1.1	1.3	1.3	IPF2827	complemer	unknown function	orf19.7303	2827	IPF2830	No significant S.c. match	

CA5542				IPF19812	complement(16543967..16545445)				IPF2827	No significant S.c. match	
CA5543	0.9	0.8	1.0	IPF5988	complemer	unknown function	orf19.7305	5988			
CA5544	0.9	0.8	1.0	IPF5987	complemer	unknown function	orf19.7306	5987	IPF5988	No significant S.c. match	
CA5545	1.0	1.1	1.0	IPF5986	complemer	similar to cytochrome-t	orf19.7307	5986	IPF5987	UNCLASSI	molecular_function unknown
CA5546	1.4	1.0	1.6	TUB1.3	complemer	Alpha-1 tubulin, 3-prim	orf19.7308	5983	IPF5986	ENERGY	molecular_function unknown
CA5547	1.5	1.6	1.1	IPF5981	complemer	similar to Saccharomy	orf19.7310	5981	CaTUB1.3	CELL CYC	structural molecule activity
CA5548	1.0	1.0	1.0	IPF5978	16557890..	unknown function	orf19.7311	5978	IPF5981	UNCLASSI	molecular_function unknown
CA5549	1.1	1.0	1.2	ERG13	complemer	3-hydroxy-3-methylglut	orf19.7312	5977	IPF5978	No significant S.c. match	
CA5550	0.9	1.1	1.0	SSU1	complemer	Sulfite sensitivity prote	orf19.7313	5976	CaERG13	Lipid fatty-a	transferase activity
CA5551	0.8	0.6	1.1	IPF5972	16565046..	putative cysteine dioxy	orf19.7314	5972	CaSSU1	CELL RES	transporter activity
CA5552	1.0	0.9	1.1	IPF5971	complemer	unknown function	orf19.7316	5971	IPF5972	No significant S.c. match	
CA5553	1.1	1.0	1.1	IPF1960.3f	complemer	putative transcriptional	orf19.7317	5969	IPF5971	No significant S.c. match	
CA5554	1.0	0.9	1.0	IPF1960.5f	complemer	putative transcriptional	orf19.7318	1960	IPF1960.3f	Nitrogen and sulphur metabolism TRANSCRIPTION SUBCELLULAR LOCALISATION	
CA5555	1.0	0.9	1.0	SUC1	16568981..	Putative zinc finger prc	orf19.7319	1962	IPF1960.5f	TRANSCRIPTION SUBCELLULAR LOCALISATION	
CA5556	0.9	0.9	0.9	LIP7	complemer	Secretory lipase	orf19.7320	1964	CaSUC1	C-compour	DNA binding
CA5557	0.9	0.9	0.9	IPF1968	complemer	unknown function	orf19.7321	1968	CaLIP7	Other virulence attributes	
CA5558	0.8	1.0	1.0	IPF1969	complemer	unknown function	orf19.7322	1969	IPF1968	UNCLASSI	molecular_function unknown
CA5559	1.1	1.2	0.9	CBP1	complemer	Corticosteroid binding	orf19.7323	1971	IPF1969	UNCLASSI	molecular_function unknown
CA5560	1.0	1.1	1.1	THI13	16579519..	Pyrimidine precursor b	orf19.7324	1974	CaCBP1	Metabolism of vitamins cofactors and prosthetic groups	
CA5561	1.2	1.1	1.0	SCO1	complemer	Inner mitochondrial me	orf19.7325	1975	CaTHI13	Nucleotide	molecular_function unknown
CA5562				IPF1977	16582043..16583782				CaSCO1	PROTEIN I	molecular_function unknown
CA5563	1.0	1.0	1.0	PHO88	complemer	Involved in phosphate	orf19.7327	1978			
CA5564	1.0	1.1	1.0	IPF1980	complemer	unknown function	orf19.7328	1980	CaPHO88	Phosphate	transporter activity
CA5565	0.9	0.9	0.9	QRI8	16588317..	E2 ubiquitin-conjugatic	orf19.7329	1981	IPF1980	No significant S.c. match	
CA5566	1.0	0.9	1.0	PET18	complemer	Putative transcriptional	orf19.7330	1983	CaQRI8	PROTEIN I	ligase activity
CA5567	0.9	0.8	1.0	FCY24	16590399..	Putative purine-cytosin	orf19.7331	1985	CaPET18	TRANSCR	molecular_function unknown
CA5568	0.9	1.0	1.0	ELF1	16592349..	Elongation-like factor	orf19.7332	1989	CaFCY24	Nucleotide metabolism TRANSPORT FACILITATION	
CA5569	1.1	1.1	1.2	PRE8	16596456..	20S proteasome subur	orf19.7335	1990	CaELF1	PROTEIN S	transporter activity,hydrolase activity
CA5570	1.0	0.9	1.0	IPF1992	complemer	putative MFS transport	orf19.7336	1992	CaPRE8	PROTEIN I	peptidase activity
CA5571	1.0	1.0	1.0	IPF2001	16607554..	unknown function	orf19.7337	2001	IPF1992	CELL RESCUE DEFENSE AND VIRULENCE ""TRANSPORT FACILITATION	
CA5572	1.1	0.9	1.1	BGL22	complemer	endo-beta-1,3-glucana	orf19.7339	2004	IPF2001	No significant S.c. match	
CA5573	1.1	1.0	1.0	IPF2005	complemer	unknown function		2005	CaBGL22	No significant S.c. match	
CA5574	1.2	1.1	1.1	IPF2007	complemer	unknown function	orf19.7341	2007	IPF2005	No significant S.c. match	
CA5575	0.6	0.6	0.7	IPF19813	16624459..	unknown function	orf19.7342	19813	IPF2007	No significant S.c. match	
CA5576	0.9	0.9	0.8	PRP4	16629096..	pre-mRNA-processing	orf19.7343	12538	IPF19813	PROTEIN FATE [folding modification destination] ""CELL FATE	
CA5577	1.0	1.0	1.0	IPF12537	16630917..	unknown function	orf19.7344	12537	CaPRP4	TRANSCR	RNA binding
CA5578	1.0	1.0	1.0	IPF12536	complemer	unknown function	orf19.7345	12536	IPF12537	UNCLASSI	molecular_function unknown
CA5579	0.9	0.9	0.9	IPF5661	complemer	unknown function	orf19.7125	5661	IPF12536	UNCLASSI	molecular_function unknown
CA5580	1.4	1.5	1.1	RVS161	complemer	cytoskeletal binding pr	orf19.7124	5657	IPF5661	No significant S.c. match	
CA5581	1.2	0.9	1.1	RAD3	complemer	DNA helicase/ATPase	orf19.7119	5649	CaRVS161	CELLULAF	protein binding
CA5582	1.2	1.2	1.0	ADK2	complemer	adenylate kinase, mito	orf19.7118	5646	CaRAD3	CELL CYC	DNA binding,helicase activity
CA5583	1.0	1.0	0.9	IPF5644	complemer	unknown function	orf19.7116	5644	CaADK2	Nucleotide	transferase activity
CA5584	1.2	1.2	1.1	SAC7	complemer	GAP for RHO1 by hom	orf19.7115	5642	IPF5644	PROTEIN I	transcription regulator activity
CA5585	0.8	1.2	0.6	CSA1	complemer	mycelial surface antige	orf19.7114	5641	CaSAC7	PROTEIN I	signal transducer activity
CA5586	0.2	0.3	0.2	IPF5625	16649726..	unknown function	orf19.7112	5625	CaCSA1	No significant S.c. match	
CA5588	6.1	4.9	3.3	SOD22.3F	16651661..	superoxide dismutase, 3-prime en		5622	IPF5625	REGULATION OF/INTERACTION WITH CELLULAR ENVIRONMENT Other virulence attributes	
CA5589	1.6	1.1	1.6	IPF5621	16652423..	unknown function	orf19.7111	5621	CaSOD22.	CELL RESCUE DEFENSE AND VIRULENCE ""SUBCELLULAR LOCALISATION	
CA5590	1.0	1.1	1.0	TAD1	16652934..	Double-stranded rna sp	orf19.7110	5619	IPF5621	CONTROL	molecular_function unknown
CA5591	1.2	1.1	1.2	IPF5618	complemer	unknown function	orf19.7109	5618	CaTAD1	TRANSCR	hydrolase activity
CA5593	1.3	1.3	1.1	IPF5615	16654858..	unknown function	orf19.7107	5615	IPF5618	UNCLASSI	molecular_function unknown
CA5594	1.0	0.9	1.0	IPF19814	16656751..	folate hydrolase (by hc	orf19.7106	19814	IPF5615	UNCLASSI	molecular_function unknown
CA5595	1.0	1.0	1.0	IPF568	16659215..	similar to Saccharomy	orf19.7105	568	IPF19814	REGULATI	molecular_function unknown
CA5596	1.1	1.0	1.0	IPF564	16661741..	unknown function	orf19.7104	564	IPF568	CELL CYC	enzyme regulator activity
CA5597	1.0	1.0	1.1	IPF563	16662817..	unknown function	orf19.7103	563	IPF564	No significant S.c. match	
CA5598	1.1	1.1	1.1	IPF562	complemer	unknown function	orf19.7101	562	IPF563	UNCLASSI	molecular_function unknown
CA5599	1.1	1.0	1.1	IPF560	16666230..	unknown function	orf19.7102	560	IPF562	SUBCELLU	DNA binding
CA5600	1.0	1.1	1.0	IPF559	16667458..	unknown function	orf19.7100	559	IPF560	UNCLASSIFIED PROTEINS	
CA5601	0.9	0.9	1.0	IPF556	16669489..	transcriptional regulatc	orf19.7098	556	IPF559	TRANSPO	transporter activity
CA5602	1.0	1.0	1.0	IPF554	complemer	RNA binding protein (b	orf19.7097	554	IPF556	TRANSCR	molecular_function unknown
CA5603	1.0	1.0	1.0	IPF553	complemer	unknown function	orf19.7096	553	IPF554	TRANSCRIPTION	
CA5604	0.9	1.0	1.0	IPF549	16674186..	unknown function	orf19.7095	549	IPF553	UNCLASSI	molecular_function unknown
CA5605	1.0	0.9	0.9	SNF31	16677266..	high-affinity glucose tra	orf19.7094	546	IPF549	UNCLASSI	molecular_function unknown
CA5606	1.0	1.0	1.0	STL2.3F	complemer	sugar transporter, 3-prime end (by		542	CaSNF31	C-compound and carbohydrate metabolism CELLULAR TRANSPORT AND TRANSPORT MECHANISMS REGULATION OF/INTERACTIC	
CA5607	1.1	1.0	1.0	STL2.5F	complemer	sugar transporter, 5-pr	orf19.7093	541	CaSTL2.3f	C-compound and carbohydrate metabolism SUBCELLULAR LOCALISATION TRANSPORT FACILITATION	
CA5608	1.1	1.2	1.0	IPF539	16681988..	unknown function	orf19.7092	539	CaSTL2.5f	C-compound and carbohydrate metabolism SUBCELLULAR LOCALISATION TRANSPORT FACILITATION	
CA5609	0.9	1.0	1.1	IPF538	complemer	unknown function	orf19.7091	538	IPF539	UNCLASSI	molecular_function unknown
CA5610	0.9	1.0	0.9	PMR1	complemer	calcium/mangenease F	orf19.7089	534	IPF538	No significant S.c. match	
CA5611	1.0	1.0	1.1	SUA5	complemer	translation initiation prc	orf19.7088	529	CaPMR1	CELLULAF	transporter activity
CA5612	1.2	1.1	1.1	KAP114	complemer	putative RAN-binding p	orf19.7086	527	CaSUA5	PROTEIN S	molecular_function unknown
CA5613	1.5	2.1	1.2	IPF525	complemer	unknown function	orf19.7085	525	CaKAP114	PROTEIN I	protein binding
CA5614	1.1	2.1	1.3	IPF522	16694750..	unknown function	orf19.7084	522	IPF525	No significant S.c. match	
CA5615	1.1	1.0	1.0	IPF520	16695958..	unknown function	orf19.7083	520	IPF522	No significant S.c. match	

CA5616	1.1	1.1	1.2	PET8	16697116..	mitochondrial carrier p	orf19.7082	519	IPF520	CELL CYC	molecular_function unknown
CA5617	1.1	1.1	1.0	SPL1	16698157..	tRNA splicing protein	orf19.7081	518	CaPET8	CELLULAF	transporter activity
CA5618	0.9	0.9	1.1	LEU2	16699837..	isopropyl malate dehyc	orf19.7080	516	CaSPL1	REGULATI	lyase activity
CA5619	1.0	1.1	1.0	IPF514	complemer	similar to Saccharomy	orf19.7079	514	CaLEU2	Amino acid	oxidoreductase activity
CA5620	1.0	0.9	1.0	IPF511	complemer	unknown function	orf19.7078	511	IPF514	CELL FATI	molecular_function unknown
CA5621	0.9	0.8	1.2	FRE7	16706280..	Ferric reductase transr	orf19.7077	507	IPF511	No significant S.c. match	
CA5622	1.0	1.0	0.9	GBP2.3	complemer	single-strand telomeric	orf19.7076	506	CaFRE7	REGULATI	oxidoreductase activity
CA5623	0.9	0.9	1.0	IPF502	complemer	unknown function	orf19.7074	502	CaGBP2.3	PROTEIN I	RNA binding
CA5624	1.0	0.9	1.1	IPF501	16711299..	unknown function	orf19.7073	501	IPF502	UNCLASSI	molecular_function unknown
CA5625	1.0	1.0	1.0	PEL1	complemer	CDP-diacylglycerol-se	orf19.7072	499	IPF501	UNCLASSIFIED PROTEINS	
CA5626	1.0	1.0	1.0	IPF498	16715125..	unknown function	orf19.7071	498	CaPEL1	Lipid fatty-a	transferase activity
CA5627	0.9	1.1	1.0	IPF495	complemer	unknown function	orf19.7069	495	IPF498	Phosphate metabolism CELLULAR TRANSPORT AND TRANSPORT MECHANISMS REGULATION OF/INTERACTION WITH CELLULAF	
CA5628	1.0	0.9	1.0	MAC1	16718263..	putative metal-binding	orf19.7068	492	IPF495	UNCLASSI	transferase activity
CA5629	1.0	1.0	1.0	CTR9	16719856..	required for G1 cyclin	orf19.7067	489	CaMAC1	TRANSCRIPTION	
CA5630	1.0	1.0	1.0	IPF486	complemer	unknown function	orf19.7065	486	CaCTR9	CELL CYC	DNA binding
CA5631	0.8	0.9	0.6	GLN4	complemer	glutaminyl-tRNA synth	orf19.7064	485	IPF486	UNCLASSI	DNA binding
CA5632	1.2	1.2	1.1	IPF480	complemer	unknown function	orf19.7063	480	CaGLN4	PROTEIN	ligase activity
CA5633	1.1	1.2	1.0	RPA135	16727850..	DNA-directed RNA pol	orf19.7062	479	IPF480	UNCLASSI	molecular_function unknown
CA5634	0.9	1.0	1.0	IPF477	complemer	unknown function	orf19.7061	477	CaRPA135	TRANSCR	nucleotidyltransferase activity
CA5635	1.0	0.9	1.0	IPF474	16732607..	unknown Function	orf19.7060	474	IPF477	UNCLASSI	molecular_function unknown
CA5636	0.7	0.5	0.9	IPF473	complemer	unknown Function	orf19.7059	473	IPF474	No significant S.c. match	
CA5637	0.9	0.9	0.8	IPF472	complemer	unknown Function	orf19.7058	472	IPF473	UNCLASSI	molecular_function unknown
CA5638	1.0	1.0	1.0	IPF470	16737308..	putative glutamine-tRN	orf19.7057	470	IPF472	UNCLASSI	molecular_function unknown
CA5639	0.9	1.1	1.0	DIP53.EXC	complemer	dicarboxylic amino aci	orf19.7056	469	IPF470	PROTEIN	transferase activity
CA5641	2.3	2.2	1.5	GAC1	complemer	ser/thr phosphoprotein	orf19.7053	461	CaDIP53.e	No significant S.c. match	
CA5642	1.1	1.0	1.0	INP52	complemer	phosphatidylinositol ph	orf19.7052	451	CaGAC1	C-compour	protein phosphatase activity
CA5643	1.0	1.0	1.0	IPF448	16762240..	unknown function	orf19.7051	448	CaINP52	Lipid fatty-a	hydrolase activity
CA5644	0.9	0.9	0.9	IPF447	16763058..	unknown function	orf19.7050	447	IPF448	UNCLASSI	molecular_function unknown
CA5645	0.8	1.0	0.8	CYB5	16764907..	Cytochrome b5 (by hor	orf19.7049	443	IPF447	UNCLASSI	molecular_function unknown
CA5646	1.1	1.0	1.1	RPS28B.3	16767339..	Ribosomal protein S28B (S33B) (Y		440	CaCYB5	Lipid fatty-a	transporter activity
CA5647	1.0	1.0	1.0	RTF1.5EO	16768246..	Nuclear protein regulating DNA bir		439	CaRPS28E	UNCLASSI	structural molecule activity
CA5648	1.1	1.1	1.0	UBC6	16770989..	E2 ubiquitin-conjugatin	orf19.7347	7116	CaRTF1.5e	TRANSCRIPTION SUBCELLULAR LOCALISATION	
CA5649	0.9	1.1	1.1	CHS4	16776234..	Chitin synthase regula	orf19.7349	7112	CaUBC6	PROTEIN FATE [folding modification destination] ""CELL FATE SUBCELLULAR LOCALISATION	
CA5650	1.3	2.4	1.4	IPF7109	16780282..	unknown function	orf19.7350	7109	CaCHS4	C-compour	enzyme regulator activity
CA5651	1.1	1.1	1.0	KIP3	complemer	Kinesin-related protein	orf19.7353	7105	IPF7109	UNCLASSI	molecular_function unknown
CA5652	0.9	0.9	1.1	IPF19815	complemer	longevity-assurance pr	orf19.7354	19815	CaKIP3	CELL CYC	motor activity
CA5653	1.0	1.0	0.9	SSN8	complemer	C-type cyclin associate	orf19.7355	15544	IPF19815	CELL FATE	
CA5654	0.9	1.0	1.0	IPF15543	complemer	unknown function	orf19.7356	15543	CaSSN8	C-compour	transcription regulator activity
CA5655	1.0	0.9	1.0	IPF18080	16791138..	unknown function	orf19.7357	18080	IPF15543	No significant S.c. match	
CA5656	1.1	1.1	1.0	IPF14683	complemer	unknown function	orf19.7358	14683	IPF18080	UNCLASSI	ligase activity
CA5657	1.0	1.0	1.1	IPF14682	complemer	putative transcription f	orf19.7359	14682	IPF14683	UNCLASSI	molecular_function unknown
CA5658	1.0	1.1	1.0	IPF1242	complemer	unknown function	orf19.7360	1242	IPF14682	TRANSCR	DNA binding,transcription regulator activity
CA5659	1.0	1.0	0.9	SEN54	16799988..	tRNA splicing endonuc	orf19.7361	1243	IPF1242	UNCLASSI	molecular_function unknown
CA5660	1.1	1.0	1.0	SKN1.3	complemer	Glucan synthase subu	orf19.7362	1245	CaSEN54	TRANSCR	RNA binding
CA5661	1.0	1.1	1.0	KRE6	16807297..	Glucan synthase subu	orf19.7363	1249	CaSKN1.3	C-compound and carbohydrate metabolism CELL FATE CONTROL OF CELLULAR ORGANIZATION SUBCELLULAR LOCALISATION	
CA5662	1.0	1.0	1.1	IPF1250	16811045..	Flavin-containing mon	orf19.7364	1250	CaKRE6	C-compour	hydrolase activity
CA5663	1.0	1.1	1.0	IPF1251	16813355..	unknown function	orf19.7365	1251	IPF1250	Nitrogen and sulphur metabolism	
CA5664	1.1	1.0	1.0	IPF1252	complemer	Conserved hypothetica	orf19.7366	1252	IPF1251	UNCLASSI	molecular_function unknown
CA5665	0.8	0.6	1.0	UBP1	complemer	Ubiquitin-specific prote	orf19.7367	1255	IPF1252	UNCLASSI	molecular_function unknown
CA5666	1.2	1.0	1.2	PUB1	complemer	Major polyadenylated	orf19.7368	1257	CaUBP1	PROTEIN I	peptidase activity
CA5667	0.9	0.8	1.0	IPF1259	16823061..	Conserved hypothetica	orf19.7369	1259	CaPUB1	TRANSCRIPTION SUBCELLULAR LOCALISATION	
CA5668	0.9	0.8	1.0	IPF1261	16824896..	unknown function	orf19.7370	1261	IPF1259	UNCLASSI	transferase activity
CA5669	1.0	1.0	1.0	IPF1264	16826657..	unknown function, Asn	orf19.7371	1264	IPF1261	UNCLASSI	molecular_function unknown
CA5670	1.0	0.9	1.1	IPF1266	16830467..	Probable transcription	orf19.7372	1266	IPF1264	TRANSCRIPTION SUBCELLULAR LOCALISATION	
CA5671	0.8	0.9	0.7	CTA4	16835029..	Probable transcription	orf19.7374	1269	IPF1266	Lipid fatty-acid and isoprenoid metabolism ""TRANSCRIPTION SUBCELLULAR LOCALISATION	
CA5672	0.9	0.9	0.8	IPF1271	complemer	Small nuclear ribonucl	orf19.7375	1271	CaCTA4	Lipid fatty-a	DNA binding
CA5673	0.9	0.8	0.9	IPF1272	16838990..	unknown function	orf19.7376	1272	IPF1271	TRANSCRIPTION ""PROTEIN FATE [folding modification destination] ""	
CA5674	0.9	0.9	0.9	IPF1274	16841646..	similar to Saccharomy	orf19.7377	1274	IPF1272	TRANSCRIPTION	
CA5675	1.1	1.1	1.1	SIS2	complemer	Involved in cell cycle-s	orf19.7378	1276	IPF1274	CELL CYC	protein binding
CA5676	0.9	0.9	0.9	FAA22	complemer	Long-chain-fatty-acid--	orf19.7379	1278	CaSIS2	CELL CYC	lyase activity,enzyme regulator activity
CA5677	1.1	1.0	1.1	IPF1286	complemer	unknown function	orf19.7380	1286	CaFAA22	Lipid fatty-acid and isoprenoid metabolism ""CELLULAR TRANSPORT AND TRANSPORT MECHANISMS SUBCELLULAR LOCALISATIC	
CA5678	1.0	1.0	1.0	IPF1292	16863716..	unknown function Hyp	orf19.7381	1292	IPF1286	No significant S.c. match	
CA5680	0.9	1.2	1.1	CAM1.EXC	16867852..	translation elongation f	orf19.7382	1299	IPF1292	Amino acid metabolism Nitrogen and sulphur metabolism TRANSCRIPTION SUBCELLULAR LOCALISATION	
CA5681	0.8	0.9	0.9	MNN9	16869877..	Required for complex	orf19.7383	1301	CaCAM1.e	PROTEIN SYNTHESIS SUBCELLULAR LOCALISATION	
CA5682	0.9	0.8	0.9	NOG1	16871390..	Nucleolar G-protein (b	orf19.7384	1302	CaMNN9	C-compour	transferase activity
CA5683	1.0	1.0	1.0	IPF3333	16874917..	unknown function	orf19.7385	3333	CaNOG1	UNCLASSI	hydrolase activity
CA5684	1.1	1.0	0.9	IPF3331	complemer	unknown function	orf19.7386	3331	IPF3333	UNCLASSI	molecular_function unknown
CA5685	1.0	1.0	0.9	HPA1	16877752..	RNA polymerase II-ass	orf19.7387	3330	IPF3331	ENERGY "	molecular_function unknown
CA5686	1.0	1.0	1.1	IPF3329	complemer	similar to Saccharomy	orf19.7388	3329	CaHPA1	CELL CYC	transcription regulator activity
CA5687	1.0	1.1	1.0	REV3.5F	16881763..	DNA-directed DNA pol	orf19.7389	3327	IPF3329	C-compour	protein kinase activity
CA5689	1.0	1.0	1.0	REV3.3F	16885545..	DNA-directed DNA pol	orf19.7390	3325	CaREV3.5I	CELL CYC	nucleotidyltransferase activity
CA5690	1.0	0.9	0.9	OCH1	complemer	Alpha-1,6-mannosyltra	orf19.7391	3324	CaREV3.3I	CELL CYCLE AND DNA PROCESSING SUBCELLULAR LOCALISATION	

CA5691	1.0	1.3	1.1	DED1	complemer	RNA helicase (by hom	orf19.7392	3320	CaOCH1	C-compour	transferase activity
CA5692	1.1	0.9	1.0	UTR1	complemer	Associated with ferric r	orf19.7393	3316	CaDED1	TRANSCR	RNA binding
CA5693	1.0	1.0	1.0	GDA1	complemer	Golgi guanosine dipho	orf19.7394	3313	CaUTR1	REGULATI	transferase activity
CA5694	0.9	0.7	0.9	IPF3311	16898207..	unknown function	orf19.7396	3311	CaGDA1	PROTEIN I	hydrolase activity
CA5695	0.9	1.1	0.9	IPF3310	16900279..	unknown function	orf19.7397	3310	IPF3311	No significant S.c. match	
CA5696	1.0	0.9	0.9	IPF3309.3E	complemer	unknown function, 3-pr	orf19.7398	3309	IPF3310	UNCLASSI	molecular_function unknown
CA5697	1.2	1.6	1.2	IPF18076.3	complemer	thiol-specific antioxidant-like protei		18076	IPF3309.3e	UNCLASSIFIED PROTEINS	
CA5699	1.0	1.1	1.0	ALS7	complemer	agglutinin-like protein	orf19.7400	19816	IPF18076.3	CELL RESCUE DEFENSE AND VIRULENCE ""SUBCELLULAR LOCALISATION	
CA5700	1.0	0.9	1.0	IPF2630	16916566..	unknown function		2630	CaALS7	SUBCELLULAR LOCALISATION Other virulence attributes	
CA5701	1.0	1.1	1.0	ISW2	16917025..	Chromatin remodeling	orf19.7401	2633	IPF2630	No significant S.c. match	
CA5702	0.9	0.9	0.9	PCH1	complemer	Putative ATPase (by h	orf19.7402	2639	CaISW2	TRANSCR	hydrolase activity
CA5703	0.7	0.6	0.8	IPF2463	complemer	unknown function	orf19.7403	2643	CaPCH1	CELL CYC	transferase activity
CA5704	1.1	0.9	1.0	CHA11	complemer	L-serine/L-threonine de	orf19.7404	2644	IPF2463	UNCLASSI	molecular_function unknown
CA5705	1.0	0.8	1.0	IPF2645	16929431..	unknown function	orf19.7405	2645	CaCHA11	Amino acid	lyase activity
CA5706	0.9	1.0	1.0	IPF2649	16930675..	unknown function	orf19.7406	2649	IPF2645	No significant S.c. match	
CA5707	1.1	0.9	1.5	ERV25	complemer	Component of COPII-c	orf19.7409	2651	IPF2649	REGULATION OF/INTERACTION WITH CELLULAR ENVIRONMENT Other virulence attributes	
CA5708	0.3	0.1	1.0	MRPL39	16933753..	Mitochondrial 60S ribosomal prote		2652	CaERV25	CELLULAF	molecular_function unknown
CA5709	1.0	1.0	1.1	IPF2653	complemer	unknown function	orf19.7410	2653	CaMRPL39	PROTEIN S	structural molecule activity
CA5710	0.8	0.8	1.1	OAC1	16937716..	Mitochondrial oxaloace	orf19.7411	2654	IPF2653	UNCLASSI	molecular_function unknown
CA5711	1.0	0.9	1.0	MUB1	16939227..	Regulation of bud site	orf19.7412	2656	CaOAC1	CELLULAF	transporter activity
CA5712	1.1	1.1	1.0	IPF2657	16941769..	unknown function	orf19.7413	2657	CaMUB1	CELL FATE	molecular_function unknown
CA5713	1.0	0.9	1.0	ALS6	16946732..	agglutinin-like protein	orf19.7414	2663	IPF2657	CELL CYC	molecular_function unknown
CA5714	5.0	2.9	4.1	IPF2431	16952072..	similar to Saccharomy	orf19.7417	2431	CaALS6	CELL FATE SUBCELLULAR LOCALISATION Other virulence attributes	
CA5715	0.9	1.0	1.2	HNT2	16952914..	Diadenosine polyphos	orf19.7419	2434	IPF2431	CELL RES	oxidoreductase activity
CA5716	1.0	1.1	1.1	MED6	16953576..	RNA polymerase II tra	orf19.7420	2436	CaHNT2	Nucleotide	hydrolase activity
CA5717	1.4	1.6	1.3	CYP51	complemer	Cyclophilin - peptidylpr	orf19.7421	2438	CaMED6	TRANSCR	transcription regulator activity
CA5718	1.0	1.0	0.9	LCP5	complemer	Ngg1p interacting prot	orf19.7422	2440	CaCYP51	PROTEIN I	isomerase activity
CA5719	1.0	1.0	1.0	IPF2441	16956623..	unknown function	orf19.7424	2441	CaLCP5	TRANSCR	RNA binding
CA5720	1.1	1.2	1.2	UNG1	complemer	Uracil-DNA glycosylas	orf19.7425	2442	IPF2441	UNCLASSI	molecular_function unknown
CA5721	1.0	0.9	1.1	IPF2443	complemer	similar to Saccharomy	orf19.7426	2443	CaUNG1	CELL CYC	DNA binding
CA5722	1.2	1.1	1.0	IPF2446	complemer	unknown function	orf19.7427	2446	IPF2443	C-compour	transferase activity
CA5723	1.3	1.0	1.3	APN1	16962169..	AP endonuclease, exo	orf19.7428	2448	IPF2446	UNCLASSI	molecular_function unknown
CA5724	1.2	1.2	1.0	NUP116	16963243..	nuclear pore protein (b	orf19.7433	19817	CaAPN1	CELL CYC	DNA binding
CA5725	1.0	1.0	1.1	GLG2	16967618..	Self-glucosylating initi	orf19.7434	2460	CaNUP116	TRANSCR	structural molecule activity
CA5726	1.0	1.0	1.1	AAF1	16976907..	Adhesion and aggrega	orf19.7436	2468	CaGLG2	C-compound and carbohydrate metabolism ENERGY	
CA5727	1.0	0.9	0.9	ECM15	complemer	Involved in cell wall biogenesis an		2469	CaAAF1	No significant S.c. match	
CA5728	1.0	1.0	1.0	IPF2471	16981597..	maltose acetyltransfer	orf19.7437	2471	CaECM15	C-compour	molecular_function unknown
CA5729	1.1	1.1	1.1	UBA1	16982761..	Ubiquitin-activating en	orf19.7438	2475	IPF2471	C-compour	transferase activity
CA5730	1.0	1.0	1.0	HST6	complemer	ATP binding cassette	orf19.7440	2479	CaUBA1	PROTEIN FATE [folding modification destination] """"CELL RESCUE DEFENSE AND VIRULENCE ""SUBCELLULAR LOCALISATION	
CA5731	1.0	1.0	0.9	IPF2870	complemer	unknown function	orf19.7441	2870	CaHST6	CELLULAF	transporter activity,hydrolase activity
CA5733	1.1	1.1	1.0	IPF2873	16991767..	unknown function	orf19.7443	2873	IPF2870	UNCLASSIFIED PROTEINS	
CA5734	1.0	1.0	1.0	IPF20029	complemer	unknown function	orf19.7444	20029	IPF2873	No significant S.c. match	
CA5735	0.6	0.3	1.0	IPF2878	16996010..	unknown function	orf19.7445	2878	IPF20029	UNCLASSI	molecular_function unknown
CA5736	0.9	1.0	0.9	OPI3	complemer	Methylene-fatty-acyl-pl	orf19.7446	2880	IPF2878	PROTEIN I	molecular_function unknown
CA5737	0.9	0.8	0.7	JEN1	17001627..	Carboxylic acid transp	orf19.7447	2882	CaOPI3	Lipid fatty-a	transferase activity
CA5738	0.9	1.0	1.1	LYS9	17003715..	Lysine biosynthesis (b	orf19.7448	2883	CaJEN1	C-compour	transporter activity
CA5739	1.0	0.9	0.9	IPF2884	complemer	unknown function	orf19.7449	2884	CaLYS9	Amino acid	oxidoreductase activity
CA5740	1.1	1.0	1.0	IPF20030	17007896..	unknown function	orf19.7450	20030	IPF2884	UNCLASSI	molecular_function unknown
CA5741	1.0	1.0	1.0	IPF2891	17010126..	unknown function		2891	IPF20030	UNCLASSI	molecular_function unknown
CA5742	0.9	0.8	1.0	FUN31	17010633..	Serine/threonine kinas	orf19.7451	2894	IPF2891	No significant S.c. match	
CA5743	0.9	0.9	0.9	IPF2895	17015039..	unknown function	orf19.7452	2895	CaFUN31	UNCLASSI	protein kinase activity
CA5744	1.0	1.1	1.0	IPF2898	17017475..	unknown function	orf19.7453	2898	IPF2895	UNCLASSI	DNA binding
CA5745	0.9	0.9	1.0	TAF60	complemer	TATA-binding protein-a	orf19.7454	2900	IPF2898	No significant S.c. match	
CA5746	0.9	0.9	1.1	IPF2902	complemer	unknown function	orf19.7455	2902	CaTAF60	TRANSCR	transcription regulator activity
CA5747	1.0	1.0	1.0	IPF2903	complemer	unknown function	orf19.7456	2903	IPF2902	UNCLASSIFIED PROTEINS	
CA5748	1.1	0.9	1.0	IPF2905	complemer	unknown function	orf19.7457	2905	IPF2903	No significant S.c. match	
CA5749	0.8	1.1	1.0	IPF2908	complemer	unknown function	orf19.7459	2908	IPF2905	No significant S.c. match	
CA5750	1.1	1.1	1.0	CHS21	complemer	Chitin synthase (by ho	orf19.5384	11660	IPF2908	CELL CYC	molecular_function unknown
CA5751	1.0	0.9	0.9	VPS8	complemer	Vacuolar sorting protei	orf19.5387	11663	CaCHS21	C-compound and carbohydrate metabolism CELL CYCLE AND DNA PROCESSING CELL FATE SUBCELLULAR LOCALISATION	
CA5752	1.0	1.1	0.9	IPF1055	17040426..	unknown function	orf19.5388	1055	CaVPS8	PROTEIN I	molecular_function unknown
CA5753	1.0	1.0	1.1	FKH1	17043418..	Fork head protein type	orf19.5389	1059	IPF1055	CELL CYC	DNA binding,transcription regulator activity
CA5754	0.9	0.9	0.9	IPF1060	17045114..	unknown function, Asn	orf19.5390	1060	CaFKH1	TRANSCR	DNA binding,transcription regulator activity
CA5755	1.0	1.0	1.0	IPF1063	complemer	Spliceosomal protein S	orf19.5391	1063	IPF1060	No significant S.c. match	
CA5756	0.6	0.6	0.9	IPF1065	complemer	unknown function	orf19.5392	1065	IPF1063	TRANSCR	RNA binding
CA5757	1.2	1.3	1.1	IPF1067	complemer	Putative glutamate dec	orf19.5393	1067	IPF1065	CELL RESCUE DEFENSE AND VIRULENCE ""TRANSPORT FACILITATION	
CA5758	0.9	0.9	0.9	IPF1069	complemer	similar to Saccharomy	orf19.5395	1069	IPF1067	No significant S.c. match	
CA5759	1.0	1.1	1.0	PET191.3	17059892..	Assembly of cytochrome oxidase,		1071	IPF1069	ENERGY	molecular_function unknown
CA5760	1.1	1.0	1.1	IPF1072	complemer	unknown function	orf19.5397	1072	CaPET191	ENERGY "	molecular_function unknown
CA5761	1.0	1.1	1.0	IFF11	complemer	unknown function	orf19.5399	1076	IPF1072	UNCLASSI	molecular_function unknown
CA5762	0.9	0.9	0.9	IPF1084.3	complemer	unknown function, 3-pr	orf19.5401	1084	CaIFF11	No significant S.c. match	
CA5763	0.9	0.9	0.8	IFF10.5	complemer	unknown function, 5-pr	orf19.5404	1089	IPF1084.3	SUBCELLULAR LOCALISATION	
CA5764	1.1	1.0	1.1	IPF20031	17073688..	similar to Saccharomy	orf19.5406	20031	CaIFF10.5	No significant S.c. match	

CA5765	0.9	0.9	0.9	SOF1	17075690..	Involved in 18S pre-rR	orf19.5407	1095	IPF20031	PROTEIN I	protein phosphatase activity
CA5766	1.0	1.0	1.0	IPF1097	17078448..	serine/threonine protei	orf19.5408	1097	CaSOF1	TRANSCR	RNA binding
CA5767	1.1	1.0	1.1	IPF1098	17080829..	unknown function	orf19.5409	1098	IPF1097	UNCLASSI	protein kinase activity
CA5768	0.9	1.0	1.0	PAC1	17082296..	Similarity to human LIS	orf19.5410	1099	IPF1098	UNCLASSI	molecular_function unknown
CA5769	1.2	1.1	1.0	UBC12	complemer	E2 ubiquitin-conjugatin	orf19.5411	1101	CaPAC1	UNCLASSI	molecular_function unknown
CA5770	1.0	1.1	1.1	IPF1103	complemer	unknown function	orf19.5412	1103	CaUBC12	PROTEIN FATE [folding modification destination]	
CA5771	1.0	1.0	1.1	IPF1104	17085835..	similar to Saccharomyc	orf19.5413	1104	IPF1103	CONTROL	molecular_function unknown
CA5772	1.0	1.1	1.0	ESA1	complemer	Histone acetyltransfera	orf19.5416	1107	IPF1104	UNCLASSI	molecular_function unknown
CA5773	0.8	1.1	0.8	DOT5	17089881..	Derepression of telome	orf19.5417	1111	CaESA1	CELL CYC	transferase activity
CA5774	0.8	0.5	1.0	IPF1113	complemer	unknown function	orf19.5418	1113	CaDOT5	TRANSCR	oxidoreductase activity
CA5775	1.5	1.5	1.0	ATP5	complemer	F1F0-ATPase complex	orf19.5419	1114	IPF1113	UNCLASSI	molecular_function unknown
CA5776	1.1	1.1	1.0	RML2	17093624..	Ribosomal L2 protein,	orf19.5420	1116	CaATP5	ENERGY (	structural molecule activity
CA5777	1.1	1.1	1.0	IPF1118	complemer	unknown function	orf19.5422	1118	CaRML2	PROTEIN S	structural molecule activity
CA5778	1.0	1.0	0.9	IPF1119	complemer	unknown function	orf19.5423	1119	IPF1118	No significant S.c. match	
CA5779	1.0	1.0	1.0	IPF1121	complemer	unknown function	orf19.5425	1121	IPF1119	CELL CYC	DNA binding,nucleotidyltransferase activity
CA5780	1.0	1.0	1.0	IPF1123	17099376..	unknown function	orf19.5426	1123	IPF1121	UNCLASSI	RNA binding
CA5781	1.0	0.9	1.0	IPF1126	complemer	unknown function	orf19.5428	1126	IPF1123	UNCLASSI	molecular_function unknown
CA5782	0.9	0.9	1.1	IPF1127	17103226..	unknown function	orf19.5429	1127	IPF1126	CELL RES	transporter activity
CA5783	0.9	0.9	1.0	IPF1128	17105376..	unknown function	orf19.5430	1128	IPF1127	UNCLASSI	molecular_function unknown
CA5784	1.0	1.0	1.0	IPF1129	complemer	unknown function	orf19.5431	1129	IPF1128	No significant S.c. match	
CA5785	1.0	1.0	1.0	TPT1	complemer	tRNA 2 -phosphotransf	orf19.5432	1133	IPF1129	No significant S.c. match	
CA5786	0.9	1.0	1.1	IPF1134	complemer	unknown function	orf19.5433	1134	CaTPT1	TRANSCR	transferase activity
CA5787	1.1	1.1	1.0	IPF1136	complemer	unknown function	orf19.5436	1136	IPF1134	UNCLASSI	molecular_function unknown
CA5788	1.2	1.8	1.8	RHR2	complemer	DL-glycerol phosphata	orf19.5437	1139	IPF1136	UNCLASSI	RNA binding
CA5789	1.0	1.2	1.1	IPF1143	17115378..	Probable zinc-finger pr	orf19.5438	1143	CARHR2	C-compour	hydrolase activity
CA5790	1.0	1.0	1.0	IPF1144	complemer	unknown function	orf19.5439	1144	IPF1143	TRANSCR	DNA binding
CA5791	1.5	1.6	1.0	RPT2	complemer	26S proteasome regula	orf19.5440	4196	IPF1144	CELL CYCLE AND DNA PROCESSING SUBCELLULAR LOCALISATION	
CA5792	0.9	0.8	0.9	IPF4195	17119905..	similar to Saccharomyc	orf19.5441	4195	CaRPT2	No significa	peptidase activity
CA5793	1.0	1.6	0.9	IPF4192	17122664..	unknown function	orf19.5442	4192	IPF4195	CELL CYC	peptidase activity
CA5794	0.9	0.8	1.1	IPF4191	17124254..	unknown function	orf19.5443	4191	IPF4192	UNCLASSIFIED PROTEINS	
CA5795	1.0	1.0	1.0	TIM44	17125726..	mitochondrial inner me	orf19.5444	4189	IPF4191	Amino acid	oxidoreductase activity
CA5796	0.9	0.9	0.9	GLO3	17127143..	zinc finger protein	orf19.5445	4185	CaTIM44	PROTEIN I	transporter activity
CA5797	1.0	1.2	1.1	IPF4182	17128513..	unknown function	orf19.5446	4182	CaGLO3	CELL CYC	enzyme regulator activity
CA5798	1.1	1.0	1.0	IPF4181	complemer	putative permease (by	orf19.5447	4181	IPF4182	No significant S.c. match	
CA5799	1.0	0.9	1.0	IPF4176	17131743..	unknown function	orf19.5449	4176	IPF4181	C-compound and carbohydrate metabolism ""Lipid fatty-acid and isoprenoid metabolism ""CELLULAR TRANSPORT AND TRANSPORT M	
CA5800	1.0	1.0	0.9	IPF4175	17132515..	mitochondrial respirato	orf19.5450	4175	IPF4176	UNCLASSIFIED PROTEINS	
CA5801	0.9	1.1	0.9	DAL1	17133747..	allantoinase	orf19.5454	4171	IPF4175	TRANSCR	oxidoreductase activity
CA5802	0.8	0.9	0.9	IPF4164	17135626..	similar to Saccharomyc	orf19.5455	4164	CaDAL1	Nitrogen ar	hydrolase activity
CA5803	1.0	1.1	1.0	IPF4163	17136031..	unknown function	orf19.5457	4163	IPF4164	CELL FATI	molecular_function unknown
CA5804	0.8	0.8	0.8	IPF4160	17137539..	unknown function	orf19.5459	4160	IPF4163	UNCLASSI	molecular_function unknown
CA5805	1.1	1.1	1.0	IPF4153	17139185..	similar to Saccharomyc	orf19.5463	4153	IPF4160	SUBCELLU	molecular_function unknown
CA5806	0.9	1.0	0.9	IPF4149	complemer	unknown function	orf19.5465	4149	IPF4153	CELLULAF	molecular_function unknown
CA5807	1.4	1.0	1.3	RPS24	complemer	ribosomal protein S24.	orf19.5466	4146	IPF4149	SUBCELLU	molecular_function unknown
CA5810	1.1	1.4	1.1	IPF4137.3F	complemer	unknown function, 3-prime end		19666	CaRPS24	PROTEIN S	structural molecule activity
CA5811	1.1	1.1	1.0	IPF2690.5F	17150621..	unknown function, 5-pr	orf19.5469	2690	IPF4137.3f	No significant S.c. match	
CA5812	1.2	1.0	1.3	IPF2690.3F	17153754..	unknown function, 3-pr	orf19.5474	2681	IPF2690.5f	No significant S.c. match	
CA5813	1.0	1.0	1.1	IPF2522	17159664..	unknown function	orf19.7460	2522	IPF2690.3f	No significant S.c. match	
CA5814	0.9	1.1	0.9	IPF2521	complemer	putative protease (by h	orf19.7463	2521	IPF2522	UNCLASSI	molecular_function unknown
CA5815	0.9	1.1	1.0	IPF2517	complemer	putative protease (by h	orf19.7464	2517	IPF2521	PROTEIN I	peptidase activity
CA5816	1.7	1.7	1.5	ACC1	complemer	acetyl-coenzyme-A ca	orf19.7466	2516	IPF2517	PROTEIN FATE [folding modification destination]	
CA5817	1.0	0.9	1.0	IPF2511	complemer	unknown function	orf19.7468	2511	CaACC1	Lipid fatty-a	ligase activity
CA5818	0.9	1.4	0.9	ARG1	17175287..	argininosuccinate synth	orf19.7469	2508	IPF2511	UNCLASSI	molecular_function unknown
CA5819	1.0	1.0	0.9	IFF4	complemer	Unknown function	orf19.7472	2507	CaARG1	Amino acid	ligase activity
CA5820	0.9	1.0	1.0	IPF2500	17182862..	unknown function	orf19.7473	2500	CaIFF4	CELL CYCLE AND DNA PROCESSING CELL FATE	
CA5821	1.0	1.1	1.0	PHO81	complemer	Cyclin-dependent kinas	orf19.7475	2499	IPF2500	No significant S.c. match	
CA5822	0.9	0.8	1.2	YRB1	complemer	GTPase-activating pro	orf19.7477	2494	CaPHO81	Phosphate	enzyme regulator activity
CA5823	0.9	0.9	1.0	COQ1	complemer	Hexaprenyl pyrophosp	orf19.7478	2493	CaYRB1	TRANSCR	protein binding
CA5824	0.8	0.8	0.7	NTH1	complemer	Neutral trehalase	orf19.7479	2490	CaCOQ1	Lipid fatty-a	transferase activity
CA5825	1.2	1.0	1.1	IPF2489	complemer	unknown function	orf19.7480	2489	CaNTH1	C-compour	hydrolase activity
CA5826	0.7	1.3	0.6	MDH11	17198457..	Malate dehydrogenase	orf19.7481	2486	IPF2489	No significant S.c. match	
CA5827	1.0	1.1	1.0	IPF2485	complemer	unknown function	orf19.7482	2485	CaMDH11	C-compound and carbohydrate metabolism ENERGY SUBCELLULAR LOCALISATION	
CA5828	1.2	1.1	1.1	CRM1	17201081..	Nuclear export factor	orf19.7483	2484	IPF2485	CELLULAR TRANSPORT AND TRANSPORT MECHANISMS CONTROL OF CELLULAR ORGANIZATION	
CA5829	1.1	1.0	1.1	ADE1	17204785..	phosphoribosyl-amidoi	orf19.7484	2482	CaCRM1	TRANSCR	protein binding
CA5830	1.0	1.0	1.1	MRPL9	complemer	Mitochondrial ribosoma	orf19.7485	2480	CaADE1	Nucleotide	ligase activity
CA5831	1.0	1.0	1.0	MRPL6	complemer	ribosomal protein L6 pi	orf19.7486	438	CaMRPL9	PROTEIN S	structural molecule activity
CA5833	1.2	1.2	1.0	IMP3	complemer	U3 small nucleolar ribo	orf19.7488	436	CaMRPL6	PROTEIN S	structural molecule activity
CA5834	1.1	1.2	1.0	LRG1	17214014..	GTPase-activating pro	orf19.7489	434	CaIMP3	TRANSCR	RNA binding
CA5835	1.1	1.0	1.0	IPF429	17218782..	unknown function		429	CaLRG1	CELL FATI	enzyme regulator activity
CA5836	1.0	1.0	1.0	IPF428	complemer	transport protein (by hc	orf19.7490	428	IPF429	UNCLASSI	molecular_function unknown
CA5837	1.1	1.1	1.0	IPF426	complemer	unknown function	orf19.7491	426	IPF428	UNCLASSI	molecular_function unknown
CA5838	1.0	0.9	0.9	IPF424	17222636..	unknown function	orf19.7492	424	IPF426	CLASSIFIC	molecular_function unknown
CA5839	1.0	0.9	1.0	IPF423	complemer	unknown function	orf19.7494	423	IPF424	UNCLASSI	molecular_function unknown

CA5840	1.0	1.0	1.0	EBP6	complemer	NADPH dehydrogenas	orf19.7495	421	IPF423	UNCLASSI	molecular_function unknown
CA5841	0.9	1.0	0.7	IPF420	complemer	unknown function	orf19.7497	420	CaEBP6	ENERGY	
CA5842	1.0	1.0	1.0	LEU1	17234862..	3-isopropylmalate deh	orf19.7498	417	IPF420	UNCLASSI	protein binding
CA5843	1.0	0.9	1.0	IPF416	complemer	unknown function	orf19.7499	416	CaLEU1	Amino acid	lyase activity
CA5844	1.0	1.0	1.0	PXA1	17240440..	long chain fatty acid AI	orf19.7500	414	IPF416	UNCLASSI	nucleotidyltransferase activity
CA5845	1.0	0.9	0.8	NAP1	17243186..	nucleosome assembly	orf19.7501	412	CaPXA1	Lipid fatty-a	transporter activity,hydrolase activity
CA5846	1.8	1.5	1.4	IPF409	17249939..	unknown function	orf19.7502	409	CaNAP1	CELL CYC	protein binding
CA5847	1.0	0.9	1.1	CDA2	complemer	chitin deacetylase (by I	orf19.7503	408	IPF409	C-compound and carbohydrate metabolism CELLULAR TRANSPORT AND TRANSPORT MECHANISMS REGULATION OF/INTERACTIO	
CA5848	1.2	1.1	1.1	IPF407	complemer	unknown function	orf19.7504	407	CaCDA2	C-compour	hydrolase activity
CA5849	1.0	0.8	1.1	IPF404.5F	17257630..	unknown function, 5-pr	orf19.7506	404	IPF407	UNCLASSI	molecular_function unknown
CA5850	0.9	1.0	1.0	IPF404.3F	17258777..	unknown function, 3-pr	orf19.7507	403	IPF404.5f	SUBCELLULAR LOCALISATION	
CA5851	1.1	1.2	1.1	KIN2	complemer	ser/thr protein kinase (	orf19.7510	402	IPF404.3f	No significant S.c. match	
CA5852	1.1	1.0	1.2	ATP17.3	complemer	F1F0-ATPase complex, F1 delta s		396	CaKIN2	SUBCELLU	protein kinase activity
CA5853	1.0	1.0	1.0	LSM6	complemer	U6 snRNA-associated Sm-like pro		395	CaATP17.3	ENERGY "	transporter activity
CA5854	1.0	1.1	1.0	IPF393	complemer	similar to Saccharomy	orf19.7511	393	CaLSM6	TRANSCR	RNA binding
CA5855	1.0	0.9	0.9	ALK3	complemer	n-alkane inducible cyt	orf19.7512	389	IPF393	CELLULAF	structural molecule activity
CA5856	1.0	1.0	1.0	ALK2	complemer	n-alkane inducible cytc	orf19.7513	387	CaALK3	CELL RESCUE DEFENSE AND VIRULENCE ""CELL FATE CONTROL OF CELLULAR ORGANIZATION	
CA5857	1.8	1.7	1.4	PCK1	complemer	phosphoenolpyruvate c	orf19.7514	385	CaALK2	CELL RESCUE DEFENSE AND VIRULENCE ""CELL FATE CONTROL OF CELLULAR ORGANIZATION	
CA5858	1.0	0.9	1.0	IPF380	complemer	unknown function	orf19.7516	380	CaPCK1	C-compour	lyase activity
CA5859	1.0	1.0	1.0	CHT1	17281907..	endochitinase 1 precur	orf19.7517	377	IPF380	No significant S.c. match	
CA5860	1.0	1.0	1.0	IPF376	complemer	transcriptional regulatc	orf19.7518	376	CaCHT1	C-compound and carbohydrate metabolism CELL CYCLE AND DNA PROCESSING SUBCELLULAR LOCALISATION	
CA5861	1.0	1.1	1.0	IPF373	17285363..	unknown function	orf19.7519	373	IPF376	TRANSCRIPTION ""CELL RESCUE DEFENSE AND VIRULENCE ""SUBCELLULAR LOCALISATION	
CA5862	0.9	0.9	0.7	POT11	17286419..	peroxysomal 3-ketoacy	orf19.7520	372	IPF373	No significant S.c. match	
CA5863	1.0	0.9	1.0	IPF370	complemer	unknown function	orf19.7521	370	CaPOT11	Lipid fatty-a	transferase activity
CA5864	1.1	0.9	1.0	IPF364	17292399..	transaminase type I (b	orf19.7522	364	IPF370	No significant S.c. match	
CA5865	1.1	1.1	1.4	MKC1	17294244..	ser/thr protein kinase c	orf19.7523	363	IPF364	Nitrogen and sulphur metabolism	
CA5866	1.0	1.0	0.9	IPF361	complemer	unknown function	orf19.7527	361	CaMKC1	CELL CYC	protein kinase activity,signal transducer activity
CA5867	0.4	0.5	0.4	EPL1	17299106..	DNA-binding protein (b	orf19.7529	355	IPF361	No significant S.c. match	
CA5868	1.0	0.9	1.4	IPF351	17301483..	unknown function	orf19.7531	351	CaEPL1	UNCLASSI	transferase activity
CA5869	1.0	1.2	1.1	MIS12	complemer	mitochondrial C1-tetral	orf19.7534	349	IPF351	UNCLASSI	molecular_function unknown
CA5870	0.9	1.0	1.0	IPF345	complemer	FH1/FH2 involved in c	orf19.7537	345	CaMIS12	Amino acid metabolism Nucleotide metabolism C-compound and carbohydrate metabolism ""Metabolism of vitamins cofactors and prosth	
CA5871	0.9	1.0	1.0	PIF2	17309819..	DNA helicase (by hom	orf19.7538	336	IPF345	CELL CYCLE AND DNA PROCESSING CELL FATE SUBCELLULAR LOCALISATION	
CA5872	1.0	1.0	0.9	IPF333	complemer	unknown function	orf19.7539	333	CaPIF2	CELL CYC	DNA binding,helicase activity
CA5873	1.0	1.0	1.0	IPF331	17314831..	GPI-anchored cell surf	orf19.7542	331	IPF333	No significant S.c. match	
CA5874	1.0	1.0	0.9	CTA23	complemer	transcriptional activatic	orf19.7544	328	IPF331	No significant S.c. match	
CA5875	0.9	0.9	1.0	IPF324.3	17319171..	unknown function, , 3-p	orf19.7545	324	CaCTA23	No significant S.c. match	
CA5876	0.9	0.9	0.9	IPF12082.5	17327332..	bumetanide-sensitive N	orf19.6833	12082	IPF324.3	No significant S.c. match	
CA5877	1.0	0.9	1.0	IPF12082.3	17329088..	bumetanide-sensitive N	orf19.6832	12080	IPF12082.5	REGULATI	transporter activity
CA5878	1.0	1.0	1.0	PRP5	17331400..	pre-mRNA processing	orf19.6831	12079	IPF12082.3	REGULATION OF/INTERACTION WITH CELLULAR ENVIRONMENT TRANSPORT FACILITATION	
CA5879	0.9	0.8	0.8	IPF12076	17334373..	enoyl CoA hydratase (I	orf19.6830	12076	CaPRP5	TRANSCR	RNA binding,helicase activity
CA5880	1.0	0.8	1.0	IPF12074	17335442..	unknown function	orf19.6829	12074	IPF12076	CLASSIFICATION NOT YET CLEAR-CUT	
CA5881	0.9	1.0	1.1	SSS1	17337707..	ER protein-translocase complex su		2429	IPF12074	Phosphate	molecular_function unknown
CA5882	1.0	1.1	1.1	RRP1	17338576..	Involved in processing	orf19.6828	20032	CaSSS1	PROTEIN I	transporter activity
CA5883	1.0	1.1	1.1	IPF2425	17339844..	unknown function	orf19.6827	2425	CaRRP1	TRANSCR	molecular_function unknown
CA5884	1.0	1.0	1.0	SLF1	complemer	Copper homeostasis p	orf19.6826	2424	IPF2425	TRANSCR	protein binding
CA5885	1.0	0.9	1.0	IPF2419	17345064..	unknown function	orf19.6824	2419	CaSLF1	PROTEIN S	RNA binding
CA5886	0.9	0.8	1.0	IPF2417	complemer	unknown function	orf19.6822	2417	IPF2419	No significant S.c. match	
CA5887	1.0	1.0	0.9	IPF2415	17348282..	similar to Saccharomy	orf19.6821	2415	IPF2417	UNCLASSI	molecular_function unknown
CA5888	0.8	0.9	0.9	IPF2414	complemer	unknown function	orf19.6820	2414	IPF2415	CELL CYC	protein binding
CA5889	0.9	1.0	1.0	IPF2409	complemer	RNA-dependent ATPa	orf19.6818	2409	IPF2414	TRANSCRIPTION	
CA5890	0.8	0.7	1.1	FCR1	17364525..	Zinc cluster transcriptic	orf19.6817	2404	IPF2409	CLASSIFIC	helicase activity
CA5891	1.0	1.1	1.0	IPF2400	complemer	putative aldehyde redu	orf19.6816	2400	CaFCR1	TRANSCRIPTION	
CA5892	3.5	4.0	2.3	GAP1	17370939..	Glyceraldehyde-3-pho	orf19.6814	2397	IPF2400	C-compour	oxidoreductase activity
CA5893	1.1	1.1	1.1	IPF2392	17372227..	unknown function	orf19.6813	2392	CaGAP1	C-compour	oxidoreductase activity
CA5894	1.0	1.0	1.0	PMT2	17373364..	O-D-mannosyltransfera	orf19.6812	2391	IPF2392	No significant S.c. match	
CA5895	0.5	0.5	0.5	ISA2	17376082..	Mitochondrial protein n	orf19.6811	2389	CaPMT2	C-compour	transferase activity
CA5896	1.0	1.0	1.0	TPD3	complemer	Ser/thr protein phospha	orf19.6810	2388	CaISA2	REGULATI	molecular_function unknown
CA5897	0.9	1.0	1.0	IPF2384	17380149..	unknown function	orf19.6809	2384	CaTPD3	CELL CYC	protein phosphatase activity
CA5898	0.8	0.7	0.9	IPF2383	complemer	unknown function	orf19.6808	2383	IPF2384	ENERGY	molecular_function unknown
CA5899	0.9	1.0	1.0	IPF2382	17381350..	unknown function	orf19.6807	2382	IPF2383	No significant S.c. match	
CA5900	1.0	0.9	0.9	IPF2379	complemer	unknown function	orf19.6806	2379	IPF2382	PROTEIN FATE [folding modification destination] ""CELLULAR TRANSPORT AND TRANSPORT MECHANISMS	
CA5901	1.0	1.0	1.0	IPF2374	17388033..	unknown function	orf19.6805	2374	IPF2379	No significant S.c. match	
CA5902	1.0	1.0	1.0	SRP68	17391904..	SIGNAL RECOGNITIC	orf19.6804	5941	IPF2374	UNCLASSI	molecular_function unknown
CA5903	1.0	0.9	0.9	IPF5942	17393983..	transmembrane sugar	orf19.6803	5942	CaSRP68	PROTEIN FATE [folding modification destination] ""SUBCELLULAR LOCALISATION	
CA5904	1.0	1.0	1.1	IPF5944	17395480..	Unknown function	orf19.6802	5944	IPF5942	UNCLASSI	transporter activity
CA5905	1.0	1.0	0.9	RPD32	17396909..	histone deacetylase B	orf19.6801	5946	IPF5944	UNCLASSI	molecular_function unknown
CA5906	1.0	1.0	1.0	IPF5949	17399353..	unknown function	orf19.6800	5949	CaRPD32	Phosphate	hydrolase activity
CA5907	1.0	0.9	0.8	SSN6	17404006..	transcriptional repress	orf19.6798	5957	IPF5949	UNCLASSIFIED PROTEINS	
CA5908	0.9	1.1	1.0	IPF5960	complemer	unknown function	orf19.6797	5960	CaSSN6	TRANSCR	transcription regulator activity
CA5909	1.1	1.0	1.0	YSA1	complemer	sugar-nucleotide hydrc	orf19.6796	5962	IPF5960	No significant S.c. match	
CA5910	1.0	1.0	1.0	IPF5964	17411494..	unknown function	orf19.6795	5964	CaYSA1	Nucleotide	hydrolase activity
CA5911	0.9	0.9	1.0	IPF5965	17412612..	NADH-ubiquinone oxic	orf19.6794	5965	IPF5964	No significant S.c. match	

CA5912	1.0	1.1	1.0	IPF5966	complemer	unknown function	orf19.6793	5966	IPF5965	No significant S.c. match	
CA5913	1.1	1.1	1.1	RRD1	17416297..	Phosphotyrosyl phospl	orf19.6792	10731	IPF5966	No significant S.c. match	
CA5914	1.4	1.3	0.8	HHT3	17418335..	histone H3	orf19.6791	10733	CaRRD1	CELL CYC	protein phosphatase activity
CA5915	1.0	0.9	0.9	IPF10735	complemer	similar to Saccharomy	orf19.6790	10735	CaHHT3	TRANSCRIPTION SUBCELLULAR LOCALISATION	
CA5916	1.0	0.9	0.9	IPF19818	17424240..	similar to Saccharomy	orf19.6789	19818	IPF10735	TRANSCR	RNA binding
CA5917	1.0	1.0	1.1	IPF8923	17428344..	unknown function	orf19.6788	8923	IPF19818	CELL CYC	structural molecule activity
CA5918	0.9	1.0	1.0	ERV14	17429739..	membrane protein	orf19.6787	8924	IPF8923	CELLULAR TRANSPORT AND TRANSPORT MECHANISMS SUBCELLULAR LOCALISATION	
CA5919	0.9	1.1	0.9	IPF8926.3	complemer	unknown function, 3-pr	orf19.6786	8926	CaERV14	CELLULAF	molecular_function unknown
CA5920	1.0	1.1	1.2	RPS12	complemer	acidic ribosomal protei	orf19.6785	8927	IPF8926.3	No significant S.c. match	
CA5921	1.2	1.1	1.1	IPF8930	17433971..	unknown function	orf19.6784	8930	CaRPS12	PROTEIN S	structural molecule activity
CA5922	1.1	1.1	1.1	MRS6	complemer	geranylgeranyltransfer	orf19.6783	8931	IPF8930	No significant S.c. match	
CA5923	1.1	1.0	1.0	IFQ2	complemer	Unknown function	orf19.6782	2825	CaMRS6	Lipid fatty-a	enzyme regulator activity
CA5924	0.8	1.0	0.7	IPF2822	complemer	unknown function	orf19.6781	2822	CaIFQ2	No significant S.c. match	
CA5925	0.9	0.8	0.9	MET8	17448528..	Siroheme synthase (by	orf19.6780	2818	IPF2822	No significant S.c. match	
CA5926	1.1	1.1	1.1	PRO2	complemer	Proline biosynthetic en	orf19.6779	2817	CaMET8	Amino acid	lyase activity
CA5927	1.0	0.9	0.9	DRS22	17451300..	Membrane-spanning C	orf19.6778	2816	CaPRO2	Amino acid	oxidoreductase activity
CA5928	0.9	0.8	1.0	IPF2815	17456300..	unknown function	orf19.6777	2815	CaDRS22	CELLULAF	hydrolase activity
CA5929	1.0	1.1	1.0	GCD2	17457337..	Translation initiation fa	orf19.6776	2813	IPF2815	No significant S.c. match	
CA5930	0.9	0.8	1.1	ECM29.EX	complemer	Involved in cell wall bic	orf19.6773	2810	CaGCD2	PROTEIN S	translation regulator activity
CA5931	1.1	1.0	1.0	ECM29.EX	complemer	Involved in cell wall bic	orf19.6772	2807	CaECM29.	CONTROL	molecular_function unknown
CA5932	0.9	1.0	1.0	UBI4	complemer	Polyubiquitin	orf19.6771	2805	CaECM29.	CONTROL OF CELLULAR ORGANIZATION UNCLASSIFIED PROTEINS	
CA5933	1.1	0.9	1.3	IPF2804	complemer	unknown function	orf19.6770	2804	CaUBI4	No significant S.c. match	
CA5934	1.0	1.0	0.9	IPF2802	17469283..	unknown function	orf19.6769	2802	IPF2804	UNCLASSI	protein binding
CA5935	1.0	1.0	1.0	IPF2798	17471697..	unknown function	orf19.6766	2798	IPF2802	UNCLASSI	molecular_function unknown
CA5936	1.5	2.6	1.3	IPF2795	complemer	unknown function	orf19.6763	2795	IPF2798	TRANSCR	RNA binding
CA5937	1.0	1.0	1.1	IPF2784	17479036..	unknown function	orf19.6760	2784	IPF2795	CELL CYCLE AND DNA PROCESSING	
CA5938	0.9	0.9	1.0	IPF3481	17481199..	unknown function	orf19.6759	3482	IPF2784	CELL FATI	molecular_function unknown
CA5939	1.0	1.0	1.0	IPF3484	17483668..	aldo/keto reductase (by	orf19.6758	3484	IPF3481	CELL FATE	
CA5940	1.1	1.0	1.0	IPF3485	17484974..	aldo/keto reductase (by	orf19.6757	3485	IPF3484	C-compound and carbohydrate metabolism SUBCELLULAR LOCALISATION	
CA5941	1.1	1.1	1.2	IPF3486	17486121..	unknown function	orf19.6756	3486	IPF3485	C-compour	oxidoreductase activity
CA5942	1.1	1.0	1.0	DLD2	17487290..	D-lactate ferrycytochro	orf19.6755	3488	IPF3486	UNCLASSI	enzyme regulator activity
CA5943	1.0	0.8	1.1	IPF3490	17489734..	unknown function	orf19.6754	3490	CaDLD2	C-compour	oxidoreductase activity
CA5944	1.0	1.1	1.1	IPF3491	17490890..	unknown function	orf19.6753	3491	IPF3490	No significant S.c. match	
CA5945	0.9	0.9	0.9	IPF3492	complemer	unknown function	orf19.6752	3492	IPF3491	UNCLASSI	molecular_function unknown
CA5946	0.9	1.0	1.0	IPF3493	17492307..	unknown function	orf19.6751	3493	IPF3492	PROTEIN S	structural molecule activity
CA5947	1.1	1.0	1.2	KRS1	complemer	Lysyl-tRNA synthetase	orf19.6749	3495	IPF3493	TRANSCR	transferase activity
CA5948	0.9	1.0	1.0	IPF3496	17495321..	unknown function	orf19.6748	3496	CaKRS1	PROTEIN S	ligase activity
CA5949	1.0	1.0	1.0	IPF3498	17496843..	unknown function	orf19.6747	3498	IPF3496	No significant S.c. match	
CA5950	1.4	1.4	1.9	TPI1	17498034..	Triose phosphate isom	orf19.6745	3499	IPF3498	UNCLASSI	molecular_function unknown
CA5951	1.0	1.0	1.0	IPF3500	complemer	unknown function	orf19.6744	3500	CaTPI1	C-compour	isomerase activity
CA5952	0.9	0.9	1.0	IPF3503	17500349..	similar to Saccharomy	orf19.6742	3503	IPF3500	CELL CYC	molecular_function unknown
CA5953	1.1	0.9	1.1	IPF3506	17503763..	unknown function	orf19.6741	3506	IPF3503	TRANSCR	protein phosphatase activity
CA5954	0.9	0.9	1.0	IPF3508	17505368..	unknown function	orf19.6740	3508	IPF3506	UNCLASSIFIED PROTEINS	
CA5955	1.0	0.9	1.0	IPF3510	17506855..	unknown function	orf19.6739	3510	IPF3508	TRANSCR	RNA binding
CA5956	1.2	1.1	1.1	VAN1	17509387..	Vanadate resistance p	orf19.6738	3512	IPF3510	C-compour	molecular_function unknown
CA5957	0.9	1.1	1.0	RRP3.3EO	17511492..	RNA-dependent ATPa	orf19.7546	17103	CaVAN1	CELL CYC	transferase activity
CA5958	1.1	0.8	1.1	PIB1	complemer	phosphatidylinositol(3)	orf19.7547	17102	CaRRP3.3	TRANSCR	RNA binding,helicase activity
CA5959	1.0	1.0	1.0	SRB7	17514692..	DNA-directed RNA pol	orf19.7548	12936	CaPIB1	Lipid fatty-a	ligase activity
CA5960	0.9	1.0	1.1	PMT5	complemer	protein mannosyltransf	orf19.7549	12934	CaSRB7	TRANSCR	transcription regulator activity
CA5961	1.0	1.0	1.0	IFA14	complemer	unknown function	orf19.7550	12930	CaPMT5	C-compound and carbohydrate metabolism ""PROTEIN FATE [folding modification destination] ""SUBCELLULAR LOCALISATION	
CA5962	1.0	1.2	1.0	ALO1	17532037..	D-arabinono-1,4-lactor	orf19.7551	967	CaIFA14	No significant S.c. match	
CA5963	1.0	1.0	1.0	IPF966	complemer	unknown function	orf19.7552	966	CaALO1	C-compour	oxidoreductase activity
CA5964	1.1	0.9	0.9	IPF963	17537148..	unknown function	orf19.7553	963	IPF966	UNCLASSI	RNA binding
CA5965	0.9	0.9	1.0	IPF961	17538370..	drug resistance proteir	orf19.7554	961	IPF963	No significant S.c. match	
CA5966	1.0	1.0	1.0	IPF959	complemer	unknown function	orf19.7556	959	IPF961	CELL RESCUE DEFENSE AND VIRULENCE ""TRANSPORT FACILITATION	
CA5967	1.0	1.0	1.1	IPF955	17542095..	member of the AAA AT	orf19.7558	955	IPF959	No significant S.c. match	
CA5968	2.2	1.5	1.8	IPF946	17547457..	unknown function	orf19.7561	946	IPF955	PROTEIN I	molecular_function unknown
CA5969	1.0	1.1	1.0	BET2	complemer	beta subunit of geranyl	orf19.7563	941	IPF946	No significant S.c. match	
CA5970	1.0	1.0	0.9	DPB2	complemer	DNA-directed DNA pol	orf19.7564	940	CaBET2	Lipid fatty-a	transferase activity
CA5971	1.0	1.1	0.9	GNP2	17560024..	high affinity glutamine	orf19.7565	937	CaDPB2	CELL CYC	nucleotidyltransferase activity
CA5972	1.0	1.0	1.0	GNP1	17564170..	high affinity glutamine	orf19.7566	934	CaGNP2	Amino acid metabolism TRANSPORT FACILITATION	
CA5973	1.0	1.1	1.0	IPF931	17566719..	unknown function	orf19.7567	931	CaGNP1	Amino acid metabolism TRANSPORT FACILITATION	
CA5974	1.0	1.0	0.9	IPF930	17568313..	unknown function	orf19.7568	930	IPF931	UNCLASSI	molecular_function unknown
CA5975	0.9	1.1	1.0	SIK1	complemer	nucleolar protein involv	orf19.7569	929	IPF930	CELL FATI	molecular_function unknown
CA5976	0.7	0.9	0.5	IPF928	complemer	zinc-finger transcriptio	orf19.7570	928	CaSIK1	TRANSCR	molecular_function unknown
CA5977	1.1	1.0	1.0	UBC4.3	17575390..	E2 ubiquitin-conjugatin	orf19.7571	926	IPF928	Nitrogen ar	transcription regulator activity
CA5978	1.0	1.1	1.0	SPT7	17576602..	transcription factor, me	orf19.7572	925	CaUBC4.3	PROTEIN FATE [folding modification destination] ""CELLULAR TRANSPORT AND TRANSPORT MECHANISMS ""CELL RESCUE DEFEI	
CA5979	1.1	1.0	1.0	IPF921	17581159..	BTB domain and Anka	orf19.7574	921	CaSPT7	TRANSCR	structural molecule activity
CA5980	1.0	0.5	1.1	IPF918	complemer	unknown function	orf19.7576	918	IPF921	TRANSCRIPTION CELLULAR TRANSPORT AND TRANSPORT MECHANISMS CELL FATE SUBCELLULAR LOCALISATION	
CA5981	0.9	0.9	1.1	MSS51	complemer	involved in maturation	orf19.7577	917	IPF918	No significant S.c. match	
CA5982	1.1	1.1	1.1	IPF915	complemer	unknown function	orf19.7578	915	CaMSS51	PROTEIN S	molecular_function unknown
CA5983	1.1	1.0	0.9	IPF913	17589318..	unknown function	orf19.7579	913	IPF915	SUBCELLU	transporter activity

CA5984	1.0	0.9	0.9	CUS1	17591992..	spliceosome associate	orf19.7581	911	IPF913	No significant S.c. match	
CA5985	1.0	1.1	1.1	IPF907	complemer	unknown function	orf19.7583	907	CaCUS1	TRANSCR	protein binding,RNA binding
CA5986	1.1	1.0	1.2	INO1	complemer	myo-inositol-1-phospha	orf19.7585	904	IPF907	Lipid fatty-acid and isoprenoid metabolism ""TRANSCRIPTION SUBCELLULAR LOCALISATION	
CA5987	1.0	1.0	1.0	CHT3	17603077..	chitinase 3 precursor	orf19.7586	696	CaINO1	C-compour	isomerase activity
CA5988	1.0	1.0	1.0	IPF693	complemer	unknown function	orf19.7588	693	CaCHT3	C-compour	hydrolase activity
CA5989	1.0	1.0	0.9	IPF692	17605943..	unknown function	orf19.7589	692	IPF693	UNCLASSI	molecular_function unknown
CA5990	0.9	0.9	0.9	IPF690.5F	17607238..	NADH dehydrogenase	orf19.7590	690	IPF692	No significant S.c. match	
CA5991	1.1	0.9	1.0	IPF690.3F	17607789..	NADH dehydrogenase	orf19.7591	689	IPF690.5f	No significant S.c. match	
CA5992	1.3	1.2	0.6	FAA4	17613862..	long-chain fatty acid--C	orf19.7592	20033	IPF690.3f	No significant S.c. match	
CA5993	0.9	1.1	1.1	ASP1	complemer	L-asparaginase (by ho	orf19.7593	679	CaFAA4	Lipid fatty-a	ligase activity
CA5994	1.0	0.9	1.0	IPF677	17618102..	unknown function	orf19.7594	677	CaASP1	Amino acid	hydrolase activity
CA5995	1.1	1.2	1.0	IPF676	complemer	unknown function	orf19.7595	676	IPF677	No significant S.c. match	
CA5996	1.0	1.0	1.0	IPF673	17620784..	unknown function	orf19.7596	673	IPF676	No significant S.c. match	
CA5998	0.9	0.9	1.0	IPF670	17622301..	unknown function	orf19.7598	670	IPF673	ENERGY	
CA5999	1.0	0.9	1.1	IPF668	17625718..	unknown function	orf19.7599	668	IPF670	PROTEIN I	molecular_function unknown
CA6000	1.4	1.6	1.4	FDH11.3	complemer	glutathione-dependent	orf19.7600	666	IPF668	UNCLASSI	RNA binding
CA6001	1.0	1.0	1.1	IPF662	17629811..	unknown function	orf19.7601	662	CaFDH11.3	C-compour	oxidoreductase activity
CA6002	0.9	1.0	1.0	IPF661	complemer	unknown function	orf19.7602	661	IPF662	UNCLASSI	transferase activity
CA6003	1.0	1.0	1.0	IPF660	17632391..	unknown function	orf19.7603	660	IPF661	UNCLASSI	enzyme regulator activity
CA6005	1.0	1.1	1.1	PUP1	complemer	20S proteasom ebeta2	orf19.7605	654	IPF660	UNCLASSI	molecular_function unknown
CA6006	0.9	0.8	1.0	IPF652	17638868..	unknown function	orf19.7606	652	CaPUP1	PROTEIN I	peptidase activity
CA6007	1.0	0.9	1.0	IPF650	17640920..	unknown function	orf19.7608	650	IPF652	No significant S.c. match	
CA6008	1.0	4.2	1.0	IPF647	17642972..	unknown function	orf19.7609	647	IPF650	No significant S.c. match	
CA6009	1.4	1.6	1.9	IPF643	17648191..	similar to Saccharomy	orf19.7610	643	IPF647	No significant S.c. match	
CA6010	1.1	1.3	1.0	TRX1	complemer	thioredoxin (by homolo	orf19.7611	640	IPF643	CELL CYC	protein phosphatase activity
CA6011	1.1	1.2	1.0	CTM1	complemer	cytochrome c methyltra	orf19.7612	639	CaTRX1	CELL CYC	oxidoreductase activity
CA6012	1.1	1.4	1.0	HCR1	17655504..	putative translation init	orf19.7613	638	CaCTM1	PROTEIN I	transferase activity
CA6013	1.0	1.0	1.0	IPF635	complemer	unknown function	orf19.7614	635	CaHCR1	TRANSCR	translation regulator activity
CA6014	1.0	1.0	1.1	TRS31	17657803..	targeting complex (TRA	orf19.7615	634	IPF635	No significant S.c. match	
CA6015	1.1	1.0	1.0	ARD1	17658711..	protein N-acetyltransfe	orf19.7617	631	CaTRS31	CELLULAF	molecular_function unknown
CA6016	1.1	1.1	1.0	IPF630	complemer	unknown function	orf19.7618	630	CaARD1	Lipid fatty-a	transferase activity
CA6017	1.0	1.0	0.9	IPF629	17661145..	unknown function	orf19.7619	629	IPF630	UNCLASSI	chaperone activity
CA6018	1.1	1.1	1.1	IPF627	complemer	unknown function	orf19.7620	627	IPF629	UNCLASSIFIED PROTEINS	
CA6019	0.9	1.0	0.9	IPF625	17664248..	unknown function	orf19.7621	625	IPF627	TRANSCR	molecular_function unknown
CA6020	1.0	1.0	1.0	SPT3	complemer	transcription factor	orf19.7622	624	IPF625	UNCLASSI	molecular_function unknown
CA6021	0.9	1.0	0.9	BFR2	complemer	involved in protein tran	orf19.7624	621	CaSPT3	TRANSCR	transcription regulator activity
CA6022	1.1	0.8	1.5	IPF618	17670702..	GPI-anchored cell surf	orf19.7625	618	CaBFR2	CELLULAF	molecular_function unknown
CA6023	1.1	1.4	1.2	CDC33	17671599..	translation initiation fac	orf19.7626	616	IPF618	No significant S.c. match	
CA6024	0.8	0.7	0.9	IPF615	complemer	unknown function	orf19.7627	615	CaCDC33	PROTEIN S	translation regulator activity
CA6025	0.9	0.9	0.9	IPF614	17673364..	unknown function	orf19.7629	614	IPF615	CELL FATI	molecular_function unknown
CA6026	1.1	1.0	1.0	IPF610	complemer	unknown function	orf19.7631	610	IPF614	UNCLASSI	molecular_function unknown
CA6027	0.9	0.9	0.9	IPF609	17677515..	unknown function	orf19.7632	609	IPF610	UNCLASSI	DNA binding
CA6028	1.1	1.1	1.1	IPF607	complemer	unknown function	orf19.7634	607	IPF609	TRANSCRIPTION SUBCELLULAR LOCALISATION	
CA6029	0.9	1.0	1.0	DRS1	17682130..	ATP dependent RNA h	orf19.7635	603	IPF607	CELL CYC	molecular_function unknown
CA6030	1.1	1.0	1.1	YHB2	complemer	flavohemoprotein (by h	orf19.7637	599	CaDRS1	TRANSCR	RNA binding,helicase activity
CA6031	1.0	1.0	1.0	PRO1	17687109..	glutamate 5-kinase (by	orf19.7638	597	CaYHB2	CELL RESCUE DEFENSE AND VIRULENCE ""SUBCELLULAR LOCALISATION	
CA6032	1.0	1.0	1.0	IPF596	17688451..	similar to Saccharomy	orf19.7642	596	CaPRO1	Amino acid	transferase activity
CA6033	1.1	1.4	1.2	COQ2	complemer	para-hydroxybenzoate	orf19.7643	592	IPF596	PROTEIN I	molecular_function unknown
CA6034	1.1	1.0	1.2	APC11	complemer	subunit of the anaphas	orf19.7644	590	CaCOQ2	Metabolism	transporter activity
CA6035	1.0	1.1	1.0	BPL1	complemer	biotin holocarboxylase	orf19.7645	589	CaAPC11	CELL CYC	protein binding
CA6036	1.1	1.1	1.1	IPF585	17694470..	unknown function	orf19.7646	585	CaBPL1	Metabolism	ligase activity
CA6037	0.9	0.9	0.9	ERC4	17695567..	ethionine resistance pr	orf19.7648	582	IPF585	UNCLASSI	molecular_function unknown
CA6038	0.8	0.9	0.9	LTV1	17697084..	low-temperature viabili	orf19.7650	579	CaERC4	UNCLASSIFIED PROTEINS	
CA6039	1.0	0.9	1.0	CKA1	17698544..	casein kinase II, cataly	orf19.7652	576	CaLTV1	CELL RES	molecular_function unknown
CA6040	1.0	0.9	0.8	CPR6	17700062..	cyclophylin (by homolo	orf19.7654	574	CaCKA1	CELL CYC	protein kinase activity
CA6041	1.2	1.3	1.1	RPO21	17701754..	DNA-directed RNA pol	orf19.7655	572	CaCPR6	TRANSCR	chaperone activity
CA6042	1.0	1.0	1.0	IPF4924	17707077..	unknown function	orf19.7657	4924	CaRPO21	TRANSCR	nucleotidyltransferase activity
CA6043	0.9	0.8	1.0	RFC4	17707814..	DNA replication factor	orf19.7658	4926	IPF4924	TRANSCR	RNA binding
CA6044	1.0	0.9	1.0	IPF4928	complemer	similar to Saccharomy	orf19.7660	4928	CaRFC4	CELL CYC	DNA binding
CA6045	1.0	0.9	1.0	IPF4929	17710477..	similar to Saccharomy	orf19.7661	4929	IPF4928	CELLULAF	protein binding
CA6046	0.9	1.0	1.0	IPF4931	complemer	unknown function	orf19.7662	4931	IPF4929	CELL CYC	DNA binding,helicase activity
CA6047	1.0	1.1	1.0	IPF4933	17713493..	unknown function	orf19.7663	4933	IPF4931	TRANSPO	molecular_function unknown
CA6048	0.9	0.9	0.9	IPF4934	complemer	unknown function	orf19.7664	4934	IPF4933	CELL CYC	molecular_function unknown
CA6049	0.9	1.0	0.9	IPF4935	complemer	unknown function	orf19.7665	4935	IPF4934	TRANSCR	molecular_function unknown
CA6050	1.0	0.9	0.9	IPF4939	17715579..	similar to Saccharomy	orf19.7666	4939	IPF4935	UNCLASSI	molecular_function unknown
CA6051	1.0	1.1	1.0	IPF4940	complemer	unknown function	orf19.7667	4940	IPF4939	TRANSPORT FACILITATION	
CA6052	0.8	0.9	0.8	IPF4942	17718141..	similar to Saccharomy	orf19.7668	4942	IPF4940	C-compour	hydrolase activity
CA6053	1.2	1.1	1.0	IPF4949	complemer	unknown function	orf19.7670	4949	IPF4942	C-compour	hydrolase activity
CA6054	1.0	1.0	1.2	IPF4952	complemer	unknown function	orf19.7672	4952	IPF4949	UNCLASSI	molecular_function unknown
CA6055	1.1	1.0	1.1	SMD1	17723811..	snRNA-associated pro	orf19.7673	4953	IPF4952	UNCLASSI	molecular_function unknown
CA6056	1.1	0.8	1.8	IPF4955	complemer	similar to Saccharomy	orf19.7675	4955	CaSMD1	TRANSCRIPTION SUBCELLULAR LOCALISATION	
CA6057	2.4	3.4	1.5	IPF4959	complemer	D-xylulose reductase (	orf19.7676	4959	IPF4955	No significa	structural molecule activity

CA6058	1.0	1.5	0.9	ATP16	complemer	F1F0-ATPase comple›	orf19.7678	4961	IPF4959	C-compour	oxidoreductase activity
CA6059	1.0	1.1	1.0	CTA26	complemer	transcriptional activatic	orf19.7680	20034	CaATP16	ENERGY (	transporter activity
CA6060	1.1	1.1	1.0	IPF8301	17728176..	unknown function	orf19.6008	8301	CaCTA26	No significant S.c. match	
CA6061	1.1	0.9	1.0	IPF8302	17732246..	unknown function	orf19.6007	8302	IPF8301	UNCLASSI	molecular_function unknown
CA6062	1.0	1.1	1.0	IPF8307	17734108..	putative permease (by	orf19.6005	8307	IPF8302	Lipid fatty-acid and isoprenoid metabolism	
CA6063	1.0	0.8	0.9	IPF8311	17738610..	unknown function	orf19.6003	8311	IPF8307	C-compound and carbohydrate metabolism SUBCELLULAR LOCALISATION TRANSPORT FACILITATION	
CA6064	1.1	1.4	1.1	RPL81	17740884..	60S ribosomal protein	orf19.6002	8312	IPF8311	UNCLASSI	molecular_function unknown
CA6065	1.0	1.0	0.9	SAP3	complemer	secreted aspartyl prote	orf19.6001	8313	CaRPL81	PROTEIN !	structural molecule activity
CA6066	0.7	1.1	0.5	CDR1	complemer	multidrug resistance pr	orf19.6000	9739	CaSAP3	PROTEIN FATE [folding modification destination] ""Other virulence attributes	
CA6067	1.0	1.1	1.0	DYN1	17751047..	dynein heavy chain, cy	orf19.5999	19819	CaCDR1	Lipid fatty-a	transporter activity
CA6068	2.3	1.2	1.7	RPS19A.3	complemer	ribosomal protein S19.e, 3-prime e		4849	CaDYN1	CELL CYC	motor activity
CA6069	1.0	1.0	1.0	IPF4847	17766009..	unknown function	orf19.5995	4847	CaRPS19A	PROTEIN !	structural molecule activity
CA6070	0.9	1.0	1.1	IPF4842	complemer	similar to Saccharomy	orf19.5994	4842	IPF4847	UNCLASSI	peptidase activity
CA6071	1.0	1.0	1.0	IPF4835	complemer	zinc finger protein (by I	orf19.5992	4835	IPF4842	CELLULAF	hydrolase activity
CA6072	0.9	0.9	1.0	DBP10	17787135..	Putative ATP-depende	orf19.5991	6	IPF4835	Amino acid metabolism TRANSCRIPTION SUBCELLULAR LOCALISATION UNCLASSIFIED PROTEINS	
CA6073	0.9	1.0	1.1	HRP1	complemer	Nuclear polyadenylate	orf19.5989	9	CaDBP10	TRANSCR	RNA binding,helicase activity
CA6074	0.8	0.6	0.9	IPF11	17792199..	unknown function	orf19.5987	11	CaHRP1	TRANSCR	RNA binding
CA6075	0.9	0.9	1.0	THI4	17793918..	Thiazole biosynthetic e	orf19.5986	14	IPF11	UNCLASSI	transferase activity
CA6076	1.0	1.1	1.1	PAC10.3	17795269..	Non-native Actin Bindi	orf19.5985	15	CaTHI4	Metabolism	molecular_function unknown
CA6077	1.0	1.0	1.1	IPF16	complemer	unknown function	orf19.5984	16	CaPAC10.3	CELL CYC	protein binding
CA6079	0.8	1.5	1.3	RPL18.EX(	17797253..	Ribosomal Protein RPI	orf19.5982	20	IPF16	UNCLASSI	molecular_function unknown
CA6080	1.2	1.1	1.1	IPF21	17798643..	unknown function	orf19.5980	21	CaRPL18.e	PROTEIN !	structural molecule activity
CA6081	0.8	0.7	1.0	IPF24	complemer	reductase (by homolog	orf19.5978	24	IPF21	UNCLASSIFIED PROTEINS	
CA6082	1.0	1.0	1.0	CEM1	complemer	3-oxoacyl-[acyl-carrier-	orf19.5977	25	IPF24	UNCLASSIFIED PROTEINS	
CA6083	0.9	0.9	0.9	IPF26	complemer	unknown function	orf19.5976	26	CaCEM1	Lipid fatty-a	transferase activity
CA6084	0.6	0.7	0.4	IPF29	complemer	zinc finger protein (by I	orf19.5975	29	IPF26	UNCLASSI	molecular_function unknown
CA6085	1.0	1.0	0.9	IPF32	17809845..	similar to Saccharomy	orf19.5974	32	IPF29	C-compound and carbohydrate metabolism TRANSCRIPTION SUBCELLULAR LOCALISATION	
CA6086	0.9	1.2	1.0	PHB2	complemer	Mitochondrial protein,	orf19.5973	33	IPF32	SUBCELLU	molecular_function unknown
CA6087	1.0	1.0	1.1	YHV1	complemer	unknown function	orf19.5971	36	CaPHB2	CELL CYC	molecular_function unknown
CA6088	1.0	0.9	1.0	HPR5	17816175..	ATP-dependent DNA I	orf19.5970	37	CaYHV1	UNCLASSI	molecular_function unknown
CA6089	1.0	1.0	1.1	RDI1	complemer	Rho GDP dissociation	orf19.5968	40	CaHPR5	CELL CYC	DNA binding,helicase activity
CA6090	1.0	0.9	1.0	IPF44	complemer	unknown function	orf19.5967	44	CaRDI1	CELL FATE	signal transducer activity
CA6091	0.8	1.0	0.8	UFD2	17822692..	Ubiquitin fusion degrac	orf19.5965	46	IPF44	UNCLASSI	molecular_function unknown
CA6092	1.9	2.6	1.6	RPL35.3	complemer	Ribosomal protein L35A, 3-prime e		47	CaUFD2	PROTEIN FATE [folding modification destination]	
CA6093	1.7	1.8	1.3	ARF22	complemer	GTP-binding protein of	orf19.5964	50	CaRPL35.3	PROTEIN !	structural molecule activity
CA6094	1.0	1.0	1.0	IPF53	complemer	unknown function	orf19.5963	53	CaARF22	PROTEIN I	hydrolase activity
CA6095	1.0	0.9	1.0	SNF3	17831851..	High affinity glucose tr	orf19.5962	55	IPF53	UNCLASSI	molecular_function unknown
CA6096	0.5	0.5	0.6	IPF56	17834497..	similar to Saccharomy	orf19.5961	56	CaSNF3	C-compour	signal transducer activity
CA6097	2.4	2.1	1.3	NCE102	17837654..	secretion of proteins th	orf19.5960	59	IPF56	PROTEIN I	molecular_function unknown
CA6098	1.0	1.1	1.0	IPF61	complemer	unknown function	orf19.5959	61	CaNCE102	CELLULAF	molecular_function unknown
CA6099	1.1	0.9	1.0	CDR2	complemer	Candida albicans drug	orf19.5958	63	IPF61	PROTEIN !	RNA binding
CA6100	1.2	1.2	1.4	IPF66	complemer	unknown function	orf19.5956	66	CaCDR2	Lipid fatty-acid and isoprenoid metabolism """"CELL RESCUE DEFENSE AND VIRULENCE ""REGULATION OF/INTERACTION WITH CE	
CA6101	1.0	1.0	1.0	SPO70.3F	complemer	involved in meiosis and sporulatior		68	IPF66	UNCLASSI	molecular_function unknown
CA6102	1.1	1.0	1.1	SPO70.5F	complemer	involved in meiosis anc	orf19.5954	70	CaSPO70.3	CELL CYCLE AND DNA PROCESSING ""PROTEIN FATE [folding modification destination] ""SUBCELLULAR LOCALISATION	
CA6103	0.9	0.9	1.0	SFP1	complemer	zinc finger protein (by I	orf19.5953	72	CaSPO70.5	CELL FATE	molecular_function unknown
CA6104	1.0	1.0	1.0	IPF65	17856243..	unknown function	orf19.5952	75	CaSFP1	CELL CYC	DNA binding,transcription regulator activity
CA6105	0.9	1.0	0.9	FAS2.5F	17858538..	fatty-acyl-CoA synthas	orf19.5951	76	IPF65	UNCLASSIFIED PROTEINS	
CA6106	1.5	1.2	1.0	FAS2.53F	17860953..	fatty-acyl-CoA synthase, alpha cha		77	CaFAS2.5f	Lipid fatty-acid and isoprenoid metabolism """"PROTEIN FATE [folding modification destination] ""SUBCELLULAR LOCALISATION	
CA6107	1.3	1.6	1.4	FAS2.3F	17861433..	fatty-acyl-CoA synthas	orf19.5949	80	CaFAS2.53	Lipid fatty-acid and isoprenoid metabolism """"PROTEIN FATE [folding modification destination] ""SUBCELLULAR LOCALISATION	
CA6108	1.1	1.3	1.0	SEC7	17864532..	Guanine nucleotide ex	orf19.5947	85	CaFAS2.3f	Lipid fatty-a	oxidoreductase activity,transferase activity
CA6109	1.0	1.8	0.9	IPF89.3	17870899..	unknown function, 3-prime end		89	CaSEC7	CELLULAF	enzyme regulator activity
CA6110	0.9	0.8	0.9	IPF91	17872156..	unknown function	orf19.5943	91	IPF89.3	C-compour	DNA binding
CA6111	1.0	1.0	1.1	IPF96	complemer	C3HC4 type zinc finge	orf19.5942	96	IPF91	UNCLASSI	molecular_function unknown
CA6112	1.0	1.2	1.0	IPF97	17875727..	unknown function	orf19.5941	97	IPF96	UNCLASSI	molecular_function unknown
CA6113	1.0	1.0	1.0	IPF100.3	17877348..	zinc finger protein, 3-pr	orf19.5940	100	IPF97	CELLULAF	molecular_function unknown
CA6114	1.0	1.0	1.0	SEN1	17879931..	positive effector of tRN	orf19.5938	110	IPF100.3	Amino acid metabolism TRANSCRIPTION SUBCELLULAR LOCALISATION UNCLASSIFIED PROTEINS	
CA6115	1.0	0.9	0.9	IPF112	complemer	unknown function	orf19.5935	112	CaSEN1	TRANSCR	RNA binding,helicase activity
CA6116	1.0	1.0	1.0	TOP3	17888645..	DNA topoisomerase III	orf19.5934	116	IPF112	CELL CYCLE AND DNA PROCESSING SUBCELLULAR LOCALISATION	
CA6117	0.9	0.8	1.0	IPF122	17893301..	unknown function	orf19.5933	122	CaTOP3	CELL CYC	isomerase activity
CA6118	1.1	1.0	1.1	IPF126	17895351..	unknown function	orf19.5932	126	IPF122	No significant S.c. match	
CA6119	1.1	1.0	1.1	ARV1	complemer	involved in sterol uptak	orf19.5931	128	IPF126	UNCLASSI	molecular_function unknown
CA6120	1.0	1.0	0.9	IPF132	complemer	unknown function	orf19.5930	132	CaARV1	SUBCELLU	molecular_function unknown
CA6121	1.0	1.1	1.1	IPF33	17900332..	unknown function	orf19.5929	133	IPF132	TRANSCRIPTION CELLULAR TRANSPORT AND TRANSPORT MECHANISMS SUBCELLULAR LOCALISATION	
CA6122	2.4	1.3	1.3	RPP2B	complemer	acidic ribosomal protei	orf19.5928	134	IPF33	No significant S.c. match	
CA6123	1.4	1.3	1.5	RPS15.3	17902660..	40S ribosomal protein	orf19.5927	135	CaRPP2B	PROTEIN !	structural molecule activity
CA6124	1.0	1.1	1.0	ARG11	17903567..	mitochondrial amino ac	orf19.5926	137	CaRPS15.3	PROTEIN !	structural molecule activity
CA6125	0.8	0.9	0.8	IPF138	complemer	unknown function	orf19.5925	138	CaARG11	Amino acid	transporter activity
CA6126	0.9	1.0	1.0	IPF143	complemer	unknown function	orf19.5924	143	IPF138	UNCLASSI	molecular_function unknown
CA6127	0.9	0.9	0.8	IPF149	complemer	peroxisomal membran	orf19.5921	149	IPF143	TRANSCRIPTION ""CELL RESCUE DEFENSE AND VIRULENCE ""SUBCELLULAR LOCALISATION	
CA6128	1.0	0.9	1.0	IPF152	complemer	unknown function	orf19.5919	152	IPF149	CELLULAR TRANSPORT AND TRANSPORT MECHANISMS TRANSPORT FACILITATION	
CA6129	1.2	1.0	1.1	YRA1.EXO	complemer	RNA annealing protein, exon 2 (by		155	IPF152	UNCLASSI	molecular_function unknown
CA6131	1.1	1.1	1.0	IPF4369	complemer	similar to Saccharomy	orf19.5917	4369	CaYRA1.e›	TRANSCR	molecular_function unknown

CA6132	1.0	1.0	1.0	DUR35.5F	17927011..	Urea transport protein,	orf19.5916	4365	IPF4369	TRANSCRIPTION	
CA6133	0.9	0.9	0.9	DUR35.3F	17927715..	Urea transport protein,	orf19.5915	4364	CaDUR35.	REGULATION OF/INTERACTION WITH CELLULAR ENVIRONMENT SUBCELLULAR LOCALISATION TRANSPORT FACILITATION	
CA6134	1.0	1.0	1.0	MAK21	complemer	Ribosome biogenesis		orf19.5912	4362	CaDUR35.	REGULATION OF/INTERACTION WITH CELLULAR ENVIRONMENT SUBCELLULAR LOCALISATION TRANSPORT FACILITATION	
CA6135	1.0	1.0	1.0	CMK1	17933003..	Ca2+/calmodulin-depe	orf19.5911	4358	CaMAK21	PROTEIN	molecular_function unknown
CA6137	1.0	0.9	1.0	IPF4356	complemer	unknown function	orf19.5910	4356	CaCMK1	CELLULAR COMMUNICATION/SIGNAL TRANSDUCTION MECHANISM CELL FATE SUBCELLULAR LOCALISATION	
CA6138	1.2	1.3	1.0	IPF4351	17942827..	unknown function	orf19.5908	4351	IPF4356	TRANSCR	molecular_function unknown
CA6139	0.9	1.0	0.9	ADE2	17949698..	phosphoribosylaminoir	orf19.5906	1883	IPF4351	TRANSCR	transcription regulator activity
CA6140	1.0	1.0	1.0	IPF1882	complemer	unknown function	orf19.5905	1882	CaADE2	Nucleotide	lyase activity
CA6141	2.3	3.1	1.6	RPL19A.3	17952958..	Ribosomal protein L19	orf19.5904	1881	IPF1882	CELL FATI	molecular_function unknown
CA6142	1.0	1.0	1.0	IPF1879	complemer	unknown function	orf19.5903	1879	CaRPL19A	PROTEIN	structural molecule activity
CA6143	1.1	1.0	0.9	IPF1873	17959419..	putative GTP-binding	orf19.5902	1873	IPF1879	SUBCELLU	molecular_function unknown
CA6144	1.0	1.1	1.1	PKC1	complemer	Ser/thr protein kinase	orf19.5901	1872	IPF1873	Nucleotide metabolism C-compound and carbohydrate metabolism CELL CYCLE AND DNA PROCESSING CELLULAR COMMUNICATIC	
CA6145	1.0	1.0	1.0	IPF1869	complemer	unknown function	orf19.5897	1869	CaPKC1	CELL CYC	protein kinase activity
CA6146	1.1	1.0	1.0	IPF1863	17968356..	unknown function	orf19.5896	1863	IPF1869	UNCLASSI	molecular_function unknown
CA6147	1.1	0.9	1.0	IPF1862.5F	17969397..	unknown function, 5-pr	orf19.5895	1862	IPF1863	ENERGY	molecular_function unknown
CA6148	1.1	1.1	1.1	IPF1862.3F	17969860..	unknown function, 3-pr	orf19.5894	1861	IPF1862.5f	UNCLASSIFIED PROTEINS	
CA6149	0.9	0.9	0.7	RIP1	complemer	Ubiquinol cytochrome-	orf19.5893	1859	IPF1862.3f	UNCLASSI	molecular_function unknown
CA6150	1.0	1.2	0.9	IPF1857	complemer	similar to Saccharomy	orf19.5892	1857	CARIP1	ENERGY	transporter activity,oxidoreductase activity
CA6151	0.8	0.8	0.9	IPF1853	17975894..	unknown function	orf19.5890	1853	IPF1857	TRANSCR	ligase activity
CA6152	1.0	0.9	1.1	NUP85	17979392..	Nuclear pore protein (t	orf19.5887	1850	IPF1853	No significant S.c. match	
CA6153	1.0	1.2	1.0	CUP5	complemer	Vacuolar H+-ATPase (	orf19.5886	1849	CaNUP85	TRANSCR	structural molecule activity
CA6154	0.8	0.8	0.9	IPF1848	17983285..	similar to Saccharomy	orf19.5885	1848	CaCUP5	CELLULAR TRANSPORT AND TRANSPORT MECHANISMS ""CELL RESCUE DEFENSE AND VIRULENCE ""REGULATION OF/INTER	
CA6155	0.9	0.9	1.0	IPF1846	17983986..	unknown function	orf19.5884	1846	IPF1848	TRANSCR	RNA binding
CA6156	0.9	0.9	0.9	GEF1.3F	complemer	Voltage-gated chloride	orf19.5881	1844	IPF1846	UNCLASSI	molecular_function unknown
CA6157	1.0	0.9	1.0	GEF1.5F	complemer	Voltage-gated chloride	orf19.5880	1841	CaGEF1.3f	CELLULAR TRANSPORT AND TRANSPORT MECHANISMS REGULATION OF/INTERACTION WITH CELLULAR ENVIRONMENT SUB	
CA6158	1.0	0.9	0.9	IPF1839	17990466..	putative 1-Acyl dihydrc	orf19.5879	1839	CaGEF1.5f	CELLULAF	transporter activity
CA6159	1.0	1.1	1.0	IPF1837	complemer	unknown function	orf19.5877	1837	IPF1839	C-compound and carbohydrate metabolism CELL FATE	
CA6160	1.2	1.2	1.0	IPF1835	17993845..	unknown function	orf19.5876	1835	IPF1837	C-compour	transferase activity
CA6161	1.0	1.3	1.0	IPF1834	complemer	probable syntaxin (by	orf19.5875	1834	IPF1835	No significant S.c. match	
CA6162	1.0	0.9	1.0	IPF1833	17995827..	similar to opaque phas	orf19.5874	1833	IPF1834	PROTEIN I	transporter activity
CA6163	1.0	0.9	1.0	POL1	17997165..	DNA-directed DNA pol	orf19.5873	1832	IPF1833	No significant S.c. match	
CA6164	1.1	1.0	1.0	SNF5.5F	18001869..	Component of SWI/SN	orf19.5872	14342	CaPOL1	CELL CYC	nucleotidyltransferase activity
CA6165	0.4	0.3	0.8	SNF5.3F	18002327..	Component of SWI/SN	orf19.5871	14343	CaSNF5.5f	No significant S.c. match	
CA6166	1.2	1.1	1.1	CTP1	18007015..	Citrate transport protei	orf19.1329	18048	CaSNF5.3f	C-compour	transcription regulator activity
									CaCTP1	C-compour	transporter activity
