# Supplementary material for: Genome-wide analysis of Candida albicans gene expression patterns during infection of the mammalian kidney
Source: Fungal Genet Biol. 2009 Feb;46(2):210–9. doi: 10.1016/j.fgb.2008.10.012 (PMC2698078; doi:10.1016/j.fgb.2008.10.012)
Supplement: Supplementary Data 3 [file mmc3.pdf]

RPMI Sucrose - 15 mins (UP genes)

			Rep1	Rep2	Rep3	
IPF9550	CA4570	orf19.6882	5.36	4.15	4.58	similar to Saccharomyces cerevisiae Osm1p osmotic growth protein
RNH1	CA0277	orf19.13057	5.23	4.16	4.77	ribonuclease H (by homology)
IPF6629	CA4127	orf19.2762	5.18	5.36	5.53	unknown function
PGI1	CA3559	orf19.3888	4.43	4.96	4.17	Glucose-6-phosphate isomerase
IFD1	CA0840	orf19.8650	4.24	3.50	3.62	Putative aryl-alcohol dehydrogenase (by homology)

RPMI Sucrose - 15 mins (DOWN genes)

IPF4764	CA3938	orf19.9556	0.35	0.33	0.41	unknown Function
CDR1	CA6066	orf19.6000	0.31	0.24	0.28	multidrug resistance protein (by homology)
SSA4	CA1230	orf19.4980	0.28	0.36	0.31	cahsp70 mRNA for heat shock
ATP2	CA4362	orf19.5653	0.26	0.25	0.27	F1F0-ATPase complex, F1 beta subunit (by homology)
ATP1	CA4457	orf19.6854	0.17	0.22	0.19	F1F0-ATPase complex, F1 alpha subunit, exon 3
CIT1	CA3909	orf19.4393	0.05	0.03	0.07	Citrate synthase, exon 2
FRP2	CA3153	orf19.6169	0.03	0.02	0.04	related to S. cerevisiae Fun34p

RPMI Sucrose - 30 mins (UP genes)

None						*none*

RPMI Sucrose - 30 mins (DOWN genes)

IPF4764	CA3938	orf19.9556	0.46	0.47	0.44	unknown Function
IPF10595	CA2362	orf19.1287	0.45	0.45	0.49	unknown function
