# Supplementary material for: Genome-wide analysis of Candida albicans gene expression patterns during infection of the mammalian kidney
Source: Fungal Genet Biol. 2009 Feb;46(2):210–9. doi: 10.1016/j.fgb.2008.10.012 (PMC2698078; doi:10.1016/j.fgb.2008.10.012)
Supplement: Supplementary Data 5 [file mmc5.pdf]

UP - SC5314 in vivo (58 genes)

		RK1	RK2	RK3	RK4	
DIP51	CA2204	24.4	3.9	11.6	1.5	dicarboxylic amino acid permease, 5-prime end (by homology)
GAP2	CA5039	20.9	7.0	47.6	3.3	transporter activity
FRP2	CA3153	16.5	5.6	34.1	4.5	Related to S. cerevisiae Fun34p
IPF14618	orf19.6079	16.1	3.0	7.5	1.6	unknown function
FRP3	CA0415	14.6	7.9	26.4	4.5	Related to S. cerevisiae Fun34p
PHO89	CA5160	10.4	6.9	19.5	1.8	Na+-coupled phosphate transport (by homology)
ACS1	CA0848	10.1	5.1	47.9	4.0	acetyl-coenzyme-A synthetase (by homology)
CTA1	CA3011	9.6	3.9	5.3	1.2	catalase A, peroxisomal(by homology)
CAN2	CA1191	8.9	7.2	6.1	2.5	amino acid permease (by homology)
FAA4	CA5992	8.8	5.9	5.2	1.4	long-chain fatty acid--CoA ligase and synthetase 4 (by homology)
ACO1	CA3546	8.7	5.5	4.9	1.9	aconitate hydratase (by homology)
IPF17652	orf19.6078	7.6	4.8	9.6	1.5	reverse transcriptase, 3-prime end (by homology)
IPF10196	orf19.2752	7.5	3.0	3.6	1.3	unknown function
IPF19142	orf19.11467	7.0	2.7	3.1	1.0	unknown function
MLS1	CA4748	6.2	4.5	3.7	1.6	malate synthase
PHO84	CA0083	6.0	5.3	6.8	2.5	high-affinity inorganic phosphate/H+ symporter by homology
ALS2	CA0413	5.8	3.2	3.2	1.5	agglutinin-like protein, 3-prime end
IPF15781	orf19.8335	5.5	5.7	3.3	1.8	unknown function
POX4	CA1572	5.3	4.0	3.1	1.6	peroxisomal fatty acyl-CoA oxidase (by homology)
IPF3912	orf19.6514	5.2	4.6	4.7	1.3	unknown function
IPF14282	orf19.2296	5.1	3.5	2.6	1.1	Similar to mucin proteins (by homology)
ALS4	CA1528	5.1	3.3	3.2	1.6	agglutinin-like protein, 3-prime end
CIT1	CA3909	4.9	2.6	7.1	3.5	Citrate synthase, exon 2
DIP51	CA2203	4.7	4.9	7.5	1.6	dicarboxylic amino acid permease, 3-prime end (by homology)
FRE30	CA3416	4.6	2.0	2.7	1.3	Strong similarity to ferric reductase Fre2p, 3-prime end (by homology)
IPF5625	orf19.7112	4.6	3.4	2.9	1.9	unknown function
IPF12540	orf19.8824	4.5	2.4	3.2	2.3	unknown function
ALS1	CA0316	4.4	4.7	3.1	1.3	agglutinin-like protein, 3-prime end
IPF9211	orf19.3713	4.4	2.0	2.2	1.5	unknown function, 3-prime end
PRB1	CA5322	4.4	3.6	2.8	2.2	Protease B, vacuolar (by homology)
ENA22	CA4929	4.2	4.4	2.0	1.8	P-type ATPase involved in Na+ efflux (by homology)
RIM11	CA1465	4.1	2.6	2.3	1.2	Ser/thr protein kinase (by homology)
IPF2615	orf19.6688	4.0	4.8	2.0	1.4	unknown function
IFC4	CA0442	4.0	3.9	2.4	1.8	unknown function
IPF7715	orf19.8300	3.9	2.8	2.9	1.2	unknown function
IPF4820	orf19.10861	3.9	6.6	3.3	1.2	putativecomplex I intermediate associated protein CIA30 (by homology)

CDR11	CA0610	3.4	2.4	2.6	1.1	multidrug resistance protein, 5-prime end (by homology)
AMS1	CA4123	3.3	2.4	2.0	1.6	alpha-mannosidase (by homology)
IPF9496	orf19.2809	3.3	4.1	2.4	1.3	carnitine O-acetyltransferase (by homology)
GDH2	CA1775	3.2	2.2	2.1	1.1	NAD-specific glutamate dehydrogenase (NAD) (by homology)
CDR11	CA0609	3.0	3.9	2.0	1.8	multidrug resistance protein, 3-prime end (by homology)
SDH12	CA2470	2.8	2.1	2.2	1.1	Succinate dehydrogenase (by homology)
IPF15119	orf19.9109	2.8	2.5	1.6	1.3	unknown function
PHO84	CA1782	2.8	3.4	2.6	2.0	Inorganic phosphate transport protein, 3-prime end (by homology)
IPF3937	orf19.8487	2.6	2.4	2.0	0.9	Unknown function
IPF17190	orf19.11350	2.5	2.1	2.6	1.3	unknown function
IPF9167	orf19.2737	2.5	2.7	2.0	1.2	unknown function
IPF11503	orf19.3902	2.4	3.2	3.5	1.3	unknown function
CDR1	CA6066	2.4	3.8	2.7	1.8	multidrug resistance protein (by homology)
IPF20056	orf19.2659	2.4	1.3	6.6	3.1	unknown function
IPF13398	orf19.7708	2.3	2.1	2.8	0.8	protein kinase (by homology)
IPF5681	orf19.12417	2.3	2.3	2.3	1.0	unknown function
IPF10153	orf19.2170	2.2	2.5	3.6	1.1	membrane transporter by homology
IPF15013	orf19.13071	2.2	3.4	2.3	1.1	pyruvate decarboxylase regulatory protein (by homology)
IPF12799	orf19.2515	2.1	4.1	3.7	1.8	unknown function
AUT7	CA3802	2.1	3.0	2.6	1.3	microtubule-associated protein essential for autophagy, exon 2 (by homology)
KSP1	CA1288	2.0	2.1	2.2	0.9	SERINE/THREONINE-PROTEIN KINASE by homology
ZRT2	CA3160	1.4	3.7	2.2	3.2	zinc transport protein (by homology)
