# Supplementary material for: Genome-wide analysis of Candida albicans gene expression patterns during infection of the mammalian kidney
Source: Fungal Genet Biol. 2009 Feb;46(2):210–9. doi: 10.1016/j.fgb.2008.10.012 (PMC2698078; doi:10.1016/j.fgb.2008.10.012)
Supplement: Supplementary Data 6 [file mmc6.pdf]

DOWN - SC5314 in vivo (49 genes)

		RK1	RK2	RK3	RK4	
HXT62	CA1067	0.07	0.14	0.12	0.19	sugar transporter
HYR1	CA1576	0.10	0.01	0.26	0.13	hyphally regulated protein
SOD5	CA4836	0.11	0.12	0.10	0.68	Similar to superoxide dismutase (by homology)
IPF1222	orf19.2060	0.11	0.12	0.10	0.68	Similar to superoxide dismutase (by homology)
IPF2431	orf19.7417	0.11	0.27	0.20	1.02	similar to S. cerevisiae Tsa1p (by homology)
ECE1	CA1402	0.11	0.07	0.02	0.07	Cell Elongation Protein
RNR21	CA4155	0.12	0.11	0.16	0.55	ribonucleoside-diphosphate reductase (by homology)
HHF22	CA3372	0.13	0.18	0.35	0.92	histone H4 (by homology)
HTA1	CA4696	0.14	0.25	0.36	0.92	Histone H2A (by homology)
PDA1	CA4412	0.15	0.29	0.24	0.72	Pyruvate dehydrogenase alpha chain (by homology)
CDC19	CA3483	0.16	0.36	0.28	0.84	pyruvate kinase (by homology)
RPL18	CA6079	0.16	0.29	0.34	0.72	Ribosomal Protein RPL18B (large subunit), exon 2 (by homology)
ALS10	CA0448	0.16	0.06	0.07	0.10	agglutinin like protein
RPS13	CA1304	0.16	0.41	0.49	0.90	ribosomal protein, 3-prime end (by homology)
IPF12297	orf19.3117	0.16	0.06	0.11	0.39	mycelial surface antigen (by homology)
SEC61	CA3197	0.17	0.45	0.59	1.06	ER protein-translocation complex subunit
ERG251	CA0875	0.17	0.18	0.26	0.82	C-4 sterol methyl oxidase (by homology)
IPF16646	orf19.1782	0.17	0.60	0.26	0.95	unknown function
IPF3634	orf19.6720	0.17	0.50	0.37	0.88	unknown function
ADH1	CA4765	0.19	0.56	0.32	0.21	alcohol dehydrogenase (by homology)
IPF10662	orf19.5760	0.20	0.25	0.32	0.39	unknown function
CFL2	CA3461	0.21	0.46	0.33	1.22	ferric reductase (by homology)
TUB2.3	CA4897	0.21	0.09	0.27	0.39	Beta-tubulin, 3-prime end
IPF7940	orf19.6608	0.23	0.52	0.43	1.16	unknown function
IPF15870	orf19.2685	0.23	0.06	0.06	0.40	unknown function
IPF20008	orf19.2767	0.23	0.50	0.14	0.17	unknown function
BEL1	CA4589	0.24	0.28	0.59	0.48	protein of the 40S ribosomal subunit (by homology)
SUN41	CA0883	0.25	0.11	0.11	0.14	Putative cell wall beta-glucosidase (by homology)
RPS21	CA1715	0.26	0.34	0.43	0.79	ribosomal protein (by homology)
GAP5	CA4667	0.26	0.31	0.33	0.81	General amino acid permease (by homology)
HXK2	CA0127	0.27	0.28	0.36	0.54	hexokinase II, 3-prime end (by homology)
IPF277	orf19.3268	0.27	0.40	0.43	0.94	human histamine-releasing factor homolog (by homology)
IPF14171	orf19.3149	0.28	0.75	0.46	0.37	unknown function
TUB1	CA5546	0.29	0.08	0.36	0.77	Alpha-1 tubulin, 3-prime end
CLN21	CA1582	0.30	0.27	0.39	0.99	G1 cyclin (by homology)
HXT5	CA1069	0.30	0.45	0.58	0.29	sugar transporter, 3-prime end

IPF1009	orf19.4590	0.31	0.26	0.43	0.91	Weak similarity to S. cerevisiae RFX1
PHR1	CA4857	0.36	0.26	0.23	0.42	GPI-anchored pH responsive glycosyl transferase
GND1	CA5239	0.36	0.24	0.44	0.65	6-phosphogluconate dehydrogenase
IPF2050	orf19.5265	0.37	0.47	0.42	0.71	similar to S.s cerevisiae Kip1p (by homology)
FET34	CA1431	0.37	0.20	0.25	0.79	iron transport multicopper oxidase, 3-prime end (by homology)
ACH1	CA0345	0.38	0.32	0.38	0.99	acetyl-coenzyme-A hydrolase (by homology)
IPF20169	orf19.5674	0.39	0.28	0.28	0.89	unknown function
LSC1	CA0791	0.43	0.19	0.32	0.71	succinate-CoA ligase / synthetase (by homology)
RBT5	CA2558	0.43	0.56	0.32	0.30	repressed by TUP1 protein 5
ERG3	CA1956	0.43	0.20	0.46	0.85	C5,6 desaturase
HXT61	CA1070	0.43	0.38	0.78	0.46	sugar transporter
RPL2	CA3307	0.44	0.37	0.44	0.64	ribosomal protein L8, 3-prime end (by homology)
HGT11	CA1506	1.37	0.28	0.46	0.44	hexose transporter
