# Supplementary material for: Genome-wide analysis of Candida albicans gene expression patterns during infection of the mammalian kidney
Source: Fungal Genet Biol. 2009 Feb;46(2):210–9. doi: 10.1016/j.fgb.2008.10.012 (PMC2698078; doi:10.1016/j.fgb.2008.10.012)
Supplement: Supplementary Data 8 [file mmc8.pdf]

UP - CAI4 in vivo (4 genes)

		Rabbit 1		Rabbit 2		Rabbit 3		
		RK1A	RK1B	RK2A	RK2B	RK3A	RK3B	
HSP12	CA0627	2.29	4.56	2.68	2.75	1.48	1.82	Heat shock protein (by homology)
DDR48	CA4336	1.20	1.20	4.44	5.43	2.21	3.22	stress protein (by homology)
PDC11	CA2474	1.13	0.60	2.86	4.01	2.34	3.53	Pyruvate decarboxylase (by homology)
GPM1	CA4671	0.98	0.65	2.71	3.50	2.38	2.20	phosphoglycerate mutase (by homology)
