# Supplementary material for: Genome-wide analysis of Candida albicans gene expression patterns during infection of the mammalian kidney
Source: Fungal Genet Biol. 2009 Feb;46(2):210–9. doi: 10.1016/j.fgb.2008.10.012 (PMC2698078; doi:10.1016/j.fgb.2008.10.012)
Supplement: Supplementary Data 9 [file mmc9.pdf]

DOWN - CAI4 in vivo (5 genes)

		Rabbit 1		Rabbit 2		Rabbit 3		
		RK1A	RK1B	RK2A	RK2B	RK3A	RK3B	
PCK1	CA5857	1.08	0.48	0.38	0.18	0.55	0.49	phosphoenolpyruvate carboxykinase
IPF8762	orf19.822	1.05	0.59	0.25	0.33	0.39	0.11	unknown function
SOD5	CA4836	0.77	0.15	0.10	0.06	0.16	0.06	Similar to superoxide dismutase (by homology)
ADH1	CA4765	0.12	0.33	0.56	0.39	0.22	0.20	alcohol dehydrogenase (by homology)
ECE1	CA1402	0.10	0.22	0.48	0.27	0.29	0.19	Cell Elongation Protein
